# Supplementary material for: Plastome structure and adaptive evolution of Calanthe s.l. species
Source: PeerJ. 2020 Oct 13;8:e10051. doi: 10.7717/peerj.10051 (PMC7566753; doi:10.7717/peerj.10051)
Supplement: Supplemental Information 1 [file peerj-08-10051-s001.docx]

### **Table S1** Information of plastome used in this study.

| **Organism Name** | **Tribe** | **Raw reads** | **Plastid reads** | **Coverages (X)** | **Accession No** | **Vourcher specimens number or DNA number** |
| --- | --- | --- | --- | --- | --- | --- |
| ***Calanthe davidii*** | **Collabieae** | **35,093,308** | **617,312** | **93.3** | **MN708353** | **chenyq005** |
| ***Cephalantheropsis obcordata*** | **Collabieae** | **35,513,970** | **350,630** | **65.6** | **MN708351** | **NOCC5762** |
| ***Phaius tankervilliae*** | **Collabieae** | **33,316,944** | **687,476** | **96.6** | **MN708349** | **NOCC6632** |
| ***Preptanthe rubens*** | **Collabieae** | **37,137,198** | **120,216** | **16.1** | **MN708352** | **chenyq010** |
| ***Styloglossum lyroglossa*** | **Collabieae** | **31,047,326** | **361,698** | **51.3** | **MN708350** | **NOCC6438** |
| *Calanthe delavayi* | Collabieae |  |  |  | MK388860 |  |
| *Calanthe triplicata* | Collabieae |  |  |  | KF753635 |  |
| **Outgroup** |  |  |  |  |  |  |
| *Bletilla striata* | Arethuseae |  |  |  | KT588924.1 |  |
