# Supplementary material for: Plastome structure and adaptive evolution of Calanthe s.l. species
Source: PeerJ. 2020 Oct 13;8:e10051. doi: 10.7717/peerj.10051 (PMC7566753; doi:10.7717/peerj.10051)
Supplement: Supplemental Information 2 [file peerj-08-10051-s002.docx]

### **Table S2** Information of plastomes used as reference in this study.

| Organism Name | Subfamily | Tribe | Accession No |
| --- | --- | --- | --- |
| *Dendrobium primulinum* | Epidendroideae | Malaxideae | LC192810.1 |
| *Dendrobium aphyllum* |  |  | LC192953.1 |
| *Dendrobium brymerianum* |  |  | LC192954.1 |
| *Dendrobium denneanum* |  |  | LC192955.1 |
| *Dendrobium devonianum* |  |  | LC192956.1 |
| *Dendrobium falconeri* |  |  | LC192957.1 |
| *Dendrobium gratiosissimum* |  |  | LC192958.1 |
| *Dendrobium hercoglossum* |  |  | LC192959.1 |
| *Dendrobium wardianum* |  |  | LC192961.1 |
| *Dendrobium wilsonii* |  |  | LC193508.1 |
| *Dendrobium crepidatum* |  |  | LC193509.1 |
| *Phalaenopsis aphrodite subsp. formosana* |  | Vandeae | NC007499.1 |
| *Phalaenopsis equestris* |  |  | NC017609.1 |
| *Phalaenopsis* hybrid cultivar *Tiny Star* |  |  | NC025593.1 |
| *Bletilla striata* |  | Arethuseae | NC028422.1 |
| *Bletilla ochracea* |  |  | NC029483.1 |
| *Calanthe davidii* |  | Collabieae | MG925365.1 |
| *Calanthe triplicata* |  |  | KF753635.1 |
