# Supplementary material for: Plastome structure and adaptive evolution of Calanthe s.l. species
Source: PeerJ. 2020 Oct 13;8:e10051. doi: 10.7717/peerj.10051 (PMC7566753; doi:10.7717/peerj.10051)
Supplement: Supplemental Information 3 [file peerj-08-10051-s003.docx]

### **Table S3** Information of different region in aligned seven plastome sequences.

| Region | Number of sites | Number of variable sites | Number of informative sites | Nucleotide diversity |
| --- | --- | --- | --- | --- |
| Large single copy region | 91025 | 4689 (5.15%) | 1087 (1.19%) | 0.0155 |
| Small single copy region | 18460 | 998 (5.41%) | 211 (1.14%) | 0.0187 |
| Inverted repeat region | 26497 | 320 (1.21%) | 56 (0.21%) | 0.0036 |
| Plastome Alignment length | 162479 | 6327 (3.84%) | 1410 (0.86%) | 0.0128 |
