# Supplementary material for: Plastome structure and adaptive evolution of Calanthe s.l. species
Source: PeerJ. 2020 Oct 13;8:e10051. doi: 10.7717/peerj.10051 (PMC7566753; doi:10.7717/peerj.10051)
Supplement: Supplemental Information 4 [file peerj-08-10051-s004.docx]

### **Table S4** Long repeat sequences in seven *Calanthe* s.l. plastomes.

| **Species** | **Size of repeat I** | **Repeat start I** | **Repeat send I** |  | **Match direction** | **Region** | **Size of repeat II** | **Repeat start II** | **Repeat end II** | **Region** | **Distance of repeat** | **E-value** |
| --- | --- | --- | --- | --- | --- | --- | --- | --- | --- | --- | --- | --- |
| *Calanthe davidii* | 31 | 95567 | 95598 |  | F | IR1; CDS (ycf2) | 31 | 95585 | 95616 | IR1; CDS (ycf2) | 0 | 1.54E-09 |
| *Calanthe davidii* | 31 | 95567 | 95598 |  | P | IR1; CDS (ycf2) | 31 | 151255 | 151286 | IR2;CDS (ycf2) | 0 | 1.54E-09 |
| *Calanthe davidii* | 31 | 95585 | 95616 |  | P | IR1; CDS (ycf2) | 31 | 151273 | 151304 | IR2;CDS (ycf2) | 0 | 1.54E-09 |
| *Calanthe davidii* | 30 | 103366 | 103396 |  | C | IR1; IGS (rps12-trnV-GAC) | 30 | 143480 | 143510 | IR2; CDS (rps12 intron) | -3 | 6.76E-04 |
| *Calanthe davidii* | 31 | 151255 | 151286 |  | F | IR2; CDS (ycf2) | 31 | 151273 | 151304 | IR2;CDS (ycf2) | 0 | 1.54E-09 |
| *Calanthe davidii* | 30 | 40053 | 40083 |  | F | LSC; CDS (psaB) | 30 | 42277 | 42307 | LSC; CDS (psaA) | -3 | 6.76E-04 |
| *Calanthe davidii* | 39 | 44955 | 44994 |  | F | LSC; CDS (ycf3 intron) | 39 | 102965 | 103004 | IR1; IGS (rps12-trnV-GAC) | -2 | 1.57E-10 |
| *Calanthe davidii* | 30 | 44967 | 44997 |  | F | LSC; CDS (ycf3 intron) | 30 | 102977 | 103007 | IR1; IGS (rps12-trnV-GAC) |  | 6.76E-04 |
| *Calanthe davidii* | 39 | 44955 | 44994 |  | P | LSC; CDS (ycf3 intron) | 39 | 143867 | 143906 | IR2; CDS (rps12 intron) | -2 | 1.57E-10 |
| *Calanthe davidii* | 30 | 44967 | 44997 |  | P | LSC; CDS (ycf3 intron) | 30 | 143864 | 143894 | IR2; CDS (rps12 intron) | -3 | 6.76E-04 |
| *Calanthe davidii* | 30 | 74928 | 74958 |  | R | LSC; IGS (clpP-psbB) | 30 | 74929 | 74959 | LSC; IGS (clpP-psbB) | -1 | 5.55E-07 |
| *Calanthe davidii* | 58 | 65591 | 65649 |  | P | LSC; IGS (petA-psbJ) | 58 | 65591 | 65649 | LSC; IGS (rps16-trnQ-UUG) | -2 | 1.27E-21 |
| *Calanthe davidii* | 57 | 65287 | 65344 |  | P | LSC; IGS (petA-psbJ) | 57 | 65287 | 65344 | LSC; IGS (rps16-trnQ-UUG) | -3 | 2.71E-19 |
| *Calanthe davidii* | 48 | 30707 | 30755 |  | P | LSC; IGS (petN-psbM) | 48 | 30707 | 30755 | LSC; IGS (petN-psbM) | 0 | 8.98E-20 |
| *Calanthe davidii* | 33 | 30145 | 30178 |  | P | LSC; IGS (petN-psbM) | 33 | 30145 | 30178 | LSC; IGS (petN-psbM) | -1 | 9.54E-09 |
| *Calanthe davidii* | 46 | 43473 | 43519 |  | P | LSC; IGS (psaA-ycf3) | 46 | 43473 | 43519 | LSC; IGS (psaA-ycf3) | -2 | 1.34E-14 |
| *Calanthe davidii* | 38 | 77205 | 77243 |  | R | LSC; IGS (psbB-psbT) | 38 | 77205 | 77243 | LSC; IGS (psbB-psbT) | 0 | 9.41E-14 |
| *Calanthe davidii* | 30 | 77070 | 77100 |  | R | LSC; IGS (psbB-psbT) | 30 | 116687 | 116717 | SSC; IGS (ndhF-rpl32) | -3 | 6.76E-04 |
| *Calanthe davidii* | 32 | 37042 | 37074 |  | P | LSC; IGS (psbC-trnS-UGA);tRNA(trnS-UGA) | 32 | 46308 | 46340 | LSC; IGS (trnS-GGA-rps4); tRNA(trnS-GGA) | -3 | 5.16E-05 |
| *Calanthe davidii* | 30 | 8705 | 8735 |  | P | LSC; IGS (psbI-trnS-GCU);tRNA(trnS-GCU) | 30 | 46308 | 46338 | LSC; IGS (trnS-GGA) | -1 | 5.55E-07 |
| *Calanthe davidii* | 32 | 28706 | 28738 |  | P | LSC; IGS (rpoB-trnC-GCA) | 32 | 28706 | 28738 | LSC; IGS (rpoB-trnC-GCA) | 0 | 3.86E-10 |
| *Calanthe davidii* | 30 | 6682 | 6712 |  | F | LSC; IGS (rps16-trnQ-UUG) | 30 | 6710 | 6740 | LSC; IGS (rps16-trnQ-UUG) | -3 | 6.76E-04 |
| *Calanthe davidii* | 30 | 6613 | 6643 |  | P | LSC; IGS (rps16-trnQ-UUG) |  | 61264 | 61264 | LSC; IGS (accD-psaI) | -3 | 6.76E-04 |
| *Calanthe davidii* | 31 | 6706 | 6737 |  | R | LSC; IGS (rps16-trnQ-UUG) | 31 | 6706 | 6737 | LSC; IGS (rps16-trnQ-UUG) | -2 | 6.45E-06 |
| *Calanthe davidii* | 105 | 0 | 105 |  | P | LSC; IGS (rps19-psbA) | 105 | 87752 | 87857 | LSC;CDS (rpl22) | 0 | 4.32E-54 |
| *Calanthe davidii* | 59 | 122 | 181 |  | P | LSC; IGS (rps19-psbA) | 59 | 122 | 181 | LSC; IGS (rps19-psbA) | -1 | 3.79E-24 |
| *Calanthe davidii* | 36 | 29750 | 29786 |  | P | LSC; IGS (trnC-GCA-petN) | 36 | 29750 | 29786 | LSC; IGS (trnC-GCA-petN) | -2 | 8.54E-09 |
| *Calanthe davidii* | 31 | 32877 | 32908 |  | P | LSC; IGS (trnE-UUC-trnT-GGU) | 31 | 84384 | 84415 | LSC; IGS (rps8-rpl14) | -3 | 1.87E-04 |
| *Calanthe davidii* | 32 | 49680 | 49712 |  | P | LSC; IGS (trnF-GAA-ndhJ) | 32 | 49716 | 49748 | LSC; IGS (trnF-GAA-ndhJ);CDS(ndhJ) | -3 | 5.16E-05 |
| *Calanthe davidii* | 30 | 37877 | 37907 |  | P | LSC; IGS (trnG-UCC-trnfM-CAU) | 30 | 37877 | 37907 | LSC; IGS (trnG-UCC-trnfM-CAU) | -2 | 2.41E-05 |
| *Calanthe davidii* | 30 | 10652 | 10682 |  | F | LSC; tRNA (trnG-GCC) | 30 | 37802 | 37832 | LSC; tRNA (trnG-UCC) | -3 | 6.76E-04 |
| *Calanthe davidii* | 31 | 1749 | 1780 |  | P | LSC; tRNA (trnK-UUU) | 31 | 1749 | 1780 | LSC; tRNA (trnK-UUU) | -3 | 1.87E-04 |
| *Calanthe davidii* | 34 | 125686 | 125720 |  | P | SSC; CDS (ndhA intron) | 34 | 125686 | 125720 | SSC; CDS (ndhA intron) | 0 | 2.41E-11 |
| *Calanthe davidii* | 37 | 129414 | 129451 |  | P | SSC; CDS (ycf1) | 37 | 129414 | 129451 | SSC; CDS (ycf1) | -3 | 7.90E-08 |
| *Calanthe davidii* | 36 | 116933 | 116969 |  | F | SSC; IGS (ndhF-rpl32) | 36 | 116964 | 117000 | SSC; IGS (ndhF-rpl32) | 0 | 1.51E-12 |
| *Calanthe davidii* | 38 | 116902 | 116940 |  | F | SSC; IGS (ndhF-rpl32) | 38 | 116937 | 116975 | SSC; IGS (ndhF-rpl32) | -1 | 1.07E-11 |
| *Calanthe davidii* | 32 | 116902 | 116934 |  | F | SSC; IGS (ndhF-rpl32) | 32 | 116968 | 117000 | SSC; IGS (ndhF-rpl32) | -1 | 3.70E-08 |
| *Calanthe davidii* | 30 | 121894 | 121924 |  | F | SSC; IGS (psaC-ndhE) | 30 | 121908 | 121938 | SSC; IGS (psaC-ndhE) | -1 | 5.55E-07 |
| *Calanthe davidii* | 33 | 121855 | 121888 |  | P | SSC; IGS (psaC-ndhE) | 33 | 121892 | 121925 | SSC; IGS (psaC-ndhE) | 0 | 9.64E-11 |
| *Calanthe davidii* | 30 | 121856 | 121886 |  | P | SSC; IGS (psaC-ndhE) | 30 | 121908 | 121938 | SSC; IGS (psaC-ndhE) | -1 | 5.55E-07 |
| *Calanthe davidii* | 37 | 128051 | 128088 |  | P | SSC; IGS (rps15-ycf1) | 37 | 128051 | 128088 | SSC; IGS (rps15-ycf1) | -3 | 7.90E-08 |
| *Calanthe delavayi* | 31 | 91270 | 91301 |  | F | IR1; CDS (ycf2) | 31 | 91288 | 91319 | IR1; CDS (ycf2) | 0 | 1.38E-09 |
| *Calanthe delavayi* | 31 | 91270 | 91301 |  | P | IR1; CDS (ycf2) | 31 | 142273 | 142304 | IR2; CDS (ycf2) | 0 | 1.38E-09 |
| *Calanthe delavayi* | 31 | 91288 | 91319 |  | P | IR1; CDS (ycf2) | 31 | 142291 | 142322 | IR2; CDS (ycf2) | 0 | 1.38E-09 |
| *Calanthe delavayi* | 41 | 98852 | 98893 |  | C | IR1; IGS (rps12-trnV-GAC) | 41 | 134699 | 134740 | IR2; CDS (rps12 intron) | -2 | 9.68E-12 |
| *Calanthe delavayi* | 31 | 98871 | 98902 |  | C | IR1; IGS (rps12-trnV-GAC) | 31 | 134690 | 134721 | IR2; CDS (rps12 intron) | -2 | 5.76E-06 |
| *Calanthe delavayi* | 32 | 98817 | 98849 |  | F | IR1; IGS (rps12-trnV-GAC) | 32 | 98831 | 98863 | IR1; IGS (rps12-trnV-GAC) | -2 | 1.54E-06 |
| *Calanthe delavayi* | 32 | 98817 | 98849 |  | P | IR1; IGS (rps12-trnV-GAC) | 32 | 134729 | 134761 | IR2; CDS (rps12 intron) | -2 | 1.54E-06 |
| *Calanthe delavayi* | 32 | 98831 | 98863 |  | P | IR1; IGS (rps12-trnV-GAC) | 32 | 134743 | 134775 | IR2; CDS (rps12 intron) | -2 | 1.54E-06 |
| *Calanthe delavayi* | 41 | 98852 | 98893 |  | R | IR1; IGS (rps12-trnV-GAC) | 41 | 98852 | 98893 | IR1; IGS (rps12-trnV-GAC) | -2 | 9.68E-12 |
| *Calanthe delavayi* | 31 | 98871 | 98902 |  | R | IR1; IGS (rps12-trnV-GAC) | 31 | 98871 | 98902 | IR1; IGS (rps12-trnV-GAC) | -2 | 5.76E-06 |
| *Calanthe delavayi* | 32 | 134729 | 134761 |  | F | IR2; CDS (rps12 intron) | 32 | 134743 | 134775 | IR2; CDS (rps12 intron) | -2 | 1.54E-06 |
| *Calanthe delavayi* | 41 | 134699 | 134740 |  | R | IR2; CDS (rps12 intron) | 41 | 134699 | 134740 | IR2; CDS (rps12 intron) | -2 | 9.68E-12 |
| *Calanthe delavayi* | 31 | 134690 | 134721 |  | R | IR2; CDS (rps12 intron) | 31 | 134690 | 134721 | IR2; CDS (rps12 intron) | -2 | 5.76E-06 |
| *Calanthe delavayi* | 31 | 142273 | 142304 |  | F | IR2; CDS (ycf2) | 31 | 142291 | 142322 | IR2; CDS (ycf2) | 0 | 1.38E-09 |
| *Calanthe delavayi* | 30 | 39073 | 39103 |  | F | LSC; CDS (psaB) | 30 | 41297 | 41327 | LSC; CDS (psaA) | -3 | 6.03E-04 |
| *Calanthe delavayi* | 39 | 43946 | 43985 |  | F | LSC; CDS (ycf3 inrton) | 39 | 98437 | 98476 | IR1; IGS (rps12-trnV-GAC) | -2 | 1.40E-10 |
| *Calanthe delavayi* | 39 | 43946 | 43985 |  | P | LSC; CDS (ycf3 inrton) | 39 | 135116 | 135155 | IR2; CDS (rps12 intron) | -2 | 1.40E-10 |
| *Calanthe delavayi* | 30 | 43958 | 43988 |  | F | LSC; CDS(ycf3 intron) | 30 | 98449 | 98479 | IR1; IGS (rps12-trnV-GAC) | -3 | 6.03E-04 |
| *Calanthe delavayi* | 30 | 43958 | 43988 |  | P | LSC; CDS(ycf3 intron) | 30 | 135113 | 135143 | IR2; CDS (rps12 intron) | -3 | 6.03E-04 |
| *Calanthe delavayi* | 32 | 49384 | 49416 |  | P | LSC; IGS (ndhJ-trnV-UAC) | 32 | 53207 | 53239 | LSC; IGS (atpB-rbcL) | -3 | 4.61E-05 |
| *Calanthe delavayi* | 37 | 61779 | 61816 |  | C | LSC; IGS (petA-psbJ ) | 37 | 61780 | 61817 | LSC; IGS (petA-psbJ ) | -3 | 7.05E-08 |
| *Calanthe delavayi* | 31 | 61724 | 61755 |  | F | LSC; IGS (petA-psbJ ) | 31 | 61757 | 61788 | LSC; IGS (petA-psbJ ) | -2 | 5.76E-06 |
| *Calanthe delavayi* | 30 | 61791 | 61821 |  | F | LSC; IGS (petA-psbJ ) | 30 | 61793 | 61823 | LSC; IGS (petA-psbJ ) | -3 | 6.03E-04 |
| *Calanthe delavayi* | 33 | 61787 | 61820 |  | R | LSC; IGS (petA-psbJ ) | 33 | 61787 | 61820 | LSC; IGS (petA-psbJ ) | -2 | 4.09E-07 |
| *Calanthe delavayi* | 33 | 61788 | 61821 |  | R | LSC; IGS (petA-psbJ ) | 33 | 61788 | 61821 | LSC; IGS (petA-psbJ ) | -2 | 4.09E-07 |
| *Calanthe delavayi* | 65 | 61457 | 61522 |  | P | LSC; IGS (petA-psbJ) | 65 | 61457 | 61522 | LSC; IGS (petA-psbJ) | -3 | 5.50E-24 |
| *Calanthe delavayi* | 36 | 61786 | 61822 |  | P | LSC; IGS (petA-psbJ) | 36 | 61786 | 61822 | LSC; IGS (petA-psbJ) | 0 | 1.34E-12 |
| *Calanthe delavayi* | 48 | 30021 | 30069 |  | P | LSC; IGS (petN-psbM) | 48 | 30021 | 30069 | LSC; IGS (petN-psbM) | 0 | 8.01E-20 |
| *Calanthe delavayi* | 30 | 29578 | 29608 |  | P | LSC; IGS (petN-psbM) | 30 | 113961 | 113991 | SSC; IGS (psaC-ndhE) | -3 | 6.03E-04 |
| *Calanthe delavayi* | 35 | 73137 | 73172 |  | P | LSC; IGS (psbB-psbT) | 35 | 73137 | 73172 | LSC; IGS (psbB-psbT) | -3 | 9.50E-07 |
| *Calanthe delavayi* | 32 | 36091 | 36123 |  | P | LSC; IGS (psbC-trnS-UGA);tRNA(trnS-UGA) | 32 | 45388 | 45420 | LSC; IGS (trnS-GGA-rps4);tRNA(trnS-GGA) | -3 | 4.61E-05 |
| *Calanthe delavayi* | 32 | 28232 | 28264 |  | P | LSC; IGS (rpoB-trnC-GCA) | 32 | 28232 | 28264 | LSC; IGS (rpoB-trnC-GCA) | 0 | 3.44E-10 |
| *Calanthe delavayi* | 36 | 29164 | 29200 |  | P | LSC; IGS (trnC-GCA-petN) | 36 | 29164 | 29200 | LSC; IGS (trnC-GCA-petN) | -2 | 7.62E-09 |
| *Calanthe delavayi* | 32 | 48693 | 48725 |  | P | LSC; IGS (trnF-GAA-ndhJ) | 32 | 48729 | 48761 | LSC; IGS (trnF-GAA-ndhJ);CDS(ndhJ) | -3 | 4.61E-05 |
| *Calanthe delavayi* | 30 | 10266 | 10296 |  | F | LSC; tRNA (trnG-GCC) | 30 | 36852 | 36882 | LSC; tRNA (trnG-UCC) | -3 | 6.03E-04 |
| *Calanthe delavayi* | 31 | 1611 | 1642 |  | P | LSC; tRNA (trnK-UUU) | 31 | 1611 | 1642 | LSC; tRNA (trnK-UUU) | -3 | 1.67E-04 |
| *Calanthe delavayi* | 39 | 3673 | 3712 |  | R | LSC; tRNA (trnK-UUU) | 39 | 3673 | 3712 | LSC; tRNA (trnK-UUU) | -2 | 1.40E-10 |
| *Calanthe delavayi* | 30 | 8421 | 8451 |  | P | LSC; tRNA (trnS-GCU) | 30 | 45388 | 45418 | LSC; tRNA (trnS-GGA) | -1 | 4.95E-07 |
| *Calanthe delavayi* | 34 | 117031 | 117065 |  | P | SSC; CDS (ndhA inrton) | 34 | 117031 | 117065 | SSC; CDS (ndhA inrton) | 0 | 2.15E-11 |
| *Calanthe delavayi* | 37 | 120667 | 120704 |  | P | SSC; CDS (ycf1) | 37 | 120667 | 120704 | SSC; CDS (ycf1) | -3 | 7.05E-08 |
| *Calanthe delavayi* | 37 | 119294 | 119331 |  | P | SSC; IGS (rps15-ycf1) | 37 | 119294 | 119331 | SSC; IGS (rps15-ycf1) | -3 | 7.05E-08 |
| *Calanthe delavayi* | 74 | 108867 | 108941 |  | F | SSC; IGS (trnN-GUU-rpl32) | 74 | 108904 | 108978 | SSC; IGS (trnN-GUU-rpl32) | 0 | 1.78E-35 |
| *Calanthe delavayi* | 37 | 108867 | 108904 |  | F | SSC; IGS (trnN-GUU-rpl32) | 37 | 108941 | 108978 | SSC; IGS (trnN-GUU-rpl32) | 0 | 3.36E-13 |
| *Calanthe delavayi* | 30 | 108846 | 108876 |  | R | SSC; IGS (trnN-GUU-rpl32) | 30 | 108846 | 108876 | SSC; IGS (trnN-GUU-rpl32) | -2 | 2.15E-05 |
| *Styloglossum lyroglossa* | 31 | 93243 | 93274 |  | F | IR1; CDS (ycf2) | 31 | 93261 | 93292 | IR1; CDS (ycf2) | 0 | 1.48E-09 |
| *Styloglossum lyroglossa* | 31 | 93243 | 93274 |  | P | IR1; CDS (ycf2) | 31 | 148270 | 148301 | IR2; CDS (ycf2) | 0 | 1.48E-09 |
| *Styloglossum lyroglossa* | 31 | 93261 | 93292 |  | P | IR1; CDS (ycf2) | 31 | 148288 | 148319 | IR2; CDS (ycf2) | 0 | 1.48E-09 |
| *Styloglossum lyroglossa* | 31 | 148270 | 148301 |  | F | IR2; CDS (ycf2) | 31 | 148288 | 148319 | IR2; CDS (ycf2) | 0 | 1.48E-09 |
| *Styloglossum lyroglossa* | 30 | 39018 | 39048 |  | F | LSC; CDS (psaB) | 30 | 41242 | 41272 | LSC; CDS (psaA) | -3 | 6.51E-04 |
| *Styloglossum lyroglossa* | 30 | 43887 | 43917 |  | F | LSC; CDS (ycf3 inrton) | 30 | 100656 | 100686 | IR1; IGS (rps12-trnV-GAC) | -3 | 6.51E-04 |
| *Styloglossum lyroglossa* | 30 | 43887 | 43917 |  | P | LSC; CDS (ycf3 inrton) | 30 | 140876 | 140906 | IR2; CDS(rps12 intron) | -3 | 6.51E-04 |
| *Styloglossum lyroglossa* | 39 | 43875 | 43914 |  | F | LSC; CDS (ycf3 intron) | 39 | 100644 | 100683 | IR1; IGS (rps12-trnV-GAC) | -2 | 1.51E-10 |
| *Styloglossum lyroglossa* | 39 | 43875 | 43914 |  | P | LSC; CDS (ycf3 intron) | 39 | 140879 | 140918 | IR2; CDS (rps12 intron) | -2 | 1.51E-10 |
| *Styloglossum lyroglossa* | 30 | 63537 | 63567 |  | P | LSC; IGS (petA-psbJ) | 30 | 63537 | 63567 | LSC; IGS (petA-psbJ) | 0 | 5.94E-09 |
| *Styloglossum lyroglossa* | 48 | 30259 | 30307 |  | P | LSC; IGS (petN-pabM) | 48 | 30259 | 30307 | LSC; IGS (petN-pabM) | -2 | 8.77E-16 |
| *Styloglossum lyroglossa* | 32 | 36013 | 36045 |  | P | LSC; IGS (psbC-trnS-UGA);tRNA(trnS-UGA) | 32 | 45250 | 45282 | LSC; tRNA (trnS-GGA);IGS(trnS-GGA-rps4) | -3 | 4.97E-05 |
| *Styloglossum lyroglossa* | 31 | 8434 | 8465 |  | P | LSC; IGS (psbI-trnS-GCU);tRNA(trnS-GCU) | 31 | 45248 | 45279 | LSC; tRNA (trnS-GGA) | -1 | 1.38E-07 |
| *Styloglossum lyroglossa* | 31 | 6486 | 6517 |  | P | LSC; IGS (rps16-trnQ-UUG) | 31 | 74961 | 74992 | LSC; IGS (psbB-psbT) | -3 | 1.80E-04 |
| *Styloglossum lyroglossa* | 105 | 0 | 105 |  | P | LSC; IGS (rps19-psbA) | 105 | 85421 | 85526 | LSC; CDS (rpl22);IGS(rpl22-rps19) | 0 | 4.16E-54 |
| *Styloglossum lyroglossa* | 40 | 109 | 149 |  | P | LSC; IGS (rps19-psbA) | 40 | 109 | 149 | LSC; IGS (rps19-psbA) | -2 | 3.98E-11 |
| *Styloglossum lyroglossa* | 52 | 29426 | 29478 |  | P | LSC; IGS (trnC-GCA-petN) | 52 | 29426 | 29478 | LSC; IGS (trnC-GCA-petN) | 0 | 3.38E-22 |
| *Styloglossum lyroglossa* | 32 | 48792 | 48824 |  | P | LSC; IGS (trnF-GAA-ndhJ) | 32 | 48828 | 48860 | LSC; IGS (trnF-GAA-ndhJ) | -3 | 4.97E-05 |
| *Styloglossum lyroglossa* | 36 | 36838 | 36874 |  | P | LSC; IGS (trnG-UCC-trnfM-CAU) | 36 | 36838 | 36874 | LSC; IGS (trnG-UCC-trnfM-CAU) | 0 | 1.45E-12 |
| *Styloglossum lyroglossa* | 30 | 10388 | 10418 |  | F | LSC; tRNA (trnG-UCC) | 30 | 36766 | 36796 | LSC; tRNA (trnG-UCC) | -3 | 6.51E-04 |
| *Styloglossum lyroglossa* | 31 | 3784 | 3815 |  | R | LSC; tRNA (trnK-UUU) | 31 | 3792 | 3823 | LSC;tRNA (trnK-UUU) | -3 | 1.80E-04 |
| *Styloglossum lyroglossa* | 34 | 122855 | 122889 |  | P | SSC; CDS (ndhA intron) | 34 | 122855 | 122889 | SSC; CDS (ndhA intron) | 0 | 2.32E-11 |
| *Styloglossum lyroglossa* | 37 | 126592 | 126629 |  | P | SSC; CDS (ycf1) | 37 | 126592 | 126629 | SSC; CDS (ycf1) | -3 | 7.61E-08 |
| *Styloglossum lyroglossa* | 37 | 125222 | 125259 |  | P | SSC; IGS (rps15-ycf1) | 37 | 125222 | 125259 | SSC; IGS (rps15-ycf1) | -3 | 7.61E-08 |
| *Preptanthe rubens* | 30 | 95156 | 95186 |  | F | IR1; CDS (ycf2) | 30 | 95174 | 95204 | IR1; CDS (ycf2) | 0 | 6.11E-09 |
| *Preptanthe rubens* | 30 | 95156 | 95186 |  | P | IR1; CDS (ycf2) | 30 | 150613 | 150643 | IR2; CDS (ycf2) | 0 | 6.11E-09 |
| *Preptanthe rubens* | 30 | 95174 | 95204 |  | P | IR1; CDS (ycf2) | 30 | 150631 | 150661 | IR2; CDS (ycf2) | 0 | 6.11E-09 |
| *Preptanthe rubens* | 30 | 150613 | 150643 |  | F | IR2; CDS (rps12 intron) | 30 | 150631 | 150661 | IR2; CDS (rps12 intron) | 0 | 6.11E-09 |
| *Preptanthe rubens* | 49 | 147320 | 147369 |  | P | IR2; IGS (ndhB-trnL-CAA) | 49 | 147320 | 147369 | IR2; IGS (ndhB-trnL-CAA) | -1 | 3.27E-18 |
| *Preptanthe rubens* | 31 | 5347 | 5378 |  | P | LSC; CDS (rps16 intron) | 31 | 55991 | 56022 | LSC; IGS (atpB-rbcL) | -3 | 1.85E-04 |
| *Preptanthe rubens* | 39 | 44258 | 44297 |  | F | LSC; CDS (yf3 intron) | 39 | 102506 | 102545 | IR1; IGS (rps12-trnV-GAC) | -2 | 1.55E-10 |
| *Preptanthe rubens* | 39 | 44258 | 44297 |  | P | LSC; CDS (yf3 intron) | 39 | 143272 | 143311 | IR2; CDS (rps12 intron) | -2 | 1.55E-10 |
| *Preptanthe rubens* | 31 | 55946 | 55977 |  | F | LSC; IGS (atpB-rbcL) | 31 | 74490 | 74521 | LSC; IGS (clpP-psbB) | -1 | 1.42E-07 |
| *Preptanthe rubens* | 32 | 74296 | 74328 |  | C | LSC; IGS (clpP-psbB) | 32 | 74335 | 74367 | LSC; IGS (clpP-psbB) | -3 | 5.11E-05 |
| *Preptanthe rubens* | 31 | 74302 | 74333 |  | C | LSC; IGS (clpP-psbB) | 31 | 74303 | 74334 | LSC; IGS (clpP-psbB) | -3 | 1.85E-04 |
| *Preptanthe rubens* | 34 | 74264 | 74298 |  | F | LSC; IGS (clpP-psbB) | 34 | 74284 | 74318 | LSC; IGS (clpP-psbB) | -1 | 2.43E-09 |
| *Preptanthe rubens* | 32 | 74286 | 74318 |  | F | LSC; IGS (clpP-psbB) | 32 | 74326 | 74358 | LSC; IGS (clpP-psbB) | -3 | 5.11E-05 |
| *Preptanthe rubens* | 30 | 74304 | 74334 |  | F | LSC; IGS (clpP-psbB) | 30 | 74342 | 74372 | LSC; IGS (clpP-psbB) | -3 | 6.69E-04 |
| *Preptanthe rubens* | 41 | 74325 | 74366 |  | P | LSC; IGS (clpP-psbB) | 41 | 74342 | 74383 | LSC; IGS (clpP-psbB) | -3 | 4.19E-10 |
| *Preptanthe rubens* | 37 | 74290 | 74327 |  | R | LSC; IGS (clpP-psbB) | 37 | 74342 | 74379 | LSC; IGS (clpP-psbB) | -3 | 7.82E-08 |
| *Preptanthe rubens* | 30 | 74304 | 74334 |  | R | LSC; IGS (clpP-psbB) | 30 | 74304 | 74334 | LSC; IGS (clpP-psbB) | -2 | 2.39E-05 |
| *Preptanthe rubens* | 30 | 74343 | 74373 |  | R | LSC; IGS (clpP-psbB) | 30 | 74343 | 74373 | LSC; IGS (clpP-psbB) | -2 | 2.39E-05 |
| *Preptanthe rubens* | 30 | 74286 | 74316 |  | R | LSC; IGS (clpP-psbB) | 30 | 74302 | 74332 | LSC; IGS (clpP-psbB) | -3 | 6.69E-04 |
| *Preptanthe rubens* | 30 | 74304 | 74334 |  | R | LSC; IGS (clpP-psbB) | 30 | 74353 | 74383 | LSC; IGS (clpP-psbB) | -3 | 6.69E-04 |
| *Preptanthe rubens* | 53 | 65046 | 65099 |  | P | LSC; IGS (petA-psbJ) | 53 | 65046 | 65099 | LSC; IGS (petA-psbJ) | -3 | 5.49E-17 |
| *Preptanthe rubens* | 31 | 65384 | 65415 |  | P | LSC; IGS (petA-psbJ) | 31 | 74219 | 74250 | LSC; IGS (clpP-psbB) | -3 | 1.85E-04 |
| *Preptanthe rubens* | 48 | 29943 | 29991 |  | P | LSC; IGS (petN-psbM) | 48 | 29943 | 29991 | LSC; IGS (petN-psbM) | -2 | 9.02E-16 |
| *Preptanthe rubens* | 33 | 36340 | 36373 |  | P | LSC; IGS (psbC-trnS-UGA);tRNA(trnS-UGA) | 33 | 45693 | 45726 | LSC; IGS (trnS-GAA-rps4);tRNA(trnS-GAA) | -3 | 1.41E-05 |
| *Preptanthe rubens* | 30 | 8654 | 8684 |  | P | LSC; IGS (psbI-trnS-GCU);tRNA(trnS-GCU) | 30 | 45694 | 45724 | LSC; IGS (trnS-GAA-rps4);tRNA(trnS-GAA) | -1 | 5.50E-07 |
| *Preptanthe rubens* | 34 | 28417 | 28451 |  | F | LSC; IGS (rpoB-trnG-GCA) | 34 | 28430 | 28464 | LSC; IGS (rpoB-trnG-GCA) | 0 | 2.39E-11 |
| *Preptanthe rubens* | 32 | 28555 | 28587 |  | P | LSC; IGS (rpoB-trnG-GCA) | 32 | 28555 | 28587 | LSC; IGS (rpoB-trnG-GCA) | 0 | 3.82E-10 |
| *Preptanthe rubens* | 105 | 0 | 105 |  | P | LSC; IGS (rps19-psbA) | 105 | 87497 | 87602 | LSC; CDS (rps19) | 0 | 4.28E-54 |
| *Preptanthe rubens* | 59 | 123 | 182 |  | P | LSC; IGS (rps19-psbA) | 59 | 123 | 182 | LSC; IGS (rps19-psbA) | -1 | 3.75E-24 |
| *Preptanthe rubens* | 46 | 32188 | 32234 |  | P | LSC; IGS (trnE-UUC-trnT-GGU) | 46 | 32188 | 32234 | LSC; IGS (trnE-UUC-trnT-GGU) | -2 | 1.32E-14 |
| *Preptanthe rubens* | 47 | 32210 | 32257 |  | P | LSC; IGS (trnE-UUC-trnT-GGU) | 47 | 32210 | 32257 | LSC; IGS (trnE-UUC-trnT-GGU) | -3 | 1.56E-13 |
| *Preptanthe rubens* | 32 | 49411 | 49443 |  | P | LSC; IGS (trnF-GAA-ndhJ) | 32 | 49447 | 49479 | LSC; IGS (trnF-GAA-ndhJ);CDS(ndhJ) | -3 | 5.11E-05 |
| *Preptanthe rubens* | 46 | 37177 | 37223 |  | P | LSC; IGS (trnG-UCC-trnfM-CAU) | 46 | 37177 | 37223 | LSC; IGS (trnG-UCC-trnfM-CAU) | 0 | 1.42E-18 |
| *Preptanthe rubens* | 49 | 98448 | 98497 |  | F | LSC; IGS (trnL-CAA-ndhB) | 49 | 147320 | 147369 | IR2; IGS (ndhB-trnL-CAA) | -1 | 3.27E-18 |
| *Preptanthe rubens* | 49 | 98448 | 98497 |  | P | LSC; IGS (trnL-CAA-ndhB) | 49 | 98448 | 98497 | LSC; IGS (trnL-CAA-ndhB) | -1 | 3.27E-18 |
| *Preptanthe rubens* | 30 | 10414 | 10444 |  | F | LSC; tRNA (trnG-GCC) | 30 | 37110 | 37140 | LSC; IGS (psbZ-trnC-UCC) | -3 | 6.69E-04 |
| *Preptanthe rubens* | 31 | 1747 | 1778 |  | P | LSC; tRNA (trnK-UUU intron) | 31 | 1747 | 1778 | LSC; tRNA (trnK-UUU intron) | -3 | 1.85E-04 |
| *Preptanthe rubens* | 30 | 39364 | 39394 |  | F | LSC;CDS (psaB) | 30 | 41588 | 41618 | LSC; CDS (psaA) | -3 | 6.69E-04 |
| *Preptanthe rubens* | 30 | 44270 | 44300 |  | F | LSC;CDS (ycf3 intron) | 30 | 102518 | 102548 | IR1; IGS (rps12-trnV-GAC) | -3 | 6.69E-04 |
| *Preptanthe rubens* | 30 | 44270 | 44300 |  | P | LSC;CDS (ycf3 intron) | 30 | 143269 | 143299 | IR2; CDS (rps12 intron) | -3 | 6.69E-04 |
| *Preptanthe rubens* | 34 | 125095 | 125129 |  | P | SSC; CDS (ndhA intron) | 34 | 125095 | 125129 | SSC; CDS (ndhA intron) | 0 | 2.39E-11 |
| *Preptanthe rubens* | 37 | 128830 | 128867 |  | P | SSC; CDS (ycf1) | 37 | 128830 | 128867 | SSC; CDS (ycf1) | -3 | 7.82E-08 |
| *Preptanthe rubens* | 30 | 117319 | 117349 |  | P | SSC; IGS (rpl32-trnL-UAG) | 30 | 117357 | 117387 | SSC; IGS (rpl32-trnL-UAG) | 0 | 6.11E-09 |
| *Preptanthe rubens* | 30 | 117281 | 117311 |  | R | SSC; IGS (rpl32-trnL-UAG) | 30 | 117281 | 117311 | SSC; IGS (rpl32-trnL-UAG) | -2 | 2.39E-05 |
| *Preptanthe rubens* | 37 | 127453 | 127490 |  | P | SSC; IGS (rps15-ycf1) | 37 | 127453 | 127490 | SSC; IGS (rps15-ycf1) | -3 | 7.82E-08 |
| *Calanthe triplicata* | 30 | 95144 | 95174 |  | F | IR1; CDS (ycf2) | 30 | 95162 | 95192 | IR1; CDS (ycf2) | 0 | 6.15E-09 |
| *Calanthe triplicata* | 30 | 95144 | 95174 |  | P | IR1; CDS (ycf2) | 30 | 150872 | 150902 | IR2; CDS (ycf2) | 0 | 6.15E-09 |
| *Calanthe triplicata* | 30 | 95162 | 95192 |  | P | IR1; CDS (ycf2) | 30 | 150890 | 150920 | IR2; CDS (ycf2) | 0 | 6.15E-09 |
| *Calanthe triplicata* | 43 | 102965 | 103008 |  | C | IR1; IGS (rps12-trnV-GAC) | 43 | 143063 | 143106 | IR2; CDS (rps12 intron) | -3 | 3.05E-11 |
| *Calanthe triplicata* | 32 | 102971 | 103003 |  | C | IR1; IGS (rps12-trnV-GAC) | 32 | 143061 | 143093 | IR2; CDS (rps12 intron) | -2 | 1.72E-06 |
| *Calanthe triplicata* | 30 | 102946 | 102976 |  | C | IR1; IGS (rps12-trnV-GAC) | 30 | 143093 | 143123 | IR2; CDS (rps12 intron) | -3 | 6.74E-04 |
| *Calanthe triplicata* | 30 | 102965 | 102995 |  | F | IR1; IGS (rps12-trnV-GAC) | 30 | 102973 | 103003 | IR1; IGS (rps12-trnV-GAC) | -3 | 6.74E-04 |
| *Calanthe triplicata* | 30 | 102965 | 102995 |  | P | IR1; IGS (rps12-trnV-GAC) | 30 | 143061 | 143091 | IR2; CDS (rps12 intron) | -3 | 6.74E-04 |
| *Calanthe triplicata* | 30 | 102973 | 103003 |  | P | IR1; IGS (rps12-trnV-GAC) | 30 | 143069 | 143099 | IR2; CDS (rps12 intron) | -3 | 6.74E-04 |
| *Calanthe triplicata* | 32 | 102971 | 103003 |  | R | IR1; IGS (rps12-trnV-GAC) | 32 | 102971 | 103003 | IR1; IGS (rps12-trnV-GAC) | -2 | 1.72E-06 |
| *Calanthe triplicata* | 30 | 143061 | 143091 |  | F | IR2; CDS (rps12 intron) | 30 | 143069 | 143099 | IR2; CDS (rps12 intron) | -3 | 6.74E-04 |
| *Calanthe triplicata* | 32 | 143061 | 143093 |  | R | IR2; CDS (rps12 intron) | 32 | 143061 | 143093 | IR2; CDS (rps12 intron) | -2 | 1.72E-06 |
| *Calanthe triplicata* | 30 | 150872 | 150902 |  | F | IR2; CDS (ycf2) | 30 | 150890 | 150920 | IR2; CDS (ycf2) | 0 | 6.15E-09 |
| *Calanthe triplicata* | 30 | 40006 | 40036 |  | F | LSC; CDS (psaB) | 30 | 42230 | 42260 | LSC; CDS (psaA) | -3 | 6.74E-04 |
| *Calanthe triplicata* | 30 | 44907 | 44937 |  | F | LSC; CDS (ycf3 inrton) | 30 | 102557 | 102587 | IR1; IGS (rps12-trnV-GAC) | -3 | 6.74E-04 |
| *Calanthe triplicata* | 30 | 44907 | 44937 |  | P | LSC; CDS (ycf3 inrton) | 30 | 143477 | 143507 | IR2; CDS (rps12 intron) | -3 | 6.74E-04 |
| *Calanthe triplicata* | 39 | 44895 | 44934 |  | F | LSC; CDS (ycf3 intron) | 39 | 102545 | 102584 | IR1; IGS (rps12-trnV-GAC) | -2 | 1.56E-10 |
| *Calanthe triplicata* | 39 | 44895 | 44934 |  | P | LSC; CDS (ycf3 intron) | 39 | 143480 | 143519 | IR2; CDS (rps12 intron) | -2 | 1.56E-10 |
| *Calanthe triplicata* | 30 | 74338 | 74368 |  | F | LSC; IGS (clpP-psbB) | 30 | 116509 | 116539 | SSC; IGS (ndhF-rpl32) | -3 | 6.74E-04 |
| *Calanthe triplicata* | 65 | 65291 | 65356 |  | P | LSC; IGS (petA-psbJ ) | 65 | 65291 | 65356 | LSC; IGS (petA-psbJ) | -3 | 6.14E-24 |
| *Calanthe triplicata* | 48 | 30621 | 30669 |  | P | LSC; IGS (petN-pabM) | 48 | 30621 | 30669 | LSC; IGS (petN-pabM) | 0 | 8.95E-20 |
| *Calanthe triplicata* | 34 | 76753 | 76787 |  | R | LSC; IGS (psbB-psbT) | 34 | 76753 | 76787 | LSC; IGS (psbB-psbT) | 0 | 2.40E-11 |
| *Calanthe triplicata* | 32 | 36995 | 37027 |  | P | LSC; IGS (psbC-trnS-UGA);tRNA(trnS-UGA) | 32 | 46338 | 46370 | LSC; IGS (trnS-GGA-rps4);tRNA(trnS-GGA) | -3 | 5.15E-05 |
| *Calanthe triplicata* | 30 | 8604 | 8634 |  | P | LSC; IGS (psbI-trnS-GCU);tRNA(trnS-GCU) | 30 | 46336 | 46366 | LSC; tRNA (trnS-GGA) | -2 | 2.41E-05 |
| *Calanthe triplicata* | 32 | 28599 | 28631 |  | P | LSC; IGS (rpoB-trnC-GCA) | 32 | 28599 | 28631 | LSC; IGS (rpoB-trnC-GCA) | 0 | 3.84E-10 |
| *Calanthe triplicata* | 30 | 6661 | 6691 |  | F | LSC; IGS (rps16-trnQ-UUG) | 30 | 76818 | 76848 | LSC; IGS (psbB-psbT) | -3 | 6.74E-04 |
| *Calanthe triplicata* | 59 | 25 | 84 |  | P | LSC; IGS (rps19-psbA) | 59 | 25 | 84 | LSC; IGS (rps19-psbA) | -1 | 3.78E-24 |
| *Calanthe triplicata* | 36 | 29659 | 29695 |  | P | LSC; IGS (trnC-GCA-petN) | 36 | 29659 | 29695 | LSC; IGS (trnC-GCA-petN) | -2 | 8.51E-09 |
| *Calanthe triplicata* | 30 | 32801 | 32831 |  | F | LSC; IGS (trnE-UUC-trnT-GGU) | 30 | 32814 | 32844 | LSC; IGS (trnE-UUC-trnT-GGU) | -2 | 2.41E-05 |
| *Calanthe triplicata* | 30 | 37830 | 37860 |  | P | LSC; IGS (trnG-UCC-trnfM-CAU) | 30 | 37830 | 37860 | LSC; IGS (trnG-UCC-trnfM-CAU) | -2 | 2.41E-05 |
| *Calanthe triplicata* | 30 | 9346 | 9376 |  | P | LSC; IGS (trnS-GCU-trnG-GCC) | 30 | 9346 | 9376 | LSC; IGS (trnS-GCU-trnG-GCC) | 0 | 6.15E-09 |
| *Calanthe triplicata* | 36 | 47748 | 47784 |  | R | LSC; IGS (trnT-UGU-trnLUAA) | 36 | 47748 | 47784 | LSC; IGS (trnT-UGU-trnLUAA) | -2 | 8.51E-09 |
| *Calanthe triplicata* | 30 | 10584 | 10614 |  | F | LSC; tRNA(trnK-UUU) | 30 | 37755 | 37785 | LSC; tRNA(trnG-UCC) | -3 | 6.74E-04 |
| *Calanthe triplicata* | 39 | 3795 | 3834 |  | R | LSC; tRNA(trnK-UUU) | 39 | 3795 | 3834 | LSC; tRNA(trnK-UUU) | -2 | 1.56E-10 |
| *Calanthe triplicata* | 37 | 128954 | 128991 |  | P | SSC; CDS (ycf1) | 37 | 128954 | 128991 | SSC; CDS (ycf1) | -3 | 7.87E-08 |
| *Calanthe triplicata* | 34 | 125189 | 125223 |  | P | SSC; IGS (psaC-ndhE) | 34 | 125189 | 125223 | SSC; IGS (psaC-ndhE) | 0 | 2.40E-11 |
| *Calanthe triplicata* | 37 | 127556 | 127593 |  | P | SSC; IGS (rps15-ycf1) | 37 | 127556 | 127593 | SSC; IGS (rps15-ycf1) | -3 | 7.87E-08 |
| *Cephalantheropsis obcordata* | 31 | 94523 | 94554 |  | F | IR1; CDS (ycf2) | 31 | 94541 | 94572 | IR1; CDS (ycf2) | 0 | 1.52E-09 |
| *Cephalantheropsis obcordata* | 31 | 94523 | 94554 |  | P | IR1; CDS (ycf2) | 31 | 149998 | 150029 | IR2; CDS (ycf2) | 0 | 1.52E-09 |
| *Cephalantheropsis obcordata* | 31 | 94541 | 94572 |  | P | IR1; CDS (ycf2) | 31 | 150016 | 150047 | IR2; CDS (ycf2) | 0 | 1.52E-09 |
| *Cephalantheropsis obcordata* | 30 | 102325 | 102355 |  | C | IR1; IGS (rps12-trnV-GAC) | 30 | 142218 | 142248 | IR2; CDS(rps12) | -3 | 6.67E-04 |
| *Cephalantheropsis obcordata* | 31 | 149998 | 150029 |  | F | IR2; CDS (ycf2) | 31 | 150016 | 150047 | IR2; CDS (ycf2) | 0 | 1.52E-09 |
| *Cephalantheropsis obcordata* | 30 | 73311 | 73341 |  | F | LSC; CDS (clpP intron) | 30 | 115680 | 115710 | SSC; IGS (ndhF-rpl32) | -3 | 6.67E-04 |
| *Cephalantheropsis obcordata* | 33 | 73313 | 73346 |  | R | LSC; CDS (clpP intron) | 33 | 115679 | 115712 | SSC; IGS (ndhF-rpl32) | -3 | 1.40E-05 |
| *Cephalantheropsis obcordata* | 30 | 39463 | 39493 |  | F | LSC; CDS (psaB) | 30 | 41687 | 41717 | LSC; CDS (psaA) | -3 | 6.67E-04 |
| *Cephalantheropsis obcordata* | 39 | 44354 | 44393 |  | F | LSC; CDS (ycf3 inrton) | 39 | 101919 | 101958 | IR1; IGS (rps12-trnV-GAC) | -2 | 1.55E-10 |
| *Cephalantheropsis obcordata* | 30 | 44366 | 44396 |  | F | LSC; CDS (ycf3 inrton) | 30 | 101931 | 101961 | IR1; IGS (rps12-trnV-GAC) | -3 | 6.67E-04 |
| *Cephalantheropsis obcordata* | 39 | 44354 | 44393 |  | P | LSC; CDS (ycf3 inrton) | 39 | 142612 | 142651 | IR2; CDS (rps12 intron) | -2 | 1.55E-10 |
| *Cephalantheropsis obcordata* | 30 | 44366 | 44396 |  | P | LSC; CDS (ycf3 inrton) | 30 | 142609 | 142639 | IR2; CDS(rps12) | -3 | 6.67E-04 |
| *Cephalantheropsis obcordata* | 30 | 55518 | 55548 |  | C | LSC; IGS (atpB-rbcL) | 30 | 55519 | 55549 | LSC; IGS (atpB-rbcL) | -3 | 6.67E-04 |
| *Cephalantheropsis obcordata* | 34 | 55512 | 55546 |  | P | LSC; IGS (atpB-rbcL) | 34 | 64820 | 64854 | LSC; IGS (petA-psbJ ) | -2 | 1.20E-07 |
| *Cephalantheropsis obcordata* | 32 | 55504 | 55536 |  | P | LSC; IGS (atpB-rbcL) | 32 | 64828 | 64860 | LSC; IGS (petA-psbJ ) | -3 | 5.09E-05 |
| *Cephalantheropsis obcordata* | 31 | 73852 | 73883 |  | F | LSC; IGS (clpP-psbB) | 31 | 73886 | 73917 | LSC; IGS (clpP-psbB) | -3 | 1.85E-04 |
| *Cephalantheropsis obcordata* | 31 | 51432 | 51463 |  | F | LSC; IGS (ndhC-trnV-UAC) | 31 | 85092 | 85123 | LSC; CDS (rpl16 intron) | -3 | 1.85E-04 |
| *Cephalantheropsis obcordata* | 53 | 64535 | 64588 |  | P | LSC; IGS (petA-psbJ ) | 53 | 64535 | 64588 | LSC; IGS (petA-psbJ ) | -3 | 5.47E-17 |
| *Cephalantheropsis obcordata* | 48 | 30155 | 30203 |  | P | LSC; IGS (petN-pabM) | 48 | 30155 | 30203 | LSC; IGS (petN-pabM) | 0 | 8.85E-20 |
| *Cephalantheropsis obcordata* | 46 | 42882 | 42928 |  | P | LSC; IGS (psaA-ycf3) | 46 | 42882 | 42928 | LSC; IGS (psaA-ycf3) | -2 | 1.32E-14 |
| *Cephalantheropsis obcordata* | 32 | 36442 | 36474 |  | P | LSC; IGS (psbC-trnS-UGA);tRNA(trnS-UGA) | 32 | 45796 | 45828 | LSC; IGS (trnS-GAA-rps4);tRNA(trnS-GAA) | -3 | 5.09E-05 |
| *Cephalantheropsis obcordata* | 31 | 8321 | 8352 |  | P | LSC; IGS (psbI-trnS-GCU);tRNA(trnS-GCU) | 31 | 45794 | 45825 | LSC; tRNA (trnS-GGA) | -1 | 1.41E-07 |
| *Cephalantheropsis obcordata* | 32 | 28224 | 28256 |  | P | LSC; IGS (rpoB-trnC-GCA) | 32 | 28224 | 28256 | LSC; IGS (rpoB-trnC-GCA) | 0 | 3.80E-10 |
| *Cephalantheropsis obcordata* | 40 | 4 | 44 |  | P | LSC; IGS (rps19-psbA) | 40 | 4 | 44 | LSC; IGS (rps19-psbA) | 0 | 5.80E-15 |
| *Cephalantheropsis obcordata* | 40 | 83288 | 83328 |  | P | LSC; IGS (rps8-rpl14);CDS(rpl14) | 40 | 83288 | 83328 | LSC; IGS (rps8-rpl14);CDS(rpl14) | -2 | 4.07E-11 |
| *Cephalantheropsis obcordata* | 52 | 29234 | 29286 |  | P | LSC; IGS (trnC-GCA-petN) | 52 | 29234 | 29286 | LSC; IGS (trnC-GCA-petN) | 0 | 3.46E-22 |
| *Cephalantheropsis obcordata* | 36 | 32643 | 32679 |  | P | LSC; IGS (trnE-UUC-trnT-GGU) | 36 | 32643 | 32679 | LSC; IGS (trnE-UUC-trnT-GGU) | -2 | 8.42E-09 |
| *Cephalantheropsis obcordata* | 30 | 32312 | 32342 |  | R | LSC; IGS (trnE-UUC-trnT-GGU) | 30 | 47348 | 47378 | LSC; IGS (trnT-UGU-trnL-UAA) | -3 | 6.67E-04 |
| *Cephalantheropsis obcordata* | 32 | 49320 | 49352 |  | P | LSC; IGS (trnF-GAA-ndhJ) | 32 | 49356 | 49388 | LSC; IGS (trnF-GAA-ndhJ);CDS(ndhJ) | -2 | 1.70E-06 |
| *Cephalantheropsis obcordata* | 36 | 37284 | 37320 |  | P | LSC; IGS (trnG-UCC-trnfM-CAU) | 36 | 37284 | 37320 | LSC; IGS (trnG-UCC-trnfM-CAU) | 0 | 1.49E-12 |
| *Cephalantheropsis obcordata* | 30 | 4482 | 4512 |  | F | LSC; IGS (trnK-UUU-rps16) | 30 | 4495 | 4525 | LSC; IGS (trnK-UUU-rps16) | -1 | 5.48E-07 |
| *Cephalantheropsis obcordata* | 30 | 10168 | 10198 |  | F | LSC; tRNA (trnG-GCC) | 30 | 37220 | 37250 | LSC; tRNA (trnG-UCC) | -3 | 6.67E-04 |
| *Cephalantheropsis obcordata* | 31 | 3706 | 3737 |  | R | LSC; tRNA (trnK-UUU) | 31 | 3714 | 3745 | LSC; tRNA (trnK-UUU) | -3 | 1.85E-04 |
| *Cephalantheropsis obcordata* | 37 | 128170 | 128207 |  | P | SSC; CDS (ycf1) | 37 | 128170 | 128207 | SSC; CDS (ycf1) | -3 | 7.79E-08 |
| *Cephalantheropsis obcordata* | 30 | 128001 | 128031 |  | P | SSC; CDS (ycf1) | 30 | 128001 | 128031 | SSC; CDS (ycf1) | -2 | 2.38E-05 |
| *Cephalantheropsis obcordata* | 32 | 115598 | 115630 |  | F | SSC; IGS (ndhF-rpl32) | 32 | 115618 | 115650 | SSC; IGS (ndhF-rpl32) | -3 | 5.09E-05 |
| *Cephalantheropsis obcordata* | 30 | 115911 | 115941 |  | P | SSC; IGS (ndhF-rpl32) | 30 | 115913 | 115943 | SSC; IGS (ndhF-rpl32) | -1 | 5.48E-07 |
| *Cephalantheropsis obcordata* | 37 | 126800 | 126837 |  | P | SSC; IGS (rps15-ycf1) | 37 | 126800 | 126837 | SSC; IGS (rps15-ycf1) | -3 | 7.79E-08 |
| *Phaius tankervilliae* | 37 | 102355 | 102392 |  | F | IR1; IGS (rps12-trnV-GAC) | 37 | 102386 | 102423 | IR1; IGS (rps12-trnV-GAC) | -1 | 4.14E-11 |
| *Phaius tankervilliae* | 37 | 102355 | 102392 |  | P | IR1; IGS (rps12-trnV-GAC) | 37 | 142444 | 142481 | IR2; IGS (trnV-GAC-rps7) | -1 | 4.14E-11 |
| *Phaius tankervilliae* | 37 | 102386 | 102423 |  | P | IR1; IGS (rps12-trnV-GAC) | 37 | 142475 | 142512 | IR2; IGS (trnV-GAC-rps7) | -1 | 4.14E-11 |
| *Phaius tankervilliae* | 31 | 150111 | 150142 |  | F | IR2; CDS (ycf2) | 31 | 150129 | 150160 | IR2; CDS (ycf2) | 0 | 1.53E-09 |
| *Phaius tankervilliae* | 37 | 142444 | 142481 |  | F | IR2; IGS (trnV-GAC-rps7) | 37 | 142475 | 142512 | IR2; IGS (trnV-GAC-rps7) | -1 | 4.14E-11 |
| *Phaius tankervilliae* | 30 | 39219 | 39249 |  | F | LSC; CDS (psaB) | 30 | 41443 | 41473 | LSC; CDS (psaA) | -3 | 6.70E-04 |
| *Phaius tankervilliae* | 40 | 5212 | 5252 |  | P | LSC; CDS (rps16) | 40 | 5212 | 5252 | LSC; CDS (rps16) | 0 | 5.82E-15 |
| *Phaius tankervilliae* | 31 | 94707 | 94738 |  | F | LSC; CDS (ycf2) | 31 | 94725 | 94756 | LSC; CDS (ycf2) | 0 | 1.53E-09 |
| *Phaius tankervilliae* | 31 | 94707 | 94738 |  | P | LSC; CDS (ycf2) | 31 | 150111 | 150142 | IR2; CDS (ycf2) | 0 | 1.53E-09 |
| *Phaius tankervilliae* | 31 | 94725 | 94756 |  | P | LSC; CDS (ycf2) | 31 | 150129 | 150160 | IR2; CDS (ycf2) | 0 | 1.53E-09 |
| *Phaius tankervilliae* | 39 | 44106 | 44145 |  | F | LSC; CDS (ycf3 intron) | 39 | 101995 | 102034 | IR1; IGS (rps12-trnV-GAC) | -2 | 1.55E-10 |
| *Phaius tankervilliae* | 30 | 44118 | 44148 |  | F | LSC; CDS (ycf3 intron) | 30 | 102007 | 102037 | LSC; IGS (rps12-trnV-GAC) | -3 | 6.70E-04 |
| *Phaius tankervilliae* | 39 | 44106 | 44145 |  | P | LSC; CDS (ycf3 intron) | 39 | 142833 | 142872 | IR2; IGS (trnV-GAC-rps7) | -2 | 1.55E-10 |
| *Phaius tankervilliae* | 30 | 44118 | 44148 |  | P | LSC; CDS (ycf3 intron) | 30 | 142830 | 142860 | IR2;CDS (rps12 intron) | -3 | 6.70E-04 |
| *Phaius tankervilliae* | 30 | 55418 | 55448 |  | F | LSC; IGS (atpB-rbcL) | 30 | 65030 | 65060 | LSC; IGS (petA-psbJ) | -3 | 6.70E-04 |
| *Phaius tankervilliae* | 32 | 115831 | 115863 |  | P | LSC; IGS (ndhF-rpl32) | 32 | 116117 | 116149 | LSC; IGS (ndhF-rpl32) | -2 | 1.70E-06 |
| *Phaius tankervilliae* | 53 | 64716 | 64769 |  | P | LSC; IGS (petA-psbJ) | 53 | 64716 | 64769 | LSC; IGS (petA-psbJ) | -3 | 5.49E-17 |
| *Phaius tankervilliae* | 48 | 30076 | 30124 |  | P | LSC; IGS (petN-pabM) | 48 | 30076 | 30124 | LSC; IGS (petN-pabM) | -2 | 9.02E-16 |
| *Phaius tankervilliae* | 32 | 36225 | 36257 |  | P | LSC; IGS (psbC-trnS-UGA);tRNA(trnS-UGA) | 32 | 45540 | 45572 | LSC; tRNA (trnS-GGA) | -3 | 5.11E-05 |
| *Phaius tankervilliae* | 30 | 77358 | 77388 |  | F | LSC; IGS (psbH-petB) | 30 | 77380 | 77410 | LSC; IGS (psbH-petB) | -3 | 6.70E-04 |
| *Phaius tankervilliae* | 30 | 8273 | 8303 |  | P | LSC; IGS (psbl-trnS-GCU);tRNA(trnS-GCU) | 30 | 45540 | 45570 | LSC; tRNA (trnS-GGA) | -1 | 5.50E-07 |
| *Phaius tankervilliae* | 30 | 57862 | 57892 |  | F | LSC; IGS (rbcL-accD) | 30 | 57874 | 57904 | LSC; IGS (rbcL-accD) | -3 | 6.70E-04 |
| *Phaius tankervilliae* | 32 | 28187 | 28219 |  | P | LSC; IGS (rpoB-trnC-GCA) | 32 | 28187 | 28219 | LSC; IGS (rpoB-trnC-GCA) | 0 | 3.82E-10 |
| *Phaius tankervilliae* | 40 | 6 | 46 |  | P | LSC; IGS (rps19-psbA) | 40 | 6 | 46 | LSC; IGS (rps19-psbA) | 0 | 5.82E-15 |
| *Phaius tankervilliae* | 30 | 83484 | 83514 |  | P | LSC; IGS (rps8-rpl14);CDS(rpl14) | 30 | 83484 | 83514 | LSC; IGS (rps8-rpl14);CDS(rpl14) | 0 | 6.11E-09 |
| *Phaius tankervilliae* | 30 | 29188 | 29218 |  | P | LSC; IGS (trnC-GCA-petN) | 30 | 29188 | 29218 | LSC; IGS (trnC-GCA-petN) | -2 | 2.39E-05 |
| *Phaius tankervilliae* | 36 | 32209 | 32245 |  | F | LSC; IGS (trnE-UUC-trnT-GGU) | 36 | 32226 | 32262 | LSC; IGS (trnE-UUC-trnT-GGU) | 0 | 1.49E-12 |
| *Phaius tankervilliae* | 34 | 32173 | 32207 |  | F | LSC; IGS (trnE-UUC-trnT-GGU) | 34 | 32188 | 32222 | LSC; IGS (trnE-UUC-trnT-GGU) | 0 | 2.39E-11 |
| *Phaius tankervilliae* | 32 | 49145 | 49177 |  | P | LSC; IGS (trnF-GAA-ndhJ) | 32 | 49181 | 49213 | LSC; IGS (trnF-GAA-ndhJ);CDS(ndhJ) | -3 | 5.11E-05 |
| *Phaius tankervilliae* | 30 | 10136 | 10166 |  | F | LSC; tRNA (trnG-GCC) | 30 | 36991 | 37021 | LSC; tRNA (trnG-UCC) | -3 | 6.70E-04 |
| *Phaius tankervilliae* | 31 | 3411 | 3442 |  | P | LSC; tRNA (trnK-UUU intron) | 31 | 3411 | 3442 | LSC; tRNA (trnK-UUU intron) | -3 | 1.85E-04 |
| *Phaius tankervilliae* | 34 | 124600 | 124634 |  | P | SSC; CDS (ndhA intron) | 34 | 124600 | 124634 | SSC; CDS (ndhA intron) | 0 | 2.39E-11 |
| *Phaius tankervilliae* | 37 | 128314 | 128351 |  | P | SSC; CDS (ycf1) | 37 | 128314 | 128351 | SSC; CDS (ycf1) | -3 | 7.82E-08 |
| *Phaius tankervilliae* | 30 | 12863 | 12893 |  | R | SSC; CDS (ycf1) | 30 | 68804 | 68834 | LSC; IGS (psaJ-rpl33) | -3 | 6.70E-04 |
| *Phaius tankervilliae* | 35 | 120885 | 120920 |  | P | SSC; IGS (psaC-ndhE) | 35 | 120923 | 120958 | SSC; IGS (psaC-ndhE) | -3 | 1.05E-06 |
| *Phaius tankervilliae* | 37 | 126944 | 126981 |  | P | SSC; IGS (rps15-ycf1) | 37 | 126944 | 126981 | SSC; IGS (rps15-ycf1) | -3 | 7.82E-08 |
