# Supplementary material for: Plastome structure and adaptive evolution of Calanthe s.l. species
Source: PeerJ. 2020 Oct 13;8:e10051. doi: 10.7717/peerj.10051 (PMC7566753; doi:10.7717/peerj.10051)
Supplement: Supplemental Information 5 [file peerj-08-10051-s005.docx]

### **Table S5** The tandem repeats distribution in seven *Calanthe* s.l. plastomes.

| **Species** | **Start** | **End** | **Total length** | **Consensus Size(bp)** | **Copy number** | **Region** |
| --- | --- | --- | --- | --- | --- | --- |
| *Calanthe davidii* | 5971 | 5996 | 26 | 13 | 2.0 | LSC; CDS (*rps16*) |
| *Calanthe davidii* | 6604 | 6647 | 44 | 13 | 3.4 | LSC; IGS (*rps16*-*trnQ*-UUG) |
| *Calanthe davidii* | 6680 | 6787 | 108 | 26 | 4.2 | LSC; IGS (*rps16*-*trnQ*-UUG) |
| *Calanthe davidii* | 6684 | 6787 | 104 | 6 | 16.5 | LSC; IGS (*rps16*-*trnQ*-UUG) |
| *Calanthe davidii* | 6685 | 6734 | 50 | 14 | 3.8 | LSC; IGS (*rps16*-*trnQ*-UUG) |
| *Calanthe davidii* | 9486 | 9522 | 37 | 17 | 2.1 | LSC; IGS (*trnS*-GCU-*trnG*-GCC) |
| *Calanthe davidii* | 11028 | 11052 | 25 | 11 | 2.3 | LSC; IGS (*trnG*-UCU-*atpA*) |
| *Calanthe davidii* | 28430 | 28462 | 33 | 17 | 1.9 | LSC; IGS (*rpoB*-*trnC*-GCA) |
| *Calanthe davidii* | 28615 | 28651 | 37 | 15 | 2.4 | LSC; IGS (*rpoB*-*trnC*-GCA) |
| *Calanthe davidii* | 30152 | 30195 | 44 | 11 | 4.1 | LSC; IGS (*petN*-*psbM*) |
| *Calanthe davidii* | 32873 | 32983 | 111 | 2 | 55.5 | LSC; IGS (*trnE*-UUC-*trnT*-GGU) |
| *Calanthe davidii* | 32934 | 32983 | 50 | 7 | 7.5 | LSC; IGS (*trnE*-UUC-*trnT*-GGU) |
| *Calanthe davidii* | 32934 | 32983 | 50 | 16 | 3.2 | LSC; IGS (*trnE*-UUC-*trnT*-GGU) |
| *Calanthe davidii* | 46543 | 46569 | 27 | 13 | 2.1 | LSC; IGS (*trnS*-GGA-*rps4*) |
| *Calanthe davidii* | 52314 | 52339 | 26 | 13 | 2.0 | LSC; IGS (*ndhC*-*trnV*-UAC) |
| *Calanthe davidii* | 56175 | 56208 | 34 | 16 | 2.1 | LSC; IGS (*atpB*-*rbcL*) |
| *Calanthe davidii* | 58615 | 58658 | 44 | 19 | 2.3 | LSC; IGS (*rbcL*-*accD*) |
| *Calanthe davidii* | 60905 | 60950 | 46 | 23 | 2.0 | LSC; IGS (*accD*-*psaI*) |
| *Calanthe davidii* | 62526 | 62557 | 32 | 16 | 2.0 | LSC; IGS (*ycf4*-*cemA*) |
| *Calanthe davidii* | 65411 | 65447 | 37 | 18 | 2.1 | LSC; IGS (*petA*-*psbJ*) |
| *Calanthe davidii* | 69149 | 69177 | 29 | 14 | 2.1 | LSC; IGS (*trnP*-UGG-*psaJ*) |
| *Calanthe davidii* | 70283 | 70312 | 30 | 15 | 2.0 | LSC; IGS (rp*l*33-*rps18*) |
| *Calanthe davidii* | 70319 | 70362 | 44 | 21 | 2.1 | LSC; CDS (*rps18*) |
| *Calanthe davidii* | 74019 | 74049 | 31 | 10 | 3.1 | LSC; CDS (*clpP* intron) |
| *Calanthe davidii* | 74210 | 74245 | 36 | 18 | 2.0 | LSC; CDS (*clpP* intron) |
| *Calanthe davidii* | 74679 | 74727 | 49 | 16 | 3.0 | LSC; IGS (*clpP*-*psbB*) |
| *Calanthe davidii* | 74739 | 74777 | 39 | 15 | 2.6 | LSC; IGS (*clpP*-*psbB*) |
| *Calanthe davidii* | 74968 | 75007 | 40 | 18 | 2.1 | LSC; IGS (clpP-psbB) |
| *Calanthe davidii* | 74968 | 75047 | 80 | 27 | 2.9 | LSC; IGS (clpP-psbB) |
| *Calanthe davidii* | 77197 | 77261 | 65 | 12 | 4.7 | LSC; IGS (*psbB*-*psbT*) |
| *Calanthe davidii* | 77206 | 77243 | 38 | 17 | 2.2 | LSC; IGS (*psbB*-*psbT*) |
| *Calanthe davidii* | 77478 | 77515 | 38 | 15 | 2.4 | LSC; IGS (*psbB*-*psbT*) |
| *Calanthe davidii* | 85930 | 85972 | 43 | 16 | 2.7 | LSC; CDS (*rpl16* intron) |
| *Calanthe davidii* | 86089 | 86130 | 42 | 10 | 4.3 | LSC; CDS (*rpl16* intron) |
| *Calanthe davidii* | 86093 | 86136 | 44 | 18 | 2.4 | LSC; CDS (*rpl16* intron) |
| *Calanthe davidii* | 95556 | 95616 | 61 | 18 | 3.6 | IR1; CDS (*ycf2*) |
| *Calanthe davidii* | 103346 | 103372 | 27 | 14 | 1.9 | IR1; IGS (*rps12*-*trnV*-GAC) |
| *Calanthe davidii* | 103367 | 103391 | 25 | 12 | 2.1 | IR1; IGS (*rps12*-*trnV*-GAC) |
| *Calanthe davidii* | 103395 | 103474 | 80 | 35 | 2.3 | IR1; IGS (*rps12*-*trnV*-GAC) |
| *Calanthe davidii* | 103397 | 103465 | 69 | 5 | 13.4 | IR1; IGS (*rps12*-*trnV*-GAC) |
| *Calanthe davidii* | 103397 | 103470 | 74 | 12 | 4.9 | IR1; IGS (*rps12*-*trnV*-GAC) |
| *Calanthe davidii* | 103398 | 103468 | 71 | 35 | 2.1 | IR1; IGS (*rps12*-*trnV*-GAC) |
| *Calanthe davidii* | 103407 | 103460 | 54 | 21 | 2.6 | IR1; IGS (*rps12*-*trnV*-GAC) |
| *Calanthe davidii* | 116805 | 116842 | 38 | 10 | 3.9 | SSC; IGS (*ndhF*-*rpl32*) |
| *Calanthe davidii* | 116870 | 117000 | 131 | 31 | 4.3 | SSC; IGS (*ndhF*-*rpl32*) |
| *Calanthe davidii* | 116912 | 116991 | 80 | 2 | 38.5 | SSC; IGS (*ndhF*-*rpl32*) |
| *Calanthe davidii* | 116916 | 117000 | 85 | 11 | 7.7 | SSC; IGS (*ndhF*-*rpl32*) |
| *Calanthe davidii* | 117138 | 117162 | 25 | 12 | 2.1 | SSC; IGS (*ndhF*-*rpl32*) |
| *Calanthe davidii* | 117167 | 117193 | 27 | 13 | 2.1 | SSC; IGS (*ndhF*-*rpl32*) |
| *Calanthe davidii* | 121856 | 121882 | 27 | 14 | 1.9 | SSC; IGS (*psaC*-*ndhE*) |
| *Calanthe davidii* | 121899 | 121938 | 40 | 14 | 2.9 | SSC; IGS (*psaC*-*ndhE*) |
| *Calanthe davidii* | 122083 | 122140 | 58 | 28 | 2.1 | SSC; IGS (*psaC*-*ndhE*) |
| *Calanthe davidii* | 127524 | 127603 | 80 | 11 | 7.3 | SSC; IGS (*ndhH*-*rps15*) |
| *Calanthe davidii* | 129632 | 129662 | 31 | 15 | 2.1 | SSC; CDS (*ycf1*) |
| *Calanthe davidii* | 143402 | 143475 | 74 | 12 | 4.9 | IR2;CDS (*rps12* intron) |
| *Calanthe davidii* | 143404 | 143474 | 71 | 35 | 2.1 | IR2;CDS (*rps12* intron) |
| *Calanthe davidii* | 143407 | 143475 | 69 | 5 | 13.4 | IR2;CDS (*rps12* intron) |
| *Calanthe davidii* | 143412 | 143465 | 54 | 21 | 2.7 | IR2;CDS (*rps12* intron) |
| *Calanthe davidii* | 143481 | 143505 | 25 | 12 | 2.1 | IR2;CDS (*rps12* intron) |
| *Calanthe davidii* | 143500 | 143526 | 27 | 14 | 1.9 | IR2;CDS (*rps12* intron) |
| *Calanthe davidii* | 151256 | 151316 | 61 | 18 | 3.6 | IR2; CDS (*ycf2*) |
| *Calanthe davidii* | 151260 | 151304 | 45 | 9 | 5.0 | IR2; CDS (*ycf2*) |
| *Calanthe delavayi* | 1612 | 1644 | 33 | 10 | 3.4 | LSC; tRNA (*trnK*-UUU) |
| *Calanthe delavayi* | 3415 | 3439 | 25 | 13 | 1.9 | LSC; tRNA (*trnK*-UUU) |
| *Calanthe delavayi* | 4572 | 4599 | 28 | 14 | 2.0 | LSC; IGS (*trnK*-UUU-*rps16*) |
| *Calanthe delavayi* | 6465 | 6493 | 29 | 11 | 2.6 | LSC; IGS (*rps16*-*trnQ*-UUG) |
| *Calanthe delavayi* | 6499 | 6579 | 81 | 8 | 10.1 | LSC; IGS (*rps16*-*trnQ*-UUG) |
| *Calanthe delavayi* | 6511 | 6568 | 58 | 20 | 3.0 | LSC; IGS (*rps16*-*trnQ*-UUG) |
| *Calanthe delavayi* | 6544 | 6591 | 48 | 12 | 3.3 | LSC; IGS (*rps16*-*trnQ*-UUG) |
| *Calanthe delavayi* | 8903 | 8928 | 26 | 11 | 2.4 | LSC;IGS (*trnS*-GCU-*trnG*-GCC) |
| *Calanthe delavayi* | 8919 | 8958 | 40 | 13 | 3.2 | LSC;IGS (*trnS*-GCU-*trnG*-GCC) |
| *Calanthe delavayi* | 9049 | 9086 | 38 | 18 | 2.1 | LSC;IGS (*trnS*-GCU-*trnG*-GCC) |
| *Calanthe delavayi* | 9221 | 9257 | 37 | 17 | 2.1 | LSC;IGS (*trnS*-GCU-*trnG*-GCC) |
| *Calanthe delavayi* | 27968 | 28001 | 34 | 17 | 2.0 | LSC; IGS (*rpoB*-*trnC*-GCA) |
| *Calanthe delavayi* | 29541 | 29626 | 86 | 39 | 2.2 | LSC; IGS (*petN*-*psbM*) |
| *Calanthe delavayi* | 29582 | 29633 | 52 | 11 | 4.9 | LSC; IGS (*petN*-*psbM*) |
| *Calanthe delavayi* | 32179 | 32229 | 51 | 18 | 2.5 | LSC; IGS (*trnE*-UUC-*trnT*-GGU) |
| *Calanthe delavayi* | 32185 | 32228 | 44 | 18 | 2.3 | LSC; IGS (*trnE*-UUC-*trnT*-GGU) |
| *Calanthe delavayi* | 32223 | 32284 | 62 | 26 | 2.3 | LSC; IGS (*trnE*-UUC-*trnT*-GGU) |
| *Calanthe delavayi* | 32233 | 32281 | 49 | 22 | 2.3 | LSC; IGS (*trnE*-UUC-*trnT*-GGU) |
| *Calanthe delavayi* | 32259 | 32286 | 28 | 14 | 2.0 | LSC; IGS (*trnE*-UUC-*trnT*-GGU) |
| *Calanthe delavayi* | 35954 | 35990 | 37 | 16 | 2.3 | LSC; IGS (*psbC*-*trnS*-UGA) |
| *Calanthe delavayi* | 49383 | 49420 | 38 | 13 | 2.8 | LSC; IGS (*ndhJ*-*trnV*-UAC) |
| *Calanthe delavayi* | 49432 | 49462 | 31 | 15 | 2.1 | LSC; IGS (*ndhJ*-*trnV*-UAC) |
| *Calanthe delavayi* | 53028 | 53078 | 51 | 16 | 3.1 | LSC; IGS (*atpB*-*rbcL*) |
| *Calanthe delavayi* | 53039 | 53069 | 31 | 8 | 4.0 | LSC; IGS (*atpB*-*rbcL*) |
| *Calanthe delavayi* | 53161 | 53229 | 69 | 18 | 3.5 | LSC; IGS (*atpB*-*rbcL*) |
| *Calanthe delavayi* | 53161 | 53239 | 79 | 41 | 1.9 | LSC; IGS (*atpB*-*rbcL*) |
| *Calanthe delavayi* | 58685 | 58716 | 32 | 16 | 2.0 | LSC; IGS (*ycf4*-*cemA*) |
| *Calanthe delavayi* | 61727 | 61780 | 54 | 19 | 3.1 | LSC; IGS (*petA*-*psbJ*) |
| *Calanthe delavayi* | 61740 | 61774 | 35 | 13 | 2.6 | LSC; IGS (*petA*-*psbJ*) |
| *Calanthe delavayi* | 61780 | 61833 | 54 | 2 | 29.0 | LSC; IGS (*petA*-*psbJ*) |
| *Calanthe delavayi* | 66334 | 66363 | 30 | 15 | 2.0 | LSC; IGS (*rpl33*-*rps18*) |
| *Calanthe delavayi* | 66370 | 66413 | 44 | 21 | 2.1 | LSC; CDS (*rps18*) |
| *Calanthe delavayi* | 70076 | 70108 | 33 | 11 | 3.1 | LSC; CDS (*clpP* intron) |
| *Calanthe delavayi* | 70077 | 70108 | 32 | 10 | 3.1 | LSC; CDS (*clpP* intron) |
| *Calanthe delavayi* | 70735 | 70765 | 31 | 16 | 2.0 | LSC; IGS (*clpP*-*psbB*) |
| *Calanthe delavayi* | 70888 | 70949 | 62 | 27 | 2.3 | LSC; IGS (*clpP*-*psbB*) |
| *Calanthe delavayi* | 73023 | 73054 | 32 | 16 | 2.0 | LSC; IGS (*psbB*-*psbT*) |
| *Calanthe delavayi* | 73036 | 73091 | 56 | 21 | 2.6 | LSC; IGS (*psbB*-*psbT*) |
| *Calanthe delavayi* | 73132 | 73183 | 52 | 2 | 24.0 | LSC; IGS (*psbB*-*psbT*) |
| *Calanthe delavayi* | 73138 | 73172 | 35 | 9 | 3.9 | LSC; IGS (*psbB*-*psbT*) |
| *Calanthe delavayi* | 73139 | 73181 | 43 | 18 | 2.4 | LSC; IGS (*psbB*-*psbT*) |
| *Calanthe delavayi* | 78637 | 78685 | 49 | 24 | 2.0 | LSC; CDS (*rps11*) |
| *Calanthe delavayi* | 81577 | 81619 | 43 | 16 | 2.7 | LSC; CDS (*rpl16* intron) |
| *Calanthe delavayi* | 81723 | 81798 | 76 | 18 | 4.2 | LSC; CDS (*rpl16* intron) |
| *Calanthe delavayi* | 81754 | 81792 | 39 | 11 | 3.9 | LSC; CDS (*rpl16* intron) |
| *Calanthe delavayi* | 83586 | 83642 | 57 | 23 | 2.7 | LSC; IGS (*rpl22*-*rps19*) |
| *Calanthe delavayi* | 83597 | 83642 | 46 | 23 | 2.0 | LSC;IGS (*rpl22*-*rps19*) |
| *Calanthe delavayi* | 91259 | 91319 | 61 | 18 | 3.6 | IR1;CDS (*ycf2*) |
| *Calanthe delavayi* | 98818 | 98858 | 41 | 14 | 2.9 | IR1; IGS (*rps12*-*trnV*-GAC) |
| *Calanthe delavayi* | 98860 | 98896 | 37 | 13 | 2.9 | IR1; IGS (*rps12*-*trnV*-GAC) |
| *Calanthe delavayi* | 98860 | 98898 | 39 | 10 | 3.8 | IR1; IGS (*rps12*-*trnV*-GAC) |
| *Calanthe delavayi* | 98860 | 98899 | 40 | 18 | 2.2 | IR1; IGS (*rps12*-*trnV*-GAC) |
| *Calanthe delavayi* | 98889 | 98945 | 57 | 27 | 2.1 | IR1; IGS (*rps12*-*trnV*-GAC) |
| *Calanthe delavayi* | 108826 | 108885 | 60 | 20 | 3.0 | SSC; IGS (*trnN*-GUU-*rpl32*) |
| *Calanthe delavayi* | 108868 | 108978 | 111 | 37 | 3.0 | SSC; IGS (*trnN*-GUU-*rpl32*) |
| *Calanthe delavayi* | 109041 | 109072 | 32 | 15 | 2.1 | SSC; IGS (*trnN*-GUU-*rpl32*) |
| *Calanthe delavayi* | 111672 | 111708 | 37 | 19 | 1.9 | SSC; IGS (*ccsA*-*ndhD*) |
| *Calanthe delavayi* | 113979 | 114036 | 58 | 28 | 2.1 | IR1; IGS (*psaC*-*ndhE*) |
| *Calanthe delavayi* | 134694 | 134733 | 40 | 18 | 2.2 | IR2; CDS(*rps12* intron) |
| *Calanthe delavayi* | 134695 | 134733 | 39 | 10 | 3.8 | IR2; CDS(*rps12* intron) |
| *Calanthe delavayi* | 134735 | 134775 | 41 | 14 | 2.9 | IR2; CDS(*rps12* intron) |
| *Calanthe delavayi* | 142274 | 142334 | 61 | 18 | 3.6 | IR2; CDS (*ycf2*) |
| *Calanthe delavayi* | 142278 | 142322 | 45 | 9 | 5.0 | IR2; CDS (*ycf2*) |
| *Calanthe delavayi* | 149951 | 149996 | 46 | 23 | 2.0 | IR2; CDS (*ycf2*) |
| *Calanthe delavayi* | 149951 | 150007 | 57 | 23 | 2.7 | IR2; CDS (*ycf2*) |
| *Styloglossum lyroglossa* | 4215 | 4264 | 50 | 20 | 2.4 | LSC; tRNA (*trnK*-UUU) |
| *Styloglossum lyroglossa* | 5253 | 5286 | 34 | 13 | 2.5 | LSC; CDS (*rps16* intron) |
| *Styloglossum lyroglossa* | 5278 | 5316 | 39 | 19 | 2.1 | LSC; CDS (*rps16* intron) |
| *Styloglossum lyroglossa* | 6454 | 6487 | 34 | 14 | 2.3 | LSC; IGS (*rps16*-*trnQ*-UUG) |
| *Styloglossum lyroglossa* | 6484 | 6534 | 51 | 22 | 2.5 | LSC; IGS (*rps16*-*trnQ*-UUG) |
| *Styloglossum lyroglossa* | 6486 | 6531 | 46 | 14 | 3.4 | LSC; IGS (*rps16*-*trnQ*-UUG) |
| *Styloglossum lyroglossa* | 6489 | 6528 | 40 | 12 | 3.1 | LSC; IGS (*rps16*-*trnQ*-UUG) |
| *Styloglossum lyroglossa* | 14307 | 14338 | 32 | 16 | 2.0 | LSC; IGS (*atpH*-*atpI*) |
| *Styloglossum lyroglossa* | 28304 | 28340 | 37 | 15 | 2.4 | LSC; IGS (*rpoB*-*trnC*-GCA) |
| *Styloglossum lyroglossa* | 28649 | 28711 | 63 | 21 | 3.0 | LSC; IGS (*rpoB*-*trnC*-GCA) |
| *Styloglossum lyroglossa* | 28658 | 28711 | 54 | 15 | 3.7 | LSC; IGS (*rpoB*-*trnC*-GCA) |
| *Styloglossum lyroglossa* | 50939 | 50964 | 26 | 13 | 2.0 | LSC; IGS (*ndhC*-*trnV*-UAC) |
| *Styloglossum lyroglossa* | 55020 | 55064 | 45 | 23 | 1.9 | LSC; IGS (*atpB*-*rbcL*) |
| *Styloglossum lyroglossa* | 55051 | 55098 | 48 | 22 | 2.1 | LSC; IGS (*atpB*-*rbcL*) |
| *Styloglossum lyroglossa* | 63503 | 63535 | 33 | 16 | 2.1 | LSC; IGS (*petA*-*psbJ*) |
| *Styloglossum lyroglossa* | 63543 | 63593 | 51 | 13 | 3.8 | LSC; IGS (*petA*-*psbJ*) |
| *Styloglossum lyroglossa* | 63544 | 63596 | 53 | 21 | 2.5 | LSC; IGS (*petA*-*psbJ*) |
| *Styloglossum lyroglossa* | 63550 | 63582 | 33 | 15 | 2.2 | LSC; IGS (*petA*-*psbJ*) |
| *Styloglossum lyroglossa* | 68203 | 68232 | 30 | 15 | 2.0 | LSC; IGS (*rpl33*-*rps18*) |
| *Styloglossum lyroglossa* | 68239 | 68283 | 45 | 21 | 2.1 | LSC; IGS (*rpl33*-*rps18*) |
| *Styloglossum lyroglossa* | 72702 | 72763 | 62 | 27 | 2.3 | LSC; IGS (*clpP*-*psbB*) |
| *Styloglossum lyroglossa* | 74793 | 74836 | 44 | 13 | 3.2 | LSC; IGS (*psbB*-*psbT*) |
| *Styloglossum lyroglossa* | 74803 | 74850 | 48 | 15 | 3.3 | LSC; IGS (*psbB*-*psbT*) |
| *Styloglossum lyroglossa* | 74941 | 74988 | 48 | 12 | 3.7 | LSC; IGS (*psbB*-*psbT*) |
| *Styloglossum lyroglossa* | 78347 | 78371 | 25 | 12 | 2.1 | LSC; CDS (*petD* intron) |
| *Styloglossum lyroglossa* | 80654 | 80702 | 49 | 24 | 2.0 | LSC; CDS (*rps11*) |
| *Styloglossum lyroglossa* | 83650 | 83692 | 43 | 16 | 2.7 | LSC; CDS (*rpl16* intron) |
| *Styloglossum lyroglossa* | 83808 | 83848 | 41 | 18 | 2.3 | LSC; CDS (*rpl16* intron) |
| *Styloglossum lyroglossa* | 93232 | 93292 | 61 | 18 | 3.6 | IR1; CDS (*ycf2*) |
| *Styloglossum lyroglossa* | 101079 | 101112 | 34 | 12 | 2.8 | IR1; IGS (*rps12*-*trnV*-GAC) |
| *Styloglossum lyroglossa* | 101091 | 101128 | 38 | 19 | 2.0 | IR1; IGS (*rps12*-*trnV*-GAC) |
| *Styloglossum lyroglossa* | 114090 | 114141 | 52 | 26 | 2.0 | SSC; IGS (*ndhF*-*rpl32*) |
| *Styloglossum lyroglossa* | 114512 | 114551 | 40 | 17 | 2.4 | SSC; IGS (*ndhF*-*rpl32*) |
| *Styloglossum lyroglossa* | 116948 | 116984 | 37 | 19 | 1.9 | SSC; IGS (*ccsA*-*ndhD*);CDS(*ndhD*) |
| *Styloglossum lyroglossa* | 119261 | 119318 | 58 | 28 | 2.1 | SSC; IGS (*psaC*-*ndhE*) |
| *Styloglossum lyroglossa* | 140439 | 140499 | 61 | 13 | 4.6 | IR2; CDS (*rps12* intron) |
| *Styloglossum lyroglossa* | 140450 | 140484 | 35 | 12 | 2.8 | IR2; CDS (*rps12* intron) |
| *Styloglossum lyroglossa* | 148271 | 148331 | 61 | 18 | 3.6 | IR2; CDS (*ycf2*) |
| *Styloglossum lyroglossa* | 148275 | 148319 | 45 | 9 | 5.0 | IR2; CDS (*ycf2*) |
| *Preptanthe rubens* | 193 | 230 | 38 | 10 | 4.0 | LSC; IGS (*rps19*-*psbA*) |
| *Preptanthe rubens* | 3743 | 3767 | 25 | 12 | 2.1 | LSC; tRNA (*trnK*-UUU) |
| *Preptanthe rubens* | 4281 | 4308 | 28 | 14 | 2.0 | LSC; tRNA (*trnK*-UUU) |
| *Preptanthe rubens* | 5325 | 5363 | 39 | 11 | 3.9 | LSC; CDS (*rps16* intron) |
| *Preptanthe rubens* | 6705 | 6775 | 71 | 8 | 10.1 | LSC; IGS (*rps16*-*trnQ*-UUG) |
| *Preptanthe rubens* | 6710 | 6771 | 62 | 14 | 4.1 | LSC; IGS (*rps16*-*trnQ*-UUG) |
| *Preptanthe rubens* | 6715 | 6776 | 62 | 21 | 2.8 | LSC; IGS (*rps16*-*trnQ*-UUG) |
| *Preptanthe rubens* | 6737 | 6775 | 39 | 14 | 3.0 | LSC; IGS (*rps16*-*trnQ*-UUG) |
| *Preptanthe rubens* | 6794 | 6849 | 56 | 16 | 3.1 | LSC; IGS (*rps16*-*trnQ*-UUG) |
| *Preptanthe rubens* | 9127 | 9155 | 29 | 14 | 2.1 | LSC; IGS (*trnS*-GCU-*trnG*-GCC) |
| *Preptanthe rubens* | 9172 | 9210 | 39 | 15 | 2.6 | LSC; IGS (*trnS*-GCU-*trnG*-GCC) |
| *Preptanthe rubens* | 13241 | 13268 | 28 | 13 | 2.2 | LSC; CDS (*atpF* intron) |
| *Preptanthe rubens* | 28252 | 28285 | 34 | 17 | 2.0 | LSC; IGS (*rpoB*-*trnC*-GCA) |
| *Preptanthe rubens* | 28418 | 28464 | 47 | 13 | 3.6 | LSC; IGS (*rpoB*-*trnC*-GCA) |
| *Preptanthe rubens* | 28464 | 28508 | 45 | 16 | 2.9 | LSC; IGS (*rpoB*-*trnC*-GCA) |
| *Preptanthe rubens* | 32133 | 32173 | 41 | 21 | 2.0 | LSC; IGS (*trnE*-UUC-*trnT*-GGU) |
| *Preptanthe rubens* | 32161 | 32248 | 88 | 2 | 40.5 | LSC; IGS (*trnE*-UUC-*trnT*-GGU) |
| *Preptanthe rubens* | 32161 | 32248 | 88 | 9 | 10.1 | LSC; IGS (*trnE*-UUC-*trnT*-GGU) |
| *Preptanthe rubens* | 32162 | 32248 | 87 | 20 | 4.2 | LSC; IGS (*trnE*-UUC-*trnT*-GGU) |
| *Preptanthe rubens* | 32168 | 32248 | 81 | 18 | 4.5 | LSC; IGS (*trnE*-UUC-*trnT*-GGU) |
| *Preptanthe rubens* | 47875 | 47936 | 62 | 18 | 3.4 | LSC; tRNA (*trnL*-UAA) |
| *Preptanthe rubens* | 47968 | 47992 | 25 | 11 | 2.3 | LSC; tRNA (*trnL*-UAA) |
| *Preptanthe rubens* | 51462 | 51493 | 32 | 16 | 2.0 | LSC; IGS (*ndhC*-*trnV*-UAC) |
| *Preptanthe rubens* | 51687 | 51715 | 29 | 13 | 2.3 | LSC; IGS (*ndhC*-*trnV*-UAC) |
| *Preptanthe rubens* | 52098 | 52122 | 25 | 13 | 1.9 | LSC; IGS (*ndhC*-*trnV*-UAC) |
| *Preptanthe rubens* | 56025 | 56081 | 57 | 23 | 2.4 | LSC; IGS (*atpB*-*rbcL*) |
| *Preptanthe rubens* | 56086 | 56116 | 31 | 16 | 1.9 | LSC; IGS (*atpB*-*rbcL*) |
| *Preptanthe rubens* | 56163 | 56238 | 76 | 18 | 3.8 | LSC; IGS (*atpB*-*rbcL*) |
| *Preptanthe rubens* | 65301 | 65341 | 41 | 21 | 2.0 | LSC; IGS (*petA*-*psbJ*) |
| *Preptanthe rubens* | 65309 | 65373 | 65 | 16 | 3.8 | LSC; IGS (*petA*-*psbJ*) |
| *Preptanthe rubens* | 70032 | 70075 | 44 | 21 | 2.1 | LSC; CDS (*rps18*) |
| *Preptanthe rubens* | 74224 | 74318 | 95 | 35 | 2.6 | LSC; IGS (*clpP*-*psbB*) |
| *Preptanthe rubens* | 74224 | 74386 | 163 | 20 | 8.3 | LSC; IGS (*clpP*-*psbB*) |
| *Preptanthe rubens* | 74230 | 74370 | 141 | 9 | 15.9 | LSC; IGS (*clpP*-*psbB*) |
| *Preptanthe rubens* | 74230 | 74370 | 141 | 20 | 7.4 | LSC; IGS (*clpP*-*psbB*) |
| *Preptanthe rubens* | 76775 | 76836 | 62 | 25 | 2.3 | LSC; IGS (*psbB*-*psbT*) |
| *Preptanthe rubens* | 76778 | 76834 | 57 | 26 | 2.2 | LSC; IGS (*psbB*-*psbT*) |
| *Preptanthe rubens* | 76778 | 76835 | 58 | 12 | 4.5 | LSC; IGS (*psbB*-*psbT*) |
| *Preptanthe rubens* | 77035 | 77079 | 45 | 24 | 2.0 | LSC; IGS (*psbB*-*psbT*) |
| *Preptanthe rubens* | 82468 | 82516 | 49 | 24 | 2.0 | LSC; CDS (*rps11*) |
| *Preptanthe rubens* | 83913 | 83943 | 31 | 15 | 2.1 | LSC; IGS (*rps8*-*rpl14*) |
| *Preptanthe rubens* | 85599 | 85651 | 53 | 18 | 2.8 | LSC; CDS (*rpl16* intron) |
| *Preptanthe rubens* | 95150 | 95204 | 55 | 9 | 6.1 | IR1; CDS (*ycf2*) |
| *Preptanthe rubens* | 95157 | 95204 | 48 | 18 | 2.7 | IR1; CDS (*ycf2*) |
| *Preptanthe rubens* | 116325 | 116374 | 50 | 14 | 3.5 | SSC; IGS (*ndhF*-*rpl32*) |
| *Preptanthe rubens* | 116341 | 116385 | 45 | 7 | 6.9 | SSC; IGS (*ndhF*-*rpl32*) |
| *Preptanthe rubens* | 116341 | 116387 | 47 | 11 | 4.6 | SSC; IGS (*ndhF*-*rpl32*) |
| *Preptanthe rubens* | 116595 | 116620 | 26 | 13 | 2.0 | SSC; IGS (*ndhF*-*rpl32*) |
| *Preptanthe rubens* | 117279 | 117311 | 33 | 12 | 2.8 | SSC; IGS (*rpl32*-*trnL*-UAG) |
| *Preptanthe rubens* | 119135 | 119171 | 37 | 19 | 1.9 | SSC; IGS (*ccsA*-*ndhD*);CDS(*ndhD*) |
| *Preptanthe rubens* | 121466 | 121499 | 34 | 14 | 2.3 | SSC; IGS (*psaC*-*ndhE*) |
| *Preptanthe rubens* | 129048 | 129078 | 31 | 15 | 2.1 | SSC; CDS (*ycf1*) |
| *Preptanthe rubens* | 150614 | 150661 | 48 | 18 | 2.7 | IR2; CDS (*ycf2*) |
| *Preptanthe rubens* | 150614 | 150668 | 55 | 9 | 6.1 | IR2; CDS (*ycf2*) |
| *Calanthe triplicata* | 1649 | 1685 | 37 | 19 | 2.0 | LSC; tRNA (*trnK*-UUU) |
| *Calanthe triplicata* | 3452 | 3486 | 35 | 16 | 2.2 | LSC; tRNA (*trnK*-UUU) |
| *Calanthe triplicata* | 3792 | 3847 | 56 | 24 | 2.3 | LSC; tRNA (*trnK*-UUU) |
| *Calanthe triplicata* | 4517 | 4562 | 46 | 20 | 2.2 | LSC; IGS (*trnK*-UUU-*rps16*) |
| *Calanthe triplicata* | 5892 | 5917 | 26 | 13 | 2.0 | LSC; CDS (rpl16 intron) |
| *Calanthe triplicata* | 6471 | 6503 | 33 | 12 | 3.0 | LSC; IGS (*rps16*-*trnQ*-UUG) |
| *Calanthe triplicata* | 6600 | 6627 | 28 | 14 | 2.0 | LSC; IGS (*rps16*-*trnQ*-UUG) |
| *Calanthe triplicata* | 6605 | 6669 | 65 | 6 | 10.3 | LSC; IGS (*rps16*-*trnQ*-UUG) |
| *Calanthe triplicata* | 6605 | 6669 | 65 | 18 | 3.4 | LSC; IGS (*rps16*-*trnQ*-UUG) |
| *Calanthe triplicata* | 6626 | 6669 | 44 | 12 | 3.8 | LSC; IGS (*rps16*-*trnQ*-UUG) |
| *Calanthe triplicata* | 6667 | 6694 | 28 | 11 | 2.5 | LSC; IGS (*rps16*-*trnQ*-UUG) |
| *Calanthe triplicata* | 6731 | 6779 | 49 | 19 | 2.5 | LSC; IGS (*rps16*-*trnQ*-UUG) |
| *Calanthe triplicata* | 9114 | 9185 | 72 | 27 | 2.7 | LSC; IGS (*trnS*-GCU-*trnG*-GCC) |
| *Calanthe triplicata* | 9117 | 9146 | 30 | 15 | 2.0 | LSC; IGS (*trnS*-GCU-*trnG*-GCC) |
| *Calanthe triplicata* | 9225 | 9252 | 28 | 14 | 2.0 | LSC; IGS (*trnS*-GCU-*trnG*-GCC) |
| *Calanthe triplicata* | 9434 | 9470 | 37 | 17 | 2.1 | LSC; IGS (*trnS*-GCU-*trnG*-GCC) |
| *Calanthe triplicata* | 27845 | 27870 | 26 | 13 | 2.0 | LSC; IGS (*rpoB*-*trnC*-GCA) |
| *Calanthe triplicata* | 28323 | 28356 | 34 | 17 | 2.0 | LSC; IGS (*rpoB*-*trnC*-GCA) |
| *Calanthe triplicata* | 30077 | 30101 | 25 | 12 | 2.1 | LSC; IGS (*petN*-*psbM*) |
| *Calanthe triplicata* | 30121 | 30193 | 73 | 33 | 2.3 | LSC; IGS (*petN*-*psbM*) |
| *Calanthe triplicata* | 30172 | 30226 | 55 | 16 | 3.5 | LSC; IGS (*petN*-*psbM*) |
| *Calanthe triplicata* | 32792 | 32838 | 47 | 21 | 2.4 | LSC; IGS (*trnE*-UUC-*trnT*-GGU) |
| *Calanthe triplicata* | 32792 | 32844 | 53 | 13 | 4.0 | LSC; IGS (*trnE*-UUC-*trnT*-GGU) |
| *Calanthe triplicata* | 32794 | 32878 | 85 | 2 | 42.5 | LSC; IGS (*trnE*-UUC-*trnT*-GGU) |
| *Calanthe triplicata* | 32800 | 32859 | 60 | 13 | 4.3 | LSC; IGS (*trnE*-UUC-*trnT*-GGU) |
| *Calanthe triplicata* | 32880 | 32906 | 27 | 13 | 2.1 | LSC; IGS (*trnE*-UUC-*trnT*-GGU) |
| *Calanthe triplicata* | 33191 | 33225 | 35 | 17 | 2.1 | LSC; IGS (*trnE*-UUC-*trnT*-GGU) |
| *Calanthe triplicata* | 52297 | 52322 | 26 | 13 | 2.0 | LSC; IGS (*ndhC*-*trnV*-UAC) |
| *Calanthe triplicata* | 56151 | 56184 | 34 | 16 | 2.1 | LSC; IGS (*atpB*-*rbcL*) |
| *Calanthe triplicata* | 58630 | 58673 | 44 | 19 | 2.3 | LSC; IGS (*rbcL*-*accD*) |
| *Calanthe triplicata* | 62534 | 62565 | 32 | 16 | 2.0 | LSC; IGS (*ycf4*-*cemA*) |
| *Calanthe triplicata* | 69849 | 69878 | 30 | 15 | 2.0 | LSC; IGS (*rpl33*-*rps18*) |
| *Calanthe triplicata* | 69885 | 69928 | 44 | 21 | 2.1 | LSC; CDS (*rps18*) |
| *Calanthe triplicata* | 73221 | 73246 | 26 | 13 | 2.0 | LSC; CDS (*clpP* intron) |
| *Calanthe triplicata* | 74309 | 74343 | 35 | 15 | 2.3 | LSC; IGS (*clpP*-*p*sbB) |
| *Calanthe triplicata* | 74405 | 74439 | 35 | 17 | 2.1 | LSC; IGS (*clpP*-*psbB*) |
| *Calanthe triplicata* | 74477 | 74519 | 43 | 16 | 2.8 | LSC; IGS (*clpP*-*psbB*) |
| *Calanthe triplicata* | 74594 | 74621 | 28 | 14 | 2.0 | LSC; IGS (*clpP*-*psbB*) |
| *Calanthe triplicata* | 76590 | 76631 | 42 | 17 | 2.4 | LSC; IGS (*psbB*-*psbT*) |
| *Calanthe triplicata* | 76743 | 76807 | 65 | 12 | 4.7 | LSC; IGS (*psbB*-*psbT*) |
| *Calanthe triplicata* | 76752 | 76787 | 36 | 17 | 2.1 | LSC; IGS (*psbB*-*psbT*) |
| *Calanthe triplicata* | 77010 | 77047 | 38 | 15 | 2.4 | LSC; IGS (*psbB*-*psbT*) |
| *Calanthe triplicata* | 79825 | 79862 | 38 | 19 | 2.0 | LSC; CDS (*petD* intron) |
| *Calanthe triplicata* | 82677 | 82702 | 26 | 13 | 2.0 | LSC; IGS (*rps11*-*rpl36*) |
| *Calanthe triplicata* | 83932 | 83971 | 40 | 13 | 3.2 | LSC; IGS (*rps8*-*rpl14*) |
| *Calanthe triplicata* | 85672 | 85712 | 41 | 18 | 2.3 | LSC; CDS (*rpl16* intron) |
| *Calanthe triplicata* | 95132 | 95192 | 61 | 9 | 7.1 | IR1; CDS (*ycf2*) |
| *Calanthe triplicata* | 95145 | 95192 | 48 | 18 | 2.7 | IR1; CDS (*ycf2*) |
| *Calanthe triplicata* | 102926 | 102952 | 27 | 14 | 1.9 | IR1; IGS (*rps12*-*trnV*-GAC) |
| *Calanthe triplicata* | 102947 | 103015 | 69 | 3 | 23.3 | IR1; IGS (*rps12*-*trnV*-GAC) |
| *Calanthe triplicata* | 102954 | 103008 | 55 | 8 | 6.8 | IR1; IGS (*rps12*-*trnV*-GAC) |
| *Calanthe triplicata* | 102959 | 103009 | 51 | 19 | 2.8 | IR1; IGS (*rps12*-*trnV*-GAC) |
| *Calanthe triplicata* | 103011 | 103054 | 44 | 14 | 3.3 | IR1; IGS (*rps12*-*trnV*-GAC) |
| *Calanthe triplicata* | 103239 | 103286 | 48 | 24 | 2.0 | IR1; IGS (*rps12*-trnV-GAC) |
| *Calanthe triplicata* | 116814 | 116864 | 51 | 25 | 2.1 | SSC; IGS (*ndhF*-*rpl32*) |
| *Calanthe triplicata* | 124450 | 124476 | 27 | 13 | 2.1 | SSC; CDS (*ndhA* intron) |
| *Calanthe triplicata* | 127028 | 127108 | 81 | 11 | 7.3 | SSC; IGS (*ndhH*-*rps15*) |
| *Calanthe triplicata* | 127722 | 127765 | 44 | 22 | 2.0 | SSC; IGS (*rps15*-*ycf1*) |
| *Calanthe triplicata* | 129172 | 129202 | 31 | 15 | 2.1 | SSC; CDS (*ycf1*) |
| *Calanthe triplicata* | 142779 | 142826 | 48 | 24 | 2.0 | IR2; CDS (*rps12* intron) |
| *Calanthe triplicata* | 143011 | 143054 | 44 | 14 | 3.3 | IR2; CDS (*rps12* intron) |
| *Calanthe triplicata* | 143050 | 143118 | 69 | 3 | 23.3 | IR2; CDS (*rps12* intron) |
| *Calanthe triplicata* | 143056 | 143106 | 51 | 19 | 2.8 | IR2; CDS (*rps12* intron) |
| *Calanthe triplicata* | 143057 | 143111 | 55 | 8 | 6.8 | IR2; CDS (*rps12* intron) |
| *Calanthe triplicata* | 143113 | 143139 | 27 | 14 | 1.9 | IR2; CDS (*rps12* intron) |
| *Calanthe triplicata* | 150873 | 150920 | 48 | 18 | 2.7 | IR2; CDS (*ycf2*) |
| *Calanthe triplicata* | 150873 | 150933 | 61 | 9 | 7.1 | IR2; CDS (y*c*f2) |
| *Calanthe triplicata* | 150873 | 150933 | 61 | 18 | 3.6 | IR2; CDS (*ycf2*) |
| *Cephalantheropsis obcordata* | 4483 | 4523 | 41 | 13 | 3.2 | LSC; IGS (*trnK*-UUU-*rps16*) |
| *Cephalantheropsis obcordata* | 6346 | 6389 | 44 | 14 | 3.2 | LSC; IGS (*rps16*-*trnQ*-UUG) |
| *Cephalantheropsis obcordata* | 6350 | 6398 | 49 | 14 | 3.7 | LSC; IGS (*rps16*-*trnQ*-UUG) |
| *Cephalantheropsis obcordata* | 6478 | 6525 | 48 | 23 | 2.0 | LSC; IGS (*rps16*-*trnQ*-UUG) |
| *Cephalantheropsis obcordata* | 6493 | 6525 | 33 | 8 | 4.3 | LSC; IGS (*rps16*-*trnQ*-UUG) |
| *Cephalantheropsis obcordata* | 8782 | 8834 | 53 | 14 | 3.6 | LSC; IGS (*trnS*-GCU-*trnG*-GCC) |
| *Cephalantheropsis obcordata* | 14120 | 14151 | 32 | 16 | 2.0 | LSC; IGS (*atpH*-*atpI*) |
| *Cephalantheropsis obcordata* | 27955 | 27988 | 34 | 17 | 2.0 | LSC; IGS (*rpoB*-*trnC*-GCA) |
| *Cephalantheropsis obcordata* | 28133 | 28169 | 37 | 15 | 2.4 | LSC; IGS (*rpoB*-*trnC*-GCA) |
| *Cephalantheropsis obcordata* | 32353 | 32377 | 25 | 12 | 2.1 | LSC; IGS (*trnE*-UUC-*trnT*-GGU) |
| *Cephalantheropsis obcordata* | 37000 | 37026 | 27 | 14 | 1.9 | LSC; IGS (*psbZ*-*trnG*-UCC) |
| *Cephalantheropsis obcordata* | 51465 | 51490 | 26 | 13 | 2.0 | LSC; IGS (*ndhC*-trnV-UAC) |
| *Cephalantheropsis obcordata* | 55481 | 55523 | 43 | 8 | 5.4 | LSC; IGS (*atpB*-*rbcL*) |
| *Cephalantheropsis obcordata* | 55484 | 55521 | 38 | 13 | 3.1 | LSC; IGS (*atpB*-*rbcL*) |
| *Cephalantheropsis obcordata* | 55562 | 55595 | 34 | 13 | 2.6 | LSC; IGS (*atpB*-*rbcL*) |
| *Cephalantheropsis obcordata* | 55575 | 55633 | 59 | 21 | 2.7 | LSC; IGS (*atpB*-r*b*cL) |
| *Cephalantheropsis obcordata* | 57786 | 57812 | 27 | 13 | 2.1 | LSC; IGS (*rbcL*-*accD*) |
| *Cephalantheropsis obcordata* | 59754 | 59797 | 44 | 23 | 1.9 | LSC; CDS (*accD*) |
| *Cephalantheropsis obcordata* | 60164 | 60237 | 74 | 26 | 2.9 | LSC; IGS (*accD*-*psaI*) |
| *Cephalantheropsis obcordata* | 60174 | 60228 | 55 | 28 | 2.0 | LSC; IGS (*accD*-*psaI*) |
| *Cephalantheropsis obcordata* | 69486 | 69515 | 30 | 15 | 2.0 | LSC; IGS (*rpl33*-*rps18*) |
| *Cephalantheropsis obcordata* | 69522 | 69565 | 44 | 21 | 2.1 | LSC; IGS (*rpl33*-*rps18*);CDS(*rps18*) |
| *Cephalantheropsis obcordata* | 73201 | 73226 | 26 | 13 | 2.0 | LSC; CDS (*clpP* intron) |
| *Cephalantheropsis obcordata* | 73864 | 73917 | 54 | 17 | 3.2 | LSC; CDS (*clpP* intron) |
| *Cephalantheropsis obcordata* | 73962 | 74023 | 62 | 27 | 2.3 | LSC; CDS (*clpP* intron) |
| *Cephalantheropsis obcordata* | 76029 | 76086 | 58 | 28 | 2.1 | LSC; IGS (*psbB*-*psbT*) |
| *Cephalantheropsis obcordata* | 82032 | 82057 | 26 | 13 | 2.0 | LSC; IGS (*rps11*-*rpl36*) |
| *Cephalantheropsis obcordata* | 84845 | 84887 | 43 | 16 | 2.7 | LSC; CDS (*rpl16* intron) |
| *Cephalantheropsis obcordata* | 84928 | 84961 | 34 | 17 | 2.0 | LSC; CDS (*rpl16* intron) |
| *Cephalantheropsis obcordata* | 85028 | 85068 | 41 | 18 | 2.3 | LSC; CDS (*rpl16* intron) |
| *Cephalantheropsis obcordata* | 94512 | 94572 | 61 | 18 | 3.6 | IR1; CDS (*ycf2*) |
| *Cephalantheropsis obcordata* | 102307 | 102347 | 41 | 10 | 4.4 | IR1; IGS (*rps12*-*trnV*-GAC) |
| *Cephalantheropsis obcordata* | 102345 | 102407 | 63 | 12 | 5.0 | IR1; IGS (*rps12*-*trnV*-GAC) |
| *Cephalantheropsis obcordata* | 115608 | 115650 | 43 | 20 | 2.2 | SSC; IGS (*ndhF*-*rpl32*) |
| *Cephalantheropsis obcordata* | 115647 | 115675 | 29 | 14 | 2.1 | SSC; IGS (*ndhF*-*rpl32*) |
| *Cephalantheropsis obcordata* | 115685 | 115710 | 26 | 13 | 2.0 | SSC; IGS (*ndhF*-*rpl32*) |
| *Cephalantheropsis obcordata* | 116103 | 116141 | 39 | 17 | 2.3 | SSC; IGS (*ndhF*-*rpl32*) |
| *Cephalantheropsis obcordata* | 116106 | 116144 | 39 | 13 | 3.1 | SSC; IGS (*ndhF*-*rpl32*) |
| *Cephalantheropsis obcordata* | 116633 | 116665 | 33 | 12 | 2.6 | SSC; IGS (*rpl32*-*trnL*-UAG) |
| *Cephalantheropsis obcordata* | 118548 | 118584 | 37 | 19 | 1.9 | SSC; IGS (*ccsA*-*ndhD*);CDS(*ndhD*) |
| *Cephalantheropsis obcordata* | 120692 | 120731 | 40 | 14 | 2.9 | SSC; IGS (*psaC*-*ndhE*) |
| *Cephalantheropsis obcordata* | 120877 | 120934 | 58 | 28 | 2.1 | SSC; IGS (*psaC*-*ndhE*) |
| *Cephalantheropsis obcordata* | 142164 | 142226 | 63 | 12 | 5.0 | IR2; CDS (*rps12* intron) |
| *Cephalantheropsis obcordata* | 142224 | 142264 | 41 | 10 | 4.4 | IR2; CDS (*rps12* intron) |
| *Cephalantheropsis obcordata* | 149999 | 150059 | 61 | 18 | 3.6 | IR2; CDS (*ycf2*) |
| *Cephalantheropsis obcordata* | 150003 | 150047 | 45 | 9 | 5.0 | IR2; CDS (*ycf2*) |
| *Phaius tankervilliae* | 55 | 90 | 36 | 10 | 3.5 | LSC; IGS (*psbA*-*rps19*) |
| *Phaius tankervilliae* | 3528 | 3562 | 35 | 16 | 2.2 | LSC; tRNA (*trnK*-UUU) |
| *Phaius tankervilliae* | 6370 | 6405 | 36 | 15 | 2.3 | LSC; IGS (*rps16*-*trnQ*-UUG) |
| *Phaius tankervilliae* | 6456 | 6480 | 25 | 12 | 2.1 | LSC; IGS (*rps16*-*trnQ*-UUG) |
| *Phaius tankervilliae* | 8639 | 8664 | 26 | 13 | 2.0 | LSC; IGS (*trnS*-GCU-*trnG*-GCC) |
| *Phaius tankervilliae* | 9072 | 9103 | 32 | 15 | 2.1 | LSC; IGS (*trnS*-GCU-*trnG*-GCC) |
| *Phaius tankervilliae* | 27910 | 27943 | 34 | 17 | 2.0 | LSC; IGS (*rpoB*-*trnC*-GCA) |
| *Phaius tankervilliae* | 31795 | 31824 | 30 | 15 | 2.0 | LSC; IGS (*trnY*-GUA-*trnE*-UUC) |
| *Phaius tankervilliae* | 32169 | 32256 | 88 | 17 | 5.4 | LSC; IGS (*trnE*-UUC-*trnT*-GGU) |
| *Phaius tankervilliae* | 32174 | 32256 | 83 | 15 | 5.3 | LSC; IGS (*trnE*-UUC-*trnT*-GGU) |
| *Phaius tankervilliae* | 32180 | 32262 | 83 | 17 | 5.1 | LSC; IGS (*trnE*-UUC-*trnT*-GGU) |
| *Phaius tankervilliae* | 51416 | 51444 | 29 | 13 | 2.3 | LSC; IGS (*ndhC*-*trnV*-UAC) |
| *Phaius tankervilliae* | 55655 | 55693 | 39 | 13 | 3.2 | LSC; IGS (*atpB*-*rbcL*) |
| *Phaius tankervilliae* | 55656 | 55694 | 39 | 7 | 6.1 | LSC; IGS (*atpB*-*rbcL*) |
| *Phaius tankervilliae* | 55657 | 55693 | 37 | 11 | 3.2 | LSC; IGS (*atpB*-*rbcL*) |
| *Phaius tankervilliae* | 55860 | 55896 | 37 | 18 | 2.1 | LSC; IGS (*atpB*-*rbcL*) |
| *Phaius tankervilliae* | 57869 | 57904 | 36 | 12 | 3.0 | LSC; IGS (*rbcL*-*accD*) |
| *Phaius tankervilliae* | 60354 | 60399 | 46 | 23 | 2.0 | LSC; IGS (*accD*-*psaI*) |
| *Phaius tankervilliae* | 60355 | 60434 | 80 | 26 | 3.2 | LSC; IGS (*accD*-*psaI*) |
| *Phaius tankervilliae* | 69653 | 69682 | 30 | 15 | 2.0 | LSC; IGS (*rpl33*-*rps18*) |
| *Phaius tankervilliae* | 69689 | 69732 | 44 | 21 | 2.1 | LSC; CDS (*rps18*) |
| *Phaius tankervilliae* | 73456 | 73504 | 49 | 13 | 3.8 | LSC; CDS (*clpP* intron) |
| *Phaius tankervilliae* | 74017 | 74049 | 33 | 15 | 2.2 | LSC; IGS (*clpP*-*psbB*) |
| *Phaius tankervilliae* | 74089 | 74117 | 29 | 15 | 1.9 | LSC; IGS (*clpP*-*psbB*) |
| *Phaius tankervilliae* | 77359 | 77404 | 46 | 22 | 2.1 | LSC; IGS (*psbH*-*petB*) |
| *Phaius tankervilliae* | 77366 | 77404 | 39 | 10 | 3.7 | LSC; IGS (*psbH*-*petB*) |
| *Phaius tankervilliae* | 82038 | 82086 | 49 | 24 | 2.0 | LSC; CDS (*rps11*) |
| *Phaius tankervilliae* | 82228 | 82253 | 26 | 13 | 2.0 | LSC; IGS (*rps11*-*rpl36*) |
| *Phaius tankervilliae* | 85017 | 85059 | 43 | 16 | 2.7 | LSC; CDS (*rpl16* intron) |
| *Phaius tankervilliae* | 87033 | 87057 | 25 | 12 | 2.1 | LSC; IGS (*rpl22*-*rps19*) |
| *Phaius tankervilliae* | 94696 | 94756 | 61 | 18 | 3.6 | IR1; CDS (*ycf2*) |
| *Phaius tankervilliae* | 102356 | 102423 | 68 | 31 | 2.2 | IR1; IGS (*rps12*-*trnV*-GAC) |
| *Phaius tankervilliae* | 102406 | 102483 | 78 | 26 | 3.1 | IR1; IGS (*rps12*-*trnV*-GAC) |
| *Phaius tankervilliae* | 102424 | 102467 | 44 | 15 | 2.9 | IR1; IGS (*rps12*-*trnV*-GAC) |
| *Phaius tankervilliae* | 102437 | 102527 | 91 | 20 | 4.4 | IR1; IGS (*rps12*-*trnV*-GAC) |
| *Phaius tankervilliae* | 102445 | 102532 | 88 | 29 | 2.8 | IR1; IGS (*rps12*-*trnV*-GAC) |
| *Phaius tankervilliae* | 115819 | 115856 | 38 | 20 | 2.0 | SSC; IGS (*ndhF*-*rpl32*) |
| *Phaius tankervilliae* | 115955 | 116003 | 49 | 17 | 2.8 | SSC; IGS (*ndhF*-*rpl32*) |
| *Phaius tankervilliae* | 115960 | 116011 | 52 | 18 | 2.9 | SSC; IGS (*ndhF*-*rpl32*) |
| *Phaius tankervilliae* | 116167 | 116192 | 26 | 13 | 2.0 | SSC; IGS (*ndhF*-*rpl32*) |
| *Phaius tankervilliae* | 116313 | 116352 | 40 | 17 | 2.4 | SSC; IGS (*ndhF*-*rpl32*) |
| *Phaius tankervilliae* | 118749 | 118785 | 37 | 19 | 1.9 | SSC; IGS (*ccsA*-*ndhD*);CDS(*ndhD*) |
| *Phaius tankervilliae* | 120895 | 120919 | 25 | 10 | 2.5 | SSC; IGS (*psaC*-*ndhE*) |
| *Phaius tankervilliae* | 142336 | 142423 | 88 | 29 | 2.8 | IR2; IGS (*rps12* intron) |
| *Phaius tankervilliae* | 142384 | 142445 | 62 | 20 | 3.1 | IR2; IGS (*rps12* intron) |
| *Phaius tankervilliae* | 142385 | 142462 | 78 | 26 | 3.1 | IR2; IGS (*rps12* intron) |
| *Phaius tankervilliae* | 142401 | 142444 | 44 | 16 | 2.9 | IR2; IGS (*rps12* intron) |
| *Phaius tankervilliae* | 142445 | 142512 | 68 | 31 | 2.2 | IR2; IGS (*rps12* intron) |
| *Phaius tankervilliae* | 150112 | 150172 | 61 | 18 | 3.6 | IR2; CDS (*ycf2*) |
| *Phaius tankervilliae* | 150116 | 150160 | 45 | 9 | 5.0 | IR2; CDS (*ycf2*) |
| *Phaius tankervilliae* | 157811 | 157835 | 25 | 12 | 2.1 | IR2; IGS (*rps19*-*psbA*) |
