# Supplementary material for: Plastome structure and adaptive evolution of Calanthe s.l. species
Source: PeerJ. 2020 Oct 13;8:e10051. doi: 10.7717/peerj.10051 (PMC7566753; doi:10.7717/peerj.10051)
Supplement: Supplemental Information 6 [file peerj-08-10051-s006.docx]

### **Table S6** Simple sequence repeats distribution in seven *Calanthe* s.l. plastomes.

| **Species** | **No** | **Start** | **End** | **SSR type** | **Region** |
| --- | --- | --- | --- | --- | --- |
| *Calanthe davidii* | 1 | 1523 | 1533 | (T)11 | LSC; IGS (*psbA*-*trnK*-UUU) |
| *Calanthe davidii* | 2 | 3597 | 3608 | (TTC)4 | LSC; tRNA (*trnK*-UUU) |
| *Calanthe davidii* | 3 | 5319 | 5330 | (TCTA)3 | LSC; CDS (*rps16*) |
| *Calanthe davidii* | 4 | 5339 | 5354 | (ATAG)4 | LSC; CDS (*rps16*) |
| *Calanthe davidii* | 5 | 5527 | 5536 | (TA)5 | LSC; CDS (*rps16*) |
| *Calanthe davidii* | 6 | 6716 | 6727 | (TA)6 | LSC; IGS (*rps16*-*trnQ*-UUG) |
| *Calanthe davidii* | 7 | 7191 | 7200 | (A)10 | LSC; IGS (*rps16*-*trnQ*-UUG) |
| *Calanthe davidii* | 8 | 8416 | 8430 | (ATCTT)3 | LSC; IGS (*psbK*-*psbI*) |
| *Calanthe davidii* | 9 | 9713 | 9722 | (A)10 | LSC; IGS (*trnS*-GCU-*trnG*-GCC) |
| *Calanthe davidii* | 10 | 12073 | 12084 | (GTCT)3 | LSC; CDS (*atpA*) |
| *Calanthe davidii* | 11 | 16139 | 16148 | (A)10 | LSC; IGS (*atpI*-*rps2*) |
| *Calanthe davidii* | 12 | 17040 | 17051 | (T)12 | LSC; IGS (*rps2*-*rpoC2*) |
| *Calanthe davidii* | 13 | 19147 | 19156 | (T)10 | LSC; CDS (*rpoC2*) |
| *Calanthe davidii* | 14 | 19253 | 19263 | (T)11 | LSC; CDS (*rpoC2*) |
| *Calanthe davidii* | 15 | 20632 | 20641 | (AT)5 | LSC; CDS (*rpoC2*) |
| *Calanthe davidii* | 16 | 28981 | 28992 | (AT)6 | LSC; IGS (*rpoB*-*trnC*-GCA) |
| *Calanthe davidii* | 17 | 32949 | 32960 | (AT)6 | LSC; IGS (*trnE*-UUC-*trnT*-GGU) |
| *Calanthe davidii* | 18 | 32965 | 32978 | (AT)7 | LSC; IGS (*trnE*-UUC-*trnT*-GGU) |
| *Calanthe davidii* | 19 | 33423 | 33432 | (AT)5 | LSC; IGS (*trnE*-UUC-*trnT*-GGU) |
| *Calanthe davidii* | 20 | 33469 | 33479 | (A)11 | LSC; IGS (*trnE*-UUC-*trnT*-GGU) |
| *Calanthe davidii* | 21 | 33620 | 33629 | (A)10 | LSC; IGS (*trnT*-GGU-*psbD*) |
| *Calanthe davidii* | 22 | 43517 | 43527 | (T)11 | LSC; IGS (*psaA*-*ycf3*) |
| *Calanthe davidii* | 23 | 47933 | 47943 | (A)11 | LSC; IGS (*trnT*-UGU-*trnL*-UAA) |
| *Calanthe davidii* | 24 | 48950 | 48962 | (T)13 | LSC; IGS (*trnL*-UAA-*trnF*-GAA) |
| *Calanthe davidii* | 25 | 51023 | 51032 | (T)10 | LSC; CDS (*ndhK*) |
| *Calanthe davidii* | 26 | 52757 | 52768 | (CTA)4 | LSC; IGS (*ndhC*-*trnV*-UAC) |
| *Calanthe davidii* | 27 | 60507 | 60516 | (A)10 | LSC; CDS (*accD*) |
| *Calanthe davidii* | 28 | 62569 | 62580 | (A)12 | LSC; IGS (*ycf4*-*cemA*) |
| *Calanthe davidii* | 29 | 63875 | 63886 | (AATG)3 | LSC; CDS (*cemA*) |
| *Calanthe davidii* | 30 | 65619 | 65628 | (TA)5 | LSC; IGS (*petA*-*psbJ*) |
| *Calanthe davidii* | 31 | 66891 | 66900 | (A)10 | LSC; IGS (*psbE*-*petL*) |
| *Calanthe davidii* | 32 | 67863 | 67872 | (T)10 | LSC; IGS (*psbE*-*petL*) |
| *Calanthe davidii* | 33 | 69466 | 69475 | (A)10 | LSC; IGS (*psaJ*-*rpl33*) |
| *Calanthe davidii* | 34 | 69646 | 69657 | (ATTA)3 | LSC; IGS (*psaJ*-*rpl33*) |
| *Calanthe davidii* | 35 | 70139 | 70148 | (TG)5 | LSC; IGS (*rpl33*-*rps18*) |
| *Calanthe davidii* | 36 | 73548 | 73557 | (A)10 | LSC; CDS (*clpP* intron) |
| *Calanthe davidii* | 37 | 73585 | 73599 | (T)15 | LSC; CDS (*clpP* intron) |
| *Calanthe davidii* | 38 | 73844 | 73853 | (T)10 | LSC; CDS (*clpP* intron) |
| *Calanthe davidii* | 39 | 74936 | 74951 | (TA)8 | LSC; IGS (*clpP*-*psbB*) |
| *Calanthe davidii* | 40 | 77457 | 77470 | (TA)7 | LSC; IGS (*psbB*-*psbT*) |
| *Calanthe davidii* | 41 | 80233 | 80243 | (T)11 | LSC; CDS (*petD* intron) |
| *Calanthe davidii* | 42 | 81440 | 81451 | (TTTA)3 | LSC; IGS (*petD*-*rpoA*) |
| *Calanthe davidii* | 43 | 83327 | 83341 | (T)15 | LSC; IGS (*rpl36*-*infA*) |
| *Calanthe davidii* | 44 | 84234 | 84243 | (T)10 | LSC; IGS (*rps8*-*rpl14*) |
| *Calanthe davidii* | 45 | 86494 | 86503 | (T)10 | LSC; IGS (*rpl16* intron) |
| *Calanthe davidii* | 46 | 93769 | 93778 | (GA)5 | IR1; CDS (*ycf2*) |
| *Calanthe davidii* | 47 | 116796 | 116805 | (T)10 | SSC; IGS (*ndhF*-*rpl32*) |
| *Calanthe davidii* | 48 | 116930 | 116943 | (TA)7 | SSC; IGS (*ndhF*-*rpl32*) |
| *Calanthe davidii* | 49 | 116965 | 116974 | (TA)5 | SSC; IGS (*ndhF*-*rpl32*) |
| *Calanthe davidii* | 50 | 122075 | 122086 | (AAT)4 | SSC; IGS (*psaC*-*ndhE*) |
| *Calanthe davidii* | 51 | 122237 | 122248 | (TTGA)3 | SSC; CDS (*ndhE*) |
| *Calanthe davidii* | 52 | 122680 | 122690 | (A)11 | SSC; IGS (*ndhE*-*nad6*) |
| *Calanthe davidii* | 53 | 125592 | 125601 | (A)10 | SSC; CDS (*ndhA* intron) |
| *Calanthe davidii* | 54 | 129065 | 129074 | (T)10 | SSC; CDS (*ycf1*) |
| *Calanthe davidii* | 55 | 130997 | 131006 | (A)10 | SSC; CDS (*ycf1*) |
| *Calanthe davidii* | 56 | 132311 | 132324 | (T)14 | SSC; CDS (*ycf1*) |
| *Calanthe davidii* | 57 | 132432 | 132441 | (T)10 | SSC; CDS (*ycf1*) |
| *Calanthe davidii* | 58 | 132565 | 132575 | (T)11 | SSC; CDS (*ycf1*) |
| *Calanthe davidii* | 59 | 132593 | 132604 | (A)12 | SSC; CDS (*ycf1*) |
| *Calanthe davidii* | 60 | 153094 | 153103 | (TC)5 | IR2; CDS (*ycf2*) |
| *Calanthe delavayi* | 1 | 1676 | 1685 | (T)10 | LSC; tRNA (*trnK*-UUU intron) |
| *Calanthe delavayi* | 2 | 5120 | 5131 | (TCTA)3 | LSC;CDS (*rps16* intron) |
| *Calanthe delavayi* | 3 | 5140 | 5155 | (ATAG)4 | LSC;CDS (*rps16* intron) |
| *Calanthe delavayi* | 4 | 6902 | 6911 | (A)10 | LSC; IGS (*rps16*-*trnQ*-UUG) |
| *Calanthe delavayi* | 5 | 8047 | 8056 | (T)10 | LSC; IGS (*psbK*-*psbI*) |
| *Calanthe delavayi* | 6 | 10552 | 10561 | (A)10 | LSC; IGS (*trnR*-*atpA*) |
| *Calanthe delavayi* | 7 | 11677 | 11688 | (GTCT)3 | LSC; CDS (*atpA*) |
| *Calanthe delavayi* | 8 | 13829 | 13838 | (T)10 | LSC; IGS (*atpF*-*atpH*) |
| *Calanthe delavayi* | 9 | 16590 | 16599 | (T)10 | LSC; CDS (*rps2*) |
| *Calanthe delavayi* | 10 | 18689 | 18698 | (T)10 | LSC; CDS (*rpoC2*) |
| *Calanthe delavayi* | 11 | 18795 | 18805 | (T)11 | LSC; CDS (*rpoC2*) |
| *Calanthe delavayi* | 12 | 20174 | 20183 | (AT)5 | LSC; CDS (*rpoC2*) |
| *Calanthe delavayi* | 13 | 28456 | 28467 | (TTTA)3 | LSC; CDS (*rpoC2*) |
| *Calanthe delavayi* | 14 | 29587 | 29598 | (TAT)4 | LSC; IGS (*petN*-*psbM*) |
| *Calanthe delavayi* | 15 | 30499 | 30510 | (AGAA)3 | LSC; IGS (*psbM*-trnD-GUC) |
| *Calanthe delavayi* | 16 | 32625 | 32634 | (T)10 | LSC;IGS (*trnE*-UUC-*trnT*-GGU) |
| *Calanthe delavayi* | 17 | 42537 | 42546 | (T)10 | LSC; IGS (*psaA*-*ycf3*) |
| *Calanthe delavayi* | 18 | 46964 | 46973 | (A)10 | LSC; IGS (*trnT*-UGU-*trnL*-UAA) |
| *Calanthe delavayi* | 19 | 47960 | 47969 | (T)10 | LSC; IGS (*trnL*-UAA-*trnF*-GAA) |
| *Calanthe delavayi* | 20 | 48108 | 48121 | (AT)7 | LSC; IGS (*trnL*-UAA-*trnF*-GAA) |
| *Calanthe delavayi* | 21 | 49542 | 49553 | (CTA)4 | LSC; IGS (*ndhJ*-*trnV*-UAC) |
| *Calanthe delavayi* | 22 | 52782 | 52791 | (T)10 | LSC; IGS (*atpB*-*rbcL*) |
| *Calanthe delavayi* | 23 | 53014 | 53031 | (TA)9 | LSC; IGS (*atpB*-*rbcL*) |
| *Calanthe delavayi* | 24 | 59134 | 59144 | (T)11 | LSC; IGS (*ycf4*-*cemA*) |
| *Calanthe delavayi* | 25 | 60050 | 60061 | (AATG)3 | LSC; CDS (*cemA*) |
| *Calanthe delavayi* | 26 | 61296 | 61307 | (T)12 | LSC; IGS (*petA*-*psbJ*) |
| *Calanthe delavayi* | 27 | 61792 | 61817 | (AT)13 | LSC; IGS (*petA*-*psbJ*) |
| *Calanthe delavayi* | 28 | 62738 | 62747 | (A)10 | LSC; CDS (*psbF*) |
| *Calanthe delavayi* | 29 | 63112 | 63122 | (A)11 | LSC; IGS (*psbE*-*petL*) |
| *Calanthe delavayi* | 30 | 65686 | 65697 | (ATTA)3 | LSC; IGS (*psbJ*-*rpl33*) |
| *Calanthe delavayi* | 31 | 66191 | 66200 | (TG)5 | LSC; IGS (*rpl33*-*rps18*) |
| *Calanthe delavayi* | 32 | 69412 | 69423 | (ATAA)3 | LSC; CDS (*clpP*) |
| *Calanthe delavayi* | 33 | 69648 | 69658 | (T)11 | LSC; CDS (*clpP* intron) |
| *Calanthe delavayi* | 34 | 69903 | 69912 | (T)10 | LSC; CDS (*clpP* intron) |
| *Calanthe delavayi* | 35 | 75927 | 75940 | (T)14 | LSC; CDS (*petD* intron) |
| *Calanthe delavayi* | 36 | 77159 | 77168 | (A)10 | LSC; IGS (*petD*-*rpoA*) |
| *Calanthe delavayi* | 37 | 80073 | 80088 | (TA)8 | LSC; IGS (*rps8*-*rpl14*) |
| *Calanthe delavayi* | 38 | 89472 | 89481 | (GA)5 | IR1; CDS(*ycf2*) |
| *Calanthe delavayi* | 39 | 98863 | 98882 | (TATTA)4 | IR1; IGS (*rps12*-*trnV*-GAC) |
| *Calanthe delavayi* | 40 | 99499 | 99509 | (T)11 | IR1; IGS (*rps12*-*trnV*-GAC) |
| *Calanthe delavayi* | 41 | 108844 | 108853 | (TA)5 | SSC; IGS (*trnN*-GUU-*rpl32*) |
| *Calanthe delavayi* | 42 | 111570 | 111580 | (T)11 | SSC; IGS (*ccsA*-*ndhD*) |
| *Calanthe delavayi* | 43 | 113971 | 113982 | (AAT)4 | SSC; IGS (*psaC*-*ndhE*) |
| *Calanthe delavayi* | 44 | 114130 | 114141 | (TTGA)3 | SSC; CDS (*ndhE*) |
| *Calanthe delavayi* | 45 | 114573 | 114582 | (A)10 | SSC; IGS (*ndhE*-*nad6*) |
| *Calanthe delavayi* | 46 | 114807 | 114816 | (A)10 | SSC; IGS (*nad6*-*ndhI*) |
| *Calanthe delavayi* | 47 | 116345 | 116362 | (CATATG)3 | SSC; CDS (*ndhA* intron) |
| *Calanthe delavayi* | 48 | 116937 | 116946 | (A)10 | SSC; CDS (*ndhA* intron) |
| *Calanthe delavayi* | 49 | 119506 | 119515 | (T)10 | SSC; CDS (*ycf1*) |
| *Calanthe delavayi* | 50 | 122256 | 122265 | (A)10 | SSC; CDS (*ycf1*) |
| *Calanthe delavayi* | 51 | 123685 | 123694 | (T)10 | SSC; CDS (*ycf1*) |
| *Calanthe delavayi* | 52 | 123846 | 123857 | (A)12 | SSC; CDS (*ycf1*) |
| *Calanthe delavayi* | 53 | 134084 | 134094 | (A)11 | IR2; CDS (*rps12* intron) |
| *Calanthe delavayi* | 54 | 134710 | 134729 | (ATAAT)4 | IR2; CDS (*rps12* intron) |
| *Calanthe delavayi* | 55 | 144112 | 144121 | (TC)5 | IR2; CDS (*ycf2*) |
| *Styloglossum lyroglossa* | 1 | 1473 | 1483 | (A)11 | LSC; IGS(*psbA*--*trnK*-UUU) |
| *Styloglossum lyroglossa* | 2 | 1770 | 1798 | (T)11(ATGAAA)3 | LSC; tRNA (*trnK*-UUU intron) |
| *Styloglossum lyroglossa* | 3 | 5251 | 5262 | (TCTA)3 | LSC;CDS (*rps16* intron) |
| *Styloglossum lyroglossa* | 4 | 6941 | 6951 | (A)11 | LSC; IGS (*rps16*-*trnQ*-UUG) |
| *Styloglossum lyroglossa* | 5 | 8144 | 8158 | (ATCTT)3 | LSC; IGS (*rps16*-*trnQ*-UUG) |
| *Styloglossum lyroglossa* | 6 | 9512 | 9521 | (T)10 | LSC; IGS (*psbK*-*psbI*) |
| *Styloglossum lyroglossa* | 7 | 10665 | 10674 | (A)10 | LSC; IGS (*trnR*-UCU-*atpA*) |
| *Styloglossum lyroglossa* | 8 | 11800 | 11811 | (GTCT)3 | LSC; CDS (*atpA*) |
| *Styloglossum lyroglossa* | 9 | 13102 | 13111 | (A)10 | LSC; CDS (*atpF* intron) |
| *Styloglossum lyroglossa* | 10 | 16739 | 16748 | (T)10 | LSC; IGS (*rps2*-*rpoC2*) |
| *Styloglossum lyroglossa* | 11 | 18851 | 18860 | (T)10 | LSC; CDS (*rpoC2*) |
| *Styloglossum lyroglossa* | 12 | 18957 | 18967 | (T)11 | LSC; CDS (*rpoC2*) |
| *Styloglossum lyroglossa* | 13 | 20336 | 20345 | (AT)5 | LSC; CDS (*rpoC2*) |
| *Styloglossum lyroglossa* | 14 | 28513 | 28523 | (A)11 | LSC; IGS (*rpoB*-*trnC*-GCA) |
| *Styloglossum lyroglossa* | 15 | 28658 | 28669 | (ATA)4 | LSC; IGS (*rpoB*-*trnC*-GCA) |
| *Styloglossum lyroglossa* | 16 | 28675 | 28686 | (TA)6 | LSC; IGS (*rpoB*-*trnC*-GCA) |
| *Styloglossum lyroglossa* | 17 | 29419 | 29428 | (T)10 | LSC; IGS (*trnC*-GCA-*petN*) |
| *Styloglossum lyroglossa* | 18 | 30743 | 30754 | (AGAA)3 | LSC; IGS (*petN*-*psbM*) |
| *Styloglossum lyroglossa* | 19 | 32583 | 32592 | (A)10 | LSC; IGS (*trnT*-GGU-*psbD*) |
| *Styloglossum lyroglossa* | 20 | 42481 | 42490 | (T)10 | LSC; IGS (*psaA*-*ycf3*) |
| *Styloglossum lyroglossa* | 21 | 46795 | 46806 | (AT)6 | LSC; IGS (*trnT*-UGU-*trnL*-UAA) |
| *Styloglossum lyroglossa* | 22 | 46851 | 46861 | (A)11 | LSC; IGS (*trnT*-UGU-*trnL*-UAA) |
| *Styloglossum lyroglossa* | 23 | 47986 | 47997 | (AT)6 | LSC; IGS (*trnL*-UAA-*trnF*-GAA) |
| *Styloglossum lyroglossa* | 24 | 50135 | 50144 | (T)10 | LSC;CDS (*ndhK*) |
| *Styloglossum lyroglossa* | 25 | 51418 | 51427 | (A)10 | LSC; IGS (*ndhC*-*trnV*-UAC) |
| *Styloglossum lyroglossa* | 26 | 60012 | 60023 | (ATA)4 | LSC; IGS (*accD*-*psaI*) |
| *Styloglossum lyroglossa* | 27 | 61943 | 61954 | (AATG)3 | LSC; CDS (*cemA*) |
| *Styloglossum lyroglossa* | 28 | 63196 | 63208 | (T)13 | LSC; IGS (*petA*-*psbJ*) |
| *Styloglossum lyroglossa* | 29 | 63543 | 63562 | (TA)10 | LSC; IGS (*petA*-*psbJ*) |
| *Styloglossum lyroglossa* | 30 | 65619 | 65633 | (ACAAA)3 | LSC; IGS (*psbE*-*petL*) |
| *Styloglossum lyroglossa* | 31 | 67380 | 67390 | (A)11 | LSC; IGS (*psaJ*-*rpl33*) |
| *Styloglossum lyroglossa* | 32 | 67561 | 67572 | (ATTA)3 | LSC; IGS (*psaJ*-*rpl33*) |
| *Styloglossum lyroglossa* | 33 | 67762 | 67771 | (T)10 | LSC; IGS (*psaJ*-*rpl33*) |
| *Styloglossum lyroglossa* | 34 | 68061 | 68070 | (TG)5 | LSC; IGS (*psaJ*-*rpl33*) |
| *Styloglossum lyroglossa* | 35 | 68817 | 68826 | (T)10 | LSC; IGS (*psaJ*-*rpl33*) |
| *Styloglossum lyroglossa* | 36 | 71506 | 71515 | (T)10 | LSC; CDS (*clpP* intron) |
| *Styloglossum lyroglossa* | 37 | 71760 | 71771 | (T)12 | LSC; CDS (*clpP* intron) |
| *Styloglossum lyroglossa* | 38 | 72566 | 72575 | (TA)5 | LSC; IGS (*clpP*-*psbB*) |
| *Styloglossum lyroglossa* | 39 | 72657 | 72666 | (TA)5 | LSC; IGS (*clpP*-*psbB*) |
| *Styloglossum lyroglossa* | 40 | 74733 | 74743 | (T)11 | LSC; IGS (*psbB*-*psbT*) |
| *Styloglossum lyroglossa* | 41 | 74820 | 74829 | (TA)5 | LSC; IGS (*psbB*-*psbT*) |
| *Styloglossum lyroglossa* | 42 | 76549 | 76561 | (A)13 | LSC; CDS (*petB* intron) |
| *Styloglossum lyroglossa* | 43 | 76563 | 76572 | (A)10 | LSC; CDS (*petB* intron) |
| *Styloglossum lyroglossa* | 44 | 77975 | 77986 | (TAAT)3 | LSC; CDS (*petD* intron) |
| *Styloglossum lyroglossa* | 45 | 81046 | 81055 | (T)10 | LSC; IGS (*rpl36*-*infA*) |
| *Styloglossum lyroglossa* | 46 | 81949 | 81958 | (T)10 | LSC; IGS (*rps8*-*rpl14*) |
| *Styloglossum lyroglossa* | 47 | 82602 | 82613 | (CTT)4 | LSC; IGS (*rpl14*-*rpl16*) |
| *Styloglossum lyroglossa* | 48 | 84208 | 84217 | (T)10 | LSC; CDS (*rpl16* intron) |
| *Styloglossum lyroglossa* | 49 | 84437 | 84448 | (ATT)4 | LSC; IGS (*rpl16*-*rps3*) |
| *Styloglossum lyroglossa* | 50 | 91466 | 91475 | (GA)5 | IR1;CDS (*ycf2*) |
| *Styloglossum lyroglossa* | 51 | 94197 | 94214 | (TAGAAG)3 | IR1;CDS (*ycf2*) |
| *Styloglossum lyroglossa* | 52 | 101085 | 101096 | (TAT)4 | IR1; IGS (*rps12*-*trnV*-GAC) |
| *Styloglossum lyroglossa* | 53 | 119091 | 119100 | (TA)5 | SSC; IGS (*psaC*-*ndhE*) |
| *Styloglossum lyroglossa* | 54 | 119253 | 119264 | (AAT)4 | SSC; IGS (*psaC*-*ndhE*) |
| *Styloglossum lyroglossa* | 55 | 119415 | 119426 | (TTGA)3 | SSC; CDS (*ndhE*) |
| *Styloglossum lyroglossa* | 56 | 122294 | 122304 | (A)11 | SSC; CDS (*ndhA* intron) |
| *Styloglossum lyroglossa* | 57 | 128082 | 128092 | (T)11 | SSC; CDS (*ycf1*) |
| *Styloglossum lyroglossa* | 58 | 128205 | 128214 | (A)10 | SSC; CDS (*ycf1*) |
| *Styloglossum lyroglossa* | 59 | 128285 | 128294 | (T)10 | SSC; CDS (*ycf1*) |
| *Styloglossum lyroglossa* | 60 | 128789 | 128798 | (A)10 | SSC; CDS (*ycf1*) |
| *Styloglossum lyroglossa* | 61 | 129498 | 129511 | (T)14 | SSC; CDS (*ycf1*) |
| *Styloglossum lyroglossa* | 62 | 129619 | 129628 | (T)10 | SSC; CDS (*ycf1*) |
| *Styloglossum lyroglossa* | 63 | 129780 | 129791 | (A)12 | SSC; CDS (*ycf1*) |
| *Styloglossum lyroglossa* | 64 | 140467 | 140478 | (ATA)4 | IR2;CDS (*rps12* intron) |
| *Styloglossum lyroglossa* | 65 | 147349 | 147366 | (CTTCTA)3 | IR2; CDS (*ycf2*) |
| *Styloglossum lyroglossa* | 66 | 150088 | 150097 | (TC)5 | IR2; CDS (*ycf2*) |
| *Preptanthe rubens* | 1 | 3562 | 3573 | (ATA)4 | LSC; tRNA (*trnK*-UUU intron) |
| *Preptanthe rubens* | 2 | 4037 | 4046 | (A)10 | LSC; tRNA (*trnK*-UUU intron) |
| *Preptanthe rubens* | 3 | 5337 | 5346 | (TA)5 | LSC;CDS (*rps16* intron) |
| *Preptanthe rubens* | 4 | 5368 | 5379 | (ATAG)3 | LSC;CDS (*rps16* intron) |
| *Preptanthe rubens* | 5 | 6472 | 6481 | (T)10 | LSC; IGS (*rps16*-*trnQ*-UUG) |
| *Preptanthe rubens* | 6 | 7139 | 7153 | (A)15 | LSC; IGS (*rps16*-*trnQ*-UUG) |
| *Preptanthe rubens* | 7 | 8363 | 8377 | (ATCTT)3 | LSC; IGS (*psbK*-*psbI*) |
| *Preptanthe rubens* | 8 | 10246 | 10255 | (A)10 | LSC; tRNA (*trnG*-GCC intron) |
| *Preptanthe rubens* | 9 | 11850 | 11861 | (GTCT)3 | LSC;CDS (*atpA*) |
| *Preptanthe rubens* | 10 | 13151 | 13161 | (A)11 | LSC;CDS (*atpF* intron) |
| *Preptanthe rubens* | 11 | 14077 | 14089 | (T)13 | LSC; IGS (*atpF*-*atpH*) |
| *Preptanthe rubens* | 12 | 16849 | 16859 | (T)11 | LSC; IGS (*rps2*-*rpoC2*) |
| *Preptanthe rubens* | 13 | 18955 | 18964 | (T)10 | LSC; CDS (*rpoC2*) |
| *Preptanthe rubens* | 14 | 19061 | 19071 | (T)11 | LSC; CDS (*rpoC2*) |
| *Preptanthe rubens* | 15 | 20440 | 20449 | (AT)5 | LSC; CDS (*rpoC2*) |
| *Preptanthe rubens* | 16 | 30432 | 30443 | (AGAA)3 | LSC; IGS (*psbM*-*trnD*-GUC) |
| *Preptanthe rubens* | 17 | 30640 | 30649 | (A)10 | LSC; IGS (*psbM*-*trnD*-GUC) |
| *Preptanthe rubens* | 18 | 31058 | 31068 | (A)11 | LSC; IGS (*psbM*-*trnD*-GUC) |
| *Preptanthe rubens* | 19 | 32185 | 32198 | (AT)7 | LSC; IGS (*trnE*-UUC-*trnT*-GGU) |
| *Preptanthe rubens* | 20 | 32225 | 32234 | (AT)5 | LSC; IGS (*trnE*-UUC-*trnT*-GGU) |
| *Preptanthe rubens* | 21 | 32610 | 32619 | (T)10 | LSC; IGS (*trnE*-UUC-*trnT*-GGU) |
| *Preptanthe rubens* | 22 | 32743 | 32752 | (A)10 | LSC; IGS (*trnE*-UUC-*trnT*-GGU) |
| *Preptanthe rubens* | 23 | 36524 | 36533 | (A)10 | LSC; IGS (*trnS*-UGA-*psbZ*) |
| *Preptanthe rubens* | 24 | 44913 | 44922 | (A)10 | LSC; IGS (*trnS*-GGA-*rps4*) |
| *Preptanthe rubens* | 25 | 47886 | 47895 | (TA)5 | LSC; tRNA (*trnL*-UAA intron) |
| *Preptanthe rubens* | 26 | 48608 | 48617 | (AT)5 | LSC; IGS (*trnL*-UAA-*trnF*-GAA) |
| *Preptanthe rubens* | 27 | 50754 | 50763 | (T)10 | LSC; CDS (*ndhK*) |
| *Preptanthe rubens* | 28 | 55788 | 55799 | (T)12 | LSC; IGS (*atpB*-*rbcL*) |
| *Preptanthe rubens* | 29 | 56014 | 56027 | (AT)7 | LSC; IGS (*atpB*-*rbcL*) |
| *Preptanthe rubens* | 30 | 60280 | 60289 | (A)10 | LSC; IGS (*accD*-*psaI*);CDS(*accD*) |
| *Preptanthe rubens* | 31 | 63620 | 63631 | (AATG)3 | LSC; CDS(*cemA*) |
| *Preptanthe rubens* | 32 | 64874 | 64883 | (T)10 | LSC; IGS (*petA*-*psbJ*) |
| *Preptanthe rubens* | 33 | 65336 | 65360 | (TAT)4(TA)8* | LSC; IGS (*petA*-*psbJ*) |
| *Preptanthe rubens* | 34 | 68662 | 68671 | (TA)5 | LSC; IGS (*trnP*-UGG-*psaJ*) |
| *Preptanthe rubens* | 35 | 69172 | 69182 | (A)11 | LSC; IGS (*psaJ*-*rpl33*) |
| *Preptanthe rubens* | 36 | 69353 | 69364 | (ATTA)3 | LSC; IGS (*psaJ*-*rpl33*) |
| *Preptanthe rubens* | 37 | 69567 | 69577 | (T)11 | LSC; IGS (*psaJ*-*rpl33*) |
| *Preptanthe rubens* | 38 | 69867 | 69876 | (TG)5 | LSC; IGS (*rpl33*-*rps18*) |
| *Preptanthe rubens* | 39 | 70618 | 70627 | (T)10 | LSC; IGS (*rps18*-*rpl20*);CDS(*rpl20*) |
| *Preptanthe rubens* | 40 | 72775 | 72784 | (A)10 | LSC; CDS(*clpP* intron) |
| *Preptanthe rubens* | 41 | 73313 | 73322 | (T)10 | LSC; CDS(*clpP* intron) |
| *Preptanthe rubens* | 42 | 74310 | 74319 | (TA)5 | LSC; IGS (*clpP*-*psbB*) |
| *Preptanthe rubens* | 43 | 74433 | 74444 | (TAT)4 | LSC; IGS (*clpP*-*psbB*) |
| *Preptanthe rubens* | 44 | 76977 | 76988 | (AAT)4 | LSC; IGS (*psbB*-*psbT*) |
| *Preptanthe rubens* | 45 | 78170 | 78179 | (A)10 | LSC; CDS(*petB* intron) |
| *Preptanthe rubens* | 46 | 82873 | 82883 | (T)11 | LSC; IGS (*rpl36*-*infA*) |
| *Preptanthe rubens* | 47 | 85824 | 85833 | (T)10 | LSC; CDS(*rpl16* intron) |
| *Preptanthe rubens* | 48 | 86012 | 86023 | (T)12 | LSC; CDS(*rpl16* intron) |
| *Preptanthe rubens* | 49 | 86176 | 86187 | (A)12 | LSC; IGS (*rpl16*-*rps3*) |
| *Preptanthe rubens* | 50 | 93363 | 93372 | (GA)5 | IR1; CDS(*ycf2*) |
| *Preptanthe rubens* | 51 | 103528 | 103537 | (T)10 | IR1; IGS (*rps12*-*trnV*-GAC) |
| *Preptanthe rubens* | 52 | 116496 | 116507 | (TA)6 | SSC; IGS (*ndhF*-*rpl32*) |
| *Preptanthe rubens* | 53 | 116781 | 116790 | (T)10 | SSC; IGS (*ndhF*-*rpl32*) |
| *Preptanthe rubens* | 54 | 121644 | 121655 | (TTGA)3 | SSC; CDS(*ndhE*) |
| *Preptanthe rubens* | 55 | 122657 | 122668 | (AAAT)3 | SSC; IGS (*nad6*-*ndhI*) |
| *Preptanthe rubens* | 56 | 124999 | 125009 | (A)11 | SSC; CDS(*ndhA* intron) |
| *Preptanthe rubens* | 57 | 128481 | 128490 | (T)10 | SSC; CDS(*ycf1*) |
| *Preptanthe rubens* | 58 | 129446 | 129455 | (T)10 | SSC; CDS(*ycf1*) |
| *Preptanthe rubens* | 59 | 130467 | 130476 | (A)10 | SSC; CDS(*ycf1*) |
| *Preptanthe rubens* | 60 | 131772 | 131785 | (T)14 | SSC; CDS(*ycf1*) |
| *Preptanthe rubens* | 61 | 131893 | 131902 | (T)10 | SSC; CDS(*ycf1*) |
| *Preptanthe rubens* | 62 | 132054 | 132065 | (A)12 | SSC; CDS(*ycf1*) |
| *Preptanthe rubens* | 63 | 142281 | 142290 | (A)10 | IR2; CDS(*rps12* intron) |
| *Preptanthe rubens* | 64 | 152446 | 152455 | (TC)5 | IR2; CDS(*ycf2*) |
| *Calanthe triplicata* | 1 | 1421 | 1430 | (T)10 | LSC; IGS (*psbA*-*trnK*-UUU) |
| *Calanthe triplicata* | 2 | 5261 | 5272 | (ATAG)3 | LSC; CDS (*rpl16* intron) |
| *Calanthe triplicata* | 3 | 5449 | 5466 | (TA)9 | LSC; CDS (*rpl16* intron) |
| *Calanthe triplicata* | 4 | 6441 | 6450 | (A)10 | LSC; IGS (*rps16*-*trnQ*-UUG) |
| *Calanthe triplicata* | 5 | 8223 | 8232 | (T)10 | LSC; IGS (*psbK*-psbI) |
| *Calanthe triplicata* | 6 | 8310 | 8324 | (ATCTT)3 | LSC; IGS (*psbK*-*psbI*) |
| *Calanthe triplicata* | 7 | 9644 | 9653 | (A)10 | LSC; IGS (*trnS*-GCU-*trnG*-GCC) |
| *Calanthe triplicata* | 8 | 9710 | 9719 | (T)10 | LSC; IGS (*trnS*-GCU-*trnG*-GCC) |
| *Calanthe triplicata* | 9 | 11993 | 12004 | (GTCT)3 | LSC; CDS (*atpA*) |
| *Calanthe triplicata* | 10 | 13295 | 13304 | (A)10 | LSC;CDS (*atpF* intron) |
| *Calanthe triplicata* | 11 | 16925 | 16934 | (T)10 | LSC; IGS (*rps2*-*rpoC2*) |
| *Calanthe triplicata* | 12 | 19024 | 19033 | (T)10 | LSC; CDS (*rpoC2*) |
| *Calanthe triplicata* | 13 | 19130 | 19140 | (T)11 | LSC; CDS (*rpoC2*) |
| *Calanthe triplicata* | 14 | 20509 | 20518 | (AT)5 | LSC; CDS (*rpoC2*) |
| *Calanthe triplicata* | 15 | 28887 | 28898 | (AT)6 | LSC; IGS (*rpoB*-*trnC*-GCA) |
| *Calanthe triplicata* | 16 | 28919 | 28930 | (TTTA)3 | LSC; IGS (*rpoB*-*trnC*-GCA) |
| *Calanthe triplicata* | 17 | 32846 | 32859 | (AT)7 | LSC; IGS (*trnE*-UUC-*trnT*-GGU) |
| *Calanthe triplicata* | 18 | 33557 | 33566 | (A)10 | LSC; IGS (*trnT*-GGU-*psbD*) |
| *Calanthe triplicata* | 19 | 43426 | 43435 | (A)10 | LSC; IGS (*psaA*-*ycf3*) |
| *Calanthe triplicata* | 20 | 48957 | 48966 | (T)10 | LSC; IGS (*trnL*-UAA-*trnF*-GAA) |
| *Calanthe triplicata* | 21 | 51027 | 51036 | (T)10 | LSC; CDS (*ndhK*) |
| *Calanthe triplicata* | 22 | 52607 | 52618 | (A)12 | LSC; IGS (*ndhC*-*trnV*-UAC) |
| *Calanthe triplicata* | 23 | 52773 | 52784 | (CTA)4 | LSC; IGS (*ndhC*-*trnV*-UAC) |
| *Calanthe triplicata* | 24 | 52795 | 52809 | (ACAAA)3 | LSC; IGS (*ndhC*-*trnV*-UAC) |
| *Calanthe triplicata* | 25 | 63015 | 63024 | (T)10 | LSC; IGS (*ycf4*-*cemA*) |
| *Calanthe triplicata* | 26 | 63930 | 63941 | (AATG)3 | LSC; IGS (*cemA*-*petA*) |
| *Calanthe triplicata* | 27 | 65176 | 65186 | (T)11 | LSC; IGS (*petA*-*psbJ*) |
| *Calanthe triplicata* | 28 | 66514 | 66523 | (A)10 | LSC; IGS (*psbE*-*petL*) |
| *Calanthe triplicata* | 29 | 69258 | 69269 | (ATTA)3 | LSC; IGS (*psaJ*-*rpl33*) |
| *Calanthe triplicata* | 30 | 69751 | 69760 | (TG)5 | LSC; IGS (*rpl33*-*rps18*) |
| *Calanthe triplicata* | 31 | 70508 | 70517 | (T)10 | LSC; CDS (*rpl20*) |
| *Calanthe triplicata* | 32 | 71878 | 71895 | (TTATAT)3 | LSC; CDS (*clpP* intron) |
| *Calanthe triplicata* | 33 | 73211 | 73220 | (T)10 | LSC; CDS (*clpP* intron) |
| *Calanthe triplicata* | 34 | 76993 | 77002 | (TA)5 | LSC; IGS (*psbB*-psbT) |
| *Calanthe triplicata* | 35 | 78376 | 78391 | (A)16 | LSC; CDS (*petB* intron) |
| *Calanthe triplicata* | 36 | 79782 | 79793 | (T)12 | LSC; CDS (*petD* intron) |
| *Calanthe triplicata* | 37 | 82899 | 82908 | (A)10 | LSC; IGS (*rpl36*-*infA*) |
| *Calanthe triplicata* | 38 | 83959 | 83970 | (TA)6 | LSC; IGS (*rps8*-*rpl14*) |
| *Calanthe triplicata* | 39 | 93341 | 93350 | (GA)5 | IR1; CDS (*ycf2*) |
| *Calanthe triplicata* | 40 | 102972 | 102983 | (TAT)4 | IR1; IGS (*rps12*-*trnV*-GAC) |
| *Calanthe triplicata* | 41 | 111295 | 111305 | (A)11 | SSC; IGS (*rrn4*.5-*rrn5*) |
| *Calanthe triplicata* | 42 | 116538 | 116547 | (TA)5 | SSC; IGS (*ndhF*-*rpl32*) |
| *Calanthe triplicata* | 43 | 116640 | 116657 | (TA)9 | SSC; IGS (*ndhF*-*rpl32*) |
| *Calanthe triplicata* | 44 | 121632 | 121643 | (AAT)4 | SSC; IGS (*psaC*-*ndhE*) |
| *Calanthe triplicata* | 45 | 121794 | 121805 | (TTGA)3 | SSC; CDS (*ndhE*) |
| *Calanthe triplicata* | 46 | 122245 | 122254 | (A)10 | SSC; CDS (*nad6*) |
| *Calanthe triplicata* | 47 | 124698 | 124707 | (A)10 | SSC; CDS (*ndhA* intron) |
| *Calanthe triplicata* | 48 | 125170 | 125181 | (A)12 | SSC; CDS (*ndhA* intron) |
| *Calanthe triplicata* | 49 | 128696 | 128705 | (T)10 | SSC; CDS (*ycf1*) |
| *Calanthe triplicata* | 50 | 130628 | 130637 | (A)10 | SSC; CDS (*ycf1*) |
| *Calanthe triplicata* | 51 | 131942 | 131955 | (T)14 | SSC; CDS (*ycf1*) |
| *Calanthe triplicata* | 52 | 132063 | 132072 | (T)10 | SSC; CDS (*ycf1*) |
| *Calanthe triplicata* | 53 | 132196 | 132206 | (T)11 | SSC; CDS (*ycf1*) |
| *Calanthe triplicata* | 54 | 132224 | 132235 | (A)12 | SSC; CDS (*ycf1*) |
| *Calanthe triplicata* | 55 | 134866 | 134876 | (T)11 | SSC; CDS (*ycf1*) |
| *Calanthe triplicata* | 56 | 143187 | 143198 | (AAT)4 | IR2; CDS (*rps12* intron) |
| *Calanthe triplicata* | 57 | 152821 | 152830 | (TC)5 | IR2; CDS (*ycf2*) |
| *Cephalantheropsis obcordata* | 1 | 1376 | 1385 | (T)10 | LSC; IGS (*psbA*-*trnK*-UUU) |
| *Cephalantheropsis obcordata* | 2 | 5191 | 5202 | (ATAG)3 | LSC; CDS (*rps16* intron) |
| *Cephalantheropsis obcordata* | 3 | 5385 | 5394 | (T)10 | LSC; CDS (*rps16* intron) |
| *Cephalantheropsis obcordata* | 4 | 6840 | 6849 | (A)10 | LSC; IGS (*rps16*-*trnQ*-UUG) |
| *Cephalantheropsis obcordata* | 5 | 7945 | 7954 | (T)10 | LSC; IGS (*psbK*-*psbI*) |
| *Cephalantheropsis obcordata* | 6 | 8031 | 8045 | (ATCTT)3 | LSC; IGS (*psbK*-psbI) |
| *Cephalantheropsis obcordata* | 7 | 9225 | 9234 | (A)10 | LSC; IGS (*trnS*-GCU-*trnG*-GCC) |
| *Cephalantheropsis obcordata* | 8 | 9291 | 9301 | (T)11 | LSC; IGS (*trnS*-GCU-*trnG*-GCC) |
| *Cephalantheropsis obcordata* | 9 | 11576 | 11587 | (GTCT)3 | LSC; CDS (*atpA*) |
| *Cephalantheropsis obcordata* | 10 | 13785 | 13796 | (T)12 | LSC; IGS (*atpF*-*atpH*) |
| *Cephalantheropsis obcordata* | 11 | 16564 | 16574 | (T)11 | LSC; IGS (*rps2*-*rpoC2*) |
| *Cephalantheropsis obcordata* | 12 | 18679 | 18688 | (T)10 | LSC; CDS (*rpoC2*) |
| *Cephalantheropsis obcordata* | 13 | 18785 | 18795 | (T)11 | LSC; CDS (*rpoC2*) |
| *Cephalantheropsis obcordata* | 14 | 20164 | 20173 | (AT)5 | LSC; CDS (*rpoC2*) |
| *Cephalantheropsis obcordata* | 15 | 30629 | 30640 | (AGAA)3 | LSC; IGS (*psbM*-*trnD*-GUC) |
| *Cephalantheropsis obcordata* | 16 | 33007 | 33016 | (A)10 | LSC; IGS (*trnT*-GGU-*psbD*) |
| *Cephalantheropsis obcordata* | 17 | 33355 | 33365 | (T)11 | LSC; IGS (*trnT*-GGU-*psbD*) |
| *Cephalantheropsis obcordata* | 18 | 46862 | 46873 | (TA)6 | LSC; IGS (*rps4*-*trnT*-UGU) |
| *Cephalantheropsis obcordata* | 19 | 47341 | 47354 | (AT)7 | LSC; IGS (*trnT*-UGU-*trnL*-UAA) |
| *Cephalantheropsis obcordata* | 20 | 47399 | 47408 | (A)10 | LSC; IGS (*trnT*-UGU-*trnL*-UAA) |
| *Cephalantheropsis obcordata* | 21 | 48407 | 48417 | (T)11 | LSC; IGS (*trnL*-UAA-*trnF*-GAA) |
| *Cephalantheropsis obcordata* | 22 | 48556 | 48569 | (AT)7 | LSC; IGS (*trnL*-UAA-*trnF*-GAA) |
| *Cephalantheropsis obcordata* | 23 | 50663 | 50672 | (T)10 | LSC; CDS (*ndhK*) |
| *Cephalantheropsis obcordata* | 24 | 51581 | 51590 | (A)10 | LSC; IGS (*ndhC*-*trnV*-UAC) |
| *Cephalantheropsis obcordata* | 25 | 52105 | 52119 | (ACAAA)3 | LSC; IGS (*ndhC*-*trnV*-UAC) |
| *Cephalantheropsis obcordata* | 26 | 55326 | 55335 | (T)10 | LSC; IGS (*atpB*-*rbcL*) |
| *Cephalantheropsis obcordata* | 27 | 55519 | 55538 | (TA)10 | LSC; IGS (*atpB*-*rbcL*) |
| *Cephalantheropsis obcordata* | 28 | 60570 | 60581 | (ATA)4 | LSC; IGS (*accD*-*psaI*) |
| *Cephalantheropsis obcordata* | 29 | 63109 | 63120 | (AATG)3 | LSC; CDS (*cemA*) |
| *Cephalantheropsis obcordata* | 30 | 64362 | 64372 | (T)11 | LSC; IGS (*petA*-*psbJ*) |
| *Cephalantheropsis obcordata* | 31 | 64829 | 64844 | (TA)8 | LSC; IGS (*petA*-*psbJ*) |
| *Cephalantheropsis obcordata* | 32 | 68652 | 68661 | (A)10 | LSC; IGS (*psbJ*-*rpl33*) |
| *Cephalantheropsis obcordata* | 33 | 68832 | 68843 | (ATTA)3 | LSC; IGS (*psbJ*-*rpl33*) |
| *Cephalantheropsis obcordata* | 34 | 69341 | 69350 | (TG)5 | LSC; IGS (*rpl33*-*rps18*) |
| *Cephalantheropsis obcordata* | 35 | 69444 | 69453 | (A)10 | LSC; IGS (*rpl33*-*rps18*) |
| *Cephalantheropsis obcordata* | 36 | 72795 | 72804 | (T)10 | LSC; CDS (*clpP* intron) |
| *Cephalantheropsis obcordata* | 37 | 73049 | 73058 | (T)10 | LSC; CDS (*clpP* intron) |
| *Cephalantheropsis obcordata* | 38 | 73261 | 73275 | (TTATA)3 | LSC; CDS (*clpP* intron) |
| *Cephalantheropsis obcordata* | 39 | 76089 | 76100 | (TA)6 | LSC; IGS (*psbB*-*psbT*) |
| *Cephalantheropsis obcordata* | 40 | 76303 | 76314 | (AAT)4 | LSC; IGS (*psbB*-*psbT*) |
| *Cephalantheropsis obcordata* | 41 | 76356 | 76365 | (TA)5 | LSC; IGS (*psbB*-*psbT*) |
| *Cephalantheropsis obcordata* | 42 | 77737 | 77750 | (A)14 | LSC; CDS (*petB* intron) |
| *Cephalantheropsis obcordata* | 43 | 79131 | 79140 | (T)10 | LSC; CDS (*petD* intron) |
| *Cephalantheropsis obcordata* | 44 | 83149 | 83158 | (T)10 | LSC; IGS (*rps8*-*rpl14*) |
| *Cephalantheropsis obcordata* | 45 | 83261 | 83270 | (AT)5 | LSC; IGS (*rps8*-*rpl14*) |
| *Cephalantheropsis obcordata* | 46 | 83802 | 83813 | (CTT)4 | LSC; IGS (*rpl14*-*rpl16*) |
| *Cephalantheropsis obcordata* | 47 | 84321 | 84332 | (AAT)4 | LSC; CDS (*rpl16* intron) |
| *Cephalantheropsis obcordata* | 48 | 92725 | 92734 | (GA)5 | IR1; CDS (*ycf2*) |
| *Cephalantheropsis obcordata* | 49 | 95477 | 95494 | (TAGAAG)3 | IR1; CDS (*ycf2*) |
| *Cephalantheropsis obcordata* | 50 | 105662 | 105671 | (G)10 | IR1; tRNA (*trnI*-GAU) |
| *Cephalantheropsis obcordata* | 51 | 116647 | 116656 | (T)10 | SSC; IGS (*rpl32*-*trnL*-UAG) |
| *Cephalantheropsis obcordata* | 52 | 120706 | 120715 | (AT)5 | SSC; IGS (*psaC*-*ndhE*) |
| *Cephalantheropsis obcordata* | 53 | 120869 | 120880 | (AAT)4 | SSC; IGS (*psaC*-*ndhE*) |
| *Cephalantheropsis obcordata* | 54 | 121031 | 121042 | (TTGA)3 | SSC; CDS (*ndhE*) |
| *Cephalantheropsis obcordata* | 55 | 121463 | 121472 | (A)10 | SSC; IGS (*ndhE*-*nad6*) |
| *Cephalantheropsis obcordata* | 56 | 124369 | 124378 | (A)10 | SSC; CDS (*ndhA* intron) |
| *Cephalantheropsis obcordata* | 57 | 129660 | 129670 | (T)11 | SSC; CDS (*ycf1*) |
| *Cephalantheropsis obcordata* | 58 | 129783 | 129792 | (A)10 | SSC; CDS (*ycf1*) |
| *Cephalantheropsis obcordata* | 59 | 131085 | 131098 | (T)14 | SSC; CDS (*ycf1*) |
| *Cephalantheropsis obcordata* | 60 | 131206 | 131215 | (T)10 | SSC; CDS (*ycf1*) |
| *Cephalantheropsis obcordata* | 61 | 131367 | 131378 | (A)12 | SSC; CDS (*ycf1*) |
| *Cephalantheropsis obcordata* | 62 | 138900 | 138909 | (C)10 | IR2; tRNA (*trnI*-GAU) |
| *Cephalantheropsis obcordata* | 63 | 149077 | 149094 | (CTTCTA)3 | IR2; CDS (*ycf2*) |
| *Cephalantheropsis obcordata* | 64 | 151837 | 151846 | (TC)5 | IR2; CDS (*ycf2*) |
| *Phaius tankervilliae* | 1 | 1662 | 1672 | (T)11 | LSC; tRNA (*trnK*-UUU) |
| *Phaius tankervilliae* | 2 | 3449 | 3463 | (ATA)5 | LSC; tRNA (*trnK*-UUU) |
| *Phaius tankervilliae* | 3 | 5222 | 5233 | (TCTA)3 | LSC; CDS (*rps16*) |
| *Phaius tankervilliae* | 4 | 6782 | 6791 | (A)10 | LSC; IGS (*rps16*-*trnQ*-UUG) |
| *Phaius tankervilliae* | 5 | 8012 | 8026 | (ATCTT)3 | LSC; IGS (*psbK*-*psbI*) |
| *Phaius tankervilliae* | 6 | 9267 | 9277 | (T)11 | LSC; IGS (*trnS*-GCU-*trnG*-GCC) |
| *Phaius tankervilliae* | 7 | 10422 | 10431 | (A)10 | LSC; IGS (*trnR*-UCU-*atpA*) |
| *Phaius tankervilliae* | 8 | 11547 | 11558 | (GTCT)3 | LSC; CDS (*atpA*) |
| *Phaius tankervilliae* | 9 | 13757 | 13767 | (T)11 | LSC; IGS (*atpF*-*atpH*) |
| *Phaius tankervilliae* | 10 | 14588 | 14599 | (AT)6 | LSC; IGS (*atpH*-*atpI*) |
| *Phaius tankervilliae* | 11 | 16535 | 16544 | (T)10 | LSC; IGS (*rps2*-*rpoC2*) |
| *Phaius tankervilliae* | 12 | 18639 | 18648 | (T)10 | LSC; CDS (*rpoC2*) |
| *Phaius tankervilliae* | 13 | 18745 | 18755 | (T)11 | LSC; CDS (*rpoC2*) |
| *Phaius tankervilliae* | 14 | 20124 | 20133 | (AT)5 | LSC; CDS (*rpoC2*) |
| *Phaius tankervilliae* | 15 | 29595 | 29606 | (TAT)4 | LSC; IGS (*petN*-*psbM*) |
| *Phaius tankervilliae* | 16 | 32793 | 32802 | (A)10 | LSC; IGS (*trnT*-GGU-*psbD*) |
| *Phaius tankervilliae* | 17 | 36409 | 36418 | (A)10 | LSC; IGS (*trnS*-UGA-*psbZ*) |
| *Phaius tankervilliae* | 18 | 42630 | 42639 | (A)10 | LSC; IGS (*psaA*-*ycf3*) |
| *Phaius tankervilliae* | 19 | 42673 | 42684 | (T)12 | LSC; IGS (*psaA*-*ycf3*) |
| *Phaius tankervilliae* | 20 | 50488 | 50497 | (T)10 | LSC; CDS (*ndhK*) |
| *Phaius tankervilliae* | 21 | 52196 | 52210 | (ACAAA)3 | LSC; IGS (*ndhC*-*trnV*-UAC) |
| *Phaius tankervilliae* | 22 | 53037 | 53046 | (T)10 | LSC; IGS (*trnV*-UAC-*trnM*-CAU) |
| *Phaius tankervilliae* | 23 | 55427 | 55438 | (T)12 | LSC; IGS (*atpB*-*rbcL*) |
| *Phaius tankervilliae* | 24 | 58441 | 58450 | (A)10 | LSC; IGS (*rbcL*-*accD*) |
| *Phaius tankervilliae* | 25 | 67524 | 67541 | (AAAATC)3 | LSC; IGS (*petL*-*petG*) |
| *Phaius tankervilliae* | 26 | 69212 | 69222 | (T)11 | LSC; IGS (*psaJ*-*rpl33*) |
| *Phaius tankervilliae* | 27 | 69511 | 69520 | (TG)5 | LSC; IGS (*rpl33*-*rps18*) |
| *Phaius tankervilliae* | 28 | 70921 | 70935 | (TTTCA)3 | LSC; IGS (*rpl20*-*rps12*) |
| *Phaius tankervilliae* | 29 | 72962 | 72972 | (T)11 | LSC; CDS (*clpP* intron) |
| *Phaius tankervilliae* | 30 | 73982 | 73991 | (TA)5 | LSC; IGS (*clpP*-*psbB*) |
| *Phaius tankervilliae* | 31 | 74113 | 74124 | (TAGA)3 | LSC; IGS (*clpP*-*psbB*) |
| *Phaius tankervilliae* | 32 | 79323 | 79332 | (T)10 | LSC; IGS (*petB*-*petD*) |
| *Phaius tankervilliae* | 33 | 83345 | 83354 | (T)10 | LSC; IGS (*rps8*-*rpl14*) |
| *Phaius tankervilliae* | 34 | 85407 | 85416 | (T)10 | LSC; CDS (*rpl16* intron) |
| *Phaius tankervilliae* | 35 | 85735 | 85744 | (A)10 | LSC; IGS (*rpl16*-*rps3*) |
| *Phaius tankervilliae* | 36 | 92918 | 92927 | (GA)5 | IR1; CDS (*ycf2*) |
| *Phaius tankervilliae* | 37 | 102867 | 102884 | (TCTGTC)3 | IR1; IGS (*rps12*-*trnV*-GAC) |
| *Phaius tankervilliae* | 38 | 115953 | 115963 | (T)11 | SSC; IGS (*ndhF*-*rpl32*) |
| *Phaius tankervilliae* | 39 | 120921 | 120932 | (AT)6 | SSC; IGS (*psaC*-*ndhE*) |
| *Phaius tankervilliae* | 40 | 121162 | 121173 | (TTGA)3 | SSC; CDS (*ndhE*) |
| *Phaius tankervilliae* | 41 | 121604 | 121613 | (A)10 | SSC; CDS (*ndhE*) |
| *Phaius tankervilliae* | 42 | 124505 | 124515 | (A)11 | SSC; CDS (*ndhA* intron) |
| *Phaius tankervilliae* | 43 | 129778 | 129787 | (T)10 | SSC; CDS (*ycf1*) |
| *Phaius tankervilliae* | 44 | 129900 | 129909 | (A)10 | SSC; CDS (*ycf1*) |
| *Phaius tankervilliae* | 45 | 131199 | 131212 | (T)14 | SSC; CDS (*ycf1*) |
| *Phaius tankervilliae* | 46 | 131320 | 131329 | (T)10 | SSC; CDS (*ycf1*) |
| *Phaius tankervilliae* | 47 | 131481 | 131492 | (A)12 | SSC; CDS (*ycf1*) |
| *Phaius tankervilliae* | 48 | 141982 | 141999 | (GAGACA)3 | IR2; CDS (*rps12* intron) |
| *Phaius tankervilliae* | 49 | 151941 | 151950 | (TC)5 | IR2; CDS (*ycf2*) |
