# Supplementary material for: Plastome structure and adaptive evolution of Calanthe s.l. species
Source: PeerJ. 2020 Oct 13;8:e10051. doi: 10.7717/peerj.10051 (PMC7566753; doi:10.7717/peerj.10051)
Supplement: Supplemental Information 8 [file peerj-08-10051-s008.docx]

### **Table S8** Maximum likelihood parameter estimates for the 76 genes of seven *Calanthe* s.l species.

| **Gene** | **Model** | **l** | **np** | **Estimates of parameters** | | **Positively selected sites** |
| --- | --- | --- | --- | --- | --- | --- |
| *acc*D | model0 | -2344.138442 | 13 | omega (dN/dS) = 0.37025 |  |  |
|  | model1 | -2336.67225 | 14 | p=0.70185 0.29815 | w=0.00000 1.00000 |  |
|  | model2 | -2331.75842 | 16 | p=0.78239 0.20217 0.01544 | w=0.09941 1.00000 13.38877 | 180 L0.982* 441 V0.981* |
|  | model3 | -2331.761218 | 17 | p=0.21637 0.75887 0.02476 | w=0.23601 0.23604 10.20282 |  |
|  | model7 | -2336.672661 | 14 | p=0.00500 q=0.01169 |  |  |
|  | model8 | -2331.760119 | 16 | p0=0.98494 p=0.15463 q=0.38585 | (p1=0.01506) w=13.63712 | 180 L0.989* 441 V0.987* |
| *atp*A | model0 | -2258.555426 | 13 | omega (dN/dS) = 0.15596 |  |  |
|  | model1 | -2257.081787 | 14 | p=0.84273 0.15727 | w=0.00000 1.00000 |  |
|  | model2 | -2256.935871 | 16 | p=0.90171 0.00000 0.09829 | w=0.00000 1.00000 1.76049 |  |
|  | model3 | -2256.935871 | 17 | p=0.69827 0.20343 0.09830 | w=0.00000 0.00000 1.76039 |  |
|  | model7 | -2257.1785 | 14 | p=0.01389 q=0.07603 |  |  |
|  | model8 | -2256.935872 | 16 | p0=0.90171 p=0.00500 q=1.52536 | (p1=0.09829) w=1.76050 |  |
| *atp*B | model0 | -2175.394694 | 13 | omega (dN/dS) = 0.07833 |  |  |
|  | model1 | -2175.394735 | 14 | p=0.99999 0.00001 | w=0.07833 1.00000 |  |
|  | model2 | -2175.394694 | 16 | p=1.00000 0.00000 0.00000 | w=0.07834 1.00000 1.00000 |  |
|  | model3 | -2175.394694 | 17 | p=0.43236 0.40296 0.16468 | w=0.07832 0.07833 0.07838 |  |
|  | model7 | -2175.397817 | 14 | p=8.45803 q = 99.00000 |  |  |
|  | model8 | -2175.397918 | 16 | p0=0.99999 p=8.47443 q = 99.00000 | (p1=0.00001) w=1.00000 |  |
| *atp*E | model0 | -576.376854 | 13 | omega (dN/dS) = 0.11620 |  |  |
|  | model1 | -576.376865 | 14 | p=0.99999 0.00001 | w=0.11619 1.00000 |  |
|  | model2 | -576.376854 | 16 | p=1.00000 0.00000 0.00000 | w=0.11620 1.00000 1.00000 |  |
|  | model3 | -576.376854 | 17 | p=0.43061 0.16471 0.40468 | w=0.11620 0.11620 0.11620 |  |
|  | model7 | -576.378107 | 14 | p = 13.06473 q = 99.00000 |  |  |
|  | model8 | -576.378118 | 16 | p0=0.99999 p = 13.06384 q = 99.00000 | (p1=0.00001) w=1.00000 |  |
| *atp*F | model0 | -831.21746 | 13 | omega (dN/dS) = 0.31580 |  |  |
|  | model1 | -831.217464 | 14 | p=0.99999 0.00001 | w=0.31580 1.00000 |  |
|  | model2 | -831.21746 | 16 | p=1.00000 0.00000 0.00000 | w=0.31581 1.00000 1.00000 |  |
|  | model3 | -831.21746 | 17 | p=0.16370 0.43215 0.40416 | w=0.31580 0.31581 0.31581 |  |
|  | model7 | -831.218548 | 14 | p = 45.77077 q = 99.00000 |  |  |
|  | model8 | -831.218551 | 16 | p0=0.99999 p =45.77061 q = 99.00000 | (p1=0.00001) w=1.00000 |  |
| *atp*H | model0 | -341.787317 | 13 | omega (dN/dS) = 0.04640 |  |  |
|  | model1 | -341.700734 | 14 | p=0.94476 0.05524 | w=0.00000 1.00000 |  |
|  | model2 | -341.503547 | 16 | p=0.98289 0.00000 0.01711 | w=0.00000 1.00000 7.55552 |  |
|  | model3 | -341.503548 | 17 | p=0.49884 0.48405 0.01711 | w=0.00000 0.00000 7.55554 |  |
|  | model7 | -341.742267 | 14 | p=0.01172 q=0.21016 |  |  |
|  | model8 | -341.503542 | 16 | p0=0.98289 p=0.00500 q = 99.00000 | (p1=0.01711) w=7.55591 |  |
| *atp*I | model0 | -1070.212963 | 13 | omega (dN/dS) = 0.11288 |  |  |
|  | model1 | -1070.212972 | 14 | p=0.99999 0.00001 | w=0.11287 1.00000 |  |
|  | model2 | -1070.212963 | 16 | p=1.00000 0.00000 0.00000 | w=0.11288 1.00000 1.00000 |  |
|  | model3 | -1070.212963 | 17 | p=0.42013 0.16557 0.41430 | w=0.11287 0.11289 0.11289 |  |
|  | model7 | -1070.213947 | 14 | p = 12.64287 q = 99.00000 |  |  |
|  | model8 | -1070.213958 | 16 | p0=0.99999 p = 12.65313 q = 99.00000 | (p1=0.00001) w=1.00000 |  |
| *ccs*A | model0 | -1497.755603 | 13 | omega (dN/dS) = 0.23059 |  |  |
|  | model1 | -1495.745937 | 14 | p=0.77712 0.22288 | w=0.00000 1.00000 |  |
|  | model2 | -1495.619613 | 16 | p=0.96589 0.00000 0.03411 | w=0.12792 1.00000 3.65682 |  |
|  | model3 | -1495.619613 | 17 | p=0.54522 0.42067 0.03411 | w=0.12792 0.12793 3.65703 |  |
|  | model7 | -1495.788138 | 14 | p=0.00954 q=0.03113 |  |  |
|  | model8 | -1495.619818 | 16 | p0=0.96597 p = 14.59864 q = 99.00000 | (p1=0.03403) w=3.65406 |  |
| *cem*A | model0 | -1467.943772 | 13 | omega (dN/dS) = 0.16830 |  |  |
|  | model1 | -1451.32835 | 14 | p=0.32891 0.67109 | w=0.02141 1.00000 |  |
|  | model2 | -1451.32835 | 16 | p=0.32891 0.54319 0.12790 | w=0.02141 1.00000 1.00000 |  |
|  | model3 | -1450.394291 | 17 | p=0.10824 0.17906 0.71270 | w=0.01106 0.01106 0.45119 |  |
|  | model7 | -1450.736405 | 14 | p=0.28360 q=0.34487 |  |  |
|  | model8 | -1450.736406 | 16 | p0=0.99999 p=0.28360 q=0.34488 | (p1=0.00001) w=1.00000 |  |
| *clp*P | model0 | -902.115417 | 13 | omega (dN/dS) = 0.06699 |  |  |
|  | model1 | -902.11546 | 14 | p=0.99999 0.00001 | w=0.06698 1.00000 |  |
|  | model2 | -902.115417 | 16 | p=1.00000 0.00000 0.00000 | w=0.06699 1.00000 1.00000 |  |
|  | model3 | -902.115417 | 17 | p=0.40610 0.42900 0.16490 | w=0.06699 0.06699 0.06699 |  |
|  | model7 | -902.118395 | 14 | p=7.15470 q = 99.00000 |  |  |
|  | model8 | -902.119463 | 16 | p0=0.99999 p=7.14583 q = 99.00000 | (p1=0.00001) w=7.18407 |  |
| *inf*A | model0 | -511.005132 | 13 | omega (dN/dS) = 0.02673 |  |  |
|  | model1 | -511.005233 | 14 | p=0.99999 0.00001 | w=0.02673 1.00000 |  |
|  | model2 | -509.698309 | 16 | p=0.19390 0.64769 0.15841 | w=0.00613 1.00000 1.00000 |  |
|  | model3 | -505.549075 | 17 | p=0.15921 0.57053 0.27026 | w=0.00078 0.08118 0.08118 |  |
|  | model7 | -509.632108 | 14 | p=0.02544 q=0.00500 |  |  |
|  | model8 | -506.878309 | 16 | p0=0.99999 p=0.66772 q=6.36445 | (p1=0.00001) w=1.00000 |  |
| *mat*K | model0 | -2763.894449 | 13 | omega (dN/dS) = 0.53260 |  |  |
|  | model1 | -2760.86906 | 14 | p=0.48587 0.51413 | w=0.00000 1.00000 |  |
|  | model2 | -2760.444903 | 16 | p=0.58512 0.38535 0.02953 | w=0.11330 1.00000 4.00868 |  |
|  | model3 | -2760.444621 | 17 | p=0.41566 0.54965 0.03469 | w=0.00000 0.79172 3.88696 |  |
|  | model7 | -2760.883295 | 14 | p=0.00500 q=0.00505 |  |  |
|  | model8 | -2760.444765 | 16 | p0=0.96785 p=0.20429 q=0.24162 | (p1=0.03215) w=3.93797 |  |
| *nad*6 | model0 | -285.738089 | 13 | omega (dN/dS) = 0.04919 |  |  |
|  | model1 | -285.650704 | 14 | p=0.94177 0.05823 | w=0.00000 1.00000 |  |
|  | model2 | -285.402914 | 16 | p=0.98157 0.00000 0.01843 | w=0.00000 1.00000 8.54904 |  |
|  | model3 | -285.510314 | 17 | p=0.42454 0.55709 0.01837 | w=0.00000 0.00000 8.66855 |  |
|  | model7 | -285.689556 | 14 | p=0.01026 q=0.18104 |  |  |
|  | model8 | -285.402907 | 16 | p0=0.98157 p=0.00500 q = 99.00000 | (p1=0.01843) w=8.54901 |  |
| *ndh*A | model0 | -1040.446765 | 13 | omega (dN/dS) = 0.21026 |  |  |
|  | model1 | -1039.201499 | 14 | p=0.80481 0.19519 | w=0.00000 1.00000 |  |
|  | model2 | -1039.010934 | 16 | p=0.88731 0.00000 0.11269 | w=0.00000 1.00000 2.02899 |  |
|  | model3 | -1039.010934 | 17 | p=0.34073 0.54657 0.11269 | w=0.00000 0.00000 2.02899 |  |
|  | model7 | -1039.202867 | 14 | p=0.00500 q=0.02038 |  |  |
|  | model8 | -1039.010934 | 16 | p0=0.88731 p=0.00500 q=1.27146 | (p1=0.11269) w=2.02901 |  |
| *ndh*B | model0 | -2153.277556 | 13 | omega (dN/dS) = 1.02913 |  |  |
|  | model1 | -2144.434796 | 14 | p=0.73496 0.26504 | w=0.00000 1.00000 |  |
|  | model2 | -2098.553322 | 16 | p=0.96629 0.00000 0.03371 | w=0.16823 1.00000 564.68827 | 247 A1.000** 248 P1.000** 249 F1.000** 250 H1.000** 252 W1.000** 253 T0.999** 254 P1.000** 255 D1.000** 256 V1.000** 257 Y0.991** 258 E1.000** 259 G0.997** |
|  | model3 | -2098.553324 | 17 | p=0.00000 0.96629 0.03371 | w=0.00000 0.16825 564.70409 |  |
|  | model7 | -2144.834232 | 14 | p=0.00500 q=0.02014 |  |  |
|  | model8 | -2098.615049 | 16 | p0=0.96299 p=0.00500 q=2.10758 | (p1=0.03701) w = 580.01461 | 80 K1.000** 149 F1.000** 247 A1.000** 248 P1.000** 249 F1.000** 250 H1.000 **251 Q1.000** 252 W1.000** 253 T1.000** 254 P1.000** 255 D1.000 **256 V1.000** 257 Y1.000** 258 E1.000** 259 G1.000**463 P1.000 **468 N1.000** |
| *ndh*D | model0 | -1262.915336 | 13 | omega (dN/dS) = 0.28244 |  |  |
|  | model1 | -1257.300439 | 14 | p=0.81795 0.18205 | w=0.00000 1.00000 |  |
|  | model2 | -1254.034124 | 16 | p=0.93646 0.00000 0.06354 | w=0.00000 1.00000 5.15206 | 12 I1.000** 23 V1.000** 28 R1.000** 51 V1.000** 61 P1.000** 70 Q1.000** 79 W1.000** 174 G1.000** 177 I1.000** 208 A1.000** |
|  | model3 | -1254.034124 | 17 | p=0.01517 0.92130 0.06353 | w=0.00000 0.00000 5.15213 |  |
|  | model7 | -1257.331857 | 14 | p=0.00500 q=0.02037 |  |  |
|  | model8 | -1254.034124 | 16 | p0=0.93647 p=0.00500 q=1.94267 | (p1=0.06353) w=5.15206 | 12 I1.000** 23 V1.000** 28 R1.000** 51 V1.000** 61 P1.000** 70 Q1.000** 79 W1.000** 174 G1.000** 177 I1.000** 208 A1.000** |
| *ndh*E | model0 | -461.916618 | 13 | omega (dN/dS) = 0.13744 |  |  |
|  | model1 | -461.916639 | 14 | p=0.99999 0.00001 | w=0.13743 1.00000 |  |
|  | model2 | -461.916618 | 16 | p=1.00000 0.00000 0.00000 | w=0.13744 1.00000 1.00000 |  |
|  | model3 | -461.916618 | 17 | p=0.40369 0.43174 0.16457 | w=0.13744 0.13744 0.13744 |  |
|  | model7 | -461.91965 | 14 | p = 15.83932 q = 99.00000 |  |  |
|  | model8 | -461.920042 | 16 | p0=0.99999 p = 15.84661 q = 99.00000 | (p1=0.00001) w=6.25182 |  |
| *ndh*H | model0 | -884.161606 | 13 | omega (dN/dS) = 0.21509 |  |  |
|  | model1 | -884.161617 | 14 | p=0.99999 0.00001 | w=0.21509 1.00000 |  |
|  | model2 | -884.161606 | 16 | p=1.00000 0.00000 0.00000 | w=0.21509 1.00000 1.00000 |  |
|  | model3 | -884.161606 | 17 | p=0.43196 0.40376 0.16427 | w=0.21509 0.21509 0.21509 |  |
|  | model7 | -884.164058 | 14 | p = 27.19781 q = 99.00000 |  |  |
|  | model8 | -884.164069 | 16 | p0=0.99999 p = 27.19708 q = 99.00000 | (p1=0.00001) w=1.00000 |  |
| *ndh*I | model0 | -246.056761 | 13 | omega (dN/dS) = 0.55973 |  |  |
|  | model1 | -245.165924 | 14 | p=0.68785 0.31215 | w=0.00000 1.00000 |  |
|  | model2 | -243.728979 | 16 | p=0.91763 0.00000 0.08237 | w=0.00000 1.00000 11.97154 |  |
|  | model3 | -243.728979 | 17 | p=0.01444 0.90319 0.08237 | w=0.00000 0.00000 11.97156 |  |
|  | model7 | -245.167287 | 14 | p=0.00500 q=0.01173 |  |  |
|  | model8 | -243.728978 | 16 | p0=0.91763 p=0.00500 q = 24.80388 | (p1=0.08237) w = 11.97143 |  |
| *ndh*J | model0 | -719.3862 | 13 | omega (dN/dS) = 0.13516 |  |  |
|  | model1 | -717.638291 | 14 | p=0.88249 0.11751 | w=0.00000 1.00000 |  |
|  | model2 | -717.415186 | 16 | p=0.93233 0.00000 0.06767 | w=0.00000 1.00000 2.11563 |  |
|  | model3 | -717.415186 | 17 | p=0.54772 0.38460 0.06767 | w=0.00000 0.00000 2.11564 |  |
|  | model7 | -717.677661 | 14 | p=0.00500 q=0.04688 |  |  |
|  | model8 | -717.415182 | 16 | p0=0.93233 p=0.00500 q = 23.65641 | (p1=0.06767) w=2.11573 |  |
| *pet*A | model0 | -1449.821973 | 13 | omega (dN/dS) = 0.20325 |  |  |
|  | model1 | -1448.142945 | 14 | p=0.80908 0.19092 | w=0.00000 1.00000 |  |
|  | model2 | -1447.400468 | 16 | p=0.92615 0.00000 0.07385 | w=0.00000 1.00000 3.48282 |  |
|  | model3 | -1447.400468 | 17 | p=0.66185 0.26430 0.07385 | w=0.00000 0.00000 3.48287 |  |
|  | model7 | -1448.150022 | 14 | p=0.00500 q=0.02044 |  |  |
|  | model8 | -1447.40047 | 16 | p0=0.92615 p=0.00500 q=1.40438 | (p1=0.07385) w=3.48282 |  |
| *pet*B | model0 | -998.659976 | 13 | omega (dN/dS) = 0.08616 |  |  |
|  | model1 | -996.655641 | 14 | p=0.92021 0.07979 | w=0.00000 1.00000 |  |
|  | model2 | -995.905778 | 16 | p=0.99482 0.00000 0.00518 | w=0.05154 1.00000 15.09041 |  |
|  | model3 | -995.905778 | 17 | p=0.00006 0.99476 0.00518 | w=0.05151 0.05154 15.09068 |  |
|  | model7 | -996.733407 | 14 | p=0.00865 q=0.12318 |  |  |
|  | model8 | -995.908529 | 16 | p0=0.99483 p=5.42877 q = 99.00000 | (p1=0.00517) w = 15.09177 |  |
| *pet*D | model0 | -692.4427 | 13 | omega (dN/dS) = 0.03753 |  |  |
|  | model1 | -692.442702 | 14 | p=0.99999 0.00001 | w=0.03752 1.00000 |  |
|  | model2 | -692.4427 | 16 | p=1.00000 0.00000 0.00000 | w=0.03753 1.00000 1.00000 |  |
|  | model3 | -692.4427 | 17 | p=0.16484 0.43086 0.40430 | w=0.03749 0.03753 0.03754 |  |
|  | model7 | -692.442742 | 14 | p=3.89973 q = 99.00000 |  |  |
|  | model8 | -692.442744 | 16 | p0=0.99999 p=3.89885 q = 99.00000 | (p1=0.00001) w=1.00000 |  |
| *pet*G | model0 | -145.068231 | 13 | omega (dN/dS) = 999.00000 |  |  |
|  | model1 | -145.524389 | 14 | p=0.00001 0.99999 | w=1.00000 1.00000 |  |
|  | model2 | -145.068231 | 16 | p=0.00000 0.00000 1.00000 | w=0.00000 1.00000 999.00000 |  |
|  | model3 | -145.068259 | 17 | p=0.00000 0.00000 1.00000 | w: 500.22298 951.34909 999.00000 |  |
|  | model7 | -145.524503 | 14 | p=1.09540 q=0.00500 |  |  |
|  | model8 | -145.068372 | 16 | p0=0.00001 p=1.77281 q=0.00500 | (p1=0.99999) w = 953.72620 |  |
| *pet*L | model0 | -124.599192 | 13 | omega (dN/dS) = 0.23546 |  |  |
|  | model1 | -124.582179 | 14 | p=0.75252 0.24748 | w=0.00000 1.00000 |  |
|  | model2 | -124.522031 | 16 | p=0.94397 0.00000 0.05603 | w=0.00000 1.00000 7.65033 |  |
|  | model3 | -124.522051 | 17 | p=0.94397 0.00000 0.05603 | w=0.00000 1.34778 7.65064 |  |
|  | model7 | -124.586022 | 14 | p=0.02994 q=0.09013 |  |  |
|  | model8 | -124.522137 | 16 | p0=0.94397 p=0.00500 q=1.87243 | (p1=0.05603) w=7.65168 |  |
| *pet*N | model0 | -126.505754 | 13 | omega (dN/dS) = 0.08708 |  |  |
|  | model1 | -126.505779 | 14 | p=0.99999 0.00001 | w=0.08707 1.00000 |  |
|  | model2 | -126.505754 | 16 | p=1.00000 0.00000 0.00000 | w=0.08708 1.00000 1.00000 |  |
|  | model3 | -126.505754 | 17 | p=0.41103 0.16373 0.42525 | w=0.08708 0.08708 0.08708 |  |
|  | model7 | -126.508593 | 14 | p=9.51782 q = 99.00000 |  |  |
|  | model8 | -126.509254 | 16 | p0=0.99999 p=9.52077 q = 99.00000 | (p1=0.00001) w=9.98625 |  |
| *psa*A | model0 | -3379.61351 | 13 | omega (dN/dS) = 0.09580 |  |  |
|  | model1 | -3375.382375 | 14 | p=0.91704 0.08296 | w=0.00000 1.00000 |  |
|  | model2 | -3374.715058 | 16 | p=0.96192 0.00000 0.03808 | w=0.00000 1.00000 2.55528 |  |
|  | model3 | -3374.715058 | 17 | p=0.96025 0.00167 0.03808 | w=0.00000 0.00000 2.55528 |  |
|  | model7 | -3375.519821 | 14 | p=0.00663 q=0.08654 |  |  |
|  | model8 | -3374.715061 | 16 | p0=0.96192 p=0.00500 q=0.99298 | (p1=0.03808) w=2.55541 |  |
| *psa*B | model0 | -3219.010897 | 13 | omega (dN/dS) = 0.10768 |  |  |
|  | model1 | -3217.127418 | 14 | p=0.89977 0.10023 | w=0.00000 1.00000 |  |
|  | model2 | -3216.903686 | 16 | p=0.94329 0.01721 0.03950 | w=0.00000 1.00000 2.34904 |  |
|  | model3 | -3216.903302 | 17 | p=0.54100 0.40703 0.05197 | w=0.00000 0.00000 2.11512 |  |
|  | model7 | -3217.127432 | 14 | p=0.00500 q=0.04798 |  |  |
|  | model8 | -3216.903302 | 16 | p0=0.94804 p=0.00500 q=0.72752 | (p1=0.05196) w=2.11524 |  |
| *psa*C | model0 | -338.477427 | 13 | omega (dN/dS) = 0.00010 |  |  |
|  | model1 | -338.476402 | 14 | p=0.99999 0.00001 | w=0.00000 1.00000 |  |
|  | model2 | -338.476273 | 16 | p=1.00000 0.00000 0.00000 | w=0.00000 1.00000 1.00000 |  |
|  | model3 | -338.476273 | 17 | p=0.40400 0.16568 0.43032 | w=0.00000 0.00000 0.00000 |  |
|  | model7 | -338.47597 | 14 | p=0.00500 q = 99.00000 |  |  |
|  | model8 | -338.476099 | 16 | p0=0.99999 p=0.00500 q = 99.00000 | (p1=0.00001) w=1.00000 |  |
| *psa*I | model0 | -146.818049 | 13 | omega (dN/dS) = 0.36102 |  |  |
|  | model1 | -146.818189 | 14 | p=0.99999 0.00001 | w=0.36101 1.00000 |  |
|  | model2 | -146.818049 | 16 | p=1.00000 0.00000 0.00000 | w=0.36102 1.00000 1.00000 |  |
|  | model3 | -146.818112 | 17 | p=0.16441 0.41511 0.42048 | w=0.36102 0.36102 0.36102 |  |
|  | model7 | -146.818298 | 14 | p = 56.03531 q = 99.00000 |  |  |
|  | model8 | -146.818578 | 16 | p0=0.99999 p = 56.07034 q = 99.00000 | (p1=0.00001) w = 45.00683 |  |
| *psa*J | model0 | -192.548715 | 13 | omega (dN/dS) = 0.14817 |  |  |
|  | model1 | -192.548716 | 14 | p=0.99999 0.00001 | w=0.14817 1.00000 |  |
|  | model2 | -192.548715 | 16 | p=1.00000 0.00000 0.00000 | w=0.14817 1.00000 1.00000 |  |
|  | model3 | -192.548715 | 17 | p=0.40402 0.16458 0.43140 | w=0.14817 0.14817 0.14817 |  |
|  | model7 | -192.54884 | 14 | p = 17.26900 q = 99.00000 |  |  |
|  | model8 | -192.548841 | 16 | p0=0.99999 p = 17.26797 q = 99.00000 | (p1=0.00001) w=1.00000 |  |
| *psb*A | model0 | -1559.14332 | 13 | omega (dN/dS) = 0.02739 |  |  |
|  | model1 | -1559.143387 | 14 | p=0.99999 0.00001 | w=0.02738 1.00000 |  |
|  | model2 | -1559.14332 | 16 | p=1.00000 0.00000 0.00000 | w=0.02739 1.00000 1.00000 |  |
|  | model3 | -1559.14332 | 17 | p=0.00000 0.00095 0.99905 | w=0.00000 0.02677 0.02739 |  |
|  | model7 | -1559.145204 | 14 | p=2.82728 q = 99.00000 |  |  |
|  | model8 | -1559.145274 | 16 | p0=0.99999 p=2.82641 q = 99.00000 | (p1=0.00001) w=1.00000 |  |
| *psb*B | model0 | -2247.334069 | 13 | omega (dN/dS) = 0.04759 |  |  |
|  | model1 | -2247.334113 | 14 | p=0.99999 0.00001 | w=0.04758 1.00000 |  |
|  | model2 | -2247.334069 | 16 | p=1.00000 0.00000 0.00000 | w=0.04759 1.00000 1.00000 |  |
|  | model3 | -2247.334069 | 17 | p=0.15077 0.51651 0.33272 | w=0.04759 0.04759 0.04759 |  |
|  | model7 | -2247.336104 | 14 | p=4.98804 q = 99.00000 |  |  |
|  | model8 | -2247.336148 | 16 | p0=0.99999 p=4.98724 q = 99.00000 | (p1=0.00001) w=1.00000 |  |
| *psb*C | model0 | -2052.175597 | 13 | omega (dN/dS) = 0.06649 |  |  |
|  | model1 | -2052.175613 | 14 | p=0.99999 0.00001 | w=0.06648 1.00000 |  |
|  | model2 | -2052.175597 | 16 | p=1.00000 0.00000 0.00000 | w=0.06649 1.00000 1.00000 |  |
|  | model3 | -2052.175597 | 17 | p=0.43134 0.16479 0.40387 | w=0.06648 0.06649 0.06649 |  |
|  | model7 | -2052.1766 | 14 | p=7.09125 q = 99.00000 |  |  |
|  | model8 | -2052.176625 | 16 | p0=0.99999 p=7.07371 q = 99.00000 | (p1=0.00001) w=1.00000 |  |
| *psb*D | model0 | -1499.202582 | 13 | omega (dN/dS) = 0.05062 |  |  |
|  | model1 | -1499.202607 | 14 | p=0.99999 0.00001 | w=0.05061 1.00000 |  |
|  | model2 | -1499.202582 | 16 | p=1.00000 0.00000 0.00000 | w=0.05062 1.00000 1.00000 |  |
|  | model3 | -1499.202582 | 17 | p=0.43128 0.40392 0.16480 | w=0.05062 0.05062 0.05063 |  |
|  | model7 | -1499.203768 | 14 | p=5.31876 q = 99.00000 |  |  |
|  | model8 | -1499.203792 | 16 | p0=0.99999 p=5.31802 q = 99.00000 | (p1=0.00001) w=1.00000 |  |
| *psb*E | model0 | -345.164396 | 13 | omega (dN/dS) = 0.00010 |  |  |
|  | model1 | -345.163813 | 14 | p=0.99999 0.00001 | w=0.00000 1.00000 |  |
|  | model2 | -345.163804 | 16 | p=1.00000 0.00000 0.00000 | w=0.00000 1.00000 1.00000 |  |
|  | model3 | -345.163735 | 17 | p=0.42972 0.40424 0.16604 | w=0.00000 0.00000 0.00000 |  |
|  | model7 | -345.163563 | 14 | p=0.00500 q = 99.00000 |  |  |
|  | model8 | -345.16364 | 16 | p0=0.99999 p=0.00500 q = 99.00000 | (p1=0.00001) w=1.00000 |  |
| *psb*F | model0 | -167.508324 | 13 | omega (dN/dS) = 0.35131 |  |  |
|  | model1 | -167.508373 | 14 | p=0.99999 0.00001 | w=0.35130 1.00000 |  |
|  | model2 | -167.508356 | 16 | p=1.00000 0.00000 0.00000 | w=0.35131 1.00000 1.00000 |  |
|  | model3 | -167.508365 | 17 | p=0.40434 0.43216 0.16350 | w=0.35131 0.35131 0.35131 |  |
|  | model7 | -167.508502 | 14 | p = 53.68805 q = 99.00000 |  |  |
|  | model8 | -167.508678 | 16 | p0=0.99999 p = 53.68580 q = 99.00000 | (p1=0.00001) w=1.00000 |  |
| *psb*H | model0 | -307.744358 | 13 | omega (dN/dS) = 0.34616 |  |  |
|  | model1 | -306.455927 | 14 | p=0.81865 0.18135 | w=0.00000 1.00000 |  |
|  | model2 | -305.569583 | 16 | p=0.94636 0.00000 0.05364 | w=0.00000 1.00000 6.50045 |  |
|  | model3 | -305.569583 | 17 | p=0.92371 0.02265 0.05364 | w=0.00000 0.00000 6.50043 |  |
|  | model7 | -306.462216 | 14 | p=0.00500 q=0.02035 |  |  |
|  | model8 | -305.569638 | 16 | p0=0.94636 p=0.00500 q = 98.99981 | (p1=0.05364) w=6.50037 |  |
| *psb*I | model0 | -146.134506 | 13 | omega (dN/dS) = 999.00000 |  |  |
|  | model1 | -146.580939 | 14 | p=0.50237 0.49763 | w=1.00000 1.00000 |  |
|  | model2 | -146.134509 | 16 | p=0.00000 0.00000 1.00000 | w=0.03969 1.00000 999.00000 |  |
|  | model3 | -146.134552 | 17 | p=0.00000 0.00000 1.00000 | w: 497.73675 954.21181 960.08072 |  |
|  | model7 | -146.58086 | 14 | p=2.46323 q=0.00500 |  |  |
|  | model8 | -146.134562 | 16 | p0=0.00001 p=1.96724 q=0.00500 | (p1=0.99999) w = 951.52678 |  |
| *psb*J | model0 | -174.717477 | 13 | omega (dN/dS) = 0.00010 |  |  |
|  | model1 | -174.716853 | 14 | p=0.99999 0.00001 | w=0.00000 1.00000 |  |
|  | model2 | -174.716769 | 16 | p=1.00000 0.00000 0.00000 | w=0.00000 1.00000 1.00000 |  |
|  | model3 | -174.716769 | 17 | p=0.43082 0.40432 0.16486 | w=0.00000 0.00000 0.00000 |  |
|  | model7 | -174.716479 | 14 | p=0.00500 q = 99.00000 |  |  |
|  | model8 | -174.716928 | 16 | p0=0.99999 p=0.00500 q=1.68008 | (p1=0.00001) w=2.20865 |  |
| *psb*K | model0 | -286.066204 | 13 | omega (dN/dS) = 0.11317 |  |  |
|  | model1 | -286.066206 | 14 | p=0.99999 0.00001 | w=0.11316 1.00000 |  |
|  | model2 | -286.066204 | 16 | p=1.00000 0.00000 0.00000 | w=0.11317 1.00000 1.00000 |  |
|  | model3 | -286.064662 | 17 | p=0.56642 0.28663 0.14695 | w=0.00000 0.27241 0.27241 |  |
|  | model7 | -286.065585 | 14 | p=0.84188 q=6.24205 |  |  |
|  | model8 | -286.06559 | 16 | p0=0.99999 p=0.84178 q=6.24172 | (p1=0.00001) w=1.00000 |  |
| *psb*L | model0 | -145.269576 | 13 | omega (dN/dS) = 2.00000 |  |  |
|  | model1 | -145.269562 | 14 | p=0.53439 0.46561 | w=0.19311 1.00000 |  |
|  | model2 | -145.269523 | 16 | p=1.00000 0.00000 0.00000 | w=0.00000 1.00000 1.00000 |  |
|  | model3 | -145.269472 | 17 | p=0.00000 0.00000 1.00000 | w=0.00000 35.97818 170.83727 |  |
|  | model7 | -145.269628 | 14 | p=0.30726 q=1.29298 |  |  |
|  | model8 | -145.269553 | 16 | p0=0.90000 p=1.09826 q=1.02553 | (p1=0.10000) w=2.00000 |  |
| *psb*M | model0 | -155.052115 | 13 | omega (dN/dS) = 0.08223 |  |  |
|  | model1 | -155.052144 | 14 | p=0.99999 0.00001 | w=0.08223 1.00000 |  |
|  | model2 | -155.052115 | 16 | p=1.00000 0.00000 0.00000 | w=0.08223 1.00000 999.00000 |  |
|  | model3 | -155.052115 | 17 | p=0.16478 0.43085 0.40437 | w=0.08223 0.08223 0.08223 |  |
|  | model7 | -155.055221 | 14 | p=8.93541 q = 99.00000 |  |  |
|  | model8 | -155.055359 | 16 | p0=0.99999 p=8.94024 q = 99.00000 | (p1=0.00001) w=2.92946 |  |
| *psb*N | model0 | -175.50628 | 13 | omega (dN/dS) = 0.00010 |  |  |
|  | model1 | -175.506138 | 14 | p=0.99999 0.00001 | w=0.00000 1.00000 |  |
|  | model2 | -175.506116 | 16 | p=1.00000 0.00000 0.00000 | w=0.00000 1.00000 1.00000 |  |
|  | model3 | -175.506116 | 17 | p=0.43400 0.40048 0.16552 | w=0.00000 0.00000 0.00000 |  |
|  | model7 | -175.50606 | 14 | p=0.00500 q = 99.00000 |  |  |
|  | model8 | -175.506075 | 16 | p0=0.99999 p=0.00500 q = 49.43691 | (p1=0.00001) w=1.00000 |  |
| *psb*T | model0 | -134.144189 | 13 | omega (dN/dS) = 999.00000 |  |  |
|  | model1 | -134.419916 | 14 | p=0.00001 0.99999 | w=0.49765 1.00000 |  |
|  | model2 | -134.144189 | 16 | p=0.00000 0.00000 1.00000 | w=0.00000 1.00000 999.00000 |  |
|  | model3 | -134.144342 | 17 | p=0.00000 1.00000 0.00000 | w: 500.24528 951.17307 951.33284 |  |
|  | model7 | -134.419915 | 14 | p=3.10894 q=0.00500 |  |  |
|  | model8 | -134.391273 | 16 | p0=0.88845 p=0.21761 q=1.28472 | (p1=0.11155) w = 951.43299 |  |
| *psb*Z | model0 | -248.207449 | 13 | omega (dN/dS) = 0.16643 |  |  |
|  | model1 | -248.207701 | 14 | p=0.99999 0.00001 | w=0.16642 1.00000 |  |
|  | model2 | -248.2077 | 16 | p=1.00000 0.00000 0.00000 | w=0.16643 1.00000 1.00000 |  |
|  | model3 | -248.207449 | 17 | p=0.43120 0.40426 0.16454 | w=0.16643 0.16643 0.16643 |  |
|  | model7 | -248.207578 | 14 | p = 19.81257 q = 99.00000 |  |  |
|  | model8 | -248.207579 | 16 | p0=0.99999 p = 19.81158 q = 99.00000 | (p1=0.00001) w=1.00000 |  |
| *rbc*L | model0 | -2207.462597 | 13 | omega (dN/dS) = 0.20234 |  |  |
|  | model1 | -2201.046179 | 14 | p=0.85288 0.14712 | w=0.00000 1.00000 |  |
|  | model2 | -2198.579539 | 16 | p=0.94566 0.00000 0.05434 | w=0.00000 1.00000 3.94946 | 30 T 1.000** 149 T 1.000** 233 Y 1.000** 237 A 1.000** 247 L 1.000** 258 M 1.000** 333 I 1.000** 335 A 1.000** 468 V 1.000** 475 E 1.000** 477 E 1.000** 484 I 1.000** |
|  | model3 | -2198.579539 | 17 | p=0.63191 0.31375 0.05434 | w=0.00000 0.00000 3.94948 | 30 T 1.000** 149 T 1.000** 233 Y 1.000** 237 A 1.000** 247 L 1.000** 258 M 1.000** 333 I 1.000** 335 A 1.000** 468 V 1.000** 475 E 1.000** 477 E 1.000** 484 I 1.000** |
|  | model7 | -2201.47692 | 14 | p=0.00500 q=0.02082 |  |  |
|  | model8 | -2198.579542 | 16 | p0=0.94566 p=0.00500 q=1.52580 | (p1=0.05434) w=3.94949 | 30 T 1.000** 149 T 1.000** 233 Y 1.000** 237 A 1.000** 247 L 1.000** 258 M 1.000** 333 I 1.000** 335 A 1.000** 468 V 1.000** 475 E 1.000** 477 E 1.000** 484 I 1.000** |
| *rpl*2 | model0 | -1104.530049 | 13 | omega (dN/dS) = 0.18960 |  |  |
|  | model1 | -1104.530065 | 14 | p=0.99999 0.00001 | w=0.18959 1.00000 |  |
|  | model2 | -1104.530064 | 16 | p=1.00000 0.00000 0.00000 | w=0.18960 1.00000 1.00000 |  |
|  | model3 | -1104.530725 | 17 | p=0.16445 0.43143 0.40411 | w=0.18956 0.18959 0.18964 |  |
|  | model7 | -1104.530216 | 14 | p = 23.56188 q = 99.00000 |  |  |
|  | model8 | -1104.530765 | 16 | p0=0.99999 p = 23.85855 q = 99.00000 | (p1=0.00001) w=2.32026 |  |
| *rpl*14 | model0 | -525.258268 | 13 | omega (dN/dS) = 0.03573 |  |  |
|  | model1 | -525.258305 | 14 | p=0.99999 0.00001 | w=0.03573 1.00000 |  |
|  | model2 | -525.258268 | 16 | p=1.00000 0.00000 0.00000 | w=0.03573 1.00000 1.00000 |  |
|  | model3 | -525.258268 | 17 | p=0.16485 0.40439 0.43076 | w=0.03573 0.03573 0.03573 |  |
|  | model7 | -525.259674 | 14 | p=3.71042 q = 99.00000 |  |  |
|  | model8 | -525.259712 | 16 | p0=0.99999 p=3.70969 q = 99.00000 | (p1=0.00001) w=1.00000 |  |
| *rpl*16 | model0 | -586.138166 | 13 | omega (dN/dS) = 0.08161 |  |  |
|  | model1 | -586.138057 | 14 | p=0.99999 0.00001 | w=0.08160 1.00000 |  |
|  | model2 | -586.138051 | 16 | p=1.00000 0.00000 0.00000 | w=0.08161 1.00000 1.00000 |  |
|  | model3 | -586.138051 | 17 | p=0.43093 0.40429 0.16479 | w=0.08161 0.08161 0.08161 |  |
|  | model7 | -586.138574 | 14 | p=8.84232 q = 99.00000 |  |  |
|  | model8 | -586.13858 | 16 | p0=0.99999 p=8.84136 q = 99.00000 | (p1=0.00001) w=1.00000 |  |
| *rpl*20 | model0 | -557.349289 | 13 | omega (dN/dS) = 0.25065 |  |  |
|  | model1 | -556.369662 | 14 | p=0.77187 0.22813 | w=0.00000 1.00000 |  |
|  | model2 | -556.295599 | 16 | p=0.83547 0.00000 0.16453 | w=0.00000 1.00000 1.52585 |  |
|  | model3 | -556.295599 | 17 | p=0.51145 0.32402 0.16453 | w=0.00000 0.00000 1.52585 |  |
|  | model7 | -556.400727 | 14 | p=0.00590 q=0.01951 |  |  |
|  | model8 | -556.295598 | 16 | p0=0.83547 p=0.00500 q = 33.39484 | (p1=0.16453) w=1.52584 |  |
| *rpl*22 | model0 | -608.494629 | 13 | omega (dN/dS) = 0.29276 |  |  |
|  | model1 | -607.479645 | 14 | p=0.68409 0.31591 | w=0.00000 1.00000 |  |
|  | model2 | -607.479645 | 16 | p=0.68409 0.23638 0.07954 | w=0.00000 1.00000 1.00000 |  |
|  | model3 | -607.471064 | 17 | p=0.66390 0.00068 0.33543 | w=0.00000 0.90724 0.90726 |  |
|  | model7 | -607.487114 | 14 | p=0.02481 q=0.05530 |  |  |
|  | model8 | -607.482426 | 16 | p0=0.98490 p=0.00506 q=0.01431 | (p1=0.01510) w=1.00000 |  |
| *rpl*23 | model0 | -394.169843 | 13 | omega (dN/dS) = 999.00000 |  |  |
|  | model1 | -395.072588 | 14 | p=0.00001 0.99999 | w=1.00000 1.00000 |  |
|  | model2 | -394.169893 | 16 | p=0.00000 0.00000 1.00000 | w=0.00000 1.00000 950.35376 |  |
|  | model3 | -394.169891 | 17 | p=0.00000 0.99944 0.00056 | w: 499.56551 951.40598 951.42058 |  |
|  | model7 | -395.072592 | 14 | p=0.91945 q=0.00500 |  |  |
|  | model8 | -394.170015 | 16 | p0=0.00001 p=1.06131 q=1.05852 | (p1=0.99999) w = 951.42931 |  |
| *rpl*32 | model0 | -228.001125 | 13 | omega (dN/dS) = 0.07663 |  |  |
|  | model1 | -228.001134 | 14 | p=0.99999 0.00001 | w=0.07663 1.00000 |  |
|  | model2 | -228.001125 | 16 | p=1.00000 0.00000 0.00000 | w=0.07663 1.00000 1.00000 |  |
|  | model3 | -228.001154 | 17 | p=0.16477 0.40415 0.43108 | w=0.07663 0.07663 0.07663 |  |
|  | model7 | -228.001777 | 14 | p=8.26479 q = 99.00000 |  |  |
|  | model8 | -228.001785 | 16 | p0=0.99999 p=8.26398 q = 99.00000 | (p1=0.00001) w=1.00000 |  |
| *rpl*33 | model0 | -300.131438 | 13 | omega (dN/dS) = 0.09179 |  |  |
|  | model1 | -300.131441 | 14 | p=0.99999 0.00001 | w=0.09178 1.00000 |  |
|  | model2 | -300.131439 | 16 | p=1.00000 0.00000 0.00000 | w=0.09179 1.00000 1.00000 |  |
|  | model3 | -300.131438 | 17 | p=0.16484 0.40535 0.42981 | w=0.09179 0.09179 0.09179 |  |
|  | model7 | -300.131646 | 14 | p = 10.05784 q = 99.00000 |  |  |
|  | model8 | -300.131649 | 16 | p0=0.99999 p = 10.05696 q = 99.00000 | (p1=0.00001) w=1.00000 |  |
| *rpl*36 | model0 | -161.399114 | 13 | omega (dN/dS) = 0.00010 |  |  |
|  | model1 | -161.398488 | 14 | p=0.99999 0.00001 | w=0.00000 1.00000 |  |
|  | model2 | -161.398406 | 16 | p=1.00000 0.00000 0.00000 | w=0.00000 1.00000 1.00000 |  |
|  | model3 | -161.398406 | 17 | p=0.40397 0.16575 0.43028 | w=0.00000 0.00000 0.00000 |  |
|  | model7 | -161.398144 | 14 | p=0.00500 q = 99.00000 |  |  |
|  | model8 | -161.39853 | 16 | p0=0.99999 p=0.00500 q=1.48457 | (p1=0.00001) w=1.53415 |  |
| *rpo*A | model0 | -1638.347847 | 13 | omega (dN/dS) = 0.39525 |  |  |
|  | model1 | -1638.268145 | 14 | p=0.79598 0.20402 | w=0.24710 1.00000 |  |
|  | model2 | -1638.268145 | 16 | p=0.79598 0.08442 0.11960 | w=0.24710 1.00000 1.00000 |  |
|  | model3 | -1638.259014 | 17 | p=0.34695 0.16782 0.48523 | w=0.00000 0.61218 0.61230 |  |
|  | model7 | -1638.26306 | 14 | p=0.68747 q=1.02824 |  |  |
|  | model8 | -1638.26306 | 16 | p0=0.99999 p=0.68747 q=1.02827 | (p1=0.00001) w=1.00000 |  |
| *rpo*B | model0 | -4795.914094 | 13 | omega (dN/dS) = 0.13413 |  |  |
|  | model1 | -4795.914174 | 14 | p=0.99999 0.00001 | w=0.13413 1.00000 |  |
|  | model2 | -4795.914094 | 16 | p=1.00000 0.00000 0.00000 | w=0.13414 1.00000 1.00000 |  |
|  | model3 | -4795.914094 | 17 | p=0.43509 0.16424 0.40067 | w=0.13412 0.13414 0.13415 |  |
|  | model7 | -4795.924587 | 14 | p = 15.38892 q = 99.00000 |  |  |
|  | model8 | -4795.924666 | 16 | p0=0.99999 p = 15.39109 q = 99.00000 | (p1=0.00001) w=1.00000 |  |
| rpoC1 | model0 | -3123.557623 | 13 | omega (dN/dS) = 0.20435 |  |  |
|  | model1 | -3119.52732 | 14 | p=0.81497 0.18503 | w=0.00000 1.00000 |  |
|  | model2 | -3118.733221 | 16 | p=0.90419 0.00000 0.09581 | w=0.00000 1.00000 2.28017 |  |
|  | model3 | -3118.733221 | 17 | p=0.38687 0.51732 0.09581 | w=0.00000 0.00000 2.28015 |  |
|  | model7 | -3119.569288 | 14 | p=0.00500 q=0.02059 |  |  |
|  | model8 | -3118.733223 | 16 | p0=0.90419 p=0.00500 q=1.19906 | (p1=0.09581) w=2.28022 |  |
| rpoC2 | model0 | -6428.392735 | 13 | omega (dN/dS) = 0.42753 |  |  |
|  | model1 | -6423.783452 | 14 | p=0.60876 0.39124 | w=0.00000 1.00000 |  |
|  | model2 | -6420.33339 | 16 | p=0.59819 0.39915 0.00266 | w=0.00000 1.00000 26.07187 | 1378 S0.987* |
|  | model3 | -6420.232115 | 17 | p=0.68366 0.31444 0.00190 | w=0.00000 1.33459 31.26079 |  |
|  | model7 | -6423.792516 | 14 | p=0.00500 q=0.00776 |  |  |
|  | model8 | -6420.333394 | 16 | p0=0.99734 p=0.00545 q=0.00787 | (p1=0.00266) w = 26.05055 | 1378 S0.987* |
| rps2 | model0 | -1096.339607 | 13 | omega (dN/dS) = 0.16070 |  |  |
|  | model1 | -1096.259923 | 14 | p=0.81978 0.18022 | w=0.00000 1.00000 |  |
|  | model2 | -1096.198592 | 16 | p=0.97900 0.00000 0.02100 | w=0.09887 1.00000 6.12826 |  |
|  | model3 | -1096.198592 | 17 | p=0.07204 0.90696 0.02100 | w=0.09880 0.09887 6.12824 |  |
|  | model7 | -1096.266057 | 14 | p=0.01471 q=0.07412 |  |  |
|  | model8 | -1096.198792 | 16 | p0=0.97905 p = 10.92761 q = 99.00000 | (p1=0.02095) w=6.12570 |  |
| rps3 | model0 | -1046.189807 | 13 | omega (dN/dS) = 0.21438 |  |  |
|  | model1 | -1046.189847 | 14 | p=0.99999 0.00001 | w=0.21437 1.00000 |  |
|  | model2 | -1046.189807 | 16 | p=1.00000 0.00000 0.00000 | w=0.21438 1.00000 1.00000 |  |
|  | model3 | -1046.189807 | 17 | p=0.40560 0.16410 0.43030 | w=0.21437 0.21438 0.21438 |  |
|  | model7 | -1046.198905 | 14 | p = 27.12417 q = 99.00000 |  |  |
|  | model8 | -1046.198945 | 16 | p0=0.99999 p = 27.12357 q = 99.00000 | (p1=0.00001) w=1.00000 |  |
| rps4 | model0 | -881.911376 | 13 | omega (dN/dS) = 0.25656 |  |  |
|  | model1 | -881.91138 | 14 | p=0.99999 0.00001 | w=0.25655 1.00000 |  |
|  | model2 | -881.911376 | 16 | p=1.00000 0.00000 0.00000 | w=0.25656 1.00000 1.00000 |  |
|  | model3 | -881.911376 | 17 | p=0.40391 0.16408 0.43201 | w=0.25656 0.25656 0.25656 |  |
|  | model7 | -881.912291 | 14 | p = 34.22219 q = 99.00000 |  |  |
|  | model8 | -881.913816 | 16 | p0=0.99999 p = 35.33472 q = 99.00000 | (p1=0.00001) w = 19.15637 |  |
| rps7 | model0 | -619.245376 | 13 | omega (dN/dS) = 0.00010 |  |  |
|  | model1 | -619.243508 | 14 | p=0.99999 0.00001 | w=0.00000 1.00000 |  |
|  | model2 | -619.24342 | 16 | p=1.00000 0.00000 0.00000 | w=0.00000 1.00000 1.00000 |  |
|  | model3 | -619.243419 | 17 | p=0.43174 0.40435 0.16391 | w=0.00000 0.00000 0.00000 |  |
|  | model7 | -619.243408 | 14 | p=0.00500 q = 98.55876 |  |  |
|  | model8 | -619.243498 | 16 | p0=0.99999 p=0.00500 q = 83.00195 | (p1=0.00001) w=1.00000 |  |
| rps8 | model0 | -612.251827 | 13 | omega (dN/dS) = 0.19264 |  |  |
|  | model1 | -608.043779 | 14 | p=0.86441 0.13559 | w=0.00000 1.00000 |  |
|  | model2 | -606.546213 | 16 | p=0.93680 0.00000 0.06320 | w=0.00000 1.00000 3.71717 |  |
|  | model3 | -606.546213 | 17 | p=0.22538 0.71142 0.06320 | w=0.00000 0.00000 3.71717 |  |
|  | model7 | -608.220291 | 14 | p=0.00500 q=0.04735 |  |  |
|  | model8 | -606.546204 | 16 | p0=0.93680 p=0.00500 q = 99.00000 | (p1=0.06320) w=3.71715 |  |
| *rps*11 | model0 | -616.070141 | 13 | omega (dN/dS) = 0.01833 |  |  |
|  | model1 | -616.070231 | 14 | p=0.99999 0.00001 | w=0.01832 1.00000 |  |
|  | model2 | -616.070141 | 16 | p=1.00000 0.00000 0.00000 | w=0.01833 1.00000 1.00000 |  |
|  | model3 | -616.070141 | 17 | p=0.16590 0.38428 0.44982 | w=0.01833 0.01833 0.01833 |  |
|  | model7 | -616.07203 | 14 | p=1.88826 q = 99.00000 |  |  |
|  | model8 | -616.072119 | 16 | p0=0.99999 p=1.88763 q = 99.00000 | (p1=0.00001) w=1.00000 |  |
| *rps*12 | model0 | -490.804864 | 13 | omega (dN/dS) = 0.00010 |  |  |
|  | model1 | -490.804682 | 14 | p=0.99999 0.00001 | w=0.00000 1.00000 |  |
|  | model2 | -490.804653 | 16 | p=1.00000 0.00000 0.00000 | w=0.00000 1.00000 12.57879 |  |
|  | model3 | -490.804653 | 17 | p=0.43336 0.40111 0.16553 | w=0.00000 0.00000 0.00000 |  |
|  | model7 | -490.804642 | 14 | p=0.00500 q = 99.00000 |  |  |
|  | model8 | -490.804597 | 16 | p0=0.99999 p=0.00500 q = 99.00000 | (p1=0.00001) w=1.00000 |  |
| *rps*14 | model0 | -435.603382 | 13 | omega (dN/dS) = 0.35038 |  |  |
|  | model1 | -435.603384 | 14 | p=0.99999 0.00001 | w=0.35037 1.00000 |  |
|  | model2 | -435.603382 | 16 | p=1.00000 0.00000 0.00000 | w=0.35038 1.00000 1.00000 |  |
|  | model3 | -435.603382 | 17 | p=0.40544 0.16345 0.43110 | w=0.35037 0.35038 0.35038 |  |
|  | model7 | -435.604013 | 14 | p = 53.48871 q = 99.00000 |  |  |
|  | model8 | -435.604987 | 16 | p0=0.99999 p = 53.48794 q = 99.00000 | (p1=0.00001) w = 819.15944 |  |
| *rps*15 | model0 | -440.680766 | 13 | omega (dN/dS) = 0.68000 |  |  |
|  | model1 | -440.680767 | 14 | p=0.99999 0.00001 | w=0.67999 1.00000 |  |
|  | model2 | -440.680766 | 16 | p=1.00000 0.00000 0.00000 | w=0.68000 1.00000 1.00000 |  |
|  | model3 | -440.680766 | 17 | p=0.15994 0.43567 0.40439 | w=0.67999 0.68000 0.68000 |  |
|  | model7 | -440.681904 | 14 | p = 99.00000 q = 46.46419 |  |  |
|  | model8 | -440.682163 | 16 | p0=0.99999 p = 98.99980 q = 44.44119 | (p1=0.00001) w=1.00000 |  |
| *rps*16 | model0 | -423.339805 | 13 | omega (dN/dS) = 0.72288 |  |  |
|  | model1 | -421.889681 | 14 | p=0.61830 0.38170 | w=0.00000 1.00000 |  |
|  | model2 | -418.773143 | 16 | p=0.87648 0.00000 0.12352 | w=0.00000 1.00000 10.07227 |  |
|  | model3 | -418.773143 | 17 | p=0.51870 0.35778 0.12352 | w=0.00000 0.00000 10.07227 |  |
|  | model7 | -422.028687 | 14 | p=0.00500 q=0.00503 |  |  |
|  | model8 | -418.773142 | 16 | p0=0.87648 p=0.00500 q = 26.25865 | (p1=0.12352) w = 10.07225 |  |
| *rps*18 | model0 | -494.523246 | 13 | omega (dN/dS) = 0.39358 |  |  |
|  | model1 | -493.689352 | 14 | p=0.64337 0.35663 | w=0.00000 1.00000 |  |
|  | model2 | -492.64419 | 16 | p=0.97914 0.00000 0.02086 | w=0.27490 1.00000 15.93878 |  |
|  | model3 | -492.64419 | 17 | p=0.26626 0.71288 0.02086 | w=0.27490 0.27490 15.93876 |  |
|  | model7 | -493.720398 | 14 | p=0.00500 q=0.00746 |  |  |
|  | model8 | -492.645579 | 16 | p0=0.97915 p = 37.62726 q = 99.00000 | (p1=0.02085) w = 15.94739 |  |
| *rps*19 | model0 | -365.005059 | 13 | omega (dN/dS) = 2.00001 |  |  |
|  | model1 | -365.004914 | 14 | p=0.99999 0.00001 | w=0.00000 1.00000 |  |
|  | model2 | -365.005047 | 16 | p=0.57004 0.24607 0.18389 | w=0.41868 1.00000 2.32233 |  |
|  | model3 | -365.005153 | 17 | p=0.27162 0.52679 0.20159 | w=1.00000 2.52506 4.14480 |  |
|  | model7 | -365.004982 | 14 | p=0.28064 q=1.33667 |  |  |
|  | model8 | -365.005036 | 16 | p0=0.90000 p=1.09826 q=1.02553 | (p1=0.10000) w=2.00001 |  |
| *ycf*1 | model0 | -9237.78526 | 13 | omega (dN/dS) = 0.67151 |  |  |
|  | model1 | -9223.245522 | 14 | p=0.47676 0.52324 | w=0.00000 1.00000 |  |
|  | model2 | -9213.870193 | 16 | p=0.46357 0.50236 0.03407 | w=0.00000 1.00000 7.03349 | 1409 F0.989* |
|  | model3 | -9213.661232 | 17 | p=0.88368 0.11471 0.00161 | w=0.32634 3.66555 23.42509 |  |
|  | model7 | -9223.350102 | 14 | p=0.00520 q=0.00503 |  |  |
|  | model8 | -9213.868413 | 16 | p0=0.96582 p=0.02940 q=0.02611 | (p1=0.03418) w=7.07011 | 1409 F0.989* |
| *ycf*2 | model0 | -9835.989855 | 13 | omega (dN/dS) = 1.99164 |  |  |
|  | model1 | -9827.797798 | 14 | p=0.99999 0.00001 | w=0.88722 1.00000 |  |
|  | model2 | -9807.334584 | 16 | p=0.98087 0.00000 0.01913 | w=0.44615 1.00000 27.59849 | 162 N0.959* 481 L0.999** 513 P0.962* 559 I0.993** 560 P0.996** 745 R0.969* 834 K0.964* 2036 L0.971* |
|  | model3 | -9807.334581 | 17 | p=0.09081 0.89006 0.01912 | w=0.40289 0.45064 27.60102 |  |
|  | model7 | -9822.667867 | 14 | p=0.00784 q=0.00802 |  |  |
|  | model8 | -9808.118043 | 16 | p0=0.91824 p=0.00502 q=2.44149 | (p1=0.08176) w = 11.26351 | 481 L0.996** 559 I0.975* 560 P0.983* 745 R0.953* 834 K0.950* 2036 L0.954* |
| *ycf*3 | model0 | -700.17295 | 13 | omega (dN/dS) = 1.02196 |  |  |
|  | model1 | -700.172994 | 14 | p=0.00001 0.99999 | w=0.42228 1.00000 |  |
|  | model2 | -700.172843 | 16 | p=0.00000 0.00047 0.99953 | w=1.00000 1.00000 1.02198 |  |
|  | model3 | -700.172808 | 17 | p=0.16458 0.59433 0.24109 | w=1.02192 1.02197 1.02199 |  |
|  | model7 | -700.172994 | 14 | p=0.75091 q=0.00500 |  |  |
|  | model8 | -700.173203 | 16 | p0=0.00001 p=1.40691 q=0.67935 | (p1=0.99999) w=1.02197 |  |
| *ycf*4 | model0 | -916.921077 | 13 | omega (dN/dS) = 0.26502 |  |  |
|  | model1 | -916.921096 | 14 | p=0.99999 0.00001 | w=0.26502 1.00000 |  |
|  | model2 | -916.921077 | 16 | p=1.00000 0.00000 0.00000 | w=0.26502 1.00000 1.00000 |  |
|  | model3 | -916.921077 | 17 | p=0.40330 0.43274 0.16396 | w=0.26502 0.26502 0.26503 |  |
|  | model7 | -916.926161 | 14 | p = 35.80465 q = 99.00000 |  |  |
|  | model8 | -916.92618 | 16 | p0=0.99999 p = 35.80389 q = 99.00000 | (p1=0.00001) w=1.00000 |  |
