# Supplementary material for: Plastome structure and adaptive evolution of Calanthe s.l. species
Source: PeerJ. 2020 Oct 13;8:e10051. doi: 10.7717/peerj.10051 (PMC7566753; doi:10.7717/peerj.10051)
Supplement: Supplemental Information 9 [file peerj-08-10051-s009.docx]

### **Table S9** Likelihood ratio test (LRT) of the variable ω ratio under different models.

| **Gene** | **M0 vs M3** | | **M1 vs M2** | | **M7 vs M8** | |
| --- | --- | --- | --- | --- | --- | --- |
|  | **2Δl** | **p (df=4)** | **2Δl** | **p (df=2)** | **2Δl** | **p (df=2)** |
| *accD* | 24.754448 | 4.21E-06 | 9.82766 | 7.34E-03 | 9.825084 | 7.35E-03 |
| *atpA* | 3.23911 | 1.98E-01 | 0.291832 | 8.64E-01 | 0.485256 | 7.85E-01 |
| *atpB* | 0 | 1.00E+00 | 8.2E-05 | 1.00E+00 | 0.000202 | 1.00E+00 |
| *atpE* | 0 | 1.00E+00 | 2.2E-05 | 1.00E+00 | 2.2E-05 | 1.00E+00 |
| *atpF* | 0 | 1.00E+00 | 8E-06 | 1.00E+00 | 6E-06 | 1.00E+00 |
| *atpH* | 0.567538 | 7.53E-01 | 0.394374 | 8.21E-01 | 0.47745 | 7.88E-01 |
| *atpI* | 0 | 1.00E+00 | 1.8E-05 | 1.00E+00 | 2.2E-05 | 1.00E+00 |
| *ccsA* | 4.27198 | 1.18E-01 | 0.252648 | 8.81E-01 | 0.33664 | 8.45E-01 |
| *cemA* | 35.098962 | 2.39E-08 | 0 | 1.00E+00 | 2E-06 | 1.00E+00 |
| *clpP* | 0 | 1.00E+00 | 8.6E-05 | 1.00E+00 | 0.002136 | 9.99E-01 |
| *infA* | 10.912114 | 4.27E-03 | 2.613848 | 2.71E-01 | 5.507598 | 6.37E-02 |
| *matK* | 6.899656 | 3.18E-02 | 0.848314 | 6.54E-01 | 0.87706 | 6.45E-01 |
| *nad6* | 0.45555 | 7.96E-01 | 0.49558 | 7.81E-01 | 0.573298 | 7.51E-01 |
| *ndhA* | 2.871662 | 2.38E-01 | 0.38113 | 8.27E-01 | 0.383866 | 8.25E-01 |
| *ndhB* | 109.448464 | 0.00E+00 | 91.762948 | 0.00E+00 | 92.438366 | 0.00E+00 |
| *ndhD* | 17.762424 | 1.39E-04 | 6.53263 | 3.82E-02 | 6.595466 | 3.70E-02 |
| *ndhE* | 0 | 1.00E+00 | 4.2E-05 | 1.00E+00 | 0.000784 | 1.00E+00 |
| *ndhH* | 0 | 1.00E+00 | 2.2E-05 | 1.00E+00 | 2.2E-05 | 1.00E+00 |
| *ndhI* | 4.655564 | 9.75E-02 | 2.87389 | 2.38E-01 | 2.876618 | 2.37E-01 |
| *ndhJ* | 3.942028 | 1.39E-01 | 0.44621 | 8.00E-01 | 0.524958 | 7.69E-01 |
| *petA* | 4.84301 | 8.88E-02 | 1.484954 | 4.76E-01 | 1.499104 | 4.73E-01 |
| *petB* | 5.508396 | 6.37E-02 | 1.499726 | 4.72E-01 | 1.649756 | 4.38E-01 |
| *petD* | 0 | 1.00E+00 | 4E-06 | 1.00E+00 | 4E-06 | 1.00E+00 |
| *petG* | 5.6E-05 | 1.00E+00 | 0.912316 | 6.34E-01 | 0.912262 | 6.34E-01 |
| *petL* | 0.154282 | 9.26E-01 | 0.120296 | 9.42E-01 | 0.12777 | 9.38E-01 |
| *petN* | 0 | 1.00E+00 | 5E-05 | 1.00E+00 | 0.001322 | 9.99E-01 |
| *psaA* | 9.796904 | 7.46E-03 | 1.334634 | 5.13E-01 | 1.60952 | 4.47E-01 |
| *psaB* | 4.21519 | 1.22E-01 | 0.447464 | 8.00E-01 | 0.44826 | 7.99E-01 |
| *psaC* | 0.002308 | 9.99E-01 | 0.000258 | 1.00E+00 | 0.000258 | 1.00E+00 |
| *psaI* | 0.000126 | 1.00E+00 | 0.00028 | 1.00E+00 | 0.00056 | 1.00E+00 |
| *psaJ* | 0 | 1.00E+00 | 2E-06 | 1.00E+00 | 2E-06 | 1.00E+00 |
| *psbA* | 0 | 1.00E+00 | 0.000134 | 1.00E+00 | 0.00014 | 1.00E+00 |
| *psbB* | 0 | 1.00E+00 | 8.8E-05 | 1.00E+00 | 8.8E-05 | 1.00E+00 |
| *psbC* | 0 | 1.00E+00 | 3.2E-05 | 1.00E+00 | 5E-05 | 1.00E+00 |
| *psbD* | 0 | 1.00E+00 | 5E-05 | 1.00E+00 | 4.8E-05 | 1.00E+00 |
| *psbE* | 0.001322 | 9.99E-01 | 1.8E-05 | 1.00E+00 | 0.000154 | 1.00E+00 |
| *psbF* | 8.2E-05 | 1.00E+00 | 3.4E-05 | 1.00E+00 | 0.000352 | 1.00E+00 |
| *psbH* | 4.34955 | 1.14E-01 | 1.772688 | 4.12E-01 | 1.785156 | 4.10E-01 |
| *psbI* | 9.2E-05 | 1.00E+00 | 0.89286 | 6.40E-01 | 0.892596 | 6.40E-01 |
| *psbJ* | 0.001416 | 9.99E-01 | 0.000168 | 1.00E+00 | 0.000898 | 1.00E+00 |
| *psbK* | 0.003084 | 9.99E-01 | 4E-06 | 1.00E+00 | 1E-05 | 1.00E+00 |
| *psbL* | 0.000208 | 1.00E+00 | 7.8E-05 | 1.00E+00 | 0.00015 | 1.00E+00 |
| *psbM* | 0 | 1.00E+00 | 5.8E-05 | 1.00E+00 | 0.000276 | 1.00E+00 |
| *psbN* | 0.000328 | 1.00E+00 | 4.4E-05 | 1.00E+00 | 3E-05 | 1.00E+00 |
| *psbT* | 0.000306 | 1.00E+00 | 0.551454 | 7.59E-01 | 0.057284 | 9.72E-01 |
| *psbZ* | 0 | 1.00E+00 | 2E-06 | 1.00E+00 | 2E-06 | 1.00E+00 |
| *rbcL* | 17.766116 | 1.39E-04 | 4.93328 | 8.49E-02 | 5.794756 | 5.52E-02 |
| *rpl14* | 0.001352 | 9.99E-01 | 2E-06 | 1.00E+00 | 0.001098 | 1.00E+00 |
| *rpl16* | 0 | 1.00E+00 | 7.4E-05 | 1.00E+00 | 7.6E-05 | 1.00E+00 |
| *rpl2* | 0.00023 | 1.00E+00 | 1.2E-05 | 1.00E+00 | 1.2E-05 | 1.00E+00 |
| *rpl20* | 2.10738 | 3.49E-01 | 0.148126 | 9.29E-01 | 0.210258 | 9.00E-01 |
| *rpl22* | 2.04713 | 3.59E-01 | 0 | 1.00E+00 | 0.009376 | 9.95E-01 |
| *rpl23* | 9.6E-05 | 1.00E+00 | 1.80539 | 4.06E-01 | 1.805154 | 4.06E-01 |
| *rpl32* | 5.8E-05 | 1.00E+00 | 1.8E-05 | 1.00E+00 | 1.6E-05 | 1.00E+00 |
| *rpl33* | 0 | 1.00E+00 | 4E-06 | 1.00E+00 | 6E-06 | 1.00E+00 |
| *rpl36* | 0.001416 | 9.99E-01 | 0.000164 | 1.00E+00 | 0.000772 | 1.00E+00 |
| *rpoA* | 0.177666 | 9.15E-01 | 0 | 1.00E+00 | 0 | 1.00E+00 |
| *rpoB* | 0 | 1.00E+00 | 0.00016 | 1.00E+00 | 0.000158 | 1.00E+00 |
| *rpoC1* | 9.648804 | 8.03E-03 | 1.588198 | 4.52E-01 | 1.67213 | 4.33E-01 |
| *rpoC2* | 16.32124 | 2.86E-04 | 6.900124 | 3.17E-02 | 6.918244 | 3.15E-02 |
| *rps11* | 0.28203 | 8.69E-01 | 0.122662 | 9.41E-01 | 0.13453 | 9.35E-01 |
| *rps12* | 0 | 1.00E+00 | 8E-05 | 1.00E+00 | 8E-05 | 1.00E+00 |
| *rps14* | 0 | 1.00E+00 | 8E-06 | 1.00E+00 | 0.00305 | 9.99E-01 |
| *rps15* | 0.003914 | 9.98E-01 | 0.000176 | 1.00E+00 | 0.00018 | 1.00E+00 |
| *rps16* | 11.411228 | 3.33E-03 | 2.995132 | 2.24E-01 | 3.348174 | 1.88E-01 |
| *rps18* | 0 | 1.00E+00 | 0.00018 | 1.00E+00 | 0.000178 | 1.00E+00 |
| *rps19* | 0.000422 | 1.00E+00 | 5.8E-05 | 1.00E+00 | 9E-05 | 1.00E+00 |
| *rps2* | 0 | 1.00E+00 | 4E-06 | 1.00E+00 | 0.001948 | 9.99E-01 |
| *rps3* | 0 | 1.00E+00 | 2E-06 | 1.00E+00 | 0.000518 | 1.00E+00 |
| *rps4* | 9.133324 | 1.04E-02 | 6.233076 | 4.43E-02 | 6.51109 | 3.86E-02 |
| *rps7* | 3.758112 | 1.53E-01 | 2.090324 | 3.52E-01 | 2.149638 | 3.41E-01 |
| *rps8* | 0.000188 | 1.00E+00 | 0.000266 | 1.00E+00 | 0.000108 | 1.00E+00 |
| *ycf1* | 48.248056 | 3.34E-11 | 18.750658 | 8.48E-05 | 18.963378 | 7.62E-05 |
| *ycf2* | 57.310548 | 3.59E-13 | 40.926428 | 1.30E-09 | 29.099648 | 4.80E-07 |
| *ycf3* | 0.000284 | 1.00E+00 | 0.000302 | 1.00E+00 | 0.000418 | 1.00E+00 |
| *ycf4* | 0 | 1.00E+00 | 3.8E-05 | 1.00E+00 | 3.8E-05 | 1.00E+00 |
