# Supplementary material for: Plastome structure and adaptive evolution of Calanthe s.l. species
Source: PeerJ. 2020 Oct 13;8:e10051. doi: 10.7717/peerj.10051 (PMC7566753; doi:10.7717/peerj.10051)
Supplement: Supplemental Information 10 [file peerj-08-10051-s010.docx]

>Preptanthe_rubens

CTCTCACTTTCGTGAGACACGCGAGAAACGATAATAAATCTCGTCGTTAGTCGTTCTACTAAGTATTCATGTGAAAAGCCTTATCTTAATAGTATTTAGACTTAAATGAATCCAACAAGATACAAGATAGCAATCGCCCAAGATCTTGTTCTAAGAACAAGATCTTGGGCGATTGCTATCTTCAAAAATTCATATCATATACTATCATATACTATCATATACATATATACAAAAGTATTATCCATTTATAGATGGAACTTCCACAGAAGCTAGATCTAGAGGGAAGTTGTGTGCATTACGTTCATGCATTACTTCCATACCAAGATTAGCACGATTTATGATATCAGCCCAAGTGTTAATAACACGACCTTGACTGTCAACTACAGATTGGTTAAAATTGAAACCGTTCAGGTTGAACGCCATAGTGCTAATACCCAAAGCAGTGAACCAGATACCCACTACTGGCCAAGCAGCCAGGAAGAAATGTAAGGAACGGGAATTGTTGAAACTAGCATATTGGAAGATCAATCGGCCAAAATAACCATGAGCGGCTACAATATTATAAGTTTCTTCCTCTTGACCGAATCTGTAACCTTCATTAGCAGACTCGTTTTCAGTGGTTTCCCTTATTAAACTAGAAGTTACCAAAGAACCATGCATAGCACTGAATAGGGAGCCGCCGAATACACCAGCTACGCCTAACATGTGAAATGGATGCATAAGAATGTTGTGCTCTGCCTGAAATACAATCATGAAGTTGAAAGTACCAGATATTCCTAAAGGCATACCATCAGAGAAACTTCCTTGACCAATAGGGTAGATCAAGAAAACAGCTGTAGCAGCCGCAACAGGAGCTGAATATGCAACAGCAATCCAAGGGCGCATACCCAGACGGAAACTAAGTTCCCACTCACGACCCATGTAACAAGCTACACCAAGTAAAAAGTGTAGAACAATAAGTTCATAAGGACCGCCATTGTATAACCACTCATCAACAGATGCTGCTTCCCATATTGGGTAAAAATGCAAACCTATAGCTGCGGAAGTAGGAATAATGGCACCTGAGATAATATTGTTTCCATAAAGTAGAGATCCAGAAACAGGTTCACGAATACCATCAATATCGACTGGGGGGGCAGCAATGAAGGCAATGATAAATACAGAAGTTGCGGTCAATAAAGTAGGGATCATCAAAACACCAAACCATCCAATGTAAAGACGGTTTTCAGTACTGGTAATCCAGTTGCAGAAGCGACCCCATAGGCTTGTACTTTCGCGTCTCTCTAAAATTGCAGTCATGGTAAGATCTTGGTTTATTCCATTTTCAAGGACTCCCAAGCACACGTATTAACTATAACTAGAGATGAGATAGATAATGAAAGGCTTGTTATTTAACAGTATAATATGTATTATATGCCAATGTCAACCAATCAAAATGAATATATATAGGAATGACCAAACCTATCAGAACAATTTGGAATAAAATAAAGAGTTCAAATAAAACTAAAAAAAATAAATATTCTTTTTTTTTACTTTGGTATGGGTTGCCCGGGACTCGAACCCGGAACTAGTCGGATGGAGTAGATAATTTTTCCTTGTTGCTATAGAAGAAAAAGATCCCTCCCCAAACCGTGCTTGCATTTTTCATTGCACACGACTTTCCCTATGTATATATCAAAAAGAAAAAATAATTAAATATCAAGAAGAAAGTATAATAATTTTGACTACTTCGTTAATTTCACCACGAATATGATGATATATCATATATATCATCATATATGAGCATTTCAGAATACAAATTCATGAATCTTTTTTTTTCATGAAAATGAAAAAGTCTCATGAACAATCATGAATGATCCACCAGATCATTGATACGGATAATGTCCAAATACCAAATACGTTTTCTATGTAATCCGTGTAAAAGAAAAGGGATTTTTTGGAGGAAGATTAAAGAAAGAGATTGTTCTTCTTCCAAAAAAAATTCTTCTAAGAATCCCGAACCTAATCTTCGCATAAAAGTGCGTACTGTACTTTTATGTTTACGAGCCAAAGTTCTAGCACACGAAAGTCGAAGTATATACTTTATACGATACAAAACCTGTTTCTTTGAGGATCCGCTGTGATAACGACAAAGATTTCTAGATATCCGACAAAATCGATCAAGAATATCAGAATCCGATAAATCGGTCCAGATCGGTTTACTAATAGGATGACCCAATACAGTACAAAATTGAGCTTTCGACAATGATCCAATAAGAGAAATAACTGGGGCTATGGTATCTAATTTCTTAGTCAGAGTATTTATTAGAAATGAATTCTCTAGCATTTGATTTCTTATTACCAAAGGATTTTTTAGTACACTTGAAAAATACCCCAGAAAAGAGAAGGAATAGTTGGGTAATTGCTTTATATGGATCCTATAAGGTTGAAACCAAAAGTGAAAATAAGATTCCCAAAAATTTACAAGATGAAATTTCCATTTCTTCATCAGAATAAGAGTTCCTTTTGAAGCCAGAATAACTTTTCCTTGATATCGAACATAATGTATTAAAGTATCTTTGAGGAACCATAGGATCCTCTGAAAAGAATTACAACACACGACTATAAGATATTCTATTTTTCCATAGAAATGTGTTCGCTCAAGAAAGACTCCAGAAGATATTGATCGTAAATAAGAAGACTGTTTACGAAGAAACAGGAATATATATTCGCATTCATATACATAAGAATTATGTAGGAACCAAAAGAATCTTTTCTTTCTTTTTGAAAAGACGTAAATGGATTTCTTTGAAGTAATGATACTATTCAAATTATGATATTCGTGGAAAAACAATCGCAATAAATGCAAAGAAGGAACATCTTTGATCCAGCATTGAAGGATTTGAACCAAGATTTCCAGATGGATGGGATGGGGTATTAGTAGATCTGACACATAATTTAAATGTGATAATTTATCCTCTAAAAAAGGAAATATTGAATGAATAGATCGTAAATTCTGATATTTTGGTATTCTTTTTTCTTCAAGGGAAGATACTAATCGCGACGAGAATGGAATTTCCAGAATGACTCCAAAACCTTCTGATACCATTTGAGAAAAAAAATTAGAAGAAAAAGAATTTTTGTGCCCCCAAAATCCATTTTCCTTTTGGTTAGAATCATTCATCGAATAAATCAAAGATTTCTGTTGATACATTCGAGTAATTAAACGTTTCACAAGTACTAAACTAGATTTATTGTCATAACCGAGAATTTCCACAGGTTCGTAAAAAATCAAACTATTGAAGCTATGATAATGAGCAAGTGAGTAAATATACTCCTGAAGGAGTAGTGGATATAGTAAGTTTTGTTGCCGAAATCTATCTTTTTTCAATAGAAATATCCTTGTAATTCTGCCATTTAAATACATTTATACTGTAACCAAAAAGGGGGAGGCACTTCTTGTGTTATGAAATGATACATAGTGCAATATGGTCAGAACGGCGTATGCATATGTTGTATGTAGTATATTGTTTCTCTTATACTAAAATACTAAAATAGTATAACTTATAACTAGACTAAACTATAATAATAATAGAAGAATAATATTATTCTTCTAATTATTTTCTATTATTAGAAGAGATAATTCTAATAATATAGTCTAGTATTATTATATACTATGCACTGTCTATACTTTTACTTTCAATAATATAGACTTTTATACATGTAAAAAACATAATGATAATCTAATGAAGTAAAAGATTATATAAAAGTAAGAATAAAAGTAAGAACAAATAAAGAATATATACCCCGGAGACAGAAAGCCCAATCTACAGATCTTTTTCCTTTCTATCCAATTTGTTTTTCTTTTACTTGTTAATATAAGTAGAATAACAAGAAAAGAAATAAATAAAATAACAATAAGAAAAAGGGGTAAAAATCTTTTATTTTTGCAACCCAATCACTCTTTTGACTTTGGAAAAAAAGACCTTTTTTATCACTATACTATTTATTATACACATCCGTCTTCAATCCACAATAAAGAATAATTAGGATTAATAAAAAAAAAAGAATCAATGGTCCACTTACAATTCACAAGAGAACCTTTTCCCACATCAGGCACTAATCTATTTTTAACGCATAATTAGTAATTTTATTGGGTAATCATTCAAATTAAGAAGGGAAGCTCGTTGCTTTTTTGTCTGACTCGAATTGGAGCCATAAGGCTCTATCCATTTATTCACTAGACCCAAAAGACTTTATCAATTGTTATCAAAAGAAAAACTCTCGTTCTATTTCTATTATGAGTACTAGAAATTGAGTACTAGAAATCTTCTTTTTCTATGATTATATTATTCTTTTTTATATTTTTTTATATTTTTATATTCTTTTTACGACATGCTTTTTTTTCCATTCATTACCTTTAAGGATCAGTCGCAGTCTTATAAACTCTACCAATAGTCTAGACGAATTCCTTCATCCAAATGTGTAAAAGATCATAGTCGCAATTAAAAGCCGAGTACTCTACCGTTGAGTTAGCAACCCGGATCAATATGAAGTGTAGATATGATCGAAATAAAAAAATCCAGCAAACTCATTCATTTTACTAAAGTGAAGGAAAGAGCCGATAGGGAATGGGGATAAAAAGATCTATTTATATACGATCAAATTATATTTGTTTGATACGCCATTGTCAATATGAATGGACTTTTTATCAAACAAATTTCAAGAAATTATATAGAAATTTGTGTTGGATTAGCATAATATAAAGAATAAAACTTTGAGCGGAAAAAAATAAGGAAAAGGTAAAGATAGAAAAAATAACGGCTTTATTTAATCAAAAAAAGAAAGGTACAATACAAATACAAGAAAAAAGATTACCCCCCTTGTTTGGGGGGTTCCACAAAAAGAAGCAAGAAAAAGAAGAATAAAATAAGTATGAATAGAACAAGAAAAGAAAAAACTTTGAATAAAGATTTACACAAAGATAGGTACTAGTTACCCTATCCTTTTTTTATTCATGATTCTTGTTTTTCCTTTCTTTTTTTATCAAAAGAAACTCGAAATTCTTTAAATAATTCTGCCTTCCTTAAAATATCATAAACAGTTCCTGTGGGTTGAGCACCTTTTTCAAGGAAATAGAAAATAGCAGAAACATTTGAATAAGTTTGATTCTTTATCGGATCATAAAAACCCACTTTTCGAAGATCTCTTCCTTCTCTTCGGGATTGAACATCAATTGCAACGATTTGATAGATGGCTCATTGGGATAGATGTAGATAAAAGATGCCCCCCTAGAAACGTATAAGAGGTTTTCTCCTCATACGGCTCAAGAAAAAATGATTCTAATTTATATAATTTATATATATATTATATATATCTATATAAATAATAGATAGATAGAAAGAGAAATGGATCCATAAAGAATTAGACTGTAATTTGAGCCTTTTTTTTCCTTCTTTACAGAAAAATAAATTTCATTTATACTCCTAACTCAAGTTGGGTAGTTTTGAAAGAGTTCGAAAGGAAATCCTTAGAATTTCTTGAGCTGTCTATAACCTCTTTTTTTGTCTCCTTTCGGATATATTTTTTTTTACTCATTCTGATCCAATTGTTGAGACAAGTGAAAATAGTGTTTCCTTGTTCCGGAATTTGTTGTCTTTGCTTTGCGCTTTGTGAAATCATTGGGTTTAGACATTACTTCGGTGATCCTTACTCCTTTTCAAAAAGGCAGCAACATACCTTTTGTTTTTTGTTCTTTCTATGAAAAGAAAAGGAAAAAATTATACGAAAGATTGATTCCTTTGTGATACACTCTTGATTGAAACAGTTTGAAAATTTCGACAAATTTGATTTTTTCATTTCAAACTTGTTCATTTTTGAACCTCTCCATTTATCTATATTTAAATATTGATGATATATTTACAAAGTTGGTCTAACTTATTAATTGTTACTAACCCTAGATTATTCCTCCGATAAATGAATCAATCATTTTTGCTCGAGCTCCATCATATGCTATACCTTTATATACCATTAGTATCTTTATTTAGTTATTTAGCAACCCAAAACAAATTCAATTAGGTTCCTAGCAGAACAAATTATGTCGAGACAAGAGCATCTTCATTCGTATAGAAAGAGAAGATGAAGATGGTGGATGTCAGAATCCACAGCCAATCATGTCCTTCAAGTCGCACGTTGCTTTCTACCACATCGTTTCAAACGAAGTTTTACCATAACATCCCTCTCATTTTATTTTAATTTTGAACCGTATGCAATTGATTCAATATGGAATCATGAATAGTCATTGGCTGAACCAATCGATACATAATAATCTACACTTATAGATAAGTGTTATCCGGAAAGGATTTCACTTAAAAATACCCCATATTTTCTTATATGATATGAATAATATCACATGAAAAAAAAACAAATCAGTTTTAAGCAAACTTATTTACATCTTCAACAGATCTTTCGGGATTGTCCATCATAAACCTCTTTTTTTTTTAATAAATTAGAAACCTCAAAAAAAGCCTTTGACTTTACTTTCATTCAAATGAAAAAAAATACCCATGAATGGATAAAAGTTTTTAGCCACTTTAACTAAATTTAATTAAATGTTTAACTAAATATTTAATTATTATAATAATAAGATAATCTGAAATAATAAGATAATATTAATAAGAAAAATATACAAAATAAAAATATACAAATAATATACAAATACAATATATAATATATATTTATAATTTTATAATTTTAGAAATTTATAATTTAAATTTAATAATTTATATTTTAATTTTCTTTCTATTTTATACTCTTATGTAATATATGTAATAATTATGTAATGTAATAATTATGTATTTATGTATTAATAAATTCTAATATTAATATATATGAATATATTAATTATGAATATTTTTTGAATATTTTCTCATTTTTTATTTATGAGATATTATTTTTCTAATATTATGATTTACTTATTATTTACCATCTCCAATTGAATCTTATTTTCATTTAATTGAAAACAGAGAGATAGTTTGAGAATAAAGTTTCTTTGTTTTCATTATTATGGAGTAGAAAAAGTCTCGTGTAAGGTAAGATCAAAGAGAAAATAAGAATCCGAGGATTCTTATCTTTTCGCATCCATCTCCAGTAAAAAAGTAAAAAAAAAAAAAAAGATATGATTCTAAGAATGATTCTTAGAATTCAGATTGGATAACATTGTATCCAACATTAAACAGAAAATTGTTGAATTAAAATTTAAATTTCAATAGAGAAGAATCTTTCGTTTCTAATGCAAACGATCTGGGACGGAAGGATTCGAACCTCCGAGTAACGGGACCAAAACCCGTTGCCTTACCGCTTGGCCACGCCCCATTTTGATTTGGTCTAAGAAAAAATAAGATAAATATTGGTTATTCGTCAATAACCAACCAAAATAAATATAGGACATGTTGTCGCTAGGATTCAACACAATAGATATAGAATCAAAAAGATTAATTGATCATTACTTAGAATTCAATTAAGATATTGTATGAAAATAGAATTCCTTGTATTCTTATTTGATTGGAAAATTTAAGGAAGGATTCTTGATTAGGGAGAAGTCAAAGAAAAATAAGAATTTCTTTCTTTTATCTGCATCTGCCTTTTTATTTGAAAATTTTCCTCAGAAAAACAACTCAATCCAATCTGATAATTTATTATCATTTCAATTTAATTTGAATCTTTAAGAACAAGAAAAGAATATTTGTTATGTTTAATATCTTTAGTTTAATCTGTATCTGTCTTAATTATACCCTTTATTCGAGTAGTTTTTTCTTCTCCAAATTGCCCGAGGCTTATGCCTTTTTCAATCCAATCGTAGACGTTATGCCGGTTATCCCTGTACTCTTTTTTCTTTTAGCCTTTGTTTGGCAAGCTGCTGTAAGTTTTCGATAAAAATGAAATATCTATTCAATTCATAATTTATTCGATAAAAAAAAGATGAGATTTTTATTCTGAAAATCAATAAGATCATAAATAAGATTCAGCTAAGTCTGGACATTAGGAACCCTCGATTCAAAAAGTGAAATTCTGGTATTTTATATTTTATATTGAAATTTCATTTGAATAAGGGAAAGGGGTGATGATCTTTATCTTTAATCAGCTAATCAGCTTATTTCTTCTTTTGTTCTGACCTTTCCTTTATAAAATCCCATACAATACCTAATATGGATATGACAAGAAATTTTTGGTAACGAAAAAATATGAATCTTATTACATTAGAAAGAAATTCGTGAAAAGAAATTTCAAAAAAACTTCCTTTTTTTTCATCTTTAGAGCTTCATATCAAAATAGTGTCTAGTTTGTGTCGTAAAATAGGTGATCTATTTCCTTAAAAAAAATGATCTTATCTTATCTTGGAGATTTGTAATGCTTACTCTTAAACTATTCGTTTACACAGTAGTAATATTCTTTGTTTCTCTCTTCATCTTCGGATTCTTATCTAATGATCCGGGGCGTAATCCCGGGCGTGAAGAATAAAAAAAGATATGAGATTTGTTTTTACTTTTTCCTTAATTTTTTCATAGGTAAATGTATAAAGAAATGGAATAGATGCTAGATAAAGATAAAAGATTCATAAAAGAAAATAATCGAAATTTATTTAAATTCTAAAATCTTAAGTGATCAGAGGGGGGTCGGAGAGAGAGGGATTCGAACCCTCGGTACGAATAATTCGTACAACGGATTAGCAATCCGACGCTTTAGTCCACTCAGCCATCTCTCCCCATTCAAAATTGTAAATTTATCTTTAACACGTGTAAGTCAAGATTGAGAACAATTTTTATTTTCCTTCAATGATTCAAAACATATTTCTCCAATAGAAAGAATACCTTACTTCTTTTATTTTGTACAAAAGGTTCATTTCCCCGGCCTGGTTAAGTATAAGTACTGGCCGGGCCAGTACTTCTTTTGTTTCGACGAATAAAAATTTTTATTGAAACAAGAATAGTAATTTTTATTCCCATTCCTAATTATTAGGAAATTATATAAGATTCTAATAGATAGATTATCTTATAAATTCTTTCAAAAATTATCTAGATCTAGATTAATGATTAATATAGAATATTCTATATTGAAATTTAAATATTAGAGATTATATATCTATTCTTAGTATATCTTAGTTAGTATATCTTAGTATATTATATATTATAATATATTATAGTACCTTATATAGTAGTACCTTATATAGTAATTCTAGTAAATTAGAGTATAAATGAGTATAAATTCTAATATTTAGTCTTTATAGTAAAAGTGTTCTACTATGATATAGTATTATAGTATCTAGTAATATAGTACTAATCGTCGTCTTTATTTTCTTATTTTTCAGTATAAAATTCGGAAATTCATTAATGAAAAACGAATTTCATTATAAATGAAAGTTGAAGTTTAATATTATATTCATCTTAATAATTCACTTAATAAGTCTTAATAAGAAAGAAGTATTAAGAAAGAAAAAAAAATACTTATAACTTTAGTACACTATTGAAATTTGACTAAACTTTTTACTATTCACTTATTTTTTTTTCAAGTTTTAAATTACGCGCCGCAGTGGAACAAAATACGTGTTAATGCTGGAATTTTCATGATTCTGATGCTATCTTGACTGCGTCTGATTACTTTGTTGGACAAATGGTCCATTCATATCCAATAATTAATATGTAGCGGGTATAGTTTAGTGGTAAAAGTGTGATTCGTTCTATTAACAACTTCAATAGTTAAAGGGTCTTTCCATTTTTTTCATATTTCATATTCCGATCAAAAACTTTATTTCTTAAAAAGATTTAATCCTTTAAACCTCAATAGGACATTTGAGGAAAGAATATACATTCTCACGATTTCTATCCAAAAGGCAATCAGAATTTTCATAAAAAAATTGGATTATGGAGTCGAGAAGCATAATTTTTTTGATTGGTTCAATTCTTCCAATTGAATGAGTATGAATAAAGGATCTATGGATGATAGTACAAAACTTTCAATCGTAACTAAATCTTCAATTTTTGCGCTGAAAAAGGGGGAGATTGAAGCCAAATAGCTAGAAAATGATGGTTTTGGTTTACTAGAACCCTCGGCTTCTTGTTTCAGCTCGGTAGAAACAAAATTCTTTTCCTTAGGATCCCGCGAGTAAAAAAGAAATAGGGAACGAAGTAACTAGACTAGAAAGATTTGATAGAATCCCCCTCTTCTATAGGGATCATCTAAAAAGCAAGTAGCTTTAGATGCATTCGTAAAAAAAAAAGCTGACATAGATGTTATGGATCTCATTTTTTCTCTGGTGGGAATACATAGATCTTCCATAAAGGAGCCGAATGAAACCAAAATCTCATGTTCGGTTTTGAATTAGAGATGTTAAAAATGATCAACCAACGTCGACTATAACCCCTAGCCTTCCAAGCTAACGATGCGGGTTCGATTCCCGCTACCCGCTATATATATTCATTCCTTAACTTCATTTAACTTCATATGATATACAATATTATATACAGATGCATTATCCTTTTCTTTTTGATATGCATCCTTCTTTTTATTGTATTCCCTAAATCCGTCTAATCCTTATTTTTTTTTATGAAAAAAATGCGAAAATAGGAATGAAGGGCGTCCATTGTCTAATGGATAGGACAGAGGTCTTCTAAACCTTTGGTATAGGTTCAAATCCTATTGGACGCAATTTATTTCTATATATCTATTCTAGATAGAGAATAGAAAAAAAAAGAAGAACTATATTTTAGAAAGGATAAGGATAAAGGCCCTTTTTTGAATGATTCGAATCAAAAATCTTTATCAAGGATTTCTCTTTATTAAGAATATAATAAAAACCACCCATTTACATTTATGCTTGTTCCTGAAGTAGAAAGAGTTCGATCTGTTCCTGAATAGCTTCTCTCAAAAGGGTTTCAGCTTCTTCGGTGAATATCTTGGTAGAAGATAGAATTTCTTGAAATTTAGGTTTCTTCTTTGTTAAGTAGGTACGTAACCCAACGAGAAATTTCTTTACCTGTCCAATTTCTAACGCATCAAGATATCCATTCGTTCCGGTGTAAATAGTAGCTATCTGGTCTTCCACCGCGAGAGGGTCTGATTGGGATTGTTTGAGCAACTCACGTAATCGTTGACCTCTTGCCAATTGATTCTGAGTAGCTTTATCTAGATCAGAAGCAAATTGTGCAAAGGCTTCTAATTCTGCAAATTGAGCTAGTTCCAATTTTGATTTGCCAGCTACTTGTTTCATGGCTTTAATTTGAGCTGCCGATCCTACTCTGGAAACAGAAATACCTACATTAATAGCAGGCCTGATTCCGGCATTGAATAGATCGGCAGATAAGAATATTTGTCCATCTGTAATAGAAATTACATTAGTAGGAATATAAGCCGAAACGTCTCCAGATTGAGTCTCTACTATTGGTAAAGCAGTCATACTTCCTTCACCTAAACGAGAACTTGATTTAGCGGCTCTTTCCAAAAGTCGTGAATGCAAATAAAAAACATCTCCTGGATAAGCTTCACGACCAGGAGGTCTTCTTAATAGAAGAGACATTTGACGATAAGCTTGTGCCTGTTTGGAGAGATCATCATAAATTATTAAAGTATGTCGTTCACGGTACATAAAATATTCAGCCAGAGCCGCTCCCGTATAAGGAGCGAGATATTGTAATGTAGCAGGCGAATCTGCTGTTTCGGCTACCACAATAGTGTATTCCATTGCTCCTCCTTCCTGGAAAGTAGTCACTACCTGAGCCACAGAAGATGCTTTTTGACCAATAGCTACATAAACACATATCACATTTTGTCCTTTTTGATTGAGAATCGTATCTGTGGCTACTGCTGTTTTACCAGTCTGTCTGTCTCCAATAATTAATTCTCGTTGCCCGCGTCCTATAGGGATCATTGAATCAATAGCAATAAGTCCCGTTTGAAGGGGCTCATATACGGAACGTCTCGAAATAATACCTGGGGCAGGAGATTCAATTAACCGAGATTCAGAAGATGAAATTTCACCTCTCCCATCAATAGGTTTAGCCAGAGCATTTATAACACGACCCAAAAAAGCCTCGCTCACAGGGATCTGAGCGATTCTTCCTGTTGCTTTTACAGAACTTCCCTCTTTTATCATCAAACCGTCACCCATTAATACAACGCCAACATTATTTGATTCCAAATTCAGAGCAATACCTATGGTACCCTCTTCAAATTCTATTAATTCACCTGCCATTACTTCATCAAGACCATGAATACGAGCAATGCCGTCGCCCACTTGAAGTACGGTACCAGTATTCACAATCTTTACTTCTCTATTATATTGTTCAATACGTTCGCGTATAATATTACTAATTTCGTCAGCTCGAAGGGTTCCCATTAGTATCTCTTTTTTATTTTTCGGAAGGAAAGGAAAAAAATAATACCTAAACTCTAAACTAGAGTAAAAAGGCTAATCAGTTATTTCTTCCACGGACCCGAGGATACCAATATTAGCACTGATGGTACGAAAATGTAATTCGCTATTTAAACAACTATTCAAAATTCCTAGAGCTTTTTGTAAGGCTTGTTGCAAAACTTGTTGTCGGACCTGATTAATTGCTCTTTGTTTTTCAAAAAAAAGAGTTTCATTTTTGTAATTTTCTAATCGTTCCAAACTATTGCAAGTAGCATTAATCAAATTTACTTTTTCTCGTTCTATATCGGAGTATCCATTCGTTCGATACTCATCTGCTTCGATTTCCACTTTCCGTAATCTAACCCGAGCTCTTTCGAGCTGTTCAATGGCTCTTTTACGTAATTCTTCTGAATTTCGAATAGTACTCAAGATCCTCTGTTTTCGCTTATCTAATAAATCATTTAATGAAAGTAGATTATCTTTCCATTCATTTAACAACTATAATATCTCTTCCCGAACCAAACATGAATCTTTCGATTCATTTGGCTCTCACGCTCAATTGTTTCTTTTATCTTTTCTTTGATTGATGGGCATTCCCATATCTTTGAACGGAATGAGCCTATCCTCTATTTTCTGTTGGTATATCGAAACTGATCTAACATCAGAATCTTAGGAGGACTCTTCAGACCATACAAAAAAGAAAAAATTGTCAGCAAAGTTGTTTCTTTATTTGTTCTTTATTTACAACTTATTTACAAAAAGAAAAAAAAAAAGATTTTAAAGAAAAAATAATTCTATTCTTTCTTCTTTATATGCTATGATAAATTAGATCAAAATTCTAATAGATAATATATAATTGAATAGAATAATTGAATAGAATTTTTAACTTCATTACTTCATTATCATTATTATTAGAATGATATTTCGATTTTTTTATCCAAAAAATTCTCTTTTTTATACAAATAGAATACATAGGTCGTCAATTCGGCAGTGGATAAAAAAAGGGGGGGAGATACCCATCTTTCCAGTTAATGGTTCAAATAATTTTATCCATATGAGTGTTCTACATCGGATAAATTCCCAATTATTCCTTTTTTATCATCTTTCAACCTTTTTGTTACTAACGTAGCGGTAGAAAGAGTACCATACTATGCTGTGCCTGGACTTCAAACAATTTATCTTTAACCATGTAAAAGACCTCAACATTATTGGTTGATAGAGAAGATAATCAAAGTTCATTTATCAATTAGTCACGAAATGCTATGGTTCTTACATATGATCTCTGAATGAAATCCAATTCCGAAGTAATTCGTCGAGATTGTGCACCCTTTGTTCCTATTTCTGCTATAAAAAAGAATACATAAATATAAAGAAAGTGCAGCCGGTGAAATCCAACCTATTCTTGAAATACACAACTCGCACACACTCCCTTTCCAAAAAAAATTAATACACCCAGCACTACGCTTAGATTTATTGGATTTGTTGCTAAAATATCGGTATTAAACTCGAAACTCCCAGCGGATGGCCAGTGCTCCACGGAAACAAAAGAATCGATTCTATTTTTCATAAGCTCTCCCCTTATAGATAGGACTAACAAAGAACAGAGTTCTTTTTGTATCACTCCGCTTATTATTCTTAATGGAATTTGAGAATTCTCAAATTTATTAGTTATGGTTATGTTTTTATTCTTTTTTTTTTTTTACATTTTTATTATACGATGAAATTAAACATTAAACAAAAGGATTTGCAAATAAAAGAGCTAATGCCACGACCAGTCCATAAATTGTTAAAGCTTCCATAAAAGCCAGACTAAGCAATAGAGTACCTCGTATTTTACCCTCTGCTTCTGGTTGTCTCGCAATACCTTCTACGGCTTGACCCGCAGCAGTACCTTGACCGACCCCAGGTCCGATAGAAGCAAGCCCTACAGCCAATCCAGCAGCAATAACGGAAGCAGCAGAAATAAGTGGATTCATGGTAAGTTCCTCGCACCAAAAAAAGAAATGGTTAATGATACAACGAACCAATGAATTAGTACTTAATCTACTTATTTTTCTACTTCTACTTTTACTATTTACTACACTTATTTTTCATTTTTTGATCTTTATCACCAAAATATATCGGGTCGAAGTAGCTAAAAGCTCCAAATGGAATTCAGAATATTACTGAATTGTCAGAACTACTTCGATATACCGTTTTTCCTTTCTACCTTATGTAAATAGATATGTATACGGTTTTAGTCATTGCGTTTCCTTGTTTAGAAACTATTCTATTCTTTCTCTTTCATTTTTCATTCAATTCACAATCACAGACAAAATAGAAAAAGGACTGATATGGAAATTCATCTAAATGCAGTGGACTAGAAAAAAAAGAGATAGGGGTAATTACTATCTAACTAGTAAATAACATATACTTTTATTCTTCCATAAATAACGTAAATCACCTGCACTTTTTATTCTATTAAATAGTATTCTGGAACCATTCTTCGAAACATACACAAGGATTTTTACTCATAGGCATTACATATACATATATGTAGTAGGAGCCCCAACCCTTCTTTCTTTTCCTATCTTTCTTCATATATACATACATATAGCTATTTGCATTTTATTCTCCAACCTAGTGGATTATATTGAACCCCCTTTGAATTGGGATAAGCTTCAAAAAAGACTATTTCGAAAGTTAGTCAATGATGACCCTCCATGGATTCACCTATATAAGCCGCAGCCAAAGTTGCAAAAATAAGAGCTTGAATACCGCTTGTAAATAATCCAAGAAACATGACAGGTATAGGAACTACTGAAGGCACTAAAGAAACAAGAACAACAACCACTAATTCATCAGCCAATATATTCCCGAAAAGTCGAAAACTAAGAGATAAAGGTTTTGTGAAATCTTCTAGAATATTAATTGGTAAAAGTATTGGAGTTGGTTGAATGTATTTCCCAAAATATCCCAATCCTTTTTTGCTAAGACCCGCATAGAAATATGCCACTGACGTGGGTAAAGCTAAAGCAACAGTAGTATTTATATCATTCGTGGGCGCAGCTAACTCCCCATGAGGTAACTTTATGATTTTCCAAGGTAAAAGAGCACCTGACCAGTTAGAAACAAAAATAAATAGGAACATCGTTCCTATAAAAGGAACCCAAGGACCATACTCCTCTCCAATCTGAGTTTTGCTCAAGTCTTGAATAAATTCAAGAACATATTCGAAGAAATTCTGACCGTCGGTCGGAATGGTTTGTGGATTTCGAACAGCTATGATGACTGAACCTAATAAGATAGCAATTACAACCCAAGAAGTGATAAGTACTTGGGCATGAATTTGTAAACCTCCTATTTGCCAATATAAATGTTGGCCTACTTCTACACCTGATATATCGTATAATCCTTTGAGTGTTTTAATGGAACATGGTATAAAATTCATATTGTCCTCTAACAGAAATCGAACTTCAAAAAAGTAATTATTTTGATTCAACCATCTCTTTCTCAATTCATCTATGTTCATTCATGAATTTAAGTTATGAATCGTATATTTCGGATACCGAGAAATCACATAAAAAAAATACCAATCATTTTATCTTTTAAATATTCTTCAATATTCAAAACATAGTTCAAACTCCAAAAGATGCAAACCAAAATAGTAACAATCAAAGAGAGTTCGCCAAAAACAAACTAATCTTCTTCTTTCTTCTTCTTAATGATAAGAGTAATGATAAGATATTCAACTGTTTTTTATATAACTAGAACGGCCCTCACAAATTGCAGATACTAATTTGGTAAGAATCAATCGAATCGAAGCTATAGCATCATCGTTGGACGGAATAGAAATATCTGCAAGATCTGGGTCACAATTTGTATCGATTAAACAAATCGTCGGAATACCCAAAATAACACATTCTCGAAGAACCGTATATTCTTCTTGCTGATCAACGATAATTACAATATCGGGCAATCCCGTCATATATTTGATCCCACCCAGATATGTTTGCAAGGTAGATAACTTTCTCTTCAACATTGCTGCATCTCCTTTGGGAAGACGATTGAATTTCCCCATCTTTTGTTCTGCTCTTAAGTCCCTGAATTTCTGAAGTCTTGTTTCTGTAGTAGACCAATTCGTTAACATACCACCAAGCCATTTTTTATTAACATAATGACATCGAGCCCTTATTGCTGCTGATGCTACTAAATCCGCTGCTTTCTTTTTAGTGCCAACGATTAAGAATTTTTTTCCCCTACTTGCTGCATCAAAAACTAAATCGCAGGCTTCTGATAAAAAACGAGCAGTTATAGTAAGATTTGTAATATGAATACCCTTACGCTTTGCAGAGATGTAAGGAGCCATTCTAGGATTCCATTTATTAGTACCATGACCAAAATGAACTCCTGCTTCCATCATTTCTTCCAAATTGATGTTCCAATATCGTCTTCCCATTTTCCCACACTTTCTCTTTTTATTTTTTTTTTTCAAAGAGATTCATTACCTTGTGAAATAAATAATTGTTCCAACGGAACCTTCTACTAGGATTGACCTTTGATCTACGACCCAAACCATGAATTTCATTCTTTATTTTTGATTTCATTGATTACGAAATCCATAATTGGACCAGAGAGTTAAAGTAAAAAGAATGAACCTCTTGTTAGGAATTAGTAAATTACATTACGTAATTTATTGGTCTGATGTCTCATGGAAAGTGTTTGGAGAATAAGAACAAAATAATTCTCTATGGTGTAACAAAATATCTCTCATTTCCAACTCGAATAGATTCTTCCTTTTAATTTTCAAATGAATGTTTTTGTCTTTCTTTGAACGATGTACTAATTTTTTGAATCCGGTACCAACTGGTATAATCCCCCCCAAAACAACGTTCTCTTTCAGGCCTTTCAACCAATCAATACGACCTCGTAGAGCAGCTTTTGCTAAAACTCGAGCAGTTTCTTGAAAACTCGCTTCGGATATGAAACTTTGAGTATTCAGAGATGACTTCGTTATTCCCAATAAGATTGCCCGATAAAAGATCGCTTCGTCCAAAACGCGCCCTGCTCGCTCTGCTCGCAACAATCCAATAAATTCCCCAGGTAAAAAAACATTAGACATTCCATCTTCTGAAACCAAAACTCTTGATGTTACTTGACGTACAATAATCTCTATATGTCTATTATGGATCTGTACCCCCTGGGATCGATAAACCTTTTGGATCTTATTAACCAAAGAGATACGACTTTGGGCTATGGTTAGTTCAGCTCCAATCAAGAATCCCCAGGGGATCCCAAGAATCCTTCGGATACGTTCGTCCCAACCTTCAACTCTTCTTTCGAGGTTCATCGATATTGAATCAATCGAACGAACTTCTAAGATTTGTTCCACTTTTGGAAGACCTTGCGTTATGTCACCAGATCTCGATTTTTCATATATAAATGTAATTAATGTATTTCCTTCAGAAAAGGTTTCCCCATAATGGCCATGTACAGTTGCTCCTGGAGTGACCAAATAGGGCTTAGCTGATCTTATAACTAAAGAGTCAACATGAACAATGAAAATTTGACCAGATTTTTTTATGCGTGATCCGTATTTCAATAGACATACATTTTCACAAAGGAATTGTCCAAGGCTAATTATTGTCCATGCCTCTTCACAAAAATCGTGATGGAGAAAACACCAATTCGAATGGAATGAATTCCAAATGATGTTACTGCATGGATCGGGATTAAAAATCCTACTATTTTCATCTAGGAAACAGTATTGAAATTGAAATGGAAGTACTTGAAGCACTTGGAAAGTCTGTTGAAAATTTTCAAATAAAAAATCTTTCTTTAAAAAGACTTGATTATAAGTTCTTAAATAGTAAGATGAACAAAATTTTACTATTTTAGGCACAATAGTACCTAAAGGACCCAACAAATCCCTAATTGGAGTCATGGGATCCGATTCTTTTGTCGCATTATTATATCTCGAAGTATTGAAGGAGCCAATTCGAGAACAATTGGATGATGACAAAATTATGAAAGATTGGCATTCCTTATTTTGATTCAACAACGTACCAAGAGTTCCCTTATGTTGGGGAAATGATTGAATCTTCGCCTTGGAATCAAAAGGATTTCTATGGGTACGATCTAATTCATTATTGGAAATAAATCCTGAACCTGCCGTATCATACCTTTTTTCGGTATACGAAATAGTGGACTTTACTAACTCAATTCGGATGAAATCACGAAGCAGATCATTTGCCCTTATTTCAACAAAAGAAGCATGAACCTCTTCTTTTATAGAATTCTTTTTTTCTTGGTCCCAAGTCAATACTAAGCAAGCCCGAACTAATTGAATACTTGTGTGAGAAATTCCTCGAATTGACTTGCCATTTCCATAAAGTATATAATTGACAATTCGAAGTTGGATATTATCCTTTTCCTGCAAGAGATCCTGAGAGAAAAGTGTTGCTAAATTTATCCCATCAGCTATTTCATATGTGACTACGGGCCGAACCAAAACAAAATACTTTTTTTTGGTAGGTGTAATCCGTTGGACATAGATCCAATTTTTCAATTTTTTTGATCCTTTAGAATTTTTTTTTTCCATTCCTGGTGGTATCAAAATGCCACTGTGCCTAGATATTTTATCCACCTCTCCAGAAAAATGAATATCTCCAGAAAATATTTTAAGTTCCATACTTTTTTTTTTTCTCTCCACTCGGACTAATCCACCTACTCGACTTCTTGTATTTATATTTAAAGCAAGTCGTGTATCTACTCCAATGATACTATTGTTCCGGACCATTATGGGCGAAGATCCGGGTAAGATATGCACTTCCTCGGGAATAAAAAAAAACCGATCTACTTTCATTTGCATTTGGTATTTCGGACTAAATTCTTTTGTTCCTCGATACTCAATCAAATCCTCTTTTTTTACGATTGAATCTACTTCGACAATCCCATATTTAGTAATTCCCGAACTGCTTCTTCTGTATCGCGGATCATCAAAATAAGCAAGAATACTATTTCTACGTAAAATACCATTTATAGGTATTTTAATCGAAATACCAAAACAGGGTATTAGTTTCTTTTCTTGTTCTTGATCATATTGTAAGGGAATCACGAATCTATTTCTTCGCTTTTTCGCCAAGGAATTCTCTTGACGAATATAAGTAGAAGGCTCTATGAGATTCCAATGATCCTTGGATCTGATTCGATAAGGTTTTGAATAGTCAAAAATTTCCCTATATTTTTTACCAAAAGTATCCAACAATTTATGTCTTACTTGATCATTAGTCAGTGAGAGATCAAAGATATATCTTTCTTCGAGAGAAAAAGAATTAGCATTCATTTGATCTTGATTCTTGTGGAGTGAAAAAGACACTATATTGGATCTGCATAGACCTCCTGCTAATATCCATAAATGACTTGTTTTTGGTAATAAATGAACATTACTATATGGATATTCGGTCGCATGATAGCCATCAGTACTCCAGTGCATTTCTCCTTCTGACTCAGAATAAATATGTTTTTGAACTCTCTCTTTAAAATGAAAAGTGGACGTTCCGGTACGAATCTCGGCAATCACTTGTTCTGATTCTACATATTGATCATTTTGAACTAAAATCAAACTTTTTGTTGGAATATTCACATTATGTAGAATATCTCGACTCTCAATAGTTACATACAAGTCTATAAAACATAGAAAAGCAGGATGCCCATGACGGGTACGTGTGGGATGAACCAAATCCTCATCAAATTTTATTTTTCCATTAGAGGGAGCTCGTACATGTTCGGCAGTACCACCCGTGAATACTCCGCCGGTATGAAAAGTTCTTAATGTTAGTTGAGTCCCCGGTTCCCCAATTGATTGACCCGCAATAATGCCTACGGCTTCTCCCAACTCGACCAGGTCACCATGAGTAGGACTCCGGCCATAACATAATTGACAGATCCAAGATGTACTCCTGCAAGTAAAAGGGGTTCGAATATATATTGGGTGTGCTCGAAAGGTTATGAATTGATTAACAAGTCCAATTCCAATATCTTTATTTCGAGTGGCAATGCATCGTAGACCAATATATATATCGTCTGCTAATACACGACCAATTAGTGTTTGGACAAAAATCTTTTCCGTCATCCCATTTCGAGGACTCACGGAAATACCTCGGATAGTACCACAATCCGTTCTACGTACAAGAATGTGTTGAACTACTTCAACAAGTCTACGCGTGAGGTATCCAGCATCTGATGTTCGTACAGCAGTATCTACAACTCCTTTGCGGGCTCCGTAGCAAGAAATTATATATTCTGTCAAAGAAAGCCCTTCCCGTAAATTGCTTTGAATGGGTAAATCAATCATTTGTCCTTGAGGATCCGACATTAATCCTCTCATACCTACTAATTGGTGTACTTGAGATGCATTTCCTCTAGCCCCCGAAAAGGACATTAGATGGACTGGATTAGAGGGATCAGTCATCCGAAAATTAGGATTCATTTCTTGTCTCAAATATTCACTTGTAGCATACCATATCTCAATGGATTGACGTAATTTTTCTACCACATGTACATTCCCATAATGATGGTTTTTCTCTAAAAGAAAACTTTGCTGTTCAGCGTCTTGAACTAACCATCCTTTAGAAGGTATTGTTAAAAGATCATCAATTCCTAATGAAATAGATGTAGCAGTGGCTCGCTGGAAACCCAAAGCCTTCACTTGATCCAGGATATGTGATGTATATGCCATTCCGAAATGATCTATTAATCTGCTAATAAGTCGTTTCATAGCAGTTCCATCTATCACCTTATTGTGAAAGACCAGATCGGTCCGTTCTGCCATAAGGACCTCCATATTCTGCTGAGTAAGATTCCACAATGGGCCTGGGTCAGTGATTCGAAAACTTCCTTTCCTAAATTTTAATTCACACAGAAATTCATAAATTATGATACCGGTGAAATTGGAGAGACCCGAATTCCCACGAGTATGGGCATCACTGATAGAATTTCTTAGTTTTTATATAGAGCATGAGTTAGATAGCATAGCCTATGAGTAGGCCCGCCAAAACCCCTGTATAGCTTCTTCTATTTCTCGATAAAAAGAAAGATGACCAACAGTAGTTCGAATGTATATACAACGAATTTCTCTTTTTACACTTCCTATTATTCGATAGTGCTTATAAATCTCATTATAATTACCAAAAGATTCATATTGAACTTCGATGGGAACTTCTCTTGAGCCAACGACGCGTTGGTCTAGTCTCCACCGGAGCCACAAAGGACTATCTAAATGGATTCGTTTCTGCCGATAAGCTCCAAGTGCATCATAAGAACTAGAAAAATACGGTTCTTTTGTATACTTAAAGTCATTCTTGTAAACTGTTTCCTTTTTATAGTTTCTGCAATTATAATTATATTGATTATACCTATTTGCACAAATACCTCGGGGATTCCCGATCGTTAATACATAGAGTCCAATAAGCATATCTTGAGTTGGTACGGAAACGGGATCCCCAATAGCTGGAGACAAGAGATTCATATGAGAAAACATAAGTAAACGAGCTTCTGCTTGAGCTTCCAAAGATAAAGGTACGTGAACAGCCATTTGATCCCCATCAAAGTCTGCGTTAAAGCCCTTACAAACTAATGGGTGTAAACAAATAGCACGCCCCTCCACTAAAATGGGTTGGAACGCCTGTATACCTAATCTATGCAGGGTAGGCGCTCTATTCAACAATACAGGATGCCCCTGCATCACTTCTTGAAGGATTTCCCATACAATAGGTTCTTTTTCCCGAATTTTGCTTTTAGCAATCCCTGTGTTAGAAGCAACATCTTGTCTGATTAGACCACGAATTACAAATGTTTGGAAGAGCTCTATTGCTATTTCTCGAGGTAATCCACATTGATGTAATGAAAGCAAAGGACCCACGACAATGACGGAACGCCCCGAATAATCGACCCGTTTACCAAGTAGAGTCTCACGAAATCTTCCTTCTTTGCCTTCAATTACATCGGAAAATGACTTGTAAACTTTATTATGACCATCTCTCATGGGTTGTCCGCGGATCCCATTATCAAAAAGTGTATCCACGGCCTCTTGTACCAATTTCTCCTGACACATTACTAATTCCCCTGGTGTAGATCTACTTGTTGCTAATAGATCGATAAGAGTATTGTTCCGATAGATGACTCTTCTATAGAGTTCATTAATATCCGAACTCATTAGTTTACCCCCGTCTATCTGAATGATTGGTCTCAACTCAGGAGGAAGAACTGGTAATAGGCACAAAACCATCCGTTCTGGGTCTACATTTGTTCGAATAAAATGTTTAGCTAATTCTATGCGCCTAACCAAAAAATCCTTTCTTCTTCTAATTTTTTTATCTTCCCATTCATTTCCTGCGGACTCTTCGTCTCCTAATTCCTTCCATTCTACCAAAGAATTATCTATAATAATTCGCAAATCCGAATCGGCTAATTGTTCTCTGATAGCACCTGCCCCCGTAGAGATTTCTCGGTTTCGAAATGTCTCGAAACCTTGAGTAGTAAAAAAGAGTGGGATGCTGTATTTCCAGGATTGGATTTCATATTCGAATGAACCTCGTAATCGTAAGAAAGTAGGTTTTTTAGCTATGGACCTAGCAAAAGAAAAATCGAGATAGGTTCCTATAGTATTGAATCCCCCCCTAACAATCGGACGTGAAGGTTTCCTCTCATCCGGCTCAAGTAGTTACACCAAATAAAGAAAGGGGTTCTTCTTCTGTTTAAACTTTGTTCGAGAAAACCCTATAAAAAGCTACTCTTTACTCAAGTTCCCAGTAAGGACCAGCCTTTCATTGATTCATTCTTATCTTATTTTATTTTTTATTTTCACTCTAACCTTTTTTACTTTCTTTTCTGTTTATTTATGTTTACGAAAAAAAAGTAAATGTGAAATTATTGAGTAGTCTACTTCCCCTCAAATGATGAATCCCCTGAAAGGAATATTTTGGGACTCGTAAAGGATTTATTTGTCTATATATTGTATTGTTCCATTCGATCTTTTTTAGGTCTCTACTCACCTCGATGGTTATGTGCCATGATATCCCTTGAAGCATATATGCGATATATAAGCTCCCGTAACCATGCCATATTCGCTTGCGCTTGCCTGAACAGAATTTCTTTCTCAAAGAAATGGAATGTATAATTCCACAAAAAGTATTTTTTCACGAGGTATGACTAACTATTCCTATATTATCTGTTACGGAATCGACCATGGATCAATCCCCCTTTTCTTCCATTTTTAATTCTTGAATGCACCTATAATTCTGAGCTTCATGTTCCTCCTCCCAAGATACATGTCAGAGCCGGGGGCATCCCAATCAGATTAAATGGGATGACAGTTTCTCAGTCCAAATCTGTCAAATGAAAATTTCGATCAAATCACACATCGCAATATACTAGGCCCTCTAATTCCCTAAGGGGCTTATCTAAAAGATTCGCAATATAACTAGGAAGACGTTTCAAATACCACACATGAGTCACTGGACATGTCAGTTTGATGTATCCCATTTGGTACCTTCGTACCCGAGAATCAACAAATTCCACCCCGCATTCTTCACAAGATTTCGGGTCTTCTTTTTCAGCCCCAATCCCTCGGTAATTTCCACAAGCACAAATCCCACTTTTTATGGGTCCAGAAATTCTTTCACAAAACAATCCATCTTTCTCCGGTTTATTAGTTTTATAATGAAAAGTATAGGGTTTTGTCACCTCTCCAACTATTTCTCCATTGGGTAGAATTTTTTTTGCCCAAGCCTTGATTTGTTGAGGGGAAACTGGTCCAATTCGAAGTTGTTGATGTTTATACTGGTCGATCATAGAATAGAAATTCTGATTCATTGAGATCAAGCTTCCTTCCTATCCATCTGGAAGTTCTTTTCAGATACAAGGAAATGATTCAGTTCCAGGGACAAAGATCGTAGTTCTCGAACGAGCAATCGAAAAGATTCTGGAGCACCCTCTGGGTTAGGTACTGCTGCTCCAATAATCGTAGCACCAAGTACTTCTTGACGAGCTCTAATATGATCAGATTTAGAAGTAAGCATCTCTTGTAAAATATGAGCAACACCAAATCCCTCTAGAGCCCAAACTTCCATTTCTCCTACTCTTTGTCCCCCTTGTTTGGCCCTCCCTCTAAGGGGTTGTTGTGTAACAAGTGCGTAATGCCCACTGGAACGTCCATGGATTTTATCATCAACTTGATGAATTAATTTTAGGATATAGGACTTTCCTATTAGAACAGGTTGTTCAAAAAGATCTCCTGTTCTTCCATCAAATATTCTGCTTTTTCCCGGATATTCGGGTTCAAATACCCATGGATTTTTTGTTTGCTTACTGGCTTCATATAATTCAGAAAACACTAGTTTTCTTGAGGCCTCTTGCTCATATCTCTCATCAAAAGGTCCTATTCTATAATGTTTCTTTAGCAGATCCCCCGCTAACCCGAGCGAACATTCAAATATCTGTCCCACATTCATTCGTGAGGGGACTCCTAATGGGTTGAATACCATATCAACGGGTGTTCCATCTTGCAAATAGGGCATATCTTGTCTAGACAAAATCTTAGAAATTATACCCTTATTCCCATGTCTTCCAGCTACTTTATCACCTACTTTGATTTCACGTTTCTGTGAAATATATACACGAATCCTTTCTGGATTAGAATTGGAAACCCCCTTTCTATGGATCCATCTCACATCAATAACTCGACCCCTTCCCCCTATAGGTAGTCTGAGAGAAGTTTCTTTTGAAGTGGATACCTGAATGCCAAGTATAGCTCGTAATAATCTATCCTCCGGGGCATATGACGATTCGCTCGCTGTCTGAGGTGTTAATTTACCTACTAAGATATCACCTGTTTCTATCCAAGATCCCAGCATCACAATTCCATTTCTGTCTAAATTTCGGAGTAAACGAGCCTCTAAATGCGGGATCTCCTTAGTGATTCTTTCAGGACCTTGGCTTGTTACATGAGTCTGAATTTCATATTTCCGGATGTGAAAAGAAGTATAAATATCTTCATAGACCAGACGTTCGCTAATTAGTACTGCGTCTTCAAAATTGTAACCTTCCCATGGCATATAAGCTACTAATACATTTTTTCCTAAAGCGAGTTCCCCACCAGCTGTAGCCGCACCACCCGCTAGAATTTGTCCCTTTTTAATGTATTTACCCCGCTGAACCTGAGTTTTTTGATGCATACAAGTATTTTTGTTGGAACGTTTATACATAACTAATGGAATGCTTAGAGTATCCCCATTACTTGAGAAAATGATCTTTTGAGTATCAGTAGAAATGATTTTTCCCTTGCGTTCGGCTATAGCTGAAATCCCCGAATCTAGAGCCGTTTGGCCTTCCAACCCAGTTCCAACAATGCACTTCTCGGACCGAGAAAGGGGAACTGCTTGGCGCTGCATATTAGAACTCATTAAAGCTCGATTCGCATCATTATGCTCGATAAAAGGAATGAGGGAAGCCCCAATAGAAAAATATTGGAAGGGAAAAATGCTTCTAAGATGAATCTCTTCCCATGCAATACTCAGGAATTCTTGACGATATCGAGCTGGAACAACCTGTTGTTCTTGAATACCCCGATTCAAGGCCAAAGAATTTCCTGCTGCTACCATATAATATTCATCTCTATTTGGTGATAAATAAACCATCTGTGCCTCTTTTGATCTATCAGATAATTCATAAAACGGACTCTCTATAGATCCCCAATAACCAACCTTCACATGAGTAGCTAATGATCCAATAAGTCCAACGTTGATTCCTTCGGACGTGTCAATTGGACAAATACGTCCATAGTGACTCGGGTGGATATCTCGTATCCGAAAACTAGCAGTTCGCCCCGTCAATCCTCCAGGACCCAAATAACTCCATTTTCGCCCATGAACAATTTGTGTCAACGGATTAGTTCGATCCAAAACTTGAGATAAAGGGTGTAGGCCAAAAAACGATTCATAAGTAGTTGTTAATGAAGTTGAAGTTACCAAATTTTGAGGAGTCGGTATCAATTTATGCCTGATTGCTCCACATATAGTTCCTCGAACCGTATTTTCTAAACGAACAAGAGCCAATCCGAATTGATCCTGTAATAGATCTGCTACAGAACGAATACGTTTATTTTTCAAGTGATTCATATCGTCAAGTGTACCCATTCCCAATTTCATTCCAATCAAATGATCCACAGCAGCCAATAGATCTCGTGGTAACAAAAAAGTATTGTTTTGGGGTATATCAAGATTAAGTCTCCGGTTCATATTTCGTCGACCAATCTTTCCTAATTCACATCTTTGTTGAAAAAATTTCTTTTGTAATTCCTTGCATAAGGACTCAGAAAATACCGGATCCCCCCCTACACAGGCAAATTGTTGATAAAACTCCAAAATAGCATTTTCTTTTGATCCAATCTTTTTTTTCTCTTTATCATTTGGGAAAGACAAGAAAATTTCAGGGTAACAAACATTCTCTAGAATTTCTCTTATATTCGAACCCATAGCTGATGATAGAACTAGAATAGATATTTTTTGTTTTCTACTTACACGGGCCCATATCCTTGCTTTTCTATCAATTTCTAATTCCGACCTTCCCCCCCAATCCGATATTATAGTACTGGTATAGACAGAAATTCCGTTATGTTCCAATTCTGAACGGTAGTAAATACCGGGACTTTGCAATATTTGGTTGATCACAATTCGGTATATTCCATTTATTATAGAGGTTCCAAAAGAATTCATTATAGGGATGTTTCCAATAAAAACGGTTTGTTCTTGCATATTTCTACCGGTTTTCCAAATTAAGACCGCGGGTACATATAATTCAGAAGAATATGTGAGTGATTCATACACAGCATCTCTTTCTTTTATCAAGGGCTCTACCAATTGATATGTTTCCACAAATAATTTAAATTCAATTTCTTGATCTCTATCTTCAATTTTTTGAAACTTATGAAATTCTTCCGCCAAGCCCTGATTAATGAACCTACAAAATCCCTCAAATTGTATCTGGCTAAATCCAGGTATTGTGGACATTCCCTCATTTCCATTCTGGATCATCTTAATCTTAAGTTTCCTCTTTATTGAAAAAATCCCATTATTGGCTTGGCTCACTATTCATCGAACCCTAACGATTGATCTAGCAATTATGGAATGGATATTCTGTTTACTGAATCACATAAAATTTTAGTCAACTCCATCCATATATATCTATATCATACGTATGAACGGAAGCAAGACAGAGAAATGTAGGGCATTTTTGATGCAAGTTCGAATTTGAGCTTGAGCCAGGTACAAATAGAAAGAAATGGAAATTTATAAAGCATTCCCGGGAAAAATTCTGTCACTTAGGCTGATGGAGTCTTTTATCGGATATCAAAATTGATCCAATTTATACCTAATTCTTTTATTATGATATTATGATTAGGGTGCAAAAAAATAAAAAATCAATGAATTTAGGATTCAATCTGCTCTCTATGAATGAGATAAAAACAGAAGAATCAGGAACAGCATAGAGTTTCCCTTTTTTTAGCACACACAATTGAATGTTCAAAAAGGAATTCTCAAATCTTTTTTTGGTAGTATAATTTCTAAAATACAATGGAATTGCGTACGTATATAGTCATAGGAGTAATCTTTAGGAAGTACATGCTAATATAGCTATCCGTATATCACATCTTTTATCATAATTGAACTTGGTGCAACATAGGTTAGGTCGCACTCTATACATAATGTCTATCTTCTATTCATATTCAATATTCAATGAAATATTAAAGCACGATGACCCTGATCCTATATAGGGATATGTGCATAAGAAATAGACCTGGGCTCGGTATCCATGTATAAAAATTTCTGTTCTGGAGTTTACACTGTTCTGGGGTTTACATATACACATATATTTTCTTATAATTCTAATTAATAATGTAATAGATAATGATTAGTCGTAAATCAATTGGATTTTGCATCCATTAAGGTAATACAAATGGAGATACAAAATTAAGAGCTGTTCACTAATTCAAAGTAAGAAAAGTAAGTAAGAAAAGTAAGTAAGAAAAGTAAGTAAGAGTAAAAGATTAATAAGTAAATAGTTAATGAAGTAAAGAGTTAATTATTCTAAATTGCCCTGCATTTTACAGTCTGTGACCGGGGCCGGGAATCTTTTCACTTTTCTATAGAAAAGTGAAAAGAGAAGGTAAGTTTTTAAGCGACGCGTGTTTTGAGATAAAATCAATCGAAGAAAAGAATAGAAACAGGATCCAATAAAATTTGGAAAAAAAAATAAGTACAATCGGATTCAAGATCCAAAAATAAAAGAAATTAAGAAAGTAAAAAATTAAAATCAAAACAAAATGAGGAGAGGAAAAGAAATAGAATAATAATATAATTAGAAATAATAGAATTATATTATTATAGATTATAGAAAGAGAATATAAGTATAAGAATATATAATATATATAATTCTATTATTTCTTTATCCATTTTGGATGGAATTTGGCGGCATGGCCAAGTGGTAAGGCGGGGGACTGCAAATCCTTTATCCCCAGTTCGAATCTGGGTGTCGCCTGATCAACAAAAAAATACTTGGAATTTTTTGATCGAACTTGACGAATTCTTGCCCCGCAAAAGCATAGGCAAGGGCTAGGTCTATTTATACTTGATTTTGAGAACCCGTAGGGCCTATAAAAGACTGTCATCTTTTTTCTCAAAATCTGGGTTCTGAGTGTGGCTCAAAAAGGTACCAATAAATCTGAAGCAACCCAATCTTATTAATGATTGGTTTGAGCTATTCTGATTATGAGTGGATTCATGAAATTCTTTCATAAAGAGCCGTAAGTAAGAATCCTATTTTGACTCTGCACCATTGATTCCACTATGATTATGAATCAATAATGGAATAATTCCTTCATTTCATAGAGATAGGGGACATCATTCGCATGGATATAGTAAGTCTCGCTTGGGCTGCTTTAATGGTAGTGTTTACATTTTCTCTTTCACTTGTAGTATGGGGAAGGAGTGGGCTTTAGAGCTAGGAATATTACTAATTTAGTACTTTACTGAAAAATCTACTTGTATCAATTGTGATCGTTTTGCGAAAGCTTAAAAAAAAACTTGACTTGCTTTAGTTTATCTATATATTATTTCTTAGATATATCAGTAGTATTATATTATTAGATTAGATTTCTACAATTCTCTAATATATGATATGGTATTTATATGGTATATAGTATATGCCCGTACTATTCTTCCTACATTAATTCTTTCGATGGGCGGATCCATAAGCATAAGAAGAGATATAAAAAATCAGGAATTAAAGCAGTTGACTTGCAATTCTTTTGGATTAATTGAAATGCATACAAATGGGTGTATAGAAAAAATAGAAAATTCGAGTGCTATTTCATTTTGATGTACGCCTAATATATATTATTACTTAGATATAGATATCTATTGTAAATTGATCTATATAAGAGAAAGCTTCTTTCTGTATACAATACAACTTTATGTTTTATGTACTAAGAATATGAAATATTCTACAATACTCAATTGTATAGTGCGGTAGAAAGAGCTATATATAGCTCTTTCTACCACACTATATCAAAGACTTCTGATTCCACATTTCATTTAAGACTTAAATAGAGTTTTAAACCCTTTCATTTCTTCAACATTTCTTCAATCATTCATAAAAATGAATAATTAAAGTTTCATTCAAATTAATCATTTTGGCTGACTGTTTTGACGTATATGATAAGTAAAAAGGCAGTAGGAACTAAAATGAACAGCGCAGTAGCAATAAGCGCAAGAATATTGACTTCCATAATCTCTTTGTTTTCTTCTTTCACAATAATTCGGGATCTAATCCCATAGAGATGATAAAGTGGACTCCTATCAATTCAAAGGTATGAATTGCATCTTGATGATACTAACAATATTATGAATAACAATATCAAATCAAATCGATTTATCGTCGCGAATTGAATAGTATAACATAGTATAACATAGGAAGATCTTTTATCCATACTCAATCTAAATTAAATAGAAAGAAAGAAAAATAGGATTCTTTCTTGTTATTGGATCATAAATCCCATTTCATTTTCCCGCTTTTCCACTTTTCCTTTTCTATCACCTATTTTTATACACTTATCCAATTCTTATTCCTTTACTTATATAAGTAATATACATAAAATTCTGAAGTCTCATATGTCCAATATTCTATAGGTCATATAATATATACAATATGCAAATAAAAAAAAAAGAAACAGTAGTAGAAATTATATGGAAAAAAGAAAAGGACGGATGAAGTATCAAAAATAGAATATTCCAAAAGATTCCTTCAAAAGGGGCGCTGCTTGGTTGGTATTTTATTAGTATCCTCCTTCTTTCCTTCTTGGATTGGAACTAGAACACATACATAAAAAATGCAGTGTGCAATTTGGATGAGAATAAGATAGATTGTTTTCAATTAGTCATTGCGGATGCACAAACAAGGTTTGTTCGAAACTAAAGAAGATGTGGTTTCATCTGAACCCAAAAAGTACTCTGTCGAGATAATTATGTGAAGTTCATTGTTTTGTTTATAATAAAGGAAGACAATTTGTGTCTGTATTCTCGGTCAAAATATGGGCACTCTATTCCATTTCGTTGATCACGAAAATAAAAATTGAAAAAAAAAAATAAGACGAGTATGGTCGCTCTTCAAATACTGAATAGAGTCTGTCTTACTCTAGTAATTAGTAAGAAGTAACGATTTTAAAAATCTACATATGTTTCTCCCTTACCAATCGGTACTAGTATAAGAAATCAAAATTTCCATATTTGTCTGATGAGAAATGCGAAACGAAAACAAAAGAAATAAGGATTCCCCTCAGAGATTAGATTTCGTTCCCCGTCTTCCCTTTCACGGAAAGGGGAGAGATGAGTTGATCAATTCGTCGGATCGGGACTGACGGGGCTCGAACCCGCAGCTTCCGCCTTGACAGGGCGGTGCTCTAACCGCTTGAACTACAATCCCAGCCATATGCATGGCATACATAGTCTTATGATTTAAGAAGAGCATTCTATTTGTCGTGTTGCAACAGAAAACAGAAACACGAAATATCCTATTCACATAGATAGATATGGACTTGAGTGAGAGTGGCATAGATTACTAGTAATCTATGTTTCTTGTATTTACTGTATTGCAAAGGAAAATAAAAAGAATGGATGAGAGAAAATGGACTATTTTTCTTTATTTTATACGGATCCGGGATTTCTGTTTCTAGAGGACAAATTGTTTAGATCTTTTTCATGGAGCGACGAATTCTTGGGCCGAGCTGGATTTGAACCAGCGTAGACATCTCGCCAACGAATTTACAGTCCGTCCCCATTAACCGCTCGGGCATCGACCCAGGAAGAATGAATTCTAGGTTTATTGATAATTCATGACCAACTTCCTTTCGCAGTCCGCTACCCCCAGGGGAAGTCGAATCCCCGTTGCCTCCTTGAAAGAGAGATGTCCTGAACCACTAGACGATGAGGGCATACCCGCCCCGACTGTCATCATACTATGACAATAGTATGAGTAGTTTTTTGGAATTGTCAATATATATAATTAGAATCAAATGTATGACTAGATCCGAGGAGTCTTTCCTACTTTTATGTTATGATCCCATAGAATTCTTTGGATTTGATGGTTCGTTCATGAATGAGCTATCCCATACTACCATAAATATTCTATAAATATTCTATCCTAGAACTAGATAGAACTCTATATCTATATAATAGAAGTATATACTATATATGATTATGATAATATATATATATATGATATGAAATATATATCTTTCATATCATATATATATGATATATATGATATGAAAGATATGAAAATCTATTCAATAAAAAAAGAGTTCAATATTAAATTCCATTTCAATAATGAAATATAACGAAAACGAAACAAAGTATAAAATCCCTTCAGCTTAGGGAGTTATTAGTCCGACAGAAAAAAGAGTCAAACTCTCATTTCTTTTCTTCAATTTGAACTCATTGCTTCGTTCAGCGTTCAGGTGAGTTATGCCTATCGCTATCTCACACTAAGCAAAAAAGGATTTAAACATTTTCTTTTTATAAGAATTCAGTTCAGGTTACGAATCCCCCATCACATGATTCAAGAACTTGAATATATGATGGAAAAGTCAACAAAGCAAGTAGGGTTGACCTTGAAACAATTCAATGCATTTTTTTTTTATTTTATTTTTTATCAAAATATGAATCTTACCCACTTCCTATATAAATCAAACTTATTGTTCCATTATGATATATATGCAATAGACTCATAATGGAAGATGAAGATGCCTAATTCATGAATTGAAAAAAAAAAGGCCCTTTTAACTCAGTGGTAGAGTAACGCCATGGTAAGGCGTAAGTCATCGGTTCAAATCCGATAAAGGGCTTTTTCCACTAAACTCAAATCTGAGTCTTCGTTTTTCAGCGGGGAATAGAAATCTTTTTGATATTTATAAAAAAAAAGAGAACGACCACCATTATGATTAGAATGAGTTCTGATTAGGAGGAGTCTTCCCCGTAATGACATAGTCATCCTCTTCATGTCTCATTATTCCATTTTTTTGTCCTGAGACATAAATATCCAACCCCTATTATGAATCGCCAAACCAAAGTATTCCTACTTCGCCATTGTTCCTATCAAAACCACCGCAAAATTGGAAATAAATAAAAAGTAAGTGGACCTGACCCGTGGAATCATTACTATATCAACTACTCTGATATTTAAATTCGATATAGATTCAATTGTAGAAGTGAATTTTTTTAATTCCTTATACCATTGGACCACACAAGTCAAAAATTTGTTTTTGATTTCATCTTCTTGTTACTGGATGTTCCATAGAACTAAATCGCTATTCTTTTCCTTTCCGCTACATAGAAAATAAAAATCTCTTTCAAATATGGATTTTCAATATCTTTCCTTGATTTCAGAATCAGATATTGTTTTGTTGTTACACCGATGGAATAGAGAATAAATGCAATAACAATAATAAGGGTTTTTCATTTCTAGTCTACCATTATTTAATATTCAATTTAGGGACAGAGAAAATCGGGAATCTGTGGATAATTCAGGGTTCCAATGGTTAGATTCTTTTTCTTTTCTATTGGACCGCAAACCGGCCTAGTTTATGAGTTCGACTCGATTAGTCCTTACGATGTACTATATTTTACTATTACTATAGAAATGAAATAAAGAATAATTTGGATCTTCGAACCCATTGGAAAAGGGCATTGAACGAAGAATCGTCCATAGATAATCAAACTACCGCATGCCCTGGAAATGAGATGAAGTGTTCGGAAATGGTTGAAGTAGTTGAATAGGAGGATCACTATGACTATAGCCCTTGGTAGATTTATCAAAGAAGAAAATGATTTATTTGATATTATGGATGATTGGTTACGGAGGGACCGTTTCGTTTTTGTAGGCTGGTCCGGCCTATTGCTCTTTCCTTGTGCTTATTTCGCTTTAGGGGGTTGGTTCACAGGTACAACTTTTGTAACTTCATGGTATACCCATGGATTGGCTAGTTCCTATTTGGAAGGTTGTAATTTCTTAACGGCTGCGGTTTCTACTCCTGCAAATAGTTTAGCACATTCTTTGTTGCTACTATGGGGTCCTGAAGCACAAGGAGATTTTACTCGCTGGTGTCAATTAGGCGGTCTGTGGACTTTTGTTGCTCTGCATGGTGCTTTCGGTCTAATAGGTTTCATGTTACGTCAATTCGAACTTGCTCGTTCTGTTCAATTGCGACCTTATAATGCAATTGCATTTTCTGCTCCAATTGCTGTTTTTGTTTCTGTATTCCTGATTTATCCACTGGGTCAATCTGGTTGGTTCTTTGCACCCAGTTTTGGTGTAGCAGCTATATTTCGATTCATCCTTTTCTTCCAAGGGTTTCATAATTGGACGTTGAACCCATTTCATATGATGGGAGTTGCTGGAGTCTTAGGCGCTGCTCTGCTATGTGCTATTCATGGCGCCACCGTAGAAAATACCTTATTCGAAGATGGTGATGGTGCAAATACATTCCGTGCTTTTAACCCAACTCAAGCTGAAGAGACTTATTCAATGGTCACTGCTAACCGTTTTTGGTCCCAAATCTTTGGGGTTGCTTTTTCCAATAAACGTTGGTTACATTTCTTTATGCTATTTGTGCCAGTAACCGGTTTATGGATGAGTGCTATTGGGGTAGTTGGTCTGGCTCTGAACCTACGTGCCTATGACTTTGTTTCCCAAGAAATCCGTGCAGCGGAAGATCCTGAATTTGAAACTTTCTACACCAAAAATATTCTCTTAAACGAAGGTATTCGTGCTTGGATGGCGGCTCAGGATCAGCCTCATGAAAACCTTATATTCCCTGAGGAGGTTCTACCCCGTGGAAACGCTCTTTAATGGAACTTTAGCCTTAGTTGGTCGTGACCAAGAAACCACCGGTTTCGCTTGGTGGGCCGGGAATGCGAGACTTATCAATTTGTCCGGTAAACTACTTGGAGCTCACGTAGCTCATGCCGGATTAATCGTATTCTGGGCCGGAGCAATGAACCTATTTGAAGTGGCTCATTTCGTACCAGAGAAACCCATGTATGAACAAGGATTGATTTTACTTCCGCACTTAGCTACTCTAGGGTGGGGAGTAGGTCCGGGGGGAGAAGTTATCGACACCTTTCCATACTTTGTATCTGGAGTACTCCACTTAATTTCTTCTGCAGTCTTAGGCTTTGGCGGTATTTATCATGCACTTCTAGGACCTGAAACTCTTGAAGAATCCTTTCCATTCTTCGGTTATGTATGGAAAGATAGAAATAAAATGACTACAATTTTGGGTATTCACTTAATTTTATTAGGTATAGGTGCTTTTCTTCTAGTACTCAAGGCTCTATATTTTGGAGGCGTATATGATACCTGGGCACCCGGAGGGGGAGATGTAAGAAAAATTGCCAACTTGACCCTTAGCCCAAGCGTGATCTTTGGTTATTTACTAAAATCTCCTTTTGGGGGAGAAGGATGGATTGTTAGTGTGGACGATTTAGAAGATATAATTGGAGGACATGTTTGGTTAGGTTCCATTTGTATACTTGGCGGAATTTGGCATATCTTAACCAAACCTTTTGCATGGGCTCGCCGTGCATTTGTATGGTCTGGCGAGGCTTACTTGTCGTATAGTTTAGGTGCTCTATCTGTTTTTGGTTTCATCGCTTGTTGTTTCGTCTGGTTCAATAATACCGCTTATCCTAGTGAGTTTTACGGACCCACTGGGCCGGAAGCTTCTCAAGCTCAAGCATTTACTTTTCTAGTGAGAGACCAACGTCTTGGGGCTAACGTGGGATCCGCCCAAGGACCCACTGGTTTAGGTAAATATCTAATGCGTTCTCCGACCGGAGAGGTTATTTTTGGGGGAGAAACTATGCGTTTTTGGGATCTTCGTGCTCCCTGGTTAGAACCTCTAAGGGGTCCCAATGGTTTGGACTTGAGTAGGCTGAAAAAAGACATACAGCCTTGGCAAGAACGCCGTTCGGCAGAATATATGACTCACGCTCCTTTAGGTTCTTTAAATTCCGTGGGTGGCGTAGCTACCGAGATCAATGCAGTCAATTATGTATCTCCTAGAAGTTGGTTAGCTACCTCTCATTTTGTTCTAGGATTTTTCCTATTTGTGGGACATTTGTGGCATGCGGGAAGGGCACGTGCAGCTGCGGCGGGGTTTGAAAAAGGAATCGATCGTGATTTGGAACCTGTTCTTTCCATGACCCCTCTTAGCTGAGATTTTCTTATTTATATCTATATATCTATTTGTCTATTTTTATTTTTTCTGTTTTGGCTCGGCTATCTCGCCTAGCCGAGCCATTCCCTTTATGAAAAAAGGGGCCAGTCCAAACGAGTAAAGAAGTAATTTCTTAAGTAAGCAAAAGGAGAGAGGGGGATTCGAACCCTCGATAATTCCTTGTTAAGAACTATACCGGTTTTCAAGACCGGGGCTATCAACCACTCAGCCATCTCTCCCAAATAGAATCTCTATTTTCTTCCTACGAATAGAACATGACCCTATGAGATGATACATTAACTAGTTGATCGATTCCATGTCCGAATAAAAAAAAAAGAAAGCGGTAATAAGTTCTAATAAATCAATTCATTCATGTCAAATCCCTTCATGATGCATTTTTGACAATTTTTACTAAGCGAGGGATCCAATGGTATAGTTCATTTGTTGGTAGTTTGGAGGATTACAAACATGACTATTGCTTTCCAATTAGCTGTTTTTGCATTAATTGCAACTTCATTAATCTTACTGATTAGCGTACCCGTTGTATTTGCTTCTTCTGATGGTTGGTCAAGTAACAAAAATGTTGTATTTTCTGGTACATCATTATGGATTGGATTAGTCTTTCTGGTAGCTATCCTGAATTCTCTCATCTCTTGAACTTCTTTGGTATTTCCCCGATCCAAAAATTACATTTCATTGTTATGAATTGAATATAGATAATTAAGAATCTGATCTTAATTGGAATCTTACTTTACTTACTAGATAGAGTCTTTGTATCTGGCCCTGCGCAAATATGATCCAGACGCATATATGATATATCATATATGTCATATATGTGTGGACATATGCGTACGTATAAGGAACGAAGAAATGCGGATATGGTCGAATGGTAAAATTTCTCTTTGCCAAGGAGAAGATGCGGGTTCGATTCCCGCTATCCGCCCATGGTGAAATAATGTACTATAAAAAGATTCCGTCGATATAGTTGACTACTATGCATACTAGTATGCATAGTAGTCAACTATAGTAGTTCTATCTTCCGCTTCACCTTTTTTCCTCCCAACAAAAAAAGTCATTAATTACTACAAATACAAAATAAAAGGGAATTTCACAAAAATGTTGCGGAGACAGGATTTGAACCCGTGACCTCAAGGTTATGAGCCTTGCGAGCTACCAAACTGCTCTACTCCGCGCTGAAAAACTGGGAACTGATGGACGAAGAAAGGTTGGATATGCCCCTCTACCATATCTATACAAATAGAATAGTCTATTTATACAAAATGGTAAAGGGGGCCTCCTCTATTCTATAGATCATAGAGATCTATCCAAATAGGAAACAAGATTTTCTCCTTACCAACTTGATCTTGTTGCGCCTGGTAACAAACATGCATAAACCATTTCTCGAAGTATGTGTCCAGATAGTCCAAAGTCTCGATAGTTCGCTCTAGGTCTTCCGGTTAAAAAACAACGTCGATGAAGACGTATAGGCGCACTATTACGTGGTGAGGATTGCAATTTTCCATGAATTTTCCATTGTTCCCTCAACGAGGGAACTTTGCTTATTTCTTTTTTTAAGGATTGACGAATCCAATAATATTTCTGTTCCAATTTCTGGCGCTTCTTCTCCCTTTGAATCAAACTTTTTCTTGCCATAATTTTCAGTTCCTATTTTTATCAATTATACAGTTCGGATCCTAGATGGAAAAATAGAAGCAAGGTACATACCTCCTCTTCATCGAAAGAAATGAGATTTTTGTTTTTGGGGGTTGGGGTATAGGGTACAAAAAAACAAGAATTAACTAAATTAACCAAATTTTCCTGATGTGGAGGCAATCAAGAAAGCTGCATAAGTGAATATATAACCTACGGAAAAGTGGGCTAATCCAACCAATCTTGCTTGCACAATGGAAAGAGCCACTGGCTTATCTTTCCATCGAATCAAATTAGCCAAAGGTGTCCGTTCATGAGCCCACGCTAAAGTTTCAATCAATTCCTGCCAATAACCACGCCAGGAAATTAAGAACATAAATCCAGTAGCCCAAACAAGATGTCCAAATAAGAACATCCACGCCCAGACCGATAAACTATTCATACCAAAAGGGTTATATCCATTGATAAGTTGTGAAGAGTTTAACCATAGATAATCTCTTAACCATCCCATCAAATAAGTGGAGGATTCATTAAATTGTGAAACGTTACCCTGCCACAATGTGATGTGTTTCCAATGCCAATAAAAAGTAACCCATCCAATGGTATTTAGCATCCAGAAAACTGCCAAATAAAATGCGTCCCAAGCAGAAATATCACAAGTACCGCCACGCCCGGGGCCGTCGCAAGGAAAACTATAACCGAAATCCTTTTTATCTGGCATTAACTTGGAACCACGCGCATCTAAAGCACCTTTTACTAAAATCAATGTAGTCGTATGCAAACCCAGAGCAATAGCATGATGAACCAAGAAATCTCCCGGCCCTATTGTTAAGAAAAGTGAATTACTATTCTCATTAATAGCATTCAACCAGCCAGGTAACCATATGCTTCGACCTGCATTGAATGCCGGGCTATTCGTTGAAGATAAGAGTACATCGAACCCATACGAAGTCTTGCCATGAGCAGATTGTATCCATTGGGCAAATATGGGTTCGATTAAGATTTGTTTTTCCGGAGTACCAAAAGCAAGCATGACGTCGTTATGAACATAAAGGCCCAAGGTATGGAACCCCAGAAATAGGCTAGCCCAACTTAAATGAGATATTATAGCTTCTTTATGGTCTAACATTCTTGCCAATACATTATCCTCATTCTGTTCCGGATTGTAATCCCTAATGAAGAATATAGCCCCATGAGCAAAGGCTCCTGTCATGATGAACCCTGCGATGTATTGGTGATGAGTATATAACGCAGCTTGAGTAGTAAAGTCTTGTGCTATGAATGCATAAGCAGGTAAAGAGTACATGTGTTGAGCTACTAAGGAAGTAATAACCCCTAAAGAGGCTAGAGCAAGGCCCAATTGAAAATGAAGCGAATTATTTATTGTGTCATAAAGACCCTTATGCCCACGTCCTAATCGACCCCCCGGAGGAATATGTGCTTCTAAAAGATCTTTGATACTATGCCCAATCCCGAAGTTAGTTCTATACATATGACCAGCAACGAGAAAAAGGAATGCAATAGCTAAATGATGATGAGCAATATCGGTTAGCCATAAACTTTGCGTTTGGGGATGGAACCCCCCAAGAAGGGTTAGGATGGCAGTTCCTGCTCCTTGGGAGGTACCAAATAAATGACTACTGGAATCAGGGTTTTGAGCATAAAGATTCCATTGACCCATAAAAAGGGGGCCCAACCCTTGGGGATACGGCAATACGTCTAAGAAATTATTCCATCTGACGTACTCTCCCCTCGATCCGGGAATAGCGACATGAACTAAATGTCCTGTCCAAGCCAAGGAACTTACTCCAAAAAGCCCTGACAAATGATGATTGAGACGAGATTCGGCATTTTTGAACCAAGAAACGCTTGGTTTCCATTTTGGTTGTAGGTGTAACCAACTCGCTATTAAGGATATAGCAGAAAGAAGTAATAGAAAAAGAGCTCCAGTATAAAGATCCCCATTGGTGCGTAATCCGATTGTATACCACCACTGATAAACACCAGAATAAGCGATATTCACTGGACCGATAGCGCCTCCTCTAGTAAAGGCTTCTACAGCAGGTTGACCAAAATGAGGATCCCAAATTGCATGAGCAATAGGTCTTACATGTAAAGGGTCCTGTATCCATGACTCAAAATTTCCTTGCCAAGCTACATGAAACAGATTTCCGGACGTCCACAGAAAGATTATTGCTAACTGTCCAAAGTGCGAAGCAAAAATGTTCTGATAAAGACGTTCTTCAGTAATATCATCATGACTCTCGAAGTCATGTGCGGTAGCAATACCAAACCAAATACGACGAGTAGTGGGGTCCTGAGCTAAGCCTTGGCTAAATCTTGGGAATCTTAATGTCATAATGCCTTTCAAATCCTCCTAGCCATTATCCTACTGCAATAATTCTTGCTAAGAAGAATGCCCATGTTGTGGCAATTCCACCCAGAAGGTAATGGGTTACTCCTACAGCACGTCCTTGTACAATACTCAAGGCTCTAGGCTGGGTAGCAGGAGCAACTTTTAATTTGTTATGAGCCCAAACGATGGATTCAATGAGTTCTTGCCAATAACCACGGCCACTGAATAGAAACATTAAACTGAAAGCCCAGACAAAATGAGCGCCTAAGAAAAAAAGACCATATGCAGATAATGAAGAACCATAAGATTGAATTACCTGAGATGCCTGTGCCCATAAGAAATCCCGGAGCCATCCATTAATAGTAATGGAACTCTGTGCAAAGTTCCCTCCTGTGATATGAGTGACTACTCCTTGATCACTTATAGTACCCCAAACATCCGACTGCATTTTCCAACTGAAATGGAAAATTACTACCGAAATTGCATTGTACATCCAGAATAGACCTAAGAATACATGATCCCAGGCGGATACTTGACATGTCCCCCCCCTACCAGGTCCATCACAAGGGAAACGAAAACCAAGATTTGCTTTATCAGGTATCAAACGGGAACTGCGAGCAAATAGAACACCCTTCAGTAGGATCAATACAGTCACATGAATTGTAAATGCATGAATGTGATGAACCAAAAAATCTGCAGTTCCTAATGGGATAGGTAACAAAGCAACTTTGCCGCCCACTGCTACTAACTCACCACCTCCCCAAGTTAAGCTGGTACTTGTTGTTGCACCAGGAGCTGTTACGCTAGGTGCTAAAGCATGGGTGTTTTGTACCCATTGAGCAAAGATGGGTTGTAATTGTATAGCGGTATCTGAAAACATATCTTGGGGACGCCCTAAAGCGCTCATAGTATCATTATGAATATACAAGCCAAAACTGTGAAAACCTAGAAATATGCATGCCCAGTTCAGATGTGATATGATTGCATCACGGTGTCTAAGGACACGATCTAATAGATCGTTGTATCGAGTAGTTGGATCGTAGTCTCTTACCATAAAGATGGCTGCATGTGCAGCAGCGCCAACTATAAGAAATCCCCCGATCCACATGTGATGTGTGAACAACGAAAGTTGTGTACCATAGTCAATAGCTAGGTATGGATAGGGGGGCATGGAATACATATGGTGAGCTACAACAATGGTTAAAGAGCCTAACATAGCCAGGTTCAGAGATAATTGAGCATGCCATGACGTTGTTAGGATTTCATAGAGACCTTTATGGCCCTGTCCCGTAAATGGACCTTTATGAGCCTCTAAAATGTCTTTCAGGCCATGACCAATGGCCCAGTTAGTCCTATACATATGACCTGCGATCAGGAACAGAATCGCAATAGCTAAATGATGGTGTGCAATATCACTCAGCCAAAGACCCCCTGTTATTGGATCTAATCCTCCACGAAAACTCAGAAATTCCGCATATTTTGCCCAATTCAAGGTGAAAAAGGGTGTTGCTCCCTCGGCAAAACTTGGATAAAGTTGAGCCAAAAGATCCCGATTCAAGATAAATTCATGAGGAAGTGGTATTTCTTTAGGATCAACTCCAGCGTCAAGAAATTGGTTAATCGGTAAAGATACGTGGATTTGGTGTCCCGCCCAAGAAAGAGACCCAAGTCCTAGTAATCCCGCTAAGTGGTGATTCAACATAGATTCTACATCTTGGAACCAAGCCAATTTGGGAGCGGCTTTGTGATAATGGAACCAACCAGCAAAAAGCATTAACGACGCAAAGATCAATGCACCAATTGCGGTACAATAGAGTTGTAATTCACTAGTGATTCCAGATGCTCGCCAAATCTGAAAAAAACCGGAGGTTATTTGTATTCCTCGGAAACCACCGCCCACATCCCCATTCAATATTTCTTGACCTACTATTGGCCAAACTACTTGGGCACTGGGTGCAATATGAGTAGGATCACTTAGCCATGCTTCATAATTGGAAAAACGGGCGCCATGGAAGTACATGCCACTCAGCCAAAGAAAGATAATAGAGAGTTGGCCAAAATGAGCACTAAATACTTTTCGAGAGATCTCCTCCAAATCACTGGTATGACTATCGAAATCGTGAGCATCAGCATGTAGGTTCCAGATCCAAGTGGTAGTATCAGGACCCTTAGCTATTGTTCTTGAGAAATGGCCGGGTCTGGCCCATTCCTCGAAAGATGTTTTTATAGGATTCCTGTCTACAACAATTTTCACTTCGGGTTCCGGCGAACGAATAATCATTAAGTCCTCCTCTTTCCGGACAACACATACAAAGAGACCCGCCAACAGTCAAGTTTTTAGTGAAGATAGATATTCACTAGAGAAATTATGATATTGAAGTCGATCCGGGGCAAGTGTTCGGATTTATTATGACATAAATAATAGGTGCCGGGGGGACCTATTATCTTGCCAAAGCATTTTCCAGGATAACGAAAAAATGATTTTTTTGCAACCTAGCTTCTTTCTATTTAGATTCTGAATGGCGAAGTGCCCATATAGATTTACTTTAATCGAAAATTAAAAAATCTAATATAGAATCTAAATAGCTAAATAGAATATAGAAATCTAGATTCTATTTAGCTATTTTTTTTTATTAAGAATTATGAATGATTTGAATTTTAGACTTCATAAGATTCATCGAAATAGCTTTATTAGTTCTATGCTATATGGTATCTGTAGCATTTATCCTTATGAGATACCGTAGAAAATGTACAAAATCAAATGATTTTAGAAATTTAGAAAGAAGGGATATAATAAAATTCTTGATTGGATCTTCTAATAGGAACGATTTATTTAATTTGATTGCTGGATCAAAAAAAAATAGAATGGTCTTATTCAAAACGCCTCGTTATTTTTAACCAATTATGTGCTTCAATATAATTCCCTGGAGTAAGCGCTATAGCTTGTTTCCAATACTCAGCAGCTTGATCGAACCAAGCCTCCGCAATTTCCGAATCGCCTTGTAGAATGGCCTGTTCTCCCCGGTCGGAATAGGTTAGTAAATTCCCTCCCTTAGAACCGTACTTGAGAGTTTCCTACCTCATACGGCTCAGCAATCGATTATTTTTGCGTCCCATCTTCTCTTAATCTATGCTATACTAACTGAATTAAATTTCTCATCAATCTATTCTATTTTCTCTTGGGTTAACCAGAAGATGTTTATTGCATAAGTTTCCAATTCTAATTTGGATCAATGATTAGTTTTCTCTTTTCTCCCACCTTCAGAAGAATGAAGCATAGATATCTCCCGATATCGTTAGAATTTTCTGAAAGGTAACTATCTCGGTTTCATATTTCATATATGGTATATGAGATTTCTATTTATATAGAATCTTTGAAAAAGACTTTCCTCCGTTAAGAAAAAAGAACTTACTATCTTTGGGATCTGATGCTACACCGCTGCTCAATACTTTAGTAGATCGACTCTATTACATAAGTTGATTTCTCTCCCATATCATGACATAAGTAGACATAAGTAAGCAGTTCTGAACTGTATTTACCAAATAATAACTTACTAATGGATCTTTACGGTGCTTTCTCTATCAATTCGACTTTTTATCCATAGAGTATAGTATATAGGCCATACCCATTTCTTCCGATTTTTTTGGTTCTCGCGAAGTCTTTTTCCTTGCTACAGCTGATAAAAATCGTTACTTTGGACGATTCATATGTAGAAAGCCTATCTTTTTTCTAGTATTTACTAGACAATTCAATCTTTTTTTTCTTTCTATAGTGAAGATAGTCGCACGTAATGACAGATCACGGCCATATTATTAAAAGCTTGTGGTAAAAATGGATTTCGTTCTAGTGCTCGGAAATAATATTCCAAAGCTTTTGTATGCTCTCCGTTGCTTGTGTGTATAAGACCTATGTTATAGAGTATATAACTTCGATCATAGGGATCAATTTCTAGTCGCGTAGCTTCATAATAATTCTGTAAAGCTTCTGCATAATTTCCTTCGGATTGAGCCGACATCCGTTACGGTCGTTCATTCTAGTAAAAAAATCTCCGTTCCAGAACCGTACGTGAGATTTTCATCTCATACGGCTCCTCCCTTCTGTGCATAGTACTAAGAGGAATAATTCATGTAATCAAAATTTCACTATTCTCATTATGAACTGACAGGAGCTGGTATTTTTACAAGAAATTTCTAGCCAGCCTTCCCACAAGAGGTTTTTTCTTAACACCAATCATATTAGTGCTATTTAGAAATGGTAACTCCAAAGATTTCTTTGTACTTAACGCTTACGATTTCCAGGAATTAGTCACTTCAACGGTCTTTGATGGTTATACGGGTACCAAAAGTACGAATGAGATGGATCTTTGTTTTCCTAACCATTCTTTTTAGTTCCGATACCGATAAGGAAAAGGTTTCTTTATAACAAAGTTTTTGTGTTGTTGATTCCTAGGTGTAGTGCTTTTTCCCCGATGCCACCTATTGGTACTAAATGAAGTAGTAATGACCTCCAATACAGAACCTATAGATGTAACCTTTTGCTCAATACTAAAATCGATAATTGAAGCATCTAAGGCTGCATCAATCGAGGATACACGACAGAAGGAATTGCTCTATCTTTAAACTTCACCTTCACCAAGCGTAGGTTTATTTCACTAATTTGTTTTTTTTATATTCCTAACTACGTTCTTTTTCTCGTAAAACTGAGGGGTAAAAAAAAAACAAGAAAAAATCAAATCGCACCATCTCTGTAATAGGTAAATGCCTTTTTTTCTCCTGAAGTTGTCGGAATTATTCGTAATAAAATATTGGCTACAATTGAAGAGGTCTTATCAATAAAATTTCCATTTATTCGAGATCTAGGCATAATTAGCAATTCATTCTATAATTCTTCTCATCCCCCTTCGGGGAAAACGATCCCACAAAAAAAGGAATTGTACAGTACAAAATAACATAAAAACAGACTAAAATGCGGTGTACCCCTTTTGGACAAAGATGAAATGAAATAGTTGAATCAGATTAGATTTCATTCCAATTTCGTAGTATACCAATGACTATTACTATTTCATCTAAATTGAATAACCAAGTTTCCTATGGATTTTGATAACTCGAGAAGTTTTGATTTGGTTATGATCCAAAAAGGAAAAGAATGGAATAATCATTCCATGATAAAATCAAATGAAAATATTCAAATAAAGTAACCATCCTTTTTGTTTGCATAACGTGTATGTGCCACACCATACAATTGAAATAGAAAGATTCGTCGGACGATTCATGAATTCCATGGGTTAGCTGATGAAAGAAGTTGTTGTTGATAAATATGAAATTGGAAAAGAAATTTCTTCTTATGGAACCCTCGGGCGGTCATACATGTACTACAATTAAGATGAAGGACTCGCTATTCATTCGGGTTTTGGTCAAGAATAACATTCTGTAGGAGAGATGGCCGAGCGGTTCAAGGCGTAGCATTGGAACTGCTATGTAGACTTTTGTTTACCGAGGGTTCGAATCCCTCTCTCTCCGTTTCTTTTCATTCATCAACGTTACCGACTACAATGTATCAAATAAAATAAGAATTGATATCATTATTCTAATGATAAGACCCTTATTTAAGAATTTAATAGATATTCTCTATCCCTAATTAATCCCTGTGATAGGTAAAATACAAATATAAATATCATATTGATATTGAAAAAAAGAAAAAGAATCCTATTGCCGATCCTTTTTTGATACATGAATGAGACAGGGCACGAGGTGCTCTATTTACTTCAGCGAACGGAGTCAAAATTGGTATGAACCTTGCTTTTTTATTTTCATTAGAATCAAGTCTGACGGGAATAATATTCTACGACCAACAACTCATTTATTTTAAGACCGACCCATTTACTATCTATTATTTGATTGACAAATCCTTTATATTGTAAGGAGTCAATAGTCAAATGGTTTGGCAATTCCCCGTGGGGGGATGAAACAAGATAATTTTGAATCAGAGCTTTCGATCTTTCTTTATCCTTCGTAGTAATAATATCTCGGGGTTTGCAACGATAACTTGGTATATCCACTATACGACCATTAACTAAAATGTGTCTATGGTTAACTAATTGTCTGGCTCCAGGAATGGTCGAAGCCATACCTAATCGAAAAAGGATGTTATCCAAACGCATCTCAAGTAGTTGTAGTAAAACCTGGCCTGTTGACCCTTTGGCTTTTCCAGCAATATGCACATATTTAAGTAATTGTCGCTCTGTCAGACCATAATGAAAACGCAATTTCTGTTTCTCTTCTAAACGAATACGATATTGTGATCTTTTTCCAGAGCGCAATTGGGTTTGAAGATCACTTCTGGATCTGGGTCTTTTACTAGTTAGTCCCGGTAAAGCCCCCAGCCGGCGTATTTTTTTGAAACGAGGTCCTCGGTAACGAGACATATAAAGGCTCCTTTTTTATTCACTTTCATTTGACAAAAAAATATAAATTCAGACTGAACTAAAGGATCAGCAAAGCAAAACTCAATTTACTAAAGTCCTACAAAACAGAATAAAATAAATTTTATCAATATTCGGATTTTTTGTATATATAGGAAATTAAAGCGAAGGTTACATTTTCCAATTTCTTCTGTAGAGATCTAATTGTTCTAGTCAACTTTTTTATTCATAGCTATAGCTGCAAGTTACCATGACATAATAGATCGGTGACCCGACATTTAGAGAAAGCGAGAGTAGAGATTTTCAATCATGGAAATAAAAAAATTGATAAAGAAAAAGAGCCGGCTATCGGAATCGAACCGATGACCATCGCATTACAAATGCGATGCTCTAACCTCTGAGCTAAGCGGGCGGATATAAGAGCAATAGTGTATAGGAATACAGGAAACTATCGGATCTTAGCTATTACCTAGTGATTCTTCTTCTTTTTTTAATTTATTGATTATTGATGATTATTGAAATTTCTTATATTTATTAGTTATATATTTTATTATATATTTGAGATTCTATTTAGAAAATAGAAAAGAAAACTATTTAGATCTAGATAAATTAGATATCTAAATATAAATAGAAATTCAATTACATATTCATATATATGTGAATATAAAATATCAATATATAAATGAAATTAGGATTTGAAATGAAAAAAAAAGAATGAATATCGACCGTTCCACTATTACAAACTACACTGTGAAAATTAATGAGGAGGAAAGGCACATATATATGTGGGATATATCTATCCATATTGAATTGCGGATACATCAATGATAGAATCAATTTGGTATTGAAACAAATAGGGTTCATCCAATAGAGATGAAATGATAGAATATAGAATAGGGGGATATGGCGAAATTGGTAGACGCTACGGACTTGATTGGATTGAGCCTTGGTATGGAAACCTGCTAAGTGGTAACTTCCAAATTCAGAGAAACCCTGGAACTAAAAATGGGCAATCCTGAGCCAAATCTTTGTTTTGAGAGAAAAGATGGAAAATAAGAATAAAAGGGATAGGTGCAGAGACTCAATGGAAGCTGTTCTAACGAATGAAATTGACTACGTTACGTTAGTAGCTAAAACCCTTCTATTGAAATGACAGAAAGGATAACCTTATATACCTAATACGTACGTATACATACTGACATAACTCTATATATGAAAATAGAAATAGAAGATTTCTATTATATAGTTTTATTCTCATATTATATATATATTTATACTCATTGCTATATTCTATATTCTATTTATATTCTAATTAGTATAGAATATTTCTATTTATTTTATAATATAATAAATATATAATAAATATAATAGTAGAATTCTATTATGAAATAATAGAATAATATTTCTATATTATTTTATATATTCTTTATTCTTATTTATATTACTCTAAATATGAGTATATGAGTAATAGTATGAGATAAGGATCTATATAAACCCTCTATTTCTATTCTCTATTAATTAGAATGATAGAGATCAAAAAATCTATGAAAAATTGAAGAGTTATTGTGAATCAATTCCAATTGAAGTTGAAAAAAGAATCGAATTCAAATATTCAGTGATCAAATGATTCATTCCAGAGTTTGATAGATCTTTTGAAGATTAATCGGACGAGAATAAAGAGAGAGTCCCATTTTACATGTAAATACCGACAATAATGAAATTTATAATAAGAGGAAAATCCGTCGAATTTTTAAATCGTGAGGGTTCAAGTCCCTCTATCCCCAATAAAAAGCCCATTTTATTTCCTCACTCTTTATTTATCCTCATCCTCTTTCTTTTTTTTTCATCAGTGGTTCAGTTTAAACAAAATGAAATATCTTTCTCATTTCATTCACTCTGTTCTTTCACAAATGGATCCGAATCAAAATCCTCGTATCTTCTTCCAATCCAATCTCATTTGTTTTATATAGTACGATATGAACATATATATATGTTCAAGGAATTTACGTTATTGAATCATTCATAGTCCATATCTTTTTCCTTACATTTACAAAGAATGTCTTCTTTTTGAAGATCTAAGAAATTCAGGGGCTAGGTCCAATTTTTTAAGATTTTCTTTTTTAATTCTTTTCATTGACATAGATATAAGTACTCTGCTAGTATGATGCACGGGAAATGGTCGGGATAGCTCAGTTGGTAGAGCAGAGGACTGAAAATCCTCGTGTCACCAGTTCAAATCTGGTTCCTGACACGTGAATAATGTATCGGATAGATATTCATACCTCATACAAATGAAATTATTTCATTGAGACGAGATATTCTTTACTTTCTTTTTCATTTTTTTCCTCTCTGTTCATACTTTTCTTATTCAAAAAGTATGTTAAAATTTCGTATATATCTCAAATCTAATAGCTAAAAAAAATTAGCTAAAAGGATTCAATCAAAGATTGGAAGGATAGGAATAGAAAGGATATATTTCAGACATAGTACAAATAAACTCCGATTCTTCTCATTTTGCATTTCTTCATTTCGTCTCTTCTGTCTTTTCTTTCAAATTTTATATTTTTTTGTCACTCTTGCTCAAGTTACTTTCTGAGGTCCCCACTAAGTGATGTGCGAGGTACAAAGTTCATGGTGCAGAATCATCCTATTGTGCTCATACGAAATGTATATTATATGATATCTTCCCGATTGGGGAATAGCAATGAGAGCCTCTTTTTCTTTTTTTATTTTAGCCCCTCCACAATACGAATGAAAGTCCAGTTACTCTGTTTCATCTAGAAGGGGAATGCCAAGATGCTCATTGATTGATAAAAAACCCCTTTTTTATCAATCAATGAGCATCTTGAATTTCATAGAAATTGGGCGTAATATAGTCTTTACGTAAGGGCCAGCCTATCCAACTTTCAGGCATCAAGATACGTTTAAGGCGAGGATGATTTTCATAAGAAATTCCCAACATATCATAAGATTCCCGTTCTTGAAAATCAGCACTTCTCCAAATCCAAAAAACTGACGGGGTTTGAGGATTACTCCTGGGAGCAAATATTTTTATGCATACCTCTTCTGGTTTATCTATACCATACCGTATTTTCGTAAGGTGATACACACTAGCTAAAAATCCGCCTGGTGCTACATCATAGGCACACTGGGAGCGTAAATAATTGTAACCATATACATATGAAATGACAGCAATGGAATCCCAATCCTCGGTTTTTATTTGTAAAGTCTCTATTCCTCGGCAATCAAAGCCTAAAGATCTATGAATTAGCTCATGCTTGACTAGCCAATCAGATAAATTAACCTGCATCTTCTTCATTTCTCCCATATTTTTATTTGTATGAATATTTCATATTGACAATGAAATTGTTAATGATTGACCCGCTGTTTCTTATTCTGCACAAATGAACCCTGCCTGATTCACTAATTCGTAGGAAGATACTGACCTTTTGGATTTGAAATCTTTTTCAAATCCAAAAGGTATCTCTGAAGTAGATGGTGATTTATAGAGTAATCCTTGATCGTAATTTCCAGTATGAGTACTGCGTCGAAGGTAAAACTTGTGATTAGTAGTAAAACATCGATTCTCCTGTTGATACGCAGTTCTATCTTCAAATATTTTTCGGGATATTTTCTTACGAAGTTTCGTTATAGCATCGATAATTGCCTCTGGCTTAGGCGGACAGCCTGGCAAATAGACATCCACAGGAATTAGCTTATCGACTCCGCGAACAGTACTATAAGAATCAGTACTGAACATCCCCCCTGTAATAGTACAGGCTCCCATAGCAATGACATATTTTGGTTCAGGCATTTGCTCATATAATCTTACTAAAGAGGGAGCCATTTTCATTGTTACTGTTCCGGCTGTTAAAATGAGGTCTGCTTGCCTTGGGCTCGATCTTGGCACCAACCCATAACGATCAAAGTCGAATCGCGAGCCTATTAATGAAGCAAATTCAATGAAACAACAACTGGTACCATAGAGAAGCGGCCATAAACTGGAAAGTCTTGACCAATTCGAGAGATCATTCGATGTAGTTGAAATAATTGAATTGGGGGTTGTTTGGTTAAGTAATGGAAAATCAACCAAATTCATAACTGTTTCAATGTAATCCTTTCCTTCCCTTTTTTTTTTATTGTCTAAATATTCAGCTAAGACCATTCCAACGCTCCTTTTCGCCATGCATAAACTGAACCCACAATTGGGATAAGCACGAAAATGAAAGCTTCTATAAATACAGATACACCCAATACATCAAAGCTCATTGCCCATGGATAAAGAAAGACCGTTTCAACATCAAAAACAACAAAAACTAGAGCAAACATATAATAGCGAATTCGGAATTGTACCCAAGCATCCCCCATGGGTTCTATACCTGATTCATAACTAGAAAGCTTTTCTGGACCTTCCCTAATCGGGGCTAAAATCCCAGAAATGAAAAATGCCAAGATAGGAACAACACTTGATATTATTAGGAATGCCCAGAAAATATCATATTCGTGAAGCAGAAACATAAGAGTACTCCTATAAATGTGGATATGAATGTGGAATAGGCTTAATTCTTCCATTGGAATTGGAATTTTCAAATCATCTAGAATCTAGAACTTCTTAGATGAAACAATAAAATAATTTTTATCAAATAAAGCCGCATAGTTGAGAGTTTGTTTGCTGTAGGACATACCTTGTTTCAAGATTCATCTAATGTCATCCCACTTCTATTTTTTCTTTTTTTTTATCCTTTCGATTCTATTTCTATATGTGATATGTAGACATAGCATGCTCTTATACTTAGTTATTTTTATTTTAGATTCTCCTTATTCTTATACTTAGTTTATACTTATTATCTTATATACTTATTATCTTAGAAATCTTAGAAAGTAATTAGAAAGTAAAGACTTATCTTATGTATATTTATTTCATATTGATCTTATATCTTATAAATAAGAAAGTGAAAGTAAAGAATCCCTTATCTTAATCTTATAGTAAAGAAGAAATTCAATAATTAAGAATTCAATAAAGAAATTACAAATTCAATTTTCAATTCAATTAAAAACAATGAAAAAAAATAAGAAAAATAAATAAGAAAGATAGTCTATTATTTATATGATATAAATTTATATGATATGAAAATAGAGTACTAAAATCGCATTTTGTAATGAACCAAAATACTTGTTTGTTTTGTTACGACAATAAAAAAATTTCGTAAGACCAACCAATGAGTTAGATTTCGCTACAAGAAAAAGGTTTGTTGCTATAAAAATAGAACAAACCTACACAACGAAAGATAATACAAAGTGGTAGATTCGACAAAATAGTGAACGATCGACATAACATAAGAGACTCCTAATAATCCTTCTAATATTCTAATATTCTAATAGTTAAAAGTATAAAAGTATAATAGAAAGAATCACACATTATCGAACAATTCGACAGACAAAATTACTAAAACCACTCAACTCTTAGAATTTAGAATATAGAATGAGAATATAGAATACGGAACATTGATGGATTTAGGGATAATGAATTAGCCCTACATTATCTTTGAAACAAATTGAAAGAATCTCTTAGGTTTGTTGTTCCACCAAAAAATGGATTAGTCAGAGGACTTCTACAAATACTTAAGATCAATAAAAGAATAGGTCCTAGATCCATGCGACTTAGGATTGGGTTGGGTCAGGTTGAAGTCTTGAAACAGAATTATTTCATTTCATGATTTGAATTTTATAAATTGACTGGGATTGGGTATACCAAAACAAAAAAAAAGTGTTAACTTTTTGATCATGGACAGGAAAAGAGGAAAAATATCATATGTAATTCATTCATGAAAGTAGATGAAGAGAGATGGGTATTTGATCCGAGATTTTACAAATACCAATTGGGTCCGTTGGAATGAATTATTGTGTTTATTTTATTGTTCCTACTACTATTCAAATTTCTATACAAAACAAAAATGGAATGTATTAGGGCTATACGGACTCGAACCGTAGACCTTCTCGGTAAAACAGAGAAAACTGATTATTATCGAAATGATTCGAACTGTTTCAAAGACCCAACATGCATTTTGTTGCATTGGGCTCTTTCATCAACTGATGTAAAGATCAGTTAGTCCACCATATTTTTTCTTTAGAGGAAGATAAGAAGATAATGAGATGGCTCCATGTGCTCTGATTCATTATTTGTATCCTTATCTAGGAGCAATACCAAAGTGTTTCAAAGAAGGGTGACCTTTATTTAGGTCTGCCTTCGGCCTAGATAAACCTAAGTGAAATGCAGTCTCTATCGCTCCGCTGCAATAGTAAAATATGAGACTTCATACACCTCAAAGCTCATAGGACGAAAAGAGGTTCTTTTGAGATCCTTATACTCATTATGCCTGGCATTGAATGGACTGGGCTTTTACCTTACAAAGGCAGGTTCTTTTTTATTTGGCACCGGAATTCGCACCTGAACCGGATCAAACCAAATTTGTCAGGCTATTTTTCTATTATTCTCTCGAATCTATGGAGTAAGACATCGACTTTTCAAAAAGATCAATTATGATCATTGCATAATGGACTCCCTTGAAAAACATTGGCGCACGTGTAAACGAGGTGCTCTACCTAACTGAGCTATAGCCCTTGTCATAACCATTTTAACATAGAGACAATTTCTTGTCAAGAAGGGTATCCCATAATCCCACATGATAACTCTCTGATCCGTTTATGTTTACTGGTAAAAGATTGATATTGCTTAGAAAACATATTTTATCTATAATCCATCGAAGTGATGGAGACCCTTTTTGTGGTGATAAATAACCTACTTAACCCAGTGGTTAGAGTATTGCTTTCATACGGCGGGAGTCATTGGTTCAAATCCAATAGTAGGTAGAACTTATTAGATACCGGATTCTATGGTATCTAATAAGTTTTTCTACCCATCCTCTTTTTTTCGTTCTATCATCAGATTAATCAAATTAGACTTCATTGTGTTCAATTTGTGGAATCAAGATGTAGTGTGTAGTGTATAATAAAGAACTCTTTTGATTTTGATTGAATGTATTGACTACTAATAGGAAATCACTTTGACAGCTTCTACTCGTGTCCTAGCTCGTCTGAGAGCTAGATTTGCTTCAATTGCTTGTCTCTTACCCTCAGCTCTACTCAAGTTAGCTTCAGCTATTTCAAGAGTTTGTTGAGCTTCTTGTGGATCAATGTCAGTACTAATTTCCGCACCATTTCCTAAAATGGTGATCTCATTATTACTTATTCTAGCGAAACCGCCCATAAGAGCCACCGTGAACCATCGGTCGTTTAGCCGTATTCTCAAAAGACCTATATCTACAGCCGTGGCAATGGGGGCATGGTTTGGTAATACTCCTATTTGTCCACTATTAGTAGATAAAATAATCTCTTTCACTTCGGAATCCCAGATCATTCGATTAGGAGTCAGTACACAAAGATTTAAGGTCATTTCTTCAATTTGCTCTCCTCTTCTAAGTTCATAGCTTTCGCGGTAGCTTCATCGATGTTACCCACCAAATAAAAGGCCTGCTCGGGAAGACCGTCTAATTCTCCGGAAAGGATGAATTGAAAACCCCGAATTGTTTCTGCGAGACCAACATATTTCCCTGGAGAACCAGTAAAGACTTCTGCCACAAAGAAAGGTTGTGATAAGAAACGCTCAATCTTGCGTGCTCTTGCTACAGTTAAACGATCTTCTTCGGATAATTCGTCCAACCCAAGGATAGCTATAATGTCCTGAAGTTCTTTGTAACGTTGTGAAGTTTGCTTAACCCTTTGCGCAGTTTCATAATGTTCCTCGCCAACGATCCGAGGTTGTAACATAGTTGACGTTGAATCTAAGGGATCTACTGCTGGATAAATACCTTTGGCAGCTAATCCTCTTGATAATACGGTAGTAGCATCTAAATGTGCAAATGTCGTGGCAGGAGCAGGGTCGGTCAAATCATCCGCAGGTACATAAACTGCTTGGATCGATGTTATGGATCCTTCCTTGGTAGAAGTAATTCTTTCTTGCAAAGAACCCATTTCCGTACTAAGGGTAGGTTGATAACCCACTGCAGAAGGCATTCTACCTAATAAGGCAGAGACTTCGGACCCTGCTTGAACGAAACGAAATATATTGTCGATGAATAGAAGTACGTCTTGCTCATTAACATCCCGGAAATATTCTGCCATGGTTAGGGCAGTCAAGCCAACTCTCATACGAGCTCCTGGCGGTTCATTCATTTGACCATAGACTAGAGCCACTTTGGATTCTGCAATATTTTTTTCATTAATCACCCCGGATTCTTTCATTTCCATGTAGAGATCATTTCCTTCACGAGTACGTTCACCTACTCCGCCAAATACGGATACGCCTCCGTGAGCTTTGGCAATGTTGTTGATCAATTCCATGATGAGTACTGTTTTACCCACTCCAGCTCCCCCAAATAGTCCGATTTTTCCCCCACGGCGATAGGGGGCTAAAAGATCCACCACTTTAATCCCTGTTTCAAAGATTGATAATTTCGTATCTAACTGTATGAAGGCGGGTGCAGATCTATGAATAGGAGATGTTGTGCGAGTATCGACAGGACCTAAATTATCAACGGGCTCTCCAAGAACGTTGAAAATTCGTCCGAGAGTAGCTCCCCCGACTGGAACACTTAGAGGAGCTCCCGTGTCAATCACTTTAATTCCTCTCATCAGACCATCTGTAGCACTCATAGCTACAGCTCTCACTCGATTATTTCCTAATAATTGTTGTACTTCACAAGTTACATTAATTTGCTGACCGACAGTATCTCGACCCTTAATTACCAAAGCATTATAAATATTAGGCATCTTGCCTGGTGGAAAAATAACATCCAGTACTGGGCCAATAATTTGAGCGATACGCCCTAGGTTTTGTTCTTCAAGTGTAGAAACCACAGGATCAGAAGTAGTGGGATTGCTTCTCATAATCATAAATCATAATAAATATGTCGAAATTCTTTTTTGAAAAGTACTGAATCAAAAATAAATATCCGATAGCAAGTTGATCGGTTAATTCCATAAGAAATAAATGGGAGTTAGCATTCAATTGAGTTGGTACCATCCAATTGAATCCAATTCAATCCTTTACTCAGTGAATGAGTCAATTTTCAATTCTTTCTATTGTACTATTGTATTTTTTTCTTTTTTTTTTTTATTTTGATTTGTGTTGTGCACCTATTCTTCTTTATATACCATATCTGTTCCCTTTTCTAGATGAATTATGCCTCTTTTAACATCTAGGATTTACATATACAACATATATTACTGTCAAGAGGGGGGGCGGGGTCCTCTATTCTTTCTTTTTCTTTCTATATTCTATATTAGTATTAGATAGAATATTAGATATTTCTATTTACTATTTATATAGATATATATATATATCTTTACTATACTTTTATACTTTATCTTTCTATCTTTAATATCTTTATCTTTACTTTAGAATTTATTAATTCTATAATTTCTTAATTCTAATTTAGAATTATTTAGAATTCTATTTCTATTCAATTTCATATTTATCTATTTTCATTTTATTTGATGTTTTTTTTATCTTTATTTTGCTATTTTTATTTATTTTTCTTTTTTTATATTTATTATAAAAATTAGTATGAAGAATAATGAATATAAAAAAAATTAAGAAGGTGATCAATTCCATTAGAAATAGAAATCTTCAAAACGAAGATTGGGTTGCGCCATATATATCAAAGAGTATAAAATAATGATGTATTTGGTGAATCAAATACATGGTCCAATAACGAAACCTTTTCAAATTTTCATTATTCATTAGTTGATAATATTAGTTTAGTTGAATCTTTTTTTAATTGTAAATATTTTTGTCAAAGGTTTCATTCACGCCTAATTCATATCGAGTAGACCTTGTTGTTGTGAGAATTCTTAATTCATGAGTTGTAGGGAGGGACTTATGTCACCACAAACAGAAACTAAAGCAAGCGTTGGATTTAAAGCTGGTGTTAAAGATTACAAATTGACTTATTATACTCCTGACTACGAAACCAAAGATACTGATATCTTGGCAGCATTCCGAGTAACTCCTCAACCGGGAGTTCCGCCTGAAGAAGCGGGGGCTGCGGTAGCTGCCGAATCTTCTACTGGTACATGGACAACTGTGTGGACTGATGGACTTACCAGTCTTGATCGTTACAAAGGACGATGCTACCACATCGAGGTCGTTGTTGGGGAGGAAAATCAATATATTGCTTATGTAGCTTATCCTTTAGACCTTTTTGAAGAAGGTTCTGTTACTAACATGTTTACTTCCATTGTGGGTAATGTATTTGGTTTCAAAGCCCTACGAGCTCTACGTCTGGAAGATCTGCGAATTCCCACTTCTTATTCCAAAACTTTCCAAGGTCCGCCTCATGGCATCCAAGTTGAAAGAGATAAATTGAACAAGTACGGTCGTCCCCTATTGGGATGTACTATTAAACCAAAATTGGGATTATCCGCAAAAAACTACGGTAGAGCGGTTTATGAATGTCTACGGGGTGGACTTGATTTTACTAAGGATGATGAAAACGTAAATTCACAACCATTTATGCGTTGGAGAGATCGTTTCTTATTTTGTGCCGAAGCGCTTTATAAAGCGCAAGCCGAAACCGGTGAAATTAAAGGACATTACTTGAATGCAACTGCGGGTACATGTGAAGAAATGATCAAAAGAGCGGTATTTGCCAGAGAATTGGGAGTTCCTATCGTAATGCATGACTACTTAACTGGGGGGTTCACTGCAAATACTAGCTTGGCTCATTATTGCCGCGACAATGGTCTACTTCTTCACATCCATCGCGCAATGCATGCAGTTATTGATAGACAGAAAAATCATGGTATGCATTTTCGTGTACTAGCTAAAGCATTACGTATGTCTGGTGGAGATCATATTCACGCTGGTACAGTAGTGGGTAAACTGGAGGGGGAACGTGAGATGACTTTGGGTTTTGTTGATTTGTTACGTGATGATTTTATTGAAAAAGATCGAAGTCGTGGTATTTTTTTCACTCAAGACTGGGTCTCTATGCCAGGTGTTCTGCCCGTGGCTTCAGGGGGTATTCATGTTTGGCATATGCCTGCCCTAACCGAAATCTTTGGGGATGATTCCGTACTACAGTTCGGTGGAGGAACTTTAGGGCACCCTTGGGGAAATGCACCCGGTGCAGTAGCTAATCGGGTGGCTTTAGAAGCATGTGTACAAGCTCGTAATGAGGGACGCGATCTTGCTCGTGAAGGTAATGATATTATTCGTGAAGCTAGCAAATGGAGCCCTGAGCTAGCCGCTGCTTGTGAAATATGGAAAGAGATCACATTCGATTTCGACCCAGTGGATAAGCTAGATAAAGAGACAAAATAAGCGCGTATAATTGAGCAATTCCTGTTTGTTCTCCTAATTGATTGCAATGAAACTTGGCCCAATCTTTTCCTCAAAAAAAGAAAGATTGGGCCGAATCGGATAAAGAATAAACATCTTATACTAATCCTATACTATGAACTATGAGGGTCTTTGTTGCATATATCTTTTTTTATATGTACAGACCTTACGATATACAAGTATATACAAAATATAAACATAAGAAGATAAGATCGAAGGAAGACTAAAAAACTTATCTATTCTATTATTTCTTGTTGGAGCCATAGGCTGAATTATGGATCCTTGGGATTGGATTGGTGGATCATTTTATATTCCTTAGTTTCAGGCCATAGATCAAGCCAAGGGAAGGATTCCTTCTACCCCTATCCTGTATATTGTCTTTTTCGTTCCCTATTGTAATATAAACTCATTTTCTTATTTGACTATATGACACGAGATTCTACGAGACGAGAATTCATTTTTAATTTATGGGAAGAAACAACTATGTATATTTTTGATGAGAATTTAAAGTTTTACATGAGAAAAACCTGTCTTTATATATCATATATCTTTTTTTGAGGAAAAGATTCTATCATAATCTCTATCATAATATTGAAATGATTCACCGGATTCCTCAAAAAGAGAATCTTTTATTTTCAAGACTCGCTCGTTTTTCATTAACAATCTTAATGATTGGATCATATACTTCATTTGAATTCTGATGAGAAATAAAAAGAAAAAAAAATAGTAAAATGATTTTTTCTTCATCGAATGACTATTCATCTATTAGTATTAGGTTTTTATCAAATAGGGGGCAGAAAGAATCTATGGAAAAATGTTGGTTCAATTCGATGTTGTCTAACAAGAAGTTAGAACATAGGTGTGGACTAAGTAAATCAATGGATGATAGTCTTGATGCTCTTGGACATACCAGTGGAAGTGAAGAAACTATTCTAAATGATGCGGAGAAAAAGATTCCTAGTTGGGACAGTTATAGTTTCAGTAATATTAATTATCTAAATTATTTATTTGATAGCAGGAATATTTGGAGTTTGATCTCTGATCATACTTTTTTAGTTAGAAATAGTAATGGTGACACTTATTCTGTATATTTTGATATTGAAAATCAGATTTTTGATATTGACAATGCTAGTTTGAGTGAACTAGAGATTCTTTTTCCTAGTTATTTGAATAGTGGGTCTAATAGTAGTAATTACTACTATTATTATTCCATGTATGATACTCAATCTAATTGGAATAATCACATTAATAGTTGCATTGATAGTTATCTTCGTTTTGAAATCAATAGTGACATTTACAGTGGTATTGACAGTTACATTTTTAGTTTCATTTGTACGGAAAGTATAAAAAGTATAAGTAGTATTGAAAGTGGAAATTCTAGTATCAAAACTAGTAGCAGTTATTTCAATAGAATAGAGAGATCTAATGATTTCGATATAAATACAAAATACAAACAGTTATGGGTTCAATGTGAGAATTGTTATGGATTAAATTATAAAAAATTTTTTAGTTCAAAAATGAATATTTGTGAATACTGCGGATATCATTTGAAAATGAGTAGTTCAGATAGAATCGAACTTTTTATTGATCCTGGCACTTGGGAGCCTATGGATGAAGATATGGTTTCTATGGACCCCATTGAATTTCATTCAGAGGAGGAACCTTATATAGATCGCATCTCTTTTTATCAAATAAAAACGGGTTTAACTGAAGCTGTTCAAACGGGCGTAGGTCAACTAAATAGTATTCCCATAGCAATTGGAGTTATGGATTTTCAGTTTATGGGAGGTAGTATGGGATCCGTAGTAGGTGAGAAAATCACCCGTTTGATCGAGTATGCTACTAATAGATCTCTACCTGTCATTATTGTGTGTGCTTCTGGAGGAGCACGCATGCAAGAAGGGAGTTTGAGCTTGATGCAAATGGCTAAAATATCTTCTGCTTCATATGATTATCAATCAAATAAAAAGTTATTCTATGTATCAATCCTTACATCTCCTACAACTGGCGGAGTTACAGCAAGTTTTGGTATGTTGGGAGATATCATTATTGCTGAACCTAATGCCTACATTGCGTTTGCGGGTAAAAGAGTAATTGAACAAACATTGAAAAAGACAGTACCCGACGGTTCACAAGTGGCTGAGTATTTATTCCATAAGGGCTTATTCGACCCAATCGTACCACGTAATCCTTTAAAAGGTGTTCTGAATGAGTTATTTCAGTTACATGGTTTCCTTCCCTTGAATCAAGATTAAAAAAAAAAGATTGAAATTCTTCATTTTCAAATGGAAGTATAGCACTAGCTTCAGTTATTTTTCTTTGTAGCGAATACGCATTTAGTTCATTATAATGAAAAAAAGAAAACTAAGAAGATGGTGTTTTCTTTGGAGACATCTGTTATAAGTGTAGTAAGAGTTAGAAGTTTTGGATAATGACTTTTTGCCCCGGATCCATTTTTTATTCTACCTCTCTTCCTGATTAGGAATAAGCATCCCTCTATCGACAAGATAAAAAAGAATTTATTTCTCCTTCCGAGAAATCCGGGAAATAAAAATAAATTTCTCCGTCCTTTTGACATATTCATATATAGAACAACGAAAACTAGATAAAAAAAGATATCGAAAAAATGAAAAGAAAAGATTAAATAAAAAGAAATAAGAAATCTCAAATCAAAGAAAGAAAATAGAAATATTCAAATAACCAAATAACAAATAATAAGAATATAGAAGTTATTTCTATTTAGCGAAAACCCCCGTTTTTCACTAGGAATCCCTGTTTGTTGGATAAGATTGGTTAAAGTCGGGAACTCATAAGAAACTCTTTTCTATCTTTCCTTTAAAAACAAAAAAGGTGTTTTTAAAGGAAAGATAGAAAGACGATGAAGATAAGGATGGGATAATAAGAATACAATTTCTTAAAGCGGATTCTCATAAATATTCGTGTAGAAAGAGGAATAGGTACACTTCATTGAGTTCTACATTCGTTATTGATTATGAAATACTTCATATTAATATTATCTTAATATTTTATCTAATAGATAGACTTATCATAAGATATCTTTATAATTATAATAGGTACAAATATGAAATTGAGGTACCCATTCTATGATAGATTTTAACCTTCCCTCTATTTTTGTTCCTGTAGTGGCCCTAGTCTTTCCGGCAATCGCAATGGCTTCTTTATCTCTTTATGTCCAAAGAAATAAGATTGTCTAAATATGATGGGACCAAATCTCATCAATTTATTTCAAAACTGGATCATCATACAGATACTTTTTTAATGTAAATGTAATATGGTAGGATATATGATATGTGGCTTTTCCGAAAACAAAAGGAAGAGTTGTTTTGTTATGTATGCCGATGCATAATATACACATTAATGCATGTATATGCGGTTATAGCTTAATCAATTAAATGATTAAATTAAAAATGATCAGCAAATCTTTTTTGAAAAATATTTGAAATAGAAACTCAATTTATCTAACCAATTATTCCTAATAGTTCCTGTTGGAATGCTGCTAGTTGATGAAAGTTACTTCGGGATCAAGCAATAAAAGTTGAGTCAAATCCTTTTGGATTATTCTCTCAATTCCAATCGAATGCAACTGGATCTAGTATAGTATGAACTGGCGATCAGAACATATATGGATAGAACTTATAACGGGGTCTCGAAAAACAAGTAATTTTTGCTGGGCCTGTATCCTTTTTTTAGGTTCACTAGGATTCTTAGTGGTTGGAACTTCCAGTTATCTTGGTAAAAATCTGATATCCGTATTTCCGTATCAGCAAATTATTTTTTTCCCACAAGGGATCGTGATGTCTTTTTATGGGATCGCGGGTCTATTCATTAGTTCTTATTTGTGGTGCACAATTTCATGGAATGTAGGTAGTGGTTATGACAAATTCGATAGAAAAGAAGGGATAATGTCCCTTTTTCGTTGGGGATTTCCTGGAAGAAATCGTCGCGTCTTCCTTCGTTTCTTTCTGAAAGATATCCAATCAATCAGAATGGAGGTGAGAGAGGGTCTTTTTCCTCGTCGTGTCCTTTATATGGAAGTCAGGGGCCAAGGAGCTATTCCCTTGACCCGTACTGATGAGAATTTGAATCCACGAGAAATTGAACAAAAAGCTGCCGAATTGGCCTATTTCTTGCGCGTACCCATTGAAGTATTTTGAAATGAACTGAAGAATAAATCTCAGTATGAGAGAAGGAACTTAATACATCTCAAAAACAGGAAGACGTATATGGAACAATATTAATAAAATCTAATATAACTAATCAAATAAAATATATTAAAAAAATCTATTTGTACACAAAAACTATTTTTTATACACATACACATGGCGTCCAGCTTCACTCTTTATACTAGTAAAAGGTCTAATAATTATAGATTCATCAAATATCCTAACATGTCTTTTGTTTTCGTAAAACATAATTCCTCTTTTTAGGCCTTTGAATTGATACCCTATCCCCGCGCTGCGGTTAGACAGTGAAATCTTTCGAATTCAAAATAGAAATACAAGAATTAAAGGATCCGGTAGGGTTCGTCTTTAAATCAAAATTCTATTCGTTAGTTCAAATGGGTTTTCGAGTCTTCATCGAAAGGAATAAATGAAGGGGAAATTGGTTACAATTTGCCTAATTGTGATCTCTGGAATATGGAAAGAATATTTACTTCTTTTTTTTTCATTTGAAAAGGGCCCTTCTTCTATGTTCTATTCTAGATCCAAAGACTCAATTTTTACACAAAAACAAAAATAGGAAAAGATCCATAGGTTCGATACCTTATTCTTGTTTTCTTGTTAGAGTTATAGTAGAGTTATAGGCTCTTGTTATATAATTTGTGCTTCATAGAAATCTCAGATAGAATCGACGAATGAAACAGGTTCATTAACAATTAACATCATGGATACAGAATGAAAAAAAAGAAAGCATTGGCTTCCCTCCCATATCTTGTGTCTATACTCTTTTTGCCCTGGTGGATTTCTCTCTCATTTAAGAAATGTCTAGAAACTTGGGTTATTAATTGGTGGAATACCAGGCAATCTGAAATCCTTTTGAATGATATTCAAGAGAAAAATGTTCTAGAAAAATTTCTGGAATTAGAAGAACTATTCCTGTTGGACGAGATGATAAAGGAGTACTCGGAGACACATATGCAAAGGCTTCATATAGGAATGCACAAGGAAACAATACAATTGGTCCAAAGACACAATGAATCTCATTTCCATATCATTTTGCATTTCTCTACAAATCTAATCTGTTTCGCTATTCTAAGTGGTTATTTTTTTCTGGGTAATGAAGAACTTTTCATTCTAAATTCTTGGATTCAGGAATTTCTCTATAACTTAAGTGATACAATCAAAGCTTTTTCGATTCTTTTAGTTACTGATTTATGGATCGGATTTCACTCGACCCATGGTTGGGAACTAATGATTGGTTCGATCTACAGCGATTTTGGATTGGCTCAGAATGATCAGATTATATCTGGTCTTGTTTCCACTTTTCCAGTGATTCTAGATACAATTGTTAAATATTGGATCTTCCATTTTTTAAATCGTGTATCTCCTTCGCTTGTAGTAATTTATCATTCAATGAATGAATGAATAATTCATTATATCTTTTTCTTTATACTTCTACCTTATTCACTTCAAGGTATTCATACTATAGTACAATTCTTTCAGTACAATCAATACAATGGCAAACTTGTGGATAGGGAATTCTACTAGATACCTATCAAATTTATTGTAGAAATTCCGGGGATCAATGATTGGACCATGCAAAATAGAAAGACTTTTTCTTGGGTAAAAGAACAGATGACTCGATCCATTTATGTATCGATCATGATATATGTAATAACTCGAGCATCTATTTCAAATGCATATCCCATTTTTGCACAGCAGGGTTATGAAAATCCACGAGAAGCAACTGGGCGAATTGTATGTGCCAATTGCCATTTAGCTAATAAGCCCGTGGATATTGAAGTTCCGCAAGCTGTACTTCCTGATACTGTATTTGAAGCAGTTGTTCGAATTCCTTATGATATGCAACTTAAACAAGTTCTTGCTAATGGTAAAAAAGGAGCTTTGAATGTAGGAGCTGTTCTTATTTTACCCGAGGGATTCGAATTAGCCCCCCCCGATCGTATTTCTCCCGAAATGAAAGAAAAGATGGGCAATCTTTCTTTTCAGTGTTATCGTCCTAATAAAAGAAATATTCTTGTGATAGGTCCTGTTCCCGGTCAGAAATATAGTGAAATCGTCTTTCCTATTCTTTCCCCCGACCCTGCGACGAAAAAAGACGTTCACTTCTTAAAATATCCCATATATGTAGGTGGGAACAGAGGAAGGGGTCAGATTTATCCTGATGGTAGCAAGAGTAACAATACAGTTTATAATGCTACATCTGCTGGTATAGTAAGCAGAATAGCACGTAAAGAAAAGGGGGGATATGAAATATCCATAGTTGATGCATCAGAAGGACGTCAAGTGGTTGATATTATACCTCCAGGACCAGAACTTCTTGTTTCAGAAGGTGAATCCATCAAGCTTGATCAACCATTAACAAGTAATCCAAATGTAGGAGGGTTTGGTCAGGGAGACGCGGAAATAGTGCTTCAAGATCCATTACGAGTCCAAGGCCTTCTGTTCTTCTTGGCATCTGTGATTTTGGCACAAATCTTTTTGGTTCTGAAAAAGAAACAGTTTGAAAAGGTTCAGTTGTACGAAATGAATTTCTAGATCTAGAGATTCCTTAAAATAAAGTTGGTAAAAGTGCCAAATTCTTGTTGATCAATAGAATTATATATGATTATATGATTCAAAAAATTCTATAAGTCTTTTCTTTGTTTTTTTTTTACTCTTTTTTATTTTGCGGGATGTCTGAAACTCATTACTTGTATACCATTTCTAATGATAGAAAATAAGTATACAAATAGAAAGGAATAGAATACAAGGCAAGGAGGACGGGAAAAATGAAATTTATAGAAAGTATTCTTAGTCTTCCTAATCGTCTATTCGATTCGATACAAGACGACACAAGAAAAGGATTTTCTTGTGTCGTCTTGTATCGAAAGAATCATGTCTTCTTTTTGCCAAAGATTTTTTATTTTCCGGATAGAATTGAACAAAAAGAACTCCTTCAATTCACTTCAACTTATAACTTATAAAAATAATTCAAACAAAAAAAGACTTATCTTGTTTTGTTTCGGACGAAAAGCGGATTAGATCTTTACGCATATCCAATTCTTTCTTTATTATCTCATTACAGTTTTCTTTTTTTCTATTTCTATTATATTTTATTTCTATTATATTATTATTATATATATATATATATTTTTTCTTTATAAATAGAAATATATAGAAAATATATAGAATCTAAATATATAAATAGAGTTTTTTTCTTTTTATTATTTGTTTTAGTTTAGTAGAAATAGTTGAGTATAAAAAGAAAAGGATTTGCAGGATGTTTCATACGGATAAATCTATACGTATTGGATAATCGATGGATTCGATTCCTCCTTTCTTGTTGCTTCATATTCAAAATATTGACATAATAGTGAATATTATATAGGAACGACAGAAACTATGAATCAGTCAATAGATTAAATTATTCAAAAACATCATAATAAAAAAGGAATAGAATAGAGTGGTTTGAAACATCATAGCATAAGGATCCATTTTGTCATTTCTAAAATAGAATATTTGGATTATGTTAACTGAGGTTCCATATGTTTTTGAATAGATCAAAGTCTCGCTCTAAGAGTAAGAACTCAGCGGGGTAAGGCCCCGATGAGTTCTTACTCTTTCATGTCTACAATCTAATCTGGTTCATATGATAGTATTACAGAGATGAACCCAACCCGGAATAGGAGCCGTAAAAGAAAATGCCTATTAAACCGATCACAGGAATACCAGTTACAGTACCTATCAGCCAAAGAGGAATCCTTCCAGTAGTATCGGTCATTTCCCCCCCTTCTTTTTCATCAAGTGGTCATGCTATAGACAAAAACAGTCATGGATAATTATGAGGATAGTATCCTTCCGAATGGGATAAGAGAATTCCTACTATTACTATTTCTTTTTCTTAATTGAAGAAATAATTGGAAAATAAAACAGCAAGTACAAAAATGAGTAATAAACCCCAGTATAGACTGGTACGATTCAATTCAACATTTTGTTCATTCGGGTTTGATTGTGTCATAGCTCTATAATTCGAATTATATTTATCGTTGGATGAACTGCATTGCTGATATTGACCCTAAAAAAGAAACAGTAGGTACAGCCAGTCCGTGAACAGCCAACCATCGCACTGTAAAAATTGGATAGGTTCGATCTATGGTCATTGGGAACCTCCTAAAAGGATTTGCTAAATTCATCGAGTTGTTCCAAAGAATCAAAACGGCCAGTTATTAATGGAATCCCTTGTCGGCTCTCTGTGAAATATTCATTTGGCCGAGGACTTCCAAAAACATCGTAAGCTAAACCCGTACTGACGAATAACCAACCCGCAATGAATAGGGAAGGTATAGTAATGCTATGAATGACCCAGTATCGAATACTAGTAATAATATCAGCAAAAGAACGTTCTCCCGTGCTTCCAGACATGCTGAGCTCCACAAATTCTTATATGTTCAAAAAAAGGGCGGGCCGATTCCGTGAAAGATGGAATCAGTAATTTTAAAACTACTGATACTGGATCTTTGTGAGATCGTCAATTTTGTACCAAAGGTGTATTTAGAGTAGACCGAATCAGTATAGCTATCCTCCCTCTAGCGCAGCAACGCAGCCTCGATCATTACCGAAAGGAAATGATATTACTATATCTTCCTTGTCTATGTATAAATGTATTTTCCTTAGAATATTAGAATATAAGATTAAGTAAGGTTTATATTAGTAGATTCATCATAATAATAGAAAGTAAAGATCTTGAATGGATGGGCTCTAATAATTAATGAGAGATATCATGATTGAAAGATCTCATTATATTCATAAAATTCCATTATATGTTATTTTCAATCTATAAAACCTTTTGGTAGTTCGGTTCATATTTTATTTTTTTGAATCCTCTCGTTTTATTCAAGTAATTGGTAATTGTTCGTTCATAGAATAAATATGCTAATACTATTTCACTTTGAACTTCTAAACTTAAGCTATTATTACTATTCTAAATAATAATTATTCTAAATGGAAATTCTTATTACTATCCCTATCTATTTAATTCTTTACTATTTAATTTTTCATTGGTTAAATGGAATTAATATATTAGAATTAGATTTCCTATTTTTTATTATTCTTTTTTATATTTAGTATTTTGAATATTTGCGAAAAGAAAAAAAAGAGATCGTTGGAACAATCCATATTCATGATGCAACTGTTGTTACATTAGATCTCCCCAAGAGTTCTTTCCTTATGGAACTACAATACAAAACAAAGATGGGATTCTTTAATATGGAAAGAATATAGAACCTAAAAGAGTAATAGAAGTTGCTATTCTTTGAATTCTTTGAATCGAGAATGAAAATATTCAATAATTTTCAATTTTTTGAAAAAGTCAAGATTTTCTTTTTTTTAGCGTCCGTCTATAATGACTGATGAATCAAGAACTTTCAATTGGAACTAAACGAATTCTTTAAATTAGTTTTTATTACCGTCATATCTGGATTGAGACTTAGGTAAATGTTTTATTCATATATGTATTAAAAGAACATATCTAATTTAGCTCTTTCATGCCTATTCTAACTAGTTATTTTGGTTTTTTACTGGCTGCTTCAACTATAACCCCAGCTCTATTTATTGGTTTGAACAAGATACGACTTATTTGAAATGAATGAAATTCAATAAACAATTTACAAAAATAAAAATCAAAGCCTCCCAGAATATTTCATTTCAGGTATTCTAGGGTTCTCAATTGTAAATTCCCGGTCATTGAGATTCACGGATAATTCAGATTAATATTAAGGGATAGATCTTACCTCTCTTTTTATTCCCTAAAACAAATCGAAATGATTGAAGTTTTTCTATTTGGAATCGTCTTAGGTCTAATTCCTATTACTTTAACAGGATTATTTGTAACTGCATATTTACAATACAGGCGCGGTGATCAGTTGGACCTTTGATTGAGTAACATTTCTTTTTTTGATTGACCTCCTCCTTGTAGGAGGTCAAATTTAAGTTGCAATTAGACTTTGTTTTTTGTTAAGTTATTTCATTGTAATTCGACATAACATAAACGGAATCACGCTCTGTAGGATTTGAACCTACGACATCGGGTTTTGGAGACCCGCGTTCTACCGAACTGAACTAAGAGCGCTTTCTTATATCCTATAACAGTAGATACGATTGTAAAGTGTAAAGAAAAGAATTTTTTTACCCCCGAGGGGTCTTGTGTACATGTAAATATAGTATGTACAAACGAAAGATTATGTCCAAAATTTCCCGATCTTACTCAATGAATCCCTCGTAACTGTCCATAGGAGAAAGAATAGGTAGGGATGACAGGATTTGAACCTGTGACATTTTGTACCCAAAACAAACGCGCTACCAAGCTGCGCTACATCCCTTTTAAAAATTGTTGTACAGTGTCATTGTATAAAATATATGTCTTGTTTTCCACATCCTTCTTTTTTGCTCTACCTATATATATAGGTAGAGAATGTTCTTGTCATTTTTTTAGGGAGCGGCAAAATTGAATCTGCTGGGTCATTGTACATATGCATTTTAGTTAGTAATTCCTAATTCTAATTTTATTTCAAGCAAAAACAAATGATCTTGAACTAAAATATCGGGTTATCTATGATCTATGTATTAGAATATCGAACTAGGACTATATATGTATTACAATATACAATACAAATAAAGAATTAAAATAAATAAGAAAAAAATAGAAAATAAAAGAAGAAGGAGGATTTTCAATGCGAGATATAAAAACATATCTCTCAACGGCACCTGTGCTAACCACTCTATGGTTTGGGTCTTTAGCAGGTCTATTGATAGAAATTAATCGTTTATTTCCGGATGCCCTGTCATTCCCCTTTTTTTCATCCTGATTGAATTCTTGTATTGATCTGTGAAGAAATGAAGAAGATTCGAGATACAATTCTACGTAACATGGCTCCAATTTTGCTTCCTTTTTCTTTCCTATCCTAAAAAAAAAAAGAAAGAGAAAGAGTGTATCGAACCTCAGTCAAAATACAGTGAATCTACGATAGAATTTGGGGGAAAAGAAATGGAAATGTGGATCCAGTCTAGGAGCGACGAAATTTTAACATAGAAAGAATAAAATACTGAGATTAGGATAGGAATAATTCATAGTTAGAAAAAATTGTATTAATTAATTATTACAATATTCTTAATATTCTTCTATTAATGAAAATGAATATGACTCTTTTTCTTTATAGTTATAGTAATTAATATAATAGTAATTCTATTTTAGATTATATTACTCCGAAGTCATTCGAATTCTAAAAAAGAAAATATTTACAAATTCCATTTCTAGTTAGTAACTTTTATTAATATAGTCTAGATATAGAATATAGATTCTTTTTTTTTTTATTTTTCTTTGGTTCGGATCAAAAAGAAAATGAAGAGAGTTCTAAAGTGAAGTCGATCCAAAATGAAAAGGAGGTTCATGGCCAAGGGTAAAGATATCAGAATTATAGTGATTTTGGAATGTACCTGTTGTGTTCGAAAGGGTGTCAATAAAGAATCGCCGGGCATTTCTAGATATATTACTCAAAAGAATCGACACAATACACCCAATCGACTAGAATTTAGAAAATTTTGTCGCTATTGTCAAAAGTATACGATTCATGGGGAAATAAAGAAATAGGTGGAACGGAATGTGTGTGTGATTTTTCCAAGTAGCGGGAAGAGTAAGAACTTTACATCTTAACATATATAATACAAACCAAATCCTATTTTGGTCGAATCTTAAATGAATAAGAAAAAAAAAGAGAATTATATTTTATAGATTCTTTTATATAGAAGGAATAAACAAACCATGGATAAATCCAAACAACCTTTTCGTAAATCCAAGCGATCTTTTCGTAGGCGTTTACCCCCAATTGGATCGGGGGATCGAATCGATTATAGAAACATGAGTTTAATTAGTCGATTTCTTAGTGAACAAGGAAAAATCTTATCTAGACGGACGAATAGATTGACCTTGAAACAACAACGATTAATTACTATTGCTATAAAACAAGCTCGTATTTTATCTTTGTTACCTTTTCGTAATAATGAGAAACAATTGGAGAGAGTGGAGTCGATCCCTAGAACTACTGGTCCTAGAACCAAAAATAAATAGATATACTCCTCAAAACTCCAATCGGAACTCAAGCTTATATTAAGGTTTTGCTCGAAAAAACTAGAATCCAGATTTGATTCTTGTATTTTGTATTATAAAAAAGGAAAAATGGGGAAGAAATCTTTTTTTCTTGAAAGATGTTCGTTTATTCCTACTACTCAAATCTTAATTTCTTTTTCTTCTATCTTCCCGGAGTCCTTTCTCCGGGAAATCTTGTTTCAATAATTCTTGTTTCATGTATAATAGATTCTATTAAGATTCTTTTATTCCTTTATTTTATTTGTATTTTTTTTTTATTTTTGATTGATGATTTTATTTGAAATAATATCAAAACAATTCTTATTTGATAGGGCTATTTGCGCAAGTATTTTACGATTCAGAAGCAATTGTCTCTTGTACAGATTGTTGATGAATAGGCTATAACTATAGAATAGCCTATCCTCACGAGTTACCGCATTTATCCGAGTGATCCACAAACGACGAAAATCTCTCTTTTTCCTGCTCCTATCTCGATGAGAGGAAACCAAAGCTCTCATTCTTTGTTGAGTAGTCGTTCGAGTAAGTCTTGAATGAGCCCCTATAAAGGTTGATGCAAATAAACGAATCTTTTTTCGACGTCTCCGAGCTGTATATCCTCGTTTAACTCTGGTCATTGAATCAAATGAAACTTTCTTGAATAACTAATTGATTTCTCTTCTTTCAGTCATCCTTTTCTTCCGGTCAATTAATAACAAAACGGATTCTTCCGATATATAAAATATAAATTCCAATGGCTTTTGCGACTATGACCTTCCCGACCACGATTTTTTCTTTCTTCAAGGTATCTCGCCTGGAAATAAGAAATTCGACTGCTACTAAATAAAAAATAATAGTGGGTTTCCTCGTTTCTATGGCAACTTCTGAAACGGTGAGGTCCTCTCTATACACCGGAGCCTCTTCTTTCATTTCATTGAATTTTATTGTGAACTTGTATAGTTCACACTCTTTGGCTCTACCCATCCATTTTTCAATTAGAATTCTTTTTTCAATTCTAATTATAAAGTAATAGTCCTTTTCACAAAAAAGCTATCCATACAGTGACGGCATTTAATTATGAAAGTTGGCTAGGTAGCTGACCCTGTTAGTCCGTTTTTTCAAGAAAAGGAATAGGAGCATAACCTTTTTCCTCCGCTTAATGGATAACTATTTATTACCAATGGAGAATTCCTTCTCATCTCAAACGGAGTGATTGGATTTGCACCAATAGAAACCATAAATTCATAACATAATTAGGTAGATGATAGATCTTCATTTTTAGATACTAGTAAATTAAATGGCCGTTTTCCACTCTATCTATCCTAATTCATTGATAGTGGTCGTTGATACTTTTGCATTTAACTCATCATGATCTTAACTGAACGAGTCGCACATACACCCTAGTACATGTTCCTCGACGCTGAGGACATCCTCGAAGAGCGGGAGATTTCGTGACATTTCTTATTGGCTGTCTTGCGTTTCTAATAAGTTGTTTAATGGTTGGCATGGCGTGTCTATAGAATCTCATTCTAGATAGAATAGAGCGGGTTGGCTTAGATCGATCTTAACCTGATGGTTGATGATTATGAATGATTTCTATTCACACGGAAATTTCGAATTTTTAAACGGAATTCGTATATTTATATGGAATCCCTAGTATTTTCATTTTCGATCGCTACAAGATCAACGATGCCATGAGCTTGGGCTTCTGTTGCTGACATAAAAACATCCCTTTCCATATCTTCGGATATAACCCATAAAGGGTTGCCCGTTCTTTGTACATAAACTTTTGTGAGAGTTTCGCGAAGCTTCAATAGTTCTTCTGCTTCCAGGATAAAATCCCCTGCTTGTGCCTCGTAAAAAGAACTAGCAGGTTGGTGAATCATAACCCTGATGATATTGATATAACATCATGAATGGTTCTTCTATCTATATATTGCACGATTGGGTTAAAGTAAGGGGTAATCAAAATAACAATAAAAAATAGAATTAAACAACCGTACGGGCATCTTTTGTACATTGCATACGGCTCTGCAATGGAATTTATTTTTTCGATAGAATTTCTTCTATCGAAAAGAAGAAAAAGAACCCATCCGATCCAAATCGTTAAATGATCCATTTACCACCCTTCCTTTCGTAGTAGTAAAAAAGATACTATGATGGTTCTGTTGCTTTATATGTTTATCTATTTATCTCGTCTGTGGTTTAGCAATCCCAAAGTTTCTTTTTGATATGATCCAAGAAGGAGAAAATGATTTTTTCCATTTTTTTGACTCTTTCTCTCATAACATAAAAATAAGAAAGATACTTCTGGTGTGGAAGAATAATGGTTTGTGACGCTGAAATTGACTCTTGCTTGACACATAAAATCAACTTGGGAATAACCCTTCTTTCATACTACTATCTCGATACAAAATCTCATGTTAGAAAAAAAAAACACTAATGGTTTGTTCATATCGAACTCGAAGTGCCATGCTATTATTACTTATTCTTTATTTGATTCATATTCGATACAGCGAAGGCATAGTATTCTTTTTTTCTCAAATAAAAAAACTCATTGGCGCCAAGCGTGAGGGAATGCTAGACGTTTGGTAATTTCTCCTCCAACCAGAATGAAAGATCCCATTGAAGCGGCTAATCCCATACATACTGTATGTACATCCGGTGACACAAATTGCATAGTATCATAAATGGCTATTCCTGGTATTACCCATCCGCCTGGAGAATTTATAAACAAATAAAGATCCCTAGTATCATCTTCTATGCTGAGATATACCATGAGACCCACAAGTTGATTCGAGATCTCGCTATCAACCTCTTGGCCTAAAAAAAGTAATCTTTCTCGATGAAGTCGGTTGATTAGGGCAAAATTGTATCCCTGAGGAACCGTACGTGCACCTTTGGATGCATACGGTTCGAAAGAATTGCGAAAAAAAAATCAATGTATTGATTCCAGTCCTATTTCTTTTTTTTTTAACATGAGTTTTGCCCTCCTTCCCCTTCCCTATATTCTATAATGTAGAAAATCAAAAAGAATTTTATGAACTTATTGAACTAACTTCTCATTGATGTATTGTTTCATCGAAATTCAAATTACGATGTAATTTTCTTGTTCCTGAATGGACCCTTTCAATTCTTTTAGGTTCTTGTTCTACTCCGGGGGAAGATTTGCCCGAATTCCATTTGCGCATATAGGTCAAATGATTCCAGTACCACTTCTTTTTTTCAATTGTTTCATAACTTTTCCCAAAATATTCGATGTATTAATAATACTACTCCATTGGTAGACAGTTTTATATTATCCCTATAGTAAGTTATATTATATTCTATTTAATTATAGAATATTAAATTATTAATGAATTTAATAATTTATTAATACTTTAATTAAATTTCTATTTTATTTTTATATTCTTTATTTAATATTTCTTATTTATATTACTACAGTTACACTCCCATTCTATTACTTTTATTTCCAATTTGGTATTCTATGAACGGAGCCTGGATACTTTATCAGTCCAAGTAAACCATCAATTATTTTAATTGATAATATTCATCCCCAATAGGAATTATTGTGCTTCACGCTCCGAATTATTGATTGTTCAATCAATACAAGATTGAATATATATTTATTGGATTGGGCGAAATAGAGAATACTCGATCGGGGGAGATAACGGGGAAATACCATATGACCCATATGTCTGACAAGTCGCACTATACGTCAACCCAAGCTGCATCTTCCTCTCCAGGATTCCGAAAAGGTACTTTTGGAACACCAATGGGCATTAAGATAAAGAAAAAATGAAGTACTATACTTTACTTTAATATGGAAACGTAACAATGGTTTTATTGTCTTCATCATTTTTTCTCTTTCTTTTCTATTTTTATATTCTATATATTTTCTATTTTTTATATATTTTTTTATTATATATATTTATTATTTATTATATATATTTATTATATATTATATATATATTATATATATTATATCTAAATATTATATATTATATATATTATATATATTATTTAGATATAATATTTAGTATTAGTAGTATTAGCATATTTCTATATTCTAATCTAATATATTCTATTATTATTATATATTCTATATATTCATTCTATATTTTCTATTTTTATATCTTTTAATTTTCTTTCTATATTCTATATTAGAATTAGAATTTTATATTCTATATATTATAGAAAATAGAACTTAATATTATTAGATAGATATATTATTATCTAGAAAGAATTAGAGTATATATTAAGTATATAGATTCTAATTTAGAATATTAGTAATATTAGTATATAGATATATACTTTATATATAGGAATATAGGACTGGAAGGTTCATAGAGGAAGACAGAATGAATAAAGAAAAATTGTAACGAACGGGATCGATCGGATCAGCCGATTGTTCGAATGATTTCGAATTATAAAAAGTATCTATGCATTCTTTTTCCCTCAAAATCTTCCCATTGCGTATTGGTACTTATCGAGTATAGAATAAGATCTGTTTCTCTTTGTTCTTCTAAATATAAATAAATAGAATTGTTCCCTTCTCTTTCTATTTCAAATTCTTTCTATTTTCTTTCATTAAAAATAAAAGAAGGGAATACAAATAAATAGAAATCTTTTCCTATACAAAATCTACCGAACAGGTGAAATACACGGTCTAGTCTTTTCCAATGCGATAAAGTTACATAATGTCTATTTCTTTTTCAGAAAGGGGTATTTACATGGGTTTGCCTTGGTATCGTGTTCATACTGTTGTATTGAATGATCCCGGTCGATTACTTTCTGTCCATATAATGCATACAGCTCTAGTTTCTGGTTGGGCCGGCTCGATGGCTCTATATGAATTAGCGGTTTTTGATCCCTCTGACCCTGTTCTTGATCCAATGTGGAGACAAGGCATGTTCGTTATACCCTTCATGACTCGTTTAGGAATAACCAATTCGTGGGGGGGTTGGAGTATCTCGGGAGGAACTATAACGAATCCGGGCATTTGGAGTTATGAAGGCGTGGCAGGGGCACATATTTTGTTTTCTGGCTTGTGCTTCTTGGCAGCTATTTGGCATTGGGTGTATTGGGACCTAGAAATATTCTGTGATGAACGTACGGGAAAACCTTCTTTGGATTTGCCCAAGATCTTTGGAATTCATTTATTTCTCTCAGGAGTCGCTTGCTTTGGCTTTGGTGCATTTCATGTAACAGGCTTATATGGTCCTGGAATATGGGTGTCTGATCCTTATGGACTAACTGGAAAAGTACAATCTGTAAATCCAGCGTGGGGTGCGGAAGGTTTTGATCCTTTTGTTCCGGGGGGAATAGCTTCTCATCATATTGCAGCAGGTACATTGGGCATATTAGCGGGTCTATTTCATCTTAGTGTCCGTCCGCCTCAACGTCTATACAAAGGATTACGCATGGGTAATATTGAAACTGTGCTTTCCAGTAGTATTGCTGCTGTTTTTTTTGCAGCTTTCGTTGTTGCTGGAACTATGTGGTATGGTTCAGCAACTACCCCAATCGAATTATTTGGCCCCACTCGTTATCAGTGGGATCAGGGGTACTTCCAGCAAGAAATATATCGAAGAGTTGGGGCCGGACTAGCCGAAAATCTGAGCTTGTCGGAAGCTTGGTCTAAAATTCCCGAAAAATTAGCTTTTTACGATTACATTGGTAATAATCCGGCGAAAGGGGGATTATTCAGAGCAGGCTCAATGGATAACGGGGATGGAATAGCTGTTGGGTGGTTAGGGCACCCCATATTTAGAGATAAAGAAGGGCGCGAACTCTTTGTACGTCGTATGCCTACCTTTTTTGAAACATTTCCGGTAGTTTTGGTAGATGGAGACGGAATTGTGAGGGCCGATGTTCCTTTTAGAAGGGCAGAATCCAAGTATAGTGTTGAACAAGTAGGGGTAACTGTTGAATTCTATGGTGGCGAACTCAATGGAGTCATTTATAGTGATCCTGCTACTGTAAAAAAATATGCTAGACGTGCCCAATTAGGTGAAATTTTTGAATTAGACCGGGCTACTTTGAAATCCGATGGTGTTTTTCGTAGCAGTCCAAGGGGTTGGTTCACTTTTGGGCATGCTACGTTTGCTTTGCTCTTCTTTTTCGGACACATTTGGCACGGCGCCAGAACCTTGTTCAGAGATGTTTTTGCCGGTATTGATCCAGATTTGGATGCTCAAGTGGAATTTGGAGCATTCCAAAAACTTGGAGATCCAACTACAAGGAAACAAGTAGTCTGATACAAAATTCCTTTGCCATCTTTTGCCTATATTTTTTTATTTTATTCTAGTTTCTAGTATTTAGCTATTTAGTATTTCTAATTTGTTAATTTATATAATTAAGCTAGAATTTCTCTTTTCTATATTAATTGAATCTATTATATATTTCATTTCATATTAAATATTTACTAAATATATTAATCTAATTATTAATCTAATATACTAATACTAATATATAAATTAATATATAAATTAGATAAATATCTATTTATTTTCTATTTTCTTTCTATATTTTTATTATTTTTATGTTTTTTATGTATAAATAGATATAAATAGAATAATATATTCTATTAGACTATTAGAATAATATATAAAAAAAATTAGAAATAGAATAATAATAATACAATATAAATATAGAAGAAATATAATTAATAAGATATGACTATATCTATATATAGTATATATAAATACTATATAGTTAGTAATAGTAAATTCTAAATCTCAATTTAGAATTTCTTTAGTAAATGATCCAAAATGAATAGGTGTGGAAGCTATAATTGTAAACCACGATCGAATCTATGGAAGCATTGGTTTATACATTCCTCTTAGTTTCAACTTTAGGGATAATTTTTTTCGCTATCTTTTTTCGAGAACCACCTAAAGTTCCAACTAAAAAAATGAAATGATTTTTCATTATTTCCATTGAAATAATGAGCCCCATATTCATATTGGGGCTCATTACTTCAACTAGTCCCCATGTTCTTCGAAGGGATCTCTTAATTTTTGAGAGGGTTGCCCAAAAGCGGTATATAAGGCATACCCAGTAAAGCTTACAAGTAACCCGGATATGGAGATGGCGACTAGGGTTGCTGTTTCCATTTTTTAGATAATTTCAAGATCACAAAGGATCACGATAATGTCGTTTATTTACAACTACAACGGAATGGTATACAAAGTCAACAGATTTAAACCCATGATGAAAGAGGATTTATGGCTACAAAAACCGTTGAGAGTAGTTCTAGATCTGGGCCAAGACGAACTGGCGTAGGGAGTTTATTGAAACCATTGAATTCGGAATATGGAAAAGTAGCTCCAGGGTGGGGGACTACACCACTTATGGGAGTTGCAATGGCTCTATTTGCAATATTTCTATCTATTATTTTAGAAATTTATAATTCATCTGTTTTACTGGATGGAATTTCAATTAATTAGTTCATAAAAACTAGGAAGTCCTAGTTTTTAAATCAAAAAATATTATTTTACTTATACTTACTTAATGCTTAAAATACTTAATACTTCAACTTAATATTACCTAAGACTTGGATTTCATTCTGTTAGTTCGCTGGTAGTTCGATCGTGAAATTTATTTGTTTCGATATTTCATTTCCGGAATATGAGCGTGTGACTTGTTATAATTGATCCTATTGATAATACAGAGAATGGACCTGTCATCTCTATCAAGATGATTCTACCTCGTCAGATATTTATTCTAGTCTCTGGAGCACGGACTATATAGAATAGATCAAGAAAAGAAATATTTGAACTATGATTCATACCTATTATTCAGACCTCGCAACCGGATTAAAAAAAAAATGGAAATAGGTCTTTTCTAAATCAAACAATTTTTTCCTTCATACTTATTTTGACCAAAATAAACCTCTTTCTCTATATTTTGTTGAGTTATTACATTCATTGAAGAAGTGATGATCAAATGGTTTTTACTCAGAAAACCTTTGAGTTTAGCTTTGGCTTTCTTAAATCATCGTGGTTCTAGTATGAATCTGAGGTTTCAATTGATTCATAGGGTCTCAACAAGAGAATTCCTATCAAACAAAAAAAAAGATAGGGAAGAGAAGATTCAAGAGGCCTGTCACGATTAACATAAAGAAAGATGAATGAGCCAACTTGAGATTGTATTTCTTGGCATTATCATCACAAAGAAGAGATTCCGGATTTTTCTTACTTCGTATCTTTGGGTCAAATCGAGTCAAGCGGCTAAGCCACAAGAAGTTTTAAACTCTCTATTCCATATCCGTTGAACCCAGTATTTGTGTGTTTCGGCTTGAGCCGTACGAGATGAAATTCTCATATACGGCTCTCAGAGGGGGAGTCTTTCTTAGGTTACCTATCTCAATAAAGTATATGATTGGTTCGAGGAACGTCTCGAGATTCAAGCGATTGCAGATGATATAACTAGTAAATATGTTCCTCCTCATGTCAACATATTTTATTGTCTAGGGGGGATTACGCTTACTTGTTTTTTAGTACAAGTAGCTACGGGTTTTGCTATGACCTTTTACTATCGTCCAACTGTTACAGAGGCTTTTTCCTCTGTTCAATACATAATGACTGAGGTCAACTTTGGTTGGTTAATTCGATCAGTTCATCGATGGTCAGCAAGTATGATGGTTCTAATGATGATCTTGCACGTATTTCGTGTGTATCTTACAGGTGGATTTAAAAAACCCCGCGAATTAACTTGGGTTACAGGGGTGGTTCTGGCTGTATTGACCGCATCTTTTGGCGTAACTGGTTATTCCTTACCCCGGGACCAAATTGGCTATTGGGCAGTAAAAATTGTAACAGGCGTGCCTGAAGCTATTCCTATAATAGGATCACCCTTGGTAGAATTATTACGTGGAAGTGCTAGTGTGGGCCAGTCCACTTTGACTCGTTTTTATAGTTTACATACTTTTGTATTACCTCTTCTTACTGCCGTATTTATGTTAATGCACTTTCCAATGATACGTAAGCAAGGTATTTCGGGTCCTTTATAGAGAAGGCAGATCATAGATATTTGTAATTTATCATATCGGGGAGGAACAAGAGTCTTTCATTGCTACAAATATGGATTATTTCAAAATAAGGCATGTTATTTGGATACTTCTATTCAACTCTGAAGTATTGTTTATTTGATACGAATCGAATAGTTAAAGTATATTTTCCTAAAAGAGGATGGATTATGGGAGTGTGTGACTTGAACTATTGATTGGTCCATGCAGATATATGATTTTATCCGCTACGTTGGAATTCACAACCTAACGTGTCTCCGCATCCAACCATCACGTCAGTCCCTTTATGTAGCATAGGATAGGCCGGTTCGCTTGAGGAGAATATTTTCTATGATCATACCCGAATCATGTCATGCATGAACAGGCTCCGTAAGATCCCTAGAATAGAATGATCCAATGTTCTATTTATTCTACTTTTTTTATTTTTAGTAATTTTCTTATAATTAATTGATAGTAATCTTAGTAAGTTTTTTATAGTATGTAGATGCATTCATTTCCTCTGCATCGACCCTGAATCTATGATACTATTGGAGTTAAATAAGGGATCTAAGGAAGAACAGGGGCTATACTTTATTAGTAACAAGTAAAAATTTTGTATTTTTGTATGTAATACAATCGAGATATTGTGGGGATAAATACCAACCAAAAGACATGAGACAATCCAAAAAGCACTTGATCATGATCAAATTTGTAAGCCTACTTGGATATTGAGCATTTCCTTGTTGCCAGAACTGAATTCTTTGCAATGAATAATGAATCGTTGAAACTCGGGAAATGGAATTTGATAAATCTTTTCTTACATAGAGTCATTCTACATTATATATGTAGATATGTATGAAATATAGATCTTCTATGGACCTATTTATGTTCTATGGATCTATTTCTGTGATTCTTTTGATTCTTGCTCGAGCCGGATGATAAAAAATTATCATGTCCGGTTCCTTTGGGGGATGGATCCACAAGAATTCACCTATCCAAATAACAAAGAAACCTGATTTGAATGATCCTGTATTAAGAGCTAAATTGGCTAAAGGGATGGGACATAATTATTATGGAGAACCTGCATGGCCCAATGATCTTTTATATATTTTTCCAGTAGTAATTCTAGGCACTATTGCATGTAATGTGGGCTTAGCAGTTCTAGAGCCGTCAATGATCGGTGAACCGGCGGATCCGTTTGCAACTCCGTTGGAAATATTACCCGAATGGTACTTCTTTCCCGTATTTCAAATACTTCGCACAGTACCCAATAAGTTATTGGGTGTTCTTTTAATGGTTTCAGTACCAATAGGATTATTTACAGTACCTTTTTTGGAGAATGTTAATAAATTCCAAAATCCATTTCGTCGTCCAGTAGCTACAACAGTCTTTTTGATCGGTACCGCAGTAGCTCTTTGGTTAGGTATTGGAGCAACTTTACCTATTGAGAAATCCCTAACTTTAGGTCTTTTTCAAATTGATTAAACCGTGAAATACCACGACATAGGTATCTAAGGAAGATCCCCTTCTGGATCTTCCCTAGATACCTCAATCTTATTATGATCTATTCTGCAAATATATGGACCGTGTCGGAGATTAAAAACTTATTCTATTCTTTATTTATTTCTAAAAACTAAAAAAAGAAAAAATTCCAATGGATTTAAAATGAAAACTTTTTCTTAGGTAAATTGATTGCAAAATACTTCTGTAGAGTGCCCAATATCTGTTTTACATCTTCTATGCGAAAATATTCCATTTTCATAAGATCTTCTTGACTGTGACTCAAAAGGTCCAATAATGTATGTATATTGGACCTTTTGAGACAATTATAGGTCTTGGAAGACAATTCTGATTGGTCAATAAAAATATATGTCAATGGAATTCCTTTTTTGTTTTTCTTGAGATTAGTGAATCTGTCTTGAAAGGAAAAAAGGGGTAGATTCCATCTGTTTTCATTTTCCTCGAAATTCATGTCCTCTTCCTCTGCATGTAGAAAAGGAATCAATAAATCAATCAAATTACGAGAAGCTTCATAAAGTGCTTCTTTAGGAGTTAAACTTCCATTCGTCCATATTTCTAGAAAGAGTATCTCTTGTTTTTCATCCCCATTCCCATAAGAATGAATACTATGATTCGCATTTCGAACAGGCATAGATACAATATCTATAGGATAACTTCCATCGTGAGAGTCGTTTGTGGATTTCATATGATATCCGCGATCTCTCTTGATTTGTAATTCAATACACAAATCAATTGGTTCTGTCAGGTTAGCTATATGCTGTGTCGTGTCAACTATTTCTACAGAAGGTGGTGAGATTATATCTTGAGCTGTTATGTATTTTGGACCCCTGATGCAAATGGATGCGTCCCTAACTCCATAGAGATTACTTCTCAATACAATTTCTTTCAAATTTATTAAAATCTCATGTACTGATTCTTCAATACCTGCTATCGTAGAATATTCATGCAGCACATTTTCAGATGTTGCACGTGTGATACATGTTCCTTCTATTTCTCCAAGTAAAGCCCTTCGCATGGCAATACCTATGGTATCGGCTTGACCTTTCATAAGCGGGGACAAAACGAAACGACCATAATAAAGACGCTTGCTGTCTACTCTTGATTCAACACATTTCCACTGTAGTGTTCGAGTGGATCCTGTTACTTCTTCTCGAACCATACTCTTATTTTTCTTTTATTTATTATTATTGGATCAGATCATTGAATAATTTATTTCTCTTGGAATCTCTTGAATTCTTATTTCTACACACGTCTTTTTTTAGGGGGGCGACATCCATTATGCGGCATGGGTGTTACATCACGTACGAAACTTAATAGCATCCCATTTCTACGAATGGCTCGTAATGCCGCATCTCTTCCGAGACCTGGACCCTTTATCATAACTTCTGCTCGTTGCATACCCTGATCAACTAATGTACGAATAGCATTAAATGCTGCGGCTTGAGCAGCATAGGGTGTTCCTTTTCTTGTGCCTCTGAATCCAGAAGTACCTGCGGAAGCCCAAGAAACCACCCGACCTCGTACGTCTGTAACAGTTACAATAGTATTGTTGAAACTCGCTTGAACATGAATAACTCCTTTTGGTATTCTACGTTCATTCTTATTTGAACCAATACGTGCATTCTTACGTAAACCAATTTTTGCTATAGGTTTTGTCATATTTTATTAGATCATATTCATAAGAATAAAATAAAAAGAAAGAAGAATCAGAAATATACATAAAGATACGGATACAGGAATATCCATTTCATATGAAAACGAATCCTTTCTTCTTTCTTTTTACATGTACATGTAACATGTACATGGGTTTTTATTTGAAAAAGTTCTTGATTTAGAACTTGAGAAGGATTACCCCTGTCTCTGTTTATGTCTCGGATTGGAACAAATGACTCTAATTCGTCCCCGCCTACGAATCAAGCGACATTTTTCACAAATTTTACGAACAGAAGCCCTTATTTTCATATTTATCATTCCTTACTTTCATTCTGAATCTATTTTTTTTTTTGAAATAAAAATCAGTTTATTGCATTTTTGAACTTCGAATTATATCCCTACGAAAAGGATGTTTAAAAGAATGATCTAATCGTTCGAATCTTTTTTGCGAAGTCTATAAATTATACGTCCCCTGGTTGAATCATAACGACTCATTTCAATTTTGACTCTATCTCCGGGTAGTATACGTATAAAACTGCGCCGGATTCTTCCTGAAATATAACCTAGAATTAGATCTTCATTATCTAACTGAACTCGGAACATACCGTTGGGAAGTGATTCAGTAATTAAACCCTCATGAATTAATTTTTGTTCTTTCATTCCAGGGAACCCCCCTTTAAGTATCAACTAATAGAGGAAGAGTTCTATAAATTCACTTCTCCTCTCTATTTTACAAATAGTAAGTTCGAGAGAAAATTAGGGTACCCCAGGAGAATCACCATATATAACACAAAATTTCTCCTCCAATTTTTTCTAGTCGAGCTTCTCGATCTGTCATTATACCTCGAGAAGTAGAAAGAATTACAATTCCCATTCCGCCTAAAATCTTAGGAATTCGTTGATAGTTGGAATAGATTCGTAGACCGGGTCGGCTGATACGCTTTAAAATTATTTTATTTCCTTTCCTAGTCTTTCTATGTCGTAAGGTTAAAACCAAGAAATTTTTTTGACTTTCTCTATGTTTTCGAACATTTTCAATAAAACCTTCTCGTAAAAGTATTTTCACAATGTTTTTAGTGATATTAGTAGATACTATTTGAACTCTTCCCTTTTGATCTATGTCAGCATTTCTTATAGAAGTTAATATATCGGCAATAATGTCCCTACCCATGACAAACTATAGTTATTGGTGCCTCCTAATTTTGATATAATAAACATGCTTCTTTTTTGTTTTATTTTTTATTGAATTTTTTTTTGAAATTCTTAAATTATAAAAAATTCTTAGAAATTTAAAGTATATGCATGAGACACAATCTATTCTATTAATCGGATCTATTTCAGATATTTGAATATTATAAATATAAATATTCCATATATAGATTATACATAGGATATATATAGATAGGATATATCCTATCTATAATCTATAAGACTTCGGGTGCTAATGAAACTATTTTAGTAAAATTCAATTGTCGCAATTCTCGAGCAATCGCACCAAAAATTCGAGTTCCTTTTGGATTTCCTTCTTGATCAATGATAACCGCTGCATTGTCATCATATCGTATTATCATGCCGTTATCACGTTTGAGTTCTTTACACGTGCGTACAATCACAGCTCTAATTATTTCTGATCTTTCTATAGGCATGTTGGGCACTGCTTCTTTGATTACAGCAACAATAACATCGCCAATATGAGCATATCGGTGATTACCAGTTCCTATGATTCGAATACACATCAATTCTTGAGCTCCACTGTTATCCGCTACATTCAAAAGGGTCTGAGGTTGAATCATATCATTTTTATTTTAATCTCTTATTTCAATGCAAGGGATAATGGAAAAAGAAATATTGTCTGTCCAGAGATAAAGAAATCCGTGGTTGTTTTTTCATTCTCAATACTCCTTCTTTTTTTACTTTTGTTTACCTATCCTGAAATAACAAATTGAGTTCGTATAGGCATTTTGCATGCAGCTATTTCCATAGCAGCTTTGGCTACAGTTTCTGATACTCCACTCATTTCATAAAGTATTCGGCCCGGTTTAACAACAGATACCCAATATTCGGGGGATCCCTTGCCCGAACCCATACGTGTTTCTGTAGGTCTTACAGTAACAGGTTTGTCGGGAAAGATACGTACCCATATCTTTCCACCACGACGCGCATATCGTGTCATTGCTCTTCGCCCTGCTTCTATTTGTCTAGCTGTGATCCAAGCAGGTTCAAGTGCCTGAAGAGCGTATCTGCCAAAACAAATATGATTGCCTCGGCAAGATATTCCTTTCATTCTACCTCTATGTTGTTTACGAAATCTGGTTCTTTTGGGGTTATAGTTGATGGTTATTTCTGAATTCCATCTCTACTGCAAAGCCGGACATGAGAGTTTCTTCTCATCCAGCTCCTCGCGAATGAAATGATTCAATAATATTACATATACACATGTATTTATGTATTTCTTTCATTCTAAGAAAGAATATACTAATTTTTTTATATTGAATTTGTTACATAGGTAGCTTTATATATAACTAGGCGTGTTTGTTTATATATAATGCTTCTTTCTTTTATCAAAATTTCTAAAGTATTTTACTTTACTACCTCTATTTAATCACGCCAACAGTCATCCAATAAATGAAATTCTTAAATTAAATCAAAATAAAGAAAGGTTTCGCGGGCGAATATTGACTCTTTCCTCCTTCATTTGTAGGGTCAATTCATGACCATTTAGAAGAAATCCATTTTTTCTTGGTTGATTCCGCCATCCTACCCAAAGAATAATTAGGATTGATTTGTTTTCAAGAAAATCCTATGTAATCACAGGTTCCATCGTTCCCATAGCTTCTCTATTAATGCTTAGGCCTGAACTCTGCAATGGAGCTCTCAACAAAATCTGTTATTTGTTTCCGAGTCAATCTCCTCAGTTTTTATTAACCCGAAGCTCAATTAAATTATTCTCTATTTTTTATTATTTCTATTATCTTCTTTTTCTATCTTATTACATACATAATAGATTGACTCTATTATCCATATTGATTAGATTATTTATATTATTATTTTAATTTAATTTCTTTTATTATATTTCTTATATTTTCTTCTATGTTTCTATTTTCTTTATATTTTTTATTTGTATATTTCTTATTCTATTGTAATTGTATATTTTGTATTTTTATTTTATGTTGATGCTTTATAACACTGCCTTTTTTATGGGGTAATTCTTCATAAACCATACATATGGGAATCATATATCATTGAGATCTTTATTCTATCTTTCTATCATCCTTCCATTTTTCCACATCCCTTTTTTTTTTCACAATTCATAATCATATTTCTTTTTTATGAAAAGAATTTAAGTTGCTACAACTATATGATCGATTCAGTCATATAGTGACTTTTTCTTGGGATCTCGACAATACGAAGCAATAAGTTGGTTATTAGTTTTGAGTTTCTTTAGTTTTGATAGTTACTAAGTTTATAGTGGGGTCAGCCTTTTTTTTTTTTCAATCTCAATTCTCAACTCTAAAGAAAAAACTAACGAGTCACACACTGAGCATAGCAATTATACTAAAATCTAAATTAAATTTAAATAAAGGGTAAATCAAATTTTTATTCAACCTTATAGAATTATAATTGTTCGTTTTTCTTTGATTAAGAAAAAAAAAAAAGAAAAAGAAGAAAAGAGTTTCTAAATTTTTTCTATCGATGAGCAGAATGGCGAGATAAAGAAAGGTCCATTATTCTTATTTTCTTATTCTTGGTTTACAAATATCCAAATTTTGATGCCTAAGACTCCATAGATAGTTCTAATTGTATGGGAACAGTGATCAATTTTAGCGCGAATTGTTTGTAAAGGAACCCTGCCTTCTCTGATCCATTCGACACGTGCAATCTCTTTTCCGTCAATACGCCCTGCAATTTGCACTTGAATTCCTTTTGTACCCGTTTGTTCAGTTAATTCAATAGCTTTTTTCATTGCCTTTCGAAATGAGACTCTATTTTTTAATTGTAAAGCTATATATTCTGCAATAATATTAGGTTGTCCATAAGGCTTTTCAATTCTTGTGATAGCAATGTTGAGTCTACGATTTACAGAATGAAACTCTTTTTGTACATTCATCTGTAATTCTTCGACTCCCCGTGTTCGTCCTTCTATTAATAAATTGGGAAATCCAATATAGATTATGACCTGAATCAAATCTATTCTTTTTTTAATTCCTATATGGACAATTCCTTCGAAACCCGAGGATATTTTCTGATTTTTTTTTACATAGTCCTTAATACATTTCCGTATTCTTTCATCTTCTTGTAGACCCATGGAAAAATTCTTTGGTTTTGCGAACCAAAAAGAATGATGACTTTGAGTTATTCCAAGTCTGAAACCAAGTGGATTTATTTTTTGTCCCATATTTTTCTATTATTTTTCCATCCATTTTTTTTCTAAATAGTAATCTAAATTTTAGATTTTTCTTTTAAAAAAATCTTTATATGACAAGTGGTTTTTTTTATCAGATAACTACGCCCTCGAGCCCGGGGTCTTAACTTTTTCACAATAGAACCTCTATTGACTTCAGCTTTACTAATAAATAAATCGGCTTCATTCAAACCCATATTATGACTAGCATTTGCTGCTGCAGAATAAACTAATTTTAAAATTGGATAAGATGCCCAATAAGGCATTAGTTCCAGTATCATGAGTGTTTCCTCATAAGAACGTCCGCGAATCTGATCAATTATTCTTTGTGCTTTGAAAACAGACATACAGATATGTTGAGCTAAAACTTTTGCTTCTCTATCCGAATTTTCGTTCTTTATCATAAAAGTTCTCCCCCGCCAATGAATGATAAGTGCCTAGGTGAAGTATAGTATAAGATAAGTCAGAAAAGTATAAGTCTTATTAGTATACTAGTAGTATACTAGAAAAAGAAAATAAATATACTTATACTCTTACTATAAGATTACTATAAGATAAAGACTCTTAAGTCTAAATACTATTAAGATAAGGCTTTTCACATGAATACTTAGTAGAACGACTAACGACGAGATTTATTATCGTTTCTCGCGTGTCTCACGAAAGTGAGAGTAGGTGCGAATTCTCCCAATTTGTGACCGACCATACGATCTGTGATATAAATAGGTAAATGTTCCTTTCCATTATGAATAGCGATTGTATGGCCAATCATTGTGGGTATAATGGTAGATGCCCGAGACCAAGTCACTATGATTTCTTTCTCCTCCCTCCTGTTGAGTTTTTCAATTCTTCCCGATAAATGATTAGCTACAAAAGGATTTTTTTTTAGTGAACGTGTCACGGCTGATTACTCCTTTTTTTACATTTTTAAAATTGGCATTCTATGTCCAATATCTCTATCTTAATCTGAAGTATAATGATGAATGGAAAAAAGAGAAAATCCTTTAGCTAGATAAGGGAAGGGGCGGATGTAGCCAAGTGGATCAAGGCAGTGGATTGTGAATCCACCATGCGCGGGTTCAATTCCCGTCGTTCGCCCATCACATTATTTCCAATTCCAAAAATTCGATTTTCAATGTTCCTATTTACGGCGACGAAGAATAAAACTATCACTATATTTGTTCCTTTTCCTACTTCTTCTTCCAAGCGCAGGATAACCCCAAGGGGTTGTGGGTTTTTTTCTACCAATTGGGGCTCTCCCTTCACCGCCCCCATGGGGATGGTCTACAGGGTTCATAACTACTCCTCTTACTACAGGACGCTTACCTAGCCAACACTTAGATCCAGCTCTACCCAAACTTTTTTGGTTCACCCCAACATTACCCACTTGTCCGACTGTTGCTAAGCAGTTTTTGGATATCAAACGGACCTCCCCAGATGGTAATCTTAATGTGGCCGATTTACCCTCTTTTGCAATCAGTTTCGCTACAGCGCCTGCTGCTCTAGCTAATTGTCCACCCTTTCCAAGTGTGATTTCTATGTTATGTATGGCCGTGCCTAAGGGCATATCGGTTGAAGTAGATTCTTCTTTTTTATCAATAAAATCAATAAAAACCCCTTCCCAAACTGTACAAGCTTCTTCCAAAGCATACGGCTTTCTAGATGTATATGATGATATCTAGACAGATGGATCTTATATGATGAAGTACCACATGAGTGGATATATAGGAATCCAAATCTGCCGAATCACTCATGTTATGATCTTCTACATCCTAGGTCTCCCCGTTCCGTCATCTGGCTTATGTTCTTCATGTAGCATTCAGACCGGATGACTCTATGAAATTACGTCGATACTTCCACATATTACGGGTAACGTAGGAGACATCTCTATTTTTCCCCGGGGGGTCTTTCTAATTACCACTGCTTAGCTTTCAATTCGCCTCTGACCATCAAATGAAATGTGAATAACCCGTCCTCCTCTCTTTGAAACAAGGGGCGCTTCCGGTTCTGTGCGCGCTTCAAACAATTTTGTCTTCTCCATATTACCATATCTCTAGAGTCAATAATTTTCTATGAGGAACTACTGAACTCAATCACTTGCTGCCGTTACTCAACAGTTTTCTGTTGAGGTCTATCCCGTAGAGGTACTCCAATTGGATCAGTGATCGATTTCTAGGTTTCGTCGTAAACCTAATTGGTTACTTCCAATTACGTAAATCAATAGTTCAAACCGCACTCAAAGGTAGGGCATTTCCCATTGATATAGGAACTTCTGTACCAGAAACAATGGTATCTCCAATTATAGCCCCTCTGGGATGTAAAATATATCTCTTCTCACCATCCCCATAGTGTATGAGACAAATGTATGCATTTCGATTAGGGTCATATTCTATGGTTACGATTCTACCAGATATCTCTTTTTCATTCCGTCGAAAGTCGATTTTACGGTATAGACGCTTATGACCTCCCCCTCTATGCCCTGCGGTAATGATCCCTCTGGCATTACGACCTTTACCACAACGATGCTGTCCATAGATCAAATTATTTCGTGGATTGGATTTCACTTGACTGTCTACGGCTCCATTGCGTGTGCTCGGGGTAGAAGTTTTGTATAAATGTATTGCCGTGTTATTAAGTCTTTTGCTTTAAGTTATTTTCTCTATAAGAGGTGGAATAGAATAACCCGGTTGAAGCGTAATGATCATACGTCTGTAATGCATTGTATGTCCCATAATAGGTCCCATCCTTCTACCCTTTCCCGGGAGTCGATGACTATTCATAGCTATTACCTTGACACCAAAGAAGAGTTCGACCCAATGCTTTATTTCTGTCCTAGTTGATCCTGATTCGACATTAGAAGTATATTGATTGTTCCCCAATAACCGAATACTTTTTTCTGTAAATACTGCATATTTGATTCCATCCATAAATCCATTTTATTCCCTATGAGTTCCAGTATCGATAAGAATTCTAGTTCTTACTGTTCATATGTTATGGTATGAATATACCATACCAATTCGCTATGTATGGATGATGAGATTCCATTGATACAGAGCCAATTCCAATAGACTTATTGAATGTTCCCATTGGCGTGCATCCAGCAGGAATTGAACCTACGAATTTGCCAATTATGAGTTGGGCGCTTTAACCATTCAGCCATGGATGCTTAACGGGGATCATCGTACATCGTGAATAACCAAATTCCAATTGAAATGAAATCTTTAGGAGGAATCAATGAAACGACATCAATTCAAATCCTGGATATTCGAATTGAGAGAGATCAAGAATTATCACTATTTCTTAGATTCATGGATCAAATTCGATTCAGTGGGATCTTTCACTCACATTTTTTTCCACCAAGAACGTTTTATGAAACTCTTTGACCCCCGAATTTGGAGTATCCTACTTTCACGTGATTCACAGGGTGCAACAAGCAATCGATATTTCACGACCAAAGGTGTAGTACTGCTTGTAGTAGTGGTCCTTATATCTCGTATTAACAATCGAAAGATGGTCGAAAGAAAAAATCTCTATTTGATGGGGCTTCTTCCTATACCTATGAATTCCATTGGACCCAGAAAGGAGACATTGGAAGAATCTTTTTGGTCTTCCAATAGAAATAGGTTGATTGTTTCGCTCCTGTATCTTCCAAAAGGGAAAAAGATTTCTGAGAGTTGTTTCATGGATCCGCAAGAGAGTACTTGGGTTATCCCAATAAATAAAAAGCGTATCATGCCTGAATCTAACCGGGGTTCGCGGTGGTGGAGGAACCGGATCGGAAAAAAGAGGGATTCTAGTTGTCAGATATCTAATGAAACCGTAGCTGGAATTGAGATCTCATTCAAAGAGAAAGATAGCAAATATCTGGAGTTTCTTTTTTTATCCTATACGGATGATCCGATCCGCAAGGACCATGATTGGGAATTTTTTGATCGTCTTTCTCCGAGGAAGAAACGAAACATAATCAACTTGAATTCGGGACAGCTATTCGAAATCTTAGGGAAAGACTTGATTTGTTATCTCATGTCTGCTTTTCGTGAAAAAAGACCAATTCAGGGGGAGAGTTTCTTCAAACAACAAGGAGCTGGGGCAACTATGCAATCCAATGATATTGAGCATGTTTCCCATCTCTTCTCGAGAAACAAGTGGGGTATTTCTTTGCAAAATTGTGCTCAATTTAATATGTGGCAATTCCGCCAAGATCTCTTCGTTAGTTGGGGGAAGAATCAGCACGAATCGAATTTTTTGAGGAACGTCTCGAGAGAGAATTGGATTTGGTTAGACAATGTGTGGTTGGTAAACAAGGATCGGTTTTTTAGCAAGGTACGGAATGTATTGTCAAATATTCAATATGATTCCACAAGATCTATTTTCGTTCAAGTAAAGGATTCTAGCCAATGGAAAGGATCTTCTTCTGATCAATCCAGAGATCATTTCGATTCCGTTAGAAATGAGAATTCAGAATATAACACATTGATCGATCAAACAGAGATTCAGCAACTAAAAGAGAGATCGATTCTTTGGGATCCTTCCTTTCTTCAAACGGAACGAACAGAGATAGAATCAGATCGATTCCCGAAATGCCTTTTTGGATCTTCCTCCATGTCCTGGCTATTCACGGAACCTGAGAAGCGGATGAATAATCATCTGCTTCCGGAAGAAATCGAAGAATTTATTGGGAATCCTACAAGATCAATTCGTTCTTTTTTCTCTGACAGATGGTCAGAACTTCATCTGGGTTCGAATCCTACTGAGAGGTCCACTAGAGATCATAAATTGTTGAAGAAAAAACAAGATGTTTCTTTTGTCCCTTCCAGGCGAGCGGAAAATAAAGAAATGGTTGATATATTCAAGATAATTACGTATTTACAAGATACCGTCTCAATTCATCCTTCGGAACCAGATCCGGGATGTGATCTGGTTCCGAAGGATGAACCGGATATGGACAGTTCCAATAAGATTTCATTCTTGAACAAAAATCCATTTTTTGATTTCTTTCATCTATTCCATGACCGGAACAAAGGGGGGTACGCGTTACGCCACGATTTTTTTGAATCAGAAGAGAGATTCCCAGAAATGGCGGATCTATTCACTCTATCAATAACCGAGCCGGATCTGGTGTTTCATAGGGGATTTGCCTTTTCTATTGATTCCTACGGGTTGGATCAAAAAAAATTCTTGAATGAGGTATTCAACTCCAGAGATGAATCGAAAAAGAAATCTTTATCGGTTCTACCTCCTCTTTTTTATGAGGAGAATGAATCTTTTTCTCGAAGGATCAGAAAAAAATCGGTCCGGATCTACTGCGGGAATGAGTTGGAAGATCCCAAACTAAAAACAGCGGTATTTGCTAGCAACAACATAATGGAGGCAGTCAATCAATATAGATTGATCCGAAATCTGATTCAAATCCAATATAGCACCTATGGAAGAAATGTATCGAATCGATTCTTTTTAATGAATAGATCCGATCGCAACTTCGAATATGGAATTCAAAGGGATCAAATAGGAAATGATACTCTGAATCATATAACTATAATGAAATATACGATCAACCAACATTTATCGAATTTGAAAAAGAGTCAGAAGAAATGGTTTGATCCTCTTATTTCTCGAACTGAGAGATCCATGAATCGGGATCCTGATGCATATAGATACAAATGGTCCAATGGGAGCAAGAATTTCCAGGAACATTTGGAACATTTCGTTTCTGAACAGAAGAATCCTTTTCAAGTAGTGCAAGTAGTGTTCGATCGATTACGTATTAATAAATATTCGATTGATTGGTCCGAGGCTATCGACAAAGAAGATTTGTCTAAGTCACTTCGTTTCTTTTTGTCCAAGTCACTTCCCTTTTTCTTTGTGAATATCGGGAATATCCCCATTCATAGGTCCGAGATCCACATCTATGAATTGAAAGGTCCGAATGATCAACTCTGCAATCAGTTGTTAGAATCCATAGGTGTTCAAATTGTTCATTTGAAGAAATTGAAACCCTTCTTATTGGATGATCATGATACTTCCCAAAGACCGAAATTCTTGATCAATGGAGGAACAATATTACCATTTTTGTTCAAAAAGATACCAAAGCGGATGATTGACTCATTCCATACTAGAAAGAATCGCAGGAAATCCTTTGATAACAAGGATTCCTATTTCTCAATGATATCCCACGATCGAGACAATTGGCTGAATCCCGTGAAACCATTTAATAGAAGTTCATTGATATCTTCTTTTTATAAAGCAAATCGACTTCGATTCTTGAATGATCCACATCACTTCTGGTTCTATTGTAACAAAAGATTCCCCTTTGATGTGGAAAAGACCCGTATCAATAATTATGATCTTACATATGGACAATTCCTCAATATCTTGTCCATTCGCAACAAAATCTTTTCTTTGTGCGTCGGTAAAAAAAAATACCTTTTTTTGGAGAGAGAGACTATTTCACCAATTGAGTCACAGGTATCTGACATCTTCATACCTAACGATTTCCCACAAAGTGGTGATGAAACGTATAACTTGTACAAATTGTACAAATCTTTCCATTTTCCAATTCGATCCGATCCATTCGTTCGTGGAGCTATTTACTCGATCGCAGACATTTCTGCAACACCTCTAACAGAGGAACAAATAGTCAATTTGGAAAAAACTTATTGTCAGCCTCTTTCAGATATGAATCTATCTGATTCAGAAGGGAATAACTTGCATCAGTATCTCAGTTTCAATTCAAACATGGGTTTGATTCACACTCCATGTTCTGAGAAGTATTTACCATCCGGAAAGAGGAAAAAACGGAGTCTTTGTCTAAATAAATGCGTTGAGAAAGGGCAGATGTATAGAACCTTTCAACGAGATAGTGCTTTTTCAAATCTCTCAAAATGGAATCTGTTCCAAACATATATGCCATGGTTCCTTACTTCGACAGGGTGCAAATATCTCAATTTCACCCTTTTAGATACTCTTTCAGACCCATTGCCGATACTGAGTAGTAGTCAAAAATTTGTATCCATTTTTCATGATATGATGCATGGATCAGATATATCACGGCCAATTCCTCAGAAGATTCTTCCACAATGGACTCTGATAAGTGAGATTTCGAGTCAATGTTTACAGAATCTTCTTCTGTCCGAAGAAATGATTCATCGAAATAATGAGTCACCCGTTCCATTGATATGGGCACATCTGAGATCAACAAATGCTCGGGAGTTCCTCTATTCCATCTTTTTCCTTCTTCTTGTTGCTGGATATCTCGTTCGTATACATCTTCTCTTTGTTTCCCGAGCCTCTAGTGAGTTACAGACAGAGTTAGAAAAGATCAAATCTTTGATGATTCCATCATACATGATGGAATTTCGAAAACTTCTGGATAGGTATCCTACATCTGAACTGAATCCTTTCTGGTTAAAGAATCTCTTTCTAGTTGTTCTGGAACAATTAGGAGATTCTCTGGAAGAAATACGGGGTTCTGCTTCTGGTGGCAACATGCTATTGGGTGGTGGTCCCGCTTATGGGGTCAAATCAATACGTTCTAAGAAGAAATATTGGAAGATCAATCTCATCGATCTTGTAAGTATCATACCAAATCCCATCAATCGAATCATTTTTTCGAGAAATACGAGACATCTAAGTCGTACAAGTAAAGAGATCTATTCATTGATAAGAAAAAGAAAAAACGTGAACGGTGATTGGATTGATGAGAAAATAGAATTCTGGGTCGCGAACAGTGATTCGATTGATGATGAAGAAAGAGAATTCTTGGTTCAGTTCTCCACCTTAACGACAGAAAAAAGGATTGATCAAATTCTATTGAGTCTGACTCATAGTGATCATTTATCAAAGAATGACTCTGGTTATCAAATGATTGAACAACCGGGATCAATTTCCTTACGATACTTAGTTGACATTCATCAAAAGGATCTAATGAATTATGAGTTCAATAGATCCTGTTTAGCAGAAAGACGGATATTCCTTGCTCATTATCAGACAATCACTTATTCACAAGCCTCGTGTGGGGCTAATAGTTTTCATTCCCCATCTCCTCATGGAAAACCCTTTTCGCTCCGCTTAGCCCTATCCCCTTCTAGAGGTATTTTAGTGATAGGTTCTATAGGAACTGGACGATCCTGTTTGGTCAAATACCTAGCGACAAACTCCTATGTTCCTTTCATTACGGTATTTTCGAACAAGTTCCTGGATGACAAGCCTAAAGGTTATCTTATTGATGATATTGATGATAGTGACGATATTGATGATAGTGACGATATTGATGATGACCTTGATACGAAGCTGCTAACTATGACGAATGTGCTAACTATGTATATGACGCCGAAAATAGACCGATTTGATATCACCCTTCAATTCGAATTAGCAAAAGCAATGTCTCCTTGCATAATATGGATTCCAAACATTCATGATCTGCATGTGAATGAGTCGAATTACTTATCCCTCGGTCTATTAGAGAACTATCTCTCCAGGGATTGTGAAAGATGTTCCACTGGAAAGATTCTTGTTATTGCTTCGACTCATATTCCCCAAAAAGTGGATCCCGCTCTAATAGCTCCGAATAAATTAAATACATGCATTAAGATACGAAGGCTTCTTCTTCCACAACAACGAAAGCACTTTTTCATTCTTTCATATACTAGGGGATTTCACTTGGAAAAGAAGATGTTCCATACTAACGGATTCGGGTCCATAACCATGGGTTCCAATGCGCGAGATCTTGTAGCACTTATCAATGAGGCCCTATCAATTAGTATTACACAGAAGAAATCCATTATAGAAACTAATACAATTAGATCAGCTCTTCATAGAAAAACTTGGGATTTTCGATCCCAGATAAGATCGGTTCAGGATCATGGGATCCTTTTCTATCAGATAGGAAGGGCTGTTACACAAAATATACTTCTAAGTAATTGCCCCATAGATCCTATATCTATCTATATGAAGAAGAAATCATGTAAGGGAGGGGATTCTTATTTGTACAAATGGTACTTCGAACTTGGAACGAGCATGAAGAAATTAACGATACTTCTTTATCTTTTGAGTTGTTCTGCCGGATCGGTCGCTCAAGATCTTTGGTCTTCATCCAGACACGATGAAAAAAATTGGATCACTTCTTATGGATTCGTTGAGAATGATTCTGATCTAGTTCATGGCCTATTACTATTATTACTATTAGAAGTAGAAGGCACTCTGGCTCTGGCGGGATCCTCACGGACAGAAAAATATTGTAGTCAGTTTGATAATAATCGAGTGACATTACTTCTTCGGTCCGAACCAAGGAATCAGTTAGATATGATGCAAAATGGATCTTGTTCTATCGTTGATCAGAGATTTCTATATGAAAAATACGAATCGGAGTTTGAAGAAGGGGAAGGGGCCCTCGATCCGCAACAGATAGAGGAGGATTTCTTCAATCACATAGTTTGGGCTCCTAGAATATGGCGCCCTAATCTATTTGATTGTATCGAAAGGCCCACTGAATTGGGATTTCCCTATTGGACTGGGTCATTTCGGGGCAAATGGATCATTTATCATAAAGAGGATGAGCTTCAAGAGAATGATTCGGAGTTCTTGCAGAGTGGAACCGTGCAGTACCAGACACGAGATAGATCTTCCAAAGAACAAGGCTTTTTTCGAACAAGCCAATTCATTTGGGACCCTGCGGATCCATTCTTTTCCCTATTCAAAGATCAGCCCTCTGTCTCTGTGTTTTCACGTCGAGAATTCTTTGCAGATGAAGAGATGTCAAAGGGGCTTATTGCTTCCCAAACAAATCCTCCTACATCTATATATAAACGCTGGTTCATCAAGAATACGCAAGAAAAGCACTTCGAATTGTTGATTCATCGCCAGAGATGGTTTAGAACCAATAGTTCATTATCTAATGGACCTTTCCGTTCTAATACTCTATCCGAGAGTTATCAGTATTTATCAAATCTGTTCCTATCTAACGGAACGCTATTGGATCAAATGACAAAGACATTGTTGAGAAAGAGATGGCTTTTCCCGGATGAAATGAAACATTTGATTCATGTAACAGGAGAAAGATTCCCCATTCCTTAGCCGTAAAGATATGTGCCCATGAAAAGGGGATTAAGTGGAACAGAATTGGCCGGATGGTAGAGTCGTGGAAACACTTGTTTCTTCCATCTTTTTGGCCTTAACTCCGTGGAACAATATGCTACTGCTGAAACATGGAAGAATTGAAATCTTAGATCACTATGTGTGGATGATATGAACTGCTTAAACAAGAATTCTTGAACGGCGAAAGAGCCTATTACTCGCTACATCAAACAATTTATACTAATGAAACCATGTAAATCCATCGGAAAATACGCATGTCCGCTGAAATGGTTGTTGCTATCTGCTCCAATAACGAATCATTGGTTTCATTGAATAACTAAAGAAGATAGATAGACCTTTCTCTTCGTCTCAGGTCGATGGATCTCCTCAATTGGAAGATCTCCCATACGGATAATACACATTCCAGTTGACCGAGCCTAATTCTAATTGCTTTGTTCCGAAGCAAAGATATCCACGGAGGCGGGTTCGTCCTATTCAGATATTCACGACCAAGAGCCTCTTTCGGATAGGCCCTGAAAGGAGAAGGAAGGCTGGAATGCCAACAGACGTCTGTCTATTCTCTAATTCACCCGACCCGATAGTACCCATTTTGAGAACGTCCAGTGCCAAAGTCACTGAATGGGTAAGTCGCCAATCCCTAATGTAATGTACTTTCTTTGCTGGGTTACGGGTACTGGTGGGTATTTTACCAGAGGTTTTTCTATCAATCTACCTTGTGCGATTCCTGTTGAATCCTATACTCGGGGGGTGCGCGCAGGGCGGACGATTTCCAAACGGACTCCTATAGAGAAGATCGCCAAGATTTCGTGATCCGCTGCCGATTACAACAGCTCGGACTCGGATCGTGAGGATCGCCGGAATACTTCGTATCAACAGATAAGATACTCGTCAATATTTATTAGATCCGAAATCTGTTTATGCCTTGAAGAGGACTCGAACCTCCACGCTCTTTAGCACGAGATTTTGAGTCTCGCGTGTCTACCATTTCACCATCAAGGCATCTTGAAAGTGAATCGTATTCCATGAATATGATATCTATCTAGTGTGATATATGGAATATATGACAAAGGTGGAGTGTTGGAGTATTTCTATCGATCGGTCATGTCATATAGGCCCGAGTCAGACATCAAATTGCTTCGATTGGAATTATCCGGAGGATACCTTCTATATATCAAACTATATATCAAAAAGATGTAAAATCAAACCTCTTTCTCGATTCAAGAGAAGCCCAAAGAAGTGAATAGGGTACCCAAATAACGATAGATATGTCAAAAGCGGGTCCGATTACGCCTATTCCTAATCCTAAATAGAATGTAACGATGTAGGGATCCATATGTAAACAGAGTATCTATTTACATACGCTCGAATGACCCCTTACTCATAATAAGAATGTACATAACCCTATTCCGGCCTGGTCCGGTCTGGAATGAACTTATAATCATGGAATCGACTCGATTCCATGATTATAAGTTCATAACTCCAGCCCATTCCCATTTTGGGCGGAAAAGTAATTTTTTTATTCCAGTTAGTAAAAGGGATCTTGAACTAAAAAATAGACCTAGAAGCTAAAAGAGGGTATCCTGAGCAATTGCAAGAATTGGGTTCATTGATATTCCTGGTATAGTAGATGCTATCACACATACAGTCATACTCAATTCGATGGAATTGTTTGATCTTAAGGGAGATCTTCTATAATTTCGCACGTGAGGGGTTATTTCTTGGTTTCGCCCAGTCATTAATAACTTGATTATTTTTAGATAATAGTAGATAGAAACAACGCTCGTAAGGAGTCCTATCGAAACCAAGAAATATAGGCCTGCCTGCCATCCACACCAGAATAGATAGAGTTTTCCGAAAAAACCTGCCAGTGGAGGAAGACCCCCTAGGGATAAGAGACATAGGGCTGAAGAGAGAGCCAAAAAAGGATCTTTCGTGTATAATCCTGCATAATCTCGAATGTTATCAGTTCCGGTACGTAGACCAAATGATACAATGCAAGCAAAAGTTCCTAGATTCATGGAGATATAGAACAACATATAAGTTATCATGCTTGCATATCCATCATTTGAGTCTCCAACAATTATTCCAATAATTACATATCCGATTTGACCGATGGACGAATATGCAAGCATACGTTTCATACTTGTTTGAGTAAGAGCAATGAGATTCCCCAATATCATGCTAAGAATAGCTAGGATTTCCAAAAGAAGATGCCATTCGGTTGATGAGAAATAAAAAAGAATATCGAAAATTCGCGTGGCTGAAGCTGAAGCAGCTACTTTCGAAGTAACAGAAAGAAAAGCAACGACTGGAGTGGGAGAGTCAGAGTCGAAAAGAGGATTCCTCACTTCTTTCTCTCATTCAAAACCGTGCATGAGACTTTCACCTCGCACGGCTCCTAAGTGATAAAAGAAAGAAGAGTAGTTCTTCTTTCTTTTTTGATTACCTTCCTCGCGTATGTATAAGACCGAATCCATTCGATTTCTAAAAAGGATTACTAATCCTTAACTTTTCGAGGAATCCTTCATCAGTGGTTGTGAATGACTGACTTTTTCAATCCTTTCGACCTTGGTTCCGTAGGAGCAAGTCAGAAAGATTGAGAAATAGAACCATCTGATTTGATTCGTTCTCCATAGCCATGAGATGATCATCTTAGGGTGATCCTTTTGTCAACGGATGCTCCTATTACACTCGTAGTCTCTGAAGGATGAGAACCAACTATGTAGCATCTACATCGATAATTCAAGTATTGTATACGTCATTAGTCCGATCCTTTGTAGAACTACCCGTAATAACGAACTTGCAAAAGGGATCTGTTTATCATAAAGAGATTCGTTGTTCCTGACCCTGCTTCACCTTAATTGTTATTTGAACAAAAGGATCACAATAAACTTTTGGTCAAAGTGATGTCTTGGTCCGAGTGGGGATAGCATTTCTCTTCTGCATGTCTATGGAGTTTTGAAAAATCCAAACATCTCAGAGATAGATATAGAGGTAGGAATTTGTCGAACGAACCGCACTCCTTCGTATACATCAGGAGTCCATTGATGAAAAGGGGCTGGGGAAAGCTTGAACCCAATTCCTACAGTGATGGATATAAGCGCAATTGAGATTCCTGGGGAGTTATACATTTGTGTATTGATAAGACCATTCACTATTTCTTGAAGCTCGATCTCCCCCCCAGATAAACCATATAGCCAAGATAAACCATGAACCAGAATAGAAGAGCTTGCCCCACCCATGAGTAAATATTTCGTAGTAGCCTCATTAGACCGTACATCTCTCTTGGTATATCCAGACAATAGGTAGGAGCATAAACTGAAACATTCTGGAGCTACAAAGATAGTTATTAAATCGTTAGCACCACATAAAAACATTCCTCCTAGAGTAGCTGTTAATACGAATAACAGAAACTCTGTTATAGCCATTTCTGTACATTCAATGTACTCTACGGATAGAGGAATACATAGAGTTGAACATAGTAAAATAAGAAATTGAAAGATTTCGTTGAAATTGTTCGTTTGGAAATTTCCCGAAAAGCTAATTATAGGTTCTTCTCTCCATCGGAAAAATAGGGCCGTTATGCTCATTACTAAACTTGTTGAAGAGATGAAATAGAACCAAGGTCTATCTTTTTGATCAGAGGTTGAATCGATCATCAGAAGAAGAATTAGGCCAAAAATTAGGATACATTCTGGGAAAATGAAACTTCCATGGAAGAGAAGCAAATGAAACGCTTTCATAAAAATTCTCGTAGAATCGAGAATGAAGTTTTCATTCTGTACATGCCAGATCATGAATTAGTAACTGCATCCAATCTACGAAAAAGTCCCAATTGTTTCGAACTTTCTATTTTTGGAATGGGATATTTAAGGAATCCCCATGAATAGGATCAAACCTTATTCCATGATATTTCCATAAGATTCCTCTTTCTTATTCTTAAGCAAGCCCCCGAGAAGGCTTAGTTGATCCATGATTTATGTTTCATCTTTCTTTTTGTTTGTTTCGAGAAAGATCTCGATCAATTCCGATTCTTTCTTTTTCTATTGATTCTTTTCCGATCGAGATGTATGGATCCATGGATCTATGTGTCTATATAGATCCTGTTCATGGATTAACGAAAATGCGCAAAAGCTCTATTTGCCTCTGCCATTCTATGAGTCTCTTCCTTTTTGCGTATGGCATCGCCACTGCCTTTGGCAGCATCTACTAATTCGGAACTTAATCGGAAAGACATATTTCGACCCGGACGCTTTCGGGATGCCCCTAATAACCAACGAATGGCAAGCGCTTTTCCTTGTGTAGATCCTATTTCGATAGGAACTTGATGAGTCGATCCGCCTACACGTCTTGCTTTTACTGCTATATCAGGAGTTACTCCACGTATTGCTTGACGTAAAACAGATAGTGGATTTGTTTCTGTCTTTTGTTGAATCTTTTTCACGGCTCGATAGATAATTTGATAAGCCAATGATTTTTTTCCGTGTTTCAGAATACGGTTAACCAACATGTTAACTAATCGATTACGATAAATTGGATCGGATTTTGCAGTTTTTTCTTCTGCAGTACCTCGACGTGACATGAGCGTGAAAGAGGTTCAAGAATCAGTTTTATTTTGATAAGGGCTAAAAACAAATCACTTATTTTGGCTTTTTGACCCCATATTGTAGGGTGGATTTAAAAAGATATGAAAAATCTCCCTCCAAGCCGTACATACGACTTTCATCGAATACGGCTTTCCACAGAATTCTATATGTATCTATGAGATCGAGTATGGAATTCTGTTTACTCACTTTCACTTTAAATTGAGTATCCGTTTCCCCCCTTTTCCTGCTAGGATTGGAAATCCTGTATTTTACATATCCATACGATCGAGTCCTTGGGTTTCCGAAATAGTGTAATGGAAAAAGAAGTGCTTCGAATCATTGCTATTGGACTCGGACCTGTTCTGAAAAAGTCGAGGTATTTCGAATTGTTTGTTGACACGGACAAAGGAAGGGAAAACCTCTTAAATTATTCCAATATTGGACCTTGGACATATAGTAGTTCCGAATCGAATCTCTTTAGAAAGAAGATCTTTTGTCTCATGGTAGCCTGCTCCAGTCCCCTTCCGAAACTTTCGTTATTGGGTTAGCCATACACTTCACATGTTTCTAGCGATTAACACGGCATCATCAAATGATACAAGTCTTGGATAAGAATCTACAACGCACTAGAACGCCCTTGTTGGCGATCCTTTACTCCGACAGCATCTAGGGTTCCTCGAACAATGTGATATCTCACACCGGGTAAATCCTTAACCCTTCCTCCTCTTACTAATACTACAGAATGTTCTTGTGAATTATGGCCAATACCAGGTATATAAGCAGTGATTTCAAATCCAGAGGTTAATCGTACTCTGGCAACTTTACGTAAGGCAGAGTTTGGTTTTTTGGGGGTGATAGTGGAAAAGTTGACAGATAAGTCACCCTTACTGTCACTCTACAGAACCGTACATGAGATTTTCACCTCATACGGCTCCTCGTTCAATTCTTTCGAAGTAATTGGATCCTTTTCTTCGTTCGAGAATCTCCTCCCTTCTTCTACTCCGTCCCGAAGAGTAACTCAGACCAATTCCTTCCCGATACCTCCTAAGGAAAATCCCGAATTGGATCCAAAATGGACGGGTTAGCGTGAGCTTATCCATGCGGTTATGCACTCTTCAAATAGGAATCCATTTTCTGAAAGATCCTGGCTTTCGTGCTTTGGTGAGTCGTCCGAGATCCTTTCGATGACCTATGTTGTGTTGAAATCTATATGATCCGATCGATTGCGTAAGGGCCGCGGTAGCAACGGAACCGGGGAAAGTATACAGAAAAGACAGTTCTTTTCTATTATGTATTATTATATATTATTATATTCTAAATTCTATTCTAATTATCTAATTCTTAGAATTCTTTCTAATTAGATTAGTTATATTAGTCTTATAGTCTTAGTTTTAGTATTAGTTAGTGATCCCGGCTCGGCGAGTCCTTTCTTCCGTGATGAACTGTTGGCACCAGTCCTACATTTTGTCTCTGTGGACCGAGGAGAAAGGGGGCTCCGCGGGAAGAGGGTTGTACCATGAGAATGAGAGAAGCAAGGAGGTCAACTGCTTCAAATATACAATATGGATTCTGGCAATGCAATGGAGTTGGACCCTCATGTCGATCCGAATGAATCAGTCTTTCCACGGGGGTCCATCTTTGCCTGCTAGGCAAGAGGATATCAAGTTACAAATTCTGTCTCTGTCTCGGTAGGACATGTATTTCTATTACTATTCAATTCATAAATGAAAATGAAGTAGTTAATGGTGGGGTTACCGTTATCCTTTTTTATTGTATGTGTTCTTAAGAAAAGGGCGTCACATAGGAACTCTGGAATGGAAAAGAAAAGTAGCTCCAGTTCCTTCGGAAATGGTAGTAAGATCCTTGGCGCAAGAAGAAGGGGTGATTCATATCATCTTGACTTGGTTCTGCTTCCGCTCTTTTTTTTTTACAATACCGAGTCGGTTCTTCTCCTACCAGTCTCGAATAGAGCATGCTGAAAAAAATCTTCTTCATGTAAAAACTGTTCGATTTAGATCGGGAAAACGGATTTTAGGAAACCATGTGCTATGGCTCGAATCCGTAGTCAATCCTATTTTCGATAGGAGCAGTTGACAATGGAATCCAAATTTTCCCATTATTTAACTATCCATAATAGAATAGTGCGAAAAGAAGGCCCGGCTCCGGGTTGTTCAGGAATAGTGGCGTTTAGTTTCTCGACCCTTTGCCTTAGGATTAGTTAGTTCTATTTCTCGATGGGACCGGGAAGGGATATAACTCAGCGGTAGAGTGTCACCTTGACGTGGTGGAAGTCATCAGTTCGAGCCTGATTATCCCTAAACCCAATGGGAGTTTTTCTATTTGGACTTGCTCCCCCGCCGTGATCGAACGAGAATGGATAAGAGGCTTGTGGGATTGACGTGATAGGGTAGGGATGGCTATATTGCTGGGAGCGAACTCCAGGCTAATATGAAGCGCATGGATGGATACAAGCCTTCGGCCTTGGAATGAAAGACAATTCCGAATCAGCTTTGTCTACGAACAAGGAAGCTATAAGTAATGCAACTATGAATCTCATGGAGAGTTCGATCCTGGCTCAGGATGAACGCTGGCGGCATGCTTAACACATGCAAGTCGGACGGGAAGTGGTGTTTCCAGTGGCGGACGGGTGAGTAACGCGTAAGAACCTGCCCTTGGGAGGGGAACAACAACTGGAAACGGTTGCTAATACCCCGTAGGCTGAGGAGCAAAAGGAGGAATCCGCCCGAGGAGGGGCTCGCGTCTGATTAGCTAGTTGGTGAGGCAATAGCTTACCAAGGCGATGATCAGTAGCTGGTCCGAGAAGATGATCAGCCACACTGGGACTGAGACACGGCCCAGACTCCTACGGGAGGCAGCAGTGGGGAATTTTCCGCAATGGGCGAAAGCCTGACGGAGCAATGCCGCGTGGAGGTAGAAGGCCCACGGGTCGTGAACTTCTTTTCTCGGAGAAGAAGCAATGACGGTATCTGAGGAATAAGCATCGGCTAACTCTGTGCCAGCAGCCGCGGTAAGACAGAGGATGCAAGCGTTATCCGGAATGATTGGGCGTAAAGCGTCTGTAGGTGGCTTTTCAAGTCCGCCGTCAAATCCCAGGGCTCAACCCTGGACAGGCGGTGGAAACTAACAAGCTGGAGTACGGTAGGGGCAGAGGGAATTTCCGGTGGAGCGGTGAAATGCGTAGAGATCGGAAAGAACACCAACGGCGAAAGCACTCTGCTGGGCCGACACTGACACTGAGAGACGAAAGCTAGGGGAGCAAATGGGATTAGATACCCCAGTAGTCCTAGCCGTAAACGATGGATACTAGGCGCTGTGCGTATCGACCCGTGCAGTGCTGTAGCTAACGCGTTAAGTATCCCGCCTGGGGAGTACGTTCGCAAGAATGAAACTCAAAGGAATTGACGGGGGCCCGCACAAGCGGTGGAGCATGTGGTTTAATTCGATGCAAAGCGAAGAACCTTACCAGGGCTTGACATGCCGTGAATCCTCTTGAAAGAGAGGGGTGCCTTCGGGAACGCGGACACAGGTGGTGCATGGCTGTCGTCAGCTCGTGCCGTAAGGTGTTGGGTTAAGTCCCGCAACGAGCGCAACCCTCGTGTTTAGTTGCCACCATTGAGTTTGGAACCCTGAACAGACTGCCGGTGATAAGCCGGAGGAAGGTGAGGATGACGTCAAGTCATCATGCCCCTTATGCCCTGGGCGACACACGTGCTACAATGGCCGGGACAAAGGGTCGCGATCCCGCGAGGGTGAGCTAACTCCAAAACCCGTCCTCAGTTCGGATTGCAGGCTGCAACTCGCCTGCATGAAGCCGGAATCGCTAGTAATCGCCGGTCAGCCATACGGCGGTGAATTCGTTCCCGGGCCTTGTACACACCGCCCGTCACACTATGGGAGCTGGCCATGCCCGAAGTCGTTACCTTAACCGCAAGGAGGGGGATGCCGAAGGCAGGGCTAGTGACTGGAGTGAAGTCGTAACAAGGTAGCCGTACTGGAAGGTGCGGCTGGATCACCTCCTTTTCAGGGAGAGCTAATGCTTATGCTTGTTGGGTATTTTGGTTTGACACTGCTTGACACCCAAAAAGAAGCGAGCTACATCTGAGCTAAGCTTGGATATGGAAGTCTTCTTTCGTTTCTCGACGGTGAAGTAAGACCAAGCCCATGAGCTTATTATCCTAGGTCGTAACAAGTTGATAGGATCTCTTTTGTACGTCCCCATGTCGCCACACGGGAGGGACATGGGGACGTACAAAAGGAAAGAGAGGGATGGGGTTTCTCTCGCTTTTGGCATAGCAGGCCTCCCCCAGGGAGGCCCACACGACGGGCTATTAGCTCAGTGGTAGAGCGCGCCCCTGATAATTGCGTCGTTGTGCCTGGGCTGTGAGGGCTCTCAGCTACATGGATAGTTCAATGTGCTCATCGGCGCCTGACCCGGAGATGTGGATCATCCAAGGCACATTAGCATGGCGTACTCCTCCTGTTCGAATCGGAGTTTGAAACCAAACTTCTCCTCAGGAGGATAGATGGGGCGATTCAGGTGAGATCCAATGGAGATCGAACTTTCTATTCACTCGTGGGATCCGGGCGGTCCGGGGGGGGGACCCCCACGGCTCCTCTCTTCTCGAGAATCCATACACCCCTTATCAGTGTATGGACAGCTATCTCTCGAGCACAGGTTGAGGTTCGGCCTCAATGGGAAAATGGAGCACCTAACAACGCATCTTCACAGACCAAGAACTACGAGATCGCCCCTTTCATTCTGGGGTGACGGAGGGATCGTACCATTCGAGCCTTTTTTTCATGCCGGAGGTCTGAAGAAAGCAGCAATCAATAGGATTTCCCGAATCCTCCCTTCCCGAAAGGAAGAACGTTAAATTCTTTTTCCTTTCCGCAGGGACCAGGAGATTGGATCTAGCCATAACATAAGAAGAATGCTTGGTATAAATAACTCACTTCTTGGTTTTCGACCCCCTCAGTCACTACGAGCGCTCCCCGATCAGTGCAATGGGATGTATCTATTTATCTATCTCTTGACTCGAAATGGGAGGAGGAGCAGGTTTTAAAAAGGATCTTAGAGTGTCTAGGGTTGGGCCAGGAGGGTCTCTTAACGCTTTCTTTTTTCTTCCCATCGGAGTGATTTCACAAAGACTTGCCATGGTAAGGGAGAAAGGGGAACAAGCACACTTGAAGAGCGCAGTACAATGGAGAGTTGTATGCTGCGTTCGGGAAGGATGAATCGCTCCCGAAAAAGAATCTATTGATTCTCTCCCAATTGGTTGGATCGTAGGTGCGATGATTTACTTCACGGGCGAGGTCTCTGGTTCAAGTCCAGGATGGCCCAGCTGCGCCAGGGAAAAGAATAGAAGAAGCATCTGACTCTTTCATGCATGCTTCACTCGGCTCGGGGGGATATAGCTCAGTTGGTAGAGCTCCGCTCTTGCAATTGGGTCGTTGCGATTACGGGTTGGATGTCTAATTGTCCAGGCGGTAATGATAGTATCTTGTACCTGAACCGGTGGCTCACTTTTTCTAAGGAATGGGGAAGAGGACCGAAACATGCCACTTAAAGACTCTACTGAGACAAAAAGATGGGCTGTCAAGAACGTAGAGGAGGTAGGATGGGCAGTTGGTCAGATCTAGTATGGATCGTACATGGACGATAGTTGGAGTCGGCGGCTCTCCTAGGGTTCCCTCATCTGGGATCCCTAGGAAAGAGGATCAAGTTGGCCCTTGCGAATAGCTTGATGCACTATCTCCCTTCAACCCTTTGAGCGAAATGTGGCAAAAGGAAGGAAAATCCATGGACCGACCCCATCGTCTCCACCCCGTAGAAACTGCGAGATCGCCCCAAGGACGCCTTCGGCATCCAGGGGTCACGGACCGACCATAGACCCTGTTCAATAAGTGGAAGGCATTAGCTGTCCGCTCTCCGGTTGGGCAGTAAGGGTCGGAGAAGGGCAATCACTCGTTCTTAAAACCAGCATTCTGAAGACCAAAGAGTCGGGCGGAAAGGGGGGGATAGCTCTTTGTTCCTGGTTCTCCTGTAGCTGGATTCTCCGGAACCACAAGGATCCTTAGAATGGTATTCCAACTCAGCACCCTTTGAGATTTTGAGAAGAGTTGCTCTTTGGAGAGCACAGTACGATGAAAGTTGTAAGCTGTGTTCGGGGGGGAGTTATTGTCTATCGTTGGCCTCTATGGTAGAATCAGCCGGAGAGGCCTGAGAGGCGGTGGTTTACCCTGTGGCGGATGTCAGCGGTTCGAGTCCGCTTATCTCCAGCCCGTGAACTTAGCTGATACTATGATAGCACCCAATTTTTCCAATTCGGCAGTTCGATCTATGATTTCTCATTCATGGACGTTGATAAGATCCTTCCATTTAGCAGCACCTTAGGATGGCATAGCCTTCACGTGAATGGCGAGGTTCAAACGAGGAAAGGCTTACGGTGGATACCTAGGCACCCAGAGACGAGGAAGGGCGTAGCAAGCGACGAAATGCTTCGGGGAGTTGAAAATAAGCATAGATCCGGAGATTCCCGAATAGGTCAACCTTTCGAACTGCTGCTGAATCCATGGGCAGGCAAGAGACAACCTGGCGAACTGAAACATCTTAGTAGCCAGAGGAAAATAAAGCAAAAGCGATTCCCGTAGTAGCGGCGAGCGAAATGGGAGCAGCCTAAACCGTGAAAACGGGGTTGTGGGAGAGCAATACAAGCGTCATGCTGCTAGGCGAAGCGGTGGAGTGCTGCACCCTAGATGGCGAGAGTCCAGTAGCCGAAAGCATCACTAGCTTACGCTCTGACCCGAGTAGCATGGGGCACGTGGAATCCCGTGTGAATCAGCAAGGACCACCTTGCAAGGCTAAATACTCCTGGGTGACCGATAGCGAAGTAGTACCGTGAGGGAAAGGTGAAAAGAACCCCCATCGGGGAGTGAAATAGAACATGAAACCGTGAGCTCCCAAGCAGTGGGAGGAGAATGTGATCTCTGACCGCGTGCCTGTTGAAGAATGAGCCGGCGACTCATAGGCAGTGGCTTGGTTAAGGGAATCCACCGTAGCCGTAGCGAAAGCGAGTCTTCATAGGGCGATTGTCACTGCTTATGGACCCGAACCTGGGTGATCTATCCATGACCAGGATGAAGCTTGGGTGAAACTAAGTGGAGGTCCGAACCGACTGATGTTGAAGAATCAGCGGATGAGTTGTGGTTAGGGGTGAAATGCCACTCGAACCCAGAGCTAGCTGGTTCTCCCCGAAATGCGTTGAGGCGCAGCAGTTGACTGGACATCTAGGGGTAAAGCACTGTTTCGGTGCGGGCCGCGAGAGCGGTACCAAATCGAGGCAAACTCTGAATACTAGATATGACCCCAAACAGGGGTCAAGGTCGGCCAGTGAGACGATGGGGGATAAGCTTCATCGTCGAGAGGGAAACAGCCCGGATCACCAGCTAAGGCCCCTAAATGACCGCTCAGTGATAAAGGAGGTAGGGGTGCAGAGACAGCCAGGAGGTTTGCCTAGAAGCAGCCACCCTTGAAAGAGTGCGTAATAGCTCACTGATCGAGCGCTCTTGCGCCGAAGATGAACGGGGCTAAGCGATCTGCCGAAGCTGTGGGATGTAAAAATCATCGGTAGGGGAGCGTTCCGCCTTAGAGGGAAGCACCCGCGCAAGCAGGTGTGGACGAAGCGGAAGCGAGAATGTCGGCTTGAGTAACGCAAACATTGGTGAGAATCCAATGCCCCGAAAACCTAAGGGTTCCTCCGCAAGGTTCGTCCACGGAGGGTGAGTCAGGGCCTAAGATCAGGCCGAAAGGCGTAGTCGATGGACAACAGGTGAATATTCCTGTACTACCCCTTGTTGGTCCCGAGGGACGGAGGAGGCTAGGTTAGCCGAAAGATGGTTATCGGTTCAAGGACGCAAGGTGACCTTGCTTTTTCAGGGTAAGAAGGGGTAGAGGAAATGCCTCGAGCCAATGTCCGAGTACCAGGCGCTACGGCGCTGAAGTAACCCATGCCATACTCCCAGGAAAAGCTCGAACGACCTTCAACAAGAGGGTACCTGTACCCGAAACCGACACAGGTGGGTAGGTAGAGAATACCTAGGGGCGCGAGACAACTCTCTCTAAGGAACTCGGCAAAATAGCCCCGTAACTTCGGGAGAAGGGGTGCCTCCTCAAAAAGGGGGTCGCAGTGACCAGGCCCGGGCGACTGTTTACCAAAAACACAGGTCTCCGCAAAGTCGTAAGACCATGTATGGGGGCTGACGCCTGCCCAGTGCCGGAAGGTCAAGGAAGTTGGTGACCTGATGACAGGGGAGCCGGCGACCGAAGCCCCGGTGAACGGCGGCCGTAACTATAACGGTCCTAAGGTAGCGAAATTCCTTGTCGGGTAAGTTCCGACCCGCACGAAAGGCGTAACGATCTGGGCACTGTCTCGGAGAGAGGCTCGGTGAAATAGACATGTCTGTGAAGATGCGGACTACCTGCACCTGGACAGAAAGACCCTATGAAGCTTTACTGTTCCCTGGGATTGGCTTTGGGCCTTTCCTGCGCAGCTTAGGTGGAGGGCGAAGAAGGCCTCCTTGCGGGGGGGCCCGAGCCATCAGTGAGATACCACTCTGGAAGAGCTAGAATTCTAACCTTGTGTCAGAACCTACGGGCCAAGGGACAGTCTCAGGTAGACAGTTTCTATGGGGCGTAGGCCTCCCAAAAGGTAACGGAGGCGCGCAAAGGTTTCCTCGGGCCGGACGGAGATTGGCCCTCGAGTGCAAGGGCATAAGGGAGCTTGACTGCAAGACCCACCCGTCGAGCAGGGACGAAAGTCGGCCTTAGTGATCCGACGGTGCCGAGTGGAAGGGCCGTCGCTCAACGGATAAAAGTTACTCTAGGGATAACAGGCTGATCTTCCCCAAGAGTTCACATCGACGGGAAGGTTTGGCACCTCGATGTCGGCTCTTCGCCACCTGGGGCTGTAGTATGTTCCAAGGGTTGGGCTGTTCGCCCATTAAAGCGGTACGTGAGCTGGGTTCAGAACGTCGTGAGACAGTTCGGTCCATATCCGGTGCGGGCGTTAGAGCATTGAGAGGACCTTTCCCTAGTACGAGAGGACCGGGAAGGACGCACCTCTGGTGTACCAGTTATCGTGCCTACGGTAAACGCTGGGTAGCCAAGTGCGGAGCGGATAACTGCTGAAAGCATCTAAGTAGTAAGCCCACCCCAAGATGAGTGCTCTCCTATTCCGACTTCCCCAGAGCCTCCGGTAGCACAGCCGAGACAGCGGCGGGTTCTCTGCCCCTGCGGGGATGGAGCGACAGAAGTATTGAGAATCCAAGATAAGGTCACGGCGAGACGAGCCGTTTATCATTACGATAGGTGTCAAGTGGAAGTGCAGTGATGTATGCAGCTGAGGCATCCTAACAGACCGAGAGATTTGAACCTTGTTCCTACACGACCTGATCAATTAGATCAGGCACTCGCCATCTATTTTCATTGTTCAACTGTTTGACAACATGAAAAAAAACCCAAAAGCTCTGCCCTCCCTCTCTATCGGATGGAAGGGCAGAGGCCTTTGGCGTCCCTTCCAGTCAAGAATCGGGGCCTCACAATCACTAGACAATATTTATCTCATGCCTTTCTTCGTTCATGGTTCGATATTCTGGTGTCCTAGGCGTAGAGGAACCACACCAATCCATCCCGAACTTGGTGGTTAAACTCTACTGCGGTGACGATACTGTAGGGGGGGTCCTGCGGAAAAATAGCTCGACGCCAGAATGATAAAAAGCTTAACACCTCTTATTTTACTTTTTCATATTTCAAAAGATCAAAATCAAAAATGCAAAGGTCGTCTTATTCAATAACATCCCTTCTCTCCCACTTCACGCCTCGGAACGCACTGTTATTCTAGAGAGAAAGGCGCTTTCACATCTTCTTAACCCAAAATGGCTGAGGAGAGGAAAGGTTCCTTTTTGAAGGTACTCCCGGGAACAGATCCAGTGTAGACGGGGTGGGGCCTGTAGCTCAGAGGATTAGAGCACGTGGCTACGAACCATGGTGTCGGGGGTTCGAATCCCTCCTCGCCCACAACCGGCCCAAAAGGGCCTTTCCCTCTGGGAGTAGTAAAATCATGATCGGGATAGCGGCCACAAAGCTATTGAACTTGGGTATGGTCCTTATAAGTCAGGTTTAGTTTATTACATATAGTAAACAGGGCTGGAATCAGCATATTTGTGTTTGACTCCCCGTAACTCTTCCTCAGCCAGGCTTGGGCAGAATAGCAGAGCAAGTACAAGTATTAGTAGCATAGCAAAAATGCGTTCCTCGTCATTAATATGTTTGCTCGCGGTAATTGTGGCCTATCGGGAGAATTGATGACTGCATCTTTGATGCACTGTTGAGAATTCTGAATTGGCTATTTGCAAGGGTTGGATCTATGCCGAGGTATTGAGGGTAATTCTCAAATATTGTAGAACAGAATGTGATACGATGAGATAGAATGCAATAGAAACAAAGACAGGGAACGAGATTACCTACTCCTAACGGTCAAAGCGAGCCCTTTCATTCAATTCTTCATTCTTTCATGAAGAATGAATCAAATCTCCCCAAGTAGGATTCGAACCTACGACCAGTCAGTTAACAGCCAACCGCTCTACCACTGAGCTACTGAGGAACAACGGGAGATTCGACCTCATAGAGTTCAACTCCCGTTCTCAACCCATGAACAATATGAGTCCGAAGCTTCCTTCGTAACTCCAGGAACTTCTTCGTAGTGGCTCCGTTCCATGCCTCATTTCATAGGGAACCTCAATGTGGCTCTATTTCATTATATTCCATCTATATCCCAATTCCATTCATTTCATATCCCTTTTGTGTCATTGACATAAGAGATGTCATTTATAGTATATCTGTTTCTATCTATATAGATATGGAAAGTTAAGGAATCATCATATAATAATCGATAAATTGCAATAGAAAAGAAAAAGGGGAGGTTTGTGATGATTTTGAAATCTTTTCTACTGGGTAATCCATTATCCTTATGCATGAAGATAATAAATTCGGTCGTTGTGGTCGGGCTCTATTATGGATTTATGACCACATTCTCCATAGGGCCCTCTTATCTCTTCCTTCTCCGAGCTCGGGTTATGGAAGAAGGAACCGAGAAGGAGGTATCAGCAACAACTGGTTTTATTGCGGGACAGCTCATGATGTTCATATCGATCTATTATGCGCCTCTGCATCTAGCATTGGGTAGACCTCATACAATAACTGTCCTAGTTCTACCGTATCTTTTGTTTCATTTCTTCTGGAACAATCATAAAAACTTTTTTGATTATGGATCTACTACCAGAAATTCAATGCGTAATCTCAGCATTCAATGTGTATTCCTGAATAATCTAATTTTTCAATTATTCAACCATTTCATTTTACCAAGTTCCACGTTAGCCAGATTAGTCAACATTTATATGTTTCGATGCAACAACAAGATTTTATTTTTAACAAGTAGTTTTGTTGGTTGGTTAATTGGTCACATTTTATTCATGAAATGGGTTGGATTGGTATTATTCTGGATACGGCAAAATCATTCTATTCGATCTAATAAGTATCTTGTGTCAGAATTGAGAAATTCTATGGCTCGAATCTTTAGCATTCTCTTATTTATCACCTGTGTTTACTATTTAGGCAGAATGCCGTCGCCTATTGTCACTAAGAAACTGAAAGAGAAAGAAACCTCAGAAACGGAAGAAAGCGATGTAGAAACAACTTACGAAATGAAGGAGACTGAACAGGAACAAGAGGGATCCACCGAAGAAAACCTTTGTTCGGAAGAAAAGGAGGGTCTGGACAAAATAGATGAAACGGAAGAGATCCGAGTTAATGGAAAGGAAAAAACAAAGGATGAATTTCACTTGAAAGAGGCACGCTATCAAGATAGCCCAGTTTACGAAGATTCTGATCTGGAGACCCATCAAGAAAATTGGGAATTGGGAAGACTGAAAGAAGAGAAAAAGAAAATAATGAATATGAATAAAAAGATTGACACATAATAAAAATACAAGAATAAATAAGATGAGATTCGTCCACCTCCCATATATTTTATTCCTTCGCCCATAAAGAAACTTGCAATACCAATCCCATTTAGAATTCCATCAATTATATATTTATCAAAAAACTGAGTTAGCTCGGCTAACCCTCTTATACTCATGGTGAAAATCCCAGTATAAAAAATATCTATATAACCACGATTATATGACCAATTGTATATCATACCTTTTATTTTATCCAGAATAATTCTTTTAGGACCTCTTTTGAAAAAGAAATTAATTAAGCCCAAATTTTTGAAAGATGAATAAATAGATCCATAAAAAATAGATGCTATAAATAGTCCGAAAAGAGCTATACTTACTGAAAAAAAAGCATTTGAAAAAAATCCATACCAATCCTCAGAAGAATTAAATTTCTGATGAAAAAGGGTTGTCGATGGAGTTAACCATTTCGATAATAGATCCAAATCTATTACTCCTCCGTTAAAAGAAATTCCTATTGATCCAATGAACAAAGTGAATAATACTAATATAAGGAGAGGAAATAACATAGTATTGCTCGATTCGTGAGGATACATATAAGTGTATCTATTTATAAAGTAAGTACTAAAGTCTCGTATCTTACTTCTTACATTCTCGTCAATTTTAGATATATTTTTTGAAAAAAAACAAACCTTATTCTTATTCATTTTTGAAAAAAATAAATTACTATTGACTTTTTTAAGTGTCCCTTTTCCCCATAGAGATATTGAATAAAACGAGCTCTTTTTTGTACTACTAAGACTACTATAATCTTGAAAATGAATGCGCAAATACCCATCAAAGGTAAGTAAATACATCCTAAACATATAAAATGCGGTTAATCCTGTTGTGAACCAAGCTATTAGTGCGAAAATTGGTGAGTACAACCAACTATCGTGAAGAATTTCATCTTTGGACCAAAAACAAGCAAGAGGCGGAATACCACAAAGAGAAAGTGTACCTAAAAAAAAAGTAATTTTTGTAATTGGAACATATTTTGTTAAACCTCCCATAAGAACCATATTCTGACTTTTCTCTGGTGAATATCCAACAATAGGTTCCATTGAATGAATAATAGATCCGGATCCCAAAAATAATAAAGCTTTAGAATAGGCATGGGTGATCAAATGAAATAAAGCAGCTCGATAAGAACCTATGCCTAGAGCTAACATAATATAACCCAATTGAGACATTGTAGAATAAGCTAAACTTCTTTTAATGTCTCTTTGGGCAAGAGCTAAAGTAGCTCCTAAAAGTACTGTTATTATACCTACTAAACAAATGAGATTCATTATGTAAGGTATAACTATGAAAAGAGGAAGAAGCCGAGCTACAAGAAAAATCCCTGCTGCTACCATAGTAGCAGCGTGTATAAGAGCCGAAATAGGAGTGGGGCCTTCCATGGCATCAGGTAACCATACGTGAAGGGGGAATTGTGCGGATTTAGCAACTGCACCGACAAATAATAAAAAGGCGCATAAAGTAGCAAATAAAGAATTGACCCCATTATTATAGATCAAGGTATTCACTATTTGGAACAAATCCCGAAATTCGAAACTACCAGTTATCCAATAAAATCCTAAGGTCCCTAATAATAAACCAAAATCTCCTATACGATTAGTTACAAAAGCTTTTTGACAAGCACTTGCTGCAACGGGTCGTGTGAACCAAAAACCTATCAATAAATACGAACACATTCCCACTAGTTCCCAAAAAATATAAATTTGTATCAAATTGGAACTAGTAACTAATCCCAACATTGAAGCATTGGAAAAACTCATATGAGCAAAAAATCTCAAATATCCTTGGTCATGAGACATATAATTGTCACTATAAATAAAAACCAGGATTCCAACAGTAGTAATTAGTATTGACATAATAGAAGTAAGTGGATCGATCAAGTGTCCGAACTCTAATAAAAAATCATTATTGATGGTCCAAGACCATAGATATTGATAGGTCAAACTTCCATTTATTTGCTGAATAGACAGATTAGCTGAAAACCACATAGCTATACTTAGTAGTAAAACACTTAGGAAAGCCCACATACGACGAAGATCTTTTGTTGTTGTCGGAATAAGTAGAAGTCCAAATCCTATTGACATAGTAACTGGGAGCGGAAAAAGGGGTATTATCCATGCATATTTATATTCCATAAGAAACCAAATTGTTCTTTTTTCTTATAATTGTTTCCAATTCACCAATTCTGATCTCTTTCGAAAAGAACAAAACAATAAGAAAAAAATATGAAAAAGATCAAATACAAAAATACAAATATTGGAATTCTGACTTTTTGTTTTATTAAATATTTGAAATAAAAGTTGCAATAGGTTGATCATATATCAAATAATATGACCAAATAATTAGTCAAGTTTATTACTTAGTAATTAATTAACTTAAAACTCTAGAAAATCTAGAAAAGAAAGATATTTTTGAAATAATATGACATCATATGAGATTTTGAATAATTGGATTTTCCCTTTACATTATATTCTAATTATGTAAAATTATGTAATTTATAGATATATGATATATTTTTATATTTCTATTAAAGTTCAGTTACAGATTTCTTTATCTCTTTCTATTTTTATATTTTTCTTTCTTTTTTTTATTGTATAATCTTTATATAGAATCTTTTCTTTTATATACTCTATTTTTATATACTATATATTATATACTATATACTATATATTTACTATATATTTTATATTTTTTTTATATACTATTTACTATTTAGATAAATTAGATAAATATTTTTTATTACTATTCTTATTAATATTAAATATGAATATTTGCAATATCGTATATATCGTATATATATATACGATATATGACTATAATGTTTTAATAATAATATATTATATTAATAATAATAGAAATTAAATAATATGAACTAGTAAAATTCAATTACTTTTTTTGTATATTTTTTTTGTATATATTTGTATATATTCTTTTATAAAACAATAAATATAAAATACGTATGCAATTATTACATTATGCAAATATCAGAATGAAGATAGACAATTGAAATCTATTTGTCTATGAAAATAGAATCATTTTAGAATATTTTTATTTTCTTATTTCTTTTCTTTTATTCTTTTTTTTTTATTTGTATGTTATGATATTATTGAGGAAATTTTGAAATTTCTGGAATTAAGTATAGATAATGACTAAGAAAAAGTAATTTATTTTAAACAAAATATGTCTTTCACATACAACGATAAAAAGGAGTCACCTACCTTTTGAATGGCAGTTCCAAAAAAACGTACTTCTATGTCAAAAAAGCATATTCGTAGAAATCTTTGGAAAAAAAAAGTATCTTTAGAGGCAGTAAAAGCTTTTTCTTTAGCTAAATCTATTTCCACCGGACAGTCAAAAAGTTTTTTTGTGCGACAAAAAAAAGTCTTGGAAAAATATTAATTGACATGTTTCAAAGAACTTCCAAATTTACATTTTTGAATTGGAAGACAAACGATTCAATTTTACTAATGTATTGTATTTGTATTTCACTTCTCCTTATTAGTTAGAGCTAGGTAATATAAAAAATAAGAATCTTTCTTTCTACTTGATTCAAAGTACTCAGTATTGTGTATTTTATTTTTTTTATCATTTTTTTTTCTATTTTTTTGAGATGATTTTTTCTTTGTCTATCGTAATTGTGCTTTTAAAAATAGAAAAGCACAATTACGATAGACAAAGAAAAATATGAATATTTCATTATACTTTATACTAAGGTGTTGGGTCTAAAAATCGTTATTAGAAAAAAAATTATGAATTTTATACCCCGACTGAGAACGAAGCTTTTGAGTTATGACTGTTCTGGATAAACAAGAGCTAGATTTCTAGTACGGAAAAGTTGATTATTAAAACTGAACTTACGAAGATGAAGATACTAATTAAGTATTATTGATATTGAACATTTCCATATGAATGTAAATGCATCTTTATTCATTGTTTCCTAATTTATATTGAATATAGACAATTCAACATGAATTTCCTATTATTATTTAAGGTAAGCCGCCATGGTGAAATTGGTAGACACGCTGCTCTTAGGAAGCAGTGCTAGAGCATCTCGGTTCGAGTCCGAGTGGCGGCATAAATAATTATTAATATTAATAATATAGACACAATAGATCTAATAGAATCTAAGATATTTAAAATATTAGATTTTATTTAATCCTCTCCCCAATTTCAATTATTTAAAATTATTTAAAAGGGACTCTTCTTTATGATATTTGCAACCTTAGAACATATATTAACTCATATTTCCTTTTCGATCATATCAATTGTGATTCTAATTCATTTGATGAACTTATTAGTTGACGAAATTGAAGGATTACGCAATTCGTCAGAAAAAGGGATGATAGCTACTTTTTTCTCTATAACAGGGTTTTTAGTTATTCGTTGGATTTCTTCGGAACATTTTCCCTTAAGTAATTTATACGAATCATTAATCTTCCTTTCATGGAGTTTATCCATTATTCATATGATTCCGTATCTTGGGAATCATAAAAATGATTTAAGCGCAATAACTGCACCAAGTGCCATTTTTACCCAAGGTTTCGCCACGTCAGGTCTTTCAAATGAAATGCATCAACCCGCAATATTAGTACCTGCTCTACAATCTCAGTGGTTAATGATGCATGTCAGTATGATGCTATTGAGCTATGCAGCTCTTTTATGCGGATCGTTATTATCAATTGCTCTTATAGTGATTACATTTCAAAAAAAAATCGATTTTTTCAAGAATTTTTTAAGTTTAAGGAAGTCGTTTTTCTTTGGTAATATGGAATATTTGAACGAAAAAGGAAGTATATTAAAAAAGACTTTTTTCCTCTCTGTTCAAAATTTTTACAAATATCAATTAATTCAGCGTTTAGATTATTGGAGTTATCGTGTCATTAGTTTAGGGTTTACCTTTTTAACCATAGGCATTCTTTCTGGAGCAGTATGGGCTAATGAAGCATGGGGTTCTTATTGGAATTGGGACCCTAAGGAAACTTGGGCATTTATTACTTGGACCATATTTGCAATTTATTTACATACTAGAACAAATTCAAAATTGCAAGATCAAGGCACGAATTCGGCATTTGTAGCTTCTATAGGATTTCTTCTAATTTGGATATGCTATTTTGGGATCAATCTATTAGGAATCGGGTTCCATAGTTATGGTTCATTCCAATTAATATCTAATTGAATAAAATAAACTACATGAAGAATACATAAAAAAATCGTCTGATACACAATTAAATTTTGTGCGAGTTTTTGAGAACCGTTTAAATAGAGGAATTATTCAAAAGGTTCTCAAAAACTTTAGATGTATTTCATTACAATTCTAATTAATCTTTCCCTTTTTTTTTCATTGTACAACGAAGAATCGTGAAAATATGAAAGTCAAAGAATTCTAAGACTTTCTTTCCAATTAATGAATTTGATTTCATTTATTATTGAATCTAAAAAAATAATTAGTATCTATAAAAATAATTAGATACTAGAACTTGTACCTTGTCAACCGATAACGGGAGAACGAAATCAGGATAAATACCAATTCCTATTACAGGTAAAAAGATACATATCGAAACAAAAAGCTCTCGTGGTCCAGAATCAAAAAAATTCGAGTTTGGAATATTGAATAGCTTGTATCCATAGAAAATCTGACGTAACATAGATAATAAAAAAATAGGAGTTATTATCATTCCAATTGCCATTACAAAAGTAATTAACATTTTTGGCATGAAAAGATATTTTGGGCTGGTAATTATTCCAAAAAAGACTAAGAATTCTGCAACAAAACCACTCATTCCTGGCAATGCAAGAGAAGCCATCGAGAAACTACTAAACATGGTAAATATTTTTGGCATTGGGATAGATATCCCCCCCATCTCTTCGAGATAAACAAAACGTATTCTATCACAACTTGTTCCTGCTAAGAAAAAAAGTGCAGCACCAATCAATCCATGAGATATTATTTGTAAAATGGCTCCATTAAGTCCCATGCCAGTTATAGAACCAATTCCTATAATTATGAAACCCATGTGAGATACGGAAGAATAGGCAATACGTTTTTTTAAATTGCGTTGACTGAGAGAGATTGAAGCTGCATAAATGATTTGTATTATTCCTACTATCACCAACCAGGGAGATAATTTAGAATGAGCGTGAGCTAATAATTCCATATTGATCCGAATCAATCCATATGCTCCCATCTTTAATAAGATTCCAGCTAAAAGCATACATGTACTGTAATGTGCTTCCCCGTGGGTATCAGGTAACCATGTATGTAGGGGTATCATCGGCGATTTGACAGCATAAGCAATAAGAAAACCAAAATAGAGTATTATTTCCAATGCTGCAGGATACGATTGATTAGCTAATTTTTCTAAATCTAATGTTGGTTCATTGGAACCATATAAACCCATACCTAGAACTCCTATTAAGAGAAAAATGGAACCTCCGGCAGTGCACAAAATAAACTTTGTAGCCGAGTACAAGCGTTTTTTTCCTCCCCACATGGATAAAAGTAAGTAAACAGGAATTAATTCTAATTCCCACATGATGAAAAAAAGTAAAAGGTCTCGAGAAGAAAATGATCCTATTTGGCCACTATACATTGCTAACATCAAGAAATAGAAAAATCGCGGATTTCGAGTAACTGGCCAAGCTGCTAAAGTAGCTAAAGTAGTGATAAATCCTGTCAGTAAAATGGGTCCTATGGAAAGTCCATCGGTTCCCAGTCTCCAGTGAAAATCAAAAAGATTGATCCAATTAAAATCCTCCTTCAATTGGGTTAATGGATCGTCCAATTGAAAATGATAACAAAATACATAGGTCATTAGAAGGAGCTCTAGTATACATATACATAGAGTATACCACCTATACGCCTTATTTCCCCTATGAGGGAAAAAGAAAATTGAAGAACCCGCAAATATGGGCAAAACAAGAAGTATTGTTAACCAAGGAAAATAACTCGTGATAAAGACAAGATAAAATTAGACCAGAAAACCCCGTGCTCGGGAGAAGAATAATATATTTTCTTTTCTCGAGTACGGGCTTTTGTCGGTAAAGAGGAATCAAATGATTCAAGTGGAGTTTTTTGTCACATATCAATAAGAAAGACCCATGCTGCGAGTTGTTTCATGCCATAAATAAACACGGACACTCAAAAAATCCGTTGGACAAGCGGATTCACATCTTTTACAACCTACACAATCTTCGGTTCTTGGCGCAGAAGCGATTTGCTTAGCTTTACATCCGTCCCAAGGTATCATTTCCAATACATCCGTGGGGCAAGCTCGAACACATTGGGTACATCCTATACATGTATCATAAATCTTTACTGAATGTGACATTGGATCTATAAATTCCAGTTTTGAGCACAAAAGATTTTCGATCTGGTAAAAGAAAATAAGAAAATTAAATAAATCATATATTTTATATTGTAGACACCAGACGAATCAATGATTTATCAAAATTTGAAGAATCAATAGATTTTCTAATCTGTTTATGAGAAAGAGCCAAGATACTTTGATTTTCATTTCAATAACCATGAAATATGAGTTTACGAATTCAATTCATGTTAAATTTACTATCTTTCTATATGTATATATTCATATACATATAGAAAGATAAATATAGAAAAATATAGAAAGATTTTAGTATATAGAAATAGAATTAAGGTGATATATTATATATCAGATGAATACGTTTGTTATTAGGTTAGTGATAGAATAGGTTAGTAACTATAAATCATAAATTTATATGAAAAAAGAGAAGAAAGAAAATAAATAATAAATAAGAAAATAATAATAAGAAAATATTATATTCTATTTAATTTTAATTAAATTATCTTACTATTCTAATTCTATTAGATTCTATATTAGAATATTCAGAATATTCTAAAAGTCTTATGTTTTGATTTATATGTGAAAATAGAATAATTATGACTAATTATTCAGCAAATTTGATTGATTGATACGAGTTGATTTTCTGTTACGATGGATCGACGAAACAATAGCTAGTCCAATAGCTGCTTCAGCAGCCGCAATGGCTATAACAAAGATTGAGAAAATTTCTCCTTTTAATTGCCGACTATCAAATATATCGGAAAATGCGACGAGATTTATATTCACCGAATTCAGTATAAGCTCAAGACACATAAGTGCTCTAACCATGCTTCGGCTTGTGATCAGTCCATAGATACCGATAGAAAATAAATAAACACTTAGAAAAAGTACATGTTCTAACATCATTGATAAAGTCCTCATCTATCTCGATTCATTTCAATATGAACGAACAAAAATTAAACCGATTCAGTTGACTAGAATAGAAGAATGACAGAACAAAAGAATATATTCACAGTAGATTTCAATAAAATAGATTAAATCCATATTTCATTTTTTAATAAAAAAAAAGAAGTTCTTATTATATTAGATTATTGACGAGCCATAGTAATTGCACCTATCAAAGAAACTAAAAGAATTATAGAAATGAGTTCAAAAGGAAGATAAAAATCTGTGGATAAATGAATCCCAATTTGTTGAACGTTACTTATTAAGTCCTGTTCTATAATCTGATTTGATCTTGTAGTCCGAAAAATTCCGGACCATGACGTATCTGGGATAGTAGTCATTAATGAAAAAAAAATACTTGTACAAACCAGTGAAGTGATCCTATCTCCAACGGTCCACAAATAGGAATCATTGGAATATTCTGAACCGTTCATGAACATCACAGCAAATATGATTAATACATTTATGGCTCCCACATAAATAAGGAACTGCGCGGCAGCTACAAAATAGGAATTCAATAAAATATAGAATAAGGATATACAAACAAGAACCAATCCCAATGAAAAGGCAGAATCGATGGGATTGGTAAGTAATACTACTCCCAGACCTCCTAATATAAGAACTGATCCCAGAAATACTACAAGAATATCATGCATTGATCCAGGTAAATCCATTATGAAATAAAAAATAAATAAATAAGTCGAAATATTTCATGACCTTACTAAGGGTCCAGGAAAGGAACTATTTTTTTATATGATACCTTCCTAATTGAATGAAAAAAAAATGAATATCATTAGATAGATAAAATAAAGTTATTTGGCTAATCCTACTTACTTTATTCCAAGCCAAATCTTAGTAATTGGTAATCGTTCTTGAACCAAGCAAGGGGTTTTTATTTTTTAATTTGATTTGTTGAATCCATAACTGTTTGAATTGTGTAATCTCCAATTATTGAGATTGGTAACCGACCTAAAGAAATTTGATTATAATTCAATTCGTGACGATCATAAGTGGAAAGCTCATATTCTTCAGTCATCGATAAACAGTTTGTTGGACAATACTCGACACAGTTACCGCAAAATATACAGACACCAAAATCAATACTATAATGAAGCAATTGTTTTTTCTTAATATATTTTTCAAATTTCCAATCAACAATGGGAAGGTCTATTGGACATACGCGAACACATACTTCACAAGCAATACATTTATCAAATTCAAAGTGGATTCGACCACGAAAACGCTCTGATGTGATTGATTTTTCATAAGGATATTGAATAGTTACAGGTAAACGATTTGTATGGGATAAGGTAATTATGAAACTTTGTCCAATGTACCTTGCGGCTCGTATTGTTTGTTTGCCATAATTCATGAACCCAGTAACCATAGGTAACATATTTTAGATATCCATGAATAAAATTTATGTTTCTTTCTCTTGGTTGAGATAAGTTATGAATATAGAATATTCATTATTGTTTTCTCTTAATTTCTTATTTTTATTTATAGTTTATAGTGAAACAAGTTGAAAAGAAGTTGTCAATAATAGATTACCTAGAGAAATAGGTAAAAGAAATTTCCATCCAAGATTTAATAATTGATCCATTCTCATCCTAGGTAAAGTCCATCTTGTTGTGATAGGAATGAACAGAAACAAATAAGCTTTAGCTAATGTAATAAAGATACTAATTGCTATTACAAAGACTCTAAACATTTTATTTATTCCGAAAAGTTCAGTAAGAGATATGTACGGAATAGAAAAATCCCACCCACCTAAGTAAAGAACTGTTACAAATAATGACGAAACTAATAGATTTAGGTAAGAAGCAAGATAAAAGAACCCGTATTTTATACCTGAATATTCGGTTTGATAACCTGCTACTAATTCCTCCTCTGCTTCTGGTAAATCAAAAGGTAATCTTTCACATTCTGCTAGAGAAGACATTATAAAAACTAGAAACCCTATGGGCTGACGCCACAGATTCCATCCCCCAAAACCATATTTGGACTGTGCCTCAATTATATCAACTGTACTTGAACTGTTAGATAATTATAGTCGATGATAGCATCACTGCTCCCATCGCTATTCCAAAACCGTACATGAAACCTAAGCTTCATACGGCTCCTCTATGGCCACAAAGAAATGTAAGGTAAGGACTAGTTTAGCATTATAGCTCTCCTTGGACCTTAGATAAATACAATGTCAAGAGAATTTGGAGGTTGGAGAGTCCTCAATTCGACCAATAACATTCTGTCTGTTAGAATAAGAAAAAGCACTTCCGAATTGATCTCATCCCTTATAATGATATTATAATGAAAATAATCAAAATTTATCTTTGTTCAGCAATAACTTAATCCTTCAATCAAATACTGGTTCTATTCATAAATAGAAAGAATTGGGCATTAGTTAATTAATCATGATAAGAATTCCCATATGCCATATGCATATGTATGAAGAAAGAAATATTTTCTTATTATTTCCGTTTTATTCTTTTTTATTATATTATTGTATTGCATTCTCATTCTATTTGTCTTGTTCCTGTTCTTCTTTCTGAAAACTAAAAAAAAGGAAAGAAAATAAAGGATTAATTCGTTCTTGATAGTCATTTATTTAATCAGCAAATAGTAGTATACTCTAGATCGGAATCGTGGGGAAGTACTGCTTGATCATTTCTACCAACTTCAAGCCCTTATTATGATTCGTTTTATGCAAAGTTTTATGCAAAAATCCCTTTTTTTAATACCTTACATTATTTCCATTACTCATCCTTTGCGTACTTTGGTGTTCCTAACTGCCCACTTCTTTTTAATTGATCCCCAGTATAGTTAGAAATACAGTTGATCCTTTGCATCCGCTTCAAGATATGACGACTAAGAAAATAATCTCAATCTTGGGGTAAACAACTTATGTTTACTTCAATTTTATTCTTGTACCTAGGGAATGAGATTTTTATTGTTTTACTGCAAATTGAGGAGCAGTTTTGTTTCACTCATATAACTATCTGGTTTAACTCATCGAACTGAAATGTTAAAAAAAAAAATATTTTTTTTATTCTTTTATTTCATATTCCTATTGAAATATTGAATTATATGAATTCTATATTCTATTTTATTGTATTTAAATTCATTTTTTATCTCTTTTCTAGAAAAGAGATAAAAAAATTCATGTTCCAACGAATCACACGTAGAGATATTGCTAACACACATAGAGTTAATGGTATTTCATAACTAATCGATTGAGCTGTAGCTCGTAGACCACCTGAAAAGGAATACTTATTATTTGATCCATATCCTGACATAAGAAGACCAATGGGGACAATACTTGAAAAGGCAATCCATAAAAAAACACCTATACTGAGATCTGCTAAAACAAGGTGATATCCAAAAGGAATTACTAAATAACTTAGTAGAATTGATATAAAACCTATAGAAGGTCCGACCCTAAATAAACGAATATTACCTCTAGATGGAAGAAGATCCTCCTTCAAAAATAGTTTGGTCCCATCCGCTAAAGCTTGAAGAATTCCTAAAGGGCCGGCATATTCAGGTCCAATACGTTGTTGTATTGCTGCAGATATTTTTCTTTCTAACCAAACAATGACTAAGACTCCCATTGTGATTCCTAAGACAAGGGTTAAAATGGGGACAAAAATCCATATTAATCCATAGACTTCTTTTAAGGATTCTAATCTATAAAAAGAATTAAGAGTTTGTACTTCTGTCGTATCAATTATCATTTCAACGATCAACTTCTCCCATAATGATATCTATACTACCTAGTATCGTCATGATATCAGCCAATTTCATTCTTTTAACTAGCTGGGGAAGAATTTGCAAATTGATGAAACCGGGTGGACGAATTTTCCATCTCCAGGGGAAAACACTATTATCTCCTATTAAATAAATTCCTAATTCTCCTTTTGGGGCCTCTACCCTTACATAAAGTTCTTGTTTTAACAATTCAAAATTGGGTGAAAGTTTTTTAGTAATAAATCTATATTCAAAATTATTCCATTCGGAATTCTTTGTCCTATGAAAACGCCGGTTTTCTAAATTCTCATAAGGTCCTCCAGGAATTCCTTCTAGAGCCTGTTGAATGATTTTTATGGATTCTTGCATTTCACCGATTCTTACTAAATAACGAGCTAATGTATCGCCTTCTTTTTGCCATTTGACTTCCCAATCAAATTTATTGTAACACTCATAGCGATCAACTTTACGAAGATCCCATTGGATTCCAGAAGCTCGTAACATTGGTCCTGATAAACCCCAATTTATTGTCTCCTCCCCACCAATAATGCCCACTCCTTCAACTCGTTCCAAAAAAATGGGATTTCGCGTAATGAGTTTTTGATACTCAACAACTTCTGTTAAAAAATAATCACAGAAATCAAAACATTTATCTATCCAGCCATAAGGTAAATCCACAGCTACTCCTCCGATACGGAAATAATTATGCATCATCCGCATACCTGTGGCGGCTTCAAATAGATCATATATTAATTCCCTCTCTCTTAAAATATAGAAAAAGGGAGTCTGTGCACCGATATCGGCCATAAAAGGGCCAAGCCATAATAAATGGGAGGCTATACGGCTCAGCTCCAGCATAATAACTCTGATATAACTGGCCCTTTTAGGCACTTGAACACTTTCCAATCGTTCTGGTGCATTTACTGTTATTGCTTCTGTGAACATAGTAGCTAAATAATCCCAACGTGTTACATAAGGTAAATATTGTATAATTGTTCGGTTCTCCGCTATTTTTTCCATCCCTCTGTGTAAATAGCCTAATATAGGTTCACAGTCAATAACATCTTCACCATCCAGAGTAACGATCAGCCGAAGAACACCATGCATTGATGGGTGGTGAGGGCCCATATTAACTATTATAAAATTTTTTCTTGTAACCGGTACAGTCATATTTTTTTCCTTAATTCATTATTTCATGAATTTCTTAAAATGTAAAATATAAAAAAAATAATAACTGAACTAATAAAAAAATAATTAAAAAAGAACAAGGATAATAATAAGAAAATAATAAGAAACTCAAAGAAGAAATTAAAAATTAATTAACGAGTTTTTGGTTCCCGAATATCTAACTGATCCATTAATTTCTTATAACGCACTTTATTTTTCTTTGACAAATAAGTCAATAATCGTTGACGTTTTCCCAGAATTATTCGTAGACCTCTTTGCGATAAAAAATCTCTTTTGTGCAATTCTAAATGTGAAGTAAGTCTCCGTATCTTACTGGTGAAATGGAATACTTGAAATTCAACAGACCCACTATTTTCTTCTTTTTCTTCTTGTGTAATAACTGAGATGAATGAATTTTTGACCATAAATTTAAATTTCTATCTTTCTTTTCTGTGAATTTTACCAATCAGGAAAAATAATAATATTTATTATGCCAGTTATTTTCATCTAGTATACATCAAAATTGGATTTCATTCATATACTACTTTGGTTTTTTATTTATGTGGTTTATGTTTTGTATATTTGATCCAAATATACAAAACATATATGTATTCACGAAAGAGAACACTTTCTTTTTTTTACTTAAAAGGATCCCGCTCTTACTAGTGAAAATTGATACACTATGAAATCAGCATCATGTATTAGAATATTACACAGTGTATATGTTTCGGCTTTCATCTACCGAATATGTTACACGATATGTAGGAAATCCACTATAAATTTTTTTCATTTTTAATTGGAATTGAATTCATTCTGAATTGTGAATACATATGTATTCTTGACATACTGAAACGACTGCTGTTATTGGTATCAAACCAATAGCGATTCATACAAGCTACATCTTCTAATCGATAATTGGGCCAAAGAAAAAATTTTAATTTAATGAAATTTTTTCTATCTGTATTAATATGTTTGTCCTCATTCAAAAATCGACCACAGTTTCTTATTTTGTTTTCATTGCAAAATCTTGGATTTCTATCCACAACATTAAAATTCTGGGAATTGAAACAAATTCGAATTCGAAATTCTCTACGACGTCTGGGAGATAGAATATTTTCAGGAACAAGTAAATCATAATGATTTTTGTCTCTGTCTCCACTCAAAAATATGTTGCTGTGTCGTGCAATGGGTTTTTCAAACCCCCTTTTCTCAATATATCTTTTTTTTGTGTATTTTTTATTTGTTTGGTGCTTCTTATTATCAACCAATGAAATATCCATAGTTTGATATATAATAGATTTTTCGTCCCTTCGTATAGATAGACAAATTGGTTCGATAAGAAATATTCCCTTTCTTATTAATTCTGCAAGAACTAGGTCCTTTTGAATCAGCATTTCGTCTAGATCTATTTCACCTCTTTGAATCGAAGATATAGCAATTTCCTTTGGATTTATCAGTCTAAGTAGGAAACAATATACCCTGATATTTTTCATTATTTTATTATTTAAGGGATCAGCCCATCTTAGTTGAAAAAGTAAATATTTTTTCAAAAAGAAATCTAGTTCCATTTCATTCTTGTTCTTATATTGATATTGTTTTTTTTTTATTTCTAATCTTGCGTAATCTTCTTCAACATCTTTTTTATTTTTTTGTTTTAGTAGATTCCATACAAGATTTATTTGGACCTGTTGTTCGTTTTCTTCCTGCTTGAAATTTCCTAATTCAAGATCTTTTTTTTTATTAGATGATTTTTTTGGATTTACGTTAATGTTTTTACTTTTTTCATTTCTAGAAAAATGAAAAAAGAGTGATTTGATTGGGATGATCCAAGGTTGAATTTTATATACATCAAAAAGTAGCACCAATTCTGGGAAGAACCAATTCTCTAGATTGGATATAGTATCATGATTTGATGCGGTATCATATAACCTTTCTTTATTCATTCCCATGCAATCAAAAAAGTTTCTTTTTTGATTGCATGGTTTGATCTCTTGATAAATTGTAGGATAAAAAAGATCTTTTTTTTCCATTTTTTTTAAATTCTTAATTTCAGTCTTAGTATTTTTATGAATCTTGAAGCCAATATGTATATCGGTCCAGATATCAATATTATTTCTAAAACAAAGATGAAGAATTCTACAATCAAAATATTTTCTATCCAAATTTCTATTCCTATCATTTGTATTCCTATCAATAAGATATCCTTTTTCTAGATAATCATTACTAGGTATACTTATTAATACATAAAATGATTCAGGTTTCGATGTATTGAAATGATATGTAATTTCTTGGTCCCCATTTCTTTCTAATGTTGATTCATAAATGTATAAATTAGGGAGCCATATGTATTTATATGATAAAAGATCATATCTGTAATGTTTTTTCCATTTGTCTTTTTGACTCATAAATAAAATTGCTGCATAGTCATTTTTATTCTTTTTATTCATGGAATAAATAAATTGGTATTTTTTATATAAATCCAATTGGTTTAATTCCTTATTTTGAATCATACAATGTTTATTTATTCTATTTCGTAATTCTTTAGGTACTAATTGAGACCTTTTTTTTTTATATAAATCGTATTGATAATGACCTTTTAACCAGTTTTTCCATTCGTTCATTCCATACGTATGAATTTTATTATGTCTTGATTTGGAATTAAATATTCCATGTATCATACAATAGTCTTTTAAATTATCCTTAAAAAAAGGATAGCTCCCTTCATATTGAAGTACAGGTCTCAAATGATACTTATTAAGTAATTTGTTTTGTGATATTTTGTAAAAAACATATGCTTGGGACAGGGAAGAGAAGTTCCAATAAGTATTGGAATTTTGATTGATATTCTTAGTATTATCAGTATTTGAAAACAATTTTTTTATAGTCAAAATAAAATTCATTGTATTTTGATTTGTTTCATCAATTCTTTCTTGTTCTTGATTTATTTCATTGTTGTAAATGGATTTATTAAAGATCTTTTTTGTTGATTCAAAAAAAAGTTGTGCATTGGTCTTAGAAACGGTAATGGTACATAGCAAAATATCTATGTATATTTTTTCAATGAATGATTTTATAAAGTAGTGTGATTTACGCATTAATCGGATATTTTTCCTTTTTAATATTTTCCAAATATGTTTCTGTGATCCCGATCTTTTATTATCATAAATTAGTTCTTTTTTTTTCTTGTCTTTTGCGGTTCGTTCAATTTGATTCCTTATTTTGATTGTCTTATCAGAAAGATCTTTCATTCTTCTTTCTATTAGTGAATGATTTAACCAATTCATCGTTCGATTTAGAACGGACGATTCACGAAGAATCTTATTATTTCTTTTTAAATCTTTTTTGTTTTCGTTTGGTTCATATACTTTTTCTTTCCTTAATTCAAATAATAAATTTGGATTTACTTTCTCCAGTTTTTTCATTATTAGCTTGATAACTCGAACTATTTCGATGACTCCTTTTGTTTTTTCTTTTCTAACTTTTAGAAAGGTTTTTCTTTTTTTATTTAAATATTTTAGAACTTGTAAAAATTCCTTTTTCACCTTTTTTTTTATTTTTTGGAGTTCTTTATAAATAGGTTCAAAAAAAAAAGGTCGTTTTCGGGGAGAACCAAAGGGAAGTTCCGCTTCCATTCCCCAGACTGTTAAAAAACAAAAATTTGTTTTTTTCTCTTTTTTTTTCATTAGATCTCTATGATGAGATCGTGCCTTAGATTTTCTCCAAGGTTTTAGACAGAAAGGATATAGAATCTTTATCTGAATACCATCTATTAACCAATCTTGGGGAAACTCTTTTTCTGATAATTGAACACCATTATAGGTGCATTTAATATGCATTTCTCTATTCCATTCCTTAAAATCCTCGTCCCACTCGGGAACTTGGAATAATAACATACGGAAAATATTTTTGGCTATTATCAATGAAGGTAATATAATGTTTTTTCTAAGAAAAGATTGGGTTACTAATATCAAGCTTCTTATTACTTGAGTAAATATAAAGGTATCCCAAGCTTCTGATATTTCTATTTGTTCACTTTTATATATTTTTTCCTCTTTCTCCTTTTCTTTTTTTGTATCTTCATTTTCAAAATAGGAAATTTTTAGTTCTGATTCTTGTTCTCTGCCCATCCAATTCCTAAAAATGAGATTCTTCATTTCGTTGATATCAAAAAACGAAAAAAAAGATGTTTTGTCTAGACGATCCAAAAAAAGTGGGGAATGTACATTTACTTGAAAGAGGTTCCAAATAACTGTTTTACGTCTTTGAGCGCGCATAGATCCTTTGATTATATTGCGGCGAAAATCCGATTGTTTTGAGTAACGTATCAAAGACACCTCTTCCTCTTCCATTTGATCATCATTTTTATCAGTGTTACTAATATTCTGAATACTATAAAAAAAATTGGTATTCTGATCATTATCGTTAGAAATTATCACACGTCTGGCTCTTCTTGAATGAATCGCAAAATCTTCTTCCTCCAATGCTTCTTCATTTTCTTCTTCCTCTTCTTCTAACAAATTCTCGGTTAATTTGTATGACCATCGAGAAACCTTTTTACTGAGTTCTTCTATTCTAATTCCAACAGATTCCTTTTTCAGTTTTTTTTGATCTTTTGTATGTGTTGTAATTACATAAAATACAAATTGCAAATATTTTGCTTGACTTTCTGAATCAATTCCTTTTTCTTCTGTAGAATTCAAAAATGATTGAGGGTAAAGTTTTACGGAATCATTAAGAAGAAGTAGATCATGAATCTTATTTATAAAAAAAAATTCTACTAAATCTTCTGTATCTGTATAAGTATTTATGATTGCGCGTGAATAAAATTTTTTTCTCATTCCTCGATATGGTCCGTTGAAAAAAGGATCATATGCTTTTGGCAAGCATTTTTTTTTTTTTTCATCATCACATAATATGGTTCTTTTTTCAAGCATATCCAGACTAGGGGATCCCTCATTTTTTTCTATAATTTTGATTCGGCTTCTCAATTCATTGTTCAAATTGCGCTTTTTTTTTTCATTAGTATAAATCCAAGAATCATAAAGATCTTCGTCATATAGTTTTTCTATTATGTACAAAGAAATTCTTCGTTCTAGCATTTCCGAAAAAGTTGATAAACTGGGCGGATATGTAAAGGATATTTTTTGTTTCCCATCACTTGTACATGTAAAAAAAAAAAATTGTGACATTTCCTTGCGTAAAGCATTTTCTAATTTAGAATTTTTTATATATCGTAATGGACGATTCCATCGTTTATAATCGAAAAGAAAAGAGACAATCTTTTTATTCATATTCATTATTTTCTTTTTCTCTTCTTTCAGTCTTCCCAATTCCCAATTTTCTTGATGGGTCTCCAGATCAGAATCTTCGTAAACTGGGCTATCTTGATAGCGTGCCTCTTTCAAGTGAAATTCATCCTTTGTTTTTTCCTTTCCATTAACTCGGATCTCTTCCGTTTCATCTATTTTGTCCAGACCCTCCTTTTCTTCCGAACAAAGGTTTTCTTCGGTGGATCCCTCTTGTTCCTGTTCAGTCTCCTTCATTTCGTAAGTTGTTTCTACATCGCTTTCTTCCGTTTCTGAGGTTTCTTTCTCTTTCAGTTTCTTAGTGACAATAGGCGACGGCATTCTGCCTAAATAGTAAACACAGGTGATAAATAAGAGAATGCTAAAGATTCGAGCCATAGAATTTCTCAATTCTGACACAAGATACTTATTAGATCGAATAGAATGATTTTGCCGTATCCAGAATAATACCAATCCAACCCATTTCATGAATAAAATGTGACCAATTAACCAACCAACAAAACTACTTGTTAAAAATAAAATCTTGTTGTTGCATCGAAACATATAAATGTTGACTAATCTGGCTAACGTGGAACTTGGTAAAATGAAATGGTTGAATAATTGAAAAATTAGATTATTCAGGAATACACATTGAATGCTGAGATTACGCATTGAATTTCTGGTAGTAGATCCATAATCAAAAAAGTTTTTATGATTGTTCCAGAAGAAATGAAACAAAAGATACGGTAGAACTAGGACAGTTATTGTATGAGGTCTACCCAATGCTAGATGCAGAGGCGCATAATAGATCGATATGAACATCATGAGCTGTCCCGCAATAAAACCAGTTGTTGCTGATACCTCCTTCTCGGTTCCTTCTTCCATAACCCGAGCTCGGAGAAGGAAGAGATAAGAGGGCCCTATGGAGAATGTGGTCATAAATCCATAATAGAGCCCGACCACAACGACCGAATTTATTATCTTCATGCATAAGGATAATGGATTACCCAGTAGAAAAGATTTCAAAATCATCACAAACCTCCCCTTTTTCTTTTCTATTGCAATTTATCGATTATTATATGATGATTCCTTAACTTTCCATATCTATATAGATAGAAACAGATATACTATAAATGACATCTCTTATGTCAATGACACAAAAGGGATATGAAATGAATGGAATTGGGATATAGATGGAATATAATGAAATAGAGCCACATTGAGGTTCCCTATGAAATGAGGCATGGAACGGAGCCACTACGAAGAAGTTCCTGGAGTTACGAAGGAAGCTTCGGACTCATATTGTTCATGGGTTGAGAACGGGAGTTGAACTCTATGAGGTCGAATCTCCCGTTGTTCCTCAGTAGCTCAGTGGTAGAGCGGTTGGCTGTTAACTGACTGGTCGTAGGTTCGAATCCTACTTGGGGAGATTTGATTCATTCTTCATGAAAGAATGAAGAATTGAATGAAAGGGCTCGCTTTGACCGTTAGGAGTAGGTAATCTCGTTCCCTGTCTTTGTTTCTATTGCATTCTATCTCATCGTATCACATTCTGTTCTACAATATTTGAGAATTACCCTCAATACCTCGGCATAGATCCAACCCTTGCAAATAGCCAATTCAGAATTCTCAACAGTGCATCAAAGATGCAGTCATCAATTCTCCCGATAGGCCACAATTACCGCGAGCAAACATATTAATGACGAGGAACGCATTTTTGCTATGCTACTAATACTTGTACTTGCTCTGCTATTCTGCCCAAGCCTGGCTGAGGAAGAGTTACGGGGAGTCAAACACAAATATGCTGATTCCAGCCCTGTTTACTATATGTAATAAACTAAACCTGACTTATAAGGACCATACCCAAGTTCAATAGCTTTGTGGCCGCTATCCCGATCATGATTTTACTACTCCCAGAGGGAAAGGCCCTTTTGGGCCGGTTGTGGGCGAGGAGGGATTCGAACCCCCGACACCATGGTTCGTAGCCACGTGCTCTAATCCTCTGAGCTACAGGCCCCACCCCGTCTACACTGGATCTGTTCCCGGGAGTACCTTCAAAAAGGAACCTTTCCTCTCCTCAGCCATTTTGGGTTAAGAAGATGTGAAAGCGCCTTTCTCTCTAGAATAACAGTGCGTTCCGAGGCGTGAAGTGGGAGAGAAGGGATGTTATTGAATAAGACGACCTTTGCATTTTTGATTTTGATCTTTTGAAATATGAAAAAGTAAAATAAGAGGTGTTAAGCTTTTTATCATTCTGGCGTCGAGCTATTTTTCCGCAGGACCCCCCCTACAGTATCGTCACCGCAGTAGAGTTTAACCACCAAGTTCGGGATGGATTGGTGTGGTTCCTCTACGCCTAGGACACCAGAATATCGAACCATGAACGAAGAAAGGCATGAGATAAATATTGTCTAGTGATTGTGAGGCCCCGATTCTTGACTGGAAGGGACGCCAAAGGCCTCTGCCCTTCCATCCGATAGAGAGGGAGGGCAGAGCTTTTGGGTTTTTTTTCATGTTGTCAAACAGTTGAACAATGAAAATAGATGGCGAGTGCCTGATCTAATTGATCAGGTCGTGTAGGAACAAGGTTCAAATCTCTCGGTCTGTTAGGATGCCTCAGCTGCATACATCACTGCACTTCCACTTGACACCTATCGTAATGATAAACGGCTCGTCTCGCCGTGACCTTATCTTGGATTCTCAATACTTCTGTCGCTCCATCCCCGCAGGGGCAGAGAACCCGCCGCTGTCTCGGCTGTGCTACCGGAGGCTCTGGGGAAGTCGGAATAGGAGAGCACTCATCTTGGGGTGGGCTTACTACTTAGATGCTTTCAGCAGTTATCCGCTCCGCACTTGGCTACCCAGCGTTTACCGTAGGCACGATAACTGGTACACCAGAGGTGCGTCCTTCCCGGTCCTCTCGTACTAGGGAAAGGTCCTCTCAATGCTCTAACGCCCGCACCGGATATGGACCGAACTGTCTCACGACGTTCTGAACCCAGCTCACGTACCGCTTTAATGGGCGAACAGCCCAACCCTTGGAACATACTACAGCCCCAGGTGGCGAAGAGCCGACATCGAGGTGCCAAACCTTCCCGTCGATGTGAACTCTTGGGGAAGATCAGCCTGTTATCCCTAGAGTAACTTTTATCCGTTGAGCGACGGCCCTTCCACTCGGCACCGTCGGATCACTAAGGCCGACTTTCGTCCCTGCTCGACGGGTGGGTCTTGCAGTCAAGCTCCCTTATGCCCTTGCACTCGAGGGCCAATCTCCGTCCGGCCCGAGGAAACCTTTGCGCGCCTCCGTTACCTTTTGGGAGGCCTACGCCCCATAGAAACTGTCTACCTGAGACTGTCCCTTGGCCCGTAGGTTCTGACACAAGGTTAGAATTCTAGCTCTTCCAGAGTGGTATCTCACTGATGGCTCGGGCCCCCCCGCAAGGAGGCCTTCTTCGCCCTCCACCTAAGCTGCGCAGGAAAGGCCCAAAGCCAATCCCAGGGAACAGTAAAGCTTCATAGGGTCTTTCTGTCCAGGTGCAGGTAGTCCGCATCTTCACAGACATGTCTATTTCACCGAGCCTCTCTCCGAGACAGTGCCCAGATCGTTACGCCTTTCGTGCGGGTCGGAACTTACCCGACAAGGAATTTCGCTACCTTAGGACCGTTATAGTTACGGCCGCCGTTCACCGGGGCTTCGGTCGCCGGCTCCCCTGTCATCAGGTCACCAACTTCCTTGACCTTCCGGCACTGGGCAGGCGTCAGCCCCCATACATGGTCTTACGACTTTGCGGAGACCTGTGTTTTTGGTAAACAGTCGCCCGGGCCTGGTCACTGCGACCCCCTTTTTGAGGAGGCACCCCTTCTCCCGAAGTTACGGGGCTATTTTGCCGAGTTCCTTAGAGAGAGTTGTCTCGCGCCCCTAGGTATTCTCTACCTACCCACCTGTGTCGGTTTCGGGTACAGGTACCCTCTTGTTGAAGGTCGTTCGAGCTTTTCCTGGGAGTATGGCATGGGTTACTTCAGCGCCGTAGCGCCTGGTACTCGGACATTGGCTCGAGGCATTTCCTCTACCCCTTCTTACCCTGAAAAAGCAAGGTCACCTTGCGTCCTTGAACCGATAACCATCTTTCGGCTAACCTAGCCTCCTCCGTCCCTCGGGACCAACAAGGGGTAGTACAGGAATATTCACCTGTTGTCCATCGACTACGCCTTTCGGCCTGATCTTAGGCCCTGACTCACCCTCCGTGGACGAACCTTGCGGAGGAACCCTTAGGTTTTCGGGGCATTGGATTCTCACCAATGTTTGCGTTACTCAAGCCGACATTCTCGCTTCCGCTTCGTCCACACCTGCTTGCGCGGGTGCTTCCCTCTAAGGCGGAACGCTCCCCTACCGATGATTTTTACATCCCACAGCTTCGGCAGATCGCTTAGCCCCGTTCATCTTCGGCGCAAGAGCGCTCGATCAGTGAGCTATTACGCACTCTTTCAAGGGTGGCTGCTTCTAGGCAAACCTCCTGGCTGTCTCTGCACCCCTACCTCCTTTATCACTGAGCGGTCATTTAGGGGCCTTAGCTGGTGATCCGGGCTGTTTCCCTCTCGACGATGAAGCTTATCCCCCATCGTCTCACTGGCCGACCTTGACCCCTGTTTGGGGTCATATCTAGTATTCAGAGTTTGCCTCGATTTGGTACCGCTCTCGCGGCCCGCACCGAAACAGTGCTTTACCCCTAGATGTCCAGTCAACTGCTGCGCCTCAACGCATTTCGGGGAGAACCAGCTAGCTCTGGGTTCGAGTGGCATTTCACCCCTAACCACAACTCATCCGCTGATTCTTCAACATCAGTCGGTTCGGACCTCCACTTAGTTTCACCCAAGCTTCATCCTGGTCATGGATAGATCACCCAGGTTCGGGTCCATAAGCAGTGACAATCGCCCTATGAAGACTCGCTTTCGCTACGGCTACGGTGGATTCCCTTAACCAAGCCACTGCCTATGAGTCGCCGGCTCATTCTTCAACAGGCACGCGGTCAGAGATCACATTCTCCTCCCACTGCTTGGGAGCTCACGGTTTCATGTTCTATTTCACTCCCCGATGGGGGTTCTTTTCACCTTTCCCTCACGGTACTACTTCGCTATCGGTCACCCAGGAGTATTTAGCCTTGCAAGGTGGTCCTTGCTGATTCACACGGGATTCCACGTGCCCCATGCTACTCGGGTCAGAGCGTAAGCTAGTGATGCTTTCGGCTACTGGACTCTCGCCATCTAGGGTGCAGCACTCCACCGCTTCGCCTAGCAGCATGACGCTTGTATTGCTCTCCCACAACCCCGTTTTCACGGTTTAGGCTGCTCCCATTTCGCTCGCCGCTACTACGGGAATCGCTTTTGCTTTATTTTCCTCTGGCTACTAAGATGTTTCAGTTCGCCAGGTTGTCTCTTGCCTGCCCATGGATTCAGCAGCAGTTCGAAAGGTTGACCTATTCGGGAATCTCCGGATCTATGCTTATTTTCAACTCCCCGAAGCATTTCGTCGCTTGCTACGCCCTTCCTCGTCTCTGGGTGCCTAGGTATCCACCGTAAGCCTTTCCTCGTTTGAACCTCGCCATTCACGTGAAGGCTATGCCATCCTAAGGTGCTGCTAAATGGAAGGATCTTATCAACGTCCATGAATGAGAAATCATAGATCGAACTGCCGAATTGGAAAAATTGGGTGCTATCATAGTATCAGCTAAGTTCACGGGCTGGAGATAAGCGGACTCGAACCGCTGACATCCGCCACAGGGTAAACCACCGCCTCTCAGGCCTCTCCGGCTGATTCTACCATAGAGGCCAACGATAGACAATAACTCCCCCCCGAACACAGCTTACAACTTTCATCGTACTGTGCTCTCCAAAGAGCAACTCTTCTCAAAATCTCAAAGGGTGCTGAGTTGGAATACCATTCTAAGGATCCTTGTGGTTCCGGAGAATCCAGCTACAGGAGAACCAGGAACAAAGAGCTATCCCCCCCTTTCCGCCCGACTCTTTGGTCTTCAGAATGCTGGTTTTAAGAACGAGTGATTGCCCTTCTCCGACCCTTACTGCCCAACCGGAGAGCGGACAGCTAATGCCTTCCACTTATTGAACAGGGTCTATGGTCGGTCCGTGACCCCTGGATGCCGAAGGCGTCCTTGGGGCGATCTCGCAGTTTCTACGGGGTGGAGACGATGGGGTCGGTCCATGGATTTTCCTTCCTTTTGCCACATTTCGCTCAAAGGGTTGAAGGGAGATAGTGCATCAAGCTATTCGCAAGGGCCAACTTGATCCTCTTTCCTAGGGATCCCAGATGAGGGAACCCTAGGAGAGCCGCCGACTCCAACTATCGTCCATGTACGATCCATACTAGATCTGACCAACTGCCCATCCTACCTCCTCTACGTTCTTGACAGCCCATCTTTTTGTCTCAGTAGAGTCTTTAAGTGGCATGTTTCGGTCCTCTTCCCCATTCCTTAGAAAAAGTGAGCCACCGGTTCAGGTACAAGATACTATCATTACCGCCTGGACAATTAGACATCCAACCCGTAATCGCAACGACCCAATTGCAAGAGCGGAGCTCTACCAACTGAGCTATATCCCCCCGAGCCGAGTGAAGCATGCATGAAAGAGTCAGATGCTTCTTCTATTCTTTTCCCTGGCGCAGCTGGGCCATCCTGGACTTGAACCAGAGACCTCGCCCGTGAAGTAAATCATCGCACCTACGATCCAACCAATTGGGAGAGAATCAATAGATTCTTTTTCGGGAGCGATTCATCCTTCCCGAACGCAGCATACAACTCTCCATTGTACTGCGCTCTTCAAGTGTGCTTGTTCCCCTTTCTCCCTTACCATGGCAAGTCTTTGTGAAATCACTCCGATGGGAAGAAAAAAGAAAGCGTTAAGAGACCCTCCTGGCCCAACCCTAGACACTCTAAGATCCTTTTTAAAACCTGCTCCTCCTCCCATTTCGAGTCAAGAGATAGATAAATAGATACATCCCATTGCACTGATCGGGGAGCGCTCGTAGTGACTGAGGGGGTCGAAAACCAAGAAGTGAGTTATTTATACCAAGCATTCTTCTTATGTTATGGCTAGATCCAATCTCCTGGTCCCTGCGGAAAGGAAAAAGAATTTAACGTTCTTCCTTTCGGGAAGGGAGGATTCGGGAAATCCTATTGATTGCTGCTTTCTTCAGACCTCCGGCATGAAAAAAAGGCTCGAATGGTACGATCCCTCCGTCACCCCAGAATGAAAGGGGCGATCTCGTAGTTCTTGGTCTGTGAAGATGCGTTGTTAGGTGCTCCATTTTCCCATTGAGGCCGAACCTCAACCTGTGCTCGAGAGATAGCTGTCCATACACTGATAAGGGGTGTATGGATTCTCGAGAAGAGAGGAGCCGTGGGGGTCCCCCCCCCGGACCGCCCGGATCCCACGAGTGAATAGAAAGTTCGATCTCCATTGGATCTCACCTGAATCGCCCCATCTATCCTCCTGAGGAGAAGTTTGGTTTCAAACTCCGATTCGAACAGGAGGAGTACGCCATGCTAATGTGCCTTGGATGATCCACATCTCCGGGTCAGGCGCCGATGAGCACATTGAACTATCCATGTAGCTGAGAGCCCTCACAGCCCAGGCACAACGACGCAATTATCAGGGGCGCGCTCTACCACTGAGCTAATAGCCCGTCGTGTGGGCCTCCCTGGGGGAGGCCTGCTATGCCAAAAGCGAGAGAAACCCCATCCCTCTCTTTCCTTTTGTACGTCCCCATGTCCCTCCCGTGTGGCGACATGGGGACGTACAAAAGAGATCCTATCAACTTGTTACGACCTAGGATAATAAGCTCATGGGCTTGGTCTTACTTCACCGTCGAGAAACGAAAGAAGACTTCCATATCCAAGCTTAGCTCAGATGTAGCTCGCTTCTTTTTGGGTGTCAAGCAGTGTCAAACCAAAATACCCAACAAGCATAAGCATTAGCTCTCCCTGAAAAGGAGGTGATCCAGCCGCACCTTCCAGTACGGCTACCTTGTTACGACTTCACTCCAGTCACTAGCCCTGCCTTCGGCATCCCCCTCCTTGCGGTTAAGGTAACGACTTCGGGCATGGCCAGCTCCCATAGTGTGACGGGCGGTGTGTACAAGGCCCGGGAACGAATTCACCGCCGTATGGCTGACCGGCGATTACTAGCGATTCCGGCTTCATGCAGGCGAGTTGCAGCCTGCAATCCGAACTGAGGACGGGTTTTGGAGTTAGCTCACCCTCGCGGGATCGCGACCCTTTGTCCCGGCCATTGTAGCACGTGTGTCGCCCAGGGCATAAGGGGCATGATGACTTGACGTCATCCTCACCTTCCTCCGGCTTATCACCGGCAGTCTGTTCAGGGTTCCAAACTCAATGGTGGCAACTAAACACGAGGGTTGCGCTCGTTGCGGGACTTAACCCAACACCTTACGGCACGAGCTGACGACAGCCATGCACCACCTGTGTCCGCGTTCCCGAAGGCACCCCTCTCTTTCAAGAGGATTCACGGCATGTCAAGCCCTGGTAAGGTTCTTCGCTTTGCATCGAATTAAACCACATGCTCCACCGCTTGTGCGGGCCCCCGTCAATTCCTTTGAGTTTCATTCTTGCGAACGTACTCCCCAGGCGGGATACTTAACGCGTTAGCTACAGCACTGCACGGGTCGATACGCACAGCGCCTAGTATCCATCGTTTACGGCTAGGACTACTGGGGTATCTAATCCCATTTGCTCCCCTAGCTTTCGTCTCTCAGTGTCAGTGTCGGCCCAGCAGAGTGCTTTCGCCGTTGGTGTTCTTTCCGATCTCTACGCATTTCACCGCTCCACCGGAAATTCCCTCTGCCCCTACCGTACTCCAGCTTGTTAGTTTCCACCGCCTGTCCAGGGTTGAGCCCTGGGATTTGACGGCGGACTTGAAAAGCCACCTACAGACGCTTTACGCCCAATCATTCCGGATAACGCTTGCATCCTCTGTCTTACCGCGGCTGCTGGCACAGAGTTAGCCGATGCTTATTCCTCAGATACCGTCATTGCTTCTTCTCCGAGAAAAGAAGTTCACGACCCGTGGGCCTTCTACCTCCACGCGGCATTGCTCCGTCAGGCTTTCGCCCATTGCGGAAAATTCCCCACTGCTGCCTCCCGTAGGAGTCTGGGCCGTGTCTCAGTCCCAGTGTGGCTGATCATCTTCTCGGACCAGCTACTGATCATCGCCTTGGTAAGCTATTGCCTCACCAACTAGCTAATCAGACGCGAGCCCCTCCTCGGGCGGATTCCTCCTTTTGCTCCTCAGCCTACGGGGTATTAGCAACCGTTTCCAGTTGTTGTTCCCCTCCCAAGGGCAGGTTCTTACGCGTTACTCACCCGTCCGCCACTGGAAACACCACTTCCCGTCCGACTTGCATGTGTTAAGCATGCCGCCAGCGTTCATCCTGAGCCAGGATCGAACTCTCCATGAGATTCATAGTTGCATTACTTATAGCTTCCTTGTTCGTAGACAAAGCTGATTCGGAATTGTCTTTCATTCCAAGGCCGAAGGCTTGTATCCATCCATGCGCTTCATATTAGCCTGGAGTTCGCTCCCAGCAATATAGCCATCCCTACCCTATCACGTCAATCCCACAAGCCTCTTATCCATTCTCGTTCGATCACGGCGGGGGAGCAAGTCCAAATAGAAAAACTCCCATTGGGTTTAGGGATAATCAGGCTCGAACTGATGACTTCCACCACGTCAAGGTGACACTCTACCGCTGAGTTATATCCCTTCCCGGTCCCATCGAGAAATAGAACTAACTAATCCTAAGGCAAAGGGTCGAGAAACTAAACGCCACTATTCCTGAACAACCCGGAGCCGGGCCTTCTTTTCGCACTATTCTATTATGGATAGTTAAATAATGGGAAAATTTGGATTCCATTGTCAACTGCTCCTATCGAAAATAGGATTGACTACGGATTCGAGCCATAGCACATGGTTTCCTAAAATCCGTTTTCCCGATCTAAATCGAACAGTTTTTACATGAAGAAGATTTTTTTCAGCATGCTCTATTCGAGACTGGTAGGAGAAGAACCGACTCGGTATTGTAAAAAAAAAAGAGCGGAAGCAGAACCAAGTCAAGATGATATGAATCACCCCTTCTTCTTGCGCCAAGGATCTTACTACCATTTCCGAAGGAACTGGAGCTACTTTTCTTTTCCATTCCAGAGTTCCTATGTGACGCCCTTTTCTTAAGAACACATACAATAAAAAAGGATAACGGTAACCCCACCATTAACTACTTCATTTTCATTTATGAATTGAATAGTAATAGAAATACATGTCCTACCGAGACAGAGACAGAATTTGTAACTTGATATCCTCTTGCCTAGCAGGCAAAGATGGACCCCCGTGGAAAGACTGATTCATTCGGATCGACATGAGGGTCCAACTCCATTGCATTGCCAGAATCCATATTGTATATTTGAAGCAGTTGACCTCCTTGCTTCTCTCATTCTCATGGTACAACCCTCTTCCCGCGGAGCCCCCTTTCTCCTCGGTCCACAGAGACAAAATGTAGGACTGGTGCCAACAGTTCATCACGGAAGAAAGGACTCGCCGAGCCGGGATCACTAACTAATACTAAAACTAAGACTATAAGACTAATATAACTAATCTAATTAGAAAGAATTCTAAGAATTAGATAATTAGAATAGAATTTAGAATATAATAATATATAATAATACATAATAGAAAAGAACTGTCTTTTCTGTATACTTTCCCCGGTTCCGTTGCTACCGCGGCCCTTACGCAATCGATCGGATCATATAGATTTCAACACAACATAGGTCATCGAAAGGATCTCGGACGACTCACCAAAGCACGAAAGCCAGGATCTTTCAGAAAATGGATTCCTATTTGAAGAGTGCATAACCGCATGGATAAGCTCACGCTAACCCGTCCATTTTGGATCCAATTCGGGATTTTCCTTAGGAGGTATCGGGAAGGAATTGGTCTGAGTTACTCTTCGGGACGGAGTAGAAGAAGGGAGGAGATTCTCGAACGAAGAAAAGGATCCAATTACTTCGAAAGAATTGAACGAGGAGCCGTATGAGGTGAAAATCTCATGTACGGTTCTGTAGAGTGACAGTAAGGGTGACTTATCTGTCAACTTTTCCACTATCACCCCCAAAAAACCAAACTCTGCCTTACGTAAAGTTGCCAGAGTACGATTAACCTCTGGATTTGAAATCACTGCTTATATACCTGGTATTGGCCATAATTCACAAGAACATTCTGTAGTATTAGTAAGAGGAGGAAGGGTTAAGGATTTACCCGGTGTGAGATATCACATTGTTCGAGGAACCCTAGATGCTGTCGGAGTAAAGGATCGCCAACAAGGGCGTTCTAGTGCGTTGTAGATTCTTATCCAAGACTTGTATCATTTGATGATGCCGTGTTAATCGCTAGAAACATGTGAAGTGTATGGCTAACCCAATAACGAAAGTTTCGGAAGGGGACTGGAGCAGGCTACCATGAGACAAAAGATCTTCTTTCTAAAGAGATTCGATTCGGAACTACTATATGTCCAAGGTCCAATATTGGAATAATTTAAGAGGTTTTCCCTTCCTTTGTCCGTGTCAACAAACAATTCGAAATACCTCGACTTTTTCAGAACAGGTCCGAGTCCAATAGCAATGATTCGAAGCACTTCTTTTTCCATTACACTATTTCGGAAACCCAAGGACTCGATCGTATGGATATGTAAAATACAGGATTTCCAATCCTAGCAGGAAAAGGGGGGAAACGGATACTCAATTTAAAGTGAAAGTGAGTAAACAGAATTCCATACTCGATCTCATAGATACATATAGAATTCTGTGGAAAGCCGTATTCGATGAAAGTCGTATGTACGGCTTGGAGGGAGATTTTTCATATCTTTTTAAATCCACCCTACAATATGGGGTCAAAAAGCCAAAATAAGTGATTTGTTTTTAGCCCTTATCAAAATAAAACTGATTCTTGAACCTCTTTCACGCTCATGTCACGTCGAGGTACTGCAGAAGAAAAAACTGCAAAATCCGATCCAATTTATCGTAATCGATTAGTTAACATGTTGGTTAACCGTATTCTGAAACACGGAAAAAAATCATTGGCTTATCAAATTATCTATCGAGCCGTGAAAAAGATTCAACAAAAGACAGAAACAAATCCACTATCTGTTTTACGTCAAGCAATACGTGGAGTAACTCCTGATATAGCAGTAAAAGCAAGACGTGTAGGCGGATCGACTCATCAAGTTCCTATCGAAATAGGATCTACACAAGGAAAAGCGCTTGCCATTCGTTGGTTATTAGGGGCATCCCGAAAGCGTCCGGGTCGAAATATGTCTTTCCGATTAAGTTCCGAATTAGTAGATGCTGCCAAAGGCAGTGGCGATGCCATACGCAAAAAGGAAGAGACTCATAGAATGGCAGAGGCAAATAGAGCTTTTGCGCATTTTCGTTAATCCATGAACAGGATCTATATAGACACATAGATCCATGGATCCATACATCTCGATCGGAAAAGAATCAATAGAAAAAGAAAGAATCGGAATTGATCGAGATCTTTCTCGAAACAAACAAAAAGAAAGATGAAACATAAATCATGGATCAACTAAGCCTTCTCGGGGGCTTGCTTAAGAATAAGAAAGAGGAATCTTATGGAAATATCATGGAATAAGGTTTGATCCTATTCATGGGGATTCCTTAAATATCCCATTCCAAAAATAGAAAGTTCGAAACAATTGGGACTTTTTCGTAGATTGGATGCAGTTACTAATTCATGATCTGGCATGTACAGAATGAAAACTTCATTCTCGATTCTACGAGAATTTTTATGAAAGCGTTTCATTTGCTTCTCTTCCATGGAAGTTTCATTTTCCCAGAATGTATCCTAATTTTTGGCCTAATTCTTCTTCTGATGATCGATTCAACCTCTGATCAAAAAGATAGACCTTGGTTCTATTTCATCTCTTCAACAAGTTTAGTAATGAGCATAACGGCCCTATTTTTCCGATGGAGAGAAGAACCTATAATTAGCTTTTCGGGAAATTTCCAAACGAACAATTTCAACGAAATCTTTCAATTTCTTATTTTACTATGTTCAACTCTATGTATTCCTCTATCCGTAGAGTACATTGAATGTACAGAAATGGCTATAACAGAGTTTCTGTTATTCGTATTAACAGCTACTCTAGGAGGAATGTTTTTATGTGGTGCTAACGATTTAATAACTATCTTTGTAGCTCCAGAATGTTTCAGTTTATGCTCCTACCTATTGTCTGGATATACCAAGAGAGATGTACGGTCTAATGAGGCTACTACGAAATATTTACTCATGGGTGGGGCAAGCTCTTCTATTCTGGTTCATGGTTTATCTTGGCTATATGGTTTATCTGGGGGGGAGATCGAGCTTCAAGAAATAGTGAATGGTCTTATCAATACACAAATGTATAACTCCCCAGGAATCTCAATTGCGCTTATATCCATCACTGTAGGAATTGGGTTCAAGCTTTCCCCAGCCCCTTTTCATCAATGGACTCCTGATGTATACGAAGGAGTGCGGTTCGTTCGACAAATTCCTACCTCTATATCTATCTCTGAGATGTTTGGATTTTTCAAAACTCCATAGACATGCAGAAGAGAAATGCTATCCCCACTCGGACCAAGACATCACTTTGACCAAAAGTTTATTGTGATCCTTTTGTTCAAATAACAATTAAGGTGAAGCAGGGTCAGGAACAACGAATCTCTTTATGATAAACAGATCCCTTTTGCAAGTTCGTTATTACGGGTAGTTCTACAAAGGATCGGACTAATGACGTATACAATACTTGAATTATCGATGTAGATGCTACATAGTTGGTTCTCATCCTTCAGAGACTACGAGTGTAATAGGAGCATCCGTTGACAAAAGGATCACCCTAAGATGATCATCTCATGGCTATGGAGAACGAATCAAATCAGATGGTTCTATTTCTCAATCTTTCTGACTTGCTCCTACGGAACCAAGGTCGAAAGGATTGAAAAAGTCAGTCATTCACAACCACTGATGAAGGATTCCTCGAAAAGTTAAGGATTAGTAATCCTTTTTAGAAATCGAATGGATTCGGTCTTATACATACGCGAGGAAGGTAATCAAAAAAGAAAGAAGAACTACTCTTCTTTCTTTTATCACTTAGGAGCCGTGCGAGGTGAAAGTCTCATGCACGGTTTTGAATGAGAGAAAGAAGTGAGGAATCCTCTTTTCGACTCTGACTCTCCCACTCCAGTCGTTGCTTTTCTTTCTGTTACTTCGAAAGTAGCTGCTTCAGCTTCAGCCACGCGAATTTTCGATATTCTTTTTTATTTCTCATCAACCGAATGGCATCTTCTTTTGGAAATCCTAGCTATTCTTAGCATGATATTGGGGAATCTCATTGCTCTTACTCAAACAAGTATGAAACGTATGCTTGCATATTCGTCCATCGGTCAAATCGGATATGTAATTATTGGAATAATTGTTGGAGACTCAAATGATGGATATGCAAGCATGATAACTTATATGTTGTTCTATATCTCCATGAATCTAGGAACTTTTGCTTGCATTGTATCATTTGGTCTACGTACCGGAACTGATAACATTCGAGATTATGCAGGATTATACACGAAAGATCCTTTTTTGGCTCTCTCTTCAGCCCTATGTCTCTTATCCCTAGGGGGTCTTCCTCCACTGGCAGGTTTTTTCGGAAAACTCTATCTATTCTGGTGTGGATGGCAGGCAGGCCTATATTTCTTGGTTTCGATAGGACTCCTTACGAGCGTTGTTTCTATCTACTATTATCTAAAAATAATCAAGTTATTAATGACTGGGCGAAACCAAGAAATAACCCCTCACGTGCGAAATTATAGAAGATCTCCCTTAAGATCAAACAATTCCATCGAATTGAGTATGACTGTATGTGTGATAGCATCTACTATACCAGGAATATCAATGAACCCAATTCTTGCAATTGCTCAGGATACCCTCTTTTAGCTTCTAGGTCTATTTTTTAGTTCAAGATCCCTTTTACTAACTGGAATAAAAAAATTACTTTTCCGCCCAAAATGGGAATGGGCTGGAGTTATGAACTTATAATCATGGAATCGAGTCGATTCCATGATTATAAGTTCATTCCAGACCGGACCAGGCCGGAATAGGGTTATGTACATTCTTATTATGAGTAAGGGGTCATTCGAGCGTATGTAAATAGATACTCTGTTTACATATGGATCCCTACATCGTTACATTCTATTTAGGATTAGGAATAGGCGTAATCGGACCCGCTTTTGACATATCTATCGTTATTTGGGTACCCTATTCACTTCTTTGGGCTTCTCTTGAATCGAGAAAGAGGTTTGATTTTACATCTTTTTGATATATAGTTTGATATATAGAAGGTATCCTCCGGATAATTCCAATCGAAGCAATTTGATGTCTGACTCGGGCCTATATGACATGACCGATCGATAGAAATACTCCAACACTCCACCTTTGTCATATATTCCATATATCACACTAGATAGATATCATATTCATGGAATACGATTCACTTTCAAGATGCCTTGATGGTGAAATGGTAGACACGCGAGACTCAAAATCTCGTGCTAAAGAGCGTGGAGGTTCGAGTCCTCTTCAAGGCATAAACAGATTTCGGATCTAATAAATATTGACGAGTATCTTATCTGTTGATACGAAGTATTCCGGCGATCCTCACGATCCGAGTCCGAGCTGTTGTAATCGGCAGCGGATCACGAAATCTTGGCGATCTTCTCTATAGGAGTCCGTTTGGAAATCGTCCGCCCTGCGCGCACCCCCCGAGTATAGGATTCAACAGGAATCGCACAAGGTAGATTGATAGAAAAACCTCTGGTAAAATACCCACCAGTACCCGTAACCCAGCAAAGAAAGTACATTACATTAGGGATTGGCGACTTACCCATTCAGTGACTTTGGCACTGGACGTTCTCAAAATGGGTACTATCGGGTCGGGTGAATTAGAGAATAGACAGACGTCTGTTGGCATTCCAGCCTTCCTTCTCCTTTCAGGGCCTATCCGAAAGAGGCTCTTGGTCGTGAATATCTGAATAGGACGAACCCGCCTCCGTGGATATCTTTGCTTCGGAACAAAGCAATTAGAATTAGGCTCGGTCAACTGGAATGTGTATTATCCGTATGGGAGATCTTCCAATTGAGGAGATCCATCGACCTGAGACGAAGAGAAAGGTCTATCTATCTTCTTTAGTTATTCAATGAAACCAATGATTCGTTATTGGAGCAGATAGCAACAACCATTTCAGCGGACATGCGTATTTTCCGATGGATTTACATGGTTTCATTAGTATAAATTGTTTGATGTAGCGAGTAATAGGCTCTTTCGCCGTTCAAGAATTCTTGTTTAAGCAGTTCATATCATCCACACATAGTGATCTAAGATTTCAATTCTTCCATGTTTCAGCAGTAGCATATTGTTCCACGGAGTTAAGGCCAAAAAGATGGAAGAAACAAGTGTTTCCACGACTCTACCATCCGGCCAATTCTGTTCCACTTAATCCCCTTTTCATGGGCACATATCTTTACGGCTAAGGAATGGGGAATCTTTCTCCTGTTACATGAATCAAATGTTTCATTTCATCCGGGAAAAGCCATCTCTTTCTCAACAATGTCTTTGTCATTTGATCCAATAGCGTTCCGTTAGATAGGAACAGATTTGATAAATACTGATAACTCTCGGATAGAGTATTAGAACGGAAAGGTCCATTAGATAATGAACTATTGGTTCTAAACCATCTCTGGCGATGAATCAACAATTCGAAGTGCTTTTCTTGCGTATTCTTGATGAACCAGCGTTTATATATAGATGTAGGAGGATTTGTTTGGGAAGCAATAAGCCCCTTTGACATCTCTTCATCTGCAAAGAATTCTCGACGTGAAAACACAGAGACAGAGGGCTGATCTTTGAATAGGGAAAAGAATGGATCCGCAGGGTCCCAAATGAATTGGCTTGTTCGAAAAAAGCCTTGTTCTTTGGAAGATCTATCTCGTGTCTGGTACTGCACGGTTCCACTCTGCAAGAACTCCGAATCATTCTCTTGAAGCTCATCCTCTTTATGATAAATGATCCATTTGCCCCGAAATGACCCAGTCCAATAGGGAAATCCCAATTCAGTGGGCCTTTCGATACAATCAAATAGATTAGGGCGCCATATTCTAGGAGCCCAAACTATGTGATTGAAGAAATCCTCCTCTATCTGTTGCGGATCGAGGGCCCCTTCCCCTTCTTCAAACTCCGATTCGTATTTTTCATATAGAAATCTCTGATCAACGATAGAACAAGATCCATTTTGCATCATATCTAACTGATTCCTTGGTTCGGACCGAAGAAGTAATGTCACTCGATTATTATCAAACTGACTACAATATTTTTCTGTCCGTGAGGATCCCGCCAGAGCCAGAGTGCCTTCTACTTCTAATAGTAATAATAGTAATAGGCCATGAACTAGATCAGAATCATTCTCAACGAATCCATAAGAAGTGATCCAATTTTTTTCATCGTGTCTGGATGAAGACCAAAGATCTTGAGCGACCGATCCGGCAGAACAACTCAAAAGATAAAGAAGTATCGTTAATTTCTTCATGCTCGTTCCAAGTTCGAAGTACCATTTGTACAAATAAGAATCCCCTCCCTTACATGATTTCTTCTTCATATAGATAGATATAGGATCTATGGGGCAATTACTTAGAAGTATATTTTGTGTAACAGCCCTTCCTATCTGATAGAAAAGGATCCCATGATCCTGAACCGATCTTATCTGGGATCGAAAATCCCAAGTTTTTCTATGAAGAGCTGATCTAATTGTATTAGTTTCTATAATGGATTTCTTCTGTGTAATACTAATTGATAGGGCCTCATTGATAAGTGCTACAAGATCTCGCGCATTGGAACCCATGGTTATGGACCCGAATCCGTTAGTATGGAACATCTTCTTTTCCAAGTGAAATCCCCTAGTATATGAAAGAATGAAAAAGTGCTTTCGTTGTTGTGGAAGAAGAAGCCTTCGTATCTTAATGCATGTATTTAATTTATTCGGAGCTATTAGAGCGGGATCCACTTTTTGGGGAATATGAGTCGAAGCAATAACAAGAATCTTTCCAGTGGAACATCTTTCACAATCCCTGGAGAGATAGTTCTCTAATAGACCGAGGGATAAGTAATTCGACTCATTCACATGCAGATCATGAATGTTTGGAATCCATATTATGCAAGGAGACATTGCTTTTGCTAATTCGAATTGAAGGGTGATATCAAATCGGTCTATTTTCGGCGTCATATACATAGTTAGCACATTCGTCATAGTTAGCAGCTTCGTATCAAGGTCATCATCAATATCGTCACTATCATCAATATCGTCACTATCATCAATATCATCAATAAGATAACCTTTAGGCTTGTCATCCAGGAACTTGTTCGAAAATACCGTAATGAAAGGAACATAGGAGTTTGTCGCTAGGTATTTGACCAAACAGGATCGTCCAGTTCCTATAGAACCTATCACTAAAATACCTCTAGAAGGGGATAGGGCTAAGCGGAGCGAAAAGGGTTTTCCATGAGGAGATGGGGAATGAAAACTATTAGCCCCACACGAGGCTTGTGAATAAGTGATTGTCTGATAATGAGCAAGGAATATCCGTCTTTCTGCTAAACAGGATCTATTGAACTCATAATTCATTAGATCCTTTTGATGAATGTCAACTAAGTATCGTAAGGAAATTGATCCCGGTTGTTCAATCATTTGATAACCAGAGTCATTCTTTGATAAATGATCACTATGAGTCAGACTCAATAGAATTTGATCAATCCTTTTTTCTGTCGTTAAGGTGGAGAACTGAACCAAGAATTCTCTTTCTTCATCATCAATCGAATCACTGTTCGCGACCCAGAATTCTATTTTCTCATCAATCCAATCACCGTTCACGTTTTTTCTTTTTCTTATCAATGAATAGATCTCTTTACTTGTACGACTTAGATGTCTCGTATTTCTCGAAAAAATGATTCGATTGATGGGATTTGGTATGATACTTACAAGATCGATGAGATTGATCTTCCAATATTTCTTCTTAGAACGTATTGATTTGACCCCATAAGCGGGACCACCACCCAATAGCATGTTGCCACCAGAAGCAGAACCCCGTATTTCTTCCAGAGAATCTCCTAATTGTTCCAGAACAACTAGAAAGAGATTCTTTAACCAGAAAGGATTCAGTTCAGATGTAGGATACCTATCCAGAAGTTTTCGAAATTCCATCATGTATGATGGAATCATCAAAGATTTGATCTTTTCTAACTCTGTCTGTAACTCACTAGAGGCTCGGGAAACAAAGAGAAGATGTATACGAACGAGATATCCAGCAACAAGAAGAAGGAAAAAGATGGAATAGAGGAACTCCCGAGCATTTGTTGATCTCAGATGTGCCCATATCAATGGAACGGGTGACTCATTATTTCGATGAATCATTTCTTCGGACAGAAGAAGATTCTGTAAACATTGACTCGAAATCTCACTTATCAGAGTCCATTGTGGAAGAATCTTCTGAGGAATTGGCCGTGATATATCTGATCCATGCATCATATCATGAAAAATGGATACAAATTTTTGACTACTACTCAGTATCGGCAATGGGTCTGAAAGAGTATCTAAAAGGGTGAAATTGAGATATTTGCACCCTGTCGAAGTAAGGAACCATGGCATATATGTTTGGAACAGATTCCATTTTGAGAGATTTGAAAAAGCACTATCTCGTTGAAAGGTTCTATACATCTGCCCTTTCTCAACGCATTTATTTAGACAAAGACTCCGTTTTTTCCTCTTTCCGGATGGTAAATACTTCTCAGAACATGGAGTGTGAATCAAACCCATGTTTGAATTGAAACTGAGATACTGATGCAAGTTATTCCCTTCTGAATCAGATAGATTCATATCTGAAAGAGGCTGACAATAAGTTTTTTCCAAATTGACTATTTGTTCCTCTGTTAGAGGTGTTGCAGAAATGTCTGCGATCGAGTAAATAGCTCCACGAACGAATGGATCGGATCGAATTGGAAAATGGAAAGATTTGTACAATTTGTACAAGTTATACGTTTCATCACCACTTTGTGGGAAATCGTTAGGTATGAAGATGTCAGATACCTGTGACTCAATTGGTGAAATAGTCTCTCTCTCCAAAAAAAGGTATTTTTTTTTACCGACGCACAAAGAAAAGATTTTGTTGCGAATGGACAAGATATTGAGGAATTGTCCATATGTAAGATCATAATTATTGATACGGGTCTTTTCCACATCAAAGGGGAATCTTTTGTTACAATAGAACCAGAAGTGATGTGGATCATTCAAGAATCGAAGTCGATTTGCTTTATAAAAAGAAGATATCAATGAACTTCTATTAAATGGTTTCACGGGATTCAGCCAATTGTCTCGATCGTGGGATATCATTGAGAAATAGGAATCCTTGTTATCAAAGGATTTCCTGCGATTCTTTCTAGTATGGAATGAGTCAATCATCCGCTTTGGTATCTTTTTGAACAAAAATGGTAATATTGTTCCTCCATTGATCAAGAATTTCGGTCTTTGGGAAGTATCATGATCATCCAATAAGAAGGGTTTCAATTTCTTCAAATGAACAATTTGAACACCTATGGATTCTAACAACTGATTGCAGAGTTGATCATTCGGACCTTTCAATTCATAGATGTGGATCTCGGACCTATGAATGGGGATATTCCCGATATTCACAAAGAAAAAGGGAAGTGACTTGGACAAAAAGAAACGAAGTGACTTAGACAAATCTTCTTTGTCGATAGCCTCGGACCAATCAATCGAATATTTATTAATACGTAATCGATCGAACACTACTTGCACTACTTGAAAAGGATTCTTCTGTTCAGAAACGAAATGTTCCAAATGTTCCTGGAAATTCTTGCTCCCATTGGACCATTTGTATCTATATGCATCAGGATCCCGATTCATGGATCTCTCAGTTCGAGAAATAAGAGGATCAAACCATTTCTTCTGACTCTTTTTCAAATTCGATAAATGTTGGTTGATCGTATATTTCATTATAGTTATATGATTCAGAGTATCATTTCCTATTTGATCCCTTTGAATTCCATATTCGAAGTTGCGATCGGATCTATTCATTAAAAAGAATCGATTCGATACATTTCTTCCATAGGTGCTATATTGGATTTGAATCAGATTTCGGATCAATCTATATTGATTGACTGCCTCCATTATGTTGTTGCTAGCAAATACCGCTGTTTTTAGTTTGGGATCTTCCAACTCATTCCCGCAGTAGATCCGGACCGATTTTTTTCTGATCCTTCGAGAAAAAGATTCATTCTCCTCATAAAAAAGAGGAGGTAGAACCGATAAAGATTTCTTTTTCGATTCATCTCTGGAGTTGAATACCTCATTCAAGAATTTTTTTTGATCCAACCCGTAGGAATCAATAGAAAAGGCAAATCCCCTATGAAACACCAGATCCGGCTCGGTTATTGATAGAGTGAATAGATCCGCCATTTCTGGGAATCTCTCTTCTGATTCAAAAAAATCGTGGCGTAACGCGTACCCCCCTTTGTTCCGGTCATGGAATAGATGAAAGAAATCAAAAAATGGATTTTTGTTCAAGAATGAAATCTTATTGGAACTGTCCATATCCGGTTCATCCTTCGGAACCAGATCACATCCCGGATCTGGTTCCGAAGGATGAATTGAGACGGTATCTTGTAAATACGTAATTATCTTGAATATATCAACCATTTCTTTATTTTCCGCTCGCCTGGAAGGGACAAAAGAAACATCTTGTTTTTTCTTCAACAATTTATGATCTCTAGTGGACCTCTCAGTAGGATTCGAACCCAGATGAAGTTCTGACCATCTGTCAGAGAAAAAAGAACGAATTGATCTTGTAGGATTCCCAATAAATTCTTCGATTTCTTCCGGAAGCAGATGATTATTCATCCGCTTCTCAGGTTCCGTGAATAGCCAGGACATGGAGGAAGATCCAAAAAGGCATTTCGGGAATCGATCTGATTCTATCTCTGTTCGTTCCGTTTGAAGAAAGGAAGGATCCCAAAGAATCGATCTCTCTTTTAGTTGCTGAATCTCTGTTTGATCGATCAATGTGTTATATTCTGAATTCTCATTTCTAACGGAATCGAAATGATCTCTGGATTGATCAGAAGAAGATCCTTTCCATTGGCTAGAATCCTTTACTTGAACGAAAATAGATCTTGTGGAATCATATTGAATATTTGACAATACATTCCGTACCTTGCTAAAAAACCGATCCTTGTTTACCAACCACACATTGTCTAACCAAATCCAATTCTCTCTCGAGACGTTCCTCAAAAAATTCGATTCGTGCTGATTCTTCCCCCAACTAACGAAGAGATCTTGGCGGAATTGCCACATATTAAATTGAGCACAATTTTGCAAAGAAATACCCCACTTGTTTCTCGAGAAGAGATGGGAAACATGCTCAATATCATTGGATTGCATAGTTGCCCCAGCTCCTTGTTGTTTGAAGAAACTCTCCCCCTGAATTGGTCTTTTTTCACGAAAAGCAGACATGAGATAACAAATCAAGTCTTTCCCTAAGATTTCGAATAGCTGTCCCGAATTCAAGTTGATTATGTTTCGTTTCTTCCTCGGAGAAAGACGATCAAAAAATTCCCAATCATGGTCCTTGCGGATCGGATCATCCGTATAGGATAAAAAAAGAAACTCCAGATATTTGCTATCTTTCTCTTTGAATGAGATCTCAATTCCAGCTACGGTTTCATTAGATATCTGACAACTAGAATCCCTCTTTTTTCCGATCCGGTTCCTCCACCACCGCGAACCCCGGTTAGATTCAGGCATGATACGCTTTTTATTTATTGGGATAACCCAAGTACTCTCTTGCGGATCCATGAAACAACTCTCAGAAATCTTTTTCCCTTTTGGAAGATACAGGAGCGAAACAATCAACCTATTTCTATTGGAAGACCAAAAAGATTCTTCCAATGTCTCCTTTCTGGGTCCAATGGAATTCATAGGTATAGGAAGAAGCCCCATCAAATAGAGATTTTTTCTTTCGACCATCTTTCGATTGTTAATACGAGATATAAGGACCACTACTACAAGCAGTACTACACCTTTGGTCGTGAAATATCGATTGCTTGTTGCACCCTGTGAATCACGTGAAAGTAGGATACTCCAAATTCGGGGGTCAAAGAGTTTCATAAAACGTTCTTGGTGGAAAAAAATGTGAGTGAAAGATCCCACTGAATCGAATTTGATCCATGAATCTAAGAAATAGTGATAATTCTTGATCTCTCTCAATTCGAATATCCAGGATTTGAATTGATGTCGTTTCATTGATTCCTCCTAAAGATTTCATTTCAATTGGAATTTGGTTATTCACGATGTACGATGATCCCCGTTAAGCATCCATGGCTGAATGGTTAAAGCGCCCAACTCATAATTGGCAAATTCGTAGGTTCAATTCCTGCTGGATGCACGCCAATGGGAACATTCAATAAGTCTATTGGAATTGGCTCTGTATCAATGGAATCTCATCATCCATACATAGCGAATTGGTATGGTATATTCATACCATAACATATGAACAGTAAGAACTAGAATTCTTATCGATACTGGAACTCATAGGGAATAAAATGGATTTATGGATGGAATCAAATATGCAGTATTTACAGAAAAAAGTATTCGGTTATTGGGGAACAATCAATATACTTCTAATGTCGAATCAGGATCAACTAGGACAGAAATAAAGCATTGGGTCGAACTCTTCTTTGGTGTCAAGGTAATAGCTATGAATAGTCATCGACTCCCGGGAAAGGGTAGAAGGATGGGACCTATTATGGGACATACAATGCATTACAGACGTATGATCATTACGCTTCAACCGGGTTATTCTATTCCACCTCTTATAGAGAAAATAACTTAAAGCAAAAGACTTAATAACACGGCAATACATTTATACAAAACTTCTACCCCGAGCACACGCAATGGAGCCGTAGACAGTCAAGTGAAATCCAATCCACGAAATAATTTGATCTATGGACAGCATCGTTGTGGTAAAGGTCGTAATGCCAGAGGGATCATTACCGCAGGGCATAGAGGGGGAGGTCATAAGCGTCTATACCGTAAAATCGACTTTCGACGGAATGAAAAAGAGATATCTGGTAGAATCGTAACCATAGAATATGACCCTAATCGAAATGCATACATTTGTCTCATACACTATGGGGATGGTGAGAAGAGATATATTTTACATCCCAGAGGGGCTATAATTGGAGATACCATTGTTTCTGGTACAGAAGTTCCTATATCAATGGGAAATGCCCTACCTTTGAGTGCGGTTTGAACTATTGATTTACGTAATTGGAAGTAACCAATTAGGTTTACGACGAAACCTAGAAATCGATCACTGATCCAATTGGAGTACCTCTACGGGATAGACCTCAACAGAAAACTGTTGAGTAACGGCAGCAAGTGATTGAGTTCAGTAGTTCCTCATAGAAAATTATTGACTCTAGAGATATGGTAATATGGAGAAGACAAAATTGTTTGAAGCGCGCACAGAACCGGAAGCGCCCCTTGTTTCAAAGAGAGGAGGACGGGTTATTCACATTTCATTTGATGGTCAGAGGCGAATTGAAAGCTAAGCAGTGGTAATTAGAAAGACCCCCCGGGGAAAAATAGAGATGTCTCCTACGTTACCCGTAATATGTGGAAGTATCGACGTAATTTCATAGAGTCATCCGGTCTGAATGCTACATGAAGAACATAAGCCAGATGACGGAACGGGGAGACCTAGGATGTAGAAGATCATAACATGAGTGATTCGGCAGATTTGGATTCCTATATATCCACTCATGTGGTACTTCATCATATAAGATCCATCTGTCTAGATATCATCATATACATCTAGAAAGCCGTATGCTTTGGAAGAAGCTTGTACAGTTTGGGAAGGGGTTTTTATTGATTTTATTGATAAAAAAGAAGAATCTACTTCAACCGATATGCCCTTAGGCACGGCCATACATAACATAGAAATCACACTTGGAAAGGGTGGACAATTAGCTAGAGCAGCAGGCGCTGTAGCGAAACTGATTGCAAAAGAGGGTAAATCGGCCACATTAAGATTACCATCTGGGGAGGTCCGTTTGATATCCAAAAACTGCTTAGCAACAGTCGGACAAGTGGGTAATGTTGGGGTGAACCAAAAAAGTTTGGGTAGAGCTGGATCTAAGTGTTGGCTAGGTAAGCGTCCTGTAGTAAGAGGAGTAGTTATGAACCCTGTAGACCATCCCCATGGGGGCGGTGAAGGGAGAGCCCCAATTGGTAGAAAAAAACCCACAACCCCTTGGGGTTATCCTGCGCTTGGAAGAAGAAGTAGGAAAAGGAACAAATATAGTGATAGTTTTATTCTTCGTCGCCGTAAATAGGAACATTGAAAATCGAATTTTTGGAATTGGAAATAATGTGATGGGCGAACGACGGGAATTGAACCCGCGCATGGTGGATTCACAATCCACTGCCTTGATCCACTTGGCTACATCCGCCCCTTCCCTTATCTAGCTAAAGGATTTTCTCTTTTTTCCATTCATCATTATACTTCAGATTAAGATAGAGATATTGGACATAGAATGCCAATTTTAAAAATGTAAAAAAAGGAGTAATCAGCCGTGACACGTTCACTAAAAAAAAATCCTTTTGTAGCTAATCATTTATCGGGAAGAATTGAAAAACTCAACAGGAGGGAGGAGAAAGAAATCATAGTGACTTGGTCTCGGGCATCTACCATTATACCCACAATGATTGGCCATACAATCGCTATTCATAATGGAAAGGAACATTTACCTATTTATATCACAGATCGTATGGTCGGTCACAAATTGGGAGAATTCGCACCTA

>Styloglossum_lyroglossa

GAACTTTTATGATAAAGAACGAAAATTCGGATAGAGAAGCAAAAGTTTTAGCTCAACATATATGTATGTCTGTTTTCAAAGCACGAAGAGTAATTGATCAGATTCCAACAAGATAGCAATCCCCCAAGATTTTGGGGGATTGCTATCTTCAAAAATTCATATCATATACTATCATATACATATGTACATATGTATACAAAAGTATTATCCATTTATAGATGGAACTTCCACAGAAGCTAGATCTAGAGGGAAGTTGTGTGCATTACGTTCATGCATTACTTCCATACCAAGATTAGCACGATTTATGATATCAGCCCAAGTGTTAATAACACGACCTTGACTGTCAACTACAGATTGGTTAAAATTGAAACCGTTCAGGTTGAACGCCATAGTGCTAATACCCAAAGCAGTGAACCAGATACCCACTACTGGCCAAGCAGCCAGGAAGAAATGTAGGGAACGGGAATTGTTGAAACTAGCATATTGGAAGATCAATCGGCCAAAATAACCATGAGCAGCTACAATATTATAAGTTTCTTCCTCTTGACCGAATCTGTAACCTTCATTAGCAGACTCGTTTTCAGTGGTTTCCCTGATTAAACTAGAAGTTACCAAAGAACCATGCATAGCACTGAATAGGGAGCCGCCGAATACACCAGCTACGCCTAACATGTGAAATGGATGCATAAGAATGTTGTGCTCTGCCTGGAATACAATCATGAAGTTGAAAGTACCAGATATTCCTAAAGGCATACCATCAGAGAAACTTCCTTGACCGACAGGGTAGATCAAGAAAACAGCTGTAGCAGCCGCAACAGGAGCTGAATATGCAACAGCAATCCAGGGGCGCATACCCAGACGGAAACTAAGTTCCCACTCACGACCCATGTAACAAGCTACACCAAGTAAAAAGTGTAGAACAATCAGTTCATAAGGACCGCCATTGTATAACCACTCATCAACAGATGCTGCTTCCCATATTGGGTAAAAATGCAAACCTATAGCTGCGGAAGTAGGAATAATGGCACCTGATATAATATTGTTTCCATAAAGTAGAGATCCAGAAACAGGTTCACGAATACCATCAATATCGACTGGAGGGGCAGCAATGAAGGCAATGATAAATACAGAAGTTGCGGTCAATAAAGTAGGGATCATCAAAACACCAAACCATCCAATGTAAAGACGGTTTTCAGTACTGGTAATCCAGTTGCAGAAGCGACCCCATAGGCTTGTACTTTCGCGTCTCTCTAAAATTGCAGTCATGGTAAGATCTTGGTTTATTCCATTTTCAAGGACTCCCAAGCACACGTATTAACTATAACTAGAGATGAGATAGATAATGAAAGGCTTGTTATTTCACAGTATAATATGTCTTATATGCCAATGTCAACCAATCAAAATGAATATCTATAGGAATGACCAAACCTATCAGAACAATTTGAAATAAAATAAAGAGTTCAAATAAAATTAAAAAAAAAAAGAAATATTCTTTTTTTTTACTTTGGTATGGGTTGCCCGGGACTTGAACCCGGAACTAGTCGGATGGAATAGATAATTTTTCCTTTTTGCTATAGAAGAAAAAAATCCCTCCCCAAACCGTGCTTGCAGTTTTCATTGCACACGACTTTCCCTATGTATATATCAAAAAGAAAAAATAATTAAATATCAAGAAGAAAGTCTAATAATTTTGACTACTTCGCGAATTTCACCACAAATATGATAATATATACGATATATCAGCATTTCAGAATACAAATTCATGAATCTTTTTTTTTTTATGAAAATGAAAATGAAAAAGTCTCATGAACAATCATGAATTATCCACCAGATCATTGATACGGATAATGTCCAAATACCAAATACGTTCTCTATGTAATCCGTGTAAAAGAAAAGGGATTTTTTGGAGGAAGATTAAAGAAAGAGATTGTCCTTCTTCCAAAAAAAATTCTTCTAAGAATCCCGAACCTAATCTTCGCATAAAAGTGCGTACTGTACTTTTATGTTTACGAGCCAAAGTTCTAGCACACGAAAGTCGAAGTATATACTTTATACGATACAAAACCTGTTTCTTTGAGGATCCGCTATGATAACGACAAAGATTTCTAGATATCTGACAAAATCGATCAAGAATATCAGAATCCGATAAATCGGTCCAGATCGGTTTACTAATAGGATGACCCAATACAGTACAAAATTGAGCTTTCGACAATGATCCAATAAGAGAAATAACTGGGGCTATGGTATCTAATTTCTTAGTCAGAGTATTTATTAGAAATGAATTCTCTAGCATTTGATTTCTTACTATCAAAGGATTTTTTAGTACACTTGAAAAATACCCCAGAAAAGAGAAGGAATAATTGGGTAATTGTTTTATATGGATCCTATAAGGTTGAAACCAAAAGTGAAAATAAGATTCCCAAAAATTTACAAGATGAAATTTCCATTTCTTCATCAGAAGAAAAGTTCCTTTTGAAGCCAGAATTGCTTTTCCTTGATATCGAACATAATGTATGAAAGTATCTTTGAGGAACCATAGGATCCTCTGAAAAAAATTACAACATACGACTATAAGATATTCTATTTTTCCATAGAAATGTGTTCGCTCAAGAAAAACTCCAGAAGATATTGATCGTAAATAAGAAGACTGTTTACGAAGAAACAGGAATAGATATTCGCATTCATATACATAAGAATTATGTAGGAACCAAAAGAATCTTTTCTTTCTTTTTGAAAAGACGTAAATGGATTTCTTTGAAGAAATGATACTATTCAAATTATGATATTTGTGGAAAAAGAATCGCAATAAATGCAAAGAAGGAACATCTTTGATCCAGCATTGAAGGATTTGAACCAAGATTTCCAGATGAATGGGATGAGGTATTAGTAGATCTGACACATAATTTAAATGTGATAATTTATCCTCTAAAAAGGGAAATATTGAATGAATAGATCGTAAATTCTGAGATTTTGGTATTCTTTTTTCTTCAAGGGAAGATACTAATCGCGACGAGAATGGAATTTCCAGAATGACTCCAAAACCTTCTGATACCTTTTGAGAAGAAAAATGAGAAGAAAAAGAATTCTTGTGCCCCCCCAAAAATCCATTTTCAGAATCATTCACCGAAGAAATCAAAGATTTCTGTTGATACATTCGAGTAATTAAACGTTTCACAAGTACTAAACTAGATTTATTGTCATAACCGAGAATTTCCACAGGTTCGTAAAAAATAAAACTATTGAAGCTATGATAATGAGCAAGTGAGTAAATATACTCCTGAAGGAGTAGTGGATATAGGAAGTTTTGTTGCCGAAATCTATCTTTTTTCAATCTAAATATCCTTGTAATTCTGCCATTTAAATACATTTCTACTGTAACCAAAAAAGGGAGACACTTCTTGTGTTAGGAAATGATACATAGTGCAATATGGTCAGAACGGCGTATACATATGTTGTATGTAGTATATTGTTATATTGTTTCTCTTATACTAAAATAGTATAACTAGAATAAACTATAATAGAAAATAATTAGACTATATAATTCTAATAATATAGTCTAGTATTCTAGTATTATTATATACTATGCACTGTCTATACTTTTACTTTCAATAATATATACTTTTATACATGTAAAAAACATAATGATAATCTAATGAAGTAAAAGATTATATAAAAGTAAGAACAAATAAAGAATATATACCCCGGAGACAGAGAGCCCAATCTACAGATCTTTTTCCTTTCCCTTTCTATCCAATTTTGTTTTCTTTTACTTGTTAATATAAGTAGAATAAAAATAAAAGAAATAAATAAAATAACAATAAGAAAAAGGGGTAAAAATCTTTTATTTTTGCAACCCAATCACTCTTCTGACTTTGGAAAAAAATCCCTTTTTTATCACTATACTATTCCTTCTACACATCCGTCTTCAATCCACAATAAAGAATAATTAGGATTAATAAAAAAAAGAATCAATGGTCCATTCACAATTCACAAGAGAACCTTTTCCCACATCAGGCACTAATCTATTTTTAACGCATAATTAGTAATTTGATCGGGTAATCATTCAAATTAAGAAGGGAAGCTCGTTGCTTTTTTGTCTTACTCGAATTGGAGCCATAAGGCTCTATCCATTTATTCACTAGACCCAAAATACTTTACCAATTGTTATCAAAAGAAAAACTCTCGTTCTATTTCTATTATGAGTACTAGAAATCTTCTTTTTCTATGATTTTATTATTCTTTTTTATATTTTTCTATTCTTTTTACGACATGCTTTTTTTTCCATTCATTACCTTTCAGGATCAGTCGCAGTCTTATAAACTCTACCAATAGTCTAGACGAATTCCTTCATCCAAATGTGTAAAAGATCATAGTCGCAATTAAAAGCCGAGTACTCTACCGTTGAGTTAGCAACCCGGATCAATATGAAGTGTAGATATGATCGAAATAAAAAATCCAGCAAACTCATTCATTTTACTAAAGTGAAGGAAAGAGCCGATAGGGAATGGGGATAAAAAGATCTATTTATATACGATCAAATTATATTTGTTTGATACGCCATTGTCAATATGAATTGTGAATATGAATGGACTTTTTATCAAACAAATTTCAAGAAATTCTATAGAAATTTGTGTTGGATTAGCATAATATAAAGAATAAAACTTTGAGCGGAAAAAGAATAAGGAAAAGGTAAAGATAGAAAAAATAGCGGCTTTATTTAATCTAAAAAAGAAAGGTACAATACAAATACAAGAAGAAAGATTACCCCCCTTGTTTGGGGGGTTCCACAAAAAGAAGAAAGAAAAAGAAGAATAAAATAAGTATGAATAGAACAAGAAAAGAAAAAACTTTGAATAAAGATTTACACAAAGATAGGTACTAGTTACCCTATCCTTTTTTTATTCATGATTCTTGTTTTTCCTTTCTTTTTTTATCAAAAGAAACTCGAAATTCTTTAAAGAATTCTGCCTTCCTTAAAATATCATAAACAGTTCCTGTGGGTTGAGCACCTTTTTCAAGGAAATATAAAATAGCAGGAACATTTGAATAAGTTTGATTCTTTATCGGATCATAAAAACCCACTTTTCGAAGATCTCTTCCTTCTCTTTGGGATCGAACATCAATTGCAACGATTCGATAGATGGCTCATTGGGATAGATGTAGATAAAAGATGCCCCCCTAGAAACGTATAGGAGGTTTTCTCCTCATACGGCTCAAGAAAAAATGATTCTAATTTCTAGAATTTCTCTTTCTATCTATCTACTATTATCTATTACTATTATCTATATAGATAGAAATTATCTATATAGATAGAAAGAGAAATGGATCCATAAAGAATTAGACTGTAATTTGAGCCTTTTTTTCCTTCTTTACAGAAAAAGAAATTTCATTTGTACTCCTAACTCAAGTTGGGTAGTTTTGAAAGAGTTCGAAAGGAAATCCTTATAATTTCTTGAGCTGTCTCTAACCTCTTTTTTTGTCTTCTTTCGGATATATTTTTTTTACTCATTCTGATCCGATTGTTGAGACAAGTGAAAATAGTGTTTCCTTGTTCCGGAATTTGTTGTCTTTGCTTTGCGCTTTGTTAAATCATTGGGTTTAGACATTACTTCAGTGATCCTTACTCCTTTTCAAAAAGGCAGCAACATACCTTTTGTTTTTTGTTCTTTCTATGGAAAGAAAAGGAAAAAATAATACGAAAGATTGATTCCTTTGTGATACACTCTTGATTCAAACAGTTTGAAAAATTCGACAAATTTGATTTTTTCATTTCAAACTTGTTCATTTTTGAACCTCTCCATTTATCTATATTTAGATATTGATGATATATTTACAAAGTTGGTCTAACTTATTGATTGTCACTAACCCTAGATTATTCCCCCGATAAATGGAATCAATCATTTTTGCTCGAGCTCCATTATATGCTATACCTTTATATACCATTAGTATCTTTATTTAGTTATTTAGCAACCCAAGACAAATGAAATTAGGTTCCTAGCAGAACAAATTATGTCGAGACAAGAGCATCTTCATTCGTATAGAAAGAGAAGATGGTGGATGTAAGAATCCACAGCCAATCATGTCCTTCAAGTCGCACGTTGCTTTCTACCACATCGTTTCAAACGAAGTTTTACCATAACATATCCCTCTCATTTTATTTTAATTTGGAACCGTATGCAATTGATTCAATATGGAATCATGAATAGTCATTGGCTGAACCAATCGATACATAATAATCTACACTTATAGATAAGTGTTATCCGGAAAGGATTTCACTTAAAAATACCCCATAATTTTCTTATATGAATAATATCACATGAAAAAAAAATACCCATGAATGGATAAAAGTTTTTAGCCACTTTAACTAAATTTCACTAAATATTTCATTATTAGAATAATAAGATAATCTGAAATAATAATATTATATAATATTATTAAGAAAAATATACAAAAATATACAATTACAATATATACAATACAATATATATTAGTATATTAGTATTAGTATATTAGAATATATATTAGAATTAGAATTCTATTTTATTTTCTTTCTATTTTATACTCTTATGTAATATATGTAATAATTATGTATTTATGTATGAATATATTTTCATATGAATATATTAATTATGAATATTTTCTCATTTTTTCTTTATGAAATATGATTTTTCTAATATTATGATTTACTTATTATTTACCATCTCCAATTGAATCTTATTTTCATTTCATTGAAAACAGAGAGATAGTTTGAGAATAAAGTTTCTTTGTTTTCATTATTATGGAGTAGAAAAAGTCTCGTGTAAGGTAAGATCAAAGAGAAAATAAGAATCCTCGTATTCTTATCTTTTCGCATCCATCTCCATCATATAAAACAAATAATCGTTAACAAAAGTAATCACGAATATTTCTATTTAAGAATAAAATACTTTAGTAAAAAAGTAAAAAAAAAAAGATATGATTCTTAGAATTCAGATTGGATAACATTGTATCCAACATGAAACATAAAATTGTTGAATTAAAATTTCAATAGAGAAGAATCTTTCGTTTCTAATGCAAACGATCTGGGACGGAAGGATTCGAACCTCCGAGTAACGGGACCAAAACCCGTTGCCTTACCGCTTGGCCACGCCCCATTTTGATTTGGTCTAAGAAAAAATAAGATAAATATTGGTTATTCGTCAATAACCAACTATAGGACATGTTGTCGCTAGGATTCAACACAATAGATATAGAATCAAAAAGATTAATTGATCATTACTTAGAATTCAATTAAGATATTGTATGAAAATAGAATTCCTTGTATTCTTATTTGATTGGAAAATGTAAGGAAGGATTCTTGATTGGGGAGAAGTCAAAGAAAAATAAGAATTTATTTCTTTTATCTGCATCTGCCTTTTTATTTGAAAATTTTCCTCAGAAAAACAACTCAATCCAATCTGATAATTTCTTATCATTTCAATTTAATTTGAATTTTTAAGAACAAGAAAAGAATATTTGTTATGTTTAATATCTTTAGTTTAATCTGTATCTGTCTTAATTATACCCTTTATTCGAGTAGTTTTTTCTTCGCCAAATTGCCCGAGGCTTATGCCTTTTTCAATCCAATCGTAGACGTTATGCCGGTTATCCCTGTACTCTTTTTTCTCTTAGCCTTTGTTTGGCAAGCTGCTGTAAGTTTTCGATAAAAATGAAATCTCTATTCAATTCATAATTTCTTCGATAAAAAAAAAGATGAGATTTTTATTCTGAAAATCAATAAGATCATAAATAAAATTCAGATAAGTCTGGACATTAGGAACCTCGGTTCAAAAAGTGAAATTCTGGTATTTTATATTTTATATTGAAATTTAATTTGAATAAGGGAAGGGGGGTGATGATCTTTATCTTTAATCAGCTAATCAGCTTATTTCTTCTTTTGTTCTGACCTTTCCTTTATAAAATCCCATACAATATCTAATTATAGATATGGATATGACAAGAAATTTTCGGTAACGAAAAAATACGAATCTTATTACATTAGAAAGAAATTCGTTAAAAGAAATTTCAAAAAAACTTCCTTTTTTTTTCATCTTTAGAGCGTCATATCAAAATAGTGTCTAGTGTGTGTCGTAAAATAGGTGATCTATTTCCTTAAAAAAAAATGATCTTATCTTATCTTGGAGATTTGTAATGCTTACTCTTAAACTATTCGTTTACACAGTAGTAATATTCTTTGTTTCTCTCTTCATCTTCGGATTCTTATCTAATGATCCGGGGCGTAATCCCGGGCGTGAAGAATAAAAAAAGATATGAGATTTGTTTTTACTTTTTCCTTAATTTTTTCATAGGTAAATGTAAAAAGAAATGGAATAGATGCTAGATAAAGATAAAAGATTCATAAAATAAAATAATCGAAATTTATTTCAATTCTAAAATCTCAAGTGATCAGGGGGGTCGGAGAGAGAGGGATTCGAACCCTCGGTACAAATAATTCGTACAACGGATTAGCAATCCGACGCTTTAGTCCACTCAGCCATCTCTCCCCATTCAAAATTGGAAATTTATCTTTAACACGTGAAGTCAAGATTGAGAACAATTTTTATTTTCCTTCAATGATTCGAAACAGATTTTTCCAATAGAAAGAATACCTTACTTCTTTTATTTTGTACAAAAGGTTCATTTCCCCGGCCTGGTTAAGTAGAAGTACTGGCCGGGCCAGTACTTCTTTTGTTCCGACGAATAAAAATTGTTATTGAAACAAGAAGAGTAATTTTTATTCCCATTCCTAATTATTAGGAATTATATAAGATTCTAATAGATAGATTATCTTAGAGATTCTTTAAAAAATTATCTAGATCTAGATTAATGATTGATATAGAATATTCTATATATTGAAATTAAAATATTAAATATTAGAGATTATATATCTATTCTTAGTTCTTAGTATATTAGTATTAATATATTTAATATATTATATACTATAATATATTATAGTACCTTATATAGTAATTCTAGTAAATTAGAGTATAAATGAGTATAAATTCTAATATTTAGTCTTTTCTAGTAAAAGTGTTCTACTATATTCTACTATAATAGTATTATAGTATCTAGTAATATAGTACTAATCGTCGTCTTTATTTTATTATTCTTAAGTATAAAAAGTATAAAATTTGGAAATTCATTAATGAAAAACAAATTTCATTATAAATGAAAGTTGAAGTTCAGTATTATATTCATCTTAATAATTCACTTAAGAAGTCTTAATAAGAAAGAAGTATTAAGAAAGAAAAAAAATATATAATATATCTTATAATATTAGAATATCTTATAAGAGAAATAATATCAAATAGAAAACACATATTTATAAAATGAGACTATAAATTATTTACTTGTTCAGAGCAAAGTACAAGCTTGAAAGTTTGAGGCCCAATTTACCCGAATTCAGAAAATCTGGCGGTTCAAGTGAAGGGAGGTTTCAAAAAAAAATTTAGTACACTATTTAAATTTGACTAAACTTTTTGCTATTCACCTATTTTTTTTTTCAAGTTTTCAATTCCGCGCCGCAGTGGAACAAAATACGTGTTAATGCTGAAATTTTCATGATTCTGATGCTATCTTGACTGCATCTGATTACTTTGTTGGACAAATGGTACATTCATATCCAATAATGAATATATAGCGGGTATAGTTTAGTGGTAAAAGTGTGATTCGTTCTATTAACAACTTCAATAGTTAAGGGGTCTTTCCATTTTTTTCATATTTCATATTCCGATCAAAAACTTTATTTCTTAAAAAGATTTAATCCTTTAACCCTCAATAGGACATTTGAGGAAAGAATATACATTCTCACGATTTCTATCCAAAAGGCAATCAGAATTGTAATAAAAAAATTGGATTATGGAGTCGAGAAGCATAATTTTTTTGATTGGTTCAATTCTTCCAATTGAATGAGTATGAATAAAGGATCTATGGATGATAATACAAAACTTTCAATCGTAACTAAATCTTCAATTTTTGCGCTGAAAAAGGGGGAGATTGAGGCCAAATAGCTAGGAAATGATGGTTTTGGTTTACTAGAACCCTCGGCTTCTTGTTTCAGCTCGGTAGAAACAAAATTATTTTCCTTAGGATCCCGCGAGTAAAAAAGAAATAGGGAACGAAGTAACTAGACTAGAAAGATTTGATAGAATCCCCCTCTTCTAGAGGGATCATCTAAAAAGCAAGTAGCTTTAGATGCATTCGTAAAAAAAGCTGACATAGATGTTATGGATCTCATTTTTTCTCTGGTGGGAATACATAGATCTTCCATAAAGGAGCCGAATGAAACCAAAATCTCATGTTCGGTTTTGAATTAGAGATGTTAAAAATAATCAACTAACGTCGACTATAACCCCTAGCCTTCCAAGCTAACGATGCGGGTTCGATTCCCGCTACCCGCTATATATATTCATTCCTTAACTTCATATGATATAAAGATGCATTATCCTTTTCTTTTTGATATGCATCCTTCTTTTTATTGTATTCCCTAAATCCGTCTAATTCTTTTTTCTTTTTATGAACGAAAATAGGAATGAAGGGCGTCCATTGTCTAATGGATAGGACAGAGGTCTTCTAAACCTTTGGTATAGGTTCAAATCCTATTGGACGCAATTTATTTCTATATATCTATTCTAGATAGAGAATAGAAAAAAAAAAGAAGAACTATATTGTAGAAAGGATAAAGGCCCTTTTTTGAATGATTCGAATCAAAAATCTTTATCAAGGATTTCTATTTATTAAGAATATAATAAAAACCACCCATTTACGTTTATTTTTTTTTATTTTTTTTCCTGAAGTAGAAAGAGTTCGATCTGTTCCTGAATAGCTTCTCTCAAAAGGGTTTCAGCTTCTTCGGTGAATATTTTGGTAGAAGATAGAATTTCTTGGAATTTAGGTTTCTTCTTTGTTAAGTAGGTACGTAACTCAACGAGAAATTTCTTTACCTGTCCAATTTCTAACGCATCAAGATGTCCATTCGTTCCGGTGTAAATAGTAGCTATCTGGTCTTCCACCGCGAGAGGGTCTGATTGGGATTGTTTGAGCAACTCACGTAATCGTTGACCTCTTGCCAATTGATTCTGAGTAGCTTTATCTAGATCAGAAGCAAATTGTGCAAAGGCTTCTAATTCTGCAAATTGAGCTAGTTCCAATTTTGATTTGCCAGCTACTTGTTTCATGGCTTTAATTTGAGCTGCCGATCCTACTCTGGAAACAGAAATACCTACATTAATAGCAGGCCTGATTCCGGCATTGAATAGATCGGCAGATAAGAATATTTGTCCATCTGTAATAGAAATTACATTAGTAGGAATATAAGCCGAAACGTCTCCAGATTGAGTCTCCACTATTGGTAAAGCAGTCATACTTCCTTCACCTAAACGAGAACTTGATTTAGCGGCTCTTTCCAAAAGTCGTGAATGCAAATAAAAAACATCTCCTGGATAAGCTTCACGACCAGGAGGTCTTCTTAATAGAAGAGACATTTGACGATAAGCTTGTGCCTGTTTGGAGAGATCATCATAAATTATTAAAGTATGTCGTTCACGGTACATAAAATATTCAGCCAGAGCCGCTCCTGTATAAGGAGCGAGATATTGTAATGTAGCAGGCGAATCTGCTGTTTCGGCTATCACAATAGTGTATTCCATTGCTCCTCCTTCCTGGAAAGTAGTCACTACCTGAGCCACAGAAGATGCTTTTTGACCAATAGCTACATAAACACATATCACATTTTGTCCTTTTTGATTGAGAATCGTATCTGTGGCTACTGCTGTTTTACCAGTCTGTCTGTCTCCAATAATTAATTCTCGTTGCCCGCGTCCTATAGGGATCATTGAATCAATAGCAATAAGTCCCGTTTGAAGGGGCTCATATACGGAACGTCTCGAAATAATACCTGGGGCAGGAGATTCAATTAACCGAGATTCAGAAGATGAAATTTCACCTCTCCCATCAATAGGTTTAGCTAGAGCATTTATAACACGACCCAAAAAAGCCTCGCTCACAGGGATCTGAGCGATTCTTCCTGTTGCTTTTACAGAACTTCCCTCTTTTATCATCAAACCATCACCCATTAATACAACGCCAACATTATTTGATTCCAAATTCAGAGCAATGCCTATGGTACCCTCTTCAAATTCTATTAATTCACCTGCCATTGCTTCATCAAGACCATGAATACGAGCAATGCCGTCGCCCACTTGAAGTACGGTACCAGTATTCACAATCTTTACTTCTCTATTATATTGTTCAATACGTTCGCGTATAATATTACTAATTTCGTCAGCTCGAAGGGTTCCCATTAGTATCTCTTTTTTATTTTTCGGAAGGAAAGGGAAAAAATAATACCTAAACTCTAAACTAGAGTAAAAAGGCTAATCAGTTATTTCTTCCACGGACCCGAGGATACCAATATTAGCACTGATGGTACGAAAATGTAATTCGCTATTCAAACAACTATTCAAAGTTCCTAGAGCTTTTTGTAAGGCTTGTTGCAAAACTTGTTGTCGGACCTGATTAATTGTTCTTTGTTTTTCAAAAAAAAGAGTTTCATTTTTGTAATTTTCTAATCGTTCCAAACTATTGGAAGTAGCATTAATCAAATTTACTTTTTCTCGTTCTATCTCGGAGTATCCATTCGTTCGATACTCATCTGCTTCGATTTCCACTTTCCGTAATCTAACCCGAGCTCTTTCGAGCTGTTCAATGGCTCTTTTACGTAATTCTTCTGAATTTCGAATAGTACTCAAGATCCTCTGTTTTCGCTTATCTAATAAATCATTTAATGAAAGTAGATTATCTTTCCATTCATTTCACAACTATCATATCTCTTCCCGAACCAAACATGAATCTTTCGATTCATTTGGCTCTCACGCTCAATTGTTTCTTTTATCTTTTCTTTGATTGATTGGCATTCCCCATATCTTTGAACGGAATGAGCCTATCCTCTCTTTTCTGTTCGTATATCGAAACTGATCTAACATCAGAATCTTAGGAGGACTCTTCAGACCAGACAAAAAATAAAAAATTGTCAGCAAAGTTGTTTCTTTATTTGTTCTTTATTTACAACTTATTTCCAAAAAGAAAAAAAAAAGATTTTAAAGAAAAAAGAATTCTATTCTTTCTTCTTTATATGCTATGATAAATTAGATCAAAATTCTATATAGATAATATCTAATTTAATAGAATTTTGAACTTCATTACTTCATTATCATTATTATTAGAATGAGATTTCAATTTTTTTATCCAAAAAATTCTCTTTTTTATACAAATAGAATACATAGGTCGTCGATTCGGCAGTGGATAAAAAAAAGGGAGGGAGATACCCATAATGGTTCAAATAATTTTATCCATATGAGTGTTCTACATCGGATAAATTCCCAATTATTCCTTTTTTATCATCTTTAAACCTTTTTGTTACTAACGTAGCGGTAGAAAGAGTACCATACTATGCTGTGCCTGGACTTCAAACAATTTATCTTTAACCATGTAAAAGACCTCAACATTGTTGGTTGATAGAGAAGAGAATCAAAGTTCATTTATCAATTAGTCACGAAATGCTATGGTTCTTACATATGATCTCTGAATGAAATCCAATTCCGAAGTAATTCGTTGAGATTGTGCACCCTTTGTTCCTATTTCTGCTATAAAAAAGAATAAAGAAAGTGCAGCCGGTGAAATCCAACCTATTCTTGAAATACACAACTCGCACACACTCCCTTTCCAAAAAAGATCAATACACCCAGCACTACGCTTAGATTTATTGGATTTGTTGCTAAAATATCGGTATTAAACTCGAAACTCCCAGCGGATGGCCAGTGCTCCACGGAAACAAAAGAATCGGTTATATTTTTCATAAGCTCTCCCCTTATAGATAGGACTAACAAAGAACAGAGTTCTTTTTGTATCACTCCGCTTATTATTCTTAATGGAATTTGAGAATTCTCAAATTTATTAGTTATAGTTATGTTTTTATTCTACGATGAAATTAAACATTAAACAAAAGGATTTGCAAATAAAAGAGCTAATGCCACGACCAGTCCATAAATTGTTAAAGCTTCCATAAAAGCCAGACTAAGCAATAAAGTACCTCGTATTTTACCCTCTGCTTCTGGTTGTCTCGCAATACCTTCTACGGCTTGGCCCGCAGCAGTACCTTGACCAACCCCAGGTCCGATAGAAGCAAGCCCTACAGCCAATCCAGCAGCAATAACGGAAGCAGCAGAAATAAGTGGATTCATGGTAAGTTCCTCGCACCAAAAAAAGAAATGGTTAATGATACAACCAACCAATGAATTAGTACTTCAATGAATTAGTACTTAATCTACTTATTTTTCTACTTTTTACTTTTTACTACACTTATTTTTTATTTTTTGATCTTTATCACCAAAATCTATCGGGTCGAAGTAGATAAAAGCTCCAAATGGAATTCAGAATATTACTGAATTGTCAGAACTACTTCGATATACCGTTTTTTCTTTCTACCTTATGTAATATGTATACGGTTTTAGTCATTGCGTTTCCTTGTTTAGAAACTATTCTATTCTTTCTCTTTCATTTTTCATTCAATTCACAATCACAGAAAAAATAGAAAAAGGACTGATATGGAAATTCATCTAAATGCAGTGGACTAGAAAAAAAAGAGATAGGGGTAATTACTATCTAACTAGTAAATAACATATACTTTTATTCTTCCATAACGTAAATCACCTGCACTTTTTATTCTATTAAATCGTATTCTGGAACCATTCTTCGAAACATACACAAGGATTTATACTCATAGGCATTACATATACATATATGTAGTAGGAGCCCCAACCCTTCTTTCTCTTCCAAATTTCATCTATCTTTCTTCATATATACATACATGTAGCTATTTGCATTTTATTCTCTAACCTAGTGGATTATATTGAACCCCCCTTGAATTGGGATAAGAAACTATTTCGAAAGTTAGTCAATGATGACCCTCCATGGATTCACCTATATAAGCCGCAGCCAAAGTTGCAAAAATAAGAGCTTGAATACCGCTTGTAAATAATCCAAGAAACATGACAGGTATAGGAACTACTGAAGGTACTAAAGAAACAAGAACAACAACCACTAATTCATCAGCCAATATATTCCCGAAAAGTCGAAAACTAAGAGATAAAGGTTTTGTGAAATCTTCTAGAATATTAATTGGTAAAAGTATTGGAGTTGGTTGAATGTATTTCCCAAAATATCCCAATCCTTTTTTGCTAAGACCCGCATAGAAATATGCCACTGACGTGGGTAAAGCTAAAGCAACAGTAGTATTTATATCATTCGTGGGCGCAGCTAACTCCCCATGAGGTAACTTTATGATTTTCCAAGGTAAAAGAGCACCTGACCAGTTAGAAACAAAAATAAATAGGAACATCGTTCCTATAAAAGGAACCCAAGGACTATACTCCTCTCCAATCTGAGTTTTGCTCAAGTCTTGAATAAATTCAAGGACATATTCGAAGAAATTCTGACCGTCGGTCGGAATGGTTTGTGGATTTCGAACAGCTATGATGACTGAACCTAATAAGATAGCAATTACAACCCAAGAAGTGATAAGTACTTGGGCATGAATTTGTAAACCTCCTATTTGCCAATAGAAATGTTGGCCTACTTCTACACCTGATATATCGTATAATCCTTTGAGTGTTTTAATGGAACATGGTATAAAATTCATATTGTCCTCTAACAGAAATCGAACTTCAAAAAAGGAATTATTTTGATTCAACCATCTCTTTCTCAATTCATCTATGTTCATTCATGAATTTAAGTTATGAATCATATATTTCGGATACCGAGAAATCATATAAAAAAATACCAATCATTTTCTCTTTTAAATATTCTTTAATATTCAAAACATAATTCAAACTCCAAAAGATGCAAACCAAAATAGTAACAATTAAAGAGAGTTCACCAAAAACAAACTAATCTTCTTCTTTCTTCTTCTTAATAATAAGAGTAATGATAAGATATTCAACCATTTTTTATATAACTAGAACGGCCCTCACAAATTGCAGATACTAATTTGGTAAGAATCAATCGAATCGAAGCTATAGCATCATCGTTGGCCGGAATAGAAATATCTGCAAGATCTGGGTCACAATTTGTATCGATTAAACAAATCGTCGGAATACCCAAAATAACACATTCTTGAAGAACCGTATATTCTTCTTGCTGATCAACGATAATTACAATATCGGGCAATCCCGTCATATATTTGATCCCACCCAGATATGTTTGCAAGGTAGATAACTTTCTCTTCAACATTGCTGCATCTCCTTTGGGAAGACGATTCAGTTTCCCCATCTTTTGTTCTGCTCTTAAGTCCCTGAATTTCTGAAGTCTTGTTTCTGTAGTAGACCAATTCGTTAACATACCACCAAGCCATTTTTTATTAACATAATGGCATCGAGCCCTTATTGCTGCTGATGCTACTAAATCCGCTGCTTTCTTTTTGGTGCCAACGATTAAGAATTTTTTTCCCCTACTTGCTGCATCAAAAACTAAATCGCAGGCTTCTGATAAAAAACGAGCAGTTATAGTAAGATTTGTAATATGAATACCTTTACGCTTTGCAGAGATGTAAGGAGCCATTCTAGGATTCCATTTATTAGTACCATGACCAAAATGAACTCCTGCTTCCATCATTTCTTCCAAATTGATGTTCCAATATCGTCTTCCCATTTTCCCACACTTTTTCTTTTTTTTTTCAAAGAAAAGAGATTCATTACCTTGTGAAATAAATAATTGTTCCAACGGAACCTTCTACCAGGATTGATCTTTGATCTACGACCCAAACCATGAATTTCATTCTTTATTTTTGATTTCATTGATTACGAAATCCATAATTGGACCAGAGAGTTAAAGTAAAAAGAATGAACCTCTTGTTAAGAATTAGTAAATTACAAAATTACATTACGTAAATGATTGGTCTGATGTCTCATAGAAAGTGTTTGGGGAATAAAAACAAAATAATTCTCTATGGTGTAACAAAATATCTCTCATTTCCAACTCGAATAGATTCTTCCTTTTGATTTTCAAATGAATGTTTTTGTCTTGCTTTGAACGATGTACTAATTTTTTGAATCCGGTACCAACTGGTATAATCCCCCCCAAAACAACGTTCTCTTTCAGGCCTTTCAACCAATCAATACGACCTCGTAGAGCAGCTTTTGCTAAAACTCGAGCAGTTTCTTGAAAACTCGCTTCGGATATGAAACTTTGAGTATTCAGAGATGACTTCGTTATTCCCAATAAGATTGCCCGATAAAAGATCGCTTCGTCCAAAACGCGCCCTGCTCGCTCTGCTCGCAACAATCCAATAAATTCCCCAGGTAAAAAAACATTAGACATTCCATCTTCTGAAACCAAAACTCTTGATGTTACTTGACGTACAATAATCTCTATATGTCTATTATGGATCTGTACCCCCTGGGATCGATAAACCTTTTGGATCTTATTAACCAAAGAGATACGACTTTGGGCTATGGTTAGTTCAGCTCCAATCAAGAATCCCCAGGGGATCCCAAGAATCCTTCGGATACGTTCGTCCCAACCTTCAACTCTTCTTTCGAGGTTCATCGATATTGAATCAATCGAACGAACTTCTAAGATTTGTTCCACTTTTGGAAGACCTTGCGTTATGTCACCAGATCTCGATTTTTCATATATAAATGTAATTAATGTATTTCCTTCAGAAAAGGTTTCCCCATAATGGCCATGTACAGTTGCTCCTGGAGTGACCAAATAGGGCTTAGCTGATCTTATAACTAAAGAGTCAACATGAACAATGAAAATTTGACCAGATTTTTTTATGCGTGATCCATATTTCAATAGACATACATTTTCACAAAGGAATTGTCCAAGGCTAATTATTGTCCATGCCTCTTCACAAGAATCGTGATGGAGAAAACACCAATTCGAATGGAATGAATTCCAAATGATGTTACTGCATGGATCGGGATTATAAATCCTACTATTTTCATCTAGGAAACAGTATTGAAATGGAAGTACTTGAAGCACTTGGAAAGTCTGTTGAAAATTTTCAAATAAAAAATATTTCTTTAAAAAGACTTGATTATAAGTTCTTAAATAGTAAGATGAACAAAATTTTACTATTTTAGGCACAATAGTACCTAAAGGACCCAACAAATCCCTAATTGGAGTCATGGGATCCGATTCTTTTGTCGCATTATTATATCTCAAAGTATTGAAGGGGCCAATTCGAGAACAATTGGATGATGACAAAATTATGAAAGATTGGCATTCCTTATTTCGATTCAACAACGTACCAAGAGTTCCCTTATGTTGGGGAAATGATTGAATCTTCGCCTTGGAATCAAAAGGATTTCTATGGGTACGATCTAATTCATTATTGGAAATAAATCCTGAACCTGCCGTATCATACCTTTTTTCGGTATACAAAATAGTGGACTTTACTAACTCAATTCGGATGAAATCACGAAGCAGATCATTTGCCCTTATTTCAACAAAAGAAGCATGAGCCTCTTCTTCTATAGAACTCTTTTTTTCTTGGTCCCAAGTCAATACTAAGCAAGTCCGAACTAATTGAATACTTGTGTGAGAAATTCCTCGAATTGACTTGCCATTTCCATAAAGTATATAATTGACAATTCGAAGTTGGACATTATCCTTTTCCTGCAAGAGATCCTGAGAGAAAAGTGTTGCTAAATTTATCCCATCAGCTATTTCATATGTGACTACGGGCCGAACCAAAACAAAATACTTTTTTTTGGTAGGTGTAATCCGTTGGACATAGATCCAATTTTTCAATTTTTTTGATCCTTTAGAATTTTTTTTTTCCATTCCTGGTGGTATCAAAATGCCACTGTGCCTAGATATTTTATCCACCTCTCCAGAAAAATGAATATCTCCAGAAAATATTTTCAGTTCCATACTTTTTTTTTTTCTCTCCACTCGAACTAATCCACCTACTCGACTTCTTGTATTTATATTTAAAGCAAGTCGTGTGTCTACTCCAATGATACTATTGTTCCGGACCATTATGGGCGAAGATCCGGGTAAAATATGCACTTCCTCGGGAATGAAAAAAAATCGATCTACTTTCATTTGCATTTGGTATTTCGGACTAAATTCCTTTGCTCCTCGATACTCAATCAAATCCTCTTTTTTTACGATTGAATCTACTTCGACAATCCCATATTTAGTAATTCCCGAACTGCTTCTTCTGTATCGCGGATCATCAAAATAAGCAAGAATACTATTTCTACGTAAAATACCATTTATAGGTATTTTAATCGAAATACCAAAACAGGGTATTAGTTTCTTTTCTTGTTCTTGATCATATTGTAAGGGAATCACGAATCTATTTCTTCGCTTTTTCGCCAAGGAATTCTCTTGACGAATATAAGTAGAAGGCTCTATAAGATTCCAATGACCCTTGGACATGATTCGATAAGGTTTTGAATAGTCAAAAATTTCCCTATATTTTTTACCAAAAGTATCCAACAATTTCTGTCTTACTTGATCATTAGTTAGTGAGAGATCAAAGATATATCTTTCTTCGAGAGAAAAAGAATTAGCATTCATTTGATCTTGATCCTTGTGGAGTGAAAAAGACACTATATTGGATCTGCATAGACCTCCTGCTAATATCCATAAATGACTTGTTTTTGGTAATAAATGAACATTACTATATGGATATTCGGTCGCATGATAGCCATCAGTACTCCAGTGCATTTCTCCTTCTGACTCAGAATAAATATGTTTTTGAACTCTCTCTTTAAAATGAAAAGTGGACGTTCCGGTACGAATCTCGGCAATCACTTGTTCTGATTCTACATATTGATCATTTTGAACTAAAATCAAACTTTTTGTTGGAATATTCACATTATGTAGAATATCTCGACTCTCAATAGTTACATACAAGTCTATAAAACATAGAAAAGCAGGATGCCCATGACGGGTACGTGTGGGATGAACCAAATCCTCATCAAATTTTATTTTTCCATTAGAGGGAGCTCGTACATGTTCGGCAGTACCACCCGTGAATACTCCGCCGGTATGAAAAGTTCTTAATGTTAGTTGAGTCCCCGGTTCCCCAATTGATTGACCCGCAATAATGCCTACGGCTTCTCCCAACTCGACCAGGTCACCATGAGTAGGACTCCGACCATAACATAATTGACAGATCCAAGATGTACTCCTGCAAGTAAAAGGGGTTCGAATATATATTGGGTTTGCTCGAAAGGTTATGAATCGATTAACAAGTCCAATTCCAATATCTTTATTTCGAGTGGCAATGCATCGTAGACCAATATATATATCGTCTGCTAATACACGACCAATTAGTGTTTGGACAAAAATCTTTTCCGTCATCCCATTTCGAGAACTCACGGAAATACCTCGGATAGTACCACAATCCGTTCTACGTACAAGAATGTGTTGAACTACTTCAACAAGTCTACGCGTGAGATATCCAGCATCTGATGTTCGTACAGCAGTATCTACAACTCCTTTGCGAGCTCCGTAGCAAGAAATTATATATTCTGTCAAAGAAAGCCCTTCCCGTAAATTGCTTTGAATGGGTAAATCAATCATTTGTCCTTGAGGATCCGACATTAATCCTCTCATACCTACTAATTGGTGTACTTGAGATGCATTTCCTCTAGCCCCCGAAAAGGACATTAGATGGACTGGATTAGAGGGATCAGTCATCCGAAAATTAGGATTCATTTCTTGTCTCAAATAGTCACTTGTAGCATACCATATCTCAATGGATTGACGTAATTTTTCTACCACATGTACATTCCCATAATGATGGTTTTTCTCTAAAAGAAAACTTTGCTGTTCAGCGTCTTGAACTAACCATCCTTTAGAAGGTATTGTTAAAAGATCATCAATTCCTAATGAAATAGATGTAGCAGTGGCTCGCTGGAAACCCAAAGCCTTCACTTGATCCAGGATATGTGATGTATATGCCATTCCGAAATGATCTATTAATCTGCTAATAAGTCGTTTCATAGCAGTTCCATCTATCACCTTATTGTGAAAGACCAGATCGGTCCGTTCTGCCATAAGGACCTCCGTATTCTGCTGAGTAAGATTCCACAATGGGCCTGGGTCAGTGATTCGAAAACTTCCTTTCCTAAATTTTAATTCACACAGAAATTCAGAAATTATGATACCGGTGAAATTGGAGAGACCCGAATTCCCACGAGTATGGGCATCACTGATAGAATTTCTTAGTTTTTATATAGAGCATGAGTTAGATAGCCTATGAGTAGGCTCGCCAAAACCCCTGTATGGCTTCTTCTATTTCTCGATAAAAAGAAAGATGACCGACAGTAGTTCGAATGTATATACAACGAATTTCTCTTTTTACACTTCCTATTATTCGATAGTGCTTATAAATCTCATTATAATTACCAAAAGATTCATATTGAACTTCGATGGGAACTTCTCTTGAGCCAACGACGCGTTGGTCTAGTCTCCACCGGAGCCACAAAGGACTATCTAAATGGATTCGTTTCTGCCGATAAGCTCCAAGTGCATCATAAGAACTAGAAAAATAAGGTTCTTTTGTATACTTAAAGTCATTCTTGTAAACTGTTTCCTTTTTATAGTTTCTGCAATTAGAATTATATTGATTATACCTATTTGCACAAATACCTCGGGGATTCCCGATCGTTAATACATAGAGTCCAATAAGCATATCTTGAGTTGGTACGGAAACGGGATCCCCAATAGCTGGAGACAAGAGATTCATATGAGAAAACATAAGTAAACGAGCTTCTGCTTGAGCTTCAAAAGATAAAGGTACGTGAACAGCCATTTGATCCCCATCAAAGTCTGCGTTAAAGCCCTTACAAACTAATGGGTGTAAACAAATAGCACGCCCCTCCACTAAAATGGGTTGGAACGCCTGTATACCTAATCTATGCAGGGTAGGCGCTCTATTCAACAATACAGGATGCCCCTGCATCACTTCTTGAAGGATTTCCCATACAATAGGTTCTTTTTCCCGAATTTTGCTTTTAGCAATCCCTGTGTTAGAAGCAACATCCTGTCTGATTAGACCACGAATTACAAATGTTTGGAAGAGCTCTATTGCTATTTCTCGAGGTAATCCACATTGATGTAATGAAAGCAAAGGACCCACGACAATGACGGAACGCCCCGAATAATCGACCCGTTTACCAAGTAGAGTCTCACGAAATCTTCCTTCTTTACCTTCAATTACATCGGAAAATGACTTGTAAACTTTATTATGACCATCTCTCATGGGTTGTCCGCGGATCCCATTATCAAAAAGTGTATCCACGGCCTCTTGTACCAATTTCTCCTGACACATTACTAATTCCCCTGGTGTAGATCTACTTGTTGCTAATAGATCGGTAAGAGTATTGTTCCGATAAATGACTCTTCTATAGAGTTCATTAATATCCGAACTCATTAGTTTACCCCCGTCTATCTGAATGATTGGTCTCAACTCAGGAGGAAGAACTGGTAATAGGCACAAAACCATCCGTTCTGGGTCTACATTTGTTCGAATAAAATGTTTAGCTAATTCTATGCGCCTAACTAAAAAATCCTTTCTTCTTCTAATTTTTTTATCTTCCCATTCATTTCCTGCGGACTCTTCGTCTCCTAATTCCTTCCATTCTACCAAAGAATTATCTATAATAATTCGCAAATCCGAATCGGCTAATTGTTCTCTGATAGCACCTGCCCCCGTAGAGATTTCTCGGTTTCGAAATGTCTCGAAACCTTGAGTAGTAAAAAAGAGTGGGATGCTGTATTTCCAGGATTGGATTTCATATTCGAATGAACCTCGTAATCGTAAGAAAGTAGGTTTTTTAGCTATGGACCTAGCAAAAGAAAAATCGAGATAGGTTCCTATATATAGTATTGGATCCCCCCCTCACAATCGGACGTGAAGGTTTCCTCTCATCCGGCTCAAGTAGTTACACCAAATAAAGAAAAGGGTTCTTCTTCTGTTTAAACTTTGTTCGAGAAAACCCTATAAAAAGCTACTCTTTACTCAAGTTCCCAGTAAGGACCAGCCTTTCATTGATTCATTCTTATCTTATTTTATTTTTTATTTTCACTCTAACCTTTTTTACTTTCTTTTATGTTTATTTATGTTTACGAAAAAAAAGGAAATGTGAAATTCTTGAATAGTCTACTTCCCCTCAAATGATGAATCCCCTGAAAGGAATATTTTGGGACTCGTAAAGGATTTCTTTGTCTATATATTGTATTGTTCCATTCGATCTTTTTTAGGTCTCTACTCACCTCGATGGTTATGTGCCATGATATCCCTTGAAGCATATATGCGATATATAAGCTCCCGTAACCATGCCATATTCGCTTGTGCTTGCCTGAACAGAATTTCTTTCTCAAAGAAATGGAATGTATAATTCCTAATTCCACAAAAAGTCTTTTTTCACGAGGTATTACTAACTATTCCTATATTATCTGTTACGGAATCGACCATGGATCAATCCCCCTTTTCTTCCATTTTGAAGTCTTGAATGCACCCATAATTCTGAGCTTCATGTTCCTCCTCCCAAGATACATGTCAGAGCCGGGGGCATCCCAATCAGATTAAATGGGATGACAGTTTCTCAGTCCAAATCTGTCAAATGAAAATTTCGATCAAATCACACATCGCAATATACTAGGCCCTCTAATTCCCTAAGGGGCTTATCTAAAAGATTCGCAATATAACTAGGAAGACGTTTCAAATACCACACATGAGTCACTGGACATGTCAGTTTGATATATCCCATTTGGTACCTTCGTACCCGAGAATCAACAAATTCCACCCCGCATTCTTCACAAGATTTCGGGTCTTCTTTTTCAGCCCCAATCCCTCGGTAATTTCCACAAGCACAAATCCCACTTTTTATGGGTCCAGAAATTCTTTCACAAAACAATCCATCTTTCTCCGGTTTATTAGTTTTATAATGAAAAGTATAGGGTTTTGTCACCTCTCCAACTATCTCTCCATTGGGTAGAATTTTTTTTGCCCAAACCTTGATTTGTTGAGGGGAAACTGGTCCAATTCGAAGTTGTTGATGTTTATACTGGTCGATCATAGAATAGAAATTCTGATTCATTGAGATCAAGCTTCCTTCCTATCCATCTGGAAGTTCTTTTCAGATACAAGGAAATGATTCAGTTCCAGGGACAAAGATCGTAGTTCTCGAACGAGCAATCGAAAAGATTCTGGAGCACCCTCTGGGTTAGGTACTGGTGCTCCAATAATCGTAGCACCAAGTACTTCTTGACGAGCTCTAATATGATCAGATTTAGAAGTAAGCATCTCTTGTAAAATATGAGCAACACCAAATCCCTCTAGAGCCCAAACTTCCATTTCTCCTACTCTTTGTCCCCCTTGTTTGGCCCTCCCTCTAAGGGGTTGTTGTGTAACAAGTGCGTAATGCCCACTGGAACGTCCATGGATTTTATCATCAACTTGATGAATTAATTTTAGGATATAGGACTTTCCTATTAGAACAGGTTGTTCAAAAAGATCTCCTGTTCTTCCATCAAATATTCTGCTTTTTCCCGGATATTCGGGTTCAAATACCCATGGATTTTTTGTTTGCTTACTGGCTTCATATAATTCAGAAAACACTAGTTTTCTTGAGGCCTCTTGCTCATATCTCTCATCAAAGGGTCCTATTCTATAATGTTTCTTTAGCAGATCCCCCGCTAACCCGAGCGAACATTCAAATATCTGTCCCACATTCATTCGTGAGGGGACTCCTAATGGGTTGAATACCATATCAACGGGCGTTCCATCTTGCAAATAGGGCATATCTTGTCTAGACAAAATCTTAGAAATTATACCCTTATTCCCATGTCTTCCAGCTACTTTATCACCTACTTTGATTTCACGTTTCTGTGAAATATATACACGAATCCTTTCTGGATTATAATTGGAAACCCCCTTTCTATGGATCCATCTCACATCAATAACTCGACCCCTTCCCCCTATAGGTAGTCTGAGAGAAGTTTCTTTTGAAGTGGATACCTGAATGCCAAGTATAGCTCGTAATAATCTATCCTCCGGGGCATATGACGATTCGCTTGCTGTCTGAGGTGTTAATTTACCTACTAAGATATCACCTGTTTCTATCCAAGATCCCAGTATCACAATTCCATTTCTGTCTAAATTTCGGAGTAAACGAGCCTCTAAATGCGGGATCTCCTTAGTGATTCTTTCAGGACCTTGGCTTGTTACATGAGTCTGAATTTCATATTTCCGGATGTGAAAAGAAGTATAAATATCTTCATAGACCAGACGTTCGCTAATTAGTACTGCGTCTTCAAAATTGTAACCTTCCCATGGCATATAAGCTACTAATACATTTTTTCCTAAAGCGAGTTCCCCACCAGCTGTAGCCGCACCACCCGCTAGAATTTGTCCCTTTTTAATGTATTTACCCCGCTGAACCTGAGTTTTTTGATGCATACAAGTATTTTTGTTGGAACGTTGATACATAACTAATGGAATGCTTAGAGTATCCCCATTACTTGAGAAAATGATCTTTTGAGTATCAGTAGAAATGATTTTTCCCTTGCGTTCGGCTATAGCTGAAATCCCCGAATCTAGAGCCGTTTGGCCTTCCAACCCAGTTCCAACAATGCACTTCTCGGACCGAGAAAGGGGAACTGCTTGGCGCTGCATATTAGAACTCATTAAAGCTCGATTCGCATCATTATGCTCGATAAAAGGAATGAGGGAAGCCCCAATAGAAAAATATTGGAAGGGAAAAATGCTTCTAAGATGAATCTCTTCCCATGCAATACTCAGGAATTCTTGACGATATCGAGCTGGAACAACCTGTTGTTCTTGAATACCCCGATTCAAGGCCAAAGAATTTCCTGCTGCTACCATATAATATTCATCTCTATTTGGTGATAAATAAATCATCTGTGCCTCTTTTGATCTCTCAGATAATTCATAAAACGGACTCTCTATAGATCCCCAATAACCAACCTTCACATGAGTAGCTAATGATCCAATAAGTCCAACGTTGATTCCTTCGGACGTGTCAATTGGACAAATACGTCCATAGTGACTCGGGTGGATATCTCGTATCCGAAAACTAGCAGTTCGCCCCGTCAATCCTCCAGGACCCAAATAACTCCATTTTCGCCCATGAACAATTTGTGTCAACGGATTAGTTCGATCCAAAACTTGAGATAAAGGATGTAGGCCAAAAAACGATTCATAAGTAGTTGTTAATGAAGTTGAAGTTACCAAATTTTGAGGAGTCGGTATCAATTTATGCCTGATTGCTCCACATATAGTTCCTCGAACCGTATTTTCTAAACGAACAAGAGCCAATCCGAATTGATCCTGTAATAGATCTGCTACAGAACGAATACGTTTATTTTTCAAGTGATTCATATCGTCAAGTGTACCCATTCCCAATTTCATTCCAATCAAATGATCCACAGCAGCCAATAGATCTCGTGGTAACAAAAATGTATTGTTTTGGGGTATATCAAGATTAAGTCTCCGGTTCATATTTCGTCGACCAATCTTTCCTAATTCACATCTTTGTTGAAAAAATTTCTTTTGTAATTCCTTGCATAAGGACTCAGAAAATACCGGATCCCCCCCTACACAGGAAAATTGTTGATAAAACTCCAAAATAGCATTTTCTTTTGATCCAATCTTTTTTTTCTCTTTATCATTTGGGAAAGACAAGAAAATTTCAGGGTAACAAACATTCTCTAGAATTTCTCTTATATTCGAACCCATAGCTGATGATAGAACTAGAATAGATATTTTTTGTTTTCTACTTACACGGGCCCATATCCTTGCTTTTCTATCAATTTCTAATTCCGACCTTCCCCCCCAATCCGATATTATAGTACTGGTATAGACAGAAATTCCGTTATGTTCCAATTCTGAACGGTAGTAAATACCGGGACTTTGCAATATTTGGTTGATCACAATTCGGTATATTCCATTTACTATAGAGGTTCCAAAAGAATTCATTATAGGGATGTTTCCAATAAAAACGGTTTGTTCTTGCATATTTCTACCGGTTTTCCAAATTAAGACCGCGGGTACATATAATTCAGAAGAATATGTGAGTGATTCATACACAGCATCTCTTTCTTTTATCAAGGGCTCTACCAATTGATATGTTTCCACAAATAATTGAAATTCAATTTCTTGATCTCTATCTTCAATTTTTTGAAACTTATGAAATTCTTCCGTCAAGCCCTGATTAATGAACCTATAAAATCCCTCAAATTGTATCTGACTAAATCCAGGTATTGTGGACATCCCCTCATTTCCATTCTGGATCATCTTAATCTTAAGTTTCCTCTTTATTGAAAAAATCCCATTATTGGCTTGGCTCACTATTCATCGAACCCTACCGATTGATCTAGCAATGATGGAATGGATATTCTGTTTACTGAATCACATAAAATTTTAGTCAACTCCATCCATATATATCATACGTATGAACGAAAGCAAGACAGAGAAATGGAGGGAATTTTCGATGCAAGTTCTCATTTGAGCTTGAGCCAGGTACAAATAGAAAGAAATGGAAATTTATAAAGCATTACCGGGAAAAATTCTGTCACTTAGGCTGATGGAGTCTTTTATCGGATATCAAAATTGATCCAATTTATACCTAATTCTTTTATTATGATATTATGATCAGGGTGCAAAAAAATAAAAAATCAATGAATTTAGGATTCAATCTGCTCTCTATGAATAAGATAAAAACAGAAGAATCAGGAACAGCATAGAGTTTCCCTTTTTTTAGCACACACAATTGAATGTTCAAAAAGGAATTCGCAAATCTTGGTAGTATAATTTCTAAAATACAATGGAATTGCGTACGTATATAGTCATAGGAGTAATCTTTAGGAAGTACATGCCAATATAGCTACCCGTATATCACATCTTTTATCATAATTGAACTTGGTGCAACATAGGTTAGGTCGCACTCTATACATAATGTCTATCTTCTATTCATATTCAATGAAATATTCAAGCACGATGATCCTATATAGGGATATGTGCATAAGAAATAGACTCGGGCTCGGTATCCATGTATACTGGAGTTTACACTGTTCTGGGGTTTACATATACACATATATTTTCTTATAATTCTAATTGATAATGTTATAGATAATGATTAGTCGTAAATCAATTGGATTTTGCATCCATTAAGGTAATACAAATGGAGATACAAAATTAAGAGCTGTTCACTAATTCAAAGTAAGAAAAGTAAGTAAGAGTAAAAGATTAATAAGTAAATAGTTAATGAAGTAAAGAGTGAATTATTCGAAATTGCCCTGCATTTTACAGTCTGTGACCGGGGCCGGGAATCTTTTCTCTTTTGTGAAAAGAGAAGGTAAGTTTTTAAACGACGCGTGTTTTGAGATAAAAGAAATCGAAGAAAAGAATAGAAACAGGATCCAATAAAAAAGAGGAATTATTTTTTGAAAAAAAAAAATAAGTACAATGGGATTCAAGATCCAAAAATAAAAGAAATTAAGAAAGTAAAAAATTCAAATCAAAACAAAATGAGGAGAGGAAAAGAAATAGAATAATAATATAATTATAGATTTATAGAAAGAGAATATAAGTATAATAATAATATATATTATATATATATAATAATATAATATATAATTATATAATTTCTTTATCCATTTTGGATGGAATTTGGCGGCATGGCCAAGTGGTAAGGCGGGGGACTGCAAATCCTTTATCCCCAGTTCGAATCTGGGTGTCGCCTGATCAACAAAAAAATACTTGGAATTTTTTGATCGAACTTGACGAATTCTTGCCCCGCAAAAGCATAGGCAAGGGCTAGGTCTGTTGATACTTGATTTTGAGAACCCGTAGGGCCTATAAAAGACTGTCATCTTTTTTCTCAAAATCTGGGTTCTGAGTGTGGCTCAAAAAGGTACCAATAAATCTGAAGCAACCCAATCTTATTAATGATTGGTTTGGGCTATTCTGAATTGAATCAAATAAAAGAAATAGTGAGAATTCATTCTGGGCATCAGAACTATGAAAGTAAGAAGTCGGAAATCTTGGTATCCAAAGGTTCCTAAGAGACACTCCGTTTTGGCTACTAAGGTTGAAGAAAGGATTCTAAAAAAGACTATAAAGACTCCTTAATTATTTTACAATATAACTTTCTTAATATTGAGGTAGTGTCTACTCACTCTGTCTGCGATGAAATTAGATTGGATAGGCTGATGGGAATTTCATTTAAGAATTGTGGCAAAGAACTGAAGATACTTTGTATCCAGGTATCCAGACTCACTAGATTCTCTGAGTGCTGCTCATAAATTTCATCAGAATGGTTGAATGGCTCTTCTTTCTATTTGTATATTTTCTTTTTTTTTTCCAATTCTTTCTATTTCAATAGAATTCTATTGAAATAGAAAGAATTGGAACTTTTTATGAGTGGATTCATGAAATTCTTTCATAAAAAGCCGTAAGTAAGAATCCTATTTTGACTCTGTACCATTGATTCCACTATGATTATGAATCAATAATGGAATAATTCCTTCATTTCATAGAGATAGGGGACATCATCCGCATGGATATAGTAAGTCTCACTTGGGCTGCTTTAATGGTAGTGTTTACATTTTCTCTTTCACTTGTAGTATGGGGAAGGAGTGGGCTTTAGAGCTAGGAATATTACTAATTTAGTACTTTACTGAAAAATCTACTTGTATCAATTGTGATCGTTTTGCGAAAGCTTAAAAAAAACTTGACTTGCTTTAGTTTATCTATATATTATTTCTTAGATATATCAGTGGTATTATATTATTATTAGATTTCTATAATTCTCTAATATATGATATGGTATTTATATGGTATATAGTATATGCACGTACTATTCTTCCTACATTAATTCTTTCGATGGCCCCCGGATCCATAAGCATAAGAAGAGATATAAAAAATCAGGAATTCAAGCAGTTGACTTGCAATTATTTTGGATTGATTGAAATGCATACAAATGGGTGTATAGAAAAAATATAAAATTCGAGTGCTATTTCATTTTGATGTACTCCTAATATATATTATTACTTAGATATAGATATCTATTGTCAATTGATCTATATATCTATATAAGATAAAGCTTCTTTCTTTATACAATATACAATACGACTTTATGCTTTATGTACTAAAAATATGAATGAATATGAAATATTCTACAATACTCAATTGTATAGTGCGGTAGAAAGAGCTATATATAGCTCTTTCTACCACACTATATCAAATACTTCTGATTCCACATTTCATTTAATACTTAAATAGAGTTTTAAACCCTTTCATTTCTTCAATCATTGATAAAAATCAAAATGAATAATTCAAGTTTCATTCAAATTAATCATTTTGGCTGACTGTTTTGACGTATATAATAAGTAAAAAGGCAGTAGGAACTAAAATGAACAGCGCAGTAGCAATAAGCGCAAGAATATTGACTTCCATAATCTCTTTGTTTTCTTCTTTCACAATAATTCGGGATCTAATCCCATAGAGATGATAAAGTGGACTCCTATCAATTCAAAGGTATGAATTGCATATTGATGATACTAAGAATATTATGAATAACAATATCAAATCAAATCGATTTATCGTCGCGAATTGAATAGTATAACATAATATAACATAGGAAGATCTTTTATCCATACTCAATCTAAATTAAATAGAAAGAAAGAAAAATAGGATTCTTTTTGTGTCTGTATTCTCGGTCAAAATATAGGCACTCTATTCCATTTCGTTGATCACGAAAATAACAAAAATAAGACGAGTATGGTCACTCTTCAAATACTGAATAGAGTCTGTCTTACTCTAGTAATTAGTAAGAAGTAACGATTTTAAAAATCTACATATGTTTCTCCCTTACCAATCGGTGCTAGTATAATAAATCAAAATTTCCATATTTTTCTGATGAGAAATGTGAAACGAAAACAAAAGAAATAAGGATTCCCCCCAGAGATTAGATTTCGTTCCCCGTCTTCCCTTTTCACGGAAAAGGGAGAGATGAGTTGATCAATTCGTCGGATCGGGACTGACGGGGCTCGAACCCGCAGCTTCCGCCTTGACAGGGCGGTGCTCTAACCGATTGAACTACAATCCCAGCCATATGCATGGCATACATAGTCTTATGATTTCAAGAAGAGCATTCTATTTGTCGTGTTGCAACAGAAACACGAAATATCCTATTCACATAGATAGATATGGACTTGAGTGAGAGTGGCATAGATTACTAGTAATCTATGTTTCTTGTATTTACTGTATTGCAAATGAAAATAAAAAGAATGGATGAGAAAAAATGGACTATTTTTCTTTATTTTATACGGATCCGGGATTTCTGTTTCTAGAGGACAAATTGTTTAGATCTTCTTCATGGAGCGACGGATTATTGGGCCGAGCTGGATTTGAACCAGCGTAGACATCTCGCCAACGAATTTACAGTCCGTCCCCATTAACCGCTCGGGCATCGACCCAGGAAGAATGAATTCTAGGTTTATTGATAATTCATGACCAACTTACTTTCGCAGTCCGCTACCCCCAGGGGAAGTCGAATCCCCGTTGCCTCCTTGAAAGAGAGATGTCCTGAACCACTAGACGATGAGGGCATACTCGCCCCGACTGTCATCATACTATGACAATAGTATGAGTAGTTTTTTGTAATTGTCAATATATATAATTAGAATCAAATGTATGACTAGATCCGAGGAGTCTTTCCTACTTTTATGTTATGATCCCATAGAATTCTTTGGATTTAATGGTTCGTTCATGAATGAGCTATCCCATACTCCCATAAATATTCTATCCTAGAACTATATAGAACTCTATATCTATATAATATAAGTATATACTACATATGTATATGATATGAAATATCATATGAAAATCTATTCAATAAAAAAAGAGTTCAATATGAAATTCCGTTTCAATAATGAAATATAACGAAAACGAAACAAAGTATAAAATCCCTTCAGCTTAGGGAGTTATTAGTCCGACAGAAAAAAGAGTCAAACTCTCATTTCTTTTCTTCAATTTGAACTCATTGCTTCGTTCAGCGTTCAGATGAGTTATACCTATCGCTATCTCACACTAAGCAAAAAAGGATTTAAACATTTTCTTTTTATAAGAATTCGGTTCAGGTTACGAATCCCCCATCACATGATTCAAGAACTTGAATATATGATGGAAAAGTCAACAAAGCAAGTAGGGTTAACCTTGAAACAATTCAATGCATTTTTTTTATTTTATTTTTTATCAAAATATGAATCTTACCCACTTCCTATATAAATCAAACTGATTGTTCCATTATGAGTCTATTGCATATATATCATAATGGAAGATGAAGATGCCTAATTCATGAATTGAAAAAAAAAGGCCCTTTTAACTCAGTGGTAGAGTAACGCCATGGTAAGGCGTAAGTCATCGGTTCAAATCCGATAAAGGGCTTTTTCCACTAAACTCAAATCTGAGTCTTCGTTTTTCAGCGGGGAATAGAAATCTTTTTGATATTTATAAAAAAAAAAGAGAACGACCACCATTATGATTAGAATGAGTTCTGATTAGGAGGAGTCTTCCCCGTAATGACATAGACATCCTCTTCATGTCTCATTATTCCATTTTTTTGTCCTGAGACATAAATATCCAACCCCTATTATGAATCGCCAAACCAAAGTATTCCTACTTCGCCATTGTTCCTATAAAACCCACCGCAAAATTGGAAATAAATAAAAAGTAAGTGGACCTGACCCGTGGAATCATTACTATATCAACTACTCTGATATTTAAATTCGATATAGATTCAATTGTAGAAGTTAATTTTTTTATTTCCTTATACCATTGGACCACACAAGTCAAAAATTTGTTTTTGATTTCATCTTCTTGTTACTGGATGTTCCATAGAACTAAATAGCTATTCTTTTCCTTTCGGCTACATAGAAAAGAAAAATCTCTTTCAAATATGGATTTTCAATATCTTTCCTTGATTTCAGAATCAGATATTGTTTTGTTGTTACACTGATGGAATAGAGAATAAATGCAAGAACTAAGGATTTTTCATTTATAGTCTACCATTATTTAATATTCAATTTAGGGACAGGGAAAATCGGGAATCTGTGGATAATTCAGGTTCCAATGGTTAGATTCTTTTTCTTTTCTATTGGACCGCAAACCGACCTAGTTTATGAGTTCGCCTCGATTAGTCCTTACTATGTACTATATTTTACATATTTTACTATTACTATAGAAAAAAGAAGAATTTGGATCTTCGAACCCATTGGAAAAGGGCATTGAACGAAGAATCGTCCATAGATAATCGAACTACCGCATGCCCTGGAAATGAGATGAAGTGTTCGGAAATGGTTGAAGTAGTTTAATAGGAGGATCACTATGACTATAGCCTTTGGTAGATTTATCAAAGAAGAAAATGATTTATTTGATATTATGGATGATTGGTTACGGAGGGACCGTTTCGTTTTTGTAGGCTGGTCCGGCCTATTGCTCTTTCCTTGTGCTTATTTCGCTTTAGGGGGTTGGTTCACAGGTACAACTTTTGTAACTTCATGGTATACCCATGGATTGGCTAGTTCCTATTTGGAAGGTTGTAATTTCTTAACGGCTGCGGTTTCTACTCCTGCAAATAGTTTAGCACATTCTTTGTTGCTACTATGGGGTCCTGAAGCACAAGGAGATTTTACTCGCTGGTGTCAATTAGGCGGTCTGTGGACTTTTGTTGCTCTCCATGGTGCTTTCGGTCTAATAGGTTTCATGTTACGTCAATTCGAACTTGCTCGTTCTGTTCAATTGCGACCTTATAATGCAATTGCATTTTCTGCTCCAATTGCTGTTTTTGTTTCTGTATTCCTGATTTATCCACTGGGTCAATCTGGTTGGTTCTTTGCACCCAGTTTTGGTGTAGCAGCTATATTTCGATTCATCCTTTTCTTCCAAGGGTTTCATAATTGGACGTTGAACCCATTTCATATGATGGGAGTTGCTGGAGTCTTAGGCGCTGCTCTGCTATGTGCTATTCATGGCGCCACCGTAGAAAATACCTTATTCGAAGATGGTGATGGTGCAAATACATTCCGTGCTTTTAACCCAACTCAAGCTGAAGAGACTTATTCAATGGTCACTGCTAACCGTTTTTGGTCCCAAATCTTTGGGGTTGCTTTTTCCAATAAACGTTGGTTACATTTCTTTATGCTATTTGTGCCAGTAACCGGTTTATGGATGAGTGCTATTGGAGTAGTTGGTCTGGCTCTGAACCTACGTGCCTATGACTTTGTTTCCCAAGAAATCCGTGCAGCGGAAGATCCTGAATTTGAAACTTTCTACACCAAAAATATTCTCTTAAACGAAGGTATTCGTGCTTGGATGGCGGCTCAGGATCAGCCTCATGAAAACCTTATATTCCCTGAGGAGGTTCTACCCCGTGGAAACGCTCTTTAATGGAACTTTAGCCTTAGTTGGTCGTGACCAAGAAACCACCGGTTTCGCTTGGTGGGCCGGGAATGCGAGACTTATCAATTTGTCCGGTAAACTACTTGGGGCTCACGTAGCTCATGCCGGATTAATCGTATTCTGGGCCGGAGCAATGAACCTATTTGAAGTGGCTCATTTCGTACCAGAGAAACCCATGTATGAACAAGGATTGATTTTACTTCCGCACTTAGCTACTCTAGGGTGGGGAGTAGGTCCGGGGGGAGAAGTTATCGACACCTTTCCATACTTTGTATCTGGAGTACTCCACTTAATTTCTTCTGCAGTCTTAGGCTTTGGCGGTATTTATCATGCACTTCTAGGACCTGAAACTCTTGAAGAATCCTTTCCATTCTTCGGTTATGTATGGAAAGATAGAAATAAAATGACTACAATTTTGGGTATTCACTTAATTTTATTAGGTATAGGTGCTTTTCTTCTAGTACTCAAAGCTCTATATTTTGGAGGCATATATGATACCTGGGCACCCGGAGGGGGAGATGTAAGAAAAATTGCCAACTTGACCCTTAGCCCAAGCGTGATCTTTGGTTATTTACTAAAATCTCCTTTTGGGGGAGAGGGATGGATTGTTAGTGTGGACGATTTAGAAGATATAATTGGAGGACATGTTTGGTTAGGTTCCATTTGTATACTTGGCGGAATTTGGCATATCTTAACCAAACCTTTTGCATGGGCTCGCCGTGCATTTGTATGGTCTGGCGAGGCTTACTTGTCGTATAGTTTAGGTGCTCTATCTGTTTTTGGTTTCATCGCTTGTTGTTTCGTCTGGTTCAATAATACCGCTTATCCTAGTGAGTTTTACGGACCCACTGGGCCGGAAGCTTCTCAAGCTCAAGCATTTACTTTTCTAGTGAGAGACCAACGTCTTGGGGCTAACGTGGGATCCGCCCAAGGACCCACTGGTTTAGGTAAATATCTAATGCGTTCTCCGACCGGAGAGGTTATTTTTGGGGGAGAAACTATGCGTTTTTGGGATCTTCGTGCTCCCTGGTTAGAACCTCTAAGGGGTCCCAATGGTTTGGACTTGGGTAGGCTGAAAAAAGACATACAGCCTTGGCAAGAACGCCGTTCGGCAGAATATATGACTCATGCTCCTTTAGGTTCTTTAAATTCCGTGGGTGGCGTAGCTACCGAGATCAATGCAGTCAATTATGTATCTCCTAGAAGTTGGTTAGCTACCTCTCATTTTGTTCTAGGATTTTTCCTATTTGTGGGACATTTGTGGCATGCGGGAAGGGCACGTGCAGCTGCAGCGGGGTTTGAAAAAGGAATCGATCGTGATTTGGAACCTGTTCTTTCCATGACCCCTCTTAGCTGAGATTTTCTTATTTATATCTATTTGTCTATTTTTCTTTTCTGGCTCGGCTAGGCGAGATAGCCGAGCCATTCCCTTTATGAAAAAAGGGGCCAGTCCAAACGAGTAAAGAAGTCATTTCTTAAGTAAGCAAAAGGAGAGAGGGGGATTCGAACCCTCGATAGTTCCTTGTTAAGAACTATACCGGTTTTCAAGACCGGGGCTATCAACCACTCAGCCATCTCTCCCAAATAGAATCTAGATTTCTTCCTACGAATAGAACATGACCTTATGAGATGATACATTAACTAGCTAATAGATTCCATGTCCGAATAAAAAAAAGAAAGCGGTAATAAGTTCTAATGAATCAATTCATTCATGTCAAATCCCTTCATGATGCATTTTTACTAAGCGAGGGATCCTATGGTATAGTTCATTTGTTGGTAGTTTGGAGGATTACAAACATGACTATTGCTTTCCAATTAGCTGTTTTTGCATTAATTGCAACTTCATTAATCTTACTGATTAGTGTACCCGTTGTATTTGCTTCTTCTGATGGTTGGTCAAGTAACAAAAATGTTGTATTTTCTGGTACATCATTATGGATTGGATTAGTCTTTCTGGTAGCTATCCTGAATTCTCTCATCTCTTGAACTTTTTTGGTATTTCCCCGATCCAAAAATAACATTTCATTGTTATGAATTAGATATAGATAATTCAGAATCTGATCTTAATTGGAATCTTACTTTACTTACTAGAGTCTTTGTATTTGGCCCTGCGCAAATATGATCCAGACGCATATATGATATATTATATATGTCATATATGTGTGGACATATGCGTATGTATCAGGAACGAAAAAATGCGGATATGGTCGAATGGTAAAATTTCTCTTTGCCAAGGAGAAGATGCGGGTTCGATTCCCGCTATCCGCCCATGGTGAAATAATGTACCATAAAAAGATTCCGTCGGTATAGTTCACTACTATGCATACTAGTATGCATAGTAGTGAATATAGTAGTTCTATCTTCCGCTTCACCTTTTTTTCCTCCCAACAAAAAAAAGTCATTAATTACTACAAATACAAAATAAAAGGGGATTTCACAAAAATGTTGCGGAGACAGGATTTGAACCCGTGACCTCAAGGTTATGAGCCTTGCGAGCTACCAAACTGCTCTACTCCGCGCTGAACAACTGGGAACTGATGGACGAAGAAAGGTTGGATATGCCCCTCTACCATATCTATACAAATAGAATAGTCTATTTATACAAAATGGTAAAGGGGCCTCCTCTATTCTATAGATCATAGAGATCTATCCAAATAAGAAACAAGATTTTCTCCTTACCAACTTGATCTTGTTGCGCCTGGTAACAAACATGCATGAACCATTTCTCGAAGTATGTGTCCAGATAGTCCAAAGTCTCGATAGTTCGCTCTAGGTCTTCCGGTTAAAAAACAACGTCGATGAAGACGTATAGGCGCACTATTACGTGGTGAGGATTGCAATTTTCCATGAATTTTCCATTGTTCCCTCAACGAGGGAACTTTGCTTATTTCTTTTTTTAAGGATTGACGAATCCAATAATATTTCTGTTCCAATTTCTGGCGCTTCTTCTCCCTTTGAATCAAACTTTTTCTTGCCATAATGTTCAGTTCCTATTTTTATCAATTATACAGTTCGGATCCTAGATGGAAAAATAGAAGAAGGTACATACCTCCTCTTCATCGAAAGAAATGAGATTTTTGTTTTTGGGGGTTGGGGATAGGGTACAAAAAAACAAGAATTAACTAAATTAACCAAATTTTCCTGATGTGGAGGCAATCAAGAAAGCTGCGTAAGTGAATATATAACCTACGGAAAAGTGGGCTAATCCAACCAATCTTGCTTGCACAATGGAAAGAGCCACTGGCTTATCTTTCCATCGAATCAAATTAGCCAAAGGTGTCCGTTCATGAGCCCACGCTAAAGTTTCAATCAATTCCTGCCAATAACCACGCCAGGAAATTAAGAACATAAATCCAGTAGCCCAAACAAGATGTCCAAATAAGAACATCCACGCCCAGACCGATAAACTATTCATACCAAAAGGGTTATATCCATTGATAAGTTGTGAAGAGTTTAACCATAGATAATCTCTTAACCATCCCATCAAATAAGTGGAGGATTCATTAAATTGTGAAACGTTACCCTGCCACAATGTGATGTGTTTCCAATGCCAATAAAAAGTAACCCATCCAATGGTATTTAGCATCCAGAAAACTGCCAAATAAAATGCGTCCCAAGCAGAAATATCACAAGTACCGCCACGCCCGGGGCCGTCGCAAGGAAAACTATAACCGAAATCCTTTTTATCTGGCATTAACTTGGAACCACGTGCATCTAAAGCACCTTTTACTAAAATCAATGTAGTCGTATGCAAACCCAGAGCAATAGCATGATGAACCAAGAAATCTCCCGGCCCTATTGTTAAGAAAAGTGAATTACTATTCTCATTAATAGCATTCAACCAGCCAGGTAACCATATGCTTCGACCTGCATTGAATGCCGGGCTATTCGTTGAAGATAAGAGTACATCGAACCCATACGAAGTCTTGCCATGAGCAGATTGTATCCATTGGGCAAATATGGGTTCGATTAAGATTTGTTTTTCCGGAGTACCAAAAGCAAGCATGACGTCGTTATGAACATAAAGGCCCAAGGTATGGAACCCCAGAAAGAGGCTGGCCCAACTTAAATGAGATGTTATAGCTTCTTTATGGTCTAACATTCTTGCCAATACATTATCCTCATTCTGTTCCGGATTGTAATCCCTAATGAAGAATATAGCCCCATGAGCAAAGGCTCCTGTCATGATGAACCCTGCGATGTATTGGTGATGAGTATATAACGCAGCTTGAGTAGTAAAGTCTTGTGCTATGAATGCATAAGCAGGTAAAGAGTACATGTGTTGAGCTACTAAGGAAGTAATAACCCCTAAAGAGGCTAGAGCAAGGCCCAATTGAAAATGAAGCGAATTATTTATTGTGTCATAAAGACCCTTATGCCCACGTCCTAATCGACCCCCCGGAGGAATATGTGCTTCTAAAAGATCTTTGATACTATGCCCAATCCCGAAGTTAGTTCTATACATATGACCAGCAACGAGAAAAAGGAATGCAATAGCTAAATGATGATGAGCAATATCGGTTAGCCATAAACTTTGCGTTTGGGGATGGAACCCCCCAAGAAGGGTTAGAATGGCAGTTCCTGCTCCTTGGGAGGTACCAAATAAATGACTACTGGAATCAGGGTTTTGAGCATAAAGATTCCATTGACCCATAAAAAGGGGTCCCAACCCTTGAGGATACGGCAATACGTCTAAGAAATTATTCCATCTGACGTACTCTCCCCTCGATCCGGGAATAGCGACATGAACTAAATGTCCTGTCCAAGCCAAGGAACTTACTCCAAAAAGCCCTGACAAATGATGATTGAGACGAGATTCGGCATTTTTGAACCAAGAAACGCTTGGTTTCCATTTTGGTTGTAGGTGTAACCAACTCGCTATTAAGGATATAGCAGAAAGAAGTAATAGAAAAAGAGCTCCAGTATAAAGATCCCCATTGGTGCGTAATCCGATTGTATACCACCACTGATAAACACCAGAATAAGCGATATTCACTGGACCGATAGCGCCTCCTCTAGTAAAGGCTTCTACAGCAGGTTGACCAAAATGAGGATCCCAAATTGCATGAGCAATAGGTCTTACATGTAAAGGGTCCTGTATCCATGACTCAAAATTTCCTTGCCAAGCTACATGAAACAGATTTCCGGACGTCCACAGAAAGATTATTGCTAACTGTCCAAAGTGCGAAGAAAAAATGTTCTGATAAAGACGTTCTTCAGTAATATCATCATGACTCTCGAAGTCATGTGCGGTAGCAATACCAAACCAAATACGACGAGTAGTGGGGTCCTGAGCTAAGCCTTGGCTAAATCTTGGGAATCTTAATGTCATAATGCCTTTCAAATCCTCCTAGCCATTATCCTACTGCAATAATTCTTGCTAAGAAGAATGCCCATGTTGTGGCAATTCCACCCAGAAGGTAATGGGTTACCCCTACAGCACGTCCTTGTACAATACTCAAGGCTCTAGGCTGGGTAGCAGGAGCAACTTTTAATTTGTTATGAGCCCAAACGATGGATTCAATGAGTTCTTGCCAATAACCACGGCCACTAAATAGAAACATTAAACTGAAAGCCCAGACAAAATGAGCGCCTAAGAAAAAAAGACCATATGCAGATAATGAAGAACCATAAGACTGAATTACCTGAGATGCCTGTGCCCATAAGAAATCCCGGAGCCATCCATTAATAGTAATGGAACTCTGTGCAAAGTTCCCTCCTGTGATATGAGTGACTACTCCTTGATCACTTATAGTACCCCAAACATCCGACTGCATTTTCCAACTGAAATGGAAAATTACTACCGAAATTGCATTGTACATCCAGAATAGACCTAAGAATACATGATCCCAGGCGGATACTTGACATGTCCCCCCCCTACCAGGTCCATCACAAGGGAAACGAAAACCAAGATTTGCTTTATCAGGTATCAAACGGGAACTGCGAGCAAATAGAACACCTTTCAGTAGGATCAATACAGTCACATGAATTGTAAATGCATGAATGTGATGAACCAAAAAATCTGCAGTTCCTAATGGGATAGGTAACAAAGCAACTTTGCCGCCCACTGCTACTAACTCACCACCTCCCCAAGTTAAGCTGGTACTTGTTGTTGCACCAGGAGCTGTTACGCTAGGTGCTAAAGCATGGGTGTTTTGTACCCATTGAGCAAAGATGGGTTGTAATTGTATAGCGGTATCTGAAAACATATCTTGGGGACGCCCTAAAGCGCTCATAGTATCATTATGAATATACAAGCCAAAACTGTGAAAACCTAGAAATATGCATGCCCAGTTCAGATGTGATATGATTGCATCACGGTGTCTAAGGACACGATCTAATAGATCGTTGTATCGAGTAGTTGGATCATAGTCTCTTACCATAAAGATGGCTGCATGTGCAGCAGCGCCAACTATAAGAAATCCCCCAATCCACATGTGATGTGTGAACAACGAAAGTTGTGTACCATAGTCAATAGCTAGGTATGGATAGGGGGGCATAGAATACATATGGTGAGCTACAACAATGGTTAAAGAGCCTAACATAGCCAGGTTCAGAGATAATTGAGCATGCCATGACGTTGTTAGTATTTCATAGAGACCTTTATGGCCCTGGCCCGTAAATGGACCTTTATGAGCCTCTAAAATGTCTTTCAGGCCATGACCAATGGCCCAGTTAGTCCTATACATATGACCTGCGATCAGGAACAGAATCGCAATAGCTAAATGATGGTGTGCAATATCACTCAGCCAAAGACCCCCTGTTATTGGATCTAATCCTCCACGAAAACTCAGAAATTCCGCATATTTTGCCCAATTCAAGGTGAAAAAGGGTGTTGCTCCCTCGGAAAAACTTGGATAAAGTTGAGCCAAAAGATCCCGATTCAAGATAAATTCATGAGGAAGTGGTATTTCTTTAGGATCAACTCCAGCGTCAAGAAATTGGTTAATCGGTAAAGATACGTGGATTTGGTGTCCCGCCCAAGAAAGAGACCCAAGTCCTAGTAACCCCGCTAAGTGGTGATTCAACATAGATTCTACATCTTGGAACCAAGCCAATTTGGGAGCGGCTTTGTGATAATGGAACCAACCAGCAAAAAGCATTAACGATGCAAAGACCAATGCACCAATTGCGGTACAATAAAGTTGTAATTCACTAGTGATTCCAGATGCTCGCCAAATCTGAAAAAAACCGGAGGTTATTTGTATTCCTCGGAAACCACCGCCCACATCCCCATTCAATATTTCTTGACCTACTATTGGCCAAACTACTTGGGCACTGGGTGCAATATGAGTAGGATCACTTAGCCATGCTTCATAATTGGAAAAACGGGCGCCATGGAAGTACATGCCACTCAGCCAAAGAAAGATAATAGAGAGTTGGCCGAAATGAGCACTAAATACTTTTCGAGAGATCTCCTCCAAATCACTGGTATGACTATCGAAATCGTGAGCATCAGCATGTAGGTTCCAGATCCAAGTGGTAGTATCAGGACCCTTAGCTATTGTTCTTGAGAAATGGCCGGGTCTGGCCCATTCCTCGAAAGACGTTTTTATAGGATTTCTGTCTACAACAATTTTCACTTCGGGTTCCGGCGAACGAATAATCATTAAGTCCTCCTCTTTCCGGACAACACATACAAAGAGACCCGCCAACAGTCAAGTTTTTAGTGAAGATAGATATTCACTAGAGAAATTATGATATTGAAGTCGATCCGGGGCAAGTGTTCGGATTTATTATGACATAAATAATAGGTGCCGGGGGGACCTATTATCTTGCCAAAGCATTTCCCAGGATAACGAAAAAATGATTTTTTTGCAACCTAGCTTCTTTCTATTTAGATTCTGAATGGCGAAGTGCCCATATATATTTACTTTAATCGAAAATTAAAAAATCTAATATAGAATCTAAATAGCTAAATAGAATATAGAAATCTAGATTCTATTTAGCTATTTTTTTTTTATTAAGAATTATGAATGATTTGAATTTTATACTTCATAAGATTCATCAAAATAGCTTTATTAGTTCTATGCTATATGATATCTGTAGCATTTATCCTTATGAGATACCGTAGAAAATGTACAAAATCAAATGATTTTAGAAATTTAGAAAGAAGGGATATAATAAAATTCTTGATTGGATCTTCTCATAGGAACGATTTCTTTAATTTGATTGCTGGATCAAAAAAAAATAGAATGGTCTTATTCAAAACGCCTCGTTATTTTTAACCAATTATGTGCTTCAATATAATTCCCTGGAGTAAGCGCTATAGCTTGTTTCCAATACTCAGCAGCTTGATCGAACCAAGCCTCCGCAATTTCCGAATCGCCTTGTAGAATGGCCTGTTCTCCCCGGTCAGAATAGGTTAGTAAATTCCCTCCTTTAGAACCGTACTTGAGAGTTTCCTACCTCATACGGCTCAGCAATCTATTATTTTTGTGTCCCATCTTCTCTTAATCTATTCTATTTTCTCTTGGGTTAACCAGAAGATGTTTATTGCATAAGTTTCCAATTCTAATTTGGATCAATGATTAGTTTTCTCTTTTCTCCCACCTTCAGAAGAATGAAGCATAGATATCCCCCGATATCATTAGAATTTTCTGAAAGGTAACTATCTCGGTTTCATATCTCATATATGGTATATGAGATTTCTATTTATATAGAATCTTTGAAAAAGACTTTCCTCCGTTAAGAAAAAAGAACTTACTATCTTTGGGATCTGATGCTACACCGCTGCTCAATACTTTAGTAGATCAACTCTATTACATAAGTTGATTTCTCTCCCATATCATGACATAAGTAGACATAAGTAAGCAGTTCTGAACTGTATTTACCATAATAACTTACTAATGGATCTTTACGGTGCTTTCTCTATCAATTCGACTCTTTATCCATAGAGTATAGTATATAGGCCATACCCATTTCTTCCGATTTTTTTTGGTTCTCGCGAAGTCTTTTTCCTTGCTACAGCTGATAAAAATCGTTACTTTGGACGATTCTTATGTAGAAAGCCTATCTTTTTTCTAGTATTTACTAGACAATTAAATCTTTTTTTCTTTCTATAGTGAAGATAGTCGCACGTAATGACAGATCACGGCCATATTATTAAAAGCTTGTGGTAAAAATGGATTTCGTTCTAGTGCTCGGAAATAATATTCCAAAGCTTTTGTATGCTCTCCGTTGCTTGTGTGTATAAGACCTATGTTATAGAGTATATAACTTCGATCATAGGGATCAATTTCTGGTCGCGTAGCTTCATAATAATTCTGTAAAGCTTCTGCATAATTTCCTTCGGATTGAGCCGACATCCGTTACGGTCGTTCATTCTAGTAAAAAAATCTCCGTTCCAGAACCGTACGTGAGATTTTCATCTCATACGGCTCCTCCCTTCTGCGCATAGTATTAAGAGGAATAATTCATTTCACTATTCTCATTATGAACTGACAGGAGCTGGTATTTTTACAAGAAATTTCTAGCCAGCCTTCCCACAAGAGGTTTTTTCTTAACACCAATCATATTAGTGCTATATAGAAATGGTAACTCCAAAGATTTCTTTGTACTTAACGCTTACGATTTCCAGGAATTAGTCACTTCAACGGTCTTTGATGGTTATACGGGTATCAAAAGTACGAATGAGATGGATCTTTGTTTTCCTAACCATTCTTTTTAGTCCCGATACCGATAAGGAAAAGGGTTATTTATAACAAAGTTTTTGTGTTGTTGATTCCTAGGTGTAGTGCTTTTTCCCCGATGCCATCTATTGGTACTAAATGAAGTAGTAATGACCTCCAATACAGAACCTATAGATGTAACCTTTCGCTCAATACTAAAATTGATAATTGAAGCATCTAAGGCTGCATCAATCGAGGATACACGACAGAAGGAATTGCTCTATCTTTAAACTTCACCTTCACCAAGCGTAGGTTTCTTTCACTAATTTGTTTTTTTCTATTCCTAACTACGTTCTTTTTCTCGTAAAACTGAGGGGTAAAAAAAACAAGAAAAAATCAAATCACACCATCTCTGTAATAGGTAAATGCCTTTTTTTCTCCTGAAGTTGTCGGAATTATTCGTAATAAAATATTGGCTACAATTGAAGAGGTCTTATCAATAAAATTTCCATTTATTCGAGATCTAGGCATAATTAGCAATCCATTCTATAATTCTTCTCATCCCCCTTCGGGGAAAACAATCCCACAAAAAAAAGGAATTGTACAGTACAAAATAACATAAAAACAGACTAATTGGAAAAAATGTGGTGTCCCCCTTTTGGACAAAGATGAAATGAAATAGTTGAATCAGATTAGATTTCATTCCAATTTCGTAGTATATTACTATTTCATCTAAGTTGAATAACCAAGTTTACTATGGATTTTGATAACTCGAGAAGTTTTGATTTGGTTATGATCCAAAAAGGAAAAGAATGGAATACTCATTCCATGATAAATTCAAATATTCAAATAAAGTAACCATCTTTTTTGTTTGCATAACGTGTATGTGCCACACCATACAATTGAAATAGAAAGATTCGTCGGACGAATCATGAATTCCATGGGTTAAAAGAAATTTCTTCTTATGGAACCATCGGGCGATCATACATGTACTACAATTAAGATAAAGGACTCGCTATTCATTCGGGTTTTGGTCAAGAATAACATTCTGTAGGAGAGATGGCCGAGCGGTTCAAGGCGTAGCATTGGAACTGCTATGTAGACTTTTGTTTACCGAGGGTTCGAATCCCTCTCTCTCCGTTTCTTTTCATTCATCAACGTTACCGATTACAATGTATCAAATAAAATAAGAATTGATATCATTATTCTAATGATAAGACCTTTATTTAATAGACATTCTCTATCCCTAATTAATCCCTGTGATAGGTAAAATACAAATAGAGATATCGTATTGATATTGAAAAAAAGAAAAAGAATTCTATTGCCGATCCTTTTTTGATACATGAATGAGACAGGGCACGAGGTGCTCTATTTACTTCAGCGAAAAGAGTCAAAATTGGTATGAACCTTGCTTTTTTATTTTCATTAGAATCAAGTCTGACGGGAATAATATTCTACGACCAACAACTCATTTATTTTAAGACCGATCCATTTACTATCTATTATTTGATTGACAAATCCTTTATATTGTAAGGAGTCAATAGTCAAATGGTTTGGCAATTCCCCGTGGGGGGATGAAACAAGATAATTTTGAATCAGAGCTTTCGATCTTTCTTTATCCTTCGTAGTAATAATATCTCGGGGTTTGCAACGATAACTTGGTATATCCACTATACGACCATTAACTAAAATGTGTCTATGGTTAATTAATTGTCTGGCTCCAGGAATGGTCGAAGCCATACCTAATCTAAAAAGGATGTTATCCAAACGCATCTCAAGTAGTTGTAGTAAAACCTGGCCTGTTGACCCTTTGGCTTTTCCAGCAATATGCACATATTTAAGTAATTGTCGCTCTGTCAGACCATAATGAAAACGCAATTTCTGTTTCTCTTCTAAACGAATACGGTATTGTGATCTTTTTCCAGAGCGCAATTGGGTTTGAAGATCACTTCTGGATCTGGGTCTTTTACTAGTTAGTCCCGGTAAAGCCCCCAGCCGGCGTATTTTTTTGAAACGAGGTCCTCGGTAACGAGACATATAAAGGCTCCTTTTTGATTCACTTTTATTTGACAAAAAAATATAAATTCAGACTGAACTAAAGGATCAGCAAAGCAAAACTCAATTTACTAAAGTCCTACAAAACAGAATAAAAGAAATTTTCTCAATATTCGGATTTTTTGTATATATAGGAAATTAAAGCGAAGGTTACATTTTCCAATTTCTTCTGTAGAGATCTAATTGTTCTAGTCAACTTTTTTATTCATAGCTATAGCTGCAAGTTACCATAACATAATAGATCGGTGACCCGACATTTAGAGAAAGCGAGAGTAGATATTTTTACTCATGGAAAGAAAAAAATTGATAAAGAAAAAGAGCCGGCTATCGGAATCGAACCGATGACCATCGCATTACAAATGCGATGCTCTAACCTCTGAGCTAAGCGGGCGGATATAAGAGCAATAGTGTATAGGAATACAGGAAACTATCGGATCTTAGCTATTACCTAGTGATTCTTCTTCTTTTTTTAATTTATTGATTATTTAAATTTCTTATATTTATTAGTTATATATTTTAGATTCTATTTAGAAAATAGAAAAGAAAACTATTTAGATATAGATAAATTAGATATCTAAATAGAAATGAAATTCCATATTCATATATATATATGAATAGAAAATATCAATATATCAATGAAATTAGGATTTGAAATGAAAAAAAAAAAGAATGAATATCGACCGTTCCACTATTCCAAACTGCACTGTAAAAATTAATGAGGAGGAAAGGCACATATATATGTGGGATATATCTATCCATATTGAATTGGGAATACATCAATGATAGAATCAATTTCGTATTGAAACAAATAGGGTTCATCTAATAGAGATGAAATGATAGAATATAGAATAGGGGGTGGAAAAAAAAGAAAATAGCAGCATACACTTTTTCGATATAGGAATCATTACCTAATGAATTCAATAGTCCCAAGATAAATGAAAGAGGTGGATGAAATTACCCTTGTCTCAAAAGAAAGGGGGATATGGCGAAATTGGTAGACGCTACGGACTTGATTGGATTGAGCCTTGGTATGGAAACCTGCTAAGTGGTAACTTCCAAATTCAGAGAAACCCTGGAACTAAAAATGGGCAATCCTGAGCCAAATCTTTGTTTTGAGAAAAAAGATGGAAAATGAGAATAAAAGGGATAGGTGCAGAGACTCAATGGAAGTTGTTCTAACGAATGAAATTGACTACGTTACGTTAGTAGCTAAAATCCTTCTATTGAAATGACAGAAAGGATAACCTTATATACCTAATACGTACGTATACATACTGACATAGCTCTATATATGAAAATAGAAATCTTCTATTTCTATTATATATTAATTATAATGATAGAGATCAAAAAATCTATGAAAAATTGAAGAGTTATTGTGAATCAATTCCAATTTAAGTTGAAAAAAGAATCGAATTCAAATATTCAGTGATCAAATGATTCATTCCAGAGTTTGATAGATCTTTTGAAGATTAATTGGACGAGAATAAAGAGAGAGTCCCATTTTACATGTCAATACCGACAACAATGAAATTTATAGTAAGAGGAAAATCCGTCGAATTTTTAAATCGTGAGGGTTCAAGTCCCTCTATCCCCAATAAAAAGCCCATTTTACTTCCTCACTCTTTATTTATTCTCATCCTCTTTCTTTTTTTTCATCAGTGGTTTAGTTTAACCAAAATGAAATATCTTTCTCATTTCATTAACTCTGTTCTTTCACAAATGGATCCGAATCAAAATCCTCGTATCTTCTTCCAATCCAATCTCATTTGTTTTTTATAGTACGATATGAACATATATATATATGTTCAAGGAATTTCCGTTATTGAATCATTCATAGTCCATATCTTTTTCCTGACATTTACAAAGAATGTTTTCTTTTTGAAGATCTAAGAAATTCAGGGGCTAGGTCCAATTTGTTAATATTTTATTTTTTAATTCTTTTCATTGACATAGATATAAGTACTCTGCTAGGATGATGCACGGGAAATGGTCGGGATAGCTCAGTTGGTAGAGCAGAGGACTGAAAATCCTCGTGTCACCAGTTCAAATCTGGTTCCTGACACGTGAATAATGTATCGGATAGATATTCATACCTCATACAAATGAAATTAATTCATTGAGATGAGATATTCTTTACTTTCTTTTTCATTTTTTTCCTCTCTATTCACACTTTTCTTATTCCAAAAGTATGTGAAAATTTCGTATATATCTCAAATCTAATAGCTAAAAAAAATTAGCTAAAAGGATTCAATCAAAGATTGGAAGGATAGGAATAGAAAGGATGTATTTCAGACATAGTACAAATAAACTCCGATTCTTCTTATTTTGCATTTCTTCATTTCATCTCTTCTGTCTTTTCTTTCAAATTTCATATTTTTTTGTCACTCTTGCTCAAGTTACTTTCTGAGGTCCCCACTAAGTGATGTGCGAGGTACAAAGTTCATGGTGCAGAATCATCCTATTGTGCTCATACGAAATGTATATTATATGATATCTTCCCGATTGGGGAATAGCAATGAGAGCTTCTTTTTCTTTTTTTTATTTTAGACCCTCCACAATACGAATGAAAGTCCAGTTACTATGTTTCATCTAGAAGGGGAATGCCAAGATGCTCATTGATTGATAAAAAAGGGGTTTTTTATCAATCAATGAGCATCTTGAATTTCATAGAAATTGGGAGTAATATAGTCTTTACGTAAGGGCCAGCCTATCCAACTTTCAGGCATCAAGATACGTTTAAGGCGAGGATGATTTTCATAAGAAATTCCCAACATATCATAAGATTCCCGTTCCTGAAAATCAGCACTTCTCCAAATCCAGAAAACTGACGGGGTTTGAGGATTACTCCTGGGAGCAAATATTTTTATGCATACCTCTTCTGGTTTATCTATACCATACCGTATTTTCGTAAGGTGATACACACTAGCTAAAAATCCGCCTGGTGCTACATCATAGGCACACTGGGAGCGCAAATAATTGTAACCATATACATATGAAATGACAGCAATGGAATCCCAATCCTCGGTTTTTATTTGTAAAGTCTCTATTCCTCGGCAATCAAAGCCTAAAGATCTATGAATTAGCTCATGCTTGACTAGCCAATCAGATAAATGAACCTGCATCTTCTTCATTTCTCCCACATTTTTCTTTGTATAAATATTTCACATTGACAATGAAATTGTTAATGATTGACCCACTGTTTATTATTCTGTACAAATGAACCCTGCCTGATTCACTAATTCGTAGGAAGATACTGACCTTTTGGATTTGAAATCTTTTTCAAATCCAAAAGGTATCTCTGAAGTAGATGGTGATTGATAGAGTAATCCTTGATCGTAATTTCCAGTATGAGTACTGCGTCGAAGGTAAAACTTGTGATTAGTAGTAAAACATCGATTCTCCTGTTGATACGTAGTTCTATCTTCAAATATTTTTCGGGATATTTTCTTACGAAGTTTCGTTATAGCATCGATAATTGCCTCTGGCTTAGGCGGACAGCCTGGCAAATAGACATCCACAGGAATTAGCTTATCGACTCCGCGAACAGTACTATAAGAATCAGTACTGAACATCCCTCCTGTAATAGTACAGGCTCCCATAGCAATGACATATTTTGGTTCAGGCATTTGCTCATATAATCTTACTAAAGAGGGAGCCATTTTCATTGTTACTGTTCCGGCTGTTAAAATGAGGTCTGCTTGCCTTGGGCTCGATCTTGGCACCAACCCATAACGATCAAAGTCGAATCGCGAGCCTATTAATGAAGCAAATTCAATGAAACAACAACTGGTACCATAGAGAAGTGGCCATAAACTGGAAAGTCTTGACCAATTCGAGAGATCATTCGATGTAGTTGAAATAATTGAATTGGGGGTTGTTTGGTTAAGTAATGGAAACTCAACCAAATTCATAACTGTTTCAATGTCATCCTTTCCTTCCCTTTTTTTTTTATTGTCTAAATATTCAGCTAAGACCATTCCAACGCTCCTTTTCGCCATGCATAAACTGAACCCACAATTGGGATAAGCACGAAAATGAAAGCTTCTATAAATACAGATACACCCAATACATCAAAGCTCATTGCCCATGGATAAAGAAAGACCGTTTCAACATCAAAAACAACAAAAACTAGAGCAAACATATAATAGCGAATTCGGAATTGTACCCAAGCATCCCCCATGGGTTCTATACCTGATTCATAACTAGAAAGCTTTTCTGGACCTTCCCTAATCGGGGCTAAAATCCCAGAAATGACAAATGCCAAGATAGGAATAACACTTGATATTATTAGGAATGCCCAGAAAATATCATATTCGTGAAGCAGAAACATAAGAGTACTCCTATAAATGTGGATATGAATGTGGAATAGGTTGAATTCTTCCATTGGAATTTGGAATTTTCAAATCATCTAGAATCTAGAACTTCTTAGATGAAAGATGAAACAATAAAAGAATTTTGATCAAATAAAGCCGCATAGTTGAGAGTTTGTTTGCTGTAGGACATACCTTGTTTCAAGATTCATCTAATGTCATCCCACTTCTATTTTTTCTTTTTTTTATCCTTTCAATTCTATTTATATATGCGATGTGTAGACATAGCATGCTCTTATACTTAGTTATTTTTATTTTAGATTCTCCTTATTCTTATACTTAGTTTATACTTAGTATCTTTTATTATCTTAGAAATCTTAGATAGTAATTAGAAAGTAAAGACTTATCTTATGTATATTTATTTCATCTTGATCTTATATCTTATAATTATATCTTATAAATAAGATAGTGAAAGTAAAGAATCCCTTTATCTTATAGTAAAGAAGAAATTAAATAATTAAGAATTCAATAAAGAAATTACAAATTCAATTTTCAATTCAACTAAAAACAATGAAAAAAAATAAGAAAAATAAATAAGCAAAGATAGTCTATTATTTATATGATATGAAAATAGAGTACTAAAATCGCATTTTGGAATGAACCAAAATACTTGTTTGTTTTGTTACGGCAATAAAAAAACTTTGTAAGACTAACCAATGAGTTAGATTTCGCTACAAGAAAAAGGTTTGTTGCTATAAAAATAGAACAAACCTAAAATACTTAAGATCAATAAAAGAATAGGTCCTAGATCCATGCGACTTAGGATTGGGTTGGGTCAGGTTGAAGTCTTGAAACAGGATTATTTCATGAATTTTCATTTCATGAAATTGGCTGGGATTGGGTATACCAAAACAAAAAAAAAAGTGTTAACTCTTTGATCATGGACAGGAAAAGAGGAAAAATATCATATGTAATTCATTCATGAAAGTGTATGAGGAGAAATGGGTATTTGATCCGAGATTTTACAAATACCAATTGGTTCCGTTGGAATGAATTATTGTGTTTATTTTATTGTTCCTACTACTACTCAAATTTCTATACAAAACAAAAATGGAATGTATTAGGGCTATACGGACTCGAACCGTAGACATTCTCGGTAAAACAGAGAAACTTATTATTATCAAAATGATTCGAACTGTTTCAAAGACCCAACATGCATTTTGTTGCATTGGGCTCTTTCATCAACTGATGAAAAGATCAGTTAGTCCACCATATTTTTTCTTTAGAGGAAGATAAGAAGATAATGAGATGGCTCCATGTGCTCTGATTCATTATTCGTATCCTGATCTAGGAGCAATACCAAAGTGTTTCAAAGAAGGGTGACCTTTATTTAGGTCTGCCTTCGGCCTAGAAAACCTAAGTGAAATGCAGTCTCTATCGCTCCGCTGCAAGAGTAAAATATGAGACTTCATACACCTCAAAGCTCATAGGACGAAAAGAGGTTCTTTTGAGATCCTTATACTCATTATGCCTGGCATTTAATGGACTGGGCTTTTACCTTACAAAGGCAGGTTCTTTTTTATTTGGCACCGGAATTCGCACCTGAACCGGATCAAACCAAATTTGTCAGGCTATTTTTCTCTTGTTCTCTCGAATCTACGGAGTAAGACATCGACTTCTCAAAAAGATCAATTATGGTCATTGCATAATGGACTCCCTTGAAAAACATTGGCGCACGTGTAAACGAGGTGCTCTACCTAACTGAGCTATAGCCCTTGTCATAACCATTTTAACATAGAGACAATTTCTTGTCAAGAAGGGTATCCTATAATCCCACACGATAACTCTCTGATCCGTTTCTATTTACTGGTAAAAGATTTATATTGCTTAGAAAACATATTTTATCTATAATCCATCGAAGTGATGGAGACCCTTTTTGTGGTGATAAATGACCTACTTAACCCAGTGGTTAGAGTATTGCTTTCATACGGCGGGAGTCATTGGTTCAAATCCAATAGTAGGTAGAACTTATTAGATACCAGATTCCATGGTATCTAATAAGTTTTTCTACCCATCCTCTTTTTTTCGTTCTATCATCAGATTAATCAAATTAGATTTCATTGTGTTCAATTTGTGGAATCAAGATGTAGTGTGTAGTGTATAATAAAGAATTCTTTTGATTTTGATTGAATGTATTGACTACTAATAGGAAATAACTTTGACAGCTTCTACTCGTGTCCTAGCTCGTCTGAGAGCTAGATTTGCTTCAATTGCTTGTCTCTTACCCTCAGCTCTACTCAAGTTAGCTTCAGCTATTTCAAGAGTTTGTTGAGCTTCTTGTGGATCAATGTCAGTACTAATTTCCGCACCATTTCCTAAAATGGTGATCTCATTATTACTTATTCTAGCGAAACCGCCCATAAGAGCCACTGTGAACCATCGGTCGTTTAGCCGTATTCTCAAAAGACCTATATCTACAGCCGTGGCAATGGGGGCATGGTTTGGTAATACTCCTATTTGTCCACTATTAGTAGATAAAATAATCTCTTTCACTTCGGAATCCCAGATCATTCGATTAGGAGTCAGTACACAAAGATTTAAGGTCATTTCTTCAATTTGCTCTCCTCTTCTAAGTTAATAGCTTTCGCGGTAGCTTCATCGATGTTACCCACCAAATAAAAGGCCTGCTCGGGAAGACCATCTAATTCTCCGGAAAGGATGAATTGAAAACCCCGAATTGTTTCTGCGAGACCAACATATTTCCCTGGAGAACCAGTAAAGACTTCTGCCACAAAGAAAGGTTGTGATAAGAAACGCTCAATCTTGCGTGCTCTTGCTACAGTTAAACGATCTTCTTCGGATAATTCGTCCAACCCAAGGATAGCTATAATGTCCTGAAGTTCTTTGTAACGTTGTGAAGTTTGCTTAACCCTTTGCGCAGTTTCATAATGTTCCTCGCCAACGATCCGAGGTTGTAACATAGTTGACGTTGAATCTAAGGGATCTACTGCTGGATAAATACCTTTGGCAGCTAATCCTCTTGATAATACGGTAGTAGCATCTAAATGTGCAAATGTCGTGGCAGGAGCAGGGTCGGTCAAATCATCCGCAGGTACATAAACTGCTTGGATCGATGTTATGGATCCTTCCTTGGTAGAAGTAATTCTTTCTTGCAAAGAACCCATTTCCGTACTAAGGGTAGGTTGATAACCCACTGCAGAAGGCATTCTACCTAATAAGGCAGAGACTTCGGACCCTGCTTGAACGAAACGAAATATATTGTCGATGAATAGAAGTACGTCTTGCTCATTAACATCCCGGAAATATTCCGCCATGGTTAGGGCAGTCAAGCCAACTCTCATACGAGCTCCTGGCGGTTCATTCATTTGACCATAGACTAGAGCCACTTTTGATTCTGCAATATTTTTTTCATTAATAACCCCGGATTCTTTCATTTCCATGTAGAGATCATTTCCTTCACGAGTACGTTCACCTACTCCGCCAAATACGGATACGCCTCCGTGAGCTTTGGCAATGTTGTTGATCAATTCCATGATGAGTACTGTTTTACCCACTCCAGCTCCCCCAAATAGTCCGATTTTTCCCCCACGGCGATAGGGGGCTAAAAGATCCACCACTTTAATCCCTGTTTCAAAGATTGATAATTTCGTATCTAACTGTATGAAGGCGGGTGCAGATCTATGAATAGGAGATGTTGTGCGAGTATCGACAGGACCTAAATTATCAACGGGCTCTCCAAGAACGTTGAAAATTCGTCCGAGAGTAGCTCCCCCGACTGGAACACTTAGAGGAGCTCCCGTGTCAATCACTTTAATTCCTCTCATCAGACCATCTGTAGCACTCATAGCTACAGCTCTCACTCGATTATTTCCTAATAATTGTTGTACTTCACAAGTTACATTAATTTGCTGACCGACAGTATCTCGACCCTTAATTACCAAAGCATTATAAATATTAGGCATCTTGCCCGGTGGAAAAATAACATCCAGTACTGGGCCAATAATTTGAGCGATACGCCCTAGGTTTTGTTCTTCAAGTGTAGAAACCACAGGATCAGAAGTAGTGGGATTGCTTCTCATAATCATAAATCATAATAAATATGTCGAAATTCTTTTTTGAAAAGTACTGAATCAAAAATAAATATCCGATAGCAAGTTGATCGGTTAATTCCATAAGAAATAAATGGGAGTTAGCATTCGATTGAGTTGTTACCATCCAATCGAATCCAATTAAATCCTTTACTCATTGAATGAGTCAATTTTCAATTCTTTCTATTGTACTATTGTATTTTTTTTTATTTTGATTTATGTTGTGCGCCTATTCTTCTTTATATACCATATCTGTTCCCTTTTCTAGATGAATTATGCCTCTTTTAACATCTAGGATTTACATATACAACATATATTACTGTCAAGAGAGAGGGGCGGGGTCCTCTATTCTTTCTTTTTATTTCTATATTCTATATTAGTATTAGATAGAATATTAGATATTTCTATTTACTATTTATATGTTATATGAATTTCTATTTCTATATTATATACTTTATCTTTCTATTTTTAATATCTTTATCTTTACTTTAGAATTTATTAATTCTAATTTAGAATTATTTAGAATTCTATTTCTATTCAATTTCATATTTATCTATTTTCATTTTATTTGATGTTTTTTTTATCTTTATTTTTCTATTTTTATTTATTTTTATATTCATTATAAAAATGAGTATGAAGAATAATGAATATAAAAAAAATTAAGAAGGTGATCAATTCTATTAGAAATAGAAATCTTCAAAACGAAGATTGGGTTGCGCCATATATATCAAAGAGTATAAAATAATGATGTATTTGGTGAATCAAATACATGGTCCAATAACGAACCCTTTTCAAATTTTCATTATTCATTAGTTGATAATATTAGTTTAGTTGAATCTTTTTTGAATTGTAAATATTTTTGTCAAAGGTTTCATTCACGCCTAATTCATATCGAGTAGACCTTGTTGTTGTGAGAATTCTTAATTCATGAGTTGTAGGGAGGGACTTATGTCACCACAAACAGAAACTAAAGCAAGCGTTGGATTTAAAGCTGGTGTTAAAGATTACAAATTGACTTATTATACTCCTGACTACGAAACCAAAGATACTGATATCTTGGCAGCATTCCGAGTAACTCCTCAACCGGGAGTTCCGCCTGAAGAAGCGGGGGCTGCGGTAGCTGCCGAATCTTCTACTGGTACATGGACAACTGTGTGGACTGATGGGCTTACCAGTCTGGATCGTTACAAAGGACGATGCTACCACATCGAGGTCGTTGTTGGGGAGGAAAATCAATATATTGCTTATGTAGCTTATCCTTTAGACCTTTTTGAAGAAGGTTCTGTTACTAACATGTTTACTTCCATTGTGGGTAATGTATTTGGTTTCAAAGCCCTGCGAGCTCTACGTCTGGAAGATCTGCGAATTCCCACTTCTTATTCCAAAACTTTTCAAGGTCCGCCTCATGGCATCCAGGTTGAAAGAGATAAATTGAACAAGTACGGTCGTCCCCTATTGGGATGTACTATTAAACCAAAATTGGGATTATCCGCAAAAAACTACGGTAGAGCGGTTTATGAATGTCTACGGGGTGGACTTGATTTTACTAAGGATGATGAAAACGTAAATTCACAACCATTTATGCGTTGGAGAGATCGTTTCTTATTTTGTGCCGAAGCGCTTTATAAAGCGCAAGCCGAAACGGGTGAAATTAAAGGACATTACTTTAATGCAACTGCGGGTACATGTGAAGAAATGATCAAAAGAGCGGTATTTGCCAGAGAATTGGGAGTTCCTATCGTAATGCATGACTACTTAACTGGGGGTTTCACCGCAAATACTAGCTTGGCTCATTATTGCCGCGACAACGGTCTACTTCTTCACATCCATCGCGCAATGCATGCAGTTATTGATAGACAGAAAAATCATGGTATGCATTTTCGTGTACTAGCTAAAGCATTACGTATGTCTGGTGGAGATCATATTCACGCTGGTACAGTAGTGGGTAAACTGGAAGGGGAACGTGAGATGACTTTGGGTTTTGTTGATTTGTTACGTGATGATTTTATTGAAAAAGATCGAAGTCGTGGTATTTTTTTCACTCAAGACTGGGTCTCTATGCCGGGTGTTCTGCCCGTGGCTTCAGGGGGTATTCATGTTTGGCATATGCCTGCCCTAACCGAAATCTTTGGGGATGATTCCGTACTACAGTTCGGTGGAGGAACTTTAGGGCACCCTTGGGGAAATGCACCCGGTGCAGTAGCTAATCGGGTGGCTTTAGAAGCATGTGTACAAGCTCGTAATGAGGGACGCGATCTTGCTCGTGAAGGTAATGATATTATTCGTGAAGCTAGCAAATGGAGCCCTGAGTTAGCCGCTGCTTGTGAAATATGGAAAGAGATCACATTCGAGTTCGAACCAGTGGATAAGCTAGATAAATAAAGAAAATAAGCGCGTATAATTTAGCAATTCCTGTTTGTTCTCCTAATTGATTGCAATGAAACTTGGCCCAATCTTTTCCTCAAAAAAAGAAAGATTGGGCCGAATCGGATAAAGTATAAACATCTTATACTAATCCTATACTATGGGTCTTTGTGTATTTGCATATATCTTTTTTTATATGTACAGACCTTACGATATACAATACGATATACAAGTATATACAAAATCTAAACATAAGAACATAAGAAGATAAGATCGAAGGAAGACTAAACAACTTATCTATTCTATTATTTCTTGTTGGAGCCATAGGCTGAATTATGGATCCTTGGGATTGGATTGGTGGATCATTTTATATTCCTTAGTTTCAGGCCATAGATCAAGCCAAGGGAAGGATTCTTTCTACCCCTATTCTGTATATTGTCTTTTTCGTTCCCTGTTGTAATAGAAACTCATTTTCTTATTTGACTATATGACACGAGATTCTACGAGACGTTTTAATTTATGGGAAGAAACAACTATGTATCTTTTTGATGAGAATTGAAAGTTTTACATGAAAAAAACCTGTCTTTATATATCATATATCTTTTTTTGAGGAAAAGATTCTATCATAATCTCTATCATAATATTGAAATGATTCACCGGACGTTTTTCATTAACAATCTTAATGATTGGATCATATACTTCATTTGAATTCTGATGAGAAATAAAAAGAAAAAAATAGTAAAATGATTTTTTCTTCATCGAATGACTATTCATCTATTAGTATTAGGTTTTTATCAAATAGGGGGCAGAAAGAATCTATGGAAAAATGTTGGTTCAATTCAATGTTGTCTAACAAGAAGTTAGAACATAGGTGTGGACTAAGTAAATCAATGGATGATAGTCTTGATGCTCTTGGACATACCAGTGGAAGTGAAGAAACTATTCTAAATGATGCGGAGAAAAAGATTCCTAGTTGGGACAGTTATAGTTTCAGTAATATTAATTATCTAAATTATTTATTTGATAGCAGGAATATTTGGAGTTTGATCTCTGATCATACTTTTTTAGTTAGAAATAGTAATGGTGACACTTATTCTGTATATTTTGATATTGAAAATCAGATTTTTGATATTGACAATGCTAGTTTGAGTGAACTAGAGATTCTTTTTCCTAGTTATTTGAATAGTGGGTCTAATAGTAGTAATTACTACTATTATTATTCCATGTATGATACTCAATCTAATTGGAATAATCACATTAATAGTTGCATTGATAGTTATCTTCGTTTTGAAATCAATAGTGACATTTACAGTAGTATCGACAGTTACCTTTTTAGTTCCGTTCGTACGGAAAGTACAAGTAGTATTGAAAGTGGAAATTCTAGTATCAAAACTAGTAGCAGTTATTTCAATATAAGAGAAATATCTAATGATTTCGATATAAATACAAAATACAAACAGTTATGGGTTCAATGTGAGAATTGTTATGGATTAAATTATAAAAAATTTTTTAGTTCAAAAATGAATATTTGTGAATACTGCGGATATCATTTGAAAATGAGTAGTTCAGATAGAATCGAACTCTTTATTGATCCTGGCACTTGGGAGCCTATGGATGAAGATATGGTTTCTATGGACCCCATTGAATTTCATTCAGAGGAGGAATCTTATATAGATCGCATCTCTTTTTATCAAATAAAAACGGGTTTAACTGAAGCTGTTCAAACGGGCGTAGGTCAACTAAATAGTATTCCCATAGCAATTGGAGTTATGGATTTTCAGTTTATGGGAGGTAGTATGGGATCCGTAGTAGGTGAGAAAATTACCCGTTTGATCGAGTATGCTACTAATCGATCTCTACCTGTCATTATTGTGTGTGCTTCTGGAGGAGCACGCATGCAAGAAGGGAGTTTGAGCTTGATGCAAATGGCTAAAATATCTTCTGCTTCATATGATTATCAATCAAATAAAAAGTTATTCTATGTATCAATCCTTACATCTCCTACAACTGGCGGAGTTACAGCAAGTTTTGGTATGTTGGGAGATATCATTATTGCTGAACCTAATGCCTACATTGCGTTTGCGGGTAAAAGAGTAATTGAACAAACATTGAAAAAGACAGTACCCGAAGGTTCACAAGAGGCTGAGTTTTTATTTGATAAGGGCTTATTCGACCCAATCGTACCACGTAATCCTTTAAAAGGTGTTCTGAATGAGTTATTTCAGCTACATGGTTTCCTTCCCTTGAATCAAGATTAAAAAAAAAGATTGAAATTCTTCATTTTCAAATAGAAGTATAGCACTAGCTTCAGTTATTTTTATTTGTTTATTTGTAGCGAATACGCATTTAGTTCATTATAATGAAAAAAATAAAACTAAGAAGATGGTGTTTTCTTTGGAGACATCCGTTATAAGTGTAATAAGAGTTAGAAGTTTTGGATAATGATTTTTTGCCCCGGATCCCTTTTTTATTCTACCTCTCTTCCTGATTAGGAATAAGCATCCACAAGATAAAAAATAATTCCTTTCTCCTTCCGAGAAATACGGGAAATAAAAATAAATTTCTCCGTCCTTTTGACATATTCATATATAGAACAACGAAAAATAGATAAAAAAAGATATTGAAAAAAATAGAAAGAAATAAAAAATCTCAAATCAAAGAAATAAAATAGAAATATTCAAATAACAAATAATAAGAATATAGAAGTTCTTTCTCTTTAGCGAAAACCCCCGTTTTTCACTAGAAATCCCTGTTTGTTGGATAAGATTGGTTAAAGTCGGGAACTCATAAGAAACTCTTTTCTATCTTTCCTTTCAAAACAAAAAAGGTGTTTTGAAAGGAAAGATAGAAAGACGATGAAGATAAGGATGGGAGAAGAAGAATCCAATTTATTAAAGTGGATTCTCATAAATATTCGTGTAGAAAGAGGAATTAGGTACACTTCATTGAGTTCTACATTCGTTATTGATTATGAAATACTTCATATTAATATTCTATCTAATAGATAGACTTATCATAAGATATATTTATAATTATAATAATAATAGGTACAAATATGAAATTGAGGTACCCATTCTATGATAGATTTTAACCTTCCCTCTATTTTTGTTCCTGTAGTGGCCCTAGTCTTTCCGGCAATCGCAATGGCTTCTTTATCTCTTTATGTCCAAAGAAATAAGATTGTCTAAATATGATGGGACCAAATCTCATCAATTTATTTCAAAACTGGATCATCATACAGATACTTTTTTAATGTAATATGGTAGGATATATGATATGTGGCTTTTCCGAAAACAAATGGAAGAGTTGTTTTGTTATGTATGCCAATGCATAATATACGCATTAATGCATGTATATGCGGTTATAGCTTAATAAATTAAATGATTAAATTCAAAATGATCAGCAAATCTTTTTTGAAAAATATTTGAAATAGAAACTCGATTTATCTAACCAATTCTTCCTAATAGTTCCTGTTGGAATGCTGCTAGTTGATGAAAGTTACTTCGGGATCAAGCAATAAAAGTCGAGTCAAATCCTTTTGGATTATTCTCTCAATTCCAATCGAATGCAACTGGATCTAGTATAGTATGAACTGGCGATCAGAACATATATGGATAGAACTTATAACGGGGTCTCGAAAAACAAGTAATTTTTGCTGGGCCTGTATCCTTTTTTTAGGTTCGCTAGGATTCTTAGTGGTTGGAACTTCCAGTTATCTTGGTAAAAATCTGATATCCGTATTTCCGTATCAGCAAATTCTTTTTTTTCCACAAGGGATCGTGATGTCTTTTTATGGGATCGCAGGTCTATTCATTAGTTCTTATTTGTGGTGCACAATTTCATGGAATGTAGGTAGTGGTTATGACCAATTCGATAGAAAAGAAGGGATAATATCCCTTTTTCGTTGGGGATTTCCTGGAATAAATCGTCGCGTCTTCCTTCGTTTCTTTCTGAAAGATATCCAATCAATCAGAATGGAGGTGAGAGAGGGTCTTTTTCCTCGTCGTGTCCTTTATATGGAAGTCAGGGGCCAAGGAGCTATTCCCTTGACCCGTACTGATGAGAAATTGACTTCACGAGAAATTGAACAAAAAGCTGCCGAATTGGCCTATTTCTTGCGCGTACCCATTGAAGTATTTTGAAATGAACTGAAGAATAAATCTCAGCATGAGAGAAGGAACTTAATACATCTCAAAAGCAGGAGGACGTATATGGAACAATATTAAGAAAATCTAATAGAATCGACGAATGAAACAGGTTCATTAACAATTTACATCATGGATACAGAATGAAAAAAAAGAAAGCATTGGCTTCCCTCCCATATCTTGTGTCTATACTCTTTTTGCCCTGGTGGATTTCTCTCTCATTTAAGAAATGTCTAGAAACTTGGGTTATTAATTGGTGGAATACCAGGCAATCCGAAATCCTTTTGAATGATATTCAAGAGAAAAATGTTCTAGAAAAATTTCTGGAATTAGAAGAACTATTCCTGTTGGACGAGATGATAAAGGAGTACTCGGAAACACATATGCAAAGACTTCGTATAGGAATGCACAAGGAAACAATACAATTGGTCCAAAGACACAATGAATCTCATTTCCATATCATTTTGCATTTCTCTACAAATCTAATCTGTTTCGCTATTCTAAGTGGTTATTTTTTTCTGGGTAATGAAGAACTTTTCATTCTAAATTCTTGGATTCAGGAATTTCTCTATAACTTAAGTGATACAATCAAAGCTTTTTTGATTCTTTTAGTTACTGATTTATGGATCGGATTTCACTCGACCCATGGTTGGGAACTAATGATTGGTTCGATCTACAGCGATTTTGGATTGGCTCAGAATGATCAGATTATATCTGGTCTTGTTTCCACTTTTCCAGTGATTCTAGATACAATTGTGAAATATTGGATCTTCCATTTTTTAAATCGTGTATCTCCTTCGCTTGTAGTAATTTATCATTCAATGAATGAATGAATAATTCATTAGATCTTTTTCTTTATACTTCTACCTTATTCACTTCAAGGTATTCATACTTCATACTATAGTACAATTCTTTCAGTACAATCAATACAATGGCAAACTTGTGGATAGGGAATTCTCCTAGATACCTATCAAATTTATTGTAGAAATTCCGGGAATCAATGATTGGACCATGCAAAATAGAAATACTTTTTCTTGGGTAAAAGAACAGATGACTCGATCCATTTATGTATTGATCATGATATATGTAATAACTCGAGCATCTATTTCAAATGCATATCCCATTTTTGCACAGCAGGGTTATGAAAATCCACGAGAAGCAACTGGGCGAATTGTATGTGCCAATTGCCATTTAGCTAATAAGCCCGTGGATATTGAAGTTCCGCAAGCTGTACTTCCTGATACTGTATTTGAAGCAGTTGTTCGAATTCCTTATGATATGCAACTGAAACAAGTTCTTGCTAATGGTAAAAAAGGAGCTTTGAATGTAGGAGCTGTTCTTATTTTACCCGAGGGATTCGAATTAGCCCCCCCCGATCGTATTTCTCCCGAAATTAAAGAAAAGATGGGCAATCTTTCTTTTCAGTGTTATCGTCCTAATAAAAGAAATATTCTTGTGATAGGTCCTGTTCTCGGTCAGAAATATAGTGAAATCGTCTTTCCTATTCTTTCCCCCGACCCTGCGACGAAAAAAGACGTTCACTTCTTAAAATATCCCATATATGTAGGTGGGAACAGAGGAAGGGGTCAGATTTATCCTGATGGTAGCAAGAGTAACAATACAGTTTATAATGCTACATCTGCTGGTATAGTAAGCAGAATAGCACGTAAAGAAAAGGGGGGATATGAAATAACCATAGTTGATGCATCAGAAGGACGTCAAGTGGTTGATATTATACCTCCAGGACCAGAACTTCTTGTTTCAGAAGGTGAATCCATCAAGCTTGATCAACCATTAACAAGTAATCCAAATGTAGGAGGTTTTGGTCAGGGAGACGCGGAAATAGTGCTTCAAGATCCATTACGAGTCCAAGGCCTTCTTTTCTTCTTGGCATCTGTGATTTTGGCACAAATCTTTTTGGTTCTGAAAAAGAAACAGTTTGAAAAGGTTCAGTTGTACGAAATGAATTTCTAAATCTAGAGATTCCTTAAAATAAAGTTGGTAAAAGTGCCAAATTCTTGTTGATTGATAGAATGATATATGATTCAAAAAATTATATAAGTCTTTTCTTTGTTTTTTTTTTTTTACTCTTTTTTATTTTGCGGGATGTCTGAAACTCATTACTTGTATACCATTCCTAATGATAGAAAATCAGTATACAAATAGAAAGGAATAGAATACAAGGCAAGGAGGACGGCAAAAATGAAATTTCTAGAAAGTATTCTTAGTCTTCTAGAACTCCTTAAATTCACTTCAACTTATAACTTAGAAAAATAATTCAAACAAAAAAAGACTTATCTTGTTTTGTTTCGGACGAAAAGCGGATTAGATCTTTACGCATATCCAATTTTTTCTTTATTATCTCATTACAGTTTTCTTTTTTTCTATTTCTATTATTTTCTATTTCTATTATATTATTCTATATATATATATATATATAGAATATATAAATATAGAATAGAAATATAGAAATAGAGTTTTTTTCTTTTTCTTATTTGTTTTAGTTTAGTAGAAATAGTTGAGTATAAAAAGAAAAGGATTTGCAGGATGTTTCATACGGATAAATCCATACGTATTGGATAATCGATGGATTCGATTCCTCCTCTCTTGTTGCTTCATATTCAAAATATTGACATAATAGTGAATATTATATAGGAACGAAAGAAACTATGAATCAGTCAATAGATTAAATTATTCAAAAACATCATAATAAAAAAAGAATAGAATAGAGTGGTTTGAAACATCAGAGCATAAGGATCCATTTTGTCATTTCTAAAATAGAATATTTGGATTATGTTAACTGAGGTTACATATGTTTTTGAATAGATCAAAGTCTCGCTCTAAGAGTAAGAACTCAGCGGGGTAAGGCCCCACTGAGTTCTTACTCTTTCATGTCTACAATCTAATCTGGTTCATATGATAGTATTACAGAGATGAACCCAACCCGGAATAGGAGCCGTAAAAGAAAATACCTATTAAACCGATCACAGGAATACCAGTTACAGTACCTATCAGCCAAAGAGGAATCCTTCCAGTAGTATCGGTCATTTCCCCCCTTCTTTTTCATCAAGTGGTCATGCTAGAGACAAAAACAGTCATGGATAATTATGAGGATAGTATCCTTCCGAATGGGATAAGAGAATTTCTACTATTTCTATTTCTTAATTGAAGAAATAATTGGAAAATAAAACAGCAAGTACAAAAATGAGTAATAAACCCCAGTATAGACTGGTACGATTCAATTCAACATTTTGTTCATTCGGGTTTGATTGTGTCATAGCTCTATAATTCGAATTAGGTTTATCGTTGGATGAACTGCATTGCTGATATTGACCCTAAAAAAGAAACAGTAGGTACAGCCAGTCCGTGAACAGCCAACCATCGCACTGTAAAAATTGGATAGGTTCGATCTATGGTCATTGGGAACCTCCTAAAAGGATTTACTAAATTCATCGAGTTGTTCCAAAGAATCAAAACGGCCAGTTATTAATGGAATCCCTTGTCGGCTCTCTGTGAAATATTCATTTGGCCGAGGACTTCCAAAAACATCGTAAGCTAAACCCGTACTGACGAATAACCAACCCGCAATGAATAGGGAAGGTATAGTAATGCTATGAATGACCCAGTATCGAATACTAGTAATAATATCAGCAAAAGAACGTTCTCCCGTGCTTCCAGACATGCTGAGCTCCACAAATTCTTATATGTTTCAAAAAAAAGGCGGACCGATTCCGTGAAAGATGGAATCAGTAAATTTAAAACTACTGATACTGGATCTTTGTGAGATCGTCAATTTTGTACCAAAGGTGTATTTAGAGTAGACCGAATCAGTATAGCTATCCTCCCTCTAGCGCAGCAACGCAGCCTCGATCATTACCGAAAGGAAATGCTATTGCTATATCTTCCTTGTCTATGTATAAATGTATTTTCCTTAGAATATTAGAATAATAAGATTAAGTAAGGTTTCTATTAGTGGATTTATCATAGTAATAGAAAGTAAAGATCTTGAATGGATGGGCTCTAATAATTAATGAGAGATATCATGATTGAAAGATCTCATTATATTCATAAAATTTCATTATATGTTATTTTAAATCTATAAAACCTTTTGGTGGTTCGGTTCATATTTTATTTTTTTGAATCCTCTCGTTTTATTCAAGTAATTGGTAATTGTTCGTTCATAGAATAAATATGCTAATACTATTTCATTTTGAACTTCTAAACTGAAGCTATTATTACTATTCTAAATAATAATTATTCTAAATCGAAATTCTTATTACTATCCCTATCTATTTAATTCTTTACTATTTCATTTTTCATTGGTTAAATGGAATTAATATATTAGAATTAGATTTCATATTTTTTATTATTCTTTTTTATATTTAGTATTTTGAATATTTGCGAAAAGAAAAAAAAGAGATCGTTGGAACAATCCATATTCGTGATGCAACTGTTGTTACATTAGATCTCCCCAAGAGTTCTTTCCTTATGGAACTACAAAACAAAACAAAGATGGGATTCTTTAATATGGAAAGAATATAGAACCTAAAAGAGTAATAGAAGTTGCTATTCTTTGAATCGAGTTGGACCAAAATCAAATTCAAATAAAGAAATAAAATAAAAATCAAATGAAAATATTCAATAATTTTCAATTTTTTAAAAAAGTCAAGGTTTTCTTTTTTTTTAGCGTCCGTCTATAATGACTGATGAATCAAGAACTTTCGATTGGAACTAAACGAATTCTTTAAATTCGTTTTTATTACCGTCATATCTGGATTGAAACTTAGGTAAATGTTTTATTCATATATGTATTAAAAGAACATATCTAATTTAGCTCTTTCATGCCTATTCTAACTAGTTATTTTGGTTTTTTACTGGCTGCTTCAACTATAACCCCAGCTCTATTTATTGGTTTGAACAAGATACGACTTATTTGAAATGAATGAAATTCAATAAACAATTTACAAAAATCAAAATCAAAGCCTCCCAGAATATTTTATTTCAGGTATTCTATGGTTCTCAATTGTAAATTCCCGGTCATTGAGATTCACGGATAATTCAGATTAATATTTAGGGATAGATCTTACCTCTCTTTTTATTCCCTAAAACAAATTGAAATGATTGAAGTTTTTCTATTTGGAATCGTCTTAGGTCTAATTCCTATTACTTTAACAGGATTATTTGTAACTGCATATTTACAATACAGGCGCGGTGATCAGTTGGACCTTTGATTGAGTAACATTTCTTTTTTTGATTGACCTCCTACAAGGAGGAGGTCAAATTTAAGTTGCAATTAGACTTTGTTTTGTTAAGTTATTTCATTGTAATTCGACATAACATAAACGAAATCACGCTCTGTAGGATTTGAACCTACGACATCGGGTTTTGGAGACCCGCGTTCTACCGAACTGAACTAAGAGCGCTTTCTTATATCGTATAACAGTAGATACGATTGTAAAGTGAAAGAAAAGTATTTTTTTACCCCCGAGGGGTCTTGTGTACATGTACATATAGTATGTACAAACGAAAGATTATGTCCAAAATCTCCCGATCTTACTCAATGAATCCCTCGTAACTGTCCATAGGAGAAAGAATAGGTAGGGATGACAGGATTTGAACCTGTGACATTTTGTACCCAAAACAAACGCGCTACCAAGCTGCGCTACATCCCTTTTAAAAATTGTTGTACAGTGTCATTGTATAAAATACATGTTTTGTTTTCCACATCCTTCTTTTTTGCTCTACCTATCTATATAGGTAGAGAATGTTCTTGTCATTTTTTTAGGGAGCGGCAAAATTGAATATGCTGGGTCATTGTACATATGCATTTTAGTTAGTAATTCCTAATTCTAATTTTATTTCAAGCAAAAACAAACGATCTTGAACTAAAATATCGGGTTATCTATGATCTATGTATTAGAATATCGAACTAGGACTATATATGTATTACAATATACAATACAAATAAAGAATTAAAATAAATAAGAAAAAAATAGAAAATAAAAGAAGAAGGAGGATTTTCAATGCGAGATATAAAAACATATCTCTCAACGGCACCTGTGCTAACCACTCTATGGTTTGGGTCTTTAGCAGGTCTATTGATAGAAATTAATCGTTTATTTCCGGATGCCTTGTCATTCCCCTTTTTTTCATCCTGATTGAATTCTTGTATTGATCTGTGAAGAAATGAACGAAGATTTGAGATACAATTCTACGTAACATGGCTCCAATTTCGCTTCCTTTTTCTTTCCTATCCTAAAAAAAAAAAGAAAGAGAAAGAGTGTATTGAACCTCAGTCAAAATACAGTGAATCTACGATAGAATTTGGGGGGAAAGAAATGGAAATGTGGATCCAGTCTAGGGGCGACGAAATTTTAACATAGAAAGAATAAAATACTGAGATTAGGATAGGAATAATTCATAGTTAGAAAAAATTGTATTAATTAATTATTACGATATTCTTAATATTCTTCTATTAATGAATATGACTCTTTTTCTTTATAGTTATAGTAATTCTATTTTAGATTATAGTACTCCGAAGTCATTTGAATTCTAATAATTCGAAAAAGAAAATATTTACAAATCCCATTTCTAGTTAGTAACTTTTATTAATAGAGTCTAGATATAGAATATAGATTCTTTTTTTTTTATTTTTATTTGGTTCGGATCAAAAAGAAAATGAAGAGAGTTCTAAAGTGAAGTCGATCCAAAATGAAAAGGAGGTTCATGGCCAAGGGTAAAGATATCAGAATTATAGTGATTTTGGAATGTACCTGTTGTGTTCGAAAGGGTGTCAATAAAGAATCGCCGGGCATTTCTAGATATATTACTCAAAAGAATCGACACAATACACCCAACCGACTAGAATTTAGAAAATTTTGTCGCTATTGTCAAAAGTATACGATTCATGGGGAACTAAAGAAATAGGTGGAACGGAATGTGTGTGTGATTTTTCCAAGTAGCGAGAAGAGTAAGAACTTTACATCTTAACATATATAATACAAACCAAATACTATTTTGGTCGAATCTTAAATGAATAAGAAAAAAAGAGAATTCTATTTTATAGATTCTTTTATATAGAAGGAATAAACAAACAAGGAATAAGCAAACCATGGATAAATCCAAACAACCTTTTCGTAAATCCAAGCGATCTTTTCGTAAGCGTTTACCCCCAATTGGATCGGGGGATCAAATCGATTATAGAAACATGAGTTTAATTAGTCGATTTCTTAGTGAACAAGGAAAAATCTTATCTAGACGGACAAATAGGTTGACCTTGAAACAACAAAGATTAATTACTATTGCTATAAAACAAGCTCGTATTTTATCTTTGTTACCTTTTCGTAATAATGAGAAACAATTGGAGAGAGTGGAGTCGATCCCTAGAACTACTGGTCCTAGAACCAAAAATAAATAGATATACTCCTAAAAACTCCAATCGGAACTCAAGCTTATATTAATGTTTTGCTCGAAAAAACTAGAATCCAGATTTGATTCTTGTATTATAAAAAAGGAAAAATGAGAAAGAAATCTTTTTTTATTGAAAAATGTTCGTTTATTCCTACTACTCAAATCTTAATTTCTTTTTTTCTATCTTCCCGGAGTCCTTTCTCCGGGAAATCCTGTTTCAATCATTCTTGTTTCATGTATAATAGATTCTATTAATATTCTTTTATTCCTTTATTTGATTTGTATTTTTTTTTTATTTTTGATTGATGATTTTATTTGAAATAATATCAAAACAATTCTTATTTGATAGGGCTATTTGCACAAGTATTTTACGATTCAGAAGCAATTGTCTCTTGTACAGATTGTTGATAAATAGGCTATAACTATAGAATAGCCTATCTTCACGAGTTACCGCATTTATCCGAGTGATCCACAAACGACGAAAATCTCTTTTTTTCCTGCTCCTATCTCGATGAGAGGAAACCAAAGCTCTCATTCTTTGTTGAGTAGTCGTTCGAGTAAGTCTTGAATGAGCCCCTATAAAGGTTGATGCAAATAAACGAATCTTTTTTCGACGTCTCCGAGCTGTATATCCTCGTTTAACTCTGGTCATTGAATCAAATGAAACTTTCTTGAATAACTAATTGATTTCTCTTCTTTCAGTCATCCTTTTCTTCCGGTCAATTAATAACAAAACGGATTCTTCCGATATATAAAATATAAATTCCAATGGCTTTTGCGACTATGACCTTCCCGACCACGATTTTTTCTTTCTTCAAGGTATCTCGCCTGGAAATAAGAAATTCGACTACTACTAAATAAAAAAGAATAGTGGGTTTCCTCGTTTCTATGGCAACTTCTGAAACGGTGAGGTCCTCTCTATACACCGGAGCCTCTTCTTTCATTTCATCGAATTTTATTGTGAACTTGTATAGTTCACACTCTTTGGCTCTACCCATCCATTTTTCAATTAGAATTCTTTTTTCAATTCTAATTAGAAAGTAATAGTCCTTTTCACAAAAAAAGCTATCCATACAGTGACGGCATTTAATTCTGAAAGTTGGCTAGGTAGCTGACCCTGTTAGTCCGTTTTTTAAAGAAAAGGAATAGGAGCATAACCTTTTTCCTCCGCTTAATGGATAACTATTTGTTACCAATGGAGAATTCCTTTTCATCTCAAACGGAGTGATTGGATTTGCACCAATAGAAACCATAAATTCATAACATAATTAGGTAGATGATAGATCTTCATTTTTAGATACTAGTAAATTAAATGGCCGTCTTCCACTCTATCTATCCTAATTCATTGATAGTGGTCGTTGATACTTTTGCATTTCTTCAAACTCATCATAATCTGAACTGAACGAGTCGCACATACACCCTAGTACATGTTCCTCGACGCTGAGGACATCCTCGAAGAGCGGGAGATTTCGTGACATTTCTTATTGGCTGTCTTGCGTTTCTAATAAGTTGTTTAATGGTTGGCATGGCGTGTCTATAGAATCTCATTCTAGATAGAATAGAGCGGGTTGGCTTAGATCAATCTTAACCTGATGGTTGATGATTATGAATGATTTCTATTCACACGGAAATTTCGAATTTTTAAACGGAATTCGTATATTTATCCCTAGTATTTTCATTTTCGATCGCTACAAGATCAACGATGCCATGAGCTTGGGCTTCTGTTGCTGACATAAAAACATCCCTTTCCATATCTTCGGATATAACCCATAAAGGGTTGCCCGTTCTTTGTACATAAACTTTTGTGAGAGTTTCGCGAAGCTTCAATAGTTCTTCTGCTTCCAGGATAAAATCCCCTGCTTGTGCCTCGTAAAAAGAACTAGCAGGTTGGTGAATCATAACCCTGATGATATTGATATAACATCATGAATGGCTCTTCTATCTATATATCGCACGATTGGGTTAAAGTAAGGGGTAATCAAAATAACAATAAAAAATAGAATTAAACAACCGTACGGGCATCTTTTGTACATTGCATACGGCTCTGCAATGGAATTTATTTTTTCGATAGAAAAAATTCTATCGAAAAGAAGAAAAAGAACCCATCCGATCCAAATCGTTAAATGATCCATTTACCACCCTTCCTTTCGTAGTAGTAAAAAAGATACTATGATGGTTCTGTTGCTTTATATGTTTATCTATTTATCTCGTCTGTGGTTTAGCAATCCCAAAGTTTCTTTTTGATATGATCCAAGAAGGATAAAATGATTTTTTCCATTTTTTTGACTCTTTCTCTCATAACATAAAAATAAGAAAGATACTTCTGGTGTGGAAGAATAATGGTTTGTGACGCTGAAATTGACTCTTGCTTGACACATAAAATCAACTTAGGAATAACCCTTCTTTCATACTACTATCTCGATACAAAATCTCATGTTAGAAAAAAAACACTAAGGGTTTGTTCATATCGAACTCGAAGTGCCATGCTATTATTACTTATTCTTTATTTGATTCATATTCGATACAGCGAAGGCATAGTATTTTTTTCTCAAATAAAAAAACTCATTGGCGCCAAGCGTGAGGGAATGCTAGACGTTTGGTAATTTCTCCTCCAACCAGAATGAAAGATCCCATTGAAGCGGCTAATCCCATACATACTGTATGTACATCCGGTGACACAAATTGCATAGTATCATAAATGGCTATTCCTGGTATTACCCATCCGCCTGGAGAATTTATAAACAAATAAAGATCCCTAGTATCATCTTCTATGCTGAGATATACCATGAGACCAACAAGTTGATTCGAGATCTCGCTATCAACCTCTTGACCTAAAAAAAGTAATCTTTCTCGATGAAGTCGGTTGATTAGGGAAAAATTGTATCCCTGAGGAACCGTACGTGCACCTTTGGATGCATACGGTTCGAAAGAATTGCGAAAAAAAAATCAATGTATTGATTCCAGTCTTATTTCTTTTTTTTTTAACATTAGTTTTGCCCTCCTTCCCCTTCCCTATATTCTATAATGTAGAAAATAAAAAAGAATTTTATGAACTTATTGAACTAACTTCTCATTGATGTATTGTTTCATCGAAATTCAAATTACGATGTAATTTTCTTGTTCCTGAATGGACCCTTTCAATTCTTTTAGGTTCTTGTTCTACTCCGGGGGAAGATTTGCCCGAATTCCATTTGCGCATATAGGTCAAATGATTCCAGTACCACTTCTTTTTTTTTTTTCAATTGTTTCATAACTTTCCCCAAAATATTCGATGTATTAATAATACTACTCCATTGGTAGGCAGTTTTATACTATCCCTATAGTAAGTATAGGAATAGTCTATGATACAAGTGGTAATCGTGTAATCATCATATATTCTACATAATAGATTCTATTAGAATATTCTATTATAGTAAGTTATATTATATTCTATTTAATTATTAATGAATTTCATAATTTCTTAATACTTTAATTAAATTTATATTTTATTTTTATATTCTTTATTTAATGTTTCTTATTTAATTATCTAATATTATTCTATTTTTTCTATTTAATATTTCTTATTTATATTACTACATTTACACTCCCATTCTATTACTTTTACTCTTACCTTTTCCAATTTTGTATTCTCTAAACGGAGCCTGGATACTTTATCAGTCCAAGTAAACCATCAATTATATTAATTGATAATATTCATCCCCAATAGGAATTATTGTGCTTCACGCTCCGAATTATTGATTGTTCAATCAATACAAGATTGAATATCTATTTATTGGATTGGGCGAAATAGAGAATACTCGATCGGGGGAGATAATGGGGAAATACCATATGACCCATATGTCTGACAAGTCGCACTATACGTCAACCCAAGCTGCATCTTCCTCTCCAGGATTCCGAAAAGGTACTTTTGGAACACCAATGGGCATTAAGATAAAGAAAAAATGAAGTACTATACTTTACTTTAATATGGAAACGTAACAATGGGTTTTATTGTCTTCATCATTTTTTCTCTTTCTTTTCTATTTTTATATCTTATATATATAATATAAGTATTAGTAGTATTAGCATATTTCTATATTCTAATCTAATATATTCTATATATTCTAGAAAATAAAACTTAATATTATATATATAAGATATATTATTATCTAGATAGAATTAGAGTATAGTATATATTAAGTATATAGATTCTAATTTAGAATATTAGTATATAGATATATACTTTATATATAGGAATATAGGACTGGAAGGTTCATAGAGGAAGACAGAATGAATAAAGAAAAATTGTAACGAACGGGATCGATCGGATCAGCCGATTGTTCGAATGATTTCGAATTATAAAAAGTATCTATGCATTCTTTTTCCCTCAAAATCCTCCCATTGCGTATTGGTACTTATCGAGTATAGAATAAGATCTGTTTCTCTTTGTTCTTTTAAATAGAAAGAAATAGAATTGTTTCCTTCTCTTTCTATTTTCAAATTCTTTCTATTCTATTTCATTAAAAATAAAAGAAGGGAATACAAATAAATATAAATCTTTTCCTATACAAAATCTACCGAACAGGTGAAATACACGGTCTAGTCTTTTCCAATGCGATAAAGTTACATAATGTCTATTTCTTTTTCAGAAAGGGGTATTTACATGGGTTTACCTTGGTATCGTGTTCATACTGTTGTATTGAATGATCCCGGTCGATTACTTTCTGTCCATATAATGCATACAGCTCTAGTTTCTGGTTGGGCCGGCTCGATGGCTCTATATGAATTAGCGGTTTTTGATCCCTCTGACCCTGTTCTTGATCCAATGTGGAGACAAGGCATGTTCGTTATACCCTTCATGACTCGTTTAGGAATAACCAATTCGTGGGGGGGTTGGAGTATCTCGGGAGGAACTATAACGAATCCGGGCATTTGGAGTTATGAAGGCGTGGCAGGGGCACATATTTTGTTTTCTGGCTTGTGCTTCTTGGCAGCTATCTGGCATTGGGTGTATTGGGACCTAGAAATATTCTGTGATGAACGTACGGGAAAACCTTCTTTGGATTTGCCCAAGATCTTTGGAATTCATTTATTTCTCTCAGGAGTCGCTTGCTTTGGCTTTGGTGCATTTCATGTAACAGGCTTATATGGTCCTGGAATATGGGTGTCTGATCCTTATGGACTAACTGGAAAAGTACAATCTGTAAACCCAGCGTGGGGTGCGGAAGGTTTTGATCCTTTTGTTCCGGGGGGAATAGCTTCTCATCATATTGCAGCAGGTACATTGGGCATATTAGCGGGTCTATTTCATCTTAGTGTCCGTCCGCCTCAACGTCTATACAAAGGATTACGCATGGGTAATATTGAAACTGTGCTTTCCAGTAGTATTGCTGCTGTTTTTTTTGCAGCTTTCGTAGTTGCTGGAACTATGTGGTATGGTTCAGCAACTACCCCAATCGAATTATTTGGCCCCACTCGTTATCAGTGGGATCAGGGGTACTTCCAGCAAGAAATATATCGAAGAGTTGGGGCCGGACTAGCCGAAAATCTGAGCTTGTCGGAAGCTTGGTCTAAAATTCCCGAAAAATTAGCTTTTTACGATTACATTGGTAATAATCCGGCGAAAGGGGGATTATTCAGAGCAGGCTCAATGGATAACGGGGATGGAATAGCTGTTGGGTGGTTAGGGCATCCCATATTTAGAGATAAAGAAGGGCGCGAACTCTTTGTACGTCGTATGCCTACCTTTTTTGAAACATTTCCAGTAGTTTTGGTAGATGGAGACGGAATTGTGAGGGCCGATGTTCCTTTTAGAAGGGCAGAATCCAAATATAGTGTTGAACAAGTAGGGGTAACTGTTGAATTCTATGGTGGCGAACTTAATGGAGTCATTTATAGTGATCCTGCTACTGTAAAAAAATATGCTAGACGTGCCCAATTAGGTGAAATTTTTGAATTAGACCGGGCTACTTTGAAATCCGATGGTGTTTTTCGTAGCAGTCCAAGGGGTTGGTTCACTTTTGGGCATGCTACGTTTGCTTTGCTCTTCTTTTTCGGACACATTTGGCACGGCGCCAGAACCTTGTTCAGAGATGTTTTTGCCGGTATTGATCCAGATTTAGATGCTCAAGTGGAATTTGGAGCATTCCAAAAACTTGGAGATCCAACTACAAGGAAACAAGTAGTCTGATACAAAATTCCTTTGACATCTTTTGCTTCTATTTTTTTTTTTATTTTATTCTAATATTTAAATATTTAGAGTATATAAATTTAGATTTCTATTATATTTATTATTTATATTATATATTTATATATATAGTATTTAGCTATTTAGTATTTCTAATTTGTTAATTTCTAGAATTAAGCTAGAATTTCTCTTTTCTATATTAATTGAATCTATTATATATTTCATTTCATATTCAATATTTCCTAATATATTAATCTAATTATTAATCTAATATACTAATACTAATATATAAATTAGATAAATATCTATTTATTTTCTATTTTCTTTCTATATTTTTATTATTTTTATGTATAAATAGAATAATATATTCTATTAGACTATTAGAATAATATATAAAAAAATTAGAAATAGAATAAGAATATTACAATATAAATATAGAAGAAATAGAATTAATAATATATGACTATATCTATATATAGTATATATACATACTATATAGATAGTAATAGTTAGTAATAGTAAATTGTAAATCTCAATTTAGAATTTATTTAGTAAATGATCCAAAATGAATAGGTGTGGAAGCTATAATTGTAAACCACGATCGAATCTATGGAAGCATTGGTTTATACATTCCTCTTAGTTTCAACTTTAGGGATAATTTTTTTCGCTATCTTTTTTCGAGAACCACCTAAAGTTCCAACTAAAAAAATAAAATGATTTTTCATTATTTCCATTGAAGTAATGAGCCCCATATTCATATTGGGGCTCATTACTTCAACTAGTCCCCATGTTCTTCGAAGGGATCTCTTAATTTTTGAGAGGGTTGCCCAAAAGCGGTATATAAGGCATACCCAGTAAAGCTTACAAGTAACCCGGATATGGAGATGGCGACTAGGGTTGCTGTTTCCATTTTTTCGATAATTTCAAGATCACGATAATGTCGTTTATTTACAACTACAACGGAATGGTATACAAAGTCAACAGATTTCAACCCATGATGAAAGAGGATTTATGGCTACAAAAACCGTTGAGAGTAGTTCTAGATCTGGGCCAAGACGAACTGGCGTAGGGAGTTTATTGAAACCATTGAATTCGGAATATGGAAAAGTAGCTCCAGGGTGGGGGACTACACCACTTATGGGAGTTGCAATGGCTCTATTTGCAATATTTCTATCTATTATTTTAGAAATTTATAATTCATCTGTTTTACTGGATGGAATTTCAATGAATTAGTTCATAAAAACTAGGGGCTTCCTAGTTTTTCAATCAAAAAATATTATTTTACTTATACTTACTTAATGCTTAAAATACTTAATACTTCAACTTAATATTACCTAAGACTTGTAAGACTTGGATTTCATTCTGGTAGTTCGATCGTGAAATTTATTTGTTTCGATATTTCATTTCCGGAATATGAGCGTGTGACTTGTTATAATGGATCCTATTGATAATACAGAGAATGGACCTGTCATCTCTATCAAGATGATTCTACCTCGTCAGATATTTATTCTAGTCTCTGGAGCACGGACTATATAGAATAGATCAAGAAAAGAAATATTTGAACTATGATTCATACCTATTATTTATACCTCGCAACCGGATTAAAAAAAATGGAAATAGGTCTTTTCTAAATCAAACAATTTTTTCCTTCATACTTCTTTTGACCAAAATAAACCTCTTTCTCTATATTTTGTTGAGTTATTACATTCATTGAAGAAGTGATGATCAAATGGTTTTTACTCAGAAAACCTTTGAGTTTAGCTTTGGCTTTCTTAAATCATCGTGGTTCTAGTATGAATCTGAGGTTTCAATTGATTCATAGGGTCTCAACAAGAGAATTCCTATCAAATAAAAAAAAAAAAACAAAAAAAAAAGATAGGGAAGAGAAGATTCAAGAGGCCTGTCACGATTAACATAAAGAAAGATGGATGAGCCAACTTGAGATTGTATTTCTTGGCATTATCATCACAAAGAAGAGATTTCGGATTTTTCTTACTTCGTATCTTTGGGTCAAATCGAGTCAAGCGGCTAAGCCACAAGAAGTTTGAAACTCTCTATTCCATATCCGTTGAACCCAGTATTTGTGTGTTTCGGCTTGAGCCGTACGAGATGAAATTCTCATATACGGTTCTCAGAGGGGGAGTCTTTCTTGGGTTACCTATCTCAATAAAGTATATGATTGGTTCGAGGAACGTCTCGAGATTCAAGCGATTGCAGATGATATAACTAGTAAATATGTTCCTCCTCATGTCAACATATTTTATTGTTTAGGGGGGATTACGCTTACTTGTTTTTTAGTACAAGTAGCTACGGGTTTTGCTATGACCTTTTACTATCGTCCAACTGTTACAGAGGCTTTTTCCTCTGTTCAATACATAATGACTGAGGCCAACTTTGGTTGGTTAATTCGATCAGTTCATCGATGGTCAGCAAGTATGATGGTTCTAATGACGATCTTGCACGTATTTCGTGTGTATCTTACAGGTGGGTTTAAAAAACCCCGCGAATTAACTTGGGTTACAGGGGTGGTTCTGGCTGTATTGACCGCATCTTTTGGCGTAACTGGTTATTCCTTACCTCGGGACCAAATTGGCTATTGGGCAGTAAAAATTGTAACAGGTGTGCCTGAAGCTATTCCTATAATAGGATCACCCTTGGTAGAATTATTACGTGGAAGTGCTAGTGTGGGCCAGTCCACTTTGACTCGTTTTTATAGTTTACATACTTTTGTATTACCTCTTCTTACTGCCGTATTTATGTTAATGCACTTTCCAATGATACGTAAGCAAGGTATTTCGGGTCCTTTATAGAGAAGGCAGATCATAGATATTTGTAATTTATCATATGGGGGAGGAACAAGAGTCTTTCATTGCTACAAATATGGATTATTTAAAAATAAGGCATGTTATTTGGATACTTCTATTCAACTCTGAAGTATTGTTTATTTGATACGAATCAAATAGTTGAAGTATATTTTCCTAAAAGAGGATGGATTATGGGAGTGTGTGACTTGAACTATTGATTGGTCCATGCAGATATATGATTTTATCCGCCACGTTGGAATTCACAACCTAACGTGTCTCCGCATCCAACCATCACGTCAGTCCCTTTATGTAGCATAGGATAGGCCGGTTCGCTTGAGGAGAATATTTTCTATGATCATACCCGAATCATGTCATGCATGAACAGGCTCCGTAAGATCCCTAGAATAGAATGATCCAATGTTCTATTTATTCCACTTTTTTTGATTTTTCTTTTTTTTTAGTAATTTTCTTATAATTAATTAATAGTATTAATATTAGTAATCTTAGTAAATTTTTTATAGTATGGAGATGCATTCATTTCCTCTGCATCGACCCTGAATTTATGATACTATTGGAGTTAAATAAGGGATCTAAGGAAGAACAGGGGCTAGACTTTATTAGTAACAAGTAAAAATTTTGTATTTTTGTATGTAATAAAATCGAGATGTTGTGGGGATAAATACCAACCAAAATACATAAGACAATCCAAAAAGCACTTGATCATGATCAAATTTGTAAGCCTACTTGGATATTGAGCATTTCCTTGTTGCCAGAACTGAATTCTTTGCAATGAATAATGAATCGTTGAAACTCGGGGAAATGGAATTTGATAAATCTTTTCTTACATAGAGTCATTCATAGAGTCATTCTACATTATATATGTAGATATGTATGAAATATAGATCTTCTATGGACCTATTTATGTTCTATGGATCTATTTCTGTGATTCTTTTGATTCTTGCTCGAGCCGGATGATAAAAAATTATCATGTCCGGTTCCTTTGGGGGATGGATCCACAAGAATTCACCTATCCAAATAACAAAGAAACCTGATTTGAATGATCCTGTATTAAGAGCTAAATTGGCTAAAGGGATGGGACATAATTATTATGGAGAACCTGCATGGCCCAATGATCTTTTATATATTTTTCCAGTAGTAATTCTAGGCACTATTGCATGTAATGTGGGCTTAGCAGTTCTAGAGCCGTCAATGATCGGTGAACCGGCGGATCCGTTTGCAACTCCGTTGGAAATATTACCCGAATGGTACTTCTTTCCCGTATTTCAAATACTTCGCACAGTACCCAATAAGTTATTGGGTGTTCTTTTAATGGTTTCAGTACCAATAGGATTATTGACAGTACCTTTTTTGGAGAATGTCAATAAATTCCAAAATCCATTTCGTCGTCCAGTAGCTACAACAGTCTTTTTGATCGGTACCGCAGTAGCTCTTTGGTTAGGTATTGGAGCAACTTTACCTATTGAGAAATCCCTAACTTTAGGTCTTTTTCAAATTGATTAAACCGTGAAATACCACGACATAGGTATCTAAGGAAGATCCAGAAGGGGATCTTCCTTAGATACATCAATCTTATTATGATCTATTCTGCAAATATATGGACCGTATCGGAGATTAAAAACTTCTTCTATTCTTTGTTTATTTCTAAAAACTAAAAAAAGAAAAAATTCCAATGGATTTAAAATGAAAACTTTTTATTAGATAAATTGATTGCAAAATGCTTCTGTAGAGTGCCCAATATCTGTTTTACATCTTCTATGCGAAAATATTCCATTTTCATAAGATCTTCTTGACTGTGACTCAAAAGGTCCAATAATGTATGTATATTGGACCTTTTGAGACAATTATAGGTCCTGGAAGACAATTCTGATTGGTCAATAAAAATACATGTCAATGGAATTCCTTTTTTGTTTTTCTTGAGATTAGTGAATCTGTCTTTAAAGGAAAAAAGGGGTAGATTCCATCTGTTTTCATTTTCCTTGAAATTCATGTCCTCTTCCTCTGCATGTAGAAAAGGAATCAATAAATCAATCAAATTACGAGAAGCCTCATAAAGTGCTTCTTTAGGAGTTAAACTTCCATTCGTCCATATTTCTAGAAAGAGTATTTCTTGTTTTTCATCCCCATTCCCATAAGAATGAATACTATGATTCGCATTTCGAACAGGCATAGATACAATATCTATAGGATAACTTCCATCGTGAGAGTCGTTTGTGGATTTCATACGATATCCGCGATCTCTCTTGATTTGTAATTCAATACACAAATCAATTGGTTCTGTCAGGTTAGCTATATGCTGTGTCGTGTCAACTATTTCTACAGAAGGTGGTGAGATGATATCTTGAGCTGTTATGTATTTTGGACCCCTGACGCAAATGGATGCGTCCCTAACTCCATAGAGATTACTTCTCAATACAATCTCTTTCAAATTTATTAAAATCTCATGTACTGATTCTTCAATACCTGCTATCGTAGAATATTCATGCAGCACATTTTCAGATGTTGCACGTGTGATACATGTTCCTTCTATTTCTCCAAGTAAAGCCCTTCGCATGGCAATACCTATTGTATCGGCTTGACCTTTCATAAGCGGGGACAAAACGAAACGACCATAATAAAGACGCTTGCTGTCTACTCTTGATTCAACACATTTCCACTGTAGTGTTCGAGTGGATCCTGCTACTTCTTCTCGAACCATACTCTTATTTTTCTTTTATTTATGATTATTGGATCAGATCATTTCCAAGAATCATTTATTTCTCTTGAAATCTCTTGAATTCTTATTTCTACACACGTCTTTTTTTAGGAGGGCGACATCCATTATGCGGCATGGGTGTTACATCACGTACGAAACTTAATAGCATCCCATTTCTACGAATGGCTCGTAATGCCGCATCTCTTCCGAGACCTGGACCCTTTATCATAACTTCTGCTCGTTGCATACCCTGATCAACTAATGTACGAATAGCATTAAATGCCGCGGCTTGAGCAGCATAGGGTGTTCCTTTTCTTGTGCCTCTGAATCCAGAAGTACCTGCGGAAGCCCAAGAAACCACCCGACCTCGTACGTCTGTAACAGTTACAATAGTATTGTTGAAACTCGCTTGAACATGAATAACTCCTTTTGGTATTCTACGTTCATTCTTATTTGAACCAATACGTGCATTCTTACGTAAACCAATTTTTGCTATAGGTTTTGTCATATTTTATTAGATCATATTCATAAGAATAAAATAAAAAGAAAGAAGAATCAGAAATCTACATAAAGATACGGATACAGGAATATCTATTTCATATGAAAACGAATCCTTTCTTCTTTCTTTTTACATGTACATGGTTTTTTCTTTTAAAAAGTTCTTGATTTAGAACTTGAGAAGGATTACCCCTGTCTCTGTTTATGTCTCGGATTGGAACAAATGACTCTAATCCGCCCCCGCCTACGAATCAAGCGACATTTTTCACAAATTTTACGAACAGAAGCCCTTATTTTCATATTTATCATTCCTTACTTTCATTCTGAATCTATTTTTTTTTTAAACAAAAATCAGTTTATTGCATTTTTGAACTTCGAATTATATCTCTACGAAAAGGATGTTTAAAAGAATGATCTAATCATTCGAATCTTTTTTGCGAAGTCTATAAATTATACGTCCCCTGGTTGAATCATAACGACTCATTTCGATTTTGACTCTATCTCCGGGTAGTATACGTATAAAACTGCGCCGGATTCTTCCTGAAATATAACCTAGAATTAGATCTTCATTATCTAACTGAACTCGGAACATACCATTGGGAAGTGATTCAGTAATTAAACCCTCATGAATTAATTTTTGTTCTTTCATTTTCCAGGGAACCCCCTTTAAGTATCAACTAATAGAGGAAGAGTTCTATAATTCATTTCTCCTCTCTATTTTACAAATAGTAAGTTCGAGAGAAAATTAGGGTACCCAGGAGAATCACCATATATAACACAAAATTTCTCCTCCAATTTTTTCTAGTCGAGCTTCTCGATCTGTCATTATACCTCGAGAAGTAGAAAGAATTACAATTCCCATTCCGCCTAAAATCTTAGGAATTCGTTGATAGTTGGAATATATTCGTAGACCGGGTCGGCTGATACGCTTTAAAATTATTTTATTCCCTTTCCTAGTCTTTCTATGTCGTAAGGTTAAAACCAAGAAATTTTTTTGACTTTCTTGATGTTTTCGAACATTTTCAATAAAACCTTCTCGTAAAAGTATTTTCACAATGTTTTTAGTGATATTAGTAGATACTATTTGAACTCTTCCCTTTTGATCTATGTCAGCATTTCTTATATAAGTTAATATATCGGCAATAATGTCCCTACCCATGACGAACTATAGTTATTGGTGCCTCCTAATTTTGATATAATCAACATGCTTCTTTTTTGTTTTATTTTTTATTGAATTTTTTTTTTGAAATTCTTAAATTATAAAAAATTATTAGAAATTTAAAGTATATGCATGAGACACAATCTATTCTATTAATCGGATTTATTTCAGATATTTGAATATTATAAATCTATATATGGAATATATAGATTATAATATAGATTATAGATAGGATATATCTTATTTTTCATCTATAATACTTCGGGTGCTAATGAAACTATTTTAGTAAAATTCAATTGTCGCAATTCTCGAGCAATCGCACCAAAAATTCGAGTTCCTTTTGGATTTCCTTCTTGATCAATGATAACTGCTGCATTGTCATCATATCGTATTATCATACCATTATCACGTTTGAGTTCTTTACACGTGCGTACAATCACAGCTCTAATTATTTCTGATCTTTCTATAGGCATGTTGGGCACTGCTTCTTTGATTACAGCAACAATAACATCGCCAATATGAGCATATCGGTGATTACCAGTTCCTATGATTCGAATACACATCAATTCTTGAGCTCCACTGTTATCCGCTACATTCAAAAGGGTCTGAGGTTGAATCATATCATTTTTATTTGAATCTCTTATTTCAATGCAAGGGATAATGGAAAAAAGAAATATTGTCTGTCCAGAGATAAAGAAATCCGTGGTTGTTTTTTCATTCTCAATACTCCTTCTTCTTCTTTTACTTTTGTTTACCTATCCTGAAATAACAAATTGAGTTCGTATAGGCATTTTGCATGCAGCTATTTCCATAGCAGCTTTGGCTACAGTTTCTGATACTCCACTCATTTCATAAAGTATTCGGTCCGGTTTAACAACAGATACCCAATATTCGGGGGATCCCTTGCCCGAACCCATACGTGTTTCTGTAGGTCTTAAAGTAACAGGTTTGTCGGGAAAGATACGTACCCATATCTTTCCACCACGACGCGCATATCGTGTCATTGCTCTTCGCCCTGCTTCTATTTGTCTAGCTGTGATCCAAGCAGGTTCAAGTGCCTGAAGAGCATATCTGCCAAAACAAATATGATTGCCTCGGCAAGATATCCCTTTCATTCTACCTCTATGTTGTTTACGAAATCTGGTTCTTTTGGGGTTATAGTTGATGGTTATTTCTGAATTCCATCTCTACTGCAAAGCCGGACATGAGAATTTCTTCTCATCCAGCTCCTCGCGAATGAAATGATTCAATAATAATATTATATTATTATATATACACATGTATATATGTATTTCATTCTAAGAAAGAATATACTAATTTTTTTTATATTGAATTTGTTACATAGGTAACTGTATATATAACTAGGCGTGTTTGTTTATATATAATGCTTTTTTCTTTTATCAAAATTTCTAAAATATTTTACTTTACCTCTATTGAATCACGCCAACAGTCATTCAATAAATGAAATTATTAAATTAAATAAAAATAAAGAAAGGTTTCGCGGGCGAATATTGACTCTTTCCTCCTTCATTTGTAGGGTCAATTCATGACCATTTAGAAGAAATCCATTTTTTATTGGTTCATTCCGCCATCCTACCCAATGAATCATTAGGATTGATTCGTTTTCAAGAAAATCCTATGTAATCACAGGTTCCATCGTTCCCATAGCTTCTCTATTAATGCTTAGGCCTGAACTCTGCAATGGAGCTCTCAACAAAATATGTTATTTGTTTCCGAGTCAATCTCCTCAGTTTTTATTAACCCGAAGCTCATTTAAATTATTCTCTATTTTTTATTATTTCTATTATCTATTTTTCTATCTTTGATATCTTATTATTTCTTTATCTATCTTATTACATACATATATAGACTGACTATATTATCCATATTGATTAGATTATTATTTTAATTTAATTTCTTTTATTATATTTCTTATATTTTCTTCTATGTTTCTATTTTCTTTCTATTTTTTATTTGTATATTTCTTATTCTATATGTAATTGTATATTTTGTATTTTTATTTGATGTTGATGCTTTATAACACTGCCTTTTTTATGGGGTAATTCTTCATAAACCATACATATGGGAATCCTATATCATTGAGATCTTTATTCTCTCTTTCTATCATCCTTCCATTTTTCCACATCCCTTTTTTTTCACAATTCATAATCAGATTTCTTTTTTATGAAAAGAATTTCAGTTGCTACAACTATATGATCGATTCAGTCATATAGTGACTGTTTCTTGGGATCTCGACAATACGAAGCAATAAGTTGGTTATTAGTTTTAAGTTTCTTTAGTTTTTATAGTTATTAAGTTTATAGTGGGGTCAGTCTTTTTTTTTTCAATCTCAATTCTCAACTCTAAAGAAAAAACTAACGAGTCACACACTGAGCATAGCAATTATACTAAAATCTAAATTAAATTTAAATAAAGGGTAAATCAAATTTTTATTCAACCTTATAGAATTATAATTGTTCGTTTTTCTTTGATTAAGAAAAAGAAGAAAAGAGTTTCTAAATTTTTTCTATCGATGAGCAGAATGGCGAGATAAAGAAAGGTCCATTATTATTATTTTCTTATTCTTGGTTTACAAATATCCAAATTTTGATGCCTAAGACTCCATAGATAGTTCTAATTGTATGGGAACAGTGATCAATTTTAGCGCGAATTGTTTGTAAAGGAACCCTGCCTTCTCTGATCCATTCGACACGTGCAATCTCTTTTCCGTCGATACGCCCTGCAATTTGCACTTGAATTCCTTTTGTACCCGTTTGTTCAGTTAATTCAATAGCTTTTTTCATTGCCTTTCGAAATGAGACTCTATTTTTTAATTGTAAAGCTATATATTCTGCAAGAATATTAGGTTGTCCATAAGGCTTTTCAATTCTTGTGATAGCAATGTTGAGTCTCCGATTTACAGAATGAAACTCTTTTTGTACATTCATCTGTAATTCTTCGACTCCCCGTGTTCGTCCTTCTATTAATAAATTCGGAAATCCAATATAGATTATGACCTGAATCAAATCTATTCTTTTTTTAATTCCTATATGGACAATTCCTTCTAAACCCGAGGATATTTTCTTATTTTTTTTTACATAGTCCTTAATACAATTCCGTATTCTTTCATCTTCTTGTAGACCCATGGAAAAATTCTTTGGTTTTGCGAACCAAAAAGAATGATGACTTTTAGTTATTCCAAGTCTGAAACCAAGTGGATTTATTTTTTGTCCCATATTTTTCTATTATTTTTCCATCTATTTTTTTTCTAAATACGAATCTAAATTTTATATTTTTCTTTTAAAAAAATCTTTATATGACAAGTTGTTTTTTTTATCAGATAACTACGCCCTCGAGCCCGGGGTCTTAACTTTTTCACAATAGCACCTCTATTGACTTCAGCTTTACTAATAAATAAATTAGCTTCATTCAAACCCATATTATGACTAGCATTTGCTGCTGCAGAATAAACTAATTTTAAAATTGGATAAGATGCCCAATAAGGCATTAGTTCCAGTATCATGAGTGTTTCCTCATAAGAACGTCCGCGAATCTGATCAATTACTCTTCGTGCTTTGAAAACAGACATACATATATGTTGAGCTAAAACTTTTGCTTCTCTATCCGAATTTTCGTTCTTTATCATAAAAGTTCTCCCCCGCCAATGAATGATAAGTGCCTAGGTGAAGTATAGTATAAGATAAGTCAGAAAAGTATAAGTCTTATTAGTATACTAGAAAAAGAAAAGAAATATACCTATACTCTTACTAGTTAAGATAAGGCTTTTCACATGAATACTTAGTAGAACGGCTAACGACGAGATTTATTATCGTTTCTCGCGTGTCTCACGAAAGTGAGAGTAGGTGCGAATTCTCCCAATTTGTGACCGACCATACGATCTGTGATATAAATAGGTAAATGTTCCTTTCCATTATGAATAGCGATTGTATGGCCAATCATTGTGGGTATAATGGTAGATGCCCGAGACCAAGTCACTATGATTTCTTTCTCCTCCCTCCTGTTGAGTTTTTCAATTCTTCCCGATAAATGATTAGCTACAAAAGGATTTTTTTTTAGTGAACGTGTCACGGCTGATTACTCCTTTTTTTACATTTTTAAAATTGGCATTCTATGTCCAATATCTCGATCTTAATCTGAAGTATAATGATGAATGGAAAAAAGAGAAAATCCTTTAGCTAGATAAGGGAAGGGGCGGATGTAGCCAAGTGGATCAAGGCAGTGGATTGTGAATCCACCATGCGCGGGTTCAATTCCCGTCGTTCGCCCATCACATTATTTCCAATTCCAAAAATTCGATTTTCAATGTTCCTATTTACGGCGACGAAGAATAAAACTATCACTATATTTGTTCCTTTTCCTACTTCTTCTTCCAAGCGCAGGATAACCCCAAGGGGTTGTGGGTTTTTTTCTACCAATTGGGGCTCTCCCTTCACCGCCCCCATGGGGATGGTCTACAGGGTTCATAACTACTCCTCTTACTACAGGACGCTTACCTAGCCAACACTTAGATCCAGCTCTACCCAAACTTTTTTGGTTCACCCCAACATTACCCACTTGTCCGACTGTTGCTAAGCAGTTTTTGGATATCAAACGGACCTCCCCAGATGGTAATCTTAATGTGGCCGATTTACCCTCTTTTGCAATCAGTTTCGCTACAGCGCCTGCTGCTCTAGCTAATTGTCCACCCTTTCCAAGTGTGATTTCTATGTTATGTATGGCCGTGCCTAAGGGCATATCGGTTGAAGTAGATTCTTCTTTTTTATCAATCAAAACCCCTTCCCAAACTGTACAAGCTTCTTCCAAAGCATACGGCTTTCTAGATGTATATGATGATATCTAGACAGATGGATCTTATATGATGAAGTACCACATGAGTGGATATATAGGAATCCAAATCTGCCGAATCACTCATGTTATGATCTTCTACATCCTAGGTCTCCCCGTTCCGTCATCTGGCTTATGTTCTTCATGTAGCATTCAGACCGGATGACTCTATGAAATTACGTCGATACTTCCACATATTACGGGTAACGTAGGAGACATCTCTATTTTTCCCCGGGGGGTCTTTCTAATTACCACTGCTTAGCTTTCAATTCGCCTCTGACCATCAAATGAAATGTGAATAACCCGTCCTCCTCTCTTTGAAACAAGGGGTGCTTCCGGTTCTGTGCGCGCTTCAAACAATTTTGTCTTCTCCATATTACCATATCTCTAGAGTCAATAATTTTCTATGAGGAACTACTGAACTCAATCACTTGCTGCCGTTACTCAACAGTTTTCTGTTGAGGTCTATCCCGTAGAGGTACTCCAATTGGATCAGTGATCGATTTCTAGGTTTCGTCGTAAACCTAATTGGTTACTTCCAATTACGTAAATCAATAGTTCAAACCGCACTCAAAGGTAGGGCATTTCCCATTGATATAGGAACTTCTGTACCAGAAACAATGGTATCTCCAATTATAGCCCCTCTGGGATGTAAAATATATCTCTTCTCACCATCCCCATAGTGTATGAGACAAATGTATGCATTTCGATTAGGGTCATATTCTATGGTTACGATTCTACCAGATATCTCTTTTTCATTCCGTCGAAAGTCGATTTTACGGTATAGACGCTTATGACCTCCCCCTCTATGCCCTGCGGTAATGATCCCTCTGGCATTACGACCTTTACCACAACGATGCTGTCCATAGATCAAATTATTTCGTGGATTGGATTTCACTTGACTGTCTACGGCTCCATTGCGTGTGCTCGGGGTAGAAGTTTTGTATAAATGTATTGCCGTGTTATTAAGTCTTTTGCTTTAAGTTCTTTTCTCTATAAGAGGTGGAATAGAATAACCCGGTTGAAGCGTAATGATCATACGTCTGTAATGCATTGTATGTCCCATAATAGGTCCCATCCTTCTACCCTTTCCCGGGAGTCGATGACTATTCATAGCTATTACCTTGACACCAAAGAAGAGTTCGACCCAATGCTTTATTTCTGTCCTAGTTGATCCTGATTCGACATTAGAAGTATATTGATTGTTCCCCAATAACCGAATACTTTTTTCTGTAAATACTGCATATTTGATTCCATCCATAAATCCATTTTCTTCCCTAAGAATTCTAGTTCTTACTGTTCATATGTTATGGTATGAATATACCATACCAATTCGCTATGTATGGATGATGAGATTCCATTGATACAGAGCCAATTCCAATAGACTTATTGAATGTTCCCATTGGCGTGCATCCAGCAGGAATTGAACCTACGAATTTGCCAATTATGAGTTGGGCGCTTTAACCATTCAGCCATGGATGCTTAACGGGGATCATCGTACATCGTGAATAACCAAATTCCAATTGAAATGAAATCTTTAGGAGGAATCAATGAAACGACATCAATTAAAATCCTGGATATTCGAATTGAGAGAGATCAAGAATTCTCACTATTTCTTAGATTCATGGATCAAATTCGATTCAGTGGGATCTTTCACTCACATTTTTTTCCACCAAGAACGTTTTATGAAACTCTTTGACCCCCGAATTTGGAGTATCCTACTTTCACGTGATTCACAGGGTGCAACAAGCAATCGATATTTCACGACCAAAGGTGTAGTACTGCTTGTAGTAGTGGTCCTTATATCTCGTATTAACAATCGAAAGATGGTCGAAAGAAAAAATCTCTATTTGATGGGGCTTCTTCCTATACCTATGAATTCCATTGGACCCAGAAAGGAGACATTGGAAGAATCTTTTTGGTCTTCCAATAGAAATAGGTTGATTGTTTCGCTCCTGTATCTTCCAAAAGGGAAAAAGATTTCTGAGAGTTGTTTCATGGATCCGCAAGAGAGTACTTGGGTTATCCCAATAAAGAAAAAGCGTATCATGCCTGAATCTAACCGGGGTTCGCGGTGGTGGAGGAACCGGATCGGAAAAAAGAGGGATTCTAGTTGTCAGATATCTAATGAAACCGTAGCTGGAATTGAGATCTCATTCAAAGAGAAAGATAGCAAATATCTGGAGTTTCTTTTTTTATCCTATACGGATGATCCGATCCGCAAGGACCATGATTGGGAATTTTTTGATCGTCTTTCTCCGAGGAAGAAACGAAACATAATCAACTTGAATTCGGGACAGCTATTCGAAATCTTAGGGAAAGACTTGATTTGTTATCTCATGTCTGCTTTTCGTGAAAAAAGACCAATTCAGGGGGAGAGTTTCTTCAAACAACAAGGAGCTGGGGCAACTATGCAATCCAATGATATTGAGCATGTTTCCCATCTCTTCTCGAGAAACAAGTGGAGTATTTCTTTGCAAAATTGTGCTCAATTTCATATGTGGCAATTCCGCCAAGATCTCTTCGTTAGTTGGGGGAAGAATCAGCACGAATCGAATTTTTTGAGGAACGTCTCGAGAGAGAATTGGATTTGGTTAGACAATGTGTGGTTGGTAAACAAGGATCGGTTTTTTAGCAAGGTACGGAATGTATTGTCAAATATTCAATATGATTCCACAAGATCTATTTTCGTTCAAGTAACGGATTCTAGCCAATGGAAAGGATCTTCTTCTGATCAATCCAGAGATCATTTCGATTCCGTTAGAAATGAGAATTCAGAATATCACACATTGATCGATCAAACAGAGATTCAGCAACTAAAAGAGAGATCGATTCTTTGGGATCCTTCCTTTCTTCAAACGGAACGAACAGAGATAGAATCAGATCGATTCCCGAAATGCCTTTTTGGATCTTCCTCCATGTCCTGGCTATTCACGGAACCTGAGAAGCGGATGAATAATCATCTGCTTCCGGAAGAAATCGAAGAATTTCTTGGGAATCCTACAAGATCAATTCGTTCTTTTTTCTCTGACAGATGGTCAGAACTTCATCTGGGTTCGAATCCTACTGAGAGGTCCACTAGAGATCATAAATTGTTGAAGAAAAAACAAGATGTTTCTTTTGTCCCTTCCAGGCGAGCGGAAAATAAAGAAATGGTTGATATATTCAAGATAATTACGTATTTACAAGATACCGTCTCAATTCATCCTTCGGAACCAGATCACATCCCGGATCTGGTTCCGAAGGATGAACCGGATATGGACAGTTCCAATAAGATTTCATTCTTGAACAAAAATCCATTTTTTGATTTCTTTCATCTATTCCATGACCGGAACAAAGGGGGATACGCGTTACGCCACGATTTTTTTGAATCAGAAGAGAGATTCCCAGAAATGGCGGATCTATTCACTCTATCAATAACCGAGCCGGATCTGGTGTTTCATAGGGGATTTGCCTTTTCTATTGATTCCTACGGGTTGGATCAAAAAAAATTCTTGAATGAGGTATTCAACTCCAGAGATGAATCGAAAAAGAAATCTTTATTGGTTCTACCTCCTCTTTTTTATGAGGAGAATGAATCTTTTTCTCGAAGGATCAGAAAAAAATCGGTCCGGATCTACTGCGGGAATGAGTTGGAAGATCCCAAACTAAAAACAGCGGTATTTGCTAGCAACAACATAATGGAGGCAGTCAATCAATATAGATTGATCCGAAATCTGATTCAAATCCAATATAGCACCTATGGAAGAAATGTATCGGATCGATTCTTTTTAATGAATAGATCCGATCGCAACTTCGAATATGGAATTCAAAGGGATCAAATAGGAAATGATACTCTGAATCATATAACTATAATGAAATATACGATCAACCAACATTTATCGAATTTGAAAAAGAGTCAGAAGAAATGGTTTGATCCTCTTATTTCTCGAACTGAGAGATCCATGAATCGGGATCCTGATGCATATAGATACAAATGGTCCAATGGGAGCAAGAATTTCCAGGAACATTTGGAACATTTCGTTTCTGAACAGAAGAATCCTTTTCAAGTAGTGCAAGTAGTGTTCGATCGATTACGTATTAATCAATATTCGATTGATTGGTCCGAGGCTATCGACAAAGAAGATTTGTCTAAGTCACTTCGTTTCTTTTTGTCCAAGTCACTTCCCTTTTTCTTTGTGAATATCGGGAATATCCCCATTCATAGGTCCGAGATCCACATCTATGAATTGAAAGGTCCGAATGATCAACTCTGCAATCAGTTGTTAGAATCCATAGGTGTTCAAATCGTTCATTTGAAGAAATTGAAACCCTTCTTATTGGATGATCATGATACTTCCCAAAGACCAAAATTCTTGATCAATGGAGGAACAATATTACCATTTTTGTTCAAAAAGATACCAAAGCGGATGATTGACTCATTCCATACTAGAAAGAATCGCAGGAAATCCTTTGATAACAAGGATTCCTATTTCTCAATGATATCCCACGATCGAGACAATTGGCTGAATCCCGTGAAACCATTTAATAGAAGTTCATTGATATCTTCTTTTTATAAAGCAAATCGACTTCGATTCTTGAATGATCCACATCACTTCTGGTTCTATTGTAACAAAAGATTCCCCTTTGATGTGGAAAAGACCCGTATCAATAATTATGATCTTACATATGGACAATTCCTCAATATCTTGTCCATTCGCAACAAAATCTTTTCTTTGTGCGTCGGTAAAAAAAAATACCTTTTTTTGGAGAGAGAGACTATTTCACCAATCGAGTCACAGGTATCTGACATCTTCATACCTAACGATTTCCCACAAAGTGGTGATGAAACGTATAACTTGTACAAATTGTACAAATCTTTCCATTTTCCAATTCGATCCGATCCATTCGTTCGTGGAGCTATTTACTCGATCGCAGACATTTCTGCAACACCTCTAACAGAGGAACAAATAGTCAATTTGGAAAAAACTTATTGTCAGCCTCTTTCAGATATGAATCTATCTGATTCAGAAGGGAATAACTTGCATCAGTATCTCAGTTTCAATTCAAACATGGGTTTGATTCACACTCCATGTTCTGAGAAGTATTTACCATCCGGAAAGAGGAAAAAACGGAGTCTTTGTCTAAATAAATGCGTTGAGAAAGGGCAGATGTATAGAACCTTTCAACGAGATAGTGCTTTTTCAAATCTCTCAAAATGGAATCTGTTCCAAACATATATGCCATGGTTCCTTACTTCGACAGGGTGCAAATATCTCAATTTCACCCTTTTAGATACTCTTTCAGACCCATTGCCGATACTGAGTAGTAGTCAAAAATTTGTATCCATTTTTCATGATATGATGCATGGATCAGATATATCACGGCCAATTCCTCAGAAGATTCTTCCACATGAGATTTCGAGTCAATGTTTACAGAATCTTCTTCTGTCCGAAGAAATGATTCATCGAAATAATGAGTCACCCGTTCCATTGATATGGGCACATCTGAGATCAACAAATGCTCGGGAGTTCCTCTATTCCATCTTTTTCCTTCTTCTTGTTGCTGGATATCTCGTTCGTATACATCTTCTCTTTGTTTCCCGAGCCTCTAGTGAGTTACAGACAGAGTTAGAAAAGATCAAATCTTTGATGATTCCATCATACATGATGGAATTTCGAAAACTTCTGGATAGGTATCCTACATCTGAACTGAATCCTTTCTGGTTAAAGAATCTCTTTCTAGTTGTTCTGGAACAATTAGGAGATTCTCTGGAAGAAATACGGGGTTCTGCTTCTGGTGGCAACATGCTATTGGGTGGTGGTCCCGCTTATGGGGTCAAATCAATACGTTCTAAGAAGAAATATTGGAAGATCAATCTCATCGATCTTGTAAGTATCATACCAAATCCCATCAATCGAATCATTTTTTCGAGAAATACGAGACATCTAAGTCGTACAAGTAAAGAGATCTATTCATTGATAAGAAAAAGAAAAAACGTGAACGGTGATTGGATTGATGAGAAAATAGAATTCTGGGTCGCGAACAGTGATTCGATTGATGATGAAGAAAGAGAATTCTTGGTTCAGTTCTCCACCTTAACGACAGAAAAAAGGATTGATCAAATTCTATTGAGTCTGACTCATAGTGATCATTTATCAAAGAATGACTCTGGTTATCAAATGATTGAACAACCGGGTTCCTTACGATACTTAGTTGACATTCATCAAAAGGATCTAATGAATTATGAGTTCAATAGATCCTGTTTAGCAGAAAGACGGATATTCCTTGCTCATTATCAGACAATCACTTATTCACAAGCCTCGTGTGGGGCTAATAGTTTTCATTCCCCATCTCCTCATGGAAAACCCTTTTCGCTCCGCTTAGCCCTATCCCCTTCTAGAGGTATTTTAGTGATAGGTTCTATAGGAACTGGACGATCCTGTTTGGTCAAATACCTAGCGACAAACTCCTATGTTCCTTTCATTACGGTATTTCCGAACAAGTTCCTGGATGACAAGCCTAAAGGTTATCTTATTGATGATATCGATATTGATGATAGTGACGATATTGATGATAGTGACGATATTGATGATGACCTTGATATTGATACGGAGCTGCTAACTATGACGAATGTGCTAACTATGTATATGACGCCGAAAATAGACCTATTTGATATCACCCTTCAATTCGAATTAGCAAAAGCAATGTCTCCTTGCATAATATGGATTCCAAACATTCATGATCTGCATGTGAATGAGTCGAATTACTTATCCCTCGGTCTATTACTCTCCAGGGATTGTGAAAGATGTTCCACTGGAAAGATTCTTGTTATTGCTTCGACTCATATTCCCCAAAAAGTGGATCCCGCTCTAATAGCTCCGAATAAATTAAATACATGCATTAAGATACGAAGGCTTCTTCTTCCACAACAACGAAAGCACTTTTTAATTCTTTCATATACTAGGGGATTTCACTTGGAAAAGAAGATGTTCCATACTAACGGATTCGGGTCCATAACCATGGGTTCCAATGCGCGAGATCTTGTAGCACTTATCAATGAGGCCCTATCAATTAGTATTACACAGAAGAAATCCATTATAGAAACTAATACAATTAGATCAGCTCTTCATAGAAAAACTTGGGATTTTCGATCCCAGATAAGATCGGTTCAGGATCATGGGATCCTTTTCTATCAGATAGGAAGGGCTGTTACACAAAATGTACTTCTAAGTAATTGCCCCATAGATCCTATATCTATCTATATGAAGAAGAGATCATGTAAGGGAGGGGATTCTTATTTGTACAAATGGTACTTCGAACTTGGAACGAGCATGAAGAAATTAACGATACTTCTTTATCTTTTGAGTTGTTCTGCCGGATCGGTCGCTCAAGATCTTTGGTCTTCATCCAGACACGATGAAAAAAATTGGATCACTTCTTATGGATTCGTTGAGAATGATTCTGATCTAGTTCATGGCCTATTACTATTATTACTATTAGAAGTAGAAGTAGAAGGCACTCTGGCTCTGGCGGGATCCTCACGGACAGAAAAATATTGCAGTCAGTTTGATAATAATCGAGTGACATTACTTCTTCGGTCCGAACCAAGGAATCAGTTAGATATGATGCAAAATGGATCTTGTTCTATCGTTGATCAGAGATTTCTATATGAAAAATACGAATCGGAGTTTGAAGAAGGGGAAGGGGCCCTCGATCCGCAACAGATAGAGGAGGATTTATTCAATCACATAGTTTGGGCTCCTAGAATATGGCGCCCTAATCTATTTGATTGTATCGAAAGGCCCACTGAATTGGGATTTCCCTATTGGACTGGGTCATTTCGGGGCAAATGGATCATTTATCATAAAGAGGATGAGCTTCAAGAGAATGATTCGGAGTTCTTGCAGAGTGGAACCATGCAGTACCAGACACGAGATAGATCTTCCAAAGAACAAGGCTTTTTTCGAACAAGCCAATTCATTTGGGACCCTGCGGATCCATTCTTTTCCCTATTCAAAGATCAGCCCTCTGTCTCTGTGTTTTCACGTCGAGAATTCTTTGCAGATGAAGAGATGTCAAAGGGGCTTATTGCTTCCCAAACAAATCCTCCTACATCTATATATAAACGCTGGTTCATCAAGAATACGCAAGAAAAGCACTTCGAATTGTTGATTCATCGCCAGAGATGGTTTAGAACCCATAGTTCATTATCTAATGGACCTTTCCGTTCTAATACTCTATCCGAGAGTTATCAGTATTTATCAAATCTGTTCCTATCTAACGGAACGCTATTGGATCAAATGACAAAGACATTGTTGAGAAAGAGATGGCTTTTCCCGGATGAAATGAAACATTTGATTCATGTAACAGGAGAAAGATTCCCCATTCCTTAGCCGTAAAGATATGTGCCCATGAAAAGGGGATTCAGTGGAACAGAATTGGCCGGATGGTAGAGTCGTGGAAACACTTGTTTATTCCATCTTTTTGGCCTTAACTCCGTGGAACAATATGCTACTGCTGAAACATGGAAGAATTGAAATCTTAGATCACTATGTGTGGATGATATGAACTGCTTAAACAAGAATTCTTGAACGGCGAAAGAGCCTATTACTCGCTACATCAAACAATTTATACTAATGAAACCATGTAAATCCATCGGAAAATACGCATGTCCGCTGAAATGGTTGTTGCTATTTGCTCCAATAACGAATCATTGGTTTCATTGAATAACTAAAGAAGATAGATAGACCTTTCTCTTCGTCTCAGGTCGATGGATCTCCTCAATTGGAAGATCTCCCATACGGATAATACACATTCCAGTTGACCGAGCCTAATTCTAATTGCTTTGTTCCGAAGCAAAGATATCCACGGAGGCGGGTTCGTCCTATTCAGATATTCACGACCAAGAGCCTCTTTCGGATAGGCCCTGAAAGGAGAAGGAAGGCTGGAATGCCAACAGACGTCTGTCTATTCTCTAATTCACCCGACCCGATAGTACCCATTTTGAGAACGTCCAGTGCCAAAGTCACTGAATGGGTAAGTCGCCAATCCCTAATGTAATGTACTTTCTTTGCTGGGTTACGGGTACTGGTGGGTATTTTACCAGAGGTTTTTCTATCAATCTACCTTGTGCGATTCCTGTTGAATCCTATACTCGGGGGGTGCGCGCAGGGCGGACGATTTCCAAACGGACTCCTATAGAGTATAGATAAGATCGCCAAGATTTCGTGATCCGCTGCCGATTCCAACAGCTCGGACTCGGATCGTGAGGATCGCCGGAATACTTCGTATCAACAGATAAGATACTCGTCAATATTGATTAGATCCGAAATCTGTTATGGAATTGCTCATTAAATAAGCATTCTCAATATTATGCCTTGAAGAGGACTCGAACCTCCACGCTCTTTAGCACGAGATTTTGAGTCTCGCGTGTCTACCATTTCACCATCAAGGCATCTTGAAAGTGAATCGTATTCCATGAATATGATATCTATCTAGTGTGATATATGGAATATATGACAAAGGTGGAGTGTTGGAGTATTTCTATCGATCGGTCATGTCATATAGGCCCGAGTCAAACATCAAATTGCTTCGATTGGAATTATCCGGAGGATACCTTCTATATATTAAACTATATATCAAAAAGATGTAAAATCAAACCTCTTTCTCGATTCAAGAGAAGCCCAAAGAAGTGAATAGGGTACCCAAATAACGATAGATATGTCAAAAGCGGGTCCGATTACGCCTATTCCTAATCCTAAATAGAATGTAACGACGTAGGGATCCATATGTAAACAGAGTATCTATTTACATACGCTCGAATGACCCCTTACTCATAATAAGAATGTACATAACCCTATTCCGGCCTGGTCCGGTCTGGAATGAACTTATAATCTGATGATCGAGTCGATTCCATGATTATAAGTTCATAACTCCAGCCCATTCCCATTTTGGGCGGAAAAGTAATTCTTTTATTCCAGTTAGTAAAAGGGATCTTGAACTAAAAAATAGACCTAGAAGCTAAAAGAGGGTATCCTGAGCAATTGCAAGAATTGGGTTCATTGATATTCCTAGTATAGTAGATGCTATCACACATACAGTCATACTCAATTCGATGGAATTGTTTGATCTTAAGGGAGATCTTCTATAATTTCGCACGTGAGGGGTTATTTCTTGGTTTCGCCCAGTCATTAATAACTTGATTATTTTTAGATAATAGTAGATAGAAACAACGCTCGTAAGGAGTCCTATCGAAACCAAGAAATATAGGCCTGCCTGCCATCCACACCAGAATAGATAGAGTTTTCCGAAAAAACCTGCCAGTGGAGGAAGACCCCCTAGGGATAAGAGACATAGGGCTGAAGAGAGAGCCAAAAAAGGATCTTTCGTGTATAATCCTGCATAATCTCGAATGTTATCAGTTCCGGTACGTAGACCAAATGATACAATGCAAGCAAAAGTTCCTAGATTCATGGAGATATAGAACAACATATAAGTTATCATGCTTGCATATCCATCATTTGAGTCTCCAACAATTATTCCAATAATTACATATCCGATTTGACCGATGGACGAATATGCAAGCATACGTTTCATACTTGTTTGAGTAAGAGCAATGAGATTCCCCAATATCATGCTAAGAATAGCTAGGATTTCCAAAAGAAGATGCCATTCGGTTGATGAGAAATAAAAAAGAATATCGAAAATTCGCGTGGCTGAAGCTGAAGCAGCTACTTTCGAAGTAACAGAAAGAAAAGCAACGACTGGAGTGGGAGAGTCAGAGTCGAAAAGAGGATTCCTCACTTCTTTCTCTCATTCAAAACCGTGCATGAGACTTTCACCTCGCACGGCTCCTAAGTGATAAAAGAAAGAAGAGTAGTTCTTCTTTCTTTTTTGATTACCTTCCTCGCGTATGTATAAGACCGAATCCATTCGATTTCTAAAAAGGATTACTAATCCTTAACTTTTCGAGGAATCCTTCATCAGTGGTTGTGAATGACTGACTTTTTCAATCCTTTCGACCTTGGTTCCGTAGGAGCAAGTCAGAAAGATTGAGAAATAGAACCATCTGATTTGATTCGTTCTCCATAGCCATGAGATGATCATCTTAGGGTGATCCTTTTGTCAACGGATGCTCCTATTACACTCGTAGTCTCTGAAGGATGAGAACCAACTATGTAGCATCTACATCGATAATTCAAGTATTGTATACGTCATTAGTCCGATCCTTTGTAGAACTACCCGTAATAACGAACTTGCAAAAGGGATCTGTTTATCATAAAGAGATTCGTTGTTCCTGACCCTGCTTCACCTTAATTGTTATTTGAACAAAAGGATCACAATAAACTTTTGGTCAAAGTGATGTCTTGGTCCGAGTGGGGATAGCATTTCTCTTCTGCATGTCTATGGAGTTTTGAAAAATCCAAACATCTCAGAGATAGATATAGAGGTAGGAATTTGTCGAACGAACCGCACTCCTTCGTATACATCAGGAGTCCATTGATGAAAAGGGGCTGGGGAAAGCTTGAACCCAATTCCTACAGTGATGGATATAAGCGCAATTGAGATTCCTGGGGAGTTATACATTTGTGTATTGATAAGACCATTCACTATTTCTTGAAGCTCGATCTCCCCTCCAGATAAACCATATAGCCAAGATAAACCATGAACCAGAATAGAAGAGCTTGCCCCACCCATGAGTAAATATTTCGTAGTAGCCTCATTAGACCGTACATCTCTCTTGGTATATCCAGATAATAGGTAGGAGCATAAACTGAAACATTCTGGAGCTACAAAGATAGTTATTAAATCGTTAGCACCACATAAAAACATTCCTCCTAGAGTAGCTGTTAATACGAATAACAGAAACTCTGTTATAGCCATTTCTGTACATTCAATGTACTCTACGGATAGAGGAATACATAGAGTTGAACATAGTAAAATAAGAAATTGAAAGATTTCGTTGAAATTGTTCGTTTGGAAATTTCCCGAAAAGCTAATTATAGGTTCTTCTCTCCATCGGAAAAATAGGGCCGTTATGCTCATTACTAAACTTGTTGAAGAGATGAAATAGAACCAAGGTCTATCTTTTTGATCAGAGGTTGAATCGATCATCAGAAGAAGAATTAGGCCAAAAATTAGGATACATTCTGGGAAAATGAAACTTCCATGGAAGAGAAGCAAATGAAACGCTTTCATAAAAATTCTCGTAGAATCGAGAATGAAGTTTTCATTCTGTACATGCCAGATCATGAATTAGTAACTGCATCCAATCTACGAAAAAGTCCCAATTGTTTCGAACTTTCTATTTTTGGAATGGGATATTTACGGAATCCCCATGAATAGGATCAAACCTTATTCCATGATATTTCCATAAGATTCCTCTTTCTTATTCTTAAGCAAGCCCCCGAGAAGGCTTAGTTGATCCATGATTTATGTTTCATCTTTCTTTTTCTTTTTGTTTGTTTCGAGAAAGATCTCGATCAATTCCGATTCTTTCTTTTTCTATTGATTCTTTTCCGATCGAGATGTATGGATCCATGGATCTATGTGTCTATATAGATCCTGTTCATGGATTAACGAAAATGCGCAAAAGCTCTATTTGCCTCTGCCATTCTATGAGTCTCTTCCTTTTTGCGTATGGCATCGCCACTGCCTTTGGCAGCATCTACTAATTCGGAACTTAATCGGAAAGACATATTTCGACCCGGACGCTTTCGGGATGCCCCTAATAACCAACGAATGGCAAGCGCTTTTCCTTGTGTAGATCCTATTTCGATAGGAACTTGATGAGTCGATCCGCCTACACGTCTTGCTTTTACTGCTATATCAGGAGTTACTCCACGTATTGCTTGACGTAAAACAGATAGTGGATTTGTTTCTGTCTTTTGTTGAATCTTTTTCACGGCTCGATAGATAATTTGATAAGCCAATGATTTTTTTCCGTGTTTCAGAATACGGTTAACCAACATGTTAACTAATCGATTACGATAAATTGGATCGGATTTTGCAGTTTTTTCTTCTGCAGTACCTCGACGTGACATGAGCGTGAAAGAGGTTCAAGAATCAGTTTTCTTTTGATAAGGGCTAAAAACAAATCACTTATTTTGGCTTTTTGACCCCATATTGTAGGGTGGATTTAAAAAGATATGAAAAATCTCCCTCCAAGCCGTACATACGACTTTCATCGAATACGGCTTTCCACAGAATTATATATGTATCTATGAGATCGAGTATGGAATTCTGTTTACTCACTTTCACTTTAAATTGAGTATCCGTTTCCCCCCTTTTCCTGCAAGGATTGGAAATCCTGTATTTTACATATCCATACGATCGAGTCCTTGGGTTTCCGAAATAGTGTAATGGAAAAAGAAGTGCTTCGAATCATTGCTATTGGACTCGGACCTGTTCTGAAAAAGTCGAGGTATTTCGAATTGTTTGTTGACACGGACAAAGGAAGGGAAAACCTCTTAAATTATTCCAATATTGGACCTTGGACATATAGTAGTTCCGAATCAAATCTCTTTAGAAAGAAGATCTTTTGTCTCATGGTAGCCTGCTCCAGTCCCCTTCCGAAACTTTCGTTATTGGGTTAGCCATACACTTCACATGTTTCTAGCGATTCACACGGCATCATCAAATGATACAAGTCTTGGATAAGAATCTACAACGCACTAGAACGCCCTTGTTGGCGATCCTTTACTCCGACAGCATCTAGGGTTCCTCGAACAATGTGATATCTCACACCGGGTAAATCCTTAACCCTTCCTCCTCTTACTAATACTACAGAATGTTCTTGTGAATTATGGCCAATACCAGGTATATAAGCAGTGATTTCAAATCCAGAGGTTAATCGTACTCTGGCAACTTTACGTAAGGCAGAGTTTGGTTTTTTGGGGGTGATAGTGGAAAAGTTGACAGATAAGTCACCCTTACTGTCACTCTACAGAACCGTACATGAGATTTTCACCTCATACGGCTCCTCGTTCAATTCTTTCGAAGTAATTGGATCCTTTTCTTCGTTCGAGAATCTCCTCCCTTCTTCTACTCCGTCCCGAAGAGTAACTCAGACCAATTCCTTCCCGATACCTCCTAAGGAAAATCCCGAATTGGATCCAAAATGGACGGGTTAGCGTGAGCTTATCCATGCGGTTATGCACTCTTCAAATAGGAATCCATTTTCTGAAAGATCCTGGCTTTCGTGCTTTGGTGAGTCGTCCGAGATCCTTTCGATGACCTATGTTGTGTTGAAATCTATATGATCCGATCGATTGCGTAAGGGCCGCGGTAGCAACGGAACCGGGGAAAGTATACAGAAAAGACAGTTCTTTATCTATTATATCTTTTCTATTATGTATTATTCTATATTCTATTATTAGAATTATATATTATTATTATATATATTATTCTATATTCTATATTCTATTCTAAATTCTATTAGAATTATCTAATTCTTAGAATTATTTCTAATTAGATTAGTTATATTAGTCTTATAGTCTTAGTTTTAGTATTAGTTAGTGATCCCGGCTCGGCGAGTCCTTTCTTCCGTGATGAACTGTTGGCACCAGTCCTACATTCTTTGCCTGCTAGGCAAGAGGATACAAATTCTGTCTCTGTCTCGGTAGGACATGTATTTCTATTACTATTCAATTCATAAATGAAAATGAAGTAGTTAATGGTGGGGTTACCGTTATCCTTTTTTATTGTATGTGTTCCTAAGAAAAGGGCGTCACATAGGAACTCTGGAATGGAAATGGAAAAGAAAAGTAGCTCCAGTTCCTTCGGAAATGGTAGTAAGATCCTTGGCGCAAGAAGAAGGGGTGTCATCTTGACTTGGTTCTGCTTCCACTCTTTTTTTTTACAATACCGAGTCGGTTCTTCTCCTACCAGTCTCGAATAGAGCATGCTGAAAAAAATCTTCTTCATGTAAAAACTGTTCGATTTAGATCGGGAAAACGGATTTTAGGAAACCATGTGCTATGGCTCGAATCCGTAGTCAATCCTATTTCCGATAGGAGCAGTTGACAATGGAATCCAAATTTTCCCATTATTTGACTATCCATAACATAATAGTGCGAAAAGAAGGCCCGGCTCCAGGTTGTTCAGGAATAGTGGCGTTTAGTTTCTCGACCCTTTGCCTTAGGATTAGTTAGTTCTATTTCTCGATGGGACCGGGAAGGGATATAACTCAGCGGTAGAGTGTCACCTTGACGTGGTGGAAGTCATCAGTTCGAGCCTGATTATCCCTAAACCCAATGGGAGTTTTTCTATTTGGACTTGCTCCCCCGCCGTGATCGAACGAGAATGGATAAGAGGCTTGTGGGATTGACGTGATAGGGTAGGGATGGCTATATTGCTGGGAGCGAACTCCAGGCTAATATGAAGCGCATGGATGGATACAAGCCTTCGGCCTTGGAATGAAAGACAATTCCGAATCAGCTTTGTCTACGAACAAGGAAGCTATAAGTAATGCAACTATGAATCTCATGGAGAGTTCGATCCTGGCTCAGGATGAACGCTGGCGGCATGCTTAACACATGCAAGTCGGACGGGAAGTGGTGTTTCCAGTGGCGGACGGGTGAGTAACGCGTAAGAACCTGCCCTTGGGAGGGGAACAACAACTGGAAACGGTTGCTAATACCCCGTAGGCTGAGGAGCAAAAGGAGGAATCCGCCCGAGGAGGGGCTCGCGTCTGATTAGCTAGTTGGTGAGGCAATAGCTTACCAAGGCGATGATCAGTAGCTGGTCCGAGAAGATGATCAGCCACACTGGGACTGAGACACGGCCCAGACTCCTACGGGAGGCAGCAGTGGGGAATTTTCCGCAATGGGCGAAAGCCTGACGGAGCAATGCCGCGTGGAGGTAGAAGGCCCACGGGTCGTGAACTTCTTTTCTCGGAGAAGAAGCAATGACGGTATCTGAGGAATAAGCATCGGCTAACTCTGTGCCAGCAGCCGCGGTAAGACAGAGGATGCAAGCGTTATCCGGAATGATTGGGCGTAAAGCGTCTGTAGGTGGCTTTTCAAGTCCGCCGTCAAATCCCAGGGCTCAACCCTGGACAGGCGGTGGAAACTAACAAGCTGGAGTACGGTAGGGGCAGAGGGAATTTCCGGTGGAGCGGTGAAATGCGTAGAGATCGGAAAGAACACCAACGGCGAAAGCACTCTGCTGGGCCGACACTGACACTGAGAGACGAAAGCTAGGGGAGCAAATGGGATTAGATACCCCAGTAGTCCTAGCCGTAAACGATGGATACTAGGCGCTGTGCGTATCGACCCGTGCAGTGCTGTAGCTAACGCGTTAAGTATCCCGCCTGGGGAGTACGTTCGCAAGAATGAAACTCAAAGGAATTGACGGGGGCCCGCACAAGCGGTGGAGCATGTGGTTTAATTCGATGCAAAGCGAAGAACCTTACCAGGGCTTGACATGCCGTGAATCCTCTTGAAAGAGAGGGGTGCCTTCGGGAACGCGGACACAGGTGGTGCATGGCTGTCGTCAGCTCGTGCCGTAAGGTGTTGGGTTAAGTCCCGCAACGAGCGCAACCCTCGTGTTTAGTTGCCACCATTGAGTTTGGAACCCTGAACAGACTGCCGGTGATAAGCCGGAGGAAGGTGAGGATGACGTCAAGTCATCATGCCCCTTATGCCCTGGGCGACACACGTGCTACAATGGCCGGGACAAAGGGTCGCGATCCCGCGAGGGTGAGCTAACTCCAAAACCCGTCCTCAGTTCGGATTGCAGGCTGCAACTCGCCTGCATGAAGCCGGAATCGCTAGTAATCGCCGGTCAGCCATACGGCGGTGAATTCGTTCCCGGGCCTTGTACACACCGCCCGTCACACTATGGGAGCTGGCCATGCCCGAAGTCGTTACCTTAACCGCAAGGAGGGGGATGCCGAAGGCAGGGCTAGTGACTGGAGTGAAGTCGTAACAAGGTAGCCGTACTGGAAGGTGCGGCTGGATCACCTCCTTTTCAGGGAGAGCTAATGCTTATGCTTGTTGGGTATTTTGGTTTGACACTGCTTGACACCCAAAAAGGAGCGAGCTACATCTGAGCTAAGCTTGGATATGGAAGTCTTCTTTCGTTTCTCGACGGTGAAGTAAGACCAAGCCCATGAGCTTATTATCCTAGGTCGTAACAAGTTGATAGGATCTCTTTTGTACGTCCCCATGTCCCTCCCGTGTGGCGACATGGGGACGTACAAAAGGAAAGAGAGGGATGGGGTTTCTCTCGCTTTTGGCATAGCAGGCCTCCCCCAGGGAGGCCCACACGACGGGCTATTAGCTCAGTGGTAGAGCGCGCCCCTGATAATTGCGTCGTTGTGCCTGGGCTGTGAGGGCTCTCAGCTACATGGATAGTTCAATGTGCTCATCGGCGCCTGACCCGGAGATGTGGATCATCCAAGGCACATTAGCATGGCGTACTCCTCCTGTTCGAATCGGAGTTTGAAACCAAACTTCTCCTCAGGAAGATAGATGGGGCGATTCAGGTGAGATCCAATGGAGATCGAACTTTCTATTCACTCGTGGGATCCGGGCGGTCCGGGGGGGGGCCCCCACGGCTCCTCTCTTCTCGAGAATCCATACACCCCTTATCAGTGTATGGACAGCTATCTCTCGAGCACAGGTTGAGGTTCGGCCTCAATGGGAAAATGGAGCACCTAACAACGCATCTTCACAGACCAAGAACTACGAGATCGCCCCTTTCATTCTGGGGTGACGGAGGGATCGTACCATTCGAGCCTTTTTTTCATGCCGGAGGTCTGAAGAAAGCAGCAATCAATAGGATTTCCCGAATCCTCCCTTCCCGAAAGGAAGAACGTGAAATTCTTTTTCCTTTCCGCAGGGACCAGGAGATTGGATCTAGCCATAACATAAGAAGAATGCTTGGTATAAATAACTCACTTCTTGGTTTTCGACCCCCTCAGTCACTACGAGCGCTCCCCGATCAGTGCAATGGGATGTATCTATTTATCTATCTCTTGACTCGAAATGGGAGGAGAGCAGGTTTGAAAAAGGATCTTAGAGTGTCTAGGGTTGGGCCAGGAGGGTCTCTTAACGCTTTCTTTTTTCTTCCCATCGGAGTGATTTCACAAAGACTTGCCATGGTAAGGGAGAAAGGGGAACAAGCACACTTGAAGAGCGCAGTACAATGGAGAGTTGTATGCTGCGTTCGGGAAGGATGAATCGCTCCCGAAAAAGAATCTATTGATTCTCTCCCAATTGGTTGGATCGTAGGTGCGATGATTTACTTCACGGGCGAGGTCTCTGGTTCAAGTCCAGGATGGCCCAGCTGCGCCAGGGAAAAGAATAGAAGAAGCATCTGACTCTTTCATGCATGCTTCACTCGGCTCGGGGGGATATAGCTCAGTTGGTAGAGCTCCGCTCTTGCAATTGGGTCGTTGCGATTACGGGTTGGATGTCTAATTGTCCAGGCGGTAATGATAGTATCTTGTACCTGAACCGGTGGCTCACTTTTTCTAAGGAATGGGGAAGAGGACCGAAACATGCCACTGAAAGACTCTACTGAGACAAAAAGATGGGCTGTCAAGAACGTAGAGGAGGTAGGATGGGCAGTTGGTCAGATCTAGTATGGATCGTACATGGACGATAGTTGGAGTCGGCGGCTCTCCTAGGGTTCCCTCATCTGGGATCCCTAGGAAAGAGGATCAAGTTGGCCCTTGCGAATAGCTTGATGCACTATCTCCCTTCAACCCTTTGAGCGAAATGTGGCAAAAGGAAGGAAAATCCATGGACCGACCCCATCGTCTCCACCCCGTAGAAACTGCGAGATCGCCCCAAGGACGCCTTCGGCATCCAGGGGTCACGGACCGACCATAGACCCTGTTCAATAAGTGGAAGGCATTAGCTGTCCGCTCTCCGGTTGGGCAGTAAGGGTCGGAGAAGGGCAATCACTCGTTCTTAAAACCAGCATTCTGAAGACCAAAGAGTCGGGCGGAAAAGGGGGGAGAGCTCTTTGTTCCTGGTTCTCCTGTAGCTGGAACCACAAGGATCCTTAGAATGGTATTCCAACTCAGCACCCTTTGAGATTTTGAGAAGAGTTGCTCTTTGGAGAGCACAGTACGATGAAAGTTGTAAGCTGTGTTCGGGGGGGAGTTATTGTCTATTGTTGGCCTCTATGGTAGAATCAGCCGGAGAGGCCTGAGAGGCGGTGGTTTACCCTGTGGCGGATGTCAGCGGTTCGAGTCCGCTTATCTCCAGCCCGTGAACTTAGCTGATACTATGATAGCATCCAATTTTTCCAATTCGGCAGTTCGATCTATGATTTCTCATTCATGGACGTTGATAAGATCCTTCCATTTAGCAGCACCTTAGGATGGCATAGCCTTCACGTGAATGGCGAGGTTCAAACGAGGAAAGGCTTACGGTGGATACCTAGGCACCCAGAGACGAGGAAGGGCGTAGCAAGCGACGAAATGCTTCGGGGAGTTGAAAATAAGCATAGATCCGGAGATTCCCGAATAGGTCAACCTTTCGAACTGCTGCTGAATCCATGGGCAGGCAAGAGACAACCTGGCGAACTGAAACATCTTAGTAGCCAGAGGAAAATAAAGCAAAAGCGATTCCCGTAGTAGCGGCGAGCGAAATGGGAGCAGCCTAAACCGTGAAAACGGGGTTGTGGGAGAGCAATAAAAGCGTCATGCTGCTAGGCGAAGCGGTGGAGTGCTGCACCCTAGATGGCGAGAGTCCAGTAGCCGAAAGCATCACTAGCTTACGCTCTGACCCGAGTAGCATGGGGCACGTGGAATCCCGTGTGAATCAGCAAGGACCACCTTGCAAGGCTAAATACTCCTGGGTGACCGATAGCGAAGTAGTACCGTGAGGGAAAGGTGAAAAGAACCCCCATCGGGGAGTGAAATAGAACATGAAACCGTGAGCTCCCAAGCAGTGGGAGGAGAATGTGATCTCTGACCGCGTGCCTGTTGAAGAATGAGCCGGCGACTCATAGGCAGTGGCTTGGTTAAGGGAATCCACCGTAGCCGTAGCGAAAGCGAGTCTTCATAGGGCGATTGTCACTGCTTATGGACCCGAACCTGGGTGATCTATCCATGACCAGGATGAAGCTTGGGTGAAACTAAGTGGAGGTCCGAACCGACTGATGTTGAAGAATCAGCGGATGAGTTGTGGTTAGGGGTGAAATGCCACTCGAACCCAGAGCTAGCTGGTTCTCCCCGAAATGCGTTGAGGCGCAGCAGTTGACTGGACATCTAGGGGTAAAGCACTGTTTCGGTGCGGGCCGCGAGAGCGGTACCAAATCGAGGCAAACTCTGAATACTAGATATGACCCCAAACAGGGGTCAAGGTCGGCCAGTGAGACGATGGGGGATAAGCTTCATCGTCGAGAGGGAAACAGCCCGGATCACCAGCTAAGGCCCCTAAATGACCGCTCAGTGATAAAGGAGGTAGGGGTGCAGAGACAGCCAGGAGGTTTGCCTAGAAGCAGCCACCCTTGAAAGAGTGCGTAATAGCTCACTGATCGAGCGCTCTTGCGCCGAAGATGAACGGGGCTAAGCGATCTGCCGAAGCTGTGGGATGTAAAAATGCATCGGTAGGGGAGCGTTCCGCCTTAGAGGGAAGCACCCGCGCAAGCAGGTGTGGACGAAGCGGAAGCGAGAATGTCGGCTTGAGTAACGCAAACATTGGTGAGAATCCAATGCCCCGAAAACCTAAGGGTTCCTCCGCAAGGTTCGTCCACGGAGGGTGAGTCAGGGCCTAAGATCAGGCCGAAAAGCGTAGTCGATGGACAACAGGTGAATATTCCTGTACTACCCCTTGTTGGTCCCGAGGGACGGAGGAGGCTAGGTTAGCCGAAAGATGGTTATCGGTTCAAGGACGCAAGGTGACCTTGCTTTTTCAGGGTAAGAAGGGGTAGAGGAAATGCCTCGAGCCAATGTCCGAGTACCAGGCGCTACGGCGCTGAAGTAACCCATGCCATACTCCCAGGAAAAGCTCGAACGACCTTCAACAAGAGGGTACCTGTACCCGAAACCGACACAGGTGGGTAGGTAGAGAATACCTAGGGGCGCGAGACAACTCTCTCTAAGGAACTCGGCAAAATAGCCCCGTAACTTCGGGAGAAGGGGTGCCTCCTCAAAAAGGGGGTCGCAGTGACCAGGCCCGGGCGACTGTTTACCAAAAACACAGGTCTCCGCAAAGTCGTAAGACCATGTATGGGGGCTGACGCCTGCCCAGTGCCGGAAGGTCAAGGAAGTTGGTGACCTGATGACAGGGGAGCCGGCGACCGAAGCCCCGGTGAACGGCGGCCGTAACTATAACGGTCCTAAGGTAGCGAAATTCCTTGTCGGGTAAGTTCCGACCCGCACGAAAGGCGTAACGATCTGGGCACTGTCTCGGAGAGAGGCTCGGTGAAATAGACATGTCTGTGAAGATGCGGACTACCTGCACCTGGACAGAAAGACCCTATGAAGCTTTACTGTTCCCTGGGATTGGCTTTGGGCCTTTCCTGCGCAGCTTAGGTGGAGGGCGAAGAAGGCCTCCTTGCGGGGGGGCCCGAGCCATCAGTGAGATACCACTCTGGAAGAGCTAGAATTCTAACCTTGTGTCAGAACCTACGGGCCAAGGGACAGTCTCAGGTAGACAGTTTCTATGGGGCGTAGGCCTCCCAAAAGGTAACGGAGGCGCGCAAAGGTTTCCTCGGGCCGGACGGAGATTGGCCCTCGAGTGCAAGGGCATAAGGGAGCTTGACTGCAAGACCCACCCGTCGAGCAGGGACGAAAGTCGGCCTTAGTGATCCGACGGTGCCGAGTGGAAGGGCCGTCGCTCAACGGATAAAAGTTACTCTAGGGATAACAGGCTGATCTTCCCCAAGAGTTCACATCGACGGGAAGGTTTGGCACCTCGATGTCGGCTCTTCGCCACCTGGGGCTGTAGTATGTTCCAAGGGTTGGGCTGTTCGCCCATTAAAGCGGTACGTGAGCTGGGTTCAGAACGTCGTGAGACAGTTCGGTCCATATCCGGTGCGGGCGTTAGAGCATTGAGAGGACCTTTCCCTAGTACGAGAGGACCGGGAAGGACGCACCTCTGGTGTACCAGTTATCGTGCCTACGGTAAACGCTGGGTAGCCAAGTGCGGAGCGGATAACTGCTGAAAGCATCTAAGTAGTAAGCCCACCCCAAGATGAGTGCTCTCCTATTCCGACTTCCCCATAGCCTCCGGTAGCACAGCCGAGACAGCGGCGGGTTCTCTGCCCCTGCGGGGATGGAGCGACAGAAGTATTGAGAATCCAAGATAAGGTCACGGCGAGACGAGCCGTTTATCATTACGATAGGTGTCAAGTGGAAGTGCAGTGATGTATGCAGCTGAGGCATCCTAACAGACCGAGAGATTTGAACCTTGTTCCTACACGACCTGATCAATTAGATCAGGCACTCGCCATCTATTTTCATTGTTCAACTGTTTGACAACATGAAACAAAAACCCAAAAGCTCTGCCCTCCCTCTCTATCGGATGGAAGGGCAGAGGCCTTTGGCGTCCCTTCCAGTCAAGAATCGGGGCCTCACAATCACTAGACAATATTTATCTCATGCCTTTCTTCGTTCATGGTTCGATATTCTGGTGTCCTAGGCGTAGAGGAACCACACCAATCCATCCCGAACTTGGTGGTTAAACTCTACTGCGGTGACGATACTGTAGGGGGGGTCCTGCGGAAAAATAGCTCGACGCCAGAATGATAAAAAGCTTAACACCTCTTATTTTACTTTTTCATATTTCAAAAGATCAAAATAAAAAATGCAAAGGTCGTCTTATTCAATAACATCCCTTCTCTCCCACTTCACGCCTCGGAACGCACTGTTATTCTAGAGAGAAAGGCGCTTTCACATCTTCTTAACCCAAAATGGCTGAGGAGAGGAAAGGCTCCTTTTTGAAGGTACTCCCGGGAACAGATCCAGTGTAGACGGGGTGGGGCCTGTAGCTCAGAGGATTAGAGCACGTGGCTACGAACCACGGTGTCGGGGGTTCGAATCCCTCCTCGCCCACAACCGGCCCAAAAGGGAAGGGCCTTTCCCTCTGGGGGTAGTAAAATCATGATCGGGATAGCGGCCACAAAGCTATTGAACTTGGGTATGGTCCTTATAAGTCAGGTTTATTTTATTACATATAGTAACCCGGGCTGGAATCAGCATATTTGTGTTTGACTCCCCGTAACTCTTCCTCAGCCAGGCTTGGGCAGAATAGCAGAGCAAGTACAAGTATTAGTAGCATAGCAAAAATGCGTTCCTCGTCATTAATATGTTTGCTCGCGGTAATTGTGGCCTATCGGGAGAATTGATGACTGCATCTTTGATGCACTGTTGAGAATTCTGAATTGGCTATTTACAAGGGTTGGATCTATGCCGAGGTATTGAGGTTAATTCTCAAATATTGTAGAACAGAATGTGATACGATGAGATAGAATGCAATAGAAACAAAGACAGGGAACGAGTTACCTACTCCTAACGGTCAAAGCGAGCCCTTTCATTCAATTCTTCATTCTTCATGAAAGAATGAATCAAATCTCCCCAAGTAGGATTCGAACCTACGACCAGTCAGTTAACAGCCAACCGCTCTACCACTGAGCTACTGAGGAACAACGGGAGATTCGACCTCATAGAGTTCAACTCCCGTTCTCAACCCATGAACAATATGAGTCCGAAGCTTCCTTCGTAACTCCAGGAACTTCTTCGTAGTGGCTCCGTTCCATGCCTCATTTCATAGGGAACCTCAATGTGGCTCTATTTCATTATATTCCATCTATATCCCAATTCCATTCATTTCATATCCCTTTTGTGTCATTGACATAAGAGATGTCATTTATAGTATATCTGTTTCTATCTATATAGATATGGAAAGTTAAGGAATCATCATATAATAATCGAGAAATTGCAATAGAAAAGAAAAAGGGGAGGTTTGTGATGATTTTGAAATCTTTTCTACTAGGTAATCCATTATCCTTATGCATGAAGATAATAAATTCGGTCGTTGTGGTCGGGCTCTATTATGGATTTCTGACCACATTCTCCATAGGGCCCTCTTATCTCTTCCTTCTCCGAGCTCGGGTTATGGAAGAAGGAACCGAGAAGGAGGTATCAGCAACAACTGGTTTTATTGCGGGACAGCTCATGATGTTCATATCGATCTATTATGCGCCTCTGCATCTAGCATTGGGTAGACCTCATACAATAACTGTCCTAGTTCTACCGTATCTTTTGTTTCATTTCTTCTGGAACAATCATAAAAACTTTTTTGATTATGGATCTACTACCAGAAATTCAATGCGTAATCTCAGCATTCAATGTGTATTCCTGAATAATCTAATTCTTCAATTATTCAACCATTTCATTTTACCAAGTTCCACGTTAGCCAGATTAGTCAACATTTATATGTTTCGATGCAACAACAAGATTTTATTTTTAACAAGTAGTTTTGTTGGTTGGTTAATTGGTCACATTTTATTCATGAAATGGGTTGGATTGGTATTATTCTGGATACGGCAAAATCATTCTATTCGATCTAATAAGTATCTTGTGTCAGAATTGAGAAATTCTATGGCTCGAATCTTTAGTATTCTCTTATTTATCACCTGTGTTTACTATTTAGGCAGAATGCCGTCGCCTATTGTCACTAAGAAACTGAAAGAGAAAGAAACCTCAGAAACGGAAGAAAGCGATGTAGAAACAACTTACGAAATGAAGGAGACTAAACAGGAACAAGAGGGATCCACCGAAGAAAACCTTTGTTCGGAAGAAAAGGAGGATCTGGACAAAATAGATGAAACGGAAGAGATCCGAGTGAATGGAAAGGAAAAAACAAAGGATGAATTTCACTTGAAAGAGGCACGCTATCAAGATAGCCCAGTTTACGAAGATTCTGATCTGGAGACCCATCAAGAAAATTGGGAATTGGGAAGACTGAAAGAAGAGAAAAAGAAAATAATGAATAAAAAGATTGACACATAATAAAAATATAAGAATAAATAAGATGAGATTCGTCCGCCTCCCATATATTTTATTCCTTCGCCCATAAAGAAACTTGCAACACCAATTCCATTTAGAATTCCATCAATTATATATTTATCAAAAAACTGAGTTAGCTCGGCTAACCCTCTTATACTCATGGTGAAAATACCAGTATAAAAAATATCTATATAACCACGATTATATGACCAATTGTATATCATACCTTTTATTTTGTCCAGAATAATTCTTTTAGGACCTCTTTTGAAAAAGAAATTAATTAAGCCCAAATTTTTGAAAGATGAATAAATAGATCCATAAAAAAGAGATGCTATAAATAGTCCGAAAAGAGCTATACTTACTGAAAAAAAAGCACTTGAAAAAAATCCATACCAATCCTCAGAAGAATTCAAATTATGATGAAAAAGGTTTGTCGATGGAGTTAACCATTTCGATAATAGATCCAAATCTATTACTCCTCCGTTAAAAGAAATTCCTATTGATCCAATGAACAAAGTTAATAGTACTAATATAAGGAGAGGAAATAACATAGTATTGCTCGATTCGTGAGGATACATATAAGTGTATTTATTTCTAAAGTAAGTACTAAAGTCTCGTATCTTATTTCTTACATTCTCGTCAATTTTAAATATATTTTTTGAAAAAAAACAAACCTTATTCTTATTCATTTTTGAAAAAAAGAAATTACTATTGACTTTCTTAAGTGTCCCTTTTCCCCATAGAGATATTGAATAAAACGAGCTCTTTTTTGTACTACTAAGACTACTATAATCTTGAAAATGAATGCGTAAATACCCATCAAAGGTAAGTAAATACATCCTAAACATATAAAATGCAGTTAATCCTGTTGTGAACCAAGCTATTAGTGCGAAAATTGGTGAGTATAACCAACTATCGTGAAGAATTTCATCTTTAGACCAAAAACAAGCAAGAGGCGGAATACCACAAAGAGAAAGTGTGCCTAAAAAAAAAGTAATTTTTGTAATTGGAACATATTTTGTTAAACCTCCCATAAGAATCATATTCTGACTTTTCTCTGGTGAATATCCAACAATAGGTTCCATTGAATGAATAATGGATCCGGATCCCAAAAACAATAAAGCTTTAGAATAGGCATGGGTGATCAAATGAAATAAAGCAGCTCGATAAGAACCTATGCCTAGAGCTAACATAATATAACCCAATTGAGACATTGTAGAATAAGCTAAACTTCTTTTAATGTCTCTTTGGGCAAGAGCTAAAGTAGCTCCTAAAAATACTGTTATTATACCTACTAAACAAATGAGATTCATTATGTAAGGTATAACTATGAAAAGAGGAAGAAGCCGAGCTACAAGAAAAATCCCTGCTGCTACCATAGTAGCAGCGTGTATAAGAGCCGAAATAGGAGTGGGGCCTTCCATGGCATCAGGTAACCATACGTGAAGGGGGAATTGTGCGGATTTAGCAACTGCACCGACAAATAATAAAAAGGCGCATAAAGTAACAAATAAAGAATTGACCCCATTATTATGGATCAAGGTATTCACTATTTGGAACAAATCCCGAAATTCGAAACTACCAGTTATCCAATAAAATCCTAAGGTCCCTAATAATAAACCAAAATCTCCTATACGATTAGTTACAAAAGCTTTTTGACAAGCACTTGCTGCAACGGGTCGTGTGAACCAAAAACCTATCAATAAATACGAACACATTCCCACTAGTTCCCAAAAAATATAAATTTGTATCAAATTGGAACTAGTAACTAATCCCAACATTGAAGCATTGGAAAAACTCATATGAGCAAAAAATCTCAAATATCCTTGGTCATGAGACATATAATTGTCACTATAAATAAAAACCAGGATTCCAACAGTAGTAATTAGTATTGACATAATAGAAGTAAGTGGATCAATCAAGTGTCCGAACTCTAATAAAAAATCATTATTGATGGTCCAAGACCATAGATATTGATAGGTCAAACTTCCATTTATTTGCTGAATAGACAGATTAGCCGAAAACCACATAGCTATACTTAGTAGTAAAACACTTAGGAAAGCCCACATACGACGAAGATCTTTTGTTGCTGTCGGAATAAGTAGAAGTCCAAATCCTATTGACATAGTAACTGGGAGTGGAAAAAGGGGTATTATCCATGCATATTTATATGTATATTCCATAAGAAACCAAATTGTTCTTTTTTCTTATAATTGTTTCCAATTCACCAATTCTGATCTATTTCGAAAAGAACAAAACAATAAGAAAAAAATATGAAAAAGATCAAATACAAAAATACAAATATTGGAATTATGACTTTTTGTTTTATTAAATATATGACATCATATGAAATTTTGAATAATTGGATTTTCCCTCTTTACATTATATTCTAATTATGGAAAATGTAAAATTATGTAATTTATAGATATATTATATATTTTTAGATTTAAGATAGATTTATTATATTTATATTAAAGTTCAGTTACAGATTTCTTTATCTCTTTCTATTTTTATATTTTTCTTTCTTTTTTTTATTGTATAATCTTTATATAGAATCTTTTCTTTTATATACTATATAGATAAATTAGATAAATATTTTCTCTTACTATTCTTAATATTAAATATGAATATTTGCAATAATTGTATATATATGACTCTAATATTTTAATATATATTAAATAATATGAAATAGTAAAATTAGATTACTTTTTTTGTATATATTCTTTTATAAAACAATAAATATAAAATACGTATGTAATTATTACATTATGCAAATATCAGAATGAAGATAGAAAATTGAAATCTATTTGTCTATGAAAATAGAATCATTTTAGAATATTTTTCTTTTCTTATTTCTTTTCTTTTATTCTTTTTTTTCTATTCGTATGTTATGATATTATTGAGGAAATTTTGAAATTTATGGAATTAAGTATAGATAATGACTAATAAAAAGTAATTTATTTTAAACAATATATGTCTTTCACATACAACGATAAAAAGGAGTCACCTACTTTTTGAATGGCAGTTCCAAAAAAACGTACTTCTATGTCAAAAAAGCATATTCGTAGAAATCTTTGGAAAAAAAAAGGATCTTTAGAGGCAGTAAAAGCTTTTTCTTTAGCTAAATCTATTTCCACTGGACAGTCAAAAAGTTTTTTTGTGCGACAAAAAAAAGTCTTGGAAAAATATTAATTGACATGTTTCAAAGAACTTCCAAATTTCCATTTTTGAATTGGAAAACAAACGATTCAATTTTACTAATGTATTGTATTTGTATTTCACTTCTCCTTATTAGTTAGAGCTAGGTAATATAAAAAATAAGAATCTTTCTTTCTACTTGATTCAAAATACTCAGTATTGTGTATTTTATTTTTTTTCTATTTTTTATAGTCTTTCTATATTTCTATTATATTCTATTTATCTAAATTTATATTGATTGATATTAAATTCGTGAGATGATTTTTTCTTTCCTATGAATTGTGCTTTTCAAAATAGAAAAGCACAATTACGATAGACAAAGAAAAATCTGAATATTTCATTATACTTTATACTAAAGTGTTGGGTCTAAAAATCGTTATTAGAAAAAAATTATGAATTTTATACCCCGACTGAGAACGAAGCTTTTGAGTTCTGACTGTTCTGGATAAACAAGAGCTAGGTTTCTAGTACGGAAAAGTTGATTATTAAAACTGAACTTACGAAGATGAAGATACTAATTAAGTATTATTGATATTGAATATTTCCATATGAATAGAAATGCATCTTTATTCATTTTTTCCTAAATTATATTGGATATATACAATATACAATTCAACATGAATTTCCTATTATTATTTAAGGTAAGCCGCCATGGTGAAATTGGTAGACACGCTGCTCTTAGGAAGCAGTGCTAGAGCATCTCGGTTCGAGTCCGAGTGGCGGCATAAATAATTATTAATATTAATAATATAGACACAATAGATCTAATAGAATCTAAGATATTTAAAATATTATATTTTAGATTTTATTTAATCCTCTCCCCAATTTTAATTATTTAAAAGGGACTCTTCTTTATGATATTTGCAACCTTAGAACATATATTAACTCATATTTCCTTTTCGATCATCTCAATTGTGATTATAATTCATTTGATGAACTTATTAGTTGACGAAATTGAAGGATTACGTAATTCGTCAGAAAAAGGGATGATAGCTACTTTTTTCTCTATAACAGGGTTTTTAGTTATTCGTTGGATTTCTTCGGAACATTTTCCCTTAAGTAATTTATACGAATCATTAATCTTCCTTTCATGGAGTTTATCCATTATTCATATGATTCCGTATCTTGGGAATCATAAAAATGATTTAAGCGCAATAACTGCGCCAAGTGCCATTTTTACCCAAGGTTTCGCCACGTCAGGTCTTTCAAATGAAATGCATCAACCCGCGATATTAGTACCTGCTCTACAATCTCAGTGGTTAATGATGCATGTCAGTATGATGCTATTGAGCTATGCATCTCTTTTATGCGGATCGTTATTATCAATTGCTCTTATAGTGATTACATTTCAAAAAAAAATCGATTTTTTAAAGAATTTTTTAAGTTTAAGGAAGTCGTTTTTCTTTGGTAATATGGAATATTTGAACGAAAAAGGAAGTGTATTAAAAAATACTTTTTTCCTCTCAGTTCAAAATTTTTACAAATATCAATTAATTCAGCGTTTAGATTATTGGAGTTATCGTGTCATTAGTTTAGGGTTTACCTTTTTAACCATAGGCATTCTTTCTGGAGCAGTATGGGCTAATGAAGCATGGGGTTCTTATTGGAATTGGGACCCTAAGGAAACTTGGGCATTTATTACTTGGACCATATTTGCAATTTATTTACATATTAGAACAAATTCAAAATTGCAAGATCAAGGCACGAATTCGGCATTTGTAGCTTCTATAGGATTTCTTCTAATTTGGATATGCTATTTTGGGATCAATCTATTAGGAATCGGGTTCCATAGTTATGGTTCATTCCAATTAATATCTAATTGAATAAAATAAACTACATGAAGAATACATAAAAAAATCGTCTGATACACAATGAAATTTTGTTCGAGTTTTTGAGAACCGTTTAAATATAGGAATTATTCAAAAGGTTCTCAAAAACTTTATATGTATTTCATTACAATTCTAATTAACCTTTCCCTTTTTTTTTCATTGTACAACGAAGAATCGTGAAAATATGAAAGTCAAAGAATTCTAAGACTTTCTTTCCAATTAATGAATTTTATTTCATTTATTATTGAATCTAAAAAAAGAATTAGTATCTATAAAAATAATTAGATACTAGAACTTGTACCTTGTCAACCGATAACGGGAGAACGAAATCAGGATAAATACCAATTCCTATTACAGGTAAAAAGATACATATCGAAACGAAGAGTTCTCGTGGTCCAGAATCAAAAAAATTCGAGTTTGGAATATTAAATAGCTTGTATCCATAGAAAATCTGACGTAACATAGATAATAAAAAAATAGGAGTTATTATCATTCCAATTGCCATTACAAAAGTAATTAACATTTTTGGCATGAAAAGATATTTTGGGCTGGTAATTATTCCAAAAAAGACTAAGAATTCTGCAACAAAACCACTCATTCCTGGCAATGCAAGAGAAGCCATCGAGAAACTACTAAACATGGTAAATATTTTTGGCATTGGGATAGATATCCCCCCCATCTCTTCGAGATAAACAAAACGTATTCTATCACAACTTGTTCCTGCTAAGAAAAAAAGTGCAGCACCAATCAATCCATGAGAGATTATTTGTAAAATGGCTCCATTAAGTCCCATGCCAGTTATAGAACCAATTCCTATAATTATGAAACCCATGTGAGATACGGAAGAATAGGCAATACGTTTTTTTAAATTGCGTTGACTGAGAGAGGTTGAAGCTGCATAAATGATTTGTATTATTCCTACTATAACCAACCAGGGAGATAATCTAGAATGAGCGTGAGCTAATAATTCCATATTGATCCGAATCAATCCATATGCTCCCATCTTTAATAAGATTCCAGCTAAAAGCATACATGTACTGTAATGTGCTTCCCCGTGGGTATCTGGTAACCATGTATGTAGGGGTATCATCGGCGATTTGACAGCATAAGCAATAAGAAAACCAAAATATAGTATTATTTCCAATGCTGCAGGATACGATTGATTAGCTAATTTTTCTAAATCTAATGTTGGTTCATTGGAACCATATAAACCCATACCTAGAACTCCTATTAATAGAAAAATGGAACCTCCGGCAGTGCACAAAATAAACTTTGTAGCCGAGTACAGGCGTTTTTTTCCTCCCCACATGGATAAAAGTAAGTAAACAGGAATTAATTCTAATTCCCACATGATGAAAAAAAGTAAAAGGTCTCGAGAAGAAAATGATCCTATTTGGCCACTATACATTGCTAACATCAAGAAATAGAAAAATCGCGGATTTCGAGTAACTGGCCAAGCTGCTAAAGTAGCTAAAGTAGTGATAAATCCTGTCAGTAAAATGGGTCCTATGGAAAGTCCATCGGTTCCCAGTCTCCAGTGAAAATCAAAAAGATTGATCCATTGAAAATCCTCCTTCAATTGGGTTAATGGATCGTCCAATTGAAAATGATAACAAAATATATAGGTCATTAGAAGGAGCTCTAGTATACATATACATAGAGTATACCACCTATACGCTTTATTTCCCCTATGAGGGAAAAAGATAATTGAAGAACCCGCGAATATGGGCAAAACAAGAAGTATTGTTAACCAAGGAAAATAACTCGTGATAAAGACAAGATAAAATTAGACCAGAAAACCCCGTGCTCGGGAGAAGAATAATATATTTTCTTTTCTCGAGTACGGGCTTTTGTCGGTAAAGAGGAATCAAATGATTCAAATGGAGTTTTTTGTCACATATCAATAAGAAAGACCCATGCTGCGAGTTGTTTCATGCCATAAATAAACACGGACACTCAAAAAATCCGTTGGACAAGCGGATTCACATCTTTTACAACCTACACAATCTTCGGTTCTTGGCGCAGAAGCAATTTGCTTAGCTTTACATCCGTCCCAAGGTATCATTTCCAATACATCCGTGGGGCAAGCTCGAACACATTGGGTACATCCTATACATGTATCATAAATCTTTACTGAATGTGACATTGGGTCTATAAATTCCAGTTTTGAGCACAAAAGATTTTCGATCTGGTAAAAGAAAATAAGAAAATGAAATAAATCATATATTTTCTATTGTAGACACCAGACGAATCAATGATTTATCAAAATTTGAAGAATCAATAGATTTTCTAATCTGTTTATGAGAAAGGGCCAAGATACTTTGATTTCCATTTCAATAACCATGAAATATGAGTTTACAAATTCAATTCATGTTAAATTTACTATCTTTCTATATATATAGTCATATACATATAGAAAGATTATAGTATATAGAAATATAATTAATATATATCAGATGAATACGTTTGTTATTAGGTTAGTGATAGAATAGGTTAGTAACTCTAAATCATATAATTTATATGAAAAAAGAGAAGAAAGAAAATAAATAAGAAAATAATAATAATAAAATATTATATTCTATTTAATTCTAATTAAATTATCTTACTATTCTAATTCTATTAGATTCTATATTAGAATATTCAGAATATTCTAAAAGTCTTATGTTTTGATTTATATGTGAAAATAGAATAATTATGACTAATTATTCAGCAAATTTGATTGATTGATACGAGTTGATTTTCTGTTACGATGGATCGACGAAACAATAGCTAGTCCAATAGCTGCTTCAGCAGCCGCAATGGCTATAACAAAGATTGAGAAAATTTCTCCTTTTAATTGCCGACTATCAAATATATCGGAAAATGTGACGAGATTTATATTCACCGAATTCAGTATAAGTTCAAGACACATAAGTGCTCTAACCATGCTTCGGCTTGTGATCAGTCCATAGATACCGATAGAAAATAAATAAACACTTAGAAAAAGTACATGCTCTAACATCATTGATAAATTCCTCATCTATCTCGATTCATTTCAATATGAACGAACAAAAATTAAACCGATTCAGTTGACTAGAATAGAAGAATTACAGAACAAAAGAAGATATTCACAGTAGATTTCAATAAAATAGATTAAATCCATTTTCATTCTTTAATTAAAAAAAAAGAAGTTCTTATTATATTAGATATTAGATTATTGACGAGCCATAGTAATTGCACCTATCAAAGAAACTAAAAGAATTATAGAAATGAGTTCAAAAGGAAGATAAAAATCTGTGGATAAATGAATCCCGATTTGTTGAACGTTACTTATTAAGTCCTGTTCTATAATCTGATTTGATCTTGTAGTCCGAAAAATTCCGGACCATGATGTATCTGGGATAGTAGTCATTAATGAAAAAAAAATACTTGTACAAACCAGTGAAGTGATCCTATCTCCAACGGTCCACAAATAGGAATCATTGGAATATTCTGAACCGTTCATGAACATCACAGAAAATATGATTAATACATTTATGGCTCCCACATAAATAAGGAACTGCGCGGCAGCTACAAAATAGGAATTCAATGAAATATAGAATAGGGATATACAAAAAAGAACCAATCCCAATGAAAAGGCAGAATCGATGGGATTGGTAAGTAATACTACTCCCAGACCTCCTAATATAAGAACTGATCCCAGAAATACTACAAGAATATCATGTATTGGTCCAGGTAAATCCATTATGAAATAAAAGATAAATAAATGAGTCGAAATATTTCATGACCTTACTAAGGGTCCAGGAAAGAAACTATTTTTTTATATGATACTTTCCTAATTGAATGAAAAAAAAATGAATATCATTAGATAGATAAAATAAAGTTCTTTGGCTAATCCTACTTACTTTATTTTAAGCCAAATCTTAGTAATTGGTAATCGTTCTTGAACCAAGCAAGGGGTTCTTATTTTTTCATTTGATTTGTTGAATCCATAACTGTTTGAATTGTGTAATCTCCAATTATTGAGATTGGTAACCGACCTAAAGAAATTTGATTATAATTCAATTCGTGACGATCATAAGTGGAAAGCTCATATTCTTCAGTCATCGATAAACAGTTTGTTGGACAATACTCGACACAGTTACCGCAAAATATACAGACACCAAAATCAATACTATAATGAAGCAATTGTTTTTTCTTAATATCTTTTTCAAATTTCCAATCAACAATGGGAAGGTCTATTGGACATACGCGAACACATACTTCACAAGCAATACATTTATCAAATTCAAAGTGGATTCGACCACGAAAACGCTCTGATGTGATTGATTTTTCATAAGGATATTGAATAGTTACAGGTAAACGATTTGTATGGGATAAGGTAATTATGAAACTTTGTCCAATGTACCTTGCGGCTCGTATTGTTTGTTTGCCATAATTCATGAACCCAGTAACCATAGGTAACATATTTTAGATATCCATGAATAAAATTTATGTTTCTTTTTCTTGGTTGATATAAGTTATGAATATAGAATATTCATTATTGTTTTCTCTTAATTTCTTATTTTTATTTATAGTTTATAGTGAAACAAGTTGAAAAGAAGTTGTCAATAATAGATTACCTAGAGAAATAGGTAAAAGAAATTTCCATCCAAGATTTAATAATTGATCCATTCTCATCCTAGGTAAAGTCCATCTTGTTGTGATAGGAATGAACAGAAACAAATAAGCTTTAGCTAATGTAATAAAGATACTAATTGCTATTACAAAGACTCTAAACATTTTATTTATTCCAAAAAGTTCAGTAAGAGATATGTACGGAATAGAAAAATCCCACCCACCTAAGTAAAGAACTGTTACAAATAATGACGAAACTAATAGATTTAAGTAAGAAGCAAGATAAAAGAACCCGTATTTTATACCTGAATATTCGGTTTGATAACCTGCTACTAATTCCTCCTCTGCTTCTGGTAAATCAAAAGGTAATCTTTCACATTCTGCTAGAGAAGACATTAGAAAAACTAGAAACCCTATGGGCTGACGCCACAGATTCCATCCCCCAAAACCATATTTGGACTGTGCCTCAATTATATCAACTGTACTTGAACTGTTAGATAATTATAGTCGATGATAGCATCACTGCTCCCATCGCTATTCCAAAACCGTACATGAAACCTAAGCTTCATACGGCTCCTCTATGGCCACAAAGAAATGTAAGGTAAGGACTAGTTTAGTATTATAGCTCTCCTTGGACCTTAGGTAAATACAATGTAAAGAGAAATTGGAGGTTGGAGAGTCCTCAATTCGACCAATAACATTCTATCTGTTAGAATAAGAAAAAGCACTTCCGAATTGATCTCATCCCTTATAATGATATTATAATGAAAATAATCAAAATTTATCTTTGTTCAGCAATAACTTAATCCTTCAATCAAATACTGGTTCTATTCATAAATAGAAAGAATTGGGCATTAGTTAATGAATCATGATAAGAATTTACATATGCATATGTATAAAGAAAGAAATATTTTCTTATTATTCTCGTTTTATTCTTTTTTATTATATTATTGTATTGCATTCTATTTGTCTTGTTCCTGTTCTTCTTTCTGAAAACTAAAAAAAAAAAGAAAATAAAGGATTAATTCGTTCTTGATAGTCATTTATTGAATCAGCGAATAGTAGCATACTCTAGATCGGAATCGTGGGGAAGTACTGCTTGATCATTTCTACCAACTTCAAGCCCTTATTATGATTCGTTTTATGCAAAGTTTTATGCAAAAATCCCTTTTTTTAATACCTTACATTATTTCCATTACTCATCCTTTGCGTACTTTGGTGTTCCTAACTGCCCACTTCTTTTTGATTGATCCCCAGTATAGTGAGAAATACGGTGGATCTTTTGCATCCGCTTCAAGATATGACGACTAAGAAAATAATCTCAATCTTGGGGTAAACAACTTATGTTTACTTCAATTTTCTTCTTGTACCTAGGGAATGAGATTTTTATTGTTTTACTGCAAATTGAGGAGCAGTTTTGTTTCACTCATATAACTATCTGGTTTAACTCATCGAACTGAAATGTTAAAAAAAATATTTTTTTTTATTCTTTTATTTCATATTCATATTTAAATATTGAATTATATGAATTCTATTTCTATTTTATTGTATTTCAATTCATTTTTTATCTCTTTTCTAGAAAAGAGATAAAAAAAATTCATGTTCCAACGAATCACACGTAGAGATATTGCTAACACACATAGAGTTAATGGTATTTCATAACTAATCGATTGAGCTGTAGCTCGTAGACCACCTGAAAAGGAATACTTATTATTTGATCCATATCCTGACATAAGAAGACCAATGGGGACAATACTTGAAAAAGCAATCCATAAAAAAACACCTATACTGAGATCTGCTAAAACAAGGTGATATCCAAAAGGAATTACTAAATAACTTAGTAGAATTGATATAAAACCTATAGAAGGTCCGACCCTAAATAAACGAATATTACCTCTAGATGGAAGAAGATCCTCCTTCAAAAGTAGTTTGGTCCCATCCGCTAAAGCTTGAAGAATTCCTAAAGGGCCGGCATATTCAGGTCCAATACGTTGTTGTATTGCTGCAGATATTTTTCTTTCTAACCACACAATGACTAATACTCCCATTGTGATTCCTAAGACAAGGGTTAAAATGGGGACAAAAATCCATATGAGTCCATAGACTTCTTTTAAGGATTCTAATCTATAAAAAGAATTAATAGTTTGTACTTCTGTCGTATCAATTATCATTTCAACGATCAACTTCTCCCATAATGATATCTATACTACCTAGTATCGTCATGATATCAGCCAATTTCATTCTTTTAACTAGCTGGGGAAGAATTTGCAAATTGATGAAACCGGGTGGACGAATTTTCCATCTCCAGGGGAAAACACTATCATCTCCTATTAAATAAATTCCTAATTCTCCTTTTGGGGCCTCTACCCTTACATAAAGTTCTTGTTTTAACAATTCAAAATTGGGTGAAAGTTTTTTAGTAATAAATCTATATTCAAAATTATTCCATTCGGAATTCTTTGTCCTATGAAAACGCCGGTTTTCTAAATTCTCATAAGGTCCTCCAGGAATTCCTTCTAGAGCCTGTTGAATGATTTTTATGGATTCTTGCATTTCACCGATTCTTACTAAATAACGAGCTAATGTATCGCCTTCTTTTTGCCATTTTACTTCCCAATCAAATTTATTGTAACACTCATAGCGATCAACTTTACGAAGATCCCATTGGATTCCAGAAGCTCGTAACATTGGTCCTGATAAACCCCAATTTATTGTTTCCTCCCCACCAATAATGCCCACCCCTTCAACTCGTTCCAAAAAAATGGGATTTCGCGTAATGAGTTTTTGATACTCAACAACTTCTGTTAAAAAATAATCACAGAAATCAAAACATTTATCTATCCAGCCATAAGGTAAATCCGCAGCTACTCCTCCGATGCGGAAATAATTATGCATCATCCGCATACCTGTGGCGGCTTCAAATAGATCATATATTAATTCCCTCTCTCTTAAAATATAGAAAAAGGGAGTCTGTGCACCGATATCGGCCATAAAAGGGCCAAGCCATAACAAATGGGAGGCTATACGGCTCAGCTCCAGCATAATAACTCTGATATAACTGGCCCTTTTAGGCACTTGAACACTTTCCAACCGTTCTGGTGCATTTACTGTTATTGCTTCTGTGAACATAGTAGCTAAATAATCCCAACGTGTTACATAAGGCAAATATTGTATAATTGTTCGGTTCTCCGCTATTTTTTCCATCCCTCTGTGTAAATAGCCTAATATAGGTTCACAGTCAATAACATCTTCACCATCCAGAGTAACGATCAGCCGAAGAACACCATGCATTGATGGGTGGTGAGGGCCCATATTAACTATTATAAAATTTTTTCTTGTAACCGGTACAGTCATATTTTTTTCCTTAATTCATTATTTCATAAATTTCTTAAAATGTAAAATACAAAAAAAATAATAACTGAACTAATAAAAAAATAATTAAAAAAGAACAAGGATAATAATAAGAAAATAATAAGAAACTCAAAGAATAAATTCAAAATTAATTAACGAGTTTTTGGTTCCCTAATATCTAACTGATCCATTAATTTCTTATAACGCACTTTATTTTTCTTTGACAAATAAGTCAATAATCGTTGACGTTTTCCCAGAATTCTTCGTAGACCCCTTTGCGATAAAAAATCTCTTTTGTGCAATTCTAAATGTGAAGTAAGTCTCCGTATCTTACTGGTGAAATGTAATACTTGAAATTCAACAGACCCACTATTTTCTTCTTTTTCTTCTTGTGTAATAACTGAGATGAATGAATTTTTGACCATAAATTTAAATTTCTATCTTTCTTTTCTGTGAATTTTACCAATCAGGAAAAATAATAATATTTATTATGCCAGTTATTTTCATCTAGTATACATCAAAATTGGATTTCATTCATACACTACTTTGGTTTTTTCTTTATGTGGTTTATGTTTTTATGTTTTGTATATTTGGATCAAATATACAAAACATATATGTATTCACGAAAGAAAACACTTTCTTTTTTTACTTAAAAGGATTCCGCTCTTCCTAGCGAAAATTGATACACTATGAAATCAGCATCATGTATTAGAATATTACACAGTGTATATGTTTCGTCTTTCATCTACCGAATATGTTACACGATATGTAGGAAATCTACTATAAATTTTTTTCATTTTTCATTGGAATTGAATTAATTCTGAATTGTGAATACATATGTATTCTTGACATACTGAAACGACTGCTGTTATTGGTATCAAACCAATAGCGATTCATACAAGCTACATCTTCTAATCGATAATTGGGCCAAAGAAAAAATTTGAATTTAATGAAATTTTTTCTATTTGTATTAATATGTTTGTCCTCAGTCAAAAATTGCCCACAGTTTCTTATTTTGTTTTCATTGCAAAATCTTGGATTTCTATCCACAACATTAAAATTCTGGGAATTGAAACAAATTCGAATTCGAAATTCTCTACGACGTCTGGGAGATAGAATATTTTCAGGAACAAGTAAATCATAATGATTTTTGTCTCCACTCAAAAATATGTTGCTGTGTCGTGCAATGGGTTTTTCAAACCCCCTTTTCTCAATATATCTTTTTTTTGTGTATTTTTTATTTGTTTGGTGCTTCTTATTATCGACCAATGAAATATCCATAGTTTGATATATAATATATTTTCCGTCCCTTCGTATAGATAGACAAATTGGTTCAATAAAAAGTATTCCCTTTCTTATCAATTCTGCAAGAACTAGGTCCTTTTGAATCAGCATTTTGGCTAGATCTATTTCACCTCTTTGAATCGAAGATATAGCAAATTCCTTTGGATTTCTCAGTCTAAGTAGGAGACAATATACCCTGATATTTTTCATTATTTTATCATGTAAGGGATCAGCCCATCTTAGTTGAAAAAGGAAATATTTTTTCAAAAAGAAATCTAGTTCCATTTCATTCTTGTTCTTATATTGATATTGGTTTTTTTTTATTTCTAATCTTGCGTAATCTTCTTCAACATCTTTTTTATTTTTTTGTTTTAGTAGATTCCATACAAGATTTCTTTGGACCTGTTGTTCGTTTTCTTCCTGCTTGAAATTTCCTAATTCAAGATCTTTTTTTTTATTAGATGATTTTTTTGGATTTATGTTAATGTTTTTACTTTTTTCATTTCTAGAAAAATGAAAAAAGAGTGATTTGATTGGGATGATCCAAGGTTGAATTTTATATACATCAAAAAGTAGCACAAATTCTGGAAAGAACCAAGTTTCTAGATTGGATATAGTATCATGATTTGATGCGGTATCATATAACTTTTCTTTATTCATTCCCATGCAATCAAAAAAGAAACTTTTTTGATTGCATGGTTTGATCTCTTGATAAATTGTAGGAAAAAAAAGATCTTTTTTTTCCATTTTTTTTAAATTCTTAATTTCATTCTTAGTATTTTTCTGAATCTTGATGCCAATATGTATATCGGTCCAGATATCAATATTATTTATAAAACAAAGATGAAGAATTCTACAATCAAAATATTTTCTATCCAAATTTATATTCCTATCAATAAGATATCCTTTTTCTATATAATCATTACTAGGTATACTTACCAATATATAAAATGATTCAGGTTTCGATATATTGAAATGATATGGAATTTCTTGGTCCCCATTTCTTTCTAATGTTGATCCAGAAATGTATAAATTAGGGAGACATATGTATTTATATGATAAAAGATCATATCTGTAATGTTTTTTCCATTTGTCTTTTTGACTCATAAATAAATTTGCTGCATAGTCATTTTTATTCATGGAATAAATAAATTGGTATTTTTTATATAAATCCAATTGGTTTGATTCCTTATTTTGAATCATACAATGTTTAATTATTCTATTTCGGAATTCTTTAGGTACTAATCGAGACATTTTTTTTTGAGATAAATCGTATTGATAATGACCTTTTAACCAGTTTTTCCATTCGTTCATTCCATACGTATGAATTTTATTATGTCTTGATTTGGAATTAAATATTCCATATATCATAAAATAGTCTTTGAAATTATCCTTAAAAAAAGGATAGCTCCCTTCATATTGAAGTACAGGTTTCAAATTATACTTATTAAGTAATTGGTTTTGTGATATTTTGTAAAAAACATATGCTTGGGACAGGGAAGAGAAGTTCCCATAAGTATTGGAATTTTGATTGATATTCTTAGTATTATCAGTATTTGAAAACAATTTTTTTATAGTCAAAATCAAATTCATTGTATTTTGATTTGTTTCATCAATTCTTTCTTGTTCTTTATTTATTTCATTGTTGTAAATGGATTTATTAAAGATCTTTTTTGTTGATTCAAAAAAAAGTTGTGCATTGATCTTAGAAACGGTAATGGTACATAGCAAAATATCTATGTATATTTTTTCAATGAATGATTTAATAAAGTAGTATGATTTACGCATTAATCGGATATTTTTCCTTTTTAATATTTTCCAAATATGTTTCTGTGATCCCGATCTTTTATTATCATAAATTAGTTCTTTTTTTTTCTTGTATTTTGCGGTTTGTTCAATTTGATTCCTTATTTTGATTGTCTTATCAGAAAGATCTTTCATTCTTCTTTCTATTAGTGAATTATTTAACCAATTCATCGTTCGATTTAGAACGGACGATTCACGAAGAATCTTATTATTTCTTTTGAAATCTTTTTTGTTTTCGTTCGGTTCATATACTTTTTCTTTCCTCAATTCAAATAAGAAATTTGGATTTACTTTCTCCAGTTTTTTCATTATTAGTTTGATAACTCGAACTATTTTGATGACTCCTTTTTTTTTTTCTTTTCTAACTTTTAGAAAGGGTTTTCTTTTTTTATTGAAAGATTTTAGAACTTGTAAAAATTCCTTTTTCACCTTTTTTTTTCTTTTTTGGAGTTCTTTATAAATAGGTTCAAAAAAAAAAGGTCGTTTTCGGGGAGAACCAAAGGGAAGTTCCGCTTCCATTCCCCAGATTGTTAAAAAACAAAAATTTATTTTTTTTTTCATTAGATCTCTATGATGAGATCGTGCCTTATATTTTCTCCAAGGTTTTAGACAGAAAGGATATATAATCTTTATCTGAATACCATCTATTAACCAATTTTGGGGAAACTCTTTTTCTGATAATTGAACACCATTATAGGTGCATTTAATATGGATTTCTTTATTCCATTCCTTAAAATCCTCGTCCCACTCGGGAACTTGTAATAATAACATACGGAAAATATTTTTGGCTATTATCAATGAAGGTAATATAATGTTTTTTCTAAGAAAAGATTGGGTTACTAATATCAAGCCTCTTATTACTTGAGCAAATAGAAAGGTATTCCAAACTTCTGATATTTCTGCTTGTTCATTTATATATATTTTTTCCTCTTTCTCCTTTTTTTCTTTTGTATCTTTATTTTCAAAATAGGAAATTTTTAGTTCTGATTCTTGTTCTCTGTCCATCCAATTCCTAAAAATGATATTCTTCATTTCGTTGATATCAAAAAACGAAAAAAAAAATGTTTTGTCTAGACGATCCAAAAAAAGTGGGGAATGTACATTTACTTGAAAGAGGTTCCAAATAACTGTTTTACGTCTTTGAGCGCGCATGGATCCTTTGATTAGATTGCGGCGAAAATCCGATTGTTTTGAGTAACGTACCAAAGACACCTCTTCCATTTGATCATCATTTTTCTCAGTGTTACTAAGATTCTGAATACTATAAATAGAAAAAAAATTGGTATTATGATCATTATCGTTAGCAATTATCACACGTCTGGCTCTTCTTGAATGAATCGAAAAATCTTCTTCCTCCAATACTTCTTCATTTTCTTCTTCCTCTTCTTCCAACAAACTCTCGGTTAATTTGTATGACCATCGAGAAACCTTTTTACTGATTTCTTCTATTCTAATTCCAACAGATTCCTTTTTCATTTTTTTTTGATCTTTTGTATGTGTTGTAATTATATCAAATACAAATTGCAAATATTTTGCTTGACTTTCTGAATCAATTCCTTTTTCTTCTGTAGAATTCAAAAATGATTGAGGGTGAAGTTTTACGGAATCATTAAGAAGTAGATCATGAATCTTATTTAGAAAAAAAAATTCTACTAAATCTTCTGTATTTGTATAAGTATTTATGATTGCGCGTGAATAGAATTTTTTTCTCATTCCTCGATATGGTCCGTTGAAAAAAGGATCATATGCTTTTGACAAGCATTTTTTTTTTTTTTCATCGTCACATAATATGGTTCTTTTTTCAAGTATATCCAGACTAGGGGATACTTCATTTTTTTCTATAATTTTGATTCGGCTTCTCAATTCATTGTTCAAATTGCGCTTTTTTTTTTCATTAGTATAAATCCAAGAATCATAAAGATCTTCGTCATATAATTTTTCTATTATGTACAAAGAAATTCTTCGTTCTAGCATTTCCGAAAAAGTTGATAAACTGGGCGGGTATGTAAAGGATATTTTTTGTTTCCCATCACTTGTACATGTAAAAAAAAAAAATTGTGACATTCCATTGCGTAAAGCATTTTCTAATTTATAATTTCTTATATATCGTAATGGACGATTCCATCGTTTATAATCGAAAAGAAAAGAAAGAAAAGTTTTTTCAACCCACAACATTATTTTCTTTTTCTCTTCTTTCAGTCTTCCCAATTCCCAATTTTCTTGATGGGTCTCCAGATCAGAATCTTCGTAAACTGGGCTATCTTGATAGCGTGCCTCTTTCAAGTGAAATTCATCCTTTGTTTTTTCCTTTCCATTCACTCGGATCTCTTCCGTTTCATCTATTTTGTCCAGATCCTCCTTTTCTTCCGAACAAAGGTTTTCTTCGGTGGATCCCTCTTGTTCCTGTTTAGTCTCCTTCATTTCGTAAGTTGTTTCTACATCGCTTTCTTCCGTTTCTGAGGTTTCTTTCTCTTTCAGTTTCTTAGTGACAATAGGCGACGGCATTCTGCCTAAATAGTAAACACAGGTGATAAATAAGAGAATACTAAAGATTCGAGCCATAGAATTTCTCAATTCTGACACAAGATACTTATTAGATCGAATAGAATGATTTTGCCGTATCCAGAATAATACCAATCCAACCCATTTCATGAATAAAATGTGACCAATTAACCAACCAACAAAACTACTTGTTAAAAATAAAATCTTGTTGTTGCATCGAAACATATAAATGTTGACTAATCTGGCTAACGTGGAACTTGGTAAAATGAAATGGTTGAATAATTGAAGAATTAGATTATTCAGGAATACACATTGAATGCTGAGATTACGCATTGAATTTCTGGTAGTAGATCCATAATCAAAAAAGTTTTTATGATTGTTCCAGAAGAAATGAAACAAAAGATACGGTAGAACTAGGACAGTTATTGTATGAGGTCTACCCAATGCTAGATGCAGAGGCGCATAATAGATCGATATGAACATCATGAGCTGTCCCGCAATAAAACCAGTTGTTGCTGATACCTCCTTCTCGGTTCCTTCTTCCATAACCCGAGCTCGGAGAAGGAAGAGATAAGAGGGCCCTATGGAGAATGTGGTCAGAAATCCATAATAGAGCCCGACCACAACGACCGAATTTATTATCTTCATGCATAAGGATAATGGATTACCTAGTAGAAAAGATTTCAAAATCATCACAAACCTCCCCTTTTTCTTTTCTATTGCAATTTCTCGATTATTATATGATGATTCCTTAACTTTCCATATCTATATAGATAGAAACAGATATACTATAAATGACATCTCTTATGTCAATGACACAAAAGGGATATGAAATGAATGGAATTGGGATATAGATGGAATATAATGAAATAGAGCCACATTGAGGTTCCCTATGAAATGAGGCATGGAACGGAGCCACTACGAAGAAGTTCCTGGAGTTACGAAGGAAGCTTCGGACTCATATTGTTCATGGGTTGAGAACGGGAGTTGAACTCTATGAGGTCGAATCTCCCGTTGTTCCTCAGTAGCTCAGTGGTAGAGCGGTTGGCTGTTAACTGACTGGTCGTAGGTTCGAATCCTACTTGGGGAGATTTGATTCATTCTTTCATGAAGAATGAAGAATTGAATGAAAGGGCTCGCTTTGACCGTTAGGAGTAGGTAACTCGTTCCCTGTCTTTGTTTCTATTGCATTCTATCTCATCGTATCACATTCTGTTCTACAATATTTGAGAATTAACCTCAATACCTCGGCATAGATCCAACCCTTGTAAATAGCCAATTCAGAATTCTCAACAGTGCATCAAAGATGCAGTCATCAATTCTCCCGATAGGCCACAATTACCGCGAGCAAACATATTAATGACGAGGAACGCATTTTTGCTATGCTACTAATACTTGTACTTGCTCTGCTATTCTGCCCAAGCCTGGCTGAGGAAGAGTTACGGGGAGTCAAACACAAATATGCTGATTCCAGCCCGGGTTACTATATGTAATAAAATAAACCTGACTTATAAGGACCATACCCAAGTTCAATAGCTTTGTGGCCGCTATCCCGATCATGATTTTACTACCCCCAGAGGGAAAGGCCCTTCCCTTTTGGGCCGGTTGTGGGCGAGGAGGGATTCGAACCCCCGACACCGTGGTTCGTAGCCACGTGCTCTAATCCTCTGAGCTACAGGCCCCACCCCGTCTACACTGGATCTGTTCCCGGGAGTACCTTCAAAAAGGAGCCTTTCCTCTCCTCAGCCATTTTGGGTTAAGAAGATGTGAAAGCGCCTTTCTCTCTAGAATAACAGTGCGTTCCGAGGCGTGAAGTGGGAGAGAAGGGATGTTATTGAATAAGACGACCTTTGCATTTTTTATTTTGATCTTTTGAAATATGAAAAAGTAAAATAAGAGGTGTTAAGCTTTTTATCATTCTGGCGTCGAGCTATTTTTCCGCAGGACCCCCCCTACAGTATCGTCACCGCAGTAGAGTTTAACCACCAAGTTCGGGATGGATTGGTGTGGTTCCTCTACGCCTAGGACACCAGAATATCGAACCATGAACGAAGAAAGGCATGAGATAAATATTGTCTAGTGATTGTGAGGCCCCGATTCTTGACTGGAAGGGACGCCAAAGGCCTCTGCCCTTCCATCCGATAGAGAGGGAGGGCAGAGCTTTTGGGTTTTTGTTTCATGTTGTCAAACAGTTGAACAATGAAAATAGATGGCGAGTGCCTGATCTAATTGATCAGGTCGTGTAGGAACAAGGTTCAAATCTCTCGGTCTGTTAGGATGCCTCAGCTGCATACATCACTGCACTTCCACTTGACACCTATCGTAATGATAAACGGCTCGTCTCGCCGTGACCTTATCTTGGATTCTCAATACTTCTGTCGCTCCATCCCCGCAGGGGCAGAGAACCCGCCGCTGTCTCGGCTGTGCTACCGGAGGCTATGGGGAAGTCGGAATAGGAGAGCACTCATCTTGGGGTGGGCTTACTACTTAGATGCTTTCAGCAGTTATCCGCTCCGCACTTGGCTACCCAGCGTTTACCGTAGGCACGATAACTGGTACACCAGAGGTGCGTCCTTCCCGGTCCTCTCGTACTAGGGAAAGGTCCTCTCAATGCTCTAACGCCCGCACCGGATATGGACCGAACTGTCTCACGACGTTCTGAACCCAGCTCACGTACCGCTTTAATGGGCGAACAGCCCAACCCTTGGAACATACTACAGCCCCAGGTGGCGAAGAGCCGACATCGAGGTGCCAAACCTTCCCGTCGATGTGAACTCTTGGGGAAGATCAGCCTGTTATCCCTAGAGTAACTTTTATCCGTTGAGCGACGGCCCTTCCACTCGGCACCGTCGGATCACTAAGGCCGACTTTCGTCCCTGCTCGACGGGTGGGTCTTGCAGTCAAGCTCCCTTATGCCCTTGCACTCGAGGGCCAATCTCCGTCCGGCCCGAGGAAACCTTTGCGCGCCTCCGTTACCTTTTGGGAGGCCTACGCCCCATAGAAACTGTCTACCTGAGACTGTCCCTTGGCCCGTAGGTTCTGACACAAGGTTAGAATTCTAGCTCTTCCAGAGTGGTATCTCACTGATGGCTCGGGCCCCCCCGCAAGGAGGCCTTCTTCGCCCTCCACCTAAGCTGCGCAGGAAAGGCCCAAAGCCAATCCCAGGGAACAGTAAAGCTTCATAGGGTCTTTCTGTCCAGGTGCAGGTAGTCCGCATCTTCACAGACATGTCTATTTCACCGAGCCTCTCTCCGAGACAGTGCCCAGATCGTTACGCCTTTCGTGCGGGTCGGAACTTACCCGACAAGGAATTTCGCTACCTTAGGACCGTTATAGTTACGGCCGCCGTTCACCGGGGCTTCGGTCGCCGGCTCCCCTGTCATCAGGTCACCAACTTCCTTGACCTTCCGGCACTGGGCAGGCGTCAGCCCCCATACATGGTCTTACGACTTTGCGGAGACCTGTGTTTTTGGTAAACAGTCGCCCGGGCCTGGTCACTGCGACCCCCTTTTTGAGGAGGCACCCCTTCTCCCGAAGTTACGGGGCTATTTTGCCGAGTTCCTTAGAGAGAGTTGTCTCGCGCCCCTAGGTATTCTCTACCTACCCACCTGTGTCGGTTTCGGGTACAGGTACCCTCTTGTTGAAGGTCGTTCGAGCTTTTCCTGGGAGTATGGCATGGGTTACTTCAGCGCCGTAGCGCCTGGTACTCGGACATTGGCTCGAGGCATTTCCTCTACCCCTTCTTACCCTGAAAAAGCAAGGTCACCTTGCGTCCTTGAACCGATAACCATCTTTCGGCTAACCTAGCCTCCTCCGTCCCTCGGGACCAACAAGGGGTAGTACAGGAATATTCACCTGTTGTCCATCGACTACGCTTTTCGGCCTGATCTTAGGCCCTGACTCACCCTCCGTGGACGAACCTTGCGGAGGAACCCTTAGGTTTTCGGGGCATTGGATTCTCACCAATGTTTGCGTTACTCAAGCCGACATTCTCGCTTCCGCTTCGTCCACACCTGCTTGCGCGGGTGCTTCCCTCTAAGGCGGAACGCTCCCCTACCGATGCATTTTTACATCCCACAGCTTCGGCAGATCGCTTAGCCCCGTTCATCTTCGGCGCAAGAGCGCTCGATCAGTGAGCTATTACGCACTCTTTCAAGGGTGGCTGCTTCTAGGCAAACCTCCTGGCTGTCTCTGCACCCCTACCTCCTTTATCACTGAGCGGTCATTTAGGGGCCTTAGCTGGTGATCCGGGCTGTTTCCCTCTCGACGATGAAGCTTATCCCCCATCGTCTCACTGGCCGACCTTGACCCCTGTTTGGGGTCATATCTAGTATTCAGAGTTTGCCTCGATTTGGTACCGCTCTCGCGGCCCGCACCGAAACAGTGCTTTACCCCTAGATGTCCAGTCAACTGCTGCGCCTCAACGCATTTCGGGGAGAACCAGCTAGCTCTGGGTTCGAGTGGCATTTCACCCCTAACCACAACTCATCCGCTGATTCTTCAACATCAGTCGGTTCGGACCTCCACTTAGTTTCACCCAAGCTTCATCCTGGTCATGGATAGATCACCCAGGTTCGGGTCCATAAGCAGTGACAATCGCCCTATGAAGACTCGCTTTCGCTACGGCTACGGTGGATTCCCTTAACCAAGCCACTGCCTATGAGTCGCCGGCTCATTCTTCAACAGGCACGCGGTCAGAGATCACATTCTCCTCCCACTGCTTGGGAGCTCACGGTTTCATGTTCTATTTCACTCCCCGATGGGGGTTCTTTTCACCTTTCCCTCACGGTACTACTTCGCTATCGGTCACCCAGGAGTATTTAGCCTTGCAAGGTGGTCCTTGCTGATTCACACGGGATTCCACGTGCCCCATGCTACTCGGGTCAGAGCGTAAGCTAGTGATGCTTTCGGCTACTGGACTCTCGCCATCTAGGGTGCAGCACTCCACCGCTTCGCCTAGCAGCATGACGCTTTTATTGCTCTCCCACAACCCCGTTTTCACGGTTTAGGCTGCTCCCATTTCGCTCGCCGCTACTACGGGAATCGCTTTTGCTTTATTTTCCTCTGGCTACTAAGATGTTTCAGTTCGCCAGGTTGTCTCTTGCCTGCCCATGGATTCAGCAGCAGTTCGAAAGGTTGACCTATTCGGGAATCTCCGGATCTATGCTTATTTTCAACTCCCCGAAGCATTTCGTCGCTTGCTACGCCCTTCCTCGTCTCTGGGTGCCTAGGTATCCACCGTAAGCCTTTCCTCGTTTGAACCTCGCCATTCACGTGAAGGCTATGCCATCCTAAGGTGCTGCTAAATGGAAGGATCTTATCAACGTCCATGAATGAGAAATCATAGATCGAACTGCCGAATTGGAAAAATTGGATGCTATCATAGTATCAGCTAAGTTCACGGGCTGGAGATAAGCGGACTCGAACCGCTGACATCCGCCACAGGGTAAACCACCGCCTCTCAGGCCTCTCCGGCTGATTCTACCATAGAGGCCAACAATAGACAATAACTCCCCCCCGAACACAGCTTACAACTTTCATCGTACTGTGCTCTCCAAAGAGCAACTCTTCTCAAAATCTCAAAGGGTGCTGAGTTGGAATACCATTCTAAGGATCCTTGTGGTTCCAGCTACAGGAGAACCAGGAACAAAGAGCTCTCCCCCCTTTTCCGCCCGACTCTTTGGTCTTCAGAATGCTGGTTTTAAGAACGAGTGATTGCCCTTCTCCGACCCTTACTGCCCAACCGGAGAGCGGACAGCTAATGCCTTCCACTTATTGAACAGGGTCTATGGTCGGTCCGTGACCCCTGGATGCCGAAGGCGTCCTTGGGGCGATCTCGCAGTTTCTACGGGGTGGAGACGATGGGGTCGGTCCATGGATTTTCCTTCCTTTTGCCACATTTCGCTCAAAGGGTTGAAGGGAGATAGTGCATCAAGCTATTCGCAAGGGCCAACTTGATCCTCTTTCCTAGGGATCCCAGATGAGGGAACCCTAGGAGAGCCGCCGACTCCAACTATCGTCCATGTACGATCCATACTAGATCTGACCAACTGCCCATCCTACCTCCTCTACGTTCTTGACAGCCCATCTTTTTGTCTCAGTAGAGTCTTTCAGTGGCATGTTTCGGTCCTCTTCCCCATTCCTTAGAAAAAGTGAGCCACCGGTTCAGGTACAAGATACTATCATTACCGCCTGGACAATTAGACATCCAACCCGTAATCGCAACGACCCAATTGCAAGAGCGGAGCTCTACCAACTGAGCTATATCCCCCCGAGCCGAGTGAAGCATGCATGAAAGAGTCAGATGCTTCTTCTATTCTTTTCCCTGGCGCAGCTGGGCCATCCTGGACTTGAACCAGAGACCTCGCCCGTGAAGTAAATCATCGCACCTACGATCCAACCAATTGGGAGAGAATCAATAGATTCTTTTTCGGGAGCGATTCATCCTTCCCGAACGCAGCATACAACTCTCCATTGTACTGCGCTCTTCAAGTGTGCTTGTTCCCCTTTCTCCCTTACCATGGCAAGTCTTTGTGAAATCACTCCGATGGGAAGAAAAAAGAAAGCGTTAAGAGACCCTCCTGGCCCAACCCTAGACACTCTAAGATCCTTTTTCAAACCTGCTCTCCTCCCATTTCGAGTCAAGAGATAGATAAATAGATACATCCCATTGCACTGATCGGGGAGCGCTCGTAGTGACTGAGGGGGTCGAAAACCAAGAAGTGAGTTATTTATACCAAGCATTCTTCTTATGTTATGGCTAGATCCAATCTCCTGGTCCCTGCGGAAAGGAAAAAGAATTTCACGTTCTTCCTTTCGGGAAGGGAGGATTCGGGAAATCCTATTGATTGCTGCTTTCTTCAGACCTCCGGCATGAAAAAAAGGCTCGAATGGTACGATCCCTCCGTCACCCCAGAATGAAAGGGGCGATCTCGTAGTTCTTGGTCTGTGAAGATGCGTTGTTAGGTGCTCCATTTTCCCATTGAGGCCGAACCTCAACCTGTGCTCGAGAGATAGCTGTCCATACACTGATAAGGGGTGTATGGATTCTCGAGAAGAGAGGAGCCGTGGGGGCCCCCCCCCGGACCGCCCGGATCCCACGAGTGAATAGAAAGTTCGATCTCCATTGGATCTCACCTGAATCGCCCCATCTATCTTCCTGAGGAGAAGTTTGGTTTCAAACTCCGATTCGAACAGGAGGAGTACGCCATGCTAATGTGCCTTGGATGATCCACATCTCCGGGTCAGGCGCCGATGAGCACATTGAACTATCCATGTAGCTGAGAGCCCTCACAGCCCAGGCACAACGACGCAATTATCAGGGGCGCGCTCTACCACTGAGCTAATAGCCCGTCGTGTGGGCCTCCCTGGGGGAGGCCTGCTATGCCAAAAGCGAGAGAAACCCCATCCCTCTCTTTCCTTTTGTACGTCCCCATGTCGCCACACGGGAGGGACATGGGGACGTACAAAAGAGATCCTATCAACTTGTTACGACCTAGGATAATAAGCTCATGGGCTTGGTCTTACTTCACCGTCGAGAAACGAAAGAAGACTTCCATATCCAAGCTTAGCTCAGATGTAGCTCGCTCCTTTTTGGGTGTCAAGCAGTGTCAAACCAAAATACCCAACAAGCATAAGCATTAGCTCTCCCTGAAAAGGAGGTGATCCAGCCGCACCTTCCAGTACGGCTACCTTGTTACGACTTCACTCCAGTCACTAGCCCTGCCTTCGGCATCCCCCTCCTTGCGGTTAAGGTAACGACTTCGGGCATGGCCAGCTCCCATAGTGTGACGGGCGGTGTGTACAAGGCCCGGGAACGAATTCACCGCCGTATGGCTGACCGGCGATTACTAGCGATTCCGGCTTCATGCAGGCGAGTTGCAGCCTGCAATCCGAACTGAGGACGGGTTTTGGAGTTAGCTCACCCTCGCGGGATCGCGACCCTTTGTCCCGGCCATTGTAGCACGTGTGTCGCCCAGGGCATAAGGGGCATGATGACTTGACGTCATCCTCACCTTCCTCCGGCTTATCACCGGCAGTCTGTTCAGGGTTCCAAACTCAATGGTGGCAACTAAACACGAGGGTTGCGCTCGTTGCGGGACTTAACCCAACACCTTACGGCACGAGCTGACGACAGCCATGCACCACCTGTGTCCGCGTTCCCGAAGGCACCCCTCTCTTTCAAGAGGATTCACGGCATGTCAAGCCCTGGTAAGGTTCTTCGCTTTGCATCGAATTAAACCACATGCTCCACCGCTTGTGCGGGCCCCCGTCAATTCCTTTGAGTTTCATTCTTGCGAACGTACTCCCCAGGCGGGATACTTAACGCGTTAGCTACAGCACTGCACGGGTCGATACGCACAGCGCCTAGTATCCATCGTTTACGGCTAGGACTACTGGGGTATCTAATCCCATTTGCTCCCCTAGCTTTCGTCTCTCAGTGTCAGTGTCGGCCCAGCAGAGTGCTTTCGCCGTTGGTGTTCTTTCCGATCTCTACGCATTTCACCGCTCCACCGGAAATTCCCTCTGCCCCTACCGTACTCCAGCTTGTTAGTTTCCACCGCCTGTCCAGGGTTGAGCCCTGGGATTTGACGGCGGACTTGAAAAGCCACCTACAGACGCTTTACGCCCAATCATTCCGGATAACGCTTGCATCCTCTGTCTTACCGCGGCTGCTGGCACAGAGTTAGCCGATGCTTATTCCTCAGATACCGTCATTGCTTCTTCTCCGAGAAAAGAAGTTCACGACCCGTGGGCCTTCTACCTCCACGCGGCATTGCTCCGTCAGGCTTTCGCCCATTGCGGAAAATTCCCCACTGCTGCCTCCCGTAGGAGTCTGGGCCGTGTCTCAGTCCCAGTGTGGCTGATCATCTTCTCGGACCAGCTACTGATCATCGCCTTGGTAAGCTATTGCCTCACCAACTAGCTAATCAGACGCGAGCCCCTCCTCGGGCGGATTCCTCCTTTTGCTCCTCAGCCTACGGGGTATTAGCAACCGTTTCCAGTTGTTGTTCCCCTCCCAAGGGCAGGTTCTTACGCGTTACTCACCCGTCCGCCACTGGAAACACCACTTCCCGTCCGACTTGCATGTGTTAAGCATGCCGCCAGCGTTCATCCTGAGCCAGGATCGAACTCTCCATGAGATTCATAGTTGCATTACTTATAGCTTCCTTGTTCGTAGACAAAGCTGATTCGGAATTGTCTTTCATTCCAAGGCCGAAGGCTTGTATCCATCCATGCGCTTCATATTAGCCTGGAGTTCGCTCCCAGCAATATAGCCATCCCTACCCTATCACGTCAATCCCACAAGCCTCTTATCCATTCTCGTTCGATCACGGCGGGGGAGCAAGTCCAAATAGAAAAACTCCCATTGGGTTTAGGGATAATCAGGCTCGAACTGATGACTTCCACCACGTCAAGGTGACACTCTACCGCTGAGTTATATCCCTTCCCGGTCCCATCGAGAAATAGAACTAACTAATCCTAAGGCAAAGGGTCGAGAAACTAAACGCCACTATTCCTGAACAACCTGGAGCCGGGCCTTCTTTTCGCACTATTATGTTATGGATAGTCAAATAATGGGAAAATTTGGATTCCATTGTCAACTGCTCCTATCGGAAATAGGATTGACTACGGATTCGAGCCATAGCACATGGTTTCCTAAAATCCGTTTTCCCGATCTAAATCGAACAGTTTTTACATGAAGAAGATTTTTTTCAGCATGCTCTATTCGAGACTGGTAGGAGAAGAACCGACTCGGTATTGTAAAAAAAAAGAGTGGAAGCAGAACCAAGTCAAGATGACACCCCTTCTTCTTGCGCCAAGGATCTTACTACCATTTCCGAAGGAACTGGAGCTACTTTTCTTTTCCATTTCCATTCCAGAGTTCCTATGTGACGCCCTTTTCTTAGGAACACATACAATAAAAAAGGATAACGGTAACCCCACCATTAACTACTTCATTTTCATTTATGAATTGAATAGTAATAGAAATACATGTCCTACCGAGACAGAGACAGAATTTGTATCCTCTTGCCTAGCAGGCAAAGAATGTAGGACTGGTGCCAACAGTTCATCACGGAAGAAAGGACTCGCCGAGCCGGGATCACTAACTAATACTAAAACTAAGACTATAAGACTAATATAACTAATCTAATTAGAAATAATTCTAAGAATTAGATAATTCTAATAGAATTTAGAATAGAATATAGAATATAGAATAATATATATAATAATAATATATAATTCTAATAATAGAATATAGAATAATACATAATAGAAAAGATATAATAGATAAAGAACTGTCTTTTCTGTATACTTTCCCCGGTTCCGTTGCTACCGCGGCCCTTACGCAATCGATCGGATCATATAGATTTCAACACAACATAGGTCATCGAAAGGATCTCGGACGACTCACCAAAGCACGAAAGCCAGGATCTTTCAGAAAATGGATTCCTATTTGAAGAGTGCATAACCGCATGGATAAGCTCACGCTAACCCGTCCATTTTGGATCCAATTCGGGATTTTCCTTAGGAGGTATCGGGAAGGAATTGGTCTGAGTTACTCTTCGGGACGGAGTAGAAGAAGGGAGGAGATTCTCGAACGAAGAAAAGGATCCAATTACTTCGAAAGAATTGAACGAGGAGCCGTATGAGGTGAAAATCTCATGTACGGTTCTGTAGAGTGACAGTAAGGGTGACTTATCTGTCAACTTTTCCACTATCACCCCCAAAAAACCAAACTCTGCCTTACGTAAAGTTGCCAGAGTACGATTAACCTCTGGATTTGAAATCACTGCTTATATACCTGGTATTGGCCATAATTCACAAGAACATTCTGTAGTATTAGTAAGAGGAGGAAGGGTTAAGGATTTACCCGGTGTGAGATATCACATTGTTCGAGGAACCCTAGATGCTGTCGGAGTAAAGGATCGCCAACAAGGGCGTTCTAGTGCGTTGTAGATTCTTATCCAAGACTTGTATCATTTGATGATGCCGTGTGAATCGCTAGAAACATGTGAAGTGTATGGCTAACCCAATAACGAAAGTTTCGGAAGGGGACTGGAGCAGGCTACCATGAGACAAAAGATCTTCTTTCTAAAGAGATTTGATTCGGAACTACTATATGTCCAAGGTCCAATATTGGAATAATTTAAGAGGTTTTCCCTTCCTTTGTCCGTGTCAACAAACAATTCGAAATACCTCGACTTTTTCAGAACAGGTCCGAGTCCAATAGCAATGATTCGAAGCACTTCTTTTTCCATTACACTATTTCGGAAACCCAAGGACTCGATCGTATGGATATGTAAAATACAGGATTTCCAATCCTTGCAGGAAAAGGGGGGAAACGGATACTCAATTTAAAGTGAAAGTGAGTAAACAGAATTCCATACTCGATCTCATAGATACATATATAATTCTGTGGAAAGCCGTATTCGATGAAAGTCGTATGTACGGCTTGGAGGGAGATTTTTCATATCTTTTTAAATCCACCCTACAATATGGGGTCAAAAAGCCAAAATAAGTGATTTGTTTTTAGCCCTTATCAAAAGAAAACTGATTCTTGAACCTCTTTCACGCTCATGTCACGTCGAGGTACTGCAGAAGAAAAAACTGCAAAATCCGATCCAATTTATCGTAATCGATTAGTTAACATGTTGGTTAACCGTATTCTGAAACACGGAAAAAAATCATTGGCTTATCAAATTATCTATCGAGCCGTGAAAAAGATTCAACAAAAGACAGAAACAAATCCACTATCTGTTTTACGTCAAGCAATACGTGGAGTAACTCCTGATATAGCAGTAAAAGCAAGACGTGTAGGCGGATCGACTCATCAAGTTCCTATCGAAATAGGATCTACACAAGGAAAAGCGCTTGCCATTCGTTGGTTATTAGGGGCATCCCGAAAGCGTCCGGGTCGAAATATGTCTTTCCGATTAAGTTCCGAATTAGTAGATGCTGCCAAAGGCAGTGGCGATGCCATACGCAAAAAGGAAGAGACTCATAGAATGGCAGAGGCAAATAGAGCTTTTGCGCATTTTCGTTAATCCATGAACAGGATCTATATAGACACATAGATCCATGGATCCATACATCTCGATCGGAAAAGAATCAATAGAAAAAGAAAGAATCGGAATTGATCGAGATCTTTCTCGAAACAAACAAAAAGAAAAAGAAAGATGAAACATAAATCATGGATCAACTAAGCCTTCTCGGGGGCTTGCTTAAGAATAAGAAAGAGGAATCTTATGGAAATATCATGGAATAAGGTTTGATCCTATTCATGGGGATTCCGTAAATATCCCATTCCAAAAATAGAAAGTTCGAAACAATTGGGACTTTTTCGTAGATTGGATGCAGTTACTAATTCATGATCTGGCATGTACAGAATGAAAACTTCATTCTCGATTCTACGAGAATTTTTATGAAAGCGTTTCATTTGCTTCTCTTCCATGGAAGTTTCATTTTCCCAGAATGTATCCTAATTTTTGGCCTAATTCTTCTTCTGATGATCGATTCAACCTCTGATCAAAAAGATAGACCTTGGTTCTATTTCATCTCTTCAACAAGTTTAGTAATGAGCATAACGGCCCTATTTTTCCGATGGAGAGAAGAACCTATAATTAGCTTTTCGGGAAATTTCCAAACGAACAATTTCAACGAAATCTTTCAATTTCTTATTTTACTATGTTCAACTCTATGTATTCCTCTATCCGTAGAGTACATTGAATGTACAGAAATGGCTATAACAGAGTTTCTGTTATTCGTATTAACAGCTACTCTAGGAGGAATGTTTTTATGTGGTGCTAACGATTTAATAACTATCTTTGTAGCTCCAGAATGTTTCAGTTTATGCTCCTACCTATTATCTGGATATACCAAGAGAGATGTACGGTCTAATGAGGCTACTACGAAATATTTACTCATGGGTGGGGCAAGCTCTTCTATTCTGGTTCATGGTTTATCTTGGCTATATGGTTTATCTGGAGGGGAGATCGAGCTTCAAGAAATAGTGAATGGTCTTATCAATACACAAATGTATAACTCCCCAGGAATCTCAATTGCGCTTATATCCATCACTGTAGGAATTGGGTTCAAGCTTTCCCCAGCCCCTTTTCATCAATGGACTCCTGATGTATACGAAGGAGTGCGGTTCGTTCGACAAATTCCTACCTCTATATCTATCTCTGAGATGTTTGGATTTTTCAAAACTCCATAGACATGCAGAAGAGAAATGCTATCCCCACTCGGACCAAGACATCACTTTGACCAAAAGTTTATTGTGATCCTTTTGTTCAAATAACAATTAAGGTGAAGCAGGGTCAGGAACAACGAATCTCTTTATGATAAACAGATCCCTTTTGCAAGTTCGTTATTACGGGTAGTTCTACAAAGGATCGGACTAATGACGTATACAATACTTGAATTATCGATGTAGATGCTACATAGTTGGTTCTCATCCTTCAGAGACTACGAGTGTAATAGGAGCATCCGTTGACAAAAGGATCACCCTAAGATGATCATCTCATGGCTATGGAGAACGAATCAAATCAGATGGTTCTATTTCTCAATCTTTCTGACTTGCTCCTACGGAACCAAGGTCGAAAGGATTGAAAAAGTCAGTCATTCACAACCACTGATGAAGGATTCCTCGAAAAGTTAAGGATTAGTAATCCTTTTTAGAAATCGAATGGATTCGGTCTTATACATACGCGAGGAAGGTAATCAAAAAAGAAAGAAGAACTACTCTTCTTTCTTTTATCACTTAGGAGCCGTGCGAGGTGAAAGTCTCATGCACGGTTTTGAATGAGAGAAAGAAGTGAGGAATCCTCTTTTCGACTCTGACTCTCCCACTCCAGTCGTTGCTTTTCTTTCTGTTACTTCGAAAGTAGCTGCTTCAGCTTCAGCCACGCGAATTTTCGATATTCTTTTTTATTTCTCATCAACCGAATGGCATCTTCTTTTGGAAATCCTAGCTATTCTTAGCATGATATTGGGGAATCTCATTGCTCTTACTCAAACAAGTATGAAACGTATGCTTGCATATTCGTCCATCGGTCAAATCGGATATGTAATTATTGGAATAATTGTTGGAGACTCAAATGATGGATATGCAAGCATGATAACTTATATGTTGTTCTATATCTCCATGAATCTAGGAACTTTTGCTTGCATTGTATCATTTGGTCTACGTACCGGAACTGATAACATTCGAGATTATGCAGGATTATACACGAAAGATCCTTTTTTGGCTCTCTCTTCAGCCCTATGTCTCTTATCCCTAGGGGGTCTTCCTCCACTGGCAGGTTTTTTCGGAAAACTCTATCTATTCTGGTGTGGATGGCAGGCAGGCCTATATTTCTTGGTTTCGATAGGACTCCTTACGAGCGTTGTTTCTATCTACTATTATCTAAAAATAATCAAGTTATTAATGACTGGGCGAAACCAAGAAATAACCCCTCACGTGCGAAATTATAGAAGATCTCCCTTAAGATCAAACAATTCCATCGAATTGAGTATGACTGTATGTGTGATAGCATCTACTATACTAGGAATATCAATGAACCCAATTCTTGCAATTGCTCAGGATACCCTCTTTTAGCTTCTAGGTCTATTTTTTAGTTCAAGATCCCTTTTACTAACTGGAATAAAAGAATTACTTTTCCGCCCAAAATGGGAATGGGCTGGAGTTATGAACTTATAATCATGGAATCGACTCGATCATCAGATTATAAGTTCATTCCAGACCGGACCAGGCCGGAATAGGGTTATGTACATTCTTATTATGAGTAAGGGGTCATTCGAGCGTATGTAAATAGATACTCTGTTTACATATGGATCCCTACGTCGTTACATTCTATTTAGGATTAGGAATAGGCGTAATCGGACCCGCTTTTGACATATCTATCGTTATTTGGGTACCCTATTCACTTCTTTGGGCTTCTCTTGAATCGAGAAAGAGGTTTGATTTTACATCTTTTTGATATATAGTTTAATATATAGAAGGTATCCTCCGGATAATTCCAATCGAAGCAATTTGATGTTTGACTCGGGCCTATATGACATGACCGATCGATAGAAATACTCCAACACTCCACCTTTGTCATATATTCCATATATCACACTAGATAGATATCATATTCATGGAATACGATTCACTTTCAAGATGCCTTGATGGTGAAATGGTAGACACGCGAGACTCAAAATCTCGTGCTAAAGAGCGTGGAGGTTCGAGTCCTCTTCAAGGCATAATATTGAGAATGCTTATTTAATGAGCAATTCCATAACAGATTTCGGATCTAATCAATATTGACGAGTATCTTATCTGTTGATACGAAGTATTCCGGCGATCCTCACGATCCGAGTCCGAGCTGTTGGAATCGGCAGCGGATCACGAAATCTTGGCGATCTTATCTATACTCTATAGGAGTCCGTTTGGAAATCGTCCGCCCTGCGCGCACCCCCCGAGTATAGGATTCAACAGGAATCGCACAAGGTAGATTGATAGAAAAACCTCTGGTAAAATACCCACCAGTACCCGTAACCCAGCAAAGAAAGTACATTACATTAGGGATTGGCGACTTACCCATTCAGTGACTTTGGCACTGGACGTTCTCAAAATGGGTACTATCGGGTCGGGTGAATTAGAGAATAGACAGACGTCTGTTGGCATTCCAGCCTTCCTTCTCCTTTCAGGGCCTATCCGAAAGAGGCTCTTGGTCGTGAATATCTGAATAGGACGAACCCGCCTCCGTGGATATCTTTGCTTCGGAACAAAGCAATTAGAATTAGGCTCGGTCAACTGGAATGTGTATTATCCGTATGGGAGATCTTCCAATTGAGGAGATCCATCGACCTGAGACGAAGAGAAAGGTCTATCTATCTTCTTTAGTTATTCAATGAAACCAATGATTCGTTATTGGAGCAAATAGCAACAACCATTTCAGCGGACATGCGTATTTTCCGATGGATTTACATGGTTTCATTAGTATAAATTGTTTGATGTAGCGAGTAATAGGCTCTTTCGCCGTTCAAGAATTCTTGTTTAAGCAGTTCATATCATCCACACATAGTGATCTAAGATTTCAATTCTTCCATGTTTCAGCAGTAGCATATTGTTCCACGGAGTTAAGGCCAAAAAGATGGAATAAACAAGTGTTTCCACGACTCTACCATCCGGCCAATTCTGTTCCACTGAATCCCCTTTTCATGGGCACATATCTTTACGGCTAAGGAATGGGGAATCTTTCTCCTGTTACATGAATCAAATGTTTCATTTCATCCGGGAAAAGCCATCTCTTTCTCAACAATGTCTTTGTCATTTGATCCAATAGCGTTCCGTTAGATAGGAACAGATTTGATAAATACTGATAACTCTCGGATAGAGTATTAGAACGGAAAGGTCCATTAGATAATGAACTATGGGTTCTAAACCATCTCTGGCGATGAATCAACAATTCGAAGTGCTTTTCTTGCGTATTCTTGATGAACCAGCGTTTATATATAGATGTAGGAGGATTTGTTTGGGAAGCAATAAGCCCCTTTGACATCTCTTCATCTGCAAAGAATTCTCGACGTGAAAACACAGAGACAGAGGGCTGATCTTTGAATAGGGAAAAGAATGGATCCGCAGGGTCCCAAATGAATTGGCTTGTTCGAAAAAAGCCTTGTTCTTTGGAAGATCTATCTCGTGTCTGGTACTGCATGGTTCCACTCTGCAAGAACTCCGAATCATTCTCTTGAAGCTCATCCTCTTTATGATAAATGATCCATTTGCCCCGAAATGACCCAGTCCAATAGGGAAATCCCAATTCAGTGGGCCTTTCGATACAATCAAATAGATTAGGGCGCCATATTCTAGGAGCCCAAACTATGTGATTGAATAAATCCTCCTCTATCTGTTGCGGATCGAGGGCCCCTTCCCCTTCTTCAAACTCCGATTCGTATTTTTCATATAGAAATCTCTGATCAACGATAGAACAAGATCCATTTTGCATCATATCTAACTGATTCCTTGGTTCGGACCGAAGAAGTAATGTCACTCGATTATTATCAAACTGACTGCAATATTTTTCTGTCCGTGAGGATCCCGCCAGAGCCAGAGTGCCTTCTACTTCTACTTCTAATAGTAATAATAGTAATAGGCCATGAACTAGATCAGAATCATTCTCAACGAATCCATAAGAAGTGATCCAATTTTTTTCATCGTGTCTGGATGAAGACCAAAGATCTTGAGCGACCGATCCGGCAGAACAACTCAAAAGATAAAGAAGTATCGTTAATTTCTTCATGCTCGTTCCAAGTTCGAAGTACCATTTGTACAAATAAGAATCCCCTCCCTTACATGATCTCTTCTTCATATAGATAGATATAGGATCTATGGGGCAATTACTTAGAAGTACATTTTGTGTAACAGCCCTTCCTATCTGATAGAAAAGGATCCCATGATCCTGAACCGATCTTATCTGGGATCGAAAATCCCAAGTTTTTCTATGAAGAGCTGATCTAATTGTATTAGTTTCTATAATGGATTTCTTCTGTGTAATACTAATTGATAGGGCCTCATTGATAAGTGCTACAAGATCTCGCGCATTGGAACCCATGGTTATGGACCCGAATCCGTTAGTATGGAACATCTTCTTTTCCAAGTGAAATCCCCTAGTATATGAAAGAATTAAAAAGTGCTTTCGTTGTTGTGGAAGAAGAAGCCTTCGTATCTTAATGCATGTATTTAATTTATTCGGAGCTATTAGAGCGGGATCCACTTTTTGGGGAATATGAGTCGAAGCAATAACAAGAATCTTTCCAGTGGAACATCTTTCACAATCCCTGGAGAGTAATAGACCGAGGGATAAGTAATTCGACTCATTCACATGCAGATCATGAATGTTTGGAATCCATATTATGCAAGGAGACATTGCTTTTGCTAATTCGAATTGAAGGGTGATATCAAATAGGTCTATTTTCGGCGTCATATACATAGTTAGCACATTCGTCATAGTTAGCAGCTCCGTATCAATATCAAGGTCATCATCAATATCGTCACTATCATCAATATCGTCACTATCATCAATATCGATATCATCAATAAGATAACCTTTAGGCTTGTCATCCAGGAACTTGTTCGGAAATACCGTAATGAAAGGAACATAGGAGTTTGTCGCTAGGTATTTGACCAAACAGGATCGTCCAGTTCCTATAGAACCTATCACTAAAATACCTCTAGAAGGGGATAGGGCTAAGCGGAGCGAAAAGGGTTTTCCATGAGGAGATGGGGAATGAAAACTATTAGCCCCACACGAGGCTTGTGAATAAGTGATTGTCTGATAATGAGCAAGGAATATCCGTCTTTCTGCTAAACAGGATCTATTGAACTCATAATTCATTAGATCCTTTTGATGAATGTCAACTAAGTATCGTAAGGAACCCGGTTGTTCAATCATTTGATAACCAGAGTCATTCTTTGATAAATGATCACTATGAGTCAGACTCAATAGAATTTGATCAATCCTTTTTTCTGTCGTTAAGGTGGAGAACTGAACCAAGAATTCTCTTTCTTCATCATCAATCGAATCACTGTTCGCGACCCAGAATTCTATTTTCTCATCAATCCAATCACCGTTCACGTTTTTTCTTTTTCTTATCAATGAATAGATCTCTTTACTTGTACGACTTAGATGTCTCGTATTTCTCGAAAAAATGATTCGATTGATGGGATTTGGTATGATACTTACAAGATCGATGAGATTGATCTTCCAATATTTCTTCTTAGAACGTATTGATTTGACCCCATAAGCGGGACCACCACCCAATAGCATGTTGCCACCAGAAGCAGAACCCCGTATTTCTTCCAGAGAATCTCCTAATTGTTCCAGAACAACTAGAAAGAGATTCTTTAACCAGAAAGGATTCAGTTCAGATGTAGGATACCTATCCAGAAGTTTTCGAAATTCCATCATGTATGATGGAATCATCAAAGATTTGATCTTTTCTAACTCTGTCTGTAACTCACTAGAGGCTCGGGAAACAAAGAGAAGATGTATACGAACGAGATATCCAGCAACAAGAAGAAGGAAAAAGATGGAATAGAGGAACTCCCGAGCATTTGTTGATCTCAGATGTGCCCATATCAATGGAACGGGTGACTCATTATTTCGATGAATCATTTCTTCGGACAGAAGAAGATTCTGTAAACATTGACTCGAAATCTCATGTGGAAGAATCTTCTGAGGAATTGGCCGTGATATATCTGATCCATGCATCATATCATGAAAAATGGATACAAATTTTTGACTACTACTCAGTATCGGCAATGGGTCTGAAAGAGTATCTAAAAGGGTGAAATTGAGATATTTGCACCCTGTCGAAGTAAGGAACCATGGCATATATGTTTGGAACAGATTCCATTTTGAGAGATTTGAAAAAGCACTATCTCGTTGAAAGGTTCTATACATCTGCCCTTTCTCAACGCATTTATTTAGACAAAGACTCCGTTTTTTCCTCTTTCCGGATGGTAAATACTTCTCAGAACATGGAGTGTGAATCAAACCCATGTTTGAATTGAAACTGAGATACTGATGCAAGTTATTCCCTTCTGAATCAGATAGATTCATATCTGAAAGAGGCTGACAATAAGTTTTTTCCAAATTGACTATTTGTTCCTCTGTTAGAGGTGTTGCAGAAATGTCTGCGATCGAGTAAATAGCTCCACGAACGAATGGATCGGATCGAATTGGAAAATGGAAAGATTTGTACAATTTGTACAAGTTATACGTTTCATCACCACTTTGTGGGAAATCGTTAGGTATGAAGATGTCAGATACCTGTGACTCGATTGGTGAAATAGTCTCTCTCTCCAAAAAAAGGTATTTTTTTTTACCGACGCACAAAGAAAAGATTTTGTTGCGAATGGACAAGATATTGAGGAATTGTCCATATGTAAGATCATAATTATTGATACGGGTCTTTTCCACATCAAAGGGGAATCTTTTGTTACAATAGAACCAGAAGTGATGTGGATCATTCAAGAATCGAAGTCGATTTGCTTTATAAAAAGAAGATATCAATGAACTTCTATTAAATGGTTTCACGGGATTCAGCCAATTGTCTCGATCGTGGGATATCATTGAGAAATAGGAATCCTTGTTATCAAAGGATTTCCTGCGATTCTTTCTAGTATGGAATGAGTCAATCATCCGCTTTGGTATCTTTTTGAACAAAAATGGTAATATTGTTCCTCCATTGATCAAGAATTTTGGTCTTTGGGAAGTATCATGATCATCCAATAAGAAGGGTTTCAATTTCTTCAAATGAACGATTTGAACACCTATGGATTCTAACAACTGATTGCAGAGTTGATCATTCGGACCTTTCAATTCATAGATGTGGATCTCGGACCTATGAATGGGGATATTCCCGATATTCACAAAGAAAAAGGGAAGTGACTTGGACAAAAAGAAACGAAGTGACTTAGACAAATCTTCTTTGTCGATAGCCTCGGACCAATCAATCGAATATTGATTAATACGTAATCGATCGAACACTACTTGCACTACTTGAAAAGGATTCTTCTGTTCAGAAACGAAATGTTCCAAATGTTCCTGGAAATTCTTGCTCCCATTGGACCATTTGTATCTATATGCATCAGGATCCCGATTCATGGATCTCTCAGTTCGAGAAATAAGAGGATCAAACCATTTCTTCTGACTCTTTTTCAAATTCGATAAATGTTGGTTGATCGTATATTTCATTATAGTTATATGATTCAGAGTATCATTTCCTATTTGATCCCTTTGAATTCCATATTCGAAGTTGCGATCGGATCTATTCATTAAAAAGAATCGATCCGATACATTTCTTCCATAGGTGCTATATTGGATTTGAATCAGATTTCGGATCAATCTATATTGATTGACTGCCTCCATTATGTTGTTGCTAGCAAATACCGCTGTTTTTAGTTTGGGATCTTCCAACTCATTCCCGCAGTAGATCCGGACCGATTTTTTTCTGATCCTTCGAGAAAAAGATTCATTCTCCTCATAAAAAAGAGGAGGTAGAACCAATAAAGATTTCTTTTTCGATTCATCTCTGGAGTTGAATACCTCATTCAAGAATTTTTTTTGATCCAACCCGTAGGAATCAATAGAAAAGGCAAATCCCCTATGAAACACCAGATCCGGCTCGGTTATTGATAGAGTGAATAGATCCGCCATTTCTGGGAATCTCTCTTCTGATTCAAAAAAATCGTGGCGTAACGCGTATCCCCCTTTGTTCCGGTCATGGAATAGATGAAAGAAATCAAAAAATGGATTTTTGTTCAAGAATGAAATCTTATTGGAACTGTCCATATCCGGTTCATCCTTCGGAACCAGATCCGGGATGTGATCTGGTTCCGAAGGATGAATTGAGACGGTATCTTGTAAATACGTAATTATCTTGAATATATCAACCATTTCTTTATTTTCCGCTCGCCTGGAAGGGACAAAAGAAACATCTTGTTTTTTCTTCAACAATTTATGATCTCTAGTGGACCTCTCAGTAGGATTCGAACCCAGATGAAGTTCTGACCATCTGTCAGAGAAAAAAGAACGAATTGATCTTGTAGGATTCCCAAGAAATTCTTCGATTTCTTCCGGAAGCAGATGATTATTCATCCGCTTCTCAGGTTCCGTGAATAGCCAGGACATGGAGGAAGATCCAAAAAGGCATTTCGGGAATCGATCTGATTCTATCTCTGTTCGTTCCGTTTGAAGAAAGGAAGGATCCCAAAGAATCGATCTCTCTTTTAGTTGCTGAATCTCTGTTTGATCGATCAATGTGTGATATTCTGAATTCTCATTTCTAACGGAATCGAAATGATCTCTGGATTGATCAGAAGAAGATCCTTTCCATTGGCTAGAATCCGTTACTTGAACGAAAATAGATCTTGTGGAATCATATTGAATATTTGACAATACATTCCGTACCTTGCTAAAAAACCGATCCTTGTTTACCAACCACACATTGTCTAACCAAATCCAATTCTCTCTCGAGACGTTCCTCAAAAAATTCGATTCGTGCTGATTCTTCCCCCAACTAACGAAGAGATCTTGGCGGAATTGCCACATATGAAATTGAGCACAATTTTGCAAAGAAATACTCCACTTGTTTCTCGAGAAGAGATGGGAAACATGCTCAATATCATTGGATTGCATAGTTGCCCCAGCTCCTTGTTGTTTGAAGAAACTCTCCCCCTGAATTGGTCTTTTTTCACGAAAAGCAGACATGAGATAACAAATCAAGTCTTTCCCTAAGATTTCGAATAGCTGTCCCGAATTCAAGTTGATTATGTTTCGTTTCTTCCTCGGAGAAAGACGATCAAAAAATTCCCAATCATGGTCCTTGCGGATCGGATCATCCGTATAGGATAAAAAAAGAAACTCCAGATATTTGCTATCTTTCTCTTTGAATGAGATCTCAATTCCAGCTACGGTTTCATTAGATATCTGACAACTAGAATCCCTCTTTTTTCCGATCCGGTTCCTCCACCACCGCGAACCCCGGTTAGATTCAGGCATGATACGCTTTTTCTTTATTGGGATAACCCAAGTACTCTCTTGCGGATCCATGAAACAACTCTCAGAAATCTTTTTCCCTTTTGGAAGATACAGGAGCGAAACAATCAACCTATTTCTATTGGAAGACCAAAAAGATTCTTCCAATGTCTCCTTTCTGGGTCCAATGGAATTCATAGGTATAGGAAGAAGCCCCATCAAATAGAGATTTTTTCTTTCGACCATCTTTCGATTGTTAATACGAGATATAAGGACCACTACTACAAGCAGTACTACACCTTTGGTCGTGAAATATCGATTGCTTGTTGCACCCTGTGAATCACGTGAAAGTAGGATACTCCAAATTCGGGGGTCAAAGAGTTTCATAAAACGTTCTTGGTGGAAAAAAATGTGAGTGAAAGATCCCACTGAATCGAATTTGATCCATGAATCTAAGAAATAGTGAGAATTCTTGATCTCTCTCAATTCGAATATCCAGGATTTTAATTGATGTCGTTTCATTGATTCCTCCTAAAGATTTCATTTCAATTGGAATTTGGTTATTCACGATGTACGATGATCCCCGTTAAGCATCCATGGCTGAATGGTTAAAGCGCCCAACTCATAATTGGCAAATTCGTAGGTTCAATTCCTGCTGGATGCACGCCAATGGGAACATTCAATAAGTCTATTGGAATTGGCTCTGTATCAATGGAATCTCATCATCCATACATAGCGAATTGGTATGGTATATTCATACCATAACATATGAACAGTAAGAACTAGAATTCTTAGGGAAGAAAATGGATTTATGGATGGAATCAAATATGCAGTATTTACAGAAAAAAGTATTCGGTTATTGGGGAACAATCAATATACTTCTAATGTCGAATCAGGATCAACTAGGACAGAAATAAAGCATTGGGTCGAACTCTTCTTTGGTGTCAAGGTAATAGCTATGAATAGTCATCGACTCCCGGGAAAGGGTAGAAGGATGGGACCTATTATGGGACATACAATGCATTACAGACGTATGATCATTACGCTTCAACCGGGTTATTCTATTCCACCTCTTATAGAGAAAAGAACTTAAAGCAAAAGACTTAATAACACGGCAATACATTTATACAAAACTTCTACCCCGAGCACACGCAATGGAGCCGTAGACAGTCAAGTGAAATCCAATCCACGAAATAATTTGATCTATGGACAGCATCGTTGTGGTAAAGGTCGTAATGCCAGAGGGATCATTACCGCAGGGCATAGAGGGGGAGGTCATAAGCGTCTATACCGTAAAATCGACTTTCGACGGAATGAAAAAGAGATATCTGGTAGAATCGTAACCATAGAATATGACCCTAATCGAAATGCATACATTTGTCTCATACACTATGGGGATGGTGAGAAGAGATATATTTTACATCCCAGAGGGGCTATAATTGGAGATACCATTGTTTCTGGTACAGAAGTTCCTATATCAATGGGAAATGCCCTACCTTTGAGTGCGGTTTGAACTATTGATTTACGTAATTGGAAGTAACCAATTAGGTTTACGACGAAACCTAGAAATCGATCACTGATCCAATTGGAGTACCTCTACGGGATAGACCTCAACAGAAAACTGTTGAGTAACGGCAGCAAGTGATTGAGTTCAGTAGTTCCTCATAGAAAATTATTGACTCTAGAGATATGGTAATATGGAGAAGACAAAATTGTTTGAAGCGCGCACAGAACCGGAAGCACCCCTTGTTTCAAAGAGAGGAGGACGGGTTATTCACATTTCATTTGATGGTCAGAGGCGAATTGAAAGCTAAGCAGTGGTAATTAGAAAGACCCCCCGGGGAAAAATAGAGATGTCTCCTACGTTACCCGTAATATGTGGAAGTATCGACGTAATTTCATAGAGTCATCCGGTCTGAATGCTACATGAAGAACATAAGCCAGATGACGGAACGGGGAGACCTAGGATGTAGAAGATCATAACATGAGTGATTCGGCAGATTTGGATTCCTATATATCCACTCATGTGGTACTTCATCATATAAGATCCATCTGTCTAGATATCATCATATACATCTAGAAAGCCGTATGCTTTGGAAGAAGCTTGTACAGTTTGGGAAGGGGTTTTGATTGATAAAAAAGAAGAATCTACTTCAACCGATATGCCCTTAGGCACGGCCATACATAACATAGAAATCACACTTGGAAAGGGTGGACAATTAGCTAGAGCAGCAGGCGCTGTAGCGAAACTGATTGCAAAAGAGGGTAAATCGGCCACATTAAGATTACCATCTGGGGAGGTCCGTTTGATATCCAAAAACTGCTTAGCAACAGTCGGACAAGTGGGTAATGTTGGGGTGAACCAAAAAAGTTTGGGTAGAGCTGGATCTAAGTGTTGGCTAGGTAAGCGTCCTGTAGTAAGAGGAGTAGTTATGAACCCTGTAGACCATCCCCATGGGGGCGGTGAAGGGAGAGCCCCAATTGGTAGAAAAAAACCCACAACCCCTTGGGGTTATCCTGCGCTTGGAAGAAGAAGTAGGAAAAGGAACAAATATAGTGATAGTTTTATTCTTCGTCGCCGTAAATAGGAACATTGAAAATCGAATTTTTGGAATTGGAAATAATGTGATGGGCGAACGACGGGAATTGAACCCGCGCATGGTGGATTCACAATCCACTGCCTTGATCCACTTGGCTACATCCGCCCCTTCCCTTATCTAGCTAAAGGATTTTCTCTTTTTTCCATTCATCATTATACTTCAGATTAAGATCGAGATATTGGACATAGAATGCCAATTTTAAAAATGTAAAAAAAGGAGTAATCAGCCGTGACACGTTCACTAAAAAAAAATCCTTTTGTAGCTAATCATTTATCGGGAAGAATTGAAAAACTCAACAGGAGGGAGGAGAAAGAAATCATAGTGACTTGGTCTCGGGCATCTACCATTATACCCACAATGATTGGCCATACAATCGCTATTCATAATGGAAAGGAACATTTACCTATTTATATCACAGATCGTATGGTCGGTCACAAATTGGGAGAATTCGCACCTACTCTCACTTTCGTGAGACACGCGAGAAACGATAATAAATCTCGTCGTTAGCCGTTCTACTAAGTATTCATGTGAAAAGCCTTATCTTAACTAGTAAGAGTATAGGTATATTTCTTTTCTTTTTCTAGTATACTAATAAGACTTATACTTTTCTGACTTATCTTATACTATACTTCACCTAGGCACTTATCATTCATTGGCGGGGGA

>Phaius_tankervilliae
[truncated: 475,216 more chars]
